# *Batrachochytrium salamandrivorans*: The North American Response and a Call for Action

**DOI:** 10.1371/journal.ppat.1005251

**Published:** 2015-12-10

**Authors:** Matthew J. Gray, James P. Lewis, Priya Nanjappa, Blake Klocke, Frank Pasmans, An Martel, Craig Stephen, Gabriela Parra Olea, Scott A. Smith, Allison Sacerdote-Velat, Michelle R. Christman, Jennifer M. Williams, Deanna H. Olson

**Affiliations:** 1 Center for Wildlife Health, University of Tennessee, Knoxville, Tennessee, United States of America; 2 Amphibian Survival Alliance, Austin, Texas, United States of America; 3 Association of Fish and Wildlife Agencies, Washington, D.C., United States of America; 4 Department of Environmental Science and Policy, George Mason University, Fairfax, Virginia, United States of America; 5 Department of Pathology, Bacteriology and Avian Diseases, Faculty of Veterinary Medicine, Ghent University, Merelbeke, Belgium; 6 Canadian Wildlife Health Cooperative, Saskatoon, Saskatchewan, Canada; 7 Instituto de Biología, Universidad Nacional Autónoma de México, México City, México; 8 Maryland Department of Natural Resources, Wye Mills, Maryland, United States of America; 9 Lincoln Park Zoo, Chicago, Illinois, United States of America; 10 New Mexico Ecological Services Field Office, U.S. Fish and Wildlife Service, Albuquerque, New Mexico, United States of America; 11 Partners in Amphibian and Reptile Conservation, Ft. Collins, Colorado, United States of America; 12 Pacific Northwest Research Station, U.S. Forest Service, Corvallis, Oregon, United States of America; Geisel School of Medicine at Dartmouth, UNITED STATES

## Introduction

*Batrachochytrium salamandrivorans* (*Bsal*) is an emerging fungal pathogen that has caused recent die-offs of native salamanders in Europe and is known to be lethal to at least some North American species in laboratory trials [1]. *Bsal* appears to have originated in Asia, and may have been introduced by humans into wild populations in Europe through commercial trade of amphibians [1]. Since the first outbreaks of *Bsal* in the Netherlands, it has been the etiologic agent of mortality events in Belgium (wild) and Germany (captivity), and was recently found in imported salamanders in the United Kingdom [1–4]. Substantial concern has been raised about the associated risk of *Bsal* to native salamanders in North America [5]. Herein, we review what policy actions are occurring in North America and elsewhere, and call for creation of a North American *Bsal* Strategic Plan.

## How Does *Bsal* Kill Its Host and Differ from *B*. *dendrobatidis (Bd)*?

**Fig 1:**
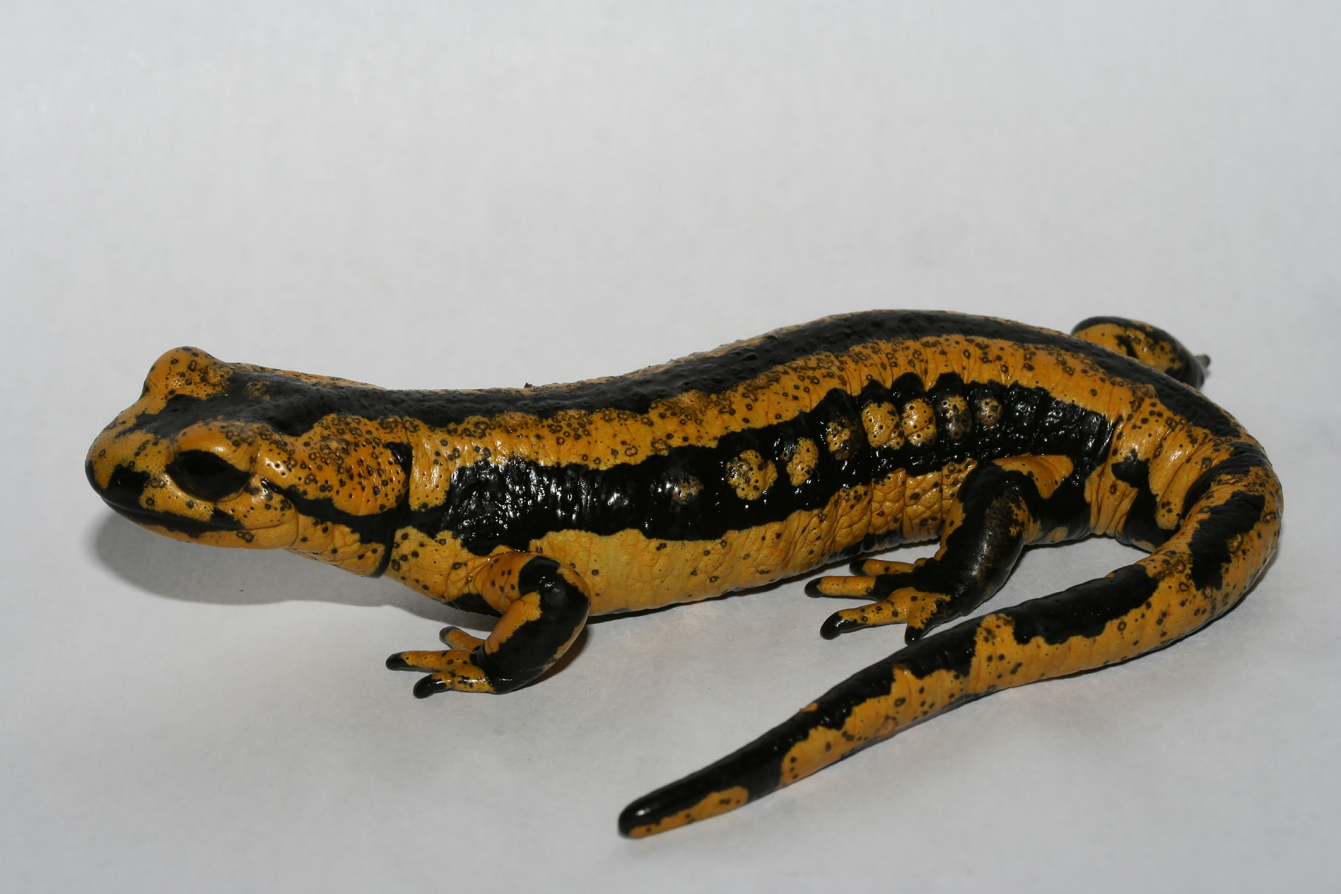
Fire salamander (*Salamandra salamandra*) covered with *Bsal* ulcerations, which are visible as black spots (photo credit = F. Pasmans).

**Fig 2:**
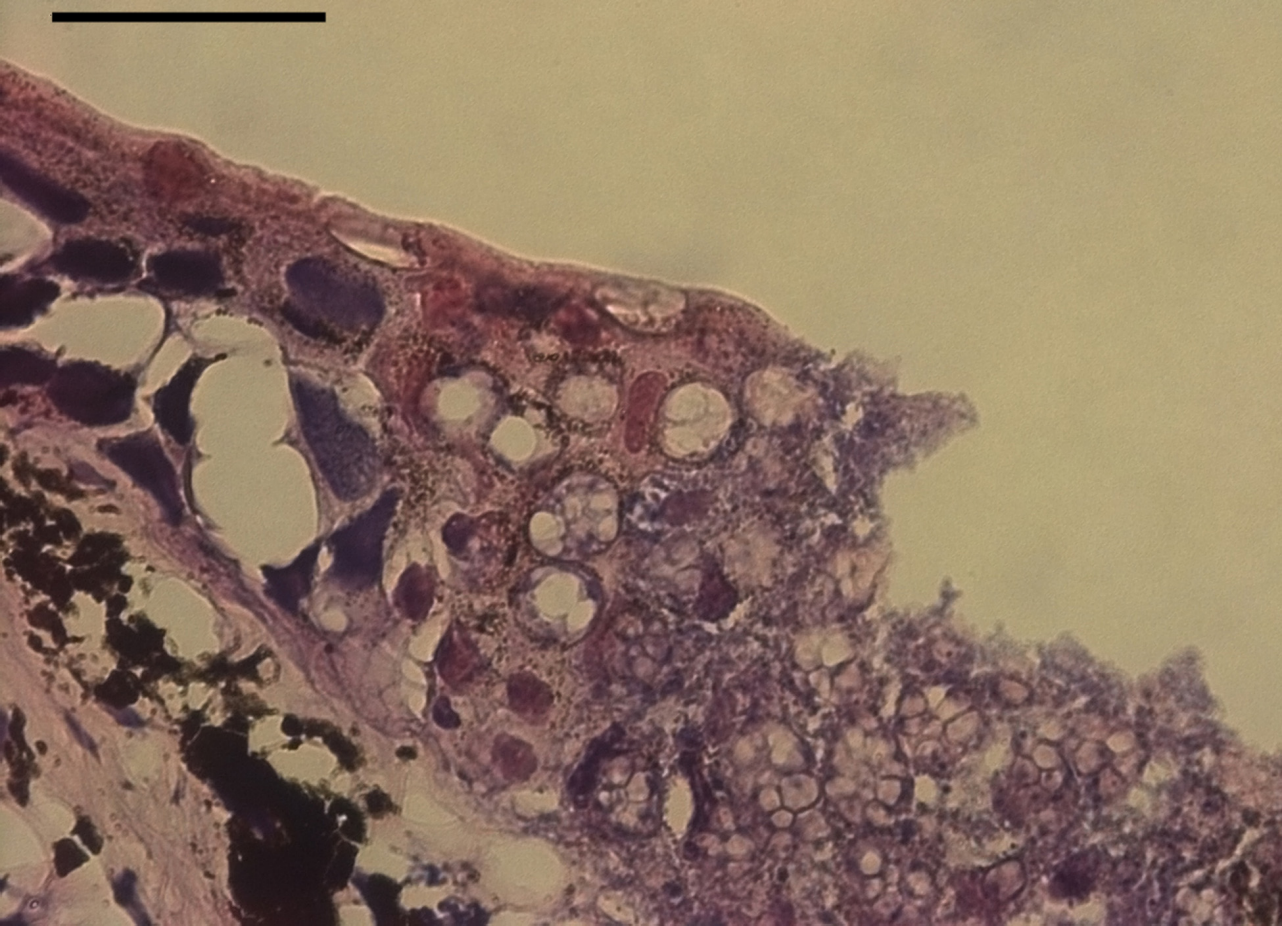
*Bsal* infection in the skin of a fire salamander (*Salamandra salamandra*), characterized by extensive epidermal necrosis, presence of high numbers of intra-epithelial colonial chytrid thalli, and loss of epithelial integrity (H&E staining, scale bar = 50 μm; photo credit = A. Martel and F. Pasmans).

*Bsal* parasitizes the epidermal cells of salamanders (order Urodela), causing skin ulcerations with significant degradation of the epidermis, which is sometimes visible macroscopically (Fig 1) and very obvious histologically (Fig 2). Loss of epidermal integrity with subsequent impairment of vital skin functions (e.g., electrolyte homeostasis, fluid balance, gas exchange, barrier against opportunistic pathogens) leads to death in susceptible species within two to three weeks after exposure [1,2]. Death is generally preceded by a brief episode of abnormal body posture and behavior. Species susceptibility correlates with the ability of *Bsal* to invade the epidermis, and is species- and developmental-stage–dependent [6]. Whereas some species succumb quickly to chytridiomycosis after *Bsal* infection, others have been shown to tolerate and eventually clear infection, suggesting the development of acquired immunity [1]. Although experimental exposure to *Bsal* zoospores leads to mortality in a wide range of salamander species, mortality events in wild salamander populations have been reported only in a single species (fire salamander, *Salamandra salamandra*). Mortality events in other species may have gone unnoticed due to the secretive nature of salamanders.

Like its sister species, *B*. *dendrobatidis* (*Bd*), *Bsal* infects the epidermal cells of amphibian skin; however, *Bd* appears to be more pathogenic to frogs (order Anura), whereas *Bsal* seems to be more pathogenic to salamanders. The clinical signs of infection for both *Bd* and *Bsal* are excessive skin shedding, lethargy, anorexia, abnormal posture, and death. However, the lesions produced by each fungus are different. While *Bd* mainly causes epidermal hyperplasia and hyperkeratosis [7,8], but only rarely skin ulcerations [9], *Bsal* typically causes skin ulcerations with significant destruction of the epidermis. Modes of *Bsal* transmission are unknown, but probably include direct contact between individuals and exposure to contaminated water or soil, similar to *Bd* [10].

## Why Do We Care?

North America is a global hotspot for salamander biodiversity, accounting for about 50% of species worldwide [11]. In particular, Mexico and the Appalachian Mountains are collectively home to more than 100 species of lungless salamanders (family Plethodontidae). Both of these areas, along with the Pacific Northwest, are known for their regionally endemic and relictual salamander species. In North American forests, the biomass of salamanders can exceed the biomass of all other vertebrate species [12,13]. Salamanders are centrally nested in aquatic and terrestrial food webs, as predators of various insects (including hosts of human pathogens [14]) and prey for higher-order predators such as reptiles, birds, and mammals (e.g., [15]). Indeed, salamanders are vital components of ecosystems, significantly affecting various ecological processes, energy flow, and trophic-level interactions, which ultimately contributes to environmental quality.

Salamanders not only perform significant ecological functions, but also provide a variety of ecosystem services for human benefit [16]. In addition to their aesthetic value and use as pets and educational tools, salamanders serve as metrics of biotic integrity and play a role in carbon cycling [13,17,18], which helps buffer climate change. Additionally, many salamander species have biomedical value, with the ability to regenerate limbs [19] and skin that produces chemicals with antibiotic, anesthetic, and analgesic properties [20]. Salamanders also are used as models to understand animal physiology [21]. Due to its apparent pathogenicity to many salamander taxa, if *Bsal* is introduced into North America, it could have serious ecological and economic impacts, including potential extinction of species. Salamander communities in the southern Appalachian Mountains, southeastern and northwestern United States, southwestern Canada, and central Mexico may be at greatest risk [11], yet salamanders in any location could be vulnerable to *Bsal*, and increased awareness overall is warranted.

## Past Lessons and Initial Responses

Introduced pathogens have significant impact on native wildlife. The related amphibian fungal pathogen, *Bd*, has had dire effects on its hosts worldwide [22]. The pathogen that causes white-nose syndrome, *Pseudogymnoascus destructans*, recently introduced from Europe to North America, has decimated many bat populations [23]. The chestnut blight fungus, *Cryphonectria parasitica*, introduced to North America from Asia in the early 1900s, caused the functional extinction of the American chestnut tree (*Castanea dentata*), forever changing eastern North American forest ecosystems. As with many invasive species, what we have learned from the emergence of these fungal pathogens in North America is that preventing introduction is the best way to protect populations, and if introduction occurs, rapid response is essential [24].

**Fig 3:**
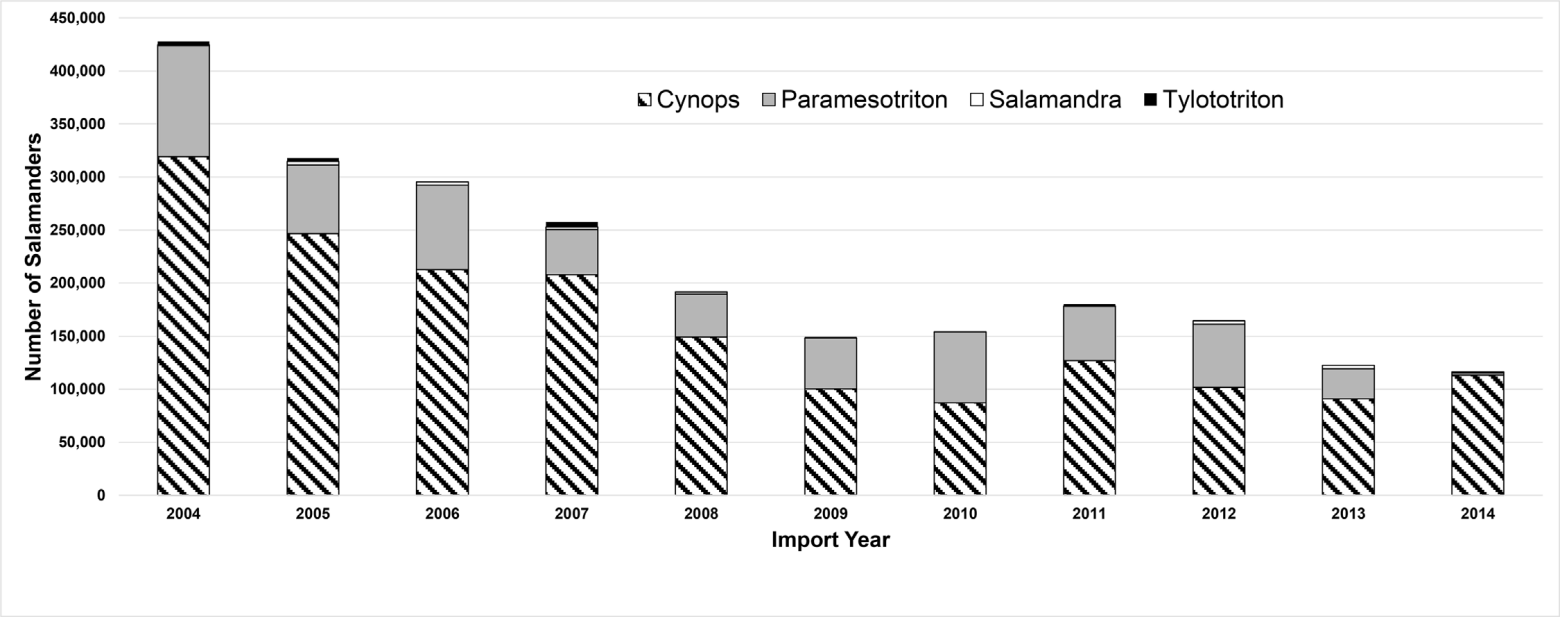
Number of live salamanders from four genera (*Cynops*, *Paramesotriton*, *Salamandra*, *Tylototriton*) imported into the US from 2004 to 2014 (USFWS Law Enforcement Management Information System [LEMIS]); these genera comprise 95% of all legally traded salamander imports.

Recognizing the threat of *Bsal* to salamander species across the globe, a coalition of organizations and individuals submitted letters to the US Fish and Wildlife Service (USFWS) requesting that the agency take emergency action to prevent the spread of *Bsal* into the US. Current USFWS regulations allow them to impose import restrictions on animal species that may be injurious to native species, but not microorganisms such as pathogens. Additionally, although animal health certificates are required by the US Department of Agriculture for imported domesticated animals that are hosts of pathogens listed as notifiable by the World Organisation for Animal Health (OIE), evidence of pathogen-free shipments is not required for imported wildlife. These policy gaps have created challenges in efforts to reduce the risk of *Bsal* introduction into the US. Currently, the USFWS, working under the authority of the Lacey Act (18 U.S.C. 42), is considering listing salamander species that could be hosts of *Bsal*, as injurious. Salamanders represented 5.5% of the amphibians imported into the US from 2004 to 2014, and 95% of those belong to four genera: *Cynops*, *Paramesotriton*, *Salamandra*, and *Tylototriton* (Fig 3). These genera contain at least one species known to be susceptible to *Bsal* infection [1]. *Cynops* and *Paramesotriton* comprise more than 90% of U.S. imported salamanders; hence, these genera may be the greatest threat. Chinese newts (*Pachytriton*) comprise approximately 4.5% of live salamanders imported into the US (S1 Table); their susceptibility to *Bsal* has not been tested [1]. We estimated the total market value was US$924,707 for salamanders imported into the US in 2014 if all animals imported that year were sold at the median market value (Table 1).

**Table 1:** Estimated annual value (USD) of salamanders imported into the US based on 2014 imports (see S1 Table) and a range of market values (low, median, and high).

Genus	Number Imported	Low Price	Median Price	High Price	Low Value	Median Value	High Value
*Cynops*	113,187	$4	$7	$10	$452,748	$792,309	$1,131,870
*Pachytriton*	2,908	$10	$15	$20	$29,080	$43,620	$58,160
*Paramesotriton*	2,536	$10	$15	$20	$25,360	$38,040	$50,720
*Salamandra*	1,027	$20	$32.5	$45	$20,540	$33,378	$46,215
*Tylototriton*	434	$30	$40	$50	$13,020	$17,360	$21,700
				Total:	$540,748	$924,707	$1,308,665

If *Bsal* arrives in the US, several partners are working together to outline appropriate actions. Partners in Amphibian and Reptile Conservation (PARC) formed a National Disease Task Team in January 2015, with one of the initial objectives to help facilitate the development of a strategic plan for *Bsal*. The US Geological Survey (USGS) held a *Bsal* workshop in June 2015, with the goal of developing an objective decision-making process to guide *Bsal* response actions. Twenty-nine professionals from four countries with expertise in disease ecology and natural resource management participated in the USGS workshop. A key outcome was the organization of a *Bsal* National Task Force for the US, which is composed of a Technical Advisory Committee (TAC) and seven working groups. The working groups are composed of experts focused on priority topics: (1) response (to *Bsal* detection); (2) surveillance and monitoring; (3) research; (4) diagnostics; (5) decision support; (6) data management; and (7) communication and outreach. The working groups are developing products that are intended to become part of a larger *Bsal* strategic plan. Chairpersons of the working groups are members of the TAC, which meets monthly to share progress on various assigned tasks. Professionals interested in contributing to *Bsal* working group tasks can contact one of the TAC co-chairs (Deanna Olson, US Forest Service; Jennifer Ballard, USFWS).

Others are also responding to the threat of *Bsal* in North America. For example, the PARC National Disease Task Team is assembling a regional list of professionals in the US to contact if an amphibian mass mortality event is encountered and disease suspected. This list will help formalize disease response and enable enhanced communication with *Bsal* working groups who can provide guidance on response procedures and post-outbreak monitoring actions. The Amphibian Survival Alliance (ASA), which holds a seat on the TAC, is taking on ancillary tasks, such as leading development of a *Bsal* website to disseminate information produced by the *Bsal* National Task Force and others as it develops. Amphibiaweb.org hosts a website on *Bsal* (http://amphibiaweb.org/chytrid/Bsal.html) and is currently developing a global *Bsal* reporting portal inspired by the Global Ranavirus Reporting System (https://mantle.io/grrs) and *Bd*-maps (http://www.bd-maps.net; M. Koo, University of California–Berkeley, personal communication). University of California–Berkeley also created a LISTSERV for *Bsal* postings (bsal@lists.berkeley.edu); subscribing can be done at https://calmail.berkeley.edu/manage/list/listinfo/bsal@lists.berkeley.edu. Smaller regional efforts are occurring, too. For example, PARC and the ASA hosted a *Bsal* meeting in Asheville, North Carolina, US, in August 2015 to inform and engage regional biologists and the public. An outcome of this meeting was the creation of a southern Appalachian *Bsal* task force, which is organized by Caleb Hickman (Eastern Band of the Cherokee Indians).

State, provincial, and territorial fish and wildlife agencies in the US and Canada have been engaging with *Bsal* through standing committees of the Association of Fish and Wildlife Agencies (AFWA) to recommend policy actions based on the risk to native salamanders. AFWA is also working with the TAC to develop a *Bsal* rapid response plan that can be customized by local, state, or federal management entities. Along with the ASA, AFWA is collaborating with various nongovernmental and commercial industry partners, as well as with contacts in the US Congress, to examine various policy options and solutions.

The Canadian government is actively working to reduce the risk of *Bsal* introduction through import control. Environment Canada is exploring emergency measures similar to those being considered in the US to prevent entry of the pathogen. The Canadian Wildlife Health Cooperative recently identified diagnostic laboratories capable of testing for *Bsal* infection in Canada, and is leading efforts for national surveillance of the pathogen and outreach education to increase awareness. Policy responses to *Bsal* have been slow in Mexico; however, scientists in Mexico and the US are collaborating in laboratory experiments to test susceptibility of their native salamander species to *Bsal*. Researchers in all three countries are independently testing wild and captive animals for *Bsal* as part of ongoing pathogen surveillance studies. Clearly, a collaborative, trilateral approach to *Bsal* surveillance, research, and response is essential to ensure salamander resources in North America are protected.

In Europe, policy actions are limited to individual European states. In Belgium and the Netherlands, abatement plans are being developed, mainly focused on raising public awareness and developing emergency action plans. Recently, the Swiss Federal Food Safety and Veterinary Office established a ban on the importation of all salamander species into Switzerland (B. Schmidt, KARCH and University of Zurich, personal communication). The only European transnational initiative currently consists of a draft recommendation on prevention and control of *Bsal*, which will be proposed to the Standing Committee of the Bern Convention in December 2015. This recommendation stresses the importance of transnational and coordinated actions to limit spread and impact of *Bsal* in Europe, but also to prevent introduction into naïve regions such as North America. We are unaware of any additional countries currently involved in policy actions to prevent the spread of *Bsal*.

## What Can We Do in North America?

Due to the potential threat of *Bsal* to North American salamanders, creation of a North American Strategic Plan for *Bsal* is warranted. Several good examples exist of strategic plans for wildlife diseases, such as for white-nose syndrome [25] and *Bd* [26]. At a minimum, components should include:

- identification of possible routes of *Bsal* entry into the US, Canada, and Mexico;
- strategies to prevent or reduce the risk of *Bsal* entry into the US, Canada, and Mexico;
- surveillance and biosecurity strategies in the wild, the pet trade, and zoological facilities;
- diagnostic assays, reference laboratories, and approaches for confirmation of positive samples;
- response and disease intervention strategies if *Bsal* is detected in North America; and
- development of an information portal for communication, outreach, and education.

Additionally, a prioritized list of essential research directions is needed. Some urgent research directions include: (1) estimating susceptibility of North American amphibian species to *Bsal*; (2) determining the most efficient modes of *Bsal* transmission; (3) identifying minimum concentrations of standard disinfectants to inactivate *Bsal*; (4) validating *Bsal* diagnostic procedures; and (5) determining the interactive effects of *Bsal* with stressors and other pathogens.

## Conclusions

All evidence suggests that we are at a critical time of action to protect global amphibian biodiversity by swift policy actions to prevent the translocation of *Bsal* (Box 1). *Bsal*’s potential effects are broad taxonomically, geographically, ecologically, and across a variety of ecosystem services. Hence, response to the threat of *Bsal* calls for a cooperative effort across nongovernmental organizations, government agencies, academic institutions, zoos, the pet industry, and concerned citizens to avoid the potential catastrophic effects of *Bsal* on salamanders outside of the pathogen’s endemic regions. Communication, collaboration, and expedited action are key to ensure that *Bsal* does not become established in North America and decimate wild salamander populations. The template developed for North America may inform similar strategic policy planning for *Bsal* elsewhere.

**Box 1. A North American Call for Action against *Bsal***

                We know *Bsal* was likely introduced to Europe,

        and is negatively impacting native salamanders there [1,2].

            We know *Bsal* is lethal to many salamander species [1].

                    We know there is support for policy action

    among governmental, nongovernmental, and industry partners.

            We know timely preventative actions can reduce

the risk of catastrophic losses of North American salamanders due to *Bsal* [24].

The question remains: “Will sufficient policy action occur before it is too late?”

## Supporting Information

S1 Table Number of live salamanders imported by taxa into the USA, 2004 to 2014 (source = USFWS LEMIS). USFWS LEMIS data (S1 Table) for live salamander imports into the US available at: http://www.amphibians.org/resources/tradedata/. Data requested in May 2015 under the US Freedom of Information Act.
(PDF)
