# Supplementary material for: Batrachochytrium salamandrivorans: The North American Response and a Call for Action
Source: PLoS Pathog. 2015 Dec 10;11(12):e1005251. doi: 10.1371/journal.ppat.1005251 (PMC4675546; doi:10.1371/journal.ppat.1005251)
Supplement: S1 Table — USFWS LEMIS data (S1 Table) for live salamander imports into the US available at: http://www.amphibians.org/resources/tradedata/. Data requested in May 2015 under the US Freedom of Information Act. (PDF) [file ppat.1005251.s001.pdf]

Table S1. Number of live salamanders imported by taxa into the USA, 2004 – 2014 (source =USFWS LEMIS).

Control Number	Species Code	Genus	Species	Subspecies	Specific Name	Generic Name	Wildlfe Desc	Qty	Unit	Ctry Org	Ctry IE	Purp	Src	Act	Dp Cd	Disp Date	Ship Date	I E	Pt Cd	U.S.Importer/ Exporter
2004313917	CYOR	CYNOPS	ORIENTALIS		CHINESE DWARF	NEWT	LIV	250	NO	HK	HK	T	C	C	C	1/3/04	1/3/04	I	LA	DOLPHIN INTERNATIONAL
2004313917	PACB	PACHYTRITON	BREVIPES		TSITOU	NEWT	LIV	100	NO	HK	HK	T	C	C	C	1/3/04	1/3/04	I	LA	DOLPHIN INTERNATIONAL
2004313948	CYOR	CYNOPS	ORIENTALIS		CHINESE DWARF	NEWT	LIV	4,400	NO	HK	HK	T	C	C	C	1/4/04	1/4/04	I	LA	DOLPHIN INTERNATIONAL
2004313948	PACB	PACHYTRITON	BREVIPES		TSITOU	NEWT	LIV	100	NO	HK	HK	T	C	C	C	1/4/04	1/4/04	I	LA	DOLPHIN INTERNATIONAL
2004313962	TRIH	TRITURUS	HONGKONGENSIS		ALPINE	NEWT	LIV	450	NO	HK	HK	T	W	C	C	1/4/04	1/4/04	I	LA	AQUACO, INC.
2004313974	TRIT	TRITURUS	SPECIES			NEWT	LIV	1,500	NO	HK	HK	T	C	C	C	1/4/04	1/4/04	I	LA	GLOBAL AQUATIC CONSULTING
2004315354	CYOR	CYNOPS	ORIENTALIS		CHINESE DWARF	NEWT	LIV	250	NO	HK	HK	T	C	C	C	1/10/04	1/10/04	I	LA	DOLPHIN INTERNATIONAL
2004315368	CYOR	CYNOPS	ORIENTALIS		CHINESE DWARF	NEWT	LIV	3,500	NO	CN	CN	T	W	C	C	1/10/04	1/10/04	I	LA	Z IMPORTS INC.
2004315441	CYOR	CYNOPS	ORIENTALIS		CHINESE DWARF	NEWT	LIV	400	NO	HK	HK	T	C	C	C	1/11/04	1/11/04	I	LA	DOLPHIN INTERNATIONAL
2004315441	PACB	PACHYTRITON	BREVIPES		TSITOU	NEWT	LIV	100	NO	HK	HK	T	C	C	C	1/11/04	1/11/04	I	LA	DOLPHIN INTERNATIONAL
2004315451	TRIT	TRITURUS	SPECIES			NEWT	LIV	1,300	NO	HK	HK	T	C	C	C	1/11/04	1/11/04	I	LA	GLOBAL AQUATIC CONSULTING
2004315465	TRIH	TRITURUS	HONGKONGENSIS		ALPINE	NEWT	LIV	500	NO	HK	HK	T	W	C	C	1/11/04	1/11/04	I	LA	AQUACO, INC.
2004316081	BODO	BOLITOGLOSSA	DOFLEINI		ALTA VERAPAZ	SALAMANDER	LIV	8	NO	HN	HN	T	W	C	C	1/13/04	1/12/04	I	LA	LA REPTILES INC
2004319231	TRIH	TRITURUS	HONGKONGENSIS		ALPINE	NEWT	LIV	200	NO	HK	HK	T	W	C	C	1/18/04	1/18/04	I	LA	AQUACO, INC.
2004319251	TRIT	TRITURUS	SPECIES			NEWT	LIV	250	NO	HK	HK	T	C	C	C	1/18/04	1/18/04	I	LA	TROPICAL ENTERPRISES
2004319260	TRIT	TRITURUS	SPECIES			NEWT	LIV	67	NO	CN	CN	T	C	C	C	1/18/04	1/18/04	I	LA	AQUATIC CONNECTION
2004319262	TRIT	TRITURUS	SPECIES			NEWT	LIV	200	NO	HK	HK	T	C	C	C	1/18/04	1/18/04	I	LA	GLOBAL AQUATIC CONSULTING
2004319275	CYOR	CYNOPS	ORIENTALIS		CHINESE DWARF	NEWT	LIV	1,750	NO	HK	HK	T	C	C	C	1/18/04	1/18/04	I	LA	DOLPHIN INTERNATIONAL
2004319275	PACB	PACHYTRITON	BREVIPES		TSITOU	NEWT	LIV	200	NO	HK	HK	T	C	C	C	1/18/04	1/18/04	I	LA	DOLPHIN INTERNATIONAL
2004323741	TRIH	TRITURUS	HONGKONGENSIS		ALPINE	NEWT	LIV	300	NO	HK	HK	T	W	C	C	1/25/04	1/25/04	I	LA	AQUACO, INC.
2004323755	TRIT	TRITURUS	SPECIES			NEWT	LIV	1,400	NO	HK	HK	T	C	C	C	1/25/04	1/25/04	I	LA	GLOBAL AQUATIC CONSULTING
2004323777	CYOR	CYNOPS	ORIENTALIS		CHINESE DWARF	NEWT	LIV	100	NO	HK	HK	T	C	C	C	1/25/04	1/25/04	I	LA	DOLPHIN INTERNATIONAL
2004323777	PACB	PACHYTRITON	BREVIPES		TSITOU	NEWT	LIV	100	NO	HK	HK	T	C	C	C	1/25/04	1/25/04	I	LA	DOLPHIN INTERNATIONAL
2004328715	PACB	PACHYTRITON	BREVIPES		TSITOU	NEWT	LIV	200	NO	HK	HK	T	C	C	C	1/31/04	1/31/04	I	LA	DOLPHIN INTERNATIONAL
2004328752	CYOR	CYNOPS	ORIENTALIS		CHINESE DWARF	NEWT	LIV	1,800	NO	HK	HK	T	C	C	C	2/1/04	2/1/04	I	LA	DOLPHIN INTERNATIONAL
2004328752	PACB	PACHYTRITON	BREVIPES		TSITOU	NEWT	LIV	200	NO	HK	HK	T	C	C	C	2/1/04	2/1/04	I	LA	DOLPHIN INTERNATIONAL
2004328778	TRIH	TRITURUS	HONGKONGENSIS		ALPINE	NEWT	LIV	275	NO	HK	HK	T	W	C	C	2/1/04	2/1/04	I	LA	AQUACO, INC.
2004328789	TRIT	TRITURUS	SPECIES			NEWT	LIV	30	NO	HK	HK	T	C	C	C	2/1/04	2/1/04	I	LA	TROPICAL ENTERPRISES
2004328790	TRIT	TRITURUS	SPECIES			NEWT	LIV	1,600	NO	HK	HK	T	C	C	C	2/1/04	2/1/04	I	LA	GLOBAL AQUATIC CONSULTING
2004334245	TRIH	TRITURUS	HONGKONGENSIS		ALPINE	NEWT	LIV	200	NO	HK	HK	T	W	C	C	2/8/04	2/8/04	I	LA	AQUACO, INC.
2004334263	TRIT	TRITURUS	SPECIES			NEWT	LIV	200	NO	HK	HK	T	C	C	C	2/8/04	2/8/04	I	LA	GLOBAL AQUATIC CONSULTING
2004334269	CYOR	CYNOPS	ORIENTALIS		CHINESE DWARF	NEWT	LIV	900	NO	HK	HK	T	C	C	C	2/8/04	2/8/04	I	LA	DOLPHIN INTERNATIONAL
2004334269	PACB	PACHYTRITON	BREVIPES		TSITOU	NEWT	LIV	300	NO	HK	HK	T	C	C	C	2/8/04	2/8/04	I	LA	DOLPHIN INTERNATIONAL
2004339695	CYOR	CYNOPS	ORIENTALIS		CHINESE DWARF	NEWT	LIV	200	NO	HK	HK	T	C	C	C	2/14/04	2/14/04	I	LA	DOLPHIN INTERNATIONAL
2004339756	CYOR	CYNOPS	ORIENTALIS		CHINESE DWARF	NEWT	LIV	300	NO	HK	HK	T	C	C	C	2/15/04	2/15/04	I	LA	DOLPHIN INTERNATIONAL
2004339756	PACB	PACHYTRITON	BREVIPES		TSITOU	NEWT	LIV	500	NO	HK	HK	T	C	C	C	2/15/04	2/15/04	I	LA	DOLPHIN INTERNATIONAL
2004339794	TRIH	TRITURUS	HONGKONGENSIS		ALPINE	NEWT	LIV	250	NO	HK	HK	T	W	C	C	2/15/04	2/15/04	I	LA	AQUACO, INC.
2004339795	TRIT	TRITURUS	SPECIES			NEWT	LIV	340	NO	HK	HK	T	C	C	C	2/15/04	2/15/04	I	LA	GLOBAL AQUATIC CONSULTING
2004344600	CYOR	CYNOPS	ORIENTALIS		CHINESE DWARF	NEWT	LIV	250	NO	HK	HK	T	C	C	C	2/21/04	2/21/04	I	LA	DOLPHIN INTERNATIONAL
2004344657	TRIT	TRITURUS	SPECIES			NEWT	LIV	200	NO	HK	HK	T	C	C	C	2/21/04	2/22/04	I	LA	GLOBAL AQUATIC CONSULTING
2004344677	CYOR	CYNOPS	ORIENTALIS		CHINESE DWARF	NEWT	LIV	700	NO	HK	HK	T	C	C	C	2/21/04	2/22/04	I	LA	DOLPHIN INTERNATIONAL
2004344677	PACB	PACHYTRITON	BREVIPES		TSITOU	NEWT	LIV	200	NO	HK	HK	T	C	C	C	2/21/04	2/22/04	I	LA	DOLPHIN INTERNATIONAL
2004344696	TRIH	TRITURUS	HONGKONGENSIS		ALPINE	NEWT	LIV	200	NO	HK	HK	T	W	C	C	2/22/04	2/22/04	I	LA	AQUACO, INC.
2004349564	CYOR	CYNOPS	ORIENTALIS		CHINESE DWARF	NEWT	LIV	600	NO	HK	HK	T	C	C	C	2/28/04	2/28/04	I	LA	DOLPHIN INTERNATIONAL
2004349564	PACB	PACHYTRITON	BREVIPES		TSITOU	NEWT	LIV	200	NO	HK	HK	T	C	C	C	2/28/04	2/28/04	I	LA	DOLPHIN INTERNATIONAL
2004349579	CYOR	CYNOPS	ORIENTALIS		CHINESE DWARF	NEWT	LIV	200	NO	CN	CN	T	W	C	C	2/28/04	2/28/04	I	LA	Z IMPORTS INC.
2004349601	CYOR	CYNOPS	ORIENTALIS		CHINESE DWARF	NEWT	LIV	5,100	NO	HK	HK	T	C	C	C	2/28/04	2/29/04	I	LA	DOLPHIN INTERNATIONAL
2004349627	TRIH	TRITURUS	HONGKONGENSIS		ALPINE	NEWT	LIV	225	NO	HK	HK	T	W	C	C	2/29/04	2/29/04	I	LA	AQUACO, INC.
2004349638	TRIT	TRITURUS	SPECIES			NEWT	LIV	2,490	NO	HK	HK	T	C	C	C	2/29/04	2/29/04	I	LA	GLOBAL AQUATIC CONSULTING
2004349641	TRIT	TRITURUS	SPECIES			NEWT	LIV	50	NO	HK	HK	T	C	C	C	2/29/04	2/29/04	I	LA	TROPICAL ENTERPRISES
2004355572	CYOR	CYNOPS	ORIENTALIS		CHINESE DWARF	NEWT	LIV	4,000	NO	CN	CN	T	W	C	C	3/6/04	3/6/04	I	LA	Z IMPORTS INC.
2004355610	CYOR	CYNOPS	ORIENTALIS		CHINESE DWARF	NEWT	LIV	7,350	NO	HK	HK	T	C	C	C	3/7/04	3/7/04	I	LA	DOLPHIN INTERNATIONAL
2004355617	TRIT	TRITURUS	SPECIES			NEWT	LIV	250	NO	HK	HK	T	C	C	C	3/7/04	3/7/04	I	LA	TROPICAL ENTERPRISES
2004355651	TRIH	TRITURUS	HONGKONGENSIS		ALPINE	NEWT	LIV	675	NO	HK	HK	T	W	C	C	3/7/04	3/7/04	I	LA	AQUACO, INC.
2004355658	TRIT	TRITURUS	SPECIES			NEWT	LIV	2,300	NO	HK	HK	T	C	C	C	3/7/04	3/7/04	I	LA	GLOBAL AQUATIC CONSULTING
2004355857	PRAN	PROTEUS	ANGUINUS			OLM	LIV	1	NO	SI	SI	S	C	C	C	1/22/04	1/22/04	I	SF	NAME REMOVED

2004361266	CYOR	CYNOPS	ORIENTALIS	CHINESE DWARF	NEWT	LIV	250	NO	HK	HK	T	C	C	C	3/13/04	3/13/04	I	LA	DOLPHIN INTERNATIONAL
2004361304	CYOR	CYNOPS	ORIENTALIS	CHINESE DWARF	NEWT	LIV	4,950	NO	HK	HK	T	C	C	C	3/14/04	3/14/04	I	LA	DOLPHIN INTERNATIONAL
2004361314	TRIH	TRITURUS	HONGKONGENSIS	ALPINE	NEWT	LIV	325	NO	HK	HK	T	W	C	C	3/14/04	3/14/04	I	LA	AQUACO, INC.
2004361339	TRIT	TRITURUS	SPECIES		NEWT	LIV	2,600	NO	HK	HK	T	C	C	C	3/14/04	3/14/04	I	LA	GLOBAL AQUATIC CONSULTING
2004364764	CYOR	CYNOPS	ORIENTALIS	CHINESE DWARF	NEWT	LIV	100	NO	HK	HK	T	C	C	C	3/27/04	3/27/04	I	LA	DOLPHIN INTERNATIONAL
2004364764	PACB	PACHYTRITON	BREVIPES	TSITOU	NEWT	LIV	200	NO	HK	HK	T	C	C	C	3/27/04	3/27/04	I	LA	DOLPHIN INTERNATIONAL
2004364806	CYOR	CYNOPS	ORIENTALIS	CHINESE DWARF	NEWT	LIV	4,100	NO	HK	HK	T	C	C	C	3/27/04	3/28/04	I	LA	DOLPHIN INTERNATIONAL
2004364806	PACB	PACHYTRITON	BREVIPES	TSITOU	NEWT	LIV	200	NO	HK	HK	T	C	C	C	3/27/04	3/28/04	I	LA	DOLPHIN INTERNATIONAL
2004364829	TRIH	TRITURUS	HONGKONGENSIS	ALPINE	NEWT	LIV	475	NO	HK	HK	T	W	C	C	3/28/04	3/28/04	I	LA	AQUACO, INC.
2004364840	TRIT	TRITURUS	SPECIES		NEWT	LIV	1,600	NO	HK	HK	T	C	C	C	3/28/04	3/28/04	I	LA	GLOBAL AQUATIC CONSULTING
2004365795	CYOR	CYNOPS	ORIENTALIS	CHINESE DWARF	NEWT	LIV	200	NO	CN	CN	T	F	C	C	2/12/04	2/4/04	I	NY	MIN HUA ENTERPRISE, INC.
2004366740	CYOR	CYNOPS	ORIENTALIS	CHINESE DWARF	NEWT	LIV	200	NO	CN	CN	T	F	C	C	2/26/04	2/18/04	I	NY	MIN HUA ENTERPRISE, INC.
2004366781	CYOR	CYNOPS	ORIENTALIS	CHINESE DWARF	NEWT	LIV	60	NO	HK	HK	T	W	C	C	2/19/04	2/14/04	I	NY	TRANSSHIP DISCOUNTS , LTD.
2004366781	PACB	PACHYTRITON	BREVIPES	TSITOU	NEWT	LIV	30	NO	HK	HK	T	W	C	C	2/19/04	2/14/04	I	NY	TRANSSHIP DISCOUNTS , LTD.
2004366781	CYOR	CYNOPS	ORIENTALIS	CHINESE DWARF	NEWT	LIV	60	NO	HK	HK	T	W	C	C	2/19/04	2/14/04	I	NY	TRANSSHIP DISCOUNTS , LTD.
2004366787	TRIH	TRITURUS	HONGKONGENSIS	ALPINE	NEWT	LIV	84	NO	HK	HK	T	C	C	C	2/3/04	2/2/04	I	NY	TRI-COUNTY TROPICALS, INC.
2004367227	SASA	SALAMANDRA	SALAMANDRA	EUROPEAN FIRE	SALAMANDER	LIV	20	NO	DE	DE	T	C	C	C	3/30/04	3/30/04	I	DF	U.S. GLOBAL EXOTICS, INC.
2004367310	CYPY	CYNOPS	PYRRHOGASTER	JAPANESE FIREBELLY	NEWT	LIV	675	NO	HK	HK	T	W	C	C	1/20/04	1/17/04	I	NY	TRANSSHIP DISCOUNTS , LTD.
2004367492	TRIH	TRITURUS	HONGKONGENSIS	ALPINE	NEWT	LIV	400	NO	HK	HK	T	W	C	C	1/11/04	1/11/04	I	NY	INTERNATIONAL PET RESOURCES LLC
2004367630	CYPY	CYNOPS	PYRRHOGASTER	JAPANESE FIREBELLY	NEWT	LIV	370	NO	HK	HK	T	W	C	C	2/3/04	1/31/04	I	NY	ERDA INC.
2004367702	CYPY	CYNOPS	PYRRHOGASTER	JAPANESE FIREBELLY	NEWT	LIV	50	NO	HK	HK	T	W	C	C	2/10/04	2/7/04	I	NY	TRANSSHIP DISCOUNTS , LTD.
2004367709	CYPY	CYNOPS	PYRRHOGASTER	JAPANESE FIREBELLY	NEWT	LIV	130	NO	HK	HK	T	W	C	C	2/3/04	1/31/04	I	NY	TRANSSHIP DISCOUNTS , LTD.
2004367772	CYPY	CYNOPS	PYRRHOGASTER	JAPANESE FIREBELLY	NEWT	LIV	180	NO	HK	HK	T	W	C	C	1/26/04	1/24/04	I	NY	TRANSSHIP DISCOUNTS , LTD.
2004367867	CNP?	CYNOPS	SPECIES	FIREBELLY	NEWT	LIV	165	NO	HK	HK	T	W	C	C	3/31/04	3/31/04	I	LA	U.S. GLOBAL EXOTICS, INC.
2004367867	CYOR	CYNOPS	ORIENTALIS	CHINESE DWARF	NEWT	LIV	968	NO	HK	HK	T	W	C	C	3/31/04	3/31/04	I	LA	U.S. GLOBAL EXOTICS, INC.
2004367867	PCHI	PARAMESOTRITON	CHINENSIS	CHINESE WARTY	NEWT	LIV	193	NO	HK	HK	T	W	C	C	3/31/04	3/31/04	I	LA	U.S. GLOBAL EXOTICS, INC.
2004367916	CYPY	CYNOPS	PYRRHOGASTER	JAPANESE FIREBELLY	NEWT	LIV	75	NO	HK	HK	T	W	C	C	1/12/04	1/10/04	I	NY	ERDA INC.
2004368169	CYPY	CYNOPS	PYRRHOGASTER	JAPANESE FIREBELLY	NEWT	LIV	400	NO	CN	CN	T	W	C	C	2/20/04	2/15/04	I	NY	FISH MART INC.
2004368319	CYPY	CYNOPS	PYRRHOGASTER	JAPANESE FIREBELLY	NEWT	LIV	70	NO	HK	HK	T	W	C	C	2/24/04	2/21/04	I	NY	TRANSSHIP DISCOUNTS , LTD.
2004368357	CYPY	CYNOPS	PYRRHOGASTER	JAPANESE FIREBELLY	NEWT	LIV	470	NO	HK	HK	T	W	C	C	1/12/04	1/10/04	I	NY	TRANSSHIP DISCOUNTS , LTD.
2004368439	CYPY	CYNOPS	PYRRHOGASTER	JAPANESE FIREBELLY	NEWT	LIV	400	NO	CN	CN	T	C	C	C	1/13/04	1/11/04	I	NY	FISH MART INC.
2004368835	CYPY	CYNOPS	PYRRHOGASTER	JAPANESE FIREBELLY	NEWT	LIV	200	NO	HK	HK	T	W	C	C	1/26/04	1/24/04	I	NY	ERDA INC.
2004369432	CYPY	CYNOPS	PYRRHOGASTER	JAPANESE FIREBELLY	NEWT	LIV	100	NO	HK	HK	T	W	C	C	2/24/04	2/21/04	I	NY	ERDA INC.
2004369612	CYOR	CYNOPS	ORIENTALIS	CHINESE DWARF	NEWT	LIV	200	NO	CN	CN	T	F	C	C	1/12/04	1/7/04	I	NY	MIN HUA ENTERPRISE, INC.
2004369622	CYPY	CYNOPS	PYRRHOGASTER	JAPANESE FIREBELLY	NEWT	LIV	100	NO	HK	HK	T	W	C	C	1/5/04	1/3/04	I	NY	ERDA INC.
2004369903	TRIH	TRITURUS	HONGKONGENSIS	ALPINE	NEWT	LIV	200	NO	HK	HK	T	C	C	C	1/6/04	1/5/04	I	NY	TRI-COUNTY TROPICALS, INC.
2004369974	CYOR	CYNOPS	ORIENTALIS	CHINESE DWARF	NEWT	LIV	100	NO	HK	HK	T	C	C	C	4/3/04	4/3/04	I	LA	DOLPHIN INTERNATIONAL
2004370030	CYOR	CYNOPS	ORIENTALIS	CHINESE DWARF	NEWT	LIV	3,700	NO	HK	HK	T	C	C	C	4/3/04	4/4/04	I	LA	DOLPHIN INTERNATIONAL
2004370030	PACB	PACHYTRITON	BREVIPES	TSITOU	NEWT	LIV	400	NO	HK	HK	T	C	C	C	4/3/04	4/4/04	I	LA	DOLPHIN INTERNATIONAL
2004370031	TRIT	TRITURUS	SPECIES		NEWT	LIV	250	NO	HK	HK	T	C	C	C	4/3/04	4/4/04	I	LA	TROPICAL ENTERPRISES
2004370032	TRIT	TRITURUS	SPECIES		NEWT	LIV	1,200	NO	HK	HK	T	C	C	C	4/3/04	4/4/04	I	LA	GLOBAL AQUATIC CONSULTING
2004370053	TRIH	TRITURUS	HONGKONGENSIS	ALPINE	NEWT	LIV	250	NO	HK	HK	T	W	C	C	4/4/04	4/4/04	I	LA	AQUACO, INC.
2004373005	SASA	SALAMANDRA	SALAMANDRA	EUROPEAN FIRE	SALAMANDER	LIV	20	NO	ES	DE	T	C	C	C	4/7/04	4/6/04	I	DF	U.S. GLOBAL EXOTICS, INC.
2004373021	SASA	SALAMANDRA	SALAMANDRA	EUROPEAN FIRE	SALAMANDER	LIV	225	NO	UA	UA	T	C	C	C	2/19/04	2/19/04	I	MI	NAME REMOVED
2004374525	TRIT	TRITURUS	SPECIES		NEWT	LIV	1,600	NO	HK	HK	T	C	C	C	1/26/04	1/28/04	I	TP	5-D TROPICAL, INC.
2004374584	TRIT	TRITURUS	SPECIES		NEWT	LIV	1,600	NO	HK	HK	T	C	C	C	3/8/04	3/10/04	I	TP	5-D TROPICAL, INC.
2004375200	CYOR	CYNOPS	ORIENTALIS	CHINESE DWARF	NEWT	LIV	3,700	NO	HK	HK	T	C	C	C	4/10/04	4/11/04	I	LA	DOLPHIN INTERNATIONAL
2004375200	PACB	PACHYTRITON	BREVIPES	TSITOU	NEWT	LIV	200	NO	HK	HK	T	C	C	C	4/10/04	4/11/04	I	LA	DOLPHIN INTERNATIONAL
2004375216	TRIT	TRITURUS	SPECIES		NEWT	LIV	125	NO	HK	HK	T	C	C	C	4/11/04	4/11/04	I	LA	TROPICAL ENTERPRISES
2004375220	TRIT	TRITURUS	SPECIES		NEWT	LIV	2,000	NO	HK	HK	T	C	C	C	4/11/04	4/11/04	I	LA	GLOBAL AQUATIC CONSULTING
2004375221	TRIH	TRITURUS	HONGKONGENSIS	ALPINE	NEWT	LIV	475	NO	HK	HK	T	W	C	C	4/11/04	4/11/04	I	LA	AQUACO, INC.
2004375319	TRIT	TRITURUS	SPECIES		NEWT	LIV	25	NO	SG	SG	T	W	C	C	3/11/04	3/11/04	I	CH	INTERCONTINENTAL IMPORTS, INC.
2004376567	TRIT	TRITURUS	SPECIES		NEWT	LIV	25	NO	HK	HK	T	W	C	C	3/21/04	3/21/04	I	LA	NAME REMOVED
2004376670	CYOR	CYNOPS	ORIENTALIS	CHINESE DWARF	NEWT	LIV	3,000	NO	CN	CN	T	W	C	C	3/20/04	3/20/04	I	LA	Z IMPORTS INC
2004376695	TRIT	TRITURUS	SPECIES		NEWT	LIV	1,000	NO	HK	HK	T	C	C	C	3/21/04	3/21/04	I	LA	GLOBAL AQUATIC CONSULTING
2004377222	TRIT	TRITURUS	SPECIES		NEWT	LIV	600	NO	HK	HK	T	W	C	C	3/21/04	3/21/04	I	LA	AQUACO, INC.
2004379765	PACB	PACHYTRITON	BREVIPES	TSITOU	NEWT	LIV	100	NO	HK	HK	T	C	C	C	4/17/04	4/17/04	I	LA	DOLPHIN INTERNATIONAL
2004379780	CYOR	CYNOPS	ORIENTALIS	CHINESE DWARF	NEWT	LIV	5,800	NO	CN	CN	T	W	C	C	4/17/04	4/17/04	I	LA	Z IMPORTS INC.
2004379808	TRIT	TRITURUS	SPECIES		NEWT	LIV	1,200	NO	HK	HK	T	C	C	C	4/18/04	4/18/04	I	LA	GLOBAL AQUATIC CONSULTING
2004379810	TRIH	TRITURUS	HONGKONGENSIS	ALPINE	NEWT	LIV	200	NO	HK	HK	T	W	C	C	4/18/04	4/18/04	I	LA	AQUACO, INC.

2004379822	TRIT	TRITURUS	SPECIES		NEWT	LIV	60	NO	HK	HK	T	C	C	C	4/18/04	4/18/04	I	LA	TROPICAL ENTERPRISES
2004379834	TRIT	TRITURUS	SPECIES		NEWT	LIV	600	NO	HK	HK	T	C	C	C	4/18/04	4/18/04	I	LA	AQUATIC CONNECTION
2004379838	CYOR	CYNOPS	ORIENTALIS	CHINESE DWARF	NEWT	LIV	3,950	NO	HK	HK	T	C	C	C	4/18/04	4/18/04	I	LA	DOLPHIN INTERNATIONAL
2004383545	CYPY	CYNOPS	PYRRHOGASTER	JAPANESE FIREBELLY	NEWT	LIV	200	NO	HK	HK	T	W	C	C	3/24/04	3/20/04	I	NY	ERDA INC.
2004383590	TRIH	TRITURUS	HONGKONGENSIS	ALPINE	NEWT	LIV	100	NO	HK	HK	T	W	C	C	3/10/04	2/28/04	I	NY	CAUMSETT STATE PARK
2004383599	CYPY	CYNOPS	PYRRHOGASTER	JAPANESE FIREBELLY	NEWT	LIV	510	NO	HK	HK	T	W	C	C	3/24/04	3/20/04	I	NY	TRANSSHIP DISCOUNTS , LTD.
2004383955	CYPY	CYNOPS	PYRRHOGASTER	JAPANESE FIREBELLY	NEWT	LIV	400	NO	CN	CN	T	W	C	C	3/18/04	3/14/04	I	NY	FISH MART INC.
2004384143	CYPY	CYNOPS	PYRRHOGASTER	JAPANESE FIREBELLY	NEWT	LIV	220	NO	HK	HK	T	W	C	C	3/16/04	3/13/04	I	NY	ERDA INC.
2004384207	TRIT	TRITURUS	SPECIES		NEWT	LIV	60	NO	HK	HK	T	C	C	C	4/25/04	4/25/04	I	LA	TROPICAL ENTERPRISES
2004384227	TRIT	TRITURUS	SPECIES		NEWT	LIV	1,200	NO	HK	HK	T	C	C	C	4/26/04	4/25/04	I	LA	GLOBAL AQUATIC CONSULTING
2004384239	CYOR	CYNOPS	ORIENTALIS	CHINESE DWARF	NEWT	LIV	10,150	NO	HK	HK	T	C	C	C	4/26/04	4/25/04	I	LA	DOLPHIN INTERNATIONAL
2004384453	CYPY	CYNOPS	PYRRHOGASTER	JAPANESE FIREBELLY	NEWT	LIV	244	NO	HK	HK	T	W	C	C	3/1/04	2/28/04	I	NY	ERDA INC.
2004384492	CYOR	CYNOPS	ORIENTALIS	CHINESE DWARF	NEWT	LIV	200	NO	CN	CN	T	F	C	C	2/2/04	1/14/04	I	NY	MIN HUA ENTERPRISE, INC.
2004384497	CYOR	CYNOPS	ORIENTALIS	CHINESE DWARF	NEWT	LIV	200	NO	CN	CN	T	F	C	C	2/2/04	2/2/04	I	NY	MIN HUA ENTERPRISE, INC.
2004384564	CYPY	CYNOPS	PYRRHOGASTER	JAPANESE FIREBELLY	NEWT	LIV	320	NO	HK	HK	T	W	C	C	3/1/04	2/28/04	I	NY	TRANSSHIP DISCOUNTS , LTD.
2004384861	CYOR	CYNOPS	ORIENTALIS	CHINESE DWARF	NEWT	LIV	200	NO	CN	CN	T	C	C	C	3/3/04	2/25/04	I	NY	MIN HUA ENTERPRISE, INC.
2004385153	CYPY	CYNOPS	PYRRHOGASTER	JAPANESE FIREBELLY	NEWT	LIV	570	NO	HK	HK	T	W	C	C	4/6/04	4/3/04	I	NY	TRANSSHIP DISCOUNTS , LTD.
2004385623	TRIH	TRITURUS	HONGKONGENSIS	ALPINE	NEWT	LIV	600	NO	HK	HK	T	W	C	C	3/24/04	3/8/04	I	NY	ALL AMERICAN TRANSHIP
2004385672	CYOR	CYNOPS	ORIENTALIS	CHINESE DWARF	NEWT	LIV	200	NO	CN	CN	T	C	C	C	3/24/04	3/17/04	I	NY	MIN HUA ENTERPRISE, INC.
2004385781	CYPY	CYNOPS	PYRRHOGASTER	JAPANESE FIREBELLY	NEWT	LIV	630	NO	HK	HK	T	W	C	C	4/6/04	4/3/04	I	NY	ERDA INC.
2004385816	TRIH	TRITURUS	HONGKONGENSIS	ALPINE	NEWT	LIV	200	NO	HK	HK	T	C	C	C	3/31/04	3/22/04	I	NY	TRI-COUNTY TROPICALS, INC.
2004386235	CYPY	CYNOPS	PYRRHOGASTER	JAPANESE FIREBELLY	NEWT	LIV	600	NO	HK	HK	T	W	C	C	4/13/04	4/10/04	I	NY	ERDA INC.
2004386242	CYPY	CYNOPS	PYRRHOGASTER	JAPANESE FIREBELLY	NEWT	LIV	130	NO	HK	HK	T	W	C	C	4/13/04	4/10/04	I	NY	TRANSSHIP DISCOUNTS , LTD.
2004387355	CYOR	CYNOPS	ORIENTALIS	CHINESE DWARF	NEWT	LIV	3,800	NO	CN	CN	T	W	C	C	4/30/04	4/30/04	I	LA	Z IMPORTS INC.
2004387756	CYOR	CYNOPS	ORIENTALIS	CHINESE DWARF	NEWT	LIV	250	NO	HK	HK	T	C	C	C	5/1/04	5/1/04	I	LA	DOLPHIN INTERNATIONAL
2004387799	CYOR	CYNOPS	ORIENTALIS	CHINESE DWARF	NEWT	LIV	4,100	NO	HK	HK	T	C	C	C	5/1/04	5/2/04	I	LA	DOLPHIN INTERNATIONAL
2004387799	PACB	PACHYTRITON	BREVIPES	TSITOU	NEWT	LIV	100	NO	HK	HK	T	C	C	C	5/1/04	5/2/04	I	LA	DOLPHIN INTERNATIONAL
2004387811	TRIT	TRITURUS	SPECIES		NEWT	LIV	490	NO	HK	HK	T	C	C	C	5/2/04	5/2/04	I	LA	TROPICAL ENTERPRISES
2004387813	TRIT	TRITURUS	SPECIES		NEWT	LIV	1,400	NO	HK	HK	T	C	C	C	5/2/04	5/2/04	I	LA	GLOBAL AQUATIC CONSULTING
2004389172	CYOR	CYNOPS	ORIENTALIS	CHINESE DWARF	NEWT	LIV	4,650	NO	HK	HK	T	C	C	C	3/21/04	3/21/04	I	LA	DOLPHIN INTERNATIONAL
2004389288	CYOR	CYNOPS	ORIENTALIS	CHINESE DWARF	NEWT	LIV	600	NO	HK	HK	T	C	C	C	3/20/04	3/20/04	I	LA	DOLPHIN INTERNATIONAL
2004390247	SASA	SALAMANDRA	SALAMANDRA	EUROPEAN FIRE	SALAMANDER	LIV	200	NO	UA	UA	T	C	C	C	4/1/04	4/1/04	I	MI	STRICTLY REPTILES, INC.
2004391644	TRIT	TRITURUS	SPECIES		NEWT	LIV	1,600	NO	HK	HK	T	C	C	C	4/6/04	4/7/04	I	TP	5-D TROPICAL, INC.
2004391787	TRIT	TRITURUS	SPECIES		NEWT	LIV	1,600	NO	HK	HK	T	C	C	C	3/16/04	3/17/04	I	TP	5-D TROPICAL, INC.
2004392470	CYOR	CYNOPS	ORIENTALIS	CHINESE DWARF	NEWT	LIV	4,700	NO	HK	HK	T	C	C	C	5/9/04	5/9/04	I	LA	DOLPHIN INTERNATIONAL
2004392470	PACB	PACHYTRITON	BREVIPES	TSITOU	NEWT	LIV	200	NO	HK	HK	T	C	C	C	5/9/04	5/9/04	I	LA	DOLPHIN INTERNATIONAL
2004392493	TRIT	TRITURUS	SPECIES		NEWT	LIV	60	NO	HK	HK	T	C	C	C	5/9/04	5/9/04	I	LA	TROPICAL ENTERPRISES
2004392494	TRIT	TRITURUS	SPECIES		NEWT	LIV	2,300	NO	HK	HK	T	C	C	C	5/9/04	5/9/04	I	LA	GLOBAL AQUATIC CONSULTING
2004392511	TRIH	TRITURUS	HONGKONGENSIS	ALPINE	NEWT	LIV	200	NO	HK	HK	T	W	C	C	5/9/04	5/9/04	I	LA	AQUACO, INC.
2004392963	CYOR	CYNOPS	ORIENTALIS	CHINESE DWARF	NEWT	LIV	100	NO	SG	SG	T	W	C	C	4/19/04	2/22/04	I	NW	INTERNATIONAL PET RESOURCES LLC
2004392963	TYVE	TYLOTOTRITON	VERRUCOSUS	CROCODILE	NEWT	LIV	40	NO	SG	SG	T	W	C	C	4/19/04	2/22/04	I	NW	INTERNATIONAL PET RESOURCES LLC
2004393594	TYVE	TYLOTOTRITON	VERRUCOSUS	CROCODILE	NEWT	LIV	40	NO	SG	SG	T	W	C	C	4/16/04	3/7/04	I	NW	INTERNATIONAL PET RESOURCES LLC
2004395519	CYOR	CYNOPS	ORIENTALIS	CHINESE DWARF	NEWT	LIV	250	NO	HK	HK	T	C	C	C	5/15/04	5/15/04	I	LA	DOLPHIN INTERNATIONAL
2004395526	CYOR	CYNOPS	ORIENTALIS	CHINESE DWARF	NEWT	LIV	2,000	NO	CN	CN	T	W	C	C	5/15/04	5/15/04	I	LA	Z IMPORTS INC.
2004395601	TRIH	TRITURUS	HONGKONGENSIS	ALPINE	NEWT	LIV	300	NO	HK	HK	T	W	C	C	5/16/04	5/16/04	I	LA	AQUACO, INC.
2004395605	CYOR	CYNOPS	ORIENTALIS	CHINESE DWARF	NEWT	LIV	3,700	NO	HK	HK	T	C	C	C	5/16/04	5/16/04	I	LA	DOLPHIN INTERNATIONAL
2004395605	PACB	PACHYTRITON	BREVIPES	TSITOU	NEWT	LIV	200	NO	HK	HK	T	C	C	C	5/16/04	5/16/04	I	LA	DOLPHIN INTERNATIONAL
2004395606	TRIT	TRITURUS	SPECIES		NEWT	LIV	1,400	NO	HK	HK	T	C	C	C	5/16/04	5/16/04	I	LA	GLOBAL AQUATIC CONSULTING
2004395607	TRIT	TRITURUS	SPECIES		NEWT	LIV	30	NO	HK	HK	T	C	C	C	5/16/04	5/16/04	I	LA	TROPICAL ENTERPRISES
2004398970	TRIT	TRITURUS	SPECIES		NEWT	LIV	1,300	NO	HK	HK	T	C	C	C	5/22/04	5/22/04	I	LA	GLOBAL AQUATIC CONSULTING
2004398982	CYOR	CYNOPS	ORIENTALIS	CHINESE DWARF	NEWT	LIV	750	NO	HK	HK	T	C	C	C	5/22/04	5/22/04	I	LA	DOLPHIN INTERNATIONAL
2004398982	PACB	PACHYTRITON	BREVIPES	TSITOU	NEWT	LIV	100	NO	HK	HK	T	C	C	C	5/22/04	5/22/04	I	LA	DOLPHIN INTERNATIONAL
2004399040	CYOR	CYNOPS	ORIENTALIS	CHINESE DWARF	NEWT	LIV	4,750	NO	HK	HK	T	C	C	C	5/23/04	5/23/04	I	LA	DOLPHIN INTERNATIONAL
2004399040	PACB	PACHYTRITON	BREVIPES	TSITOU	NEWT	LIV	200	NO	HK	HK	T	C	C	C	5/23/04	5/23/04	I	LA	DOLPHIN INTERNATIONAL
2004399052	TRIH	TRITURUS	HONGKONGENSIS	ALPINE	NEWT	LIV	200	NO	HK	HK	T	W	C	C	5/23/04	5/23/04	I	LA	AQUACO, INC.
2004399082	TRIT	TRITURUS	SPECIES		NEWT	LIV	250	NO	HK	HK	T	C	C	C	5/23/04	5/23/04	I	LA	TROPICAL ENTERPRISES
2004399111	TRIH	TRITURUS	HONGKONGENSIS	ALPINE	NEWT	LIV	400	NO	HK	HK	T	C	C	C	4/25/04	4/25/04	I	LA	CIS INTERNATIONAL HOLDINGS CORP.
2004401391	CYPY	CYNOPS	PYRRHOGASTER	JAPANESE FIREBELLY	NEWT	LIV	510	NO	HK	HK	T	W	C	C	4/21/04	4/17/04	I	NY	ERDA INC.
2004401468	CYPY	CYNOPS	PYRRHOGASTER	JAPANESE FIREBELLY	NEWT	LIV	150	NO	HK	HK	T	W	C	C	4/24/04	4/24/04	I	NY	TRANSSHIP DISCOUNTS , LTD.
2004401500	CYPY	CYNOPS	PYRRHOGASTER	JAPANESE FIREBELLY	NEWT	LIV	480	NO	HK	HK	T	W	C	C	4/27/04	4/24/04	I	NY	ERDA INC.

2004401695	CYOR	CYNOPS	ORIENTALIS	CHINESE DWARF	NEWT	LIV	200	NO	CN	CN	T	F	C	C	4/26/04	4/21/04	I	NY	MIN HUA ENTERPRISE, INC.
2004401940	TRIH	TRITURUS	HONGKONGENSIS	ALPINE	NEWT	LIV	250	NO	HK	HK	T	C	C	C	4/19/04	4/12/04	I	NY	TRI-COUNTY TROPICALS, INC.
2004402011	CYOR	CYNOPS	ORIENTALIS	CHINESE DWARF	NEWT	LIV	250	NO	HK	HK	T	C	C	C	5/29/04	5/29/04	I	LA	DOLPHIN INTERNATIONAL
2004402018	TRIT	TRITURUS	SPECIES		NEWT	LIV	700	NO	HK	HK	T	C	C	C	5/29/04	5/29/04	I	LA	GLOBAL AQUATIC CONSULTING
2004402027	CYOR	CYNOPS	ORIENTALIS	CHINESE DWARF	NEWT	LIV	3,000	NO	CN	CN	T	C	C	C	5/29/04	5/29/04	I	LA	Z IMPORTS INC.
2004402068	CYOR	CYNOPS	ORIENTALIS	CHINESE DWARF	NEWT	LIV	4,300	NO	HK	HK	T	C	C	C	5/30/04	5/30/04	I	LA	DOLPHIN INTERNATIONAL
2004402068	PACB	PACHYTRITON	BREVIPES	TSITOU	NEWT	LIV	100	NO	HK	HK	T	C	C	C	5/30/04	5/30/04	I	LA	DOLPHIN INTERNATIONAL
2004402091	TRIH	TRITURUS	HONGKONGENSIS	ALPINE	NEWT	LIV	50	NO	HK	HK	T	W	C	C	5/30/04	5/30/04	I	LA	AQUACO, INC.
2004402517	CYPY	CYNOPS	PYRRHOGASTER	JAPANESE FIREBELLY	NEWT	LIV	640	NO	HK	HK	T	W	C	C	5/18/04	5/15/04	I	NY	ERDA INC.
2004403157	CYPY	CYNOPS	PYRRHOGASTER	JAPANESE FIREBELLY	NEWT	LIV	460	NO	HK	HK	T	W	C	C	3/31/04	3/27/04	I	NY	TRANSSHIP DISCOUNTS , LTD.
2004403186	CYPY	CYNOPS	PYRRHOGASTER	JAPANESE FIREBELLY	NEWT	LIV	125	NO	HK	HK	T	W	C	C	3/31/04	3/27/04	I	NY	ERDA INC.
2004403377	CYOR	CYNOPS	ORIENTALIS	CHINESE DWARF	NEWT	LIV	200	NO	CN	CN	T	F	C	C	4/9/04	3/31/04	I	NY	MIN HUA ENTERPRISE, INC.
2004403431	CYPY	CYNOPS	PYRRHOGASTER	JAPANESE FIREBELLY	NEWT	LIV	30	NO	HK	HK	T	W	C	C	4/21/04	4/17/04	I	NY	TRANSSHIP DISCOUNTS , LTD.
2004403613	CNP?	CYNOPS	SPECIES	FIREBELLY	NEWT	LIV	400	NO	CN	CN	T	W	C	C	5/5/04	5/2/04	I	NY	FISH MART INC.
2004403655	CYPY	CYNOPS	PYRRHOGASTER	JAPANESE FIREBELLY	NEWT	LIV	1,100	NO	HK	HK	T	W	C	C	5/4/04	5/1/04	I	NY	TRANSSHIP DISCOUNTS , LTD.
2004404021	CYPY	CYNOPS	PYRRHOGASTER	JAPANESE FIREBELLY	NEWT	LIV	290	NO	HK	HK	T	W	C	C	5/10/04	5/8/04	I	NY	TRANSSHIP DISCOUNTS , LTD.
2004404057	CYPY	CYNOPS	PYRRHOGASTER	JAPANESE FIREBELLY	NEWT	LIV	480	NO	HK	HK	T	W	C	C	5/4/04	5/1/04	I	NY	ERDA INC.
2004404073	TRIH	TRITURUS	HONGKONGENSIS	ALPINE	NEWT	LIV	400	NO	HK	HK	T	W	C	C	5/10/04	4/25/04	I	NY	INTERNATIONAL PET RESOURCES LLC
2004405109	TRIT	TRITURUS	SPECIES		NEWT	LIV	500	NO	HK	HK	T	C	C	C	6/5/04	6/5/04	I	LA	GLOBAL AQUATIC CONSULTING
2004405204	CYOR	CYNOPS	ORIENTALIS	CHINESE DWARF	NEWT	LIV	8,700	NO	HK	HK	T	C	C	C	6/6/04	6/6/04	I	LA	DOLPHIN INTERNATIONAL
2004405204	TYVE	TYLOTOTRITON	VERRUCOSUS	CROCODILE	NEWT	LIV	41	NO	HK	HK	T	C	C	C	6/6/04	6/6/04	I	LA	DOLPHIN INTERNATIONAL
2004405204	PACB	PACHYTRITON	BREVIPES	TSITOU	NEWT	LIV	200	NO	HK	HK	T	C	C	C	6/6/04	6/6/04	I	LA	DOLPHIN INTERNATIONAL
2004405220	TRIH	TRITURUS	HONGKONGENSIS	ALPINE	NEWT	LIV	275	NO	HK	HK	T	W	C	C	6/6/04	6/6/04	I	LA	AQUACO, INC.
2004405430	TRIT	TRITURUS	SPECIES		NEWT	LIV	25	NO	SG	SG	T	W	C	C	4/22/04	4/22/04	I	CH	INTERCONTINENTAL IMPORTS, INC.
2004406109	CNP?	CYNOPS	SPECIES	FIREBELLY	NEWT	LIV	95	NO	HK	HK	T	W	C	C	6/9/04	6/9/04	I	LA	U.S. GLOBAL EXOTICS, INC.
2004406109	TYL?	TYLOTOTRITON	SPECIES	CROCODILE	NEWT	LIV	879	NO	HK	HK	T	W	C	C	6/9/04	6/9/04	I	LA	U.S. GLOBAL EXOTICS, INC.
2004406117	SASA	SALAMANDRA	SALAMANDRA	EUROPEAN FIRE	SALAMANDER	LIV	150	NO	RU	RU	T	C	C	C	6/9/04	6/9/04	I	LA	IZI INTERNATIONAL ZOOLOGICAL IMPORTS
2004406128	CNP?	CYNOPS	SPECIES	FIREBELLY	NEWT	LIV	95	NO	HK	HK	T	W	C	C	6/9/04	6/9/04	I	LA	U.S. GLOBAL EXOTICS, INC.
2004406128	TYL?	TYLOTOTRITON	SPECIES	CROCODILE	NEWT	LIV	879	NO	HK	HK	T	W	C	C	6/9/04	6/9/04	I	LA	U.S. GLOBAL EXOTICS, INC.
2004406781	TRIH	TRITURUS	HONGKONGENSIS	ALPINE	NEWT	LIV	25	NO	HK	HK	T	W	C	C	6/13/04	6/13/04	I	LA	AQUACO, INC.
2004408301	TRIT	TRITURUS	SPECIES		NEWT	LIV	500	NO	HK	HK	T	W	C	C	6/13/04	6/13/04	I	LA	C.I.S. INTERNATIONAL
2004408307	TRIH	TRITURUS	HONGKONGENSIS	ALPINE	NEWT	LIV	1,200	NO	HK	HK	T	C	C	C	6/12/04	6/12/04	I	LA	GLOBAL AQUATIC CONSULTING
2004409521	CYPY	CYNOPS	PYRRHOGASTER	JAPANESE FIREBELLY	NEWT	LIV	470	NO	HK	HK	T	W	C	C	5/25/04	5/22/04	I	NY	ERDA INC.
2004409695	CYOR	CYNOPS	ORIENTALIS	CHINESE DWARF	NEWT	LIV	2,000	NO	CN	CN	T	C	C	C	6/19/04	6/19/04	I	LA	Z IMPORTS INC.
2004409746	TRIH	TRITURUS	HONGKONGENSIS	ALPINE	NEWT	LIV	750	NO	HK	HK	T	W	C	C	6/19/04	6/20/04	I	LA	AQUACO, INC.
2004409774	CYOR	CYNOPS	ORIENTALIS	CHINESE DWARF	NEWT	LIV	5,250	NO	HK	HK	T	C	C	C	6/19/04	6/20/04	I	LA	DOLPHIN INTERNATIONAL
2004409774	TYVE	TYLOTOTRITON	VERRUCOSUS	CROCODILE	NEWT	LIV	25	NO	HK	HK	T	C	C	C	6/19/04	6/20/04	I	LA	DOLPHIN INTERNATIONAL
2004409774	PACB	PACHYTRITON	BREVIPES	TSITOU	NEWT	LIV	100	NO	HK	HK	T	C	C	C	6/19/04	6/20/04	I	LA	DOLPHIN INTERNATIONAL
2004409885	CYPY	CYNOPS	PYRRHOGASTER	JAPANESE FIREBELLY	NEWT	LIV	100	NO	HK	HK	T	W	C	C	5/25/04	5/22/04	I	NY	TRANSSHIP DISCOUNTS , LTD.
2004409885	PACB	PACHYTRITON	BREVIPES	TSITOU	NEWT	LIV	60	NO	HK	HK	T	W	C	C	5/25/04	5/22/04	I	NY	TRANSSHIP DISCOUNTS , LTD.
2004410281	CYPY	CYNOPS	PYRRHOGASTER	JAPANESE FIREBELLY	NEWT	LIV	608	NO	HK	HK	T	W	C	C	5/18/04	5/15/04	I	NY	TRANSSHIP DISCOUNTS , LTD.
2004410313	CYPY	CYNOPS	PYRRHOGASTER	JAPANESE FIREBELLY	NEWT	LIV	240	NO	HK	HK	T	W	C	C	5/10/04	5/8/04	I	NY	ERDA INC.
2004410318	CYPY	CYNOPS	PYRRHOGASTER	JAPANESE FIREBELLY	NEWT	LIV	400	NO	CN	CN	T	W	C	C	5/12/04	5/9/04	I	NY	FISH MART INC.
2004410362	TRIT	TRITURUS	SPECIES		NEWT	LIV	250	NO	HK	HK	T	C	C	C	5/18/04	5/17/04	I	NY	TRI-COUNTY TROPICALS, INC.
2004410775	CYOR	CYNOPS	ORIENTALIS	CHINESE DWARF	NEWT	LIV	300	NO	CN	CN	T	F	C	C	4/26/04	4/14/04	I	NY	MIN HUA ENTERPRISE, INC.
2004410937	CYPY	CYNOPS	PYRRHOGASTER	JAPANESE FIREBELLY	NEWT	LIV	150	NO	HK	HK	T	W	C	C	6/4/04	5/29/04	I	NY	TRANSSHIP DISCOUNTS , LTD.
2004410937	PACB	PACHYTRITON	BREVIPES	TSITOU	NEWT	LIV	120	NO	HK	HK	T	W	C	C	6/4/04	5/29/04	I	NY	TRANSSHIP DISCOUNTS , LTD.
2004411307	TRIT	TRITURUS	SPECIES		NEWT	LIV	400	NO	HK	HK	T	W	C	C	5/18/04	5/9/04	I	NY	ALL AMERICAN TRANSHIP
2004413414	CYOR	CYNOPS	ORIENTALIS	CHINESE DWARF	NEWT	LIV	3,500	NO	CN	CN	T	C	C	C	6/26/04	6/26/04	I	LA	Z IMPORTS INC.
2004413415	TRIT	TRITURUS	SPECIES		NEWT	LIV	80	NO	HK	HK	T	C	C	C	6/26/04	6/26/04	I	LA	GLOBAL AQUATIC CONSULTING
2004413430	CYOR	CYNOPS	ORIENTALIS	CHINESE DWARF	NEWT	LIV	250	NO	HK	HK	T	C	C	C	6/26/04	6/26/04	I	LA	DOLPHIN INTERNATIONAL
2004413483	CYOR	CYNOPS	ORIENTALIS	CHINESE DWARF	NEWT	LIV	5,900	NO	HK	HK	T	C	C	C	6/26/04	6/27/04	I	LA	DOLPHIN INTERNATIONAL
2004413511	TRIH	TRITURUS	HONGKONGENSIS	ALPINE	NEWT	LIV	536	NO	HK	HK	T	W	C	C	6/27/04	6/27/04	I	LA	AQUACO, INC.
2004414432	BODO	BOLITOGLOSSA	DOFLEINI	ALTA VERAPAZ	SALAMANDER	LIV	13	NO	GT	GT	T	W	C	C	6/3/04	6/3/04	I	MI	DHAR TROPICAL LLC
2004416569	CYOR	CYNOPS	ORIENTALIS	CHINESE DWARF	NEWT	LIV	250	NO	HK	HK	T	C	C	C	7/3/04	7/3/04	I	LA	DOLPHIN INTERNATIONAL
2004416575	TYVE	TYLOTOTRITON	VERRUCOSUS	CROCODILE	NEWT	LIV	800	NO	CN	CN	T	C	C	C	7/3/04	7/3/04	I	LA	Z IMPORTS INC.
2004416577	TRIT	TRITURUS	SPECIES		NEWT	LIV	200	NO	HK	HK	T	C	C	C	7/3/04	7/3/04	I	LA	GLOBAL AQUATIC CONSULTING
2004416625	CYOR	CYNOPS	ORIENTALIS	CHINESE DWARF	NEWT	LIV	5,100	NO	HK	HK	T	C	C	C	7/3/04	7/4/04	I	LA	DOLPHIN INTERNATIONAL
2004416625	PACB	PACHYTRITON	BREVIPES	TSITOU	NEWT	LIV	100	NO	HK	HK	T	C	C	C	7/3/04	7/4/04	I	LA	DOLPHIN INTERNATIONAL

2004416639	TRIH	TRITURUS	HONGKONGENSIS	ALPINE	NEWT	LIV	50	NO	HK	HK	T	W	C	C	7/4/04	7/4/04	I	LA	AQUACO, INC.
2004417306	TRIT	TRITURUS	SPECIES		NEWT	LIV	1,600	NO	HK	HK	T	C	C	C	6/24/04	6/9/04	I	TP	5-D TROPICAL, INC.
2004417449	TRIT	TRITURUS	SPECIES		NEWT	LIV	1,600	NO	HK	HK	T	C	C	C	6/3/04	5/26/04	I	TP	5-D TROPICAL, INC.
2004417482	TRIT	TRITURUS	SPECIES		NEWT	LIV	1,600	NO	HK	HK	T	C	C	C	6/24/04	6/23/04	I	TP	5-D TROPICAL, INC.
2004417546	TRIT	TRITURUS	SPECIES		NEWT	LIV	1,600	NO	HK	HK	T	C	C	C	4/26/04	4/28/04	I	TP	5-D TROPICAL, INC.
2004417588	TRIT	TRITURUS	SPECIES		NEWT	LIV	1,600	NO	HK	HK	T	C	C	C	5/21/04	5/19/04	I	TP	5-D TROPICAL, INC.
2004417605	TRIT	TRITURUS	SPECIES		NEWT	LIV	1,600	NO	**	HK	T	C	C	C	3/24/04	3/24/04	I	TP	5-D TROPICAL, INC.
2004417608	TRIT	TRITURUS	SPECIES		NEWT	LIV	1,600	NO	HK	HK	T	C	C	C	4/12/04	4/14/04	I	TP	5-D TROPICAL, INC.
2004417622	TRIT	TRITURUS	SPECIES		NEWT	LIV	1,600	NO	HK	HK	T	C	C	C	1/26/04	1/28/04	I	TP	5-D TROPICAL, INC.
2004418645	CYOR	CYNOPS	ORIENTALIS	CHINESE DWARF	NEWT	LIV	700	NO	CN	CN	T	C	C	C	7/10/04	7/10/04	I	LA	Z IMPORTS INC.
2004418704	CYOR	CYNOPS	ORIENTALIS	CHINESE DWARF	NEWT	LIV	5,550	NO	HK	HK	T	C	C	C	7/10/04	7/11/04	I	LA	DOLPHIN INTERNATIONAL
2004418704	PACB	PACHYTRITON	BREVIPES	TSITOU	NEWT	LIV	100	NO	HK	HK	T	C	C	C	7/10/04	7/11/04	I	LA	DOLPHIN INTERNATIONAL
2004418717	TRIT	TRITURUS	SPECIES		NEWT	LIV	155	NO	HK	HK	T	C	C	C	7/11/04	7/11/04	I	LA	TROPICAL ENTERPRISES
2004418735	TRIH	TRITURUS	HONGKONGENSIS	ALPINE	NEWT	LIV	450	NO	HK	HK	T	W	C	C	7/11/04	7/11/04	I	LA	AQUACO, INC.
2004419387	TYL?	TYLOTOTRITON	SPECIES	CROCODILE	NEWT	LIV	25	NO	HK	HK	T	W	C	C	7/13/04	7/13/04	I	LA	STRICTLY REPTILES, INC.
2004419387	TYL?	TYLOTOTRITON	SPECIES	CROCODILE	NEWT	LIV	25	NO	HK	HK	T	W	C	C	7/13/04	7/13/04	I	LA	STRICTLY REPTILES, INC.
2004419387	PCHI	PARAMESOTRITON	CHINENSIS	CHINESE WARTY	NEWT	LIV	100	NO	HK	HK	T	W	C	C	7/13/04	7/13/04	I	LA	STRICTLY REPTILES, INC.
2004419588	SASA	SALAMANDRA	SALAMANDRA	EUROPEAN FIRE	SALAMANDER	LIV	130	NO	RU	RU	T	W	C	C	7/14/04	7/14/04	I	LA	CALIFORNIA ZOOLOGICAL SUPPLY
2004421023	CYOR	CYNOPS	ORIENTALIS	CHINESE DWARF	NEWT	LIV	3,500	NO	CN	CN	T	W	C	C	7/17/04	7/17/04	I	LA	Z IMPORTS INC.
2004421026	TRIT	TRITURUS	SPECIES		NEWT	LIV	200	NO	HK	HK	T	C	C	C	7/17/04	7/17/04	I	LA	GLOBAL AQUATIC CONSULTING
2004421098	CYOR	CYNOPS	ORIENTALIS	CHINESE DWARF	NEWT	LIV	3,350	NO	HK	HK	T	C	C	C	7/17/04	7/18/04	I	LA	DOLPHIN INTERNATIONAL
2004421105	TRIH	TRITURUS	HONGKONGENSIS	ALPINE	NEWT	LIV	600	NO	HK	HK	T	W	C	C	7/18/04	7/18/04	I	LA	AQUACO, INC.
2004421123	TRIT	TRITURUS	SPECIES		NEWT	LIV	1,000	NO	HK	HK	T	C	C	C	7/18/04	7/18/04	I	LA	AQUATIC CONNECTION
2004421133	TRIT	TRITURUS	SPECIES		NEWT	LIV	400	NO	CN	HK	T	C	C	C	7/18/04	7/18/04	I	LA	C.I.S. INTERNATIONAL
2004421567	CYOR	CYNOPS	ORIENTALIS	CHINESE DWARF	NEWT	LIV	250	NO	HK	HK	T	C	C	C	6/12/04	6/12/04	I	LA	DOLPHIN INTERNATIONAL
2004421601	CYOR	CYNOPS	ORIENTALIS	CHINESE DWARF	NEWT	LIV	3,600	NO	HK	HK	T	C	C	C	6/13/04	6/13/04	I	LA	DOLPHIN INTERNATIONAL
2004421601	PACB	PACHYTRITON	BREVIPES	TSITOU	NEWT	LIV	300	NO	HK	HK	T	C	C	C	6/13/04	6/13/04	I	LA	DOLPHIN INTERNATIONAL
2004423858	CYOR	CYNOPS	ORIENTALIS	CHINESE DWARF	NEWT	LIV	5,700	NO	HK	HK	T	C	C	C	7/24/04	7/25/04	I	LA	DOLPHIN INTERNATIONAL
2004423858	PACB	PACHYTRITON	BREVIPES	TSITOU	NEWT	LIV	100	NO	HK	HK	T	C	C	C	7/24/04	7/25/04	I	LA	DOLPHIN INTERNATIONAL
2004423871	TRIH	TRITURUS	HONGKONGENSIS	ALPINE	NEWT	LIV	300	NO	HK	HK	T	W	C	C	7/25/04	7/25/04	I	LA	AQUACO, INC.
2004427564	PACB	PACHYTRITON	BREVIPES	TSITOU	NEWT	LIV	300	NO	HK	HK	T	C	C	C	7/31/04	8/1/04	I	LA	DOLPHIN INTERNATIONAL
2004427567	TRIH	TRITURUS	HONGKONGENSIS	ALPINE	NEWT	LIV	700	NO	HK	HK	T	W	C	C	7/31/04	8/1/04	I	LA	AQUACO, INC.
2004427568	TRIT	TRITURUS	SPECIES		NEWT	LIV	340	NO	HK	HK	T	C	C	C	7/31/04	8/1/04	I	LA	TROPICAL ENTERPRISES
2004430769	PACB	PACHYTRITON	BREVIPES	TSITOU	NEWT	LIV	400	NO	HK	HK	T	C	C	C	8/7/04	8/8/04	I	LA	DOLPHIN INTERNATIONAL
2004430769	CYOR	CYNOPS	ORIENTALIS	CHINESE DWARF	NEWT	LIV	4,250	NO	HK	HK	T	C	C	C	8/7/04	8/8/04	I	LA	DOLPHIN INTERNATIONAL
2004430771	TRIH	TRITURUS	HONGKONGENSIS	ALPINE	NEWT	LIV	300	NO	HK	HK	T	W	C	C	8/7/04	8/8/04	I	LA	AQUACO, INC.
2004430773	TRIT	TRITURUS	SPECIES		NEWT	LIV	250	NO	HK	HK	T	C	C	C	8/7/04	8/8/04	I	LA	TROPICAL ENTERPRISES
2004431360	CYOR	CYNOPS	ORIENTALIS	CHINESE DWARF	NEWT	LIV	1,800	NO	CN	CN	T	W	C	C	8/9/04	8/7/04	I	LA	EVOLUTION ENTERPRISE
2004431360	PLAB	PACHYTRITON	LABIATUS	UNTERSTEIN'S	NEWT	LIV	200	NO	CN	CN	T	W	C	C	8/9/04	8/7/04	I	LA	EVOLUTION ENTERPRISE
2004433889	CYPY	CYNOPS	PYRRHOGASTER	JAPANESE FIREBELLY	NEWT	LIV	600	NO	HK	HK	T	W	C	C	1/20/04	1/17/04	I	NY	ERDA INC.
2004434220	PLAB	PACHYTRITON	LABIATUS	UNTERSTEIN'S	NEWT	LIV	100	NO	CN	CN	T	C	C	C	8/14/04	8/14/04	I	LA	EVOLUTION ENTERPRISE
2004434220	CYOR	CYNOPS	ORIENTALIS	CHINESE DWARF	NEWT	LIV	150	NO	CN	CN	T	C	C	C	8/14/04	8/14/04	I	LA	EVOLUTION ENTERPRISE
2004434271	PACB	PACHYTRITON	BREVIPES	TSITOU	NEWT	LIV	200	NO	HK	HK	T	C	C	C	8/15/04	8/15/04	I	LA	DOLPHIN INTERNATIONAL
2004434271	CYOR	CYNOPS	ORIENTALIS	CHINESE DWARF	NEWT	LIV	6,700	NO	HK	HK	T	C	C	C	8/15/04	8/15/04	I	LA	DOLPHIN INTERNATIONAL
2004434273	TRIT	TRITURUS	SPECIES		NEWT	LIV	280	NO	VS	HK	T	C	C	C	8/15/04	8/15/04	I	LA	TROPICAL ENTERPRISES
2004434274	TRIH	TRITURUS	HONGKONGENSIS	ALPINE	NEWT	LIV	500	NO	HK	HK	T	W	C	C	8/15/04	8/15/04	I	LA	AQUACO, INC.
2004435003	CYPY	CYNOPS	PYRRHOGASTER	JAPANESE FIREBELLY	NEWT	LIV	530	NO	HK	HK	T	W	C	C	6/7/04	5/22/04	I	NY	ERDA INC.
2004435022	CYPY	CYNOPS	PYRRHOGASTER	JAPANESE FIREBELLY	NEWT	LIV	200	NO	HK	HK	T	W	C	C	6/7/04	6/5/04	I	NY	TRANSSHIP DISCOUNTS , LTD.
2004435297	CYPY	CYNOPS	PYRRHOGASTER	JAPANESE FIREBELLY	NEWT	LIV	600	NO	HK	HK	T	W	C	C	7/19/04	7/17/04	I	NY	ERDA INC.
2004435477	TRIH	TRITURUS	HONGKONGENSIS	ALPINE	NEWT	LIV	280	NO	HK	HK	T	C	C	C	7/21/04	7/12/04	I	NY	TRI-COUNTY TROPICALS, INC.
2004436012	CYPY	CYNOPS	PYRRHOGASTER	JAPANESE FIREBELLY	NEWT	LIV	50	NO	HK	HK	T	W	C	C	7/12/04	7/11/04	I	NY	TRANSSHIP DISCOUNTS , LTD.
2004436012	PACB	PACHYTRITON	BREVIPES	TSITOU	NEWT	LIV	60	NO	HK	HK	T	W	C	C	7/12/04	7/11/04	I	NY	TRANSSHIP DISCOUNTS , LTD.
2004438074	CYOR	CYNOPS	ORIENTALIS	CHINESE DWARF	NEWT	LIV	2,100	NO	CN	CN	T	W	C	C	8/21/04	8/21/04	I	LA	EVOLUTION ENTERPRISE
2004438074	PLAB	PACHYTRITON	LABIATUS	UNTERSTEIN'S	NEWT	LIV	200	NO	CN	CN	T	W	C	C	8/21/04	8/21/04	I	LA	EVOLUTION ENTERPRISE
2004438092	PACB	PACHYTRITON	BREVIPES	TSITOU	NEWT	LIV	100	NO	HK	HK	T	C	C	C	8/21/04	8/21/04	I	LA	DOLPHIN INTERNATIONAL
2004438092	CYOR	CYNOPS	ORIENTALIS	CHINESE DWARF	NEWT	LIV	500	NO	HK	HK	T	C	C	C	8/21/04	8/21/04	I	LA	DOLPHIN INTERNATIONAL
2004438138	TRIT	TRITURUS	SPECIES		NEWT	LIV	30	NO	VS	HK	T	C	C	C	8/22/04	8/22/04	I	LA	TROPICAL ENTERPRISES
2004438172	PACB	PACHYTRITON	BREVIPES	TSITOU	NEWT	LIV	200	NO	HK	HK	T	C	C	C	8/22/04	8/22/04	I	LA	DOLPHIN INTERNATIONAL
2004438172	CYOR	CYNOPS	ORIENTALIS	CHINESE DWARF	NEWT	LIV	6,100	NO	HK	HK	T	C	C	C	8/22/04	8/22/04	I	LA	DOLPHIN INTERNATIONAL
2004438173	TRIH	TRITURUS	HONGKONGENSIS	ALPINE	NEWT	LIV	400	NO	HK	HK	T	W	C	C	8/22/04	8/22/04	I	LA	AQUACO, INC.

2004441164	CYOR	CYNOPS	ORIENTALIS	CHINESE DWARF	NEWT	LIV	2,000	NO	CN	CN	T	W	C	C	8/28/04	8/28/04	I	LA	Z IMPORTS INC.
2004441187	CYOR	CYNOPS	ORIENTALIS	CHINESE DWARF	NEWT	LIV	1,500	NO	CN	CN	T	W	C	C	8/28/04	8/28/04	I	LA	EVOLUTION ENTERPRISE
2004441187	PLAB	PACHYTRITON	LABIATUS	UNTERSTEIN'S	NEWT	LIV	100	NO	CN	CN	T	W	C	C	8/28/04	8/28/04	I	LA	EVOLUTION ENTERPRISE
2004441216	CYOR	CYNOPS	ORIENTALIS	CHINESE DWARF	NEWT	LIV	1,500	NO	HK	HK	T	C	C	C	8/28/04	8/29/04	I	LA	DOLPHIN INTERNATIONAL
2004441239	TRIH	TRITURUS	HONGKONGENSIS	ALPINE	NEWT	LIV	1,000	NO	HK	HK	T	W	C	C	8/29/04	8/29/04	I	LA	AQUACO, INC.
2004441345	PACB	PACHYTRITON	BREVIPES	TSITOU	NEWT	LIV	100	NO	HK	HK	T	C	C	C	8/30/04	8/30/04	I	LA	DOLPHIN INTERNATIONAL
2004442369	CYPY	CYNOPS	PYRRHOGASTER	JAPANESE FIREBELLY	NEWT	LIV	150	NO	HK	HK	T	W	C	C	8/17/04	8/14/04	I	NY	TRANSSHIP DISCOUNTS , LTD.
2004442369	PACB	PACHYTRITON	BREVIPES	TSITOU	NEWT	LIV	180	NO	HK	HK	T	W	C	C	8/17/04	8/14/04	I	NY	TRANSSHIP DISCOUNTS , LTD.
2004442997	TRIH	TRITURUS	HONGKONGENSIS	ALPINE	NEWT	LIV	300	NO	HK	HK	T	W	C	C	7/28/04	7/18/04	I	NY	INTERNATIONAL PET RESOURCES LLC
2004443553	CNP?	CYNOPS	SPECIES	FIREBELLY	NEWT	LIV	520	NO	HK	HK	T	W	C	C	7/12/04	7/10/04	I	NY	ERDA INC.
2004443572	CYPY	CYNOPS	PYRRHOGASTER	JAPANESE FIREBELLY	NEWT	LIV	400	NO	CN	CN	T	W	C	C	6/22/04	6/13/04	I	NY	FISH MART INC.
2004443587	CYPY	CYNOPS	PYRRHOGASTER	JAPANESE FIREBELLY	NEWT	LIV	400	NO	CN	CN	T	W	C	C	7/20/04	7/18/04	I	NY	FISH MART INC.
2004443695	CYPY	CYNOPS	PYRRHOGASTER	JAPANESE FIREBELLY	NEWT	LIV	150	NO	HK	HK	T	W	C	C	6/29/04	6/26/04	I	NY	TRANSSHIP DISCOUNTS , LTD.
2004443695	PACB	PACHYTRITON	BREVIPES	TSITOU	NEWT	LIV	60	NO	HK	HK	T	W	C	C	6/29/04	6/26/04	I	NY	TRANSSHIP DISCOUNTS , LTD.
2004443849	CNP?	CYNOPS	SPECIES	FIREBELLY	NEWT	LIV	7	NO	HK	HK	T	W	C	C	9/2/04	9/2/04	I	LA	U.S. GLOBAL EXOTICS, INC.
2004443849	TYL?	TYLOTOTRITON	SPECIES	CROCODILE	NEWT	LIV	200	NO	HK	HK	T	W	C	C	9/2/04	9/2/04	I	LA	U.S. GLOBAL EXOTICS, INC.
2004444061	CYPY	CYNOPS	PYRRHOGASTER	JAPANESE FIREBELLY	NEWT	LIV	375	NO	HK	HK	T	W	C	C	6/22/04	6/12/04	I	NY	ERDA INC.
2004444348	CYPY	CYNOPS	PYRRHOGASTER	JAPANESE FIREBELLY	NEWT	LIV	320	NO	HK	HK	T	W	C	C	6/20/04	6/12/04	I	NY	TRANSSHIP DISCOUNTS , LTD.
2004444377	TRIH	TRITURUS	HONGKONGENSIS	ALPINE	NEWT	LIV	200	NO	HK	HK	T	C	C	C	6/20/04	6/14/04	I	NY	TRI-COUNTY TROPICALS, INC.
2004444631	CYOR	CYNOPS	ORIENTALIS	CHINESE DWARF	NEWT	LIV	1,800	NO	CN	CN	T	W	C	C	9/4/04	9/4/04	I	LA	EVOLUTION ENTERPRISE
2004444639	CYOR	CYNOPS	ORIENTALIS	CHINESE DWARF	NEWT	LIV	2,000	NO	CN	CN	T	W	C	C	9/4/04	9/4/04	I	LA	Z IMPORTS INC.
2004444646	CYOR	CYNOPS	ORIENTALIS	CHINESE DWARF	NEWT	LIV	1,000	NO	HK	HK	T	C	C	C	9/4/04	9/4/04	I	LA	DOLPHIN INTERNATIONAL
2004444695	PACB	PACHYTRITON	BREVIPES	TSITOU	NEWT	LIV	400	NO	HK	HK	T	C	C	C	9/4/04	9/5/04	I	LA	DOLPHIN INTERNATIONAL
2004444695	CYOR	CYNOPS	ORIENTALIS	CHINESE DWARF	NEWT	LIV	5,600	NO	HK	HK	T	C	C	C	9/4/04	9/5/04	I	LA	DOLPHIN INTERNATIONAL
2004447511	CYOR	CYNOPS	ORIENTALIS	CHINESE DWARF	NEWT	LIV	1,500	NO	CN	CN	T	W	C	C	9/11/04	9/11/04	I	LA	Z IMPORTS INC.
2004447514	PLAB	PACHYTRITON	LABIATUS	UNTERSTEIN'S	NEWT	LIV	100	NO	CN	CN	T	C	C	C	9/11/04	9/11/04	I	LA	EVOLUTION ENTERPRISE
2004447514	CYOR	CYNOPS	ORIENTALIS	CHINESE DWARF	NEWT	LIV	300	NO	CN	CN	T	C	C	C	9/11/04	9/11/04	I	LA	EVOLUTION ENTERPRISE
2004447522	CYOR	CYNOPS	ORIENTALIS	CHINESE DWARF	NEWT	LIV	1,000	NO	HK	HK	T	C	C	C	9/11/04	9/11/04	I	LA	DOLPHIN INTERNATIONAL
2004447598	PACB	PACHYTRITON	BREVIPES	TSITOU	NEWT	LIV	100	NO	HK	HK	T	C	C	C	9/12/04	9/12/04	I	LA	DOLPHIN INTERNATIONAL
2004447598	CYOR	CYNOPS	ORIENTALIS	CHINESE DWARF	NEWT	LIV	4,200	NO	HK	HK	T	C	C	C	9/12/04	9/12/04	I	LA	DOLPHIN INTERNATIONAL
2004447602	TRIH	TRITURUS	HONGKONGENSIS	ALPINE	NEWT	LIV	850	NO	HK	HK	T	W	C	C	9/12/04	9/12/04	I	LA	AQUACO, INC.
2004449388	BATS	BATRACHOSEPS	STEBBINSI	TEHACHAPI SLENDER	SALAMANDER	LIV	5	NO	CA	CA	T	A	C	C	9/9/04	9/9/04	I	NY	PINELLAS COUNTY SCHOOLS
2004450339	CYOR	CYNOPS	ORIENTALIS	CHINESE DWARF	NEWT	LIV	1,000	NO	HK	HK	T	C	C	C	9/18/04	9/18/04	I	LA	DOLPHIN INTERNATIONAL
2004450409	TRIH	TRITURUS	HONGKONGENSIS	ALPINE	NEWT	LIV	600	NO	HK	HK	T	W	C	C	9/19/04	9/19/04	I	LA	AQUACO, INC.
2004450419	TRIT	TRITURUS	SPECIES		NEWT	LIV	250	NO	HK	HK	T	C	C	C	9/19/04	9/19/04	I	LA	TROPICAL ENTERPRISES
2004450441	PACB	PACHYTRITON	BREVIPES	TSITOU	NEWT	LIV	300	NO	HK	HK	T	C	C	C	9/19/04	9/19/04	I	LA	DOLPHIN INTERNATIONAL
2004450441	CYOR	CYNOPS	ORIENTALIS	CHINESE DWARF	NEWT	LIV	4,780	NO	HK	HK	T	C	C	C	9/19/04	9/19/04	I	LA	DOLPHIN INTERNATIONAL
2004451431	TRIT	TRITURUS	SPECIES		NEWT	LIV	1,600	NO	HK	HK	T	C	C	C	8/26/04	8/26/04	I	TP	5-D TROPICAL, INC.
2004451442	TRIT	TRITURUS	SPECIES		NEWT	LIV	1,600	NO	HK	HK	T	C	C	C	7/22/04	7/21/04	I	TP	5-D TROPICAL, INC.
2004451509	TRIT	TRITURUS	SPECIES		NEWT	LIV	1,600	NO	HK	HK	T	C	C	C	7/30/04	7/28/04	I	TP	5-D TROPICAL, INC.
2004451655	TRIT	TRITURUS	SPECIES		NEWT	LIV	800	NO	HK	HK	T	C	C	C	8/20/04	8/18/04	I	TP	5-D TROPICAL, INC.
2004451790	SAL?	SALAMANDRA	SPECIES	FIRE	SALAMANDER	LIV	30	NO	DE	DE	T	C	C	C	9/22/04	9/22/04	I	DF	U.S GLOBAL EXOTICS INC
2004452579	PACB	PACHYTRITON	BREVIPES	TSITOU	NEWT	LIV	300	NO	HK	HK	T	C	C	C	9/26/04	9/26/04	I	LA	DOLPHIN INTERNATIONAL
2004452579	CYOR	CYNOPS	ORIENTALIS	CHINESE DWARF	NEWT	LIV	6,550	NO	HK	HK	T	C	C	C	9/26/04	9/26/04	I	LA	DOLPHIN INTERNATIONAL
2004452620	TRIH	TRITURUS	HONGKONGENSIS	ALPINE	NEWT	LIV	525	NO	HK	HK	T	W	C	C	9/26/04	9/26/04	I	LA	AQUACO, INC.
2004454958	TRIT	TRITURUS	SPECIES		NEWT	LIV	1,300	NO	HK	HK	T	C	C	C	10/2/04	10/2/04	I	LA	GLOBAL AQUATIC CONSULTING
2004454965	CYOR	CYNOPS	ORIENTALIS	CHINESE DWARF	NEWT	LIV	750	NO	HK	HK	T	C	C	C	10/2/04	10/2/04	I	LA	DOLPHIN INTERNATIONAL
2004455031	PACB	PACHYTRITON	BREVIPES	TSITOU	NEWT	LIV	400	NO	HK	HK	T	C	C	C	10/3/04	10/3/04	I	LA	DOLPHIN INTERNATIONAL
2004455031	CYOR	CYNOPS	ORIENTALIS	CHINESE DWARF	NEWT	LIV	4,518	NO	HK	HK	T	C	C	C	10/3/04	10/3/04	I	LA	DOLPHIN INTERNATIONAL
2004455033	TRIH	TRITURUS	HONGKONGENSIS	ALPINE	NEWT	LIV	150	NO	HK	HK	T	W	C	C	10/3/04	10/3/04	I	LA	AQUACO, INC.
2004455055	TRIT	TRITURUS	SPECIES		NEWT	LIV	1,200	NO	CN	HK	T	C	C	C	10/3/04	10/3/04	I	LA	AQUATIC CONNECTION
2004456791	TRIT	TRITURUS	SPECIES		NEWT	LIV	50	NO	SG	SG	T	W	C	C	9/16/04	9/16/04	I	CH	INTERCONTINENTAL IMPORTS, INC.
2004457566	TRIT	TRITURUS	SPECIES		NEWT	LIV	400	NO	HK	HK	T	C	C	C	10/9/04	10/9/04	I	LA	GLOBAL AQUATIC CONSULTING
2004457617	TRIT	TRITURUS	SPECIES		NEWT	LIV	155	NO	HK	HK	T	C	C	C	10/9/04	10/10/04	I	LA	TROPICAL ENTERPRISES
2004457618	TRIT	TRITURUS	SPECIES		NEWT	LIV	250	NO	HK	HK	T	W	C	C	10/9/04	10/10/04	I	LA	AQUACO, INC.
2004457624	PACB	PACHYTRITON	BREVIPES	TSITOU	NEWT	LIV	700	NO	HK	HK	T	C	C	C	10/10/04	10/10/04	I	LA	DOLPHIN INTERNATIONAL
2004457624	CYOR	CYNOPS	ORIENTALIS	CHINESE DWARF	NEWT	LIV	4,000	NO	HK	HK	T	C	C	C	10/10/04	10/10/04	I	LA	DOLPHIN INTERNATIONAL
2004457705	CNP?	CYNOPS	SPECIES	FIREBELLY	NEWT	LIV	12	NO	HK	HK	T	W	C	C	10/11/04	10/11/04	I	LA	Exemptions 6 and 7(C)
2004457705	CYOR	CYNOPS	ORIENTALIS	CHINESE DWARF	NEWT	LIV	41	NO	HK	HK	T	W	C	C	10/11/04	10/11/04	I	LA	Exemptions 6 and 7(C)
2004457705	CNP?	CYNOPS	SPECIES	FIREBELLY	NEWT	LIV	5	NO	HK	HK	T	W	C	C	10/11/04	10/11/04	I	LA	Exemptions 6 and 7(C)

2004457705	CNP?	CYNOPS	SPECIES	FIREBELLY	NEWT	LIV	5	NO	HK	HK	T	W	C	C	10/11/04	10/11/04	I	LA	Exemptions 6 and 7(C)
2004457705	TYL?	TYLOTOTRITON	SPECIES	CROCODILE	NEWT	LIV	5	NO	HK	HK	T	W	C	C	10/11/04	10/11/04	I	LA	Exemptions 6 and 7(C)
2004457705	NEW?	PACHYTRITON	SPECIES	CHINESE	NEWT	LIV	97	NO	HK	HK	T	W	C	C	10/11/04	10/11/04	I	LA	Exemptions 6 and 7(C)
2004457705	WNEW	PARAMESOTRITON	SPECIES	WARTY	NEWT	LIV	30	NO	HK	HK	T	W	C	C	10/11/04	10/11/04	I	LA	Exemptions 6 and 7(C)
2004457705	WNEW	PARAMESOTRITON	SPECIES	WARTY	NEWT	LIV	63	NO	HK	HK	T	W	C	C	10/11/04	10/11/04	I	LA	Exemptions 6 and 7(C)
2004458369	TRIH	TRITURUS	HONGKONGENSIS	ALPINE	NEWT	LIV	170	NO	HK	HK	T	C	C	C	8/23/04	8/23/04	I	NY	TRI-COUNTY TROPICALS, INC.
2004458700	TRIH	TRITURUS	HONGKONGENSIS	ALPINE	NEWT	LIV	260	NO	HK	HK	T	C	C	C	9/9/04	9/8/04	I	NY	TRI-COUNTY TROPICALS, INC.
2004458735	CNP?	CYNOPS	SPECIES	FIREBELLY	NEWT	LIV	150	NO	HK	HK	T	W	C	C	9/18/04	9/18/04	I	NY	TRANSSHIP DISCOUNTS , LTD.
2004458735	NEW?	PACHYTRITON	SPECIES	CHINESE	NEWT	LIV	450	NO	HK	HK	T	W	C	C	9/18/04	9/18/04	I	NY	TRANSSHIP DISCOUNTS , LTD.
2004458776	CYPY	CYNOPS	PYRRHOGASTER	JAPANESE FIREBELLY	NEWT	LIV	50	NO	HK	HK	T	W	C	C	9/13/04	9/11/04	I	NY	TRANSSHIP DISCOUNTS , LTD.
2004458776	PACB	PACHYTRITON	BREVIPES	TSITOU	NEWT	LIV	240	NO	HK	HK	T	W	C	C	9/13/04	9/11/04	I	NY	TRANSSHIP DISCOUNTS , LTD.
2004458917	CYPY	CYNOPS	PYRRHOGASTER	JAPANESE FIREBELLY	NEWT	LIV	400	NO	CN	CN	T	W	C	C	9/22/04	9/19/04	I	NY	FISH MART INC.
2004458959	CYPY	CYNOPS	PYRRHOGASTER	JAPANESE FIREBELLY	NEWT	LIV	50	NO	HK	HK	T	W	C	C	9/7/04	9/4/04	I	NY	TRANSSHIP DISCOUNTS , LTD.
2004458959	PACB	PACHYTRITON	BREVIPES	TSITOU	NEWT	LIV	60	NO	HK	HK	T	W	C	C	9/7/04	9/4/04	I	NY	TRANSSHIP DISCOUNTS , LTD.
2004459064	CYOR	CYNOPS	ORIENTALIS	CHINESE DWARF	NEWT	LIV	200	NO	SG	SG	T	W	C	C	9/9/04	8/29/04	I	NY	INTERNATIONAL PET RESOURCES LLC
2004459196	CYPY	CYNOPS	PYRRHOGASTER	JAPANESE FIREBELLY	NEWT	LIV	1,050	NO	HK	HK	T	W	C	C	9/13/04	9/11/04	I	NY	ERDA INC.
2004459259	SASA	SALAMANDRA	SALAMANDRA	EUROPEAN FIRE	SALAMANDER	LIV	85	NO	DE	DE	T	C	C	C	10/13/04	10/13/04	I	DF	U.S GLOBAL EXOTICS INC
2004459259	SAL?	SALAMANDRA	SPECIES	FIRE	SALAMANDER	LIV	4	NO	DE	DE	T	C	C	C	10/13/04	10/13/04	I	DF	U.S GLOBAL EXOTICS INC
2004459259	TRIT	TRITURUS	SPECIES		NEWT	LIV	15	NO	RU	DE	T	W	C	C	10/13/04	10/13/04	I	DF	U.S GLOBAL EXOTICS INC
2004459737	BODO	BOLITOGLOSSA	DOFLEINI	ALTA VERAPAZ	SALAMANDER	LIV	10	NO	NI	HN	T	C	C	C	10/14/04	10/14/04	I	LA	LA REPTILES INC
2004459760	CYPY	CYNOPS	PYRRHOGASTER	JAPANESE FIREBELLY	NEWT	LIV	170	NO	HK	HK	T	W	C	C	7/22/04	7/17/04	I	NY	TRANSSHIP DISCOUNTS , LTD.
2004459760	NEW?	PACHYTRITON	SPECIES	CHINESE	NEWT	LIV	60	NO	HK	HK	T	W	C	C	7/22/04	7/17/04	I	NY	TRANSSHIP DISCOUNTS , LTD.
2004460095	CYPY	CYNOPS	PYRRHOGASTER	JAPANESE FIREBELLY	NEWT	LIV	300	NO	CN	CN	T	W	C	C	8/31/04	8/29/04	I	NY	FISH MART INC.
2004460151	CYPY	CYNOPS	PYRRHOGASTER	JAPANESE FIREBELLY	NEWT	LIV	450	NO	HK	HK	T	W	C	C	7/28/04	7/24/04	I	NY	TRANSSHIP DISCOUNTS , LTD.
2004460151	PACB	PACHYTRITON	BREVIPES	TSITOU	NEWT	LIV	180	NO	HK	HK	T	W	C	C	7/28/04	7/24/04	I	NY	TRANSSHIP DISCOUNTS , LTD.
2004460294	CYPY	CYNOPS	PYRRHOGASTER	JAPANESE FIREBELLY	NEWT	LIV	200	NO	HK	HK	T	W	C	C	8/3/04	7/31/04	I	NY	TRANSSHIP DISCOUNTS , LTD.
2004460319	CYPY	CYNOPS	PYRRHOGASTER	JAPANESE FIREBELLY	NEWT	LIV	150	NO	HK	HK	T	W	C	C	8/2/04	8/21/04	I	NY	ERDA INC.
2004460514	PACB	PACHYTRITON	BREVIPES	TSITOU	NEWT	LIV	150	NO	HK	HK	T	W	C	C	8/30/04	8/28/04	I	NY	TRANSSHIP DISCOUNTS , LTD.
2004460709	CYPY	CYNOPS	PYRRHOGASTER	JAPANESE FIREBELLY	NEWT	LIV	680	NO	HK	HK	T	W	C	C	9/7/04	9/4/04	I	NY	ERDA INC.
2004461057	TRIT	TRITURUS	SPECIES		NEWT	LIV	800	NO	HK	HK	T	C	C	C	10/16/04	10/16/04	I	LA	GLOBAL AQUATIC CONSULTING
2004461061	CYOR	CYNOPS	ORIENTALIS	CHINESE DWARF	NEWT	LIV	500	NO	HK	HK	T	C	C	C	10/16/04	10/16/04	I	LA	DOLPHIN INTERNATIONAL
2004461155	CYOR	CYNOPS	ORIENTALIS	CHINESE DWARF	NEWT	LIV	4,025	NO	HK	HK	T	C	C	C	10/17/04	10/17/04	I	LA	DOLPHIN INTERNATIONAL
2004461159	TRIT	TRITURUS	SPECIES		NEWT	LIV	250	NO	HK	HK	T	C	C	C	10/17/04	10/17/04	I	LA	TROPICAL ENTERPRISES
2004461161	TRIH	TRITURUS	HONGKONGENSIS	ALPINE	NEWT	LIV	1,075	NO	HK	HK	T	W	C	C	10/17/04	10/17/04	I	LA	AQUACO, INC.
2004461174	TRIT	TRITURUS	SPECIES		NEWT	LIV	500	NO	CN	HK	T	C	C	C	10/17/04	10/17/04	I	LA	AQUATIC CONNECTION
2004462675	TYVE	TYLOTOTRITON	VERRUCOSUS	CROCODILE	NEWT	LIV	16	NO	SG	SG	T	W	C	C	8/13/04	8/14/04	I	NW	INTERNATIONAL PET RESOURCES LLC
2004465330	CYOR	CYNOPS	ORIENTALIS	CHINESE DWARF	NEWT	LIV	250	NO	HK	HK	T	C	C	C	10/23/04	10/23/04	I	LA	DOLPHIN INTERNATIONAL
2004465351	TRIT	TRITURUS	SPECIES		NEWT	LIV	1,200	NO	HK	HK	T	C	C	C	10/23/04	10/23/04	I	LA	GLOBAL AQUATIC CONSULTING
2004465398	TRIH	TRITURUS	HONGKONGENSIS	ALPINE	NEWT	LIV	600	NO	HK	HK	T	W	C	C	10/24/04	10/24/04	I	LA	AQUACO, INC.
2004465417	TRIT	TRITURUS	SPECIES		NEWT	LIV	63	NO	HK	HK	T	C	C	C	10/24/04	10/24/04	I	LA	TROPICAL ENTERPRISES
2004465418	CNP?	CYNOPS	SPECIES	FIREBELLY	NEWT	LIV	50	NO	SG	SG	T	C	C	C	10/24/04	10/24/04	I	LA	TROPICAL FISH CONSOLIDATORS LTD.
2004465440	CYOR	CYNOPS	ORIENTALIS	CHINESE DWARF	NEWT	LIV	7,350	NO	HK	HK	T	C	C	C	10/24/04	10/24/04	I	LA	DOLPHIN INTERNATIONAL
2004465440	PACB	PACHYTRITON	BREVIPES	TSITOU	NEWT	LIV	200	NO	HK	HK	T	C	C	C	10/24/04	10/24/04	I	LA	DOLPHIN INTERNATIONAL
2004466933	CNP?	CYNOPS	SPECIES	FIREBELLY	NEWT	LIV	1,117	NO	HK	HK	T	W	C	C	10/28/04	10/28/04	I	LA	U.S. GLOBAL EXOTICS, INC.
2004466933	TYL?	TYLOTOTRITON	SPECIES	CROCODILE	NEWT	LIV	77	NO	HK	HK	T	W	C	C	10/28/04	10/28/04	I	LA	U.S. GLOBAL EXOTICS, INC.
2004467830	CNP?	CYNOPS	SPECIES	FIREBELLY	NEWT	LIV	1,117	NO	HK	HK	T	W	C	C	10/29/04	10/28/04	I	LA	U.S. GLOBAL EXOTICS, INC.
2004467830	TYL?	TYLOTOTRITON	SPECIES	CROCODILE	NEWT	LIV	77	NO	HK	HK	T	W	C	C	10/29/04	10/28/04	I	LA	U.S. GLOBAL EXOTICS, INC.
2004468315	CYOR	CYNOPS	ORIENTALIS	CHINESE DWARF	NEWT	LIV	500	NO	HK	HK	T	C	C	C	10/30/04	10/30/04	I	LA	DOLPHIN INTERNATIONAL
2004468333	TRIT	TRITURUS	SPECIES		NEWT	LIV	600	NO	HK	HK	T	C	C	C	10/30/04	10/30/04	I	LA	GLOBAL AQUATIC CONSULTING
2004468338	CYOR	CYNOPS	ORIENTALIS	CHINESE DWARF	NEWT	LIV	2,500	NO	CN	CN	T	W	C	C	10/30/04	10/30/04	I	LA	Z IMPORTS INC.
2004468388	TRIH	TRITURUS	HONGKONGENSIS	ALPINE	NEWT	LIV	125	NO	HK	HK	T	W	C	C	10/30/04	10/31/04	I	LA	AQUACO, INC.
2004468398	CYOR	CYNOPS	ORIENTALIS	CHINESE DWARF	NEWT	LIV	2,250	NO	HK	HK	T	C	C	C	10/30/04	10/31/04	I	LA	DOLPHIN INTERNATIONAL
2004468398	PACB	PACHYTRITON	BREVIPES	TSITOU	NEWT	LIV	100	NO	HK	HK	T	C	C	C	10/30/04	10/31/04	I	LA	DOLPHIN INTERNATIONAL
2004469996	PACB	PACHYTRITON	BREVIPES	TSITOU	NEWT	LIV	90	NO	HK	HK	T	W	C	C	8/23/04	8/21/04	I	NY	TRANSSHIP DISCOUNTS , LTD.
2004470298	CYPY	CYNOPS	PYRRHOGASTER	JAPANESE FIREBELLY	NEWT	LIV	375	NO	HK	HK	T	W	C	C	9/27/04	9/25/04	I	NY	ERDA INCORPORATED
2004470743	CYOR	CYNOPS	ORIENTALIS	CHINESE DWARF	NEWT	LIV	50	NO	SG	SG	T	C	C	C	10/1/04	10/1/04	I	NY	NAME REMOVED
2004471155	CYOR	CYNOPS	ORIENTALIS	CHINESE DWARF	NEWT	LIV	3,000	NO	CN	CN	T	W	C	C	11/6/04	11/6/04	I	LA	Z IMPORTS INC.
2004471161	TRIT	TRITURUS	SPECIES		NEWT	LIV	200	NO	HK	HK	T	C	C	C	11/6/04	11/6/04	I	LA	GLOBAL AQUATIC CONSULTING
2004471169	CYOR	CYNOPS	ORIENTALIS	CHINESE DWARF	NEWT	LIV	1,250	NO	HK	HK	T	C	C	C	11/6/04	11/6/04	I	LA	DOLPHIN INTERNATIONAL
2004471169	PACB	PACHYTRITON	BREVIPES	TSITOU	NEWT	LIV	100	NO	HK	HK	T	C	C	C	11/6/04	11/6/04	I	LA	DOLPHIN INTERNATIONAL

2004471235	TRIH	TRITURUS	HONGKONGENSIS	ALPINE	NEWT	LIV	175	NO	HK	HK	T	W	C	C	11/6/04	11/7/04	I	LA	AQUACO, INC.
2004471253	CYOR	CYNOPS	ORIENTALIS	CHINESE DWARF	NEWT	LIV	1,750	NO	HK	HK	T	C	C	C	11/6/04	11/7/04	I	LA	DOLPHIN INTERNATIONAL
2004471253	PACB	PACHYTRITON	BREVIPES	TSITOU	NEWT	LIV	100	NO	HK	HK	T	C	C	C	11/6/04	11/7/04	I	LA	DOLPHIN INTERNATIONAL
2004471712	CNP?	CYNOPS	SPECIES	FIREBELLY	NEWT	LIV	600	NO	HK	HK	T	W	C	C	7/26/04	7/24/04	I	NY	ERDA INCORPORATED
2004471927	CYPY	CYNOPS	PYRRHOGASTER	JAPANESE FIREBELLY	NEWT	LIV	750	NO	HK	HK	T	W	C	C	10/4/04	10/2/04	I	NY	ERDA INCORPORATED
2004472249	CYPY	CYNOPS	PYRRHOGASTER	JAPANESE FIREBELLY	NEWT	LIV	300	NO	HK	HK	T	W	C	C	10/12/04	10/9/04	I	NY	ERDA INCORPORATED
2004472254	CYPY	CYNOPS	PYRRHOGASTER	JAPANESE FIREBELLY	NEWT	LIV	100	NO	HK	HK	T	W	C	C	10/12/04	10/9/04	I	NY	TRANSSHIP DISCOUNTS , LTD.
2004472254	PACB	PACHYTRITON	BREVIPES	TSITOU	NEWT	LIV	120	NO	HK	HK	T	W	C	C	10/12/04	10/9/04	I	NY	TRANSSHIP DISCOUNTS , LTD.
2004472307	CYOR	CYNOPS	ORIENTALIS	CHINESE DWARF	NEWT	LIV	200	NO	SG	SG	T	W	C	C	10/13/04	9/26/04	I	NY	INTERNATIONAL PET RESOURCES LLC
2004472468	CYPY	CYNOPS	PYRRHOGASTER	JAPANESE FIREBELLY	NEWT	LIV	350	NO	HK	HK	T	W	C	C	10/7/04	10/2/04	I	NY	TRANSSHIP DISCOUNTS , LTD.
2004472468	PACB	PACHYTRITON	BREVIPES	TSITOU	NEWT	LIV	300	NO	HK	HK	T	W	C	C	10/7/04	10/2/04	I	NY	TRANSSHIP DISCOUNTS , LTD.
2004473230	BODO	BOLITOGLOSSA	DOFLEINI	ALTA VERAPAZ	SALAMANDER	LIV	53	NO	HN	HN	T	W	C	C	11/10/04	11/10/04	I	LA	LA REPTILES INC
2004473230	BMEX	BOLITOGLOSSA	MEXICANA	MEX MUSHROOMTONGUE	SALAMANDER	LIV	1	NO	HN	HN	T	W	C	C	11/10/04	11/10/04	I	LA	LA REPTILES INC
2004474710	CYOR	CYNOPS	ORIENTALIS	CHINESE DWARF	NEWT	LIV	250	NO	HK	HK	T	C	C	C	11/13/04	11/13/04	I	LA	DOLPHIN INTERNATIONAL
2004474769	TRIH	TRITURUS	HONGKONGENSIS	ALPINE	NEWT	LIV	150	NO	HK	HK	T	W	C	C	11/13/04	11/14/04	I	LA	AQUACO, INC.
2004474790	CYOR	CYNOPS	ORIENTALIS	CHINESE DWARF	NEWT	LIV	1,450	NO	HK	HK	T	C	C	C	11/13/04	11/14/04	I	LA	DOLPHIN INTERNATIONAL
2004474790	PACB	PACHYTRITON	BREVIPES	TSITOU	NEWT	LIV	100	NO	HK	HK	T	C	C	C	11/13/04	11/14/04	I	LA	DOLPHIN INTERNATIONAL
2004474807	TRIT	TRITURUS	SPECIES		NEWT	LIV	300	NO	HK	HK	T	C	C	C	11/13/04	11/14/04	I	LA	GLOBAL AQUATIC CONSULTING
2004475811	CYPY	CYNOPS	PYRRHOGASTER	JAPANESE FIREBELLY	NEWT	LIV	355	NO	HK	HK	T	W	C	C	8/10/04	8/7/04	I	NY	TRANSSHIP DISCOUNTS , LTD.
2004475811	PACB	PACHYTRITON	BREVIPES	TSITOU	NEWT	LIV	185	NO	HK	HK	T	W	C	C	8/10/04	8/7/04	I	NY	TRANSSHIP DISCOUNTS , LTD.
2004476458	CYOR	CYNOPS	ORIENTALIS	CHINESE DWARF	NEWT	LIV	200	NO	CN	CN	T	F	C	C	10/13/04	10/6/04	I	NY	MIN HUA ENTERPRISE, INC.
2004477185	TRIH	TRITURUS	HONGKONGENSIS	ALPINE	NEWT	LIV	600	NO	HK	HK	T	W	C	C	10/29/04	10/17/04	I	NY	ALL AMERICAN TRANSHIP
2004478072	CYOR	CYNOPS	ORIENTALIS	CHINESE DWARF	NEWT	LIV	1,000	NO	HK	HK	T	C	C	C	11/20/04	11/20/04	I	LA	DOLPHIN INTERNATIONAL
2004478072	PACB	PACHYTRITON	BREVIPES	TSITOU	NEWT	LIV	100	NO	HK	HK	T	C	C	C	11/20/04	11/20/04	I	LA	DOLPHIN INTERNATIONAL
2004478081	TRIT	TRITURUS	SPECIES		NEWT	LIV	800	NO	HK	HK	T	C	C	C	11/20/04	11/20/04	I	LA	GLOBAL AQUATIC CONSULTING
2004478177	CYOR	CYNOPS	ORIENTALIS	CHINESE DWARF	NEWT	LIV	1,600	NO	HK	HK	T	C	C	C	11/21/04	11/21/04	I	LA	DOLPHIN INTERNATIONAL
2004478177	PACB	PACHYTRITON	BREVIPES	TSITOU	NEWT	LIV	200	NO	HK	HK	T	C	C	C	11/21/04	11/21/04	I	LA	DOLPHIN INTERNATIONAL
2004478195	TRIH	TRITURUS	HONGKONGENSIS	ALPINE	NEWT	LIV	250	NO	HK	HK	T	W	C	C	11/21/04	11/21/04	I	LA	AQUACO, INC.
2004478607	CYOR	CYNOPS	ORIENTALIS	CHINESE DWARF	NEWT	LIV	200	NO	SG	SG	T	W	C	C	9/30/04	9/19/04	I	NY	INTERNATIONAL PET RESOURCES LLC
2004479836	CYOR	CYNOPS	ORIENTALIS	CHINESE DWARF	NEWT	LIV	500	NO	HK	HK	T	C	C	C	11/27/04	11/27/04	I	LA	DOLPHIN INTERNATIONAL
2004479836	PACB	PACHYTRITON	BREVIPES	TSITOU	NEWT	LIV	100	NO	HK	HK	T	C	C	C	11/27/04	11/27/04	I	LA	DOLPHIN INTERNATIONAL
2004479844	TRIT	TRITURUS	SPECIES		NEWT	LIV	1,200	NO	HK	HK	T	C	C	C	11/27/04	11/27/04	I	LA	GLOBAL AQUATIC CONSULTING
2004479848	CYOR	CYNOPS	ORIENTALIS	CHINESE DWARF	NEWT	LIV	4,000	NO	CN	CN	T	W	C	C	11/27/04	11/27/04	I	LA	Z IMPORTS INC.
2004479911	TRIT	TRITURUS	SPECIES		NEWT	LIV	200	NO	HK	HK	T	C	C	C	11/27/04	11/28/04	I	LA	TROPICAL ENTERPRISES
2004479912	TRIH	TRITURUS	HONGKONGENSIS	ALPINE	NEWT	LIV	325	NO	HK	HK	T	W	C	C	11/27/04	11/28/04	I	LA	AQUACO, INC.
2004479920	CYOR	CYNOPS	ORIENTALIS	CHINESE DWARF	NEWT	LIV	1,600	NO	HK	HK	T	C	C	C	11/27/04	11/28/04	I	LA	DOLPHIN INTERNATIONAL
2004479920	PACB	PACHYTRITON	BREVIPES	TSITOU	NEWT	LIV	100	NO	HK	HK	T	C	C	C	11/27/04	11/28/04	I	LA	DOLPHIN INTERNATIONAL
2004479993	CYPY	CYNOPS	PYRRHOGASTER	JAPANESE FIREBELLY	NEWT	LIV	825	NO	HK	HK	T	W	C	C	9/20/04	9/18/04	I	NY	ERDA INCORPORATED
2004480346	CYPY	CYNOPS	PYRRHOGASTER	JAPANESE FIREBELLY	NEWT	LIV	600	NO	HK	HK	T	W	C	C	11/8/04	11/6/04	I	NY	ERDA INCORPORATED
2004480366	CNP?	CYNOPS	SPECIES	FIREBELLY	NEWT	LIV	412	NO	HK	HK	T	W	C	C	11/2/04	10/30/04	I	NY	TRANSSHIP DISCOUNTS , LTD.
2004480519	CYPY	CYNOPS	PYRRHOGASTER	JAPANESE FIREBELLY	NEWT	LIV	710	NO	HK	HK	T	W	C	C	10/27/04	10/23/04	I	NY	TRANSSHIP DISCOUNTS , LTD.
2004480857	CYOR	CYNOPS	ORIENTALIS	CHINESE DWARF	NEWT	LIV	100	NO	SG	SG	T	W	C	C	10/22/04	10/10/04	I	NY	INTERNATIONAL PET RESOURCES LLC
2004484570	CYOR	CYNOPS	ORIENTALIS	CHINESE DWARF	NEWT	LIV	350	NO	HK	HK	T	C	C	C	12/4/04	12/4/04	I	LA	DOLPHIN INTERNATIONAL
2004484637	TRIT	TRITURUS	SPECIES		NEWT	LIV	125	NO	HK	HK	T	C	C	C	12/4/04	12/5/04	I	LA	TROPICAL ENTERPRISES
2004484640	TRIH	TRITURUS	HONGKONGENSIS	ALPINE	NEWT	LIV	600	NO	HK	HK	T	W	C	C	12/4/04	12/5/04	I	LA	AQUACO, INC.
2004484645	CYOR	CYNOPS	ORIENTALIS	CHINESE DWARF	NEWT	LIV	2,200	NO	HK	HK	T	C	C	C	12/4/04	12/5/04	I	LA	DOLPHIN INTERNATIONAL
2004484645	PACB	PACHYTRITON	BREVIPES	TSITOU	NEWT	LIV	100	NO	HK	HK	T	C	C	C	12/4/04	12/5/04	I	LA	DOLPHIN INTERNATIONAL
2004484665	TRIT	TRITURUS	SPECIES		NEWT	LIV	700	NO	HK	HK	T	C	C	C	12/4/04	12/5/04	I	LA	GLOBAL AQUATIC CONSULTING
2004488333	PCAU	PARAMESOTRITON	CAUDOPUNCTATUS	GUIZHOU WARTY	NEWT	LIV	360	NO	HK	HK	T	W	C	C	10/25/04	10/25/04	I	MI	XTREME REPTILES
2004488333	PLAB	PACHYTRITON	LABIATUS	UNTERSTEIN'S	NEWT	LIV	500	NO	HK	HK	T	W	C	C	10/25/04	10/25/04	I	MI	XTREME REPTILES
2004489209	CYOR	CYNOPS	ORIENTALIS	CHINESE DWARF	NEWT	LIV	1,000	NO	CN	CN	T	W	C	C	12/11/04	12/11/04	I	LA	Z IMPORTS INC.
2004489223	CYOR	CYNOPS	ORIENTALIS	CHINESE DWARF	NEWT	LIV	750	NO	HK	HK	T	C	C	C	12/11/04	12/11/04	I	LA	DOLPHIN INTERNATIONAL
2004489296	TRIT	TRITURUS	SPECIES		NEWT	LIV	600	NO	HK	HK	T	C	C	C	12/12/04	12/12/04	I	LA	GLOBAL AQUATIC CONSULTING
2004489298	TRIT	TRITURUS	SPECIES		NEWT	LIV	60	NO	HK	HK	T	C	C	C	12/12/04	12/12/04	I	LA	TROPICAL ENTERPRISES
2004489314	CYOR	CYNOPS	ORIENTALIS	CHINESE DWARF	NEWT	LIV	6,075	NO	HK	HK	T	C	C	C	12/12/04	12/12/04	I	LA	DOLPHIN INTERNATIONAL
2004489314	PACB	PACHYTRITON	BREVIPES	TSITOU	NEWT	LIV	200	NO	HK	HK	T	C	C	C	12/12/04	12/12/04	I	LA	DOLPHIN INTERNATIONAL
2004489337	TRIH	TRITURUS	HONGKONGENSIS	ALPINE	NEWT	LIV	525	NO	HK	HK	T	W	C	C	12/12/04	12/12/04	I	LA	AQUACO, INC.
2004489910	TRIT	TRITURUS	SPECIES		NEWT	LIV	1,600	NO	HK	HK	T	C	C	C	10/1/04	9/29/04	I	TP	5-D TROPICAL, INC.
2004490324	TRIT	TRITURUS	SPECIES		NEWT	LIV	800	NO	HK	HK	T	C	C	C	11/18/04	11/10/04	I	TP	5-D TROPICAL, INC.
2004490406	TRIT	TRITURUS	SPECIES		NEWT	LIV	1,600	NO	HK	HK	T	C	C	C	10/19/04	10/20/04	I	TP	5-D TROPICAL, INC.

2004490427	TRIH	TRITURUS	HONGKONGENSIS	ALPINE	NEWT	LIV	1,600	NO	HK	HK	T	C	C	C	9/16/04	9/15/04	I	TP	5-D TROPICAL, INC.
2004490832	TYL?	TYLOTOTRITON	SPECIES	CROCODILE	NEWT	LIV	3	NO	CN	CN	T	W	C	C	12/9/04	12/9/04	I	SE	Exemptions 6 and 7(C)
2004490832	TYL?	TYLOTOTRITON	SPECIES	CROCODILE	NEWT	LIV	3	NO	CN	CN	T	W	C	C	12/9/04	12/9/04	I	SE	Exemptions 6 and 7(C)
2004490832	PCAU	PARAMESOTRITON	CAUDOPUNCTATUS	GUIZHOU WARTY	NEWT	LIV	5	NO	CN	CN	T	W	C	C	12/9/04	12/9/04	I	SE	Exemptions 6 and 7(C)
2004490832	PCHI	PARAMESOTRITON	CHINENSIS	CHINESE WARTY	NEWT	LIV	5	NO	CN	CN	T	W	C	C	12/9/04	12/9/04	I	SE	Exemptions 6 and 7(C)
2004490832	PLAB	PACHYTRITON	LABIATUS	UNTERSTEIN'S	NEWT	LIV	200	NO	CN	CN	T	W	C	C	12/9/04	12/9/04	I	SE	Exemptions 6 and 7(C)
2004490832	NEW?	PACHYTRITON	SPECIES	CHINESE	NEWT	LIV	4	NO	CN	CN	T	W	C	C	12/9/04	12/9/04	I	SE	Exemptions 6 and 7(C)
2004490832	NEW?	PACHYTRITON	SPECIES	CHINESE	NEWT	LIV	2	NO	CN	CN	T	W	C	C	12/9/04	12/9/04	I	SE	Exemptions 6 and 7(C)
2004490832	CNP?	CYNOPS	SPECIES	FIREBELLY	NEWT	LIV	10	NO	CN	CN	T	W	C	C	12/9/04	12/9/04	I	SE	Exemptions 6 and 7(C)
2004490840	BODO	BOLITOGLOSSA	DOFLEINI	ALTA VERAPAZ	SALAMANDER	LIV	20	NO	NI	HN	T	C	C	C	12/15/04	12/15/04	I	LA	LA REPTILES INC
2004490867	CNP?	CYNOPS	SPECIES	FIREBELLY	NEWT	LIV	1,975	NO	HK	HK	T	W	C	C	12/15/04	12/15/04	I	LA	U.S. GLOBAL EXOTICS, INC.
2004492025	CYOR	CYNOPS	ORIENTALIS	CHINESE DWARF	NEWT	LIV	1,000	NO	HK	HK	T	C	C	C	12/18/04	12/18/04	I	LA	DOLPHIN INTERNATIONAL
2004492054	TRIT	TRITURUS	SPECIES		NEWT	LIV	1,200	NO	HK	HK	T	C	C	C	12/19/04	12/19/04	I	LA	GLOBAL AQUATIC CONSULTING
2004492077	TRIH	TRITURUS	HONGKONGENSIS	ALPINE	NEWT	LIV	200	NO	HK	HK	T	W	C	C	12/19/04	12/19/04	I	LA	AQUACO, INC.
2004492596	CYPY	CYNOPS	PYRRHOGASTER	JAPANESE FIREBELLY	NEWT	LIV	100	NO	CN	CN	T	W	C	C	11/12/04	11/7/04	I	NY	FISH MART INCORPORATED
2004492596	CNP?	CYNOPS	SPECIES	FIREBELLY	NEWT	LIV	100	NO	CN	CN	T	W	C	C	11/12/04	11/7/04	I	NY	FISH MART INCORPORATED
2004492735	CYOR	CYNOPS	ORIENTALIS	CHINESE DWARF	NEWT	LIV	200	NO	CN	CN	T	F	C	C	10/28/04	10/20/04	I	NY	MIN HUA ENTERPRISE, INC.
2004493346	CYPY	CYNOPS	PYRRHOGASTER	JAPANESE FIREBELLY	NEWT	LIV	20	NO	HK	HK	T	W	C	C	11/15/04	11/13/04	I	NY	TRANSSHIP DISCOUNTS , LTD.
2004493346	PACB	PACHYTRITON	BREVIPES	TSITOU	NEWT	LIV	480	NO	HK	HK	T	W	C	C	11/15/04	11/13/04	I	NY	TRANSSHIP DISCOUNTS , LTD.
2004493430	CYOR	CYNOPS	ORIENTALIS	CHINESE DWARF	NEWT	LIV	100	NO	SG	SG	T	W	C	C	11/8/04	11/8/04	I	NY	AQUATIC LIFE SUPPORT
2004493721	CYPY	CYNOPS	PYRRHOGASTER	JAPANESE FIREBELLY	NEWT	LIV	200	NO	HK	HK	T	W	C	C	11/15/04	11/13/04	I	NY	ERDA INCORPORATED
2004494102	CYPY	CYNOPS	PYRRHOGASTER	JAPANESE FIREBELLY	NEWT	LIV	450	NO	HK	HK	T	W	C	C	10/18/04	10/16/04	I	NY	ERDA INCORPORATED
2004494299	PACB	PACHYTRITON	BREVIPES	TSITOU	NEWT	LIV	60	NO	HK	HK	T	W	C	C	9/28/04	9/25/04	I	NY	TRANSSHIP DISCOUNTS , LTD.
2004494347	CYPY	CYNOPS	PYRRHOGASTER	JAPANESE FIREBELLY	NEWT	LIV	425	NO	HK	HK	T	W	C	C	11/30/04	11/27/04	I	NY	ERDA INCORPORATED
2004494614	CYOR	CYNOPS	ORIENTALIS	CHINESE DWARF	NEWT	LIV	500	NO	HK	HK	T	C	C	C	12/25/04	12/25/04	I	LA	DOLPHIN INTERNATIONAL
2004494627	CYOR	CYNOPS	ORIENTALIS	CHINESE DWARF	NEWT	LIV	1,800	NO	HK	HK	T	C	C	C	12/25/04	12/26/04	I	LA	DOLPHIN INTERNATIONAL
2004494627	PACB	PACHYTRITON	BREVIPES	TSITOU	NEWT	LIV	400	NO	ID	HK	T	C	C	C	12/25/04	12/26/04	I	LA	DOLPHIN INTERNATIONAL
2004494629	TRIH	TRITURUS	HONGKONGENSIS	ALPINE	NEWT	LIV	600	NO	HK	HK	T	W	C	C	12/25/04	12/26/04	I	LA	AQUACO, INC.
2004494641	TRIT	TRITURUS	SPECIES		NEWT	LIV	800	NO	HK	HK	T	C	C	C	12/26/04	12/26/04	I	LA	GLOBAL AQUATIC CONSULTING
2005495720	CYOR	CYNOPS	ORIENTALIS	CHINESE DWARF	NEWT	LIV	1,000	NO	HK	HK	T	C	C	C	1/1/05	1/1/05	I	LA	DOLPHIN INTERNATIONAL
2005495720	PACB	PACHYTRITON	BREVIPES	TSITOU	NEWT	LIV	100	NO	HK	HK	T	C	C	C	1/1/05	1/1/05	I	LA	DOLPHIN INTERNATIONAL
2005495725	CYOR	CYNOPS	ORIENTALIS	CHINESE DWARF	NEWT	LIV	3,000	NO	CN	CN	T	C	C	C	1/1/05	1/1/05	I	LA	Z IMPORTS INC.
2005495739	CYOR	CYNOPS	ORIENTALIS	CHINESE DWARF	NEWT	LIV	650	NO	HK	HK	T	C	C	C	1/2/05	1/2/05	I	LA	DOLPHIN INTERNATIONAL
2005495739	PACB	PACHYTRITON	BREVIPES	TSITOU	NEWT	LIV	100	NO	ID	HK	T	C	C	C	1/2/05	1/2/05	I	LA	DOLPHIN INTERNATIONAL
2005495758	TRIH	TRITURUS	HONGKONGENSIS	ALPINE	NEWT	LIV	375	NO	HK	HK	T	W	C	C	1/2/05	1/2/05	I	LA	AQUACO, INC.
2005495769	TRIT	TRITURUS	SPECIES		NEWT	LIV	800	NO	HK	HK	T	C	C	C	1/2/05	1/2/05	I	LA	GLOBAL AQUATIC CONSULTING
2005496224	CYPY	CYNOPS	PYRRHOGASTER	JAPANESE FIREBELLY	NEWT	LIV	610	NO	HK	HK	T	W	C	C	10/18/04	10/16/04	I	NY	TRANSSHIP DISCOUNTS , LTD.
2005496405	CYPY	CYNOPS	PYRRHOGASTER	JAPANESE FIREBELLY	NEWT	LIV	350	NO	HK	HK	T	W	C	C	11/22/04	11/20/04	I	NY	ERDA INCORPORATED
2005496634	CYOR	CYNOPS	ORIENTALIS	CHINESE DWARF	NEWT	LIV	200	NO	CN	CN	T	F	C	C	10/27/04	10/27/04	I	NY	MIN HUA ENTERPRISE, INC.
2005498107	TRIT	TRITURUS	SPECIES		NEWT	LIV	20	NO	SG	SG	T	W	C	C	12/16/04	12/16/04	I	CH	INTERCONTINENTAL IMPORTS, INC.
2005498682	CYOR	CYNOPS	ORIENTALIS	CHINESE DWARF	NEWT	LIV	1,500	NO	CN	CN	T	C	C	C	1/8/05	1/8/05	I	LA	Z IMPORTS INC.
2005498688	CYOR	CYNOPS	ORIENTALIS	CHINESE DWARF	NEWT	LIV	1,000	NO	HK	HK	T	C	C	C	1/8/05	1/8/05	I	LA	DOLPHIN INTERNATIONAL
2005498752	TRIT	TRITURUS	SPECIES		NEWT	LIV	1,000	NO	HK	HK	T	C	C	C	1/8/05	1/9/05	I	LA	GLOBAL AQUATIC CONSULTING
2005498757	CYOR	CYNOPS	ORIENTALIS	CHINESE DWARF	NEWT	LIV	3,050	NO	HK	HK	T	C	C	C	1/8/05	1/9/05	I	LA	DOLPHIN INTERNATIONAL
2005498757	PACB	PACHYTRITON	BREVIPES	TSITOU	NEWT	LIV	100	NO	ID	HK	T	C	C	C	1/8/05	1/9/05	I	LA	DOLPHIN INTERNATIONAL
2005498794	TRIH	TRITURUS	HONGKONGENSIS	ALPINE	NEWT	LIV	500	NO	HK	HK	T	W	C	C	1/9/05	1/9/05	I	LA	AQUACO, INC.
2005502070	CYOR	CYNOPS	ORIENTALIS	CHINESE DWARF	NEWT	LIV	500	NO	HK	HK	T	C	C	C	1/15/05	1/15/05	I	LA	DOLPHIN INTERNATIONAL
2005502163	CYOR	CYNOPS	ORIENTALIS	CHINESE DWARF	NEWT	LIV	750	NO	HK	HK	T	C	C	C	1/16/05	1/16/05	I	LA	DOLPHIN INTERNATIONAL
2005502163	PACB	PACHYTRITON	BREVIPES	TSITOU	NEWT	LIV	200	NO	ID	HK	T	C	C	C	1/16/05	1/16/05	I	LA	DOLPHIN INTERNATIONAL
2005502170	TRIT	TRITURUS	SPECIES		NEWT	LIV	200	NO	HK	HK	T	C	C	C	1/16/05	1/16/05	I	LA	AQUATIC CONNECTION
2005502174	TRIH	TRITURUS	HONGKONGENSIS	ALPINE	NEWT	LIV	650	NO	HK	HK	T	W	C	C	1/16/05	1/16/05	I	LA	AQUACO, INC.
2005502201	TRIT	TRITURUS	SPECIES		NEWT	LIV	1,800	NO	HK	HK	T	C	C	C	1/16/05	1/16/05	I	LA	GLOBAL AQUATIC CONSULTING
2005505643	CYPY	CYNOPS	PYRRHOGASTER	JAPANESE FIREBELLY	NEWT	LIV	375	NO	HK	HK	T	W	C	C	11/2/04	10/23/04	I	NY	ERDA INCORPORATED
2005505847	CYOR	CYNOPS	ORIENTALIS	CHINESE DWARF	NEWT	LIV	750	NO	CN	CN	T	C	C	C	1/22/05	1/22/05	I	LA	Z IMPORTS INC.
2005505848	TRIT	TRITURUS	SPECIES		NEWT	LIV	1,400	NO	HK	HK	T	C	C	C	1/22/05	1/22/05	I	LA	GLOBAL AQUATIC CONSULTING
2005505942	TRIT	TRITURUS	SPECIES		NEWT	LIV	250	NO	HK	HK	T	W	C	C	1/23/05	1/23/05	I	LA	TROPICAL ENTERPRISES
2005505962	CYOR	CYNOPS	ORIENTALIS	CHINESE DWARF	NEWT	LIV	550	NO	HK	HK	T	C	C	C	1/23/05	1/23/05	I	LA	DOLPHIN INTERNATIONAL
2005505976	TRIH	TRITURUS	HONGKONGENSIS	ALPINE	NEWT	LIV	250	NO	HK	HK	T	W	C	C	1/23/05	1/23/05	I	LA	AQUACO, INC.
2005506418	CYPY	CYNOPS	PYRRHOGASTER	JAPANESE FIREBELLY	NEWT	LIV	100	NO	CN	CN	T	W	C	C	12/2/04	11/28/04	I	NY	FISH MART INC.
2005506418	CNP?	CYNOPS	SPECIES	FIREBELLY	NEWT	LIV	50	NO	CN	CN	T	W	C	C	12/2/04	11/28/04	I	NY	FISH MART INC.

2005506474	CYPY	CYNOPS	PYRRHOGASTER	JAPANESE FIREBELLY	NEWT	LIV	100	NO	CN	CN	T	W	C	C	12/22/04	12/19/04	I	NY	FISH MART INCORPORATED
2005506474	CNP?	CYNOPS	SPECIES	FIREBELLY	NEWT	LIV	75	NO	CN	CN	T	W	C	C	12/22/04	12/19/04	I	NY	FISH MART INCORPORATED
2005506998	CYPY	CYNOPS	PYRRHOGASTER	JAPANESE FIREBELLY	NEWT	LIV	387	NO	HK	HK	T	W	C	C	8/10/04	8/7/04	I	NY	ERDA INCORPORATED
2005507012	CYPY	CYNOPS	PYRRHOGASTER	JAPANESE FIREBELLY	NEWT	LIV	270	NO	HK	HK	T	W	C	C	11/20/04	11/20/04	I	NY	TRANSSHIP DISCOUNTS , LTD.
2005507012	PACB	PACHYTRITON	BREVIPES	TSITOU	NEWT	LIV	60	NO	HK	HK	T	W	C	C	11/20/04	11/20/04	I	NY	TRANSSHIP DISCOUNTS , LTD.
2005507030	CYPY	CYNOPS	PYRRHOGASTER	JAPANESE FIREBELLY	NEWT	LIV	50	NO	HK	HK	T	W	C	C	11/29/04	11/27/04	I	NY	TRANSSHIP DISCOUNTS , LTD.
2005507131	CYPY	CYNOPS	PYRRHOGASTER	JAPANESE FIREBELLY	NEWT	LIV	370	NO	HK	HK	T	W	C	C	12/20/04	12/18/04	I	NY	TRANSSHIP DISCOUNTS , LTD.
2005507131	PACB	PACHYTRITON	BREVIPES	TSITOU	NEWT	LIV	30	NO	HK	HK	T	W	C	C	12/20/04	12/18/04	I	NY	TRANSSHIP DISCOUNTS , LTD.
2005508569	CYOR	CYNOPS	ORIENTALIS	CHINESE DWARF	NEWT	LIV	600	NO	CN	CN	T	C	C	C	1/29/05	1/29/05	I	LA	Z IMPORTS INC.
2005508599	CYOR	CYNOPS	ORIENTALIS	CHINESE DWARF	NEWT	LIV	775	NO	HK	HK	T	C	C	C	1/29/05	1/30/05	I	LA	DOLPHIN INTERNATIONAL
2005508622	TRIH	TRITURUS	HONGKONGENSIS	ALPINE	NEWT	LIV	125	NO	VS	HK	T	W	C	C	1/30/05	1/30/05	I	LA	AQUACO, INC.
2005510600	PCAU	PARAMESOTRITON	CAUDOPUNCTATUS	GUIZHOU WARTY	NEWT	LIV	52	NO	CN	CN	T	W	C	C	2/3/05	2/3/05	I	SE	Exemptions 6 and 7(C)
2005510600	PCHI	PARAMESOTRITON	CHINENSIS	CHINESE WARTY	NEWT	LIV	100	NO	CN	CN	T	W	C	C	2/3/05	2/3/05	I	SE	Exemptions 6 and 7(C)
2005510600	PLAB	PACHYTRITON	LABIATUS	UNTERSTEIN'S	NEWT	LIV	500	NO	CN	CN	T	W	C	C	2/3/05	2/3/05	I	SE	Exemptions 6 and 7(C)
2005510600	NEW?	PACHYTRITON	SPECIES	CHINESE	NEWT	LIV	30	NO	CN	CN	T	W	C	C	2/3/05	2/3/05	I	SE	Exemptions 6 and 7(C)
2005510600	TYL?	TYLOTOTRITON	SPECIES	CROCODILE	NEWT	LIV	7	NO	CN	CN	T	W	C	C	2/3/05	2/3/05	I	SE	Exemptions 6 and 7(C)
2005510600	NEW?	PACHYTRITON	SPECIES	CHINESE	NEWT	LIV	20	NO	CN	CN	T	W	C	C	2/3/05	2/3/05	I	SE	Exemptions 6 and 7(C)
2005510600	CNP?	CYNOPS	SPECIES	FIREBELLY	NEWT	LIV	50	NO	CN	CN	T	W	C	C	2/3/05	2/3/05	I	SE	Exemptions 6 and 7(C)
2005510600	PSHG	PACHYHYNOBIUS	SHANGCHENGENSIS	SHANGCHENG	SALAMANDER	LIV	20	NO	CN	CN	T	W	C	C	2/3/05	2/3/05	I	SE	Exemptions 6 and 7(C)
2005510963	CYOR	CYNOPS	ORIENTALIS	CHINESE DWARF	NEWT	LIV	1,200	NO	CN	CN	T	C	C	C	2/5/05	2/5/05	I	LA	Z IMPORTS INC.
2005511034	TRIH	TRITURUS	HONGKONGENSIS	ALPINE	NEWT	LIV	75	NO	HK	HK	T	W	C	C	2/5/05	2/6/05	I	LA	AQUACO, INC.
2005511051	CYOR	CYNOPS	ORIENTALIS	CHINESE DWARF	NEWT	LIV	200	NO	HK	HK	T	C	C	C	2/5/05	2/6/05	I	LA	DOLPHIN INTERNATIONAL
2005513996	CYOR	CYNOPS	ORIENTALIS	CHINESE DWARF	NEWT	LIV	100	NO	HK	HK	T	C	C	C	2/12/05	2/12/05	I	LA	DOLPHIN INTERNATIONAL
2005514051	CYOR	CYNOPS	ORIENTALIS	CHINESE DWARF	NEWT	LIV	400	NO	HK	HK	T	C	C	C	2/12/05	2/13/05	I	LA	DOLPHIN INTERNATIONAL
2005516938	CYOR	CYNOPS	ORIENTALIS	CHINESE DWARF	NEWT	LIV	800	NO	CN	CN	T	C	C	C	2/19/05	2/19/05	I	LA	Z IMPORTS INC.
2005516996	CYOR	CYNOPS	ORIENTALIS	CHINESE DWARF	NEWT	LIV	475	NO	HK	HK	T	C	C	C	2/19/05	2/20/05	I	LA	DOLPHIN INTERNATIONAL
2005517005	TRIH	TRITURUS	HONGKONGENSIS	ALPINE	NEWT	LIV	25	NO	HK	HK	T	W	C	C	2/20/05	2/20/05	I	LA	AQUACO, INC.
2005517362	CYPY	CYNOPS	PYRRHOGASTER	JAPANESE FIREBELLY	NEWT	LIV	250	NO	HK	HK	T	W	C	C	2/14/05	2/12/05	I	NY	ERDA INCORPORATED
2005518617	CYOR	CYNOPS	ORIENTALIS	CHINESE DWARF	NEWT	LIV	200	NO	CN	CN	T	F	C	C	11/17/04	11/10/04	I	NY	MIN HUA ENTERPRISE, INC.
2005518864	CYPY	CYNOPS	PYRRHOGASTER	JAPANESE FIREBELLY	NEWT	LIV	50	NO	CN	CN	T	W	C	C	12/8/04	12/5/04	I	NY	FISH MART INCORPORATED
2005518864	CNP?	CYNOPS	SPECIES	FIREBELLY	NEWT	LIV	50	NO	CN	CN	T	W	C	C	12/8/04	12/5/04	I	NY	FISH MART INCORPORATED
2005519028	CYOR	CYNOPS	ORIENTALIS	CHINESE DWARF	NEWT	LIV	200	NO	CN	CN	T	F	C	C	12/6/04	11/24/04	I	NY	MIN HUA ENTERPRISE, INC.
2005519152	TRIH	TRITURUS	HONGKONGENSIS	ALPINE	NEWT	LIV	400	NO	HK	HK	T	W	C	C	12/13/04	12/5/04	I	NY	ALL AMERICAN TRANSHIP
2005519394	CYPY	CYNOPS	PYRRHOGASTER	JAPANESE FIREBELLY	NEWT	LIV	660	NO	HK	HK	T	W	C	C	12/7/04	12/4/04	I	NY	TRANSSHIP DISCOUNTS , LTD.
2005519394	PACB	PACHYTRITON	BREVIPES	TSITOU	NEWT	LIV	240	NO	HK	HK	T	W	C	C	12/7/04	12/4/04	I	NY	TRANSSHIP DISCOUNTS , LTD.
2005519522	CYPY	CYNOPS	PYRRHOGASTER	JAPANESE FIREBELLY	NEWT	LIV	60	NO	HK	HK	T	W	C	C	1/25/05	1/22/05	I	NY	TRANSSHIP DISCOUNTS , LTD.
2005519627	CYOR	CYNOPS	ORIENTALIS	CHINESE DWARF	NEWT	LIV	100	NO	HK	HK	T	C	C	C	2/26/05	2/26/05	I	LA	DOLPHIN INTERNATIONAL
2005519733	CYOR	CYNOPS	ORIENTALIS	CHINESE DWARF	NEWT	LIV	100	NO	HK	HK	T	C	C	C	2/27/05	2/27/05	I	LA	DOLPHIN INTERNATIONAL
2005519733	PACB	PACHYTRITON	BREVIPES	TSITOU	NEWT	LIV	300	NO	HK	HK	T	C	C	C	2/27/05	2/27/05	I	LA	DOLPHIN INTERNATIONAL
2005519777	CYPY	CYNOPS	PYRRHOGASTER	JAPANESE FIREBELLY	NEWT	LIV	320	NO	HK	HK	T	W	C	C	1/3/05	1/1/05	I	NY	TRANSSHIP DISCOUNTS , LTD.
2005519792	CYPY	CYNOPS	PYRRHOGASTER	JAPANESE FIREBELLY	NEWT	LIV	425	NO	HK	HK	T	W	C	C	12/7/04	12/4/04	I	NY	ERDA INCORPORATED
2005520033	CYPY	CYNOPS	PYRRHOGASTER	JAPANESE FIREBELLY	NEWT	LIV	100	NO	CN	CN	T	W	C	C	1/13/05	1/9/05	I	NY	FISH MART INCORPORATED
2005520033	CNP?	CYNOPS	SPECIES	FIREBELLY	NEWT	LIV	75	NO	CN	CN	T	W	C	C	1/13/05	1/9/05	I	NY	FISH MART INCORPORATED
2005520253	TRIH	TRITURUS	HONGKONGENSIS	ALPINE	NEWT	LIV	400	NO	HK	HK	T	W	C	C	1/24/05	1/9/05	I	NY	INTERNATIONAL PET RESOURCES LLC
2005520597	CYOR	CYNOPS	ORIENTALIS	CHINESE DWARF	NEWT	LIV	100	NO	SG	SG	T	W	C	C	1/4/05	12/19/04	I	NY	INTERNATIONAL PET RESOURCES LLC
2005520606	CYPY	CYNOPS	PYRRHOGASTER	JAPANESE FIREBELLY	NEWT	LIV	480	NO	HK	HK	T	W	C	C	12/13/04	12/11/04	I	NY	TRANSSHIP DISCOUNTS , LTD.
2005520606	PACB	PACHYTRITON	BREVIPES	TSITOU	NEWT	LIV	240	NO	HK	HK	T	W	C	C	12/13/04	12/11/04	I	NY	TRANSSHIP DISCOUNTS , LTD.
2005520609	CYPY	CYNOPS	PYRRHOGASTER	JAPANESE FIREBELLY	NEWT	LIV	375	NO	HK	HK	T	W	C	C	12/13/04	12/11/04	I	NY	ERDA INCORPORATED
2005520639	CYPY	CYNOPS	PYRRHOGASTER	JAPANESE FIREBELLY	NEWT	LIV	50	NO	HK	HK	T	W	C	C	12/27/04	12/25/04	I	NY	ERDA INCORPORATED
2005520829	CYPY	CYNOPS	PYRRHOGASTER	JAPANESE FIREBELLY	NEWT	LIV	725	NO	HK	HK	T	W	C	C	1/10/05	1/8/05	I	NY	ERDA INCORPORATED
2005520948	CYPY	CYNOPS	PYRRHOGASTER	JAPANESE FIREBELLY	NEWT	LIV	290	NO	HK	HK	T	W	C	C	1/5/05	1/8/05	I	NY	TRANSSHIP DISCOUNTS , LTD.
2005520948	PACB	PACHYTRITON	BREVIPES	TSITOU	NEWT	LIV	30	NO	HK	HK	T	W	C	C	1/5/05	1/8/05	I	NY	TRANSSHIP DISCOUNTS , LTD.
2005521010	CYPY	CYNOPS	PYRRHOGASTER	JAPANESE FIREBELLY	NEWT	LIV	200	NO	HK	HK	T	W	C	C	12/27/04	12/25/04	I	NY	TRANSSHIP DISCOUNTS , LTD.
2005522543	CNP?	CYNOPS	SPECIES	FIREBELLY	NEWT	LIV	250	NO	HK	HK	T	C	C	C	3/3/05	3/3/05	I	DF	Golden Sea International
2005523539	TRIT	TRITURUS	SPECIES		NEWT	LIV	30	NO	HK	HK	T	C	C	C	3/5/05	3/6/05	I	LA	NAME REMOVED
2005523546	CYOR	CYNOPS	ORIENTALIS	CHINESE DWARF	NEWT	LIV	150	NO	HK	HK	T	C	C	C	3/6/05	3/6/05	I	LA	DOLPHIN INTERNATIONAL
2005523546	PACB	PACHYTRITON	BREVIPES	TSITOU	NEWT	LIV	200	NO	HK	HK	T	C	C	C	3/6/05	3/6/05	I	LA	DOLPHIN INTERNATIONAL
2005528422	CYOR	CYNOPS	ORIENTALIS	CHINESE DWARF	NEWT	LIV	6,000	NO	CN	CN	T	C	C	C	3/12/05	3/12/05	I	LA	Z IMPORTS INC.
2005528428	TRIT	TRITURUS	SPECIES		NEWT	LIV	3,000	NO	HK	HK	T	C	C	C	3/12/05	3/12/05	I	LA	GLOBAL AQUATIC CONSULTING
2005528432	CYOR	CYNOPS	ORIENTALIS	CHINESE DWARF	NEWT	LIV	1,000	NO	HK	HK	T	C	C	C	3/12/05	3/12/05	I	LA	DOLPHIN INTERNATIONAL

2005528513	CYOR	CYNOPS	ORIENTALIS	CHINESE DWARF	NEWT	LIV	2,950	NO	HK	HK	T	C	C	C	3/12/05	3/13/05	I	LA	DOLPHIN INTERNATIONAL
2005528530	TRIT	TRITURUS	SPECIES		NEWT	LIV	600	NO	HK	HK	T	C	C	C	3/13/05	3/13/05	I	LA	AQUATIC CONNECTION
2005528545	TRIH	TRITURUS	HONGKONGENSIS	ALPINE	NEWT	LIV	200	NO	HK	HK	T	W	C	C	3/13/05	3/13/05	I	LA	AQUACO, INC.
2005531886	CYOR	CYNOPS	ORIENTALIS	CHINESE DWARF	NEWT	LIV	6,500	NO	CN	CN	T	C	C	C	3/19/05	3/19/05	I	LA	Z IMPORTS INC.
2005531887	TRIT	TRITURUS	SPECIES		NEWT	LIV	1,400	NO	HK	HK	T	C	C	C	3/19/05	3/19/05	I	LA	GLOBAL AQUATIC CONSULTING
2005531945	TRIH	TRITURUS	HONGKONGENSIS	ALPINE	NEWT	LIV	850	NO	VS	HK	T	W	C	C	3/20/05	3/20/05	I	LA	AQUACO, INC.
2005531954	TRIT	TRITURUS	SPECIES		NEWT	LIV	30	NO	HK	HK	T	C	C	C	3/20/05	3/20/05	I	LA	NAME REMOVED
2005531984	CYOR	CYNOPS	ORIENTALIS	CHINESE DWARF	NEWT	LIV	1,200	NO	HK	HK	T	C	C	C	3/20/05	3/20/05	I	LA	DOLPHIN INTERNATIONAL
2005532565	CYPY	CYNOPS	PYRRHOGASTER	JAPANESE FIREBELLY	NEWT	LIV	60	NO	HK	HK	T	W	C	C	2/23/05	2/19/05	I	NY	TRANSSHIP DISCOUNTS , LTD.
2005532579	CYPY	CYNOPS	PYRRHOGASTER	JAPANESE FIREBELLY	NEWT	LIV	20	NO	HK	HK	T	W	C	C	2/1/05	1/29/05	I	NY	TRANSSHIP DISCOUNTS , LTD.
2005532625	CYPY	CYNOPS	PYRRHOGASTER	JAPANESE FIREBELLY	NEWT	LIV	600	NO	HK	HK	T	W	C	C	12/20/04	12/18/04	I	NY	ERDA INCORPORATED
2005532785	CYOR	CYNOPS	ORIENTALIS	CHINESE DWARF	NEWT	LIV	200	NO	CN	CN	T	F	C	C	2/28/05	2/17/05	I	NY	MIN HUA ENTERPRISE, INC.
2005533070	CYPY	CYNOPS	PYRRHOGASTER	JAPANESE FIREBELLY	NEWT	LIV	120	NO	HK	HK	T	W	C	C	3/2/05	2/26/05	I	NY	ERDA INCORPORATED
2005533293	CYOR	CYNOPS	ORIENTALIS	CHINESE DWARF	NEWT	LIV	200	NO	CN	CN	T	F	C	C	1/13/05	1/5/05	I	NY	MIN HUA ENTERPRISE, INC.
2005533462	CYOR	CYNOPS	ORIENTALIS	CHINESE DWARF	NEWT	LIV	40	NO	SG	SG	T	W	C	C	2/7/05	1/23/05	I	NY	INTERNATIONAL PET RESOURCES LLC
2005533564	TRIH	TRITURUS	HONGKONGENSIS	ALPINE	NEWT	LIV	360	NO	HK	HK	T	W	C	C	2/7/05	1/23/05	I	NY	INTERNATIONAL PET RESOURCES LLC
2005535577	CYOR	CYNOPS	ORIENTALIS	CHINESE DWARF	NEWT	LIV	4,700	NO	CN	CN	T	C	C	C	3/26/05	3/19/05	I	LA	Z IMPORTS INC.
2005535592	CYOR	CYNOPS	ORIENTALIS	CHINESE DWARF	NEWT	LIV	750	NO	HK	HK	T	C	C	C	3/26/05	3/26/05	I	LA	DOLPHIN INTERNATIONAL
2005535630	CYOR	CYNOPS	ORIENTALIS	CHINESE DWARF	NEWT	LIV	3,450	NO	HK	HK	T	C	C	C	3/26/05	3/27/05	I	LA	DOLPHIN INTERNATIONAL
2005535639	TRIH	TRITURUS	HONGKONGENSIS	ALPINE	NEWT	LIV	600	NO	VS	HK	T	W	C	C	3/26/05	3/27/05	I	LA	AQUACO, INC.
2005535667	TRIT	TRITURUS	SPECIES		NEWT	LIV	400	NO	HK	HK	T	C	C	C	3/27/05	3/27/05	I	LA	GLOBAL AQUATIC CONSULTING
2005538666	CYOR	CYNOPS	ORIENTALIS	CHINESE DWARF	NEWT	LIV	250	NO	HK	HK	T	C	C	C	4/2/05	4/2/05	I	LA	DOLPHIN INTERNATIONAL
2005538674	TRIT	TRITURUS	SPECIES		NEWT	LIV	800	NO	HK	HK	T	C	C	C	4/2/05	4/2/05	I	LA	GLOBAL AQUATIC CONSULTING
2005538738	CYOR	CYNOPS	ORIENTALIS	CHINESE DWARF	NEWT	LIV	2,800	NO	HK	HK	T	C	C	C	4/2/05	4/3/05	I	LA	DOLPHIN INTERNATIONAL
2005538744	TRIH	TRITURUS	HONGKONGENSIS	ALPINE	NEWT	LIV	418	NO	VS	HK	T	W	C	C	4/2/05	4/3/05	I	LA	AQUACO, INC.
2005540640	CYOR	CYNOPS	ORIENTALIS	CHINESE DWARF	NEWT	LIV	250	NO	HK	HK	T	C	C	C	4/9/05	4/9/05	I	LA	DOLPHIN INTERNATIONAL
2005540649	CYOR	CYNOPS	ORIENTALIS	CHINESE DWARF	NEWT	LIV	3,700	NO	CN	CN	T	C	C	C	4/9/05	4/9/05	I	LA	Z IMPORTS INC.
2005540658	TRIT	TRITURUS	SPECIES		NEWT	LIV	600	NO	HK	HK	T	C	C	C	4/9/05	4/9/05	I	LA	GLOBAL AQUATIC CONSULTING
2005540785	TRIH	TRITURUS	HONGKONGENSIS	ALPINE	NEWT	LIV	400	NO	VS	HK	T	W	C	C	4/10/05	4/10/05	I	LA	AQUACO, INC.
2005540788	CYOR	CYNOPS	ORIENTALIS	CHINESE DWARF	NEWT	LIV	2,100	NO	HK	HK	T	C	C	C	4/10/05	4/10/05	I	LA	DOLPHIN INTERNATIONAL
2005540792	TRIT	TRITURUS	SPECIES		NEWT	LIV	200	NO	VS	HK	T	C	C	C	4/10/05	4/10/05	I	LA	AQUATIC CONNECTION
2005544642	BODO	BOLITOGLOSSA	DOFLEINI	ALTA VERAPAZ	SALAMANDER	LIV	2	NO	HN	HN	T	W	C	C	1/25/05	1/25/05	I	MI	WORLDWIDE HERP IMPORT & EXPORT
2005544976	SAL?	SALAMANDRA	SPECIES	FIRE	SALAMANDER	LIV	10	NO	DE	DE	T	C	C	C	3/17/05	3/16/05	I	MI	GLADES HERP, INC.
2005545285	NEW?	PACHYTRITON	SPECIES	CHINESE	NEWT	LIV	200	NO	CN	CN	T	C	C	C	2/4/05	2/3/05	I	MI	JP PETS
2005545285	CYOR	CYNOPS	ORIENTALIS	CHINESE DWARF	NEWT	LIV	1,500	NO	CN	CN	T	C	C	C	2/4/05	2/3/05	I	MI	JP PETS
2005545655	CYOR	CYNOPS	ORIENTALIS	CHINESE DWARF	NEWT	LIV	1,700	NO	CN	CN	T	C	C	C	4/16/05	4/16/05	I	LA	Z IMPORTS INC.
2005545657	TRIT	TRITURUS	SPECIES		NEWT	LIV	1,200	NO	HK	HK	T	C	C	C	4/16/05	4/16/05	I	LA	GLOBAL AQUATIC CONSULTING
2005545658	CYOR	CYNOPS	ORIENTALIS	CHINESE DWARF	NEWT	LIV	500	NO	HK	HK	T	C	C	C	4/16/05	4/16/05	I	LA	DOLPHIN INTERNATIONAL
2005545728	CYOR	CYNOPS	ORIENTALIS	CHINESE DWARF	NEWT	LIV	3,450	NO	HK	HK	T	C	C	C	4/16/05	4/17/05	I	LA	DOLPHIN INTERNATIONAL
2005545728	NEW?	PACHYTRITON	SPECIES	CHINESE	NEWT	LIV	100	NO	HK	HK	T	C	C	C	4/16/05	4/17/05	I	LA	DOLPHIN INTERNATIONAL
2005545791	TRIT	TRITURUS	SPECIES		NEWT	LIV	90	NO	HK	HK	T	C	C	C	4/17/05	4/17/05	I	LA	NAME REMOVED
2005546560	BODO	BOLITOGLOSSA	DOFLEINI	ALTA VERAPAZ	SALAMANDER	LIV	33	NO	HN	HN	T	W	C	C	5/25/04	5/25/04	I	MI	STRICTLY REPTILES, INC.
2005548647	ODP?	OEDIPINA	SPECIES	WORM	SALAMANDER	LIV	1	NO	CR	CR	S	W	C	C	4/21/05	2/24/05	I	AT	Atlanta Botanical Garden
2005550160	CYOR	CYNOPS	ORIENTALIS	CHINESE DWARF	NEWT	LIV	250	NO	HK	HK	T	C	C	C	4/23/05	4/23/05	I	LA	DOLPHIN INTERNATIONAL
2005550170	TRIT	TRITURUS	SPECIES		NEWT	LIV	2,200	NO	HK	HK	T	C	C	C	4/23/05	4/23/05	I	LA	GLOBAL AQUATIC CONSULTING
2005550178	CYOR	CYNOPS	ORIENTALIS	CHINESE DWARF	NEWT	LIV	3,000	NO	CN	CN	T	C	C	C	4/23/05	4/23/05	I	LA	Z IMPORTS INC.
2005550236	CYOR	CYNOPS	ORIENTALIS	CHINESE DWARF	NEWT	LIV	2,800	NO	HK	HK	T	C	C	C	4/24/05	4/24/05	I	LA	DOLPHIN INTERNATIONAL
2005550236	PACB	PACHYTRITON	BREVIPES	TSITOU	NEWT	LIV	300	NO	HK	HK	T	C	C	C	4/24/05	4/24/05	I	LA	DOLPHIN INTERNATIONAL
2005550248	TRIH	TRITURUS	HONGKONGENSIS	ALPINE	NEWT	LIV	200	NO	HK	HK	T	W	C	C	4/24/05	4/24/05	I	LA	AQUACO, INC.
2005550289	TRIT	TRITURUS	SPECIES		NEWT	LIV	400	NO	HK	HK	T	C	C	C	4/24/05	4/24/05	I	LA	AQUATIC CONNECTION
2005550981	CYPY	CYNOPS	PYRRHOGASTER	JAPANESE FIREBELLY	NEWT	LIV	2	NO	CA	CA	P	C	C	C	7/11/04	7/11/04	I	PL	Exemptions 6 and 7(C)
2005551996	PLAB	PACHYTRITON	LABIATUS	UNTERSTEIN'S	NEWT	LIV	1,640	NO	CN	CN	T	W	C	C	4/26/05	5/25/05	I	SE	Exemptions 6 and 7(C)
2005551996	PACB	PACHYTRITON	BREVIPES	TSITOU	NEWT	LIV	100	NO	CN	CN	T	W	C	C	4/26/05	5/25/05	I	SE	Exemptions 6 and 7(C)
2005551996	NEW?	PACHYTRITON	SPECIES	CHINESE	NEWT	LIV	100	NO	CN	CN	T	W	C	C	4/26/05	5/25/05	I	SE	Exemptions 6 and 7(C)
2005551996	NEW?	PACHYTRITON	SPECIES	CHINESE	NEWT	LIV	27	NO	CN	CN	T	W	C	C	4/26/05	5/25/05	I	SE	Exemptions 6 and 7(C)
2005551996	CNP?	CYNOPS	SPECIES	FIREBELLY	NEWT	LIV	300	NO	CN	CN	T	W	C	C	4/26/05	5/25/05	I	SE	Exemptions 6 and 7(C)
2005551996	CYOR	CYNOPS	ORIENTALIS	CHINESE DWARF	NEWT	LIV	1,000	NO	CN	CN	T	W	C	C	4/26/05	5/25/05	I	SE	Exemptions 6 and 7(C)
2005552305	TRIT	TRITURUS	SPECIES		NEWT	LIV	100	NO	DE	DE	T	C	C	C	4/26/05	4/26/05	I	DF	U.S. GLOBAL EXOTICS, INC.
2005552305	SAL?	SALAMANDRA	SPECIES	FIRE	SALAMANDER	LIV	50	NO	DE	DE	T	C	C	C	4/26/05	4/26/05	I	DF	U.S. GLOBAL EXOTICS, INC.
2005552715	SASA	SALAMANDRA	SALAMANDRA	EUROPEAN FIRE	SALAMANDER	LIV	200	NO	RU	RU	T	W	C	C	4/27/05	4/27/05	I	LA	L. A. REPTILE

2005553542	TRIT	TRITURUS	SPECIES		NEWT	LIV	600	NO	HK	HK	T	C	C	C	4/30/05	4/30/05	I	LA	GLOBAL AQUATIC CONSULTING
2005553544	CYOR	CYNOPS	ORIENTALIS	CHINESE DWARF	NEWT	LIV	2,000	NO	CN	CN	T	W	C	C	4/30/05	4/23/05	I	LA	Z IMPORTS INC.
2005553590	CYOR	CYNOPS	ORIENTALIS	CHINESE DWARF	NEWT	LIV	950	NO	HK	HK	T	C	C	C	4/30/05	5/1/05	I	LA	DOLPHIN INTERNATIONAL
2005553590	NEW?	PACHYTRITON	SPECIES	CHINESE	NEWT	LIV	200	NO	HK	HK	T	C	C	C	4/30/05	5/1/05	I	LA	DOLPHIN INTERNATIONAL
2005553597	TRIT	TRITURUS	SPECIES		NEWT	LIV	30	NO	HK	HK	T	C	C	C	5/1/05	5/1/05	I	LA	NAME REMOVED
2005553613	TRIH	TRITURUS	HONGKONGENSIS	ALPINE	NEWT	LIV	350	NO	HK	HK	T	W	C	C	5/1/05	5/1/05	I	LA	AQUACO, INC.
2005554653	SASA	SALAMANDRA	SALAMANDRA	EUROPEAN FIRE	SALAMANDER	LIV	300	NO	RU	RU	T	W	C	C	5/4/05	5/4/05	I	LA	STRICTLY REPTILES, INC.
2005555407	TRIT	TRITURUS	SPECIES		NEWT	LIV	100	NO	SG	SG	T	W	C	C	3/17/05	3/17/05	I	CH	INTERCONTINENTAL IMPORTS, INC.
2005555934	CYOR	CYNOPS	ORIENTALIS	CHINESE DWARF	NEWT	LIV	500	NO	HK	HK	T	C	C	C	5/7/05	5/7/05	I	LA	DOLPHIN INTERNATIONAL
2005555935	CYOR	CYNOPS	ORIENTALIS	CHINESE DWARF	NEWT	LIV	1,700	NO	CN	CN	T	W	C	C	5/7/05	5/7/05	I	LA	Z IMPORTS INC.
2005555942	TRIT	TRITURUS	SPECIES		NEWT	LIV	600	NO	HK	HK	T	C	C	C	5/7/05	5/7/05	I	LA	GLOBAL AQUATIC CONSULTING
2005555979	CYOR	CYNOPS	ORIENTALIS	CHINESE DWARF	NEWT	LIV	2,200	NO	HK	HK	T	C	C	C	5/8/05	5/8/05	I	LA	DOLPHIN INTERNATIONAL
2005555979	PACB	PACHYTRITON	BREVIPES	TSITOU	NEWT	LIV	200	NO	HK	HK	T	C	C	C	5/8/05	5/8/05	I	LA	DOLPHIN INTERNATIONAL
2005555990	TRIH	TRITURUS	HONGKONGENSIS	ALPINE	NEWT	LIV	150	NO	HK	HK	T	W	C	C	5/8/05	5/8/05	I	LA	AQUACO, INC.
2005556151	TRIT	TRITURUS	SPECIES		NEWT	LIV	50	NO	SG	SG	T	W	C	C	4/7/05	4/7/05	I	CH	INTERCONTINENTAL IMPORTS, INC.
2005557423	CNP?	CYNOPS	SPECIES	FIREBELLY	NEWT	LIV	540	NO	HK	HK	T	C	C	C	5/10/05	5/10/05	I	DF	Golden Sea International
2005558598	CYPY	CYNOPS	PYRRHOGASTER	JAPANESE FIREBELLY	NEWT	LIV	100	NO	CN	CN	T	W	C	C	3/18/05	3/13/05	I	NY	FISH MART INCORPORATED
2005558598	CNP?	CYNOPS	SPECIES	FIREBELLY	NEWT	LIV	50	NO	CN	CN	T	W	C	C	3/18/05	3/13/05	I	NY	FISH MART INCORPORATED
2005558787	CYPY	CYNOPS	PYRRHOGASTER	JAPANESE FIREBELLY	NEWT	LIV	50	NO	HK	HK	T	W	C	C	4/4/05	4/3/05	I	NY	ERDA INCORPORATED
2005559699	CYOR	CYNOPS	ORIENTALIS	CHINESE DWARF	NEWT	LIV	250	NO	HK	HK	T	C	C	C	5/14/05	5/14/05	I	LA	DOLPHIN INTERNATIONAL
2005559711	CYOR	CYNOPS	ORIENTALIS	CHINESE DWARF	NEWT	LIV	4,000	NO	CN	CN	T	C	C	C	5/14/05	5/14/05	I	LA	Z IMPORTS INC.
2005559714	TRIT	TRITURUS	SPECIES		NEWT	LIV	600	NO	HK	HK	T	C	C	C	5/14/05	5/14/05	I	LA	GLOBAL AQUATIC CONSULTING
2005559782	CYOR	CYNOPS	ORIENTALIS	CHINESE DWARF	NEWT	LIV	1,950	NO	HK	HK	T	C	C	C	5/15/05	5/15/05	I	LA	DOLPHIN INTERNATIONAL
2005559782	PACB	PACHYTRITON	BREVIPES	TSITOU	NEWT	LIV	300	NO	HK	HK	T	C	C	C	5/15/05	5/15/05	I	LA	DOLPHIN INTERNATIONAL
2005559787	TRIH	TRITURUS	HONGKONGENSIS	ALPINE	NEWT	LIV	675	NO	HK	HK	T	W	C	C	5/15/05	5/15/05	I	LA	AQUACO, INC.
2005560704	BODO	BOLITOGLOSSA	DOFLEINI	ALTA VERAPAZ	SALAMANDER	LIV	5	NO	HN	HN	T	W	C	C	5/18/05	5/18/05	I	LA	LA REPTILES INC
2005561805	CYOR	CYNOPS	ORIENTALIS	CHINESE DWARF	NEWT	LIV	3,000	NO	CN	CN	T	C	C	C	5/21/05	5/21/05	I	LA	Z IMPORTS INC.
2005561869	CYOR	CYNOPS	ORIENTALIS	CHINESE DWARF	NEWT	LIV	1,500	NO	HK	HK	T	C	C	C	5/22/05	5/22/05	I	LA	DOLPHIN INTERNATIONAL
2005561869	PACB	PACHYTRITON	BREVIPES	TSITOU	NEWT	LIV	400	NO	HK	HK	T	C	C	C	5/22/05	5/22/05	I	LA	DOLPHIN INTERNATIONAL
2005561872	TRIH	TRITURUS	HONGKONGENSIS	ALPINE	NEWT	LIV	320	NO	VS	HK	T	W	C	C	5/22/05	5/22/05	I	LA	AQUACO, INC.
2005563651	CYOR	CYNOPS	ORIENTALIS	CHINESE DWARF	NEWT	LIV	900	NO	CN	CN	T	W	C	C	5/27/05	5/27/05	I	SE	Exemptions 6 and 7(C)
2005563651	NEW?	PACHYTRITON	SPECIES	CHINESE	NEWT	LIV	8	NO	CN	CN	T	W	C	C	5/27/05	5/27/05	I	SE	Exemptions 6 and 7(C)
2005563651	NEW?	PACHYTRITON	SPECIES	CHINESE	NEWT	LIV	4	NO	CN	CN	T	W	C	C	5/27/05	5/27/05	I	SE	Exemptions 6 and 7(C)
2005563867	CYOR	CYNOPS	ORIENTALIS	CHINESE DWARF	NEWT	LIV	500	NO	HK	HK	T	C	C	C	5/28/05	5/28/05	I	LA	DOLPHIN INTERNATIONAL
2005563871	CYOR	CYNOPS	ORIENTALIS	CHINESE DWARF	NEWT	LIV	1,000	NO	CN	CN	T	C	C	C	5/28/05	5/28/05	I	LA	Z IMPORTS INC.
2005563873	TRIT	TRITURUS	SPECIES		NEWT	LIV	200	NO	HK	HK	T	C	C	C	5/28/05	5/28/05	I	LA	GLOBAL AQUATIC CONSULTING
2005563911	CYOR	CYNOPS	ORIENTALIS	CHINESE DWARF	NEWT	LIV	1,000	NO	HK	HK	T	C	C	C	5/29/05	5/29/05	I	LA	DOLPHIN INTERNATIONAL
2005563911	PACB	PACHYTRITON	BREVIPES	TSITOU	NEWT	LIV	100	NO	HK	HK	T	C	C	C	5/29/05	5/29/05	I	LA	DOLPHIN INTERNATIONAL
2005563935	TRIH	TRITURUS	HONGKONGENSIS	ALPINE	NEWT	LIV	300	NO	HK	HK	T	W	C	C	5/29/05	5/29/05	I	LA	AQUACO, INC.
2005565468	PACB	PACHYTRITON	BREVIPES	TSITOU	NEWT	LIV	100	NO	CN	CN	T	W	C	C	6/3/05	6/3/05	I	SE	Exemptions 6 and 7(C)
2005565786	TRIT	TRITURUS	SPECIES		NEWT	LIV	1,200	NO	HK	HK	T	C	C	C	6/4/05	6/4/05	I	LA	GLOBAL AQUATIC CONSULTING
2005565798	CYOR	CYNOPS	ORIENTALIS	CHINESE DWARF	NEWT	LIV	750	NO	HK	HK	T	C	C	C	6/4/05	6/4/05	I	LA	DOLPHIN INTERNATIONAL
2005565896	TRIH	TRITURUS	HONGKONGENSIS	ALPINE	NEWT	LIV	200	NO	HK	HK	T	W	C	C	6/5/05	6/5/05	I	LA	AQUACO, INC.
2005565898	CYOR	CYNOPS	ORIENTALIS	CHINESE DWARF	NEWT	LIV	1,250	NO	HK	HK	T	C	C	C	6/5/05	6/5/05	I	LA	DOLPHIN INTERNATIONAL
2005565898	PACB	PACHYTRITON	BREVIPES	TSITOU	NEWT	LIV	200	NO	HK	HK	T	C	C	C	6/5/05	6/5/05	I	LA	DOLPHIN INTERNATIONAL
2005567330	CYPY	CYNOPS	PYRRHOGASTER	JAPANESE FIREBELLY	NEWT	LIV	176	NO	HK	HK	T	W	C	C	4/11/05	4/9/05	I	NY	ERDA INCORPORATED
2005567342	CYOR	CYNOPS	ORIENTALIS	CHINESE DWARF	NEWT	LIV	100	NO	SG	SG	T	W	C	C	4/11/05	3/27/05	I	NY	INTERNATIONAL PET RESOURCES , LLC
2005567348	CYPY	CYNOPS	PYRRHOGASTER	JAPANESE FIREBELLY	NEWT	LIV	250	NO	HK	HK	T	W	C	C	4/11/05	4/9/05	I	NY	TRANSSHIP DISCOUNTS , LTD.
2005567770	CYPY	CYNOPS	PYRRHOGASTER	JAPANESE FIREBELLY	NEWT	LIV	150	NO	HK	HK	T	W	C	C	1/19/05	1/15/05	I	NY	TRANSSHIP DISCOUNTS , LTD.
2005567770	PACB	PACHYTRITON	BREVIPES	TSITOU	NEWT	LIV	300	NO	HK	HK	T	W	C	C	1/19/05	1/15/05	I	NY	TRANSSHIP DISCOUNTS , LTD.
2005567809	CYPY	CYNOPS	PYRRHOGASTER	JAPANESE FIREBELLY	NEWT	LIV	200	NO	HK	HK	T	W	C	C	1/19/05	1/15/05	I	NY	ERDA INCORPORATED
2005567863	CYPY	CYNOPS	PYRRHOGASTER	JAPANESE FIREBELLY	NEWT	LIV	300	NO	HK	HK	T	W	C	C	3/16/05	3/12/05	I	NY	TRANSSHIP DISCOUNTS , LTD.
2005568002	CYOR	CYNOPS	ORIENTALIS	CHINESE DWARF	NEWT	LIV	200	NO	CN	CN	T	F	C	C	3/11/05	3/2/05	I	NY	MIN HUA ENTERPRISE, INC.
2005568009	CYPY	CYNOPS	PYRRHOGASTER	JAPANESE FIREBELLY	NEWT	LIV	450	NO	HK	HK	T	W	C	C	4/18/05	4/16/05	I	NY	ERDA INCORPORATED
2005568127	CYPY	CYNOPS	PYRRHOGASTER	JAPANESE FIREBELLY	NEWT	LIV	100	NO	CN	CN	T	W	C	C	2/3/05	1/30/05	I	NY	FISH MART INCORPORATED
2005568127	CNP?	CYNOPS	SPECIES	FIREBELLY	NEWT	LIV	50	NO	CN	CN	T	W	C	C	2/3/05	1/30/05	I	NY	FISH MART INCORPORATED
2005568331	CYPY	CYNOPS	PYRRHOGASTER	JAPANESE FIREBELLY	NEWT	LIV	600	NO	HK	HK	T	C	C	C	3/17/05	3/12/05	I	NY	ERDA INCORPORATED
2005568484	CYOR	CYNOPS	ORIENTALIS	CHINESE DWARF	NEWT	LIV	4,000	NO	HK	CN	T	W	C	C	6/11/05	6/11/05	I	LA	Z IMPORTS INC.
2005568492	PACB	PACHYTRITON	BREVIPES	TSITOU	NEWT	LIV	100	NO	HK	HK	T	C	C	C	6/11/05	6/11/05	I	LA	DOLPHIN INTERNATIONAL
2005568567	CYOR	CYNOPS	ORIENTALIS	CHINESE DWARF	NEWT	LIV	2,200	NO	HK	HK	T	C	C	C	6/12/05	6/12/05	I	LA	DOLPHIN INTERNATIONAL

2005568836	CYPY	CYNOPS	PYRRHOGASTER	JAPANESE FIREBELLY	NEWT	LIV	20	NO	HK	HK	T	W	C	C	3/1/05	2/26/05	I	NY	TRANSSHIP DISCOUNTS , LTD.
2005568862	CYPY	CYNOPS	PYRRHOGASTER	JAPANESE FIREBELLY	NEWT	LIV	175	NO	HK	HK	T	W	C	C	2/25/05	2/19/05	I	NY	ERDA INCORPORATED
2005569180	CYPY	CYNOPS	PYRRHOGASTER	JAPANESE FIREBELLY	NEWT	LIV	150	NO	HK	HK	T	W	C	C	4/4/05	4/3/05	I	NY	TRANSSHIP DISCOUNTS , LTD.
2005569269	CYOR	CYNOPS	ORIENTALIS	CHINESE DWARF	NEWT	LIV	100	NO	SG	SG	T	W	C	C	3/23/05	3/5/05	I	NY	INTERNATIONAL PET RESOURCES , LLC
2005569298	CYOR	CYNOPS	ORIENTALIS	CHINESE DWARF	NEWT	LIV	200	NO	CN	CN	T	F	C	C	3/18/05	3/9/05	I	NY	MIN HUA ENTERPRISE, INC.
2005569382	CYPY	CYNOPS	PYRRHOGASTER	JAPANESE FIREBELLY	NEWT	LIV	1,200	NO	HK	HK	T	W	C	C	3/23/05	3/19/05	I	NY	ERDA INCORPORATED
2005569789	CYPY	CYNOPS	PYRRHOGASTER	JAPANESE FIREBELLY	NEWT	LIV	100	NO	CN	CN	T	W	C	C	2/28/05	2/20/05	I	NY	FISH MART INCORPORATED
2005569789	CNP?	CYNOPS	SPECIES	FIREBELLY	NEWT	LIV	50	NO	CN	CN	T	W	C	C	2/28/05	2/20/05	I	NY	FISH MART INCORPORATED
2005569990	CYPY	CYNOPS	PYRRHOGASTER	JAPANESE FIREBELLY	NEWT	LIV	100	NO	CN	CN	T	W	C	C	3/14/05	3/6/05	I	NY	FISH MART INCORPORATED
2005569990	CNP?	CYNOPS	SPECIES	FIREBELLY	NEWT	LIV	75	NO	CN	CN	T	W	C	C	3/14/05	3/6/05	I	NY	FISH MART INCORPORATED
2005570130	CYPY	CYNOPS	PYRRHOGASTER	JAPANESE FIREBELLY	NEWT	LIV	200	NO	HK	HK	T	W	C	C	4/19/05	4/16/05	I	NY	TRANSSHIP DISCOUNTS , LTD.
2005572135	AMBM	AMBYSTOMA	MEXICANUM		AXOLOTL	LIV	1	NO	IE	IE	P	C	C	C	5/24/05	5/24/05	I	CH	NAME REMOVED
2005572196	TYKW	TYLOTOTRITON	KWEICHOWENSIS	KWEICHOW CROCODILE	NEWT	LIV	1,000	NO	CN	CN	T	W	C	C	6/17/05	6/17/05	I	SE	Exemptions 6 and 7(C)
2005572196	CNP?	CYNOPS	SPECIES	FIREBELLY	NEWT	LIV	224	NO	CN	CN	T	W	C	C	6/17/05	6/17/05	I	SE	Exemptions 6 and 7(C)
2005572389	AMBA	AMBYSTOMA	MACULATUM	SPOTTED	SALAMANDER	LIV	10	NO	US	ES	T	W	C	C	4/26/05	4/26/05	I	SF	WONDERFUL WORLD PET SUPPLIERS
2005572669	CYOR	CYNOPS	ORIENTALIS	CHINESE DWARF	NEWT	LIV	500	NO	HK	HK	T	C	C	C	6/18/05	6/18/05	I	LA	DOLPHIN INTERNATIONAL
2005572669	PACB	PACHYTRITON	BREVIPES	TSITOU	NEWT	LIV	100	NO	HK	HK	T	C	C	C	6/18/05	6/18/05	I	LA	DOLPHIN INTERNATIONAL
2005572689	CYOR	CYNOPS	ORIENTALIS	CHINESE DWARF	NEWT	LIV	3,000	NO	CN	CN	T	C	C	C	6/18/05	6/18/05	I	LA	Z IMPORTS INC.
2005572695	TRIT	TRITURUS	SPECIES		NEWT	LIV	600	NO	HK	HK	T	C	C	C	6/18/05	6/18/05	I	LA	GLOBAL AQUATIC CONSULTING
2005572805	TRIT	TRITURUS	SPECIES		NEWT	LIV	180	NO	HK	HK	T	C	C	C	6/19/05	6/19/05	I	LA	NAME REMOVED
2005572829	TYVE	TYLOTOTRITON	VERRUCOSUS	CROCODILE	NEWT	LIV	80	NO	SG	SG	T	W	C	C	5/23/04	4/4/04	I	NW	INTERNATIONAL PET RESOURCES , LLC
2005578233	CYOR	CYNOPS	ORIENTALIS	CHINESE DWARF	NEWT	LIV	250	NO	HK	HK	T	C	C	C	6/25/05	6/25/05	I	LA	DOLPHIN INTERNATIONAL
2005578259	TRIT	TRITURUS	SPECIES		NEWT	LIV	1,000	NO	HK	HK	T	C	C	C	6/25/05	6/25/05	I	LA	GLOBAL AQUATIC CONSULTING
2005578337	CYOR	CYNOPS	ORIENTALIS	CHINESE DWARF	NEWT	LIV	1,600	NO	HK	HK	T	C	C	C	6/26/05	6/26/05	I	LA	DOLPHIN INTERNATIONAL
2005578337	PACB	PACHYTRITON	BREVIPES	TSITOU	NEWT	LIV	100	NO	HK	HK	T	C	C	C	6/26/05	6/26/05	I	LA	DOLPHIN INTERNATIONAL
2005578344	TRIH	TRITURUS	HONGKONGENSIS	ALPINE	NEWT	LIV	300	NO	HK	HK	T	W	C	C	6/26/05	6/26/05	I	LA	AQUACO, INC.
2005578827	TYKW	TYLOTOTRITON	KWEICHOWENSIS	KWEICHOW CROCODILE	NEWT	LIV	400	NO	HK	HK	T	W	C	C	6/27/05	6/27/05	I	LA	U.S. GLOBAL EXOTICS, INC.
2005578993	SASA	SALAMANDRA	SALAMANDRA	EUROPEAN FIRE	SALAMANDER	LIV	8	NO	GB	GB	T	C	C	C	9/19/04	9/9/04	I	MI	NEW ENGLAND REPTILE DISTRIBUTORS, INC.
2005579003	BODO	BOLITOGLOSSA	DOFLEINI	ALTA VERAPAZ	SALAMANDER	LIV	50	NO	HN	HN	T	W	C	C	11/25/04	11/22/04	I	MI	STRICTLY REPTILES, INC.
2005580807	CYPY	CYNOPS	PYRRHOGASTER	JAPANESE FIREBELLY	NEWT	LIV	500	NO	HK	HK	T	W	C	C	6/8/05	6/4/05	I	NY	TRANSSHIP DISCOUNTS , LTD.
2005580820	CYPY	CYNOPS	PYRRHOGASTER	JAPANESE FIREBELLY	NEWT	LIV	300	NO	HK	HK	T	C	C	C	6/8/05	6/4/05	I	NY	ERDA INCORPORATED
2005580893	CYOR	CYNOPS	ORIENTALIS	CHINESE DWARF	NEWT	LIV	40	NO	SG	SG	T	W	C	C	5/9/05	4/24/05	I	NY	INTERNATIONAL PET RESOURCES , LLC
2005580910	CYPY	CYNOPS	PYRRHOGASTER	JAPANESE FIREBELLY	NEWT	LIV	450	NO	HK	HK	T	W	C	C	5/9/05	5/7/05	I	NY	ERDA INCORPORATED
2005581137	CYPY	CYNOPS	PYRRHOGASTER	JAPANESE FIREBELLY	NEWT	LIV	150	NO	HK	HK	T	W	C	C	6/1/05	5/28/05	I	NY	ERDA INCORPORATED
2005581838	CYOR	CYNOPS	ORIENTALIS	CHINESE DWARF	NEWT	LIV	200	NO	CN	CN	T	F	C	C	5/25/05	5/16/05	I	NY	MIN HUA ENTERPRISE, INC.
2005582263	CYPY	CYNOPS	PYRRHOGASTER	JAPANESE FIREBELLY	NEWT	LIV	150	NO	HK	HK	T	W	C	C	5/2/05	4/30/05	I	NY	TRANSSHIP DISCOUNTS , LTD.
2005582322	CYPY	CYNOPS	PYRRHOGASTER	JAPANESE FIREBELLY	NEWT	LIV	1,200	NO	CN	CN	T	W	C	C	6/30/05	6/30/05	I	SE	AFRICAN NORTHWEST
2005582460	CNP?	CYNOPS	SPECIES	FIREBELLY	NEWT	LIV	150	NO	HK	HK	T	W	C	C	5/2/05	4/30/05	I	NY	ERDA INCORPORATED
2005582644	CYPY	CYNOPS	PYRRHOGASTER	JAPANESE FIREBELLY	NEWT	LIV	200	NO	HK	HK	T	W	C	C	5/17/05	5/14/05	I	NY	TRANSSHIP DISCOUNTS , LTD.
2005582677	CYPY	CYNOPS	PYRRHOGASTER	JAPANESE FIREBELLY	NEWT	LIV	450	NO	HK	HK	T	C	C	C	5/16/05	5/14/05	I	NY	ERDA INCORPORATED
2005582750	CYOR	CYNOPS	ORIENTALIS	CHINESE DWARF	NEWT	LIV	4,000	NO	CN	CN	T	W	C	C	7/1/05	7/1/05	I	LA	Z IMPORTS INC.
2005582779	TYVE	TYLOTOTRITON	VERRUCOSUS	CROCODILE	NEWT	LIV	16	NO	SG	SG	T	W	C	C	5/18/05	5/1/05	I	NY	INTERNATIONAL PET RESOURCES , LLC
2005582907	PSHG	PACHYHYNOBIUS	SHANGCHENGENSIS	SHANGCHENG	SALAMANDER	LIV	30	NO	CN	CN	T	W	C	C	7/1/05	7/1/05	I	SE	Exemptions 6 and 7(C)
2005582907	CYOR	CYNOPS	ORIENTALIS	CHINESE DWARF	NEWT	LIV	1,000	NO	CN	CN	T	W	C	C	7/1/05	7/1/05	I	SE	Exemptions 6 and 7(C)
2005583074	CYOR	CYNOPS	ORIENTALIS	CHINESE DWARF	NEWT	LIV	60	NO	SG	SG	T	C	C	C	5/12/05	5/12/05	I	NY	Exemptions 6 and 7(C)
2005583162	TRIT	TRITURUS	SPECIES		NEWT	LIV	600	NO	HK	HK	T	C	C	C	7/2/05	7/2/05	I	LA	GLOBAL AQUATIC CONSULTING
2005583183	CYOR	CYNOPS	ORIENTALIS	CHINESE DWARF	NEWT	LIV	750	NO	HK	HK	T	C	C	C	7/2/05	7/2/05	I	LA	DOLPHIN INTERNATIONAL
2005583183	PACB	PACHYTRITON	BREVIPES	TSITOU	NEWT	LIV	200	NO	HK	HK	T	C	C	C	7/2/05	7/2/05	I	LA	DOLPHIN INTERNATIONAL
2005583233	CYOR	CYNOPS	ORIENTALIS	CHINESE DWARF	NEWT	LIV	750	NO	HK	HK	T	C	C	C	7/3/05	7/3/05	I	LA	DOLPHIN INTERNATIONAL
2005583233	PACB	PACHYTRITON	BREVIPES	TSITOU	NEWT	LIV	300	NO	HK	HK	T	C	C	C	7/3/05	7/3/05	I	LA	DOLPHIN INTERNATIONAL
2005583240	TRIT	TRITURUS	SPECIES		NEWT	LIV	400	NO	HK	HK	T	C	C	C	7/3/05	7/3/05	I	LA	AQUATIC CONNECTION
2005583241	TRIT	TRITURUS	SPECIES		NEWT	LIV	500	NO	HK	HK	T	C	C	C	7/3/05	7/3/05	I	LA	NAME REMOVED
2005583250	TRIH	TRITURUS	HONGKONGENSIS	ALPINE	NEWT	LIV	400	NO	HK	HK	T	W	C	C	7/3/05	7/3/05	I	LA	AQUACO, INC.
2005583277	SASA	SALAMANDRA	SALAMANDRA	EUROPEAN FIRE	SALAMANDER	LIV	16	NO	VN	VN	T	W	C	C	7/4/05	7/4/05	I	LA	L. A. REPTILE
2005583973	CYPY	CYNOPS	PYRRHOGASTER	JAPANESE FIREBELLY	NEWT	LIV	350	NO	HK	HK	T	W	C	C	5/9/05	5/7/05	I	NY	TRANSSHIP DISCOUNTS , LTD.
2005583973	NEW?	PACHYTRITON	SPECIES	CHINESE	NEWT	LIV	60	NO	HK	HK	T	W	C	C	5/9/05	5/7/05	I	NY	TRANSSHIP DISCOUNTS , LTD.
2005584608	CYOR	CYNOPS	ORIENTALIS	CHINESE DWARF	NEWT	LIV	100	NO	SG	SG	T	W	C	C	5/3/05	4/17/05	I	NY	INTERNATIONAL PET RESOURCES , LLC
2005585027	CYPY	CYNOPS	PYRRHOGASTER	JAPANESE FIREBELLY	NEWT	LIV	700	NO	HK	HK	T	W	C	C	4/25/05	4/23/05	I	NY	TRANSSHIP DISCOUNTS , LTD.
2005585027	PACB	PACHYTRITON	BREVIPES	TSITOU	NEWT	LIV	60	NO	HK	HK	T	W	C	C	4/25/05	4/23/05	I	NY	TRANSSHIP DISCOUNTS , LTD.

2005585107	TRIH	TRITURUS	HONGKONGENSIS	ALPINE	NEWT	LIV	400	NO	HK	HK	T	W	C	C	4/26/05	4/10/05	I	NY	INTERNATIONAL PET RESOURCES , LLC
2005587542	TRIH	TRITURUS	HONGKONGENSIS	ALPINE	NEWT	LIV	100	NO	HK	HK	T	W	C	C	7/11/05	7/10/05	I	LA	AQUACO, INC.
2005587630	CYOR	CYNOPS	ORIENTALIS	CHINESE DWARF	NEWT	LIV	2,100	NO	HK	HK	T	C	C	C	7/11/05	7/10/05	I	LA	DOLPHIN INTERNATIONAL
2005587630	PACB	PACHYTRITON	BREVIPES	TSITOU	NEWT	LIV	300	NO	HK	HK	T	C	C	C	7/11/05	7/10/05	I	LA	DOLPHIN INTERNATIONAL
2005587630	TYVE	TYLOTOTRITON	VERRUCOSUS	CROCODILE	NEWT	LIV	300	NO	HK	HK	T	C	C	C	7/11/05	7/10/05	I	LA	DOLPHIN INTERNATIONAL
2005588341	TRIT	TRITURUS	SPECIES		NEWT	LIV	30	NO	TH	HK	T	C	C	C	7/12/05	7/10/05	I	LA	NAME REMOVED
2005588532	TRIT	TRITURUS	SPECIES		NEWT	LIV	100	NO	DE	DE	T	C	C	C	7/12/05	7/12/05	I	DF	U.S. GLOBAL EXOTICS, INC.
2005589045	BODO	BOLITOGLOSSA	DOFLEINI	ALTA VERAPAZ	SALAMANDER	LIV	20	NO	HN	HN	T	W	C	C	7/13/05	7/13/05	I	LA	LA REPTILES INC
2005589100	BODO	BOLITOGLOSSA	DOFLEINI	ALTA VERAPAZ	SALAMANDER	LIV	20	NO	HN	HN	T	W	C	C	7/13/05	7/13/05	I	LA	LA REPTILES INC
2005590512	TRIT	TRITURUS	SPECIES		NEWT	LIV	1,800	NO	HK	HK	T	C	C	C	7/15/05	7/9/05	I	LA	GLOBAL AQUATIC CONSULTING
2005590513	CYOR	CYNOPS	ORIENTALIS	CHINESE DWARF	NEWT	LIV	250	NO	HK	HK	T	C	C	C	7/15/05	7/9/05	I	LA	DOLPHIN INTERNATIONAL
2005590537	CYOR	CYNOPS	ORIENTALIS	CHINESE DWARF	NEWT	LIV	3,000	NO	CN	CN	T	W	C	C	7/15/05	7/15/05	I	LA	Z IMPORTS INC.
2005591020	CYOR	CYNOPS	ORIENTALIS	CHINESE DWARF	NEWT	LIV	500	NO	HK	HK	T	C	C	C	7/16/05	7/16/05	I	LA	DOLPHIN INTERNATIONAL
2005591037	TRIT	TRITURUS	SPECIES		NEWT	LIV	600	NO	HK	HK	T	C	C	C	7/16/05	7/16/05	I	LA	GLOBAL AQUATIC CONSULTING
2005591108	TRIT	TRITURUS	SPECIES		NEWT	LIV	30	NO	HK	HK	T	C	C	C	7/17/05	7/17/05	I	LA	NAME REMOVED
2005591134	CYOR	CYNOPS	ORIENTALIS	CHINESE DWARF	NEWT	LIV	1,800	NO	HK	HK	T	C	C	C	7/17/05	7/17/05	I	LA	DOLPHIN INTERNATIONAL
2005591134	TYVE	TYLOTOTRITON	VERRUCOSUS	CROCODILE	NEWT	LIV	58	NO	HK	HK	T	C	C	C	7/17/05	7/17/05	I	LA	DOLPHIN INTERNATIONAL
2005591432	SASA	SALAMANDRA	SALAMANDRA	EUROPEAN FIRE	SALAMANDER	LIV	19	NO	VN	VN	T	W	C	C	7/18/05	7/18/05	I	LA	L. A. REPTILE
2005594672	CYOR	CYNOPS	ORIENTALIS	CHINESE DWARF	NEWT	LIV	750	NO	HK	HK	T	C	C	C	7/23/05	7/23/05	I	LA	DOLPHIN INTERNATIONAL
2005594691	TRIT	TRITURUS	SPECIES		NEWT	LIV	1,200	NO	HK	HK	T	C	C	C	7/23/05	7/23/05	I	LA	GLOBAL AQUATIC CONSULTING
2005594750	TRIT	TRITURUS	SPECIES		NEWT	LIV	60	NO	HK	HK	T	C	C	C	7/24/05	7/24/05	I	LA	NAME REMOVED
2005594777	CYOR	CYNOPS	ORIENTALIS	CHINESE DWARF	NEWT	LIV	900	NO	HK	HK	T	C	C	C	7/24/05	7/24/05	I	LA	DOLPHIN INTERNATIONAL
2005594777	TYVE	TYLOTOTRITON	VERRUCOSUS	CROCODILE	NEWT	LIV	35	NO	HK	HK	T	C	C	C	7/24/05	7/24/05	I	LA	DOLPHIN INTERNATIONAL
2005594777	PACB	PACHYTRITON	BREVIPES	TSITOU	NEWT	LIV	200	NO	HK	HK	T	C	C	C	7/24/05	7/24/05	I	LA	DOLPHIN INTERNATIONAL
2005594798	TRIT	TRITURUS	SPECIES		NEWT	LIV	600	NO	HK	HK	T	C	C	C	7/24/05	7/24/05	I	LA	AQUATIC CONNECTION
2005596768	CYOR	CYNOPS	ORIENTALIS	CHINESE DWARF	NEWT	LIV	2,000	NO	CN	CN	T	W	C	C	7/29/05	7/29/05	I	LA	Z IMPORTS INC.
2005597050	CYOR	CYNOPS	ORIENTALIS	CHINESE DWARF	NEWT	LIV	250	NO	HK	HK	T	C	C	C	7/30/05	7/30/05	I	LA	DOLPHIN INTERNATIONAL
2005597062	TRIT	TRITURUS	SPECIES		NEWT	LIV	400	NO	HK	HK	T	C	C	C	7/30/05	7/30/05	I	LA	GLOBAL AQUATIC CONSULTING
2005597157	TRIH	TRITURUS	HONGKONGENSIS	ALPINE	NEWT	LIV	300	NO	HK	HK	T	W	C	C	7/31/05	7/31/05	I	LA	AQUACO, INC.
2005597168	SASA	SALAMANDRA	SALAMANDRA	EUROPEAN FIRE	SALAMANDER	LIV	12	NO	VN	VN	T	W	C	C	7/31/05	7/31/05	I	LA	L. A. REPTILE
2005597174	CYOR	CYNOPS	ORIENTALIS	CHINESE DWARF	NEWT	LIV	1,250	NO	HK	HK	T	C	C	C	7/31/05	7/31/05	I	LA	DOLPHIN INTERNATIONAL
2005597174	TYVE	TYLOTOTRITON	VERRUCOSUS	CROCODILE	NEWT	LIV	200	NO	HK	HK	T	C	C	C	7/31/05	7/31/05	I	LA	DOLPHIN INTERNATIONAL
2005597174	PACB	PACHYTRITON	BREVIPES	TSITOU	NEWT	LIV	100	NO	HK	HK	T	C	C	C	7/31/05	7/31/05	I	LA	DOLPHIN INTERNATIONAL
2005599794	CYOR	CYNOPS	ORIENTALIS	CHINESE DWARF	NEWT	LIV	2,000	NO	CN	CN	T	W	C	C	8/5/05	8/5/05	I	LA	Z IMPORTS INC.
2005600104	CYOR	CYNOPS	ORIENTALIS	CHINESE DWARF	NEWT	LIV	600	NO	HK	HK	T	C	C	C	8/6/05	8/6/05	I	LA	DOLPHIN INTERNATIONAL
2005600111	TRIT	TRITURUS	SPECIES		NEWT	LIV	200	NO	HK	HK	T	C	C	C	8/6/05	8/6/05	I	LA	GLOBAL AQUATIC CONSULTING
2005600151	CYOR	CYNOPS	ORIENTALIS	CHINESE DWARF	NEWT	LIV	1,450	NO	HK	HK	T	C	C	C	8/6/05	8/7/05	I	LA	DOLPHIN INTERNATIONAL
2005600158	TRIH	TRITURUS	HONGKONGENSIS	ALPINE	NEWT	LIV	200	NO	VS	HK	T	W	C	C	8/6/05	8/7/05	I	LA	AQUACO, INC.
2005602986	CYOR	CYNOPS	ORIENTALIS	CHINESE DWARF	NEWT	LIV	500	NO	HK	HK	T	C	C	C	8/13/05	8/13/05	I	LA	DOLPHIN INTERNATIONAL
2005602986	PACB	PACHYTRITON	BREVIPES	TSITOU	NEWT	LIV	100	NO	HK	HK	T	C	C	C	8/13/05	8/13/05	I	LA	DOLPHIN INTERNATIONAL
2005603097	CYOR	CYNOPS	ORIENTALIS	CHINESE DWARF	NEWT	LIV	750	NO	HK	HK	T	C	C	C	8/14/05	8/14/05	I	LA	DOLPHIN INTERNATIONAL
2005603097	TYVE	TYLOTOTRITON	VERRUCOSUS	CROCODILE	NEWT	LIV	50	NO	HK	HK	T	C	C	C	8/14/05	8/14/05	I	LA	DOLPHIN INTERNATIONAL
2005603589	SASA	SALAMANDRA	SALAMANDRA	EUROPEAN FIRE	SALAMANDER	LIV	30	NO	VN	VN	T	W	C	C	8/15/05	8/14/05	I	LA	L. A. REPTILE
2005604584	TYVE	TYLOTOTRITON	VERRUCOSUS	CROCODILE	NEWT	LIV	904	NO	HK	HK	T	W	C	C	8/17/05	8/17/05	I	LA	U.S. GLOBAL EXOTICS, INC.
2005604676	SASA	SALAMANDRA	SALAMANDRA	EUROPEAN FIRE	SALAMANDER	LIV	6	NO	CA	CA	T	C	C	C	8/17/05	8/17/05	I	MI	TWO AMIGOS IMPORT & EXPORT, INC.
2005605512	CYOR	CYNOPS	ORIENTALIS	CHINESE DWARF	NEWT	LIV	500	NO	HK	HK	T	C	C	C	8/20/05	8/20/05	I	LA	DOLPHIN INTERNATIONAL
2005605590	CYOR	CYNOPS	ORIENTALIS	CHINESE DWARF	NEWT	LIV	1,950	NO	HK	HK	T	C	C	C	8/21/05	8/21/05	I	LA	DOLPHIN INTERNATIONAL
2005605590	TYVE	TYLOTOTRITON	VERRUCOSUS	CROCODILE	NEWT	LIV	100	NO	HK	HK	T	C	C	C	8/21/05	8/21/05	I	LA	DOLPHIN INTERNATIONAL
2005605590	PACB	PACHYTRITON	BREVIPES	TSITOU	NEWT	LIV	200	NO	HK	HK	T	C	C	C	8/21/05	8/21/05	I	LA	DOLPHIN INTERNATIONAL
2005605596	TRIT	TRITURUS	SPECIES		NEWT	LIV	1,200	NO	HK	HK	T	C	C	C	8/21/05	8/21/05	I	LA	GLOBAL AQUATIC CONSULTING
2005605608	TRIH	TRITURUS	HONGKONGENSIS	ALPINE	NEWT	LIV	200	NO	HK	HK	T	W	C	C	8/21/05	8/21/05	I	LA	AQUACO, INC.
2005608002	CYOR	CYNOPS	ORIENTALIS	CHINESE DWARF	NEWT	LIV	250	NO	HK	HK	T	C	C	C	8/27/05	8/27/05	I	LA	DOLPHIN INTERNATIONAL
2005608035	TRIT	TRITURUS	SPECIES		NEWT	LIV	400	NO	HK	HK	T	C	C	C	8/27/05	8/27/05	I	LA	GLOBAL AQUATIC CONSULTING
2005608096	CYOR	CYNOPS	ORIENTALIS	CHINESE DWARF	NEWT	LIV	3,200	NO	HK	HK	T	C	C	C	8/27/05	8/28/05	I	LA	DOLPHIN INTERNATIONAL
2005608096	TYVE	TYLOTOTRITON	VERRUCOSUS	CROCODILE	NEWT	LIV	67	NO	HK	HK	T	C	C	C	8/27/05	8/28/05	I	LA	DOLPHIN INTERNATIONAL
2005608096	PACB	PACHYTRITON	BREVIPES	TSITOU	NEWT	LIV	200	NO	HK	HK	T	C	C	C	8/27/05	8/28/05	I	LA	DOLPHIN INTERNATIONAL
2005608113	TRIH	TRITURUS	HONGKONGENSIS	ALPINE	NEWT	LIV	350	NO	HK	HK	T	W	C	C	8/28/05	8/28/05	I	LA	AQUACO, INC.
2005608114	TRIT	TRITURUS	SPECIES		NEWT	LIV	30	NO	HK	HK	T	C	C	C	8/28/05	8/28/05	I	LA	NAME REMOVED
2005611078	CYOR	CYNOPS	ORIENTALIS	CHINESE DWARF	NEWT	LIV	3,000	NO	CN	CN	T	C	C	C	9/2/05	9/2/05	I	LA	Z IMPORTS INC.
2005611459	CYOR	CYNOPS	ORIENTALIS	CHINESE DWARF	NEWT	LIV	250	NO	HK	HK	T	C	C	C	9/2/05	9/3/05	I	LA	DOLPHIN INTERNATIONAL

2005611493	TRIT	TRITURUS	SPECIES		NEWT	LIV	200	NO	HK	HK	T	C	C	C	9/3/05	9/3/05	I	LA	GLOBAL AQUATIC CONSULTING
2005611564	CYOR	CYNOPS	ORIENTALIS	CHINESE DWARF	NEWT	LIV	950	NO	HK	HK	T	C	C	C	9/3/05	9/4/05	I	LA	DOLPHIN INTERNATIONAL
2005611564	PACB	PACHYTRITON	BREVIPES	TSITOU	NEWT	LIV	200	NO	HK	HK	T	C	C	C	9/3/05	9/4/05	I	LA	DOLPHIN INTERNATIONAL
2005611575	TRIH	TRITURUS	HONGKONGENSIS	ALPINE	NEWT	LIV	550	NO	HK	HK	T	W	C	C	9/3/05	9/4/05	I	LA	AQUACO, INC.
2005611644	CNP?	CYNOPS	SPECIES	FIREBELLY	NEWT	LIV	6	NO	CN	CN	S	W	C	C	7/28/05	7/27/05	I	SF	DEPARTMENT OF HERPETOLOGY
2005613714	CYOR	CYNOPS	ORIENTALIS	CHINESE DWARF	NEWT	LIV	3,000	NO	CN	CN	T	C	C	C	9/9/05	9/9/05	I	LA	Z IMPORTS INC.
2005613860	CYPY	CYNOPS	PYRRHOGASTER	JAPANESE FIREBELLY	NEWT	LIV	200	NO	HK	HK	T	W	C	C	3/28/05	3/26/05	I	NY	TRANSSHIP DISCOUNTS , LTD.
2005613896	CYPY	CYNOPS	PYRRHOGASTER	JAPANESE FIREBELLY	NEWT	LIV	1,350	NO	HK	HK	T	W	C	C	3/28/05	3/26/05	I	NY	ERDA INCORPORATED
2005614187	CYOR	CYNOPS	ORIENTALIS	CHINESE DWARF	NEWT	LIV	500	NO	HK	HK	T	C	C	C	9/10/05	9/10/05	I	LA	DOLPHIN INTERNATIONAL
2005614195	TRIT	TRITURUS	SPECIES		NEWT	LIV	200	NO	HK	HK	T	C	C	C	9/10/05	9/10/05	I	LA	GLOBAL AQUATIC CONSULTING
2005614266	CYOR	CYNOPS	ORIENTALIS	CHINESE DWARF	NEWT	LIV	6,800	NO	HK	HK	T	C	C	C	9/11/05	9/11/05	I	LA	DOLPHIN INTERNATIONAL
2005614266	PACB	PACHYTRITON	BREVIPES	TSITOU	NEWT	LIV	200	NO	HK	HK	T	C	C	C	9/11/05	9/11/05	I	LA	DOLPHIN INTERNATIONAL
2005614281	TRIT	TRITURUS	SPECIES		NEWT	LIV	30	NO	HK	HK	T	W	C	C	9/11/05	9/11/05	I	LA	NAME REMOVED
2005614292	TRIH	TRITURUS	HONGKONGENSIS	ALPINE	NEWT	LIV	50	NO	HK	HK	T	W	C	C	9/11/05	9/11/05	I	LA	AQUACO, INC.
2005615280	CYPY	CYNOPS	PYRRHOGASTER	JAPANESE FIREBELLY	NEWT	LIV	50	NO	HK	HK	T	W	C	C	7/24/05	7/16/05	I	NY	TRANSSHIP DISCOUNTS , LTD.
2005616201	CYOR	CYNOPS	ORIENTALIS	CHINESE DWARF	NEWT	LIV	100	NO	SG	SG	T	W	C	C	6/28/05	6/12/05	I	NY	INTERNATIONAL PET RESOURCES , LLC
2005616318	CYPY	CYNOPS	PYRRHOGASTER	JAPANESE FIREBELLY	NEWT	LIV	405	NO	HK	HK	T	W	C	C	6/22/05	6/18/05	I	NY	ERDA INCORPORATED
2005616562	CYPY	CYNOPS	PYRRHOGASTER	JAPANESE FIREBELLY	NEWT	LIV	300	NO	HK	HK	T	W	C	C	6/28/05	6/25/05	I	NY	ERDA INCORPORATED
2005616610	CYPY	CYNOPS	PYRRHOGASTER	JAPANESE FIREBELLY	NEWT	LIV	570	NO	HK	HK	T	W	C	C	7/7/05	7/2/05	I	NY	ERDA INCORPORATED
2005616951	CYPY	CYNOPS	PYRRHOGASTER	JAPANESE FIREBELLY	NEWT	LIV	200	NO	HK	HK	T	W	C	C	6/28/05	5/25/05	I	NY	TRANSSHIP DISCOUNTS , LTD.
2005617107	CYPY	CYNOPS	PYRRHOGASTER	JAPANESE FIREBELLY	NEWT	LIV	500	NO	CN	CN	T	W	C	C	7/13/05	6/26/05	I	NY	FISH MART INCORPORATED
2005617107	CNP?	CYNOPS	SPECIES	FIREBELLY	NEWT	LIV	100	NO	CN	CN	T	W	C	C	7/13/05	6/26/05	I	NY	FISH MART INCORPORATED
2005617263	CNP?	CYNOPS	SPECIES	FIREBELLY	NEWT	LIV	18	NO	HK	HK	T	W	C	C	9/15/05	9/15/05	I	LA	Exemptions 6 and 7(C)
2005617263	TYL?	TYLOTOTRITON	SPECIES	CROCODILE	NEWT	LIV	45	NO	HK	HK	T	W	C	C	9/15/05	9/15/05	I	LA	Exemptions 6 and 7(C)
2005617263	TYVE	TYLOTOTRITON	VERRUCOSUS	CROCODILE	NEWT	LIV	2	NO	HK	HK	T	W	C	C	9/15/05	9/15/05	I	LA	Exemptions 6 and 7(C)
2005617263	PDEL	PARAMESOTRITON	DELOUSTALI	VIETNAM WARTY	NEWT	LIV	8	NO	HK	HK	T	W	C	C	9/15/05	9/15/05	I	LA	Exemptions 6 and 7(C)
2005617263	PCHI	PARAMESOTRITON	CHINENSIS	CHINESE WARTY	NEWT	LIV	80	NO	HK	HK	T	W	C	C	9/15/05	9/15/05	I	LA	Exemptions 6 and 7(C)
2005617263	NEW?	PACHYTRITON	SPECIES	CHINESE	NEWT	LIV	112	NO	HK	HK	T	W	C	C	9/15/05	9/15/05	I	LA	Exemptions 6 and 7(C)
2005617263	BTC?	BATRACHUPERUS	SPECIES	MOUNTAIN	SALAMANDER	LIV	7	NO	HK	HK	T	W	C	C	9/15/05	9/15/05	I	LA	Exemptions 6 and 7(C)
2005617263	PSHG	PACHYHYNOBIUS	SHANGCHENGENSIS	SHANGCHENG	SALAMANDER	LIV	20	NO	HK	HK	T	W	C	C	9/15/05	9/15/05	I	LA	Exemptions 6 and 7(C)
2005617351	CYPY	CYNOPS	PYRRHOGASTER	JAPANESE FIREBELLY	NEWT	LIV	750	NO	HK	HK	T	W	C	C	5/23/05	5/21/05	I	NY	ERDA INCORPORATED
2005617424	CYOR	CYNOPS	ORIENTALIS	CHINESE DWARF	NEWT	LIV	200	NO	CN	CN	T	F	C	C	7/6/05	7/1/05	I	NY	MIN HUA ENTERPRISE, INC.
2005617855	BODO	BOLITOGLOSSA	DOFLEINI	ALTA VERAPAZ	SALAMANDER	LIV	10	NO	HN	HN	T	W	C	C	9/16/05	9/1/05	I	LA	LA REPTILES INC
2005617869	CYPY	CYNOPS	PYRRHOGASTER	JAPANESE FIREBELLY	NEWT	LIV	380	NO	HK	HK	T	W	C	C	7/13/05	7/9/05	I	NY	ERDA INCORPORATED
2005617917	NEW?	PACHYTRITON	SPECIES	CHINESE	NEWT	LIV	800	NO	CN	CN	T	C	C	C	9/16/05	9/16/05	I	LA	DA TIEN PETS EMPORIUM INC
2005618224	CYPY	CYNOPS	PYRRHOGASTER	JAPANESE FIREBELLY	NEWT	LIV	660	NO	HK	HK	T	W	C	C	8/4/05	7/30/05	I	NY	ERDA INCORPORATED
2005618288	CYOR	CYNOPS	ORIENTALIS	CHINESE DWARF	NEWT	LIV	200	NO	CN	CN	T	F	C	C	6/27/05	6/15/05	I	NY	MIN HUA ENTERPRISE, INC.
2005618469	CYOR	CYNOPS	ORIENTALIS	CHINESE DWARF	NEWT	LIV	500	NO	HK	HK	T	C	C	C	9/17/05	9/17/05	I	LA	DOLPHIN INTERNATIONAL
2005618498	TRIT	TRITURUS	SPECIES		NEWT	LIV	600	NO	HK	HK	T	C	C	C	9/17/05	9/17/05	I	LA	GLOBAL AQUATIC CONSULTING
2005618622	CYPY	CYNOPS	PYRRHOGASTER	JAPANESE FIREBELLY	NEWT	LIV	250	NO	HK	HK	T	W	C	C	8/11/05	8/6/05	I	NY	ERDA INCORPORATED
2005618977	TRIH	TRITURUS	HONGKONGENSIS	ALPINE	NEWT	LIV	200	NO	VS	HK	T	W	C	C	9/19/05	9/18/05	I	LA	AQUACO, INC.
2005619027	CYOR	CYNOPS	ORIENTALIS	CHINESE DWARF	NEWT	LIV	2,200	NO	HK	HK	T	C	C	C	9/19/05	9/18/05	I	LA	DOLPHIN INTERNATIONAL
2005619027	PACB	PACHYTRITON	BREVIPES	TSITOU	NEWT	LIV	200	NO	HK	HK	T	C	C	C	9/19/05	9/18/05	I	LA	DOLPHIN INTERNATIONAL
2005620261	SASA	SALAMANDRA	SALAMANDRA	EUROPEAN FIRE	SALAMANDER	LIV	100	NO	GB	GB	T	W	C	C	9/21/05	9/19/05	I	MI	TWO AMIGOS IMPORT & EXPORT, INC.
2005620261	TRIT	TRITURUS	SPECIES		NEWT	LIV	250	NO	GB	GB	T	W	C	C	9/21/05	9/19/05	I	MI	TWO AMIGOS IMPORT & EXPORT, INC.
2005621152	CYPY	CYNOPS	PYRRHOGASTER	JAPANESE FIREBELLY	NEWT	LIV	200	NO	HK	HK	T	W	C	C	7/17/05	7/9/05	I	NY	TRANSSHIP DISCOUNTS , LTD.
2005621180	TRIH	TRITURUS	HONGKONGENSIS	ALPINE	NEWT	LIV	200	NO	HK	HK	T	W	C	C	8/4/05	7/17/05	I	NY	INTERNATIONAL PET RESOURCES , LLC
2005621206	CYPY	CYNOPS	PYRRHOGASTER	JAPANESE FIREBELLY	NEWT	LIV	50	NO	HK	HK	T	W	C	C	8/4/05	7/30/05	I	NY	TRANSSHIP DISCOUNTS , LTD.
2005621206	PACB	PACHYTRITON	BREVIPES	TSITOU	NEWT	LIV	30	NO	HK	HK	T	W	C	C	8/4/05	7/30/05	I	NY	TRANSSHIP DISCOUNTS , LTD.
2005621240	TRIH	TRITURUS	HONGKONGENSIS	ALPINE	NEWT	LIV	400	NO	HK	HK	T	W	C	C	8/11/05	6/26/05	I	NY	INTERNATIONAL PET RESOURCES , LLC
2005621757	CYPY	CYNOPS	PYRRHOGASTER	JAPANESE FIREBELLY	NEWT	LIV	745	NO	HK	HK	T	W	C	C	7/29/05	7/23/05	I	NY	ERDA INCORPORATED
2005622157	CYPY	CYNOPS	PYRRHOGASTER	JAPANESE FIREBELLY	NEWT	LIV	150	NO	HK	HK	T	W	C	C	6/22/05	6/18/05	I	NY	TRANSSHIP DISCOUNTS , LTD.
2005622157	PACB	PACHYTRITON	BREVIPES	TSITOU	NEWT	LIV	60	NO	HK	HK	T	W	C	C	6/22/05	6/18/05	I	NY	TRANSSHIP DISCOUNTS , LTD.
2005622301	CYPY	CYNOPS	PYRRHOGASTER	JAPANESE FIREBELLY	NEWT	LIV	50	NO	HK	HK	T	W	C	C	6/2/05	5/2/05	I	NY	TRANSSHIP DISCOUNTS , LTD.
2005622421	CYOR	CYNOPS	ORIENTALIS	CHINESE DWARF	NEWT	LIV	3,000	NO	CN	CN	T	C	C	C	9/23/05	9/23/05	I	LA	Z IMPORTS INC.
2005622859	CYOR	CYNOPS	ORIENTALIS	CHINESE DWARF	NEWT	LIV	600	NO	HK	HK	T	C	C	C	9/24/05	9/24/05	I	LA	DOLPHIN INTERNATIONAL
2005622944	CYOR	CYNOPS	ORIENTALIS	CHINESE DWARF	NEWT	LIV	100	NO	HK	HK	T	C	C	C	9/25/05	9/25/05	I	LA	DOLPHIN INTERNATIONAL
2005622944	PACB	PACHYTRITON	BREVIPES	TSITOU	NEWT	LIV	200	NO	HK	HK	T	C	C	C	9/25/05	9/25/05	I	LA	DOLPHIN INTERNATIONAL
2005623416	PLAB	PACHYTRITON	LABIATUS	UNTERSTEIN'S	NEWT	LIV	200	NO	CN	CN	T	W	C	C	9/26/05	9/22/05	I	SE	NAME REMOVED
2005623416	CNP?	CYNOPS	SPECIES	FIREBELLY	NEWT	LIV	100	NO	CN	CN	T	W	C	C	9/26/05	9/22/05	I	SE	NAME REMOVED

2005623437	CYPY	CYNOPS	PYRRHOGASTER	JAPANESE FIREBELLY	NEWT	LIV	50	NO	HK	HK	T	W	C	C	5/23/05	5/21/05	I	NY	TRANSSHIP DISCOUNTS , LTD.
2005624241	NEW?	PACHYTRITON	SPECIES	CHINESE	NEWT	LIV	200	NO	CN	CN	T	C	C	C	9/27/05	9/27/05	I	SF	Golden Phoenix Fisheries, USA
2005626148	CYOR	CYNOPS	ORIENTALIS	CHINESE DWARF	NEWT	LIV	3,000	NO	CN	CN	T	C	C	C	9/30/05	9/30/05	I	LA	Z IMPORTS INC.
2005626495	PACB	PACHYTRITON	BREVIPES	TSITOU	NEWT	LIV	100	NO	HK	HK	T	C	C	C	10/1/05	10/1/05	I	LA	DOLPHIN INTERNATIONAL
2005626662	TRIH	TRITURUS	HONGKONGENSIS	ALPINE	NEWT	LIV	450	NO	HK	HK	T	W	C	C	10/3/05	10/2/05	I	LA	AQUACO, INC.
2005626690	CYOR	CYNOPS	ORIENTALIS	CHINESE DWARF	NEWT	LIV	1,050	NO	HK	HK	T	C	C	C	10/3/05	10/2/05	I	LA	DOLPHIN INTERNATIONAL
2005629083	BODO	BOLITOGLOSSA	DOFLEINI	ALTA VERAPAZ	SALAMANDER	LIV	30	NO	GT	GT	T	W	C	C	8/25/05	8/25/05	I	MI	DHAR TROPICAL LLC
2005629238	NEW?	PACHYTRITON	SPECIES	CHINESE	NEWT	LIV	800	NO	CN	CN	T	C	C	C	10/7/05	10/7/05	I	LA	DA TIEN PETS EMPORIUM INC
2005629247	SAL?	SALAMANDRA	SPECIES	FIRE	SALAMANDER	LIV	100	NO	SI	SI	T	C	C	C	10/7/05	10/7/05	I	LA	L. A. REPTILE
2005629922	TRIT	TRITURUS	SPECIES		NEWT	LIV	2,800	NO	HK	HK	T	C	C	C	10/8/05	10/8/05	I	LA	GLOBAL AQUATIC CONSULTING
2005629970	CYOR	CYNOPS	ORIENTALIS	CHINESE DWARF	NEWT	LIV	1,100	NO	HK	HK	T	C	C	C	10/8/05	10/9/05	I	LA	DOLPHIN INTERNATIONAL
2005629970	PACB	PACHYTRITON	BREVIPES	TSITOU	NEWT	LIV	300	NO	HK	HK	T	C	C	C	10/8/05	10/9/05	I	LA	DOLPHIN INTERNATIONAL
2005629994	TRIH	TRITURUS	HONGKONGENSIS	ALPINE	NEWT	LIV	475	NO	HK	HK	T	W	C	C	10/8/05	10/9/05	I	LA	AQUACO, INC.
2005630022	TRIT	TRITURUS	SPECIES		NEWT	LIV	700	NO	VS	HK	T	C	C	C	10/9/05	10/9/05	I	LA	AQUATIC CONNECTION
2005630276	BODO	BOLITOGLOSSA	DOFLEINI	ALTA VERAPAZ	SALAMANDER	LIV	40	NO	GT	GT	T	W	C	C	8/11/05	8/11/05	I	MI	D.H.A.R. TROPICALS LLC
2005630276	BMEX	BOLITOGLOSSA	MEXICANA	MEX MUSHROOMTONGUE	SALAMANDER	LIV	1	NO	GT	GT	T	W	C	C	8/11/05	8/11/05	I	MI	D.H.A.R. TROPICALS LLC
2005631287	BODO	BOLITOGLOSSA	DOFLEINI	ALTA VERAPAZ	SALAMANDER	LIV	25	NO	HN	HN	T	W	C	C	9/21/05	9/21/05	I	MI	NAME REMOVED
2005633560	CYOR	CYNOPS	ORIENTALIS	CHINESE DWARF	NEWT	LIV	3,000	NO	CN	CN	T	C	C	C	10/14/05	10/14/05	I	LA	Z IMPORTS INC.
2005633602	CYPY	CYNOPS	PYRRHOGASTER	JAPANESE FIREBELLY	NEWT	LIV	200	NO	CN	CN	T	C	C	C	8/18/05	8/15/05	I	NY	FISH MART INCORPORATED
2005634103	TRIT	TRITURUS	SPECIES		NEWT	LIV	600	NO	HK	HK	T	C	C	C	10/15/05	10/15/05	I	LA	GLOBAL AQUATIC CONSULTING
2005634110	CYOR	CYNOPS	ORIENTALIS	CHINESE DWARF	NEWT	LIV	1,100	NO	HK	HK	T	C	C	C	10/15/05	10/15/05	I	LA	DOLPHIN INTERNATIONAL
2005634110	PACB	PACHYTRITON	BREVIPES	TSITOU	NEWT	LIV	100	NO	HK	HK	T	C	C	C	10/15/05	10/15/05	I	LA	DOLPHIN INTERNATIONAL
2005634180	CYOR	CYNOPS	ORIENTALIS	CHINESE DWARF	NEWT	LIV	4,900	NO	HK	HK	T	C	C	C	10/16/05	10/16/05	I	LA	DOLPHIN INTERNATIONAL
2005634180	PACB	PACHYTRITON	BREVIPES	TSITOU	NEWT	LIV	100	NO	HK	HK	T	C	C	C	10/16/05	10/16/05	I	LA	DOLPHIN INTERNATIONAL
2005634190	TRIT	TRITURUS	SPECIES		NEWT	LIV	120	NO	HK	HK	T	C	C	C	10/16/05	10/16/05	I	LA	NAME REMOVED
2005634206	TRIH	TRITURUS	HONGKONGENSIS	ALPINE	NEWT	LIV	50	NO	HK	HK	T	W	C	C	10/16/05	10/16/05	I	LA	AQUACO, INC.
2005634286	CYPY	CYNOPS	PYRRHOGASTER	JAPANESE FIREBELLY	NEWT	LIV	130	NO	HK	HK	T	W	C	C	8/30/05	8/27/05	I	NY	ERDA INCORPORATED
2005634363	CYPY	CYNOPS	PYRRHOGASTER	JAPANESE FIREBELLY	NEWT	LIV	660	NO	HK	HK	T	W	C	C	9/22/05	9/18/05	I	NY	TRANSSHIP DISCOUNTS , LTD.
2005634408	CYPY	CYNOPS	PYRRHOGASTER	JAPANESE FIREBELLY	NEWT	LIV	375	NO	HK	HK	T	W	C	C	9/22/05	9/17/05	I	NY	ERDA INCORPORATED
2005635804	CYPY	CYNOPS	PYRRHOGASTER	JAPANESE FIREBELLY	NEWT	LIV	580	NO	HK	HK	T	W	C	C	9/6/05	9/3/05	I	NY	ERDA INCORPORATED
2005636152	CYPY	CYNOPS	PYRRHOGASTER	JAPANESE FIREBELLY	NEWT	LIV	200	NO	CN	CN	T	C	C	C	8/16/05	7/24/05	I	NY	FISH MART INCORPORATED
2005636152	CNP?	CYNOPS	SPECIES	FIREBELLY	NEWT	LIV	1	NO	CN	CN	T	C	C	C	8/16/05	7/24/05	I	NY	FISH MART INCORPORATED
2005637332	CYPY	CYNOPS	PYRRHOGASTER	JAPANESE FIREBELLY	NEWT	LIV	720	NO	HK	HK	T	W	C	C	8/24/05	8/20/05	I	NY	ERDA INCORPORATED
2005637809	CYPY	CYNOPS	PYRRHOGASTER	JAPANESE FIREBELLY	NEWT	LIV	310	NO	HK	HK	T	W	C	C	8/30/05	8/28/05	I	NY	TRANSSHIP DISCOUNTS , LTD.
2005638084	CYOR	CYNOPS	ORIENTALIS	CHINESE DWARF	NEWT	LIV	3,000	NO	CN	CN	T	C	C	C	10/21/05	10/21/05	I	LA	Z IMPORTS INC.
2005638701	CYOR	CYNOPS	ORIENTALIS	CHINESE DWARF	NEWT	LIV	250	NO	HK	HK	T	C	C	C	10/22/05	10/22/05	I	LA	DOLPHIN INTERNATIONAL
2005638745	TRIT	TRITURUS	SPECIES		NEWT	LIV	800	NO	HK	HK	T	C	C	C	10/22/05	10/22/05	I	LA	GLOBAL AQUATIC CONSULTING
2005638830	CYOR	CYNOPS	ORIENTALIS	CHINESE DWARF	NEWT	LIV	2,150	NO	HK	HK	T	C	C	C	10/23/05	10/23/05	I	LA	DOLPHIN INTERNATIONAL
2005638830	PACB	PACHYTRITON	BREVIPES	TSITOU	NEWT	LIV	100	NO	HK	HK	T	C	C	C	10/23/05	10/23/05	I	LA	DOLPHIN INTERNATIONAL
2005638859	TRIT	TRITURUS	SPECIES		NEWT	LIV	300	NO	HK	HK	T	C	C	C	10/23/05	10/23/05	I	LA	AQUATIC CONNECTION
2005638863	TRIH	TRITURUS	HONGKONGENSIS	ALPINE	NEWT	LIV	625	NO	HK	HK	T	W	C	C	10/23/05	10/23/05	I	LA	AQUACO, INC.
2005639647	CYPY	CYNOPS	PYRRHOGASTER	JAPANESE FIREBELLY	NEWT	LIV	1	NO	CN	CN	T	W	C	C	3/7/05	2/27/05	I	NY	FISH MART INCORPORATED
2005639647	CNP?	CYNOPS	SPECIES	FIREBELLY	NEWT	LIV	1	NO	CN	CN	T	W	C	C	3/7/05	2/27/05	I	NY	FISH MART INCORPORATED
2005640729	CYPY	CYNOPS	PYRRHOGASTER	JAPANESE FIREBELLY	NEWT	LIV	70	NO	HK	HK	T	W	C	C	8/23/05	8/21/05	I	NY	TRANSSHIP DISCOUNTS , LTD.
2005641135	CYPY	CYNOPS	PYRRHOGASTER	JAPANESE FIREBELLY	NEWT	LIV	300	NO	HK	HK	T	W	C	C	4/25/05	4/23/05	I	NY	ERDA INCORPORATED
2005641770	CYPY	CYNOPS	PYRRHOGASTER	JAPANESE FIREBELLY	NEWT	LIV	70	NO	HK	HK	T	W	C	C	3/7/05	3/5/05	I	NY	TRANSSHIP DISCOUNTS , LTD.
2005641781	TRIH	TRITURUS	HONGKONGENSIS	ALPINE	NEWT	LIV	600	NO	HK	HK	T	W	C	C	8/16/05	7/31/05	I	NY	INTERNATIONAL PET RESOURCES , LLC
2005641801	CYPY	CYNOPS	PYRRHOGASTER	JAPANESE FIREBELLY	NEWT	LIV	400	NO	CN	CN	T	W	C	C	5/4/05	5/1/05	I	NY	FISH MART INCORPORATED
2005641801	CNP?	CYNOPS	SPECIES	FIREBELLY	NEWT	LIV	200	NO	CN	CN	T	W	C	C	5/4/05	5/1/05	I	NY	FISH MART INCORPORATED
2005642021	SASA	SALAMANDRA	SALAMANDRA	EUROPEAN FIRE	SALAMANDER	LIV	1,000	NO	SI	SI	T	C	C	C	10/28/05	10/28/05	I	DF	U.S. GLOBAL EXOTICS, INC.
2005642354	TRIT	TRITURUS	SPECIES		NEWT	LIV	200	NO	HK	HK	T	C	C	C	10/29/05	10/29/05	I	LA	GLOBAL AQUATIC CONSULTING
2005642362	CYOR	CYNOPS	ORIENTALIS	CHINESE DWARF	NEWT	LIV	750	NO	HK	HK	T	C	C	C	10/29/05	10/29/05	I	LA	DOLPHIN INTERNATIONAL
2005642404	CYOR	CYNOPS	ORIENTALIS	CHINESE DWARF	NEWT	LIV	1,100	NO	HK	HK	T	C	C	C	10/30/05	10/30/05	I	LA	DOLPHIN INTERNATIONAL
2005642404	PACB	PACHYTRITON	BREVIPES	TSITOU	NEWT	LIV	800	NO	HK	HK	T	C	C	C	10/30/05	10/30/05	I	LA	DOLPHIN INTERNATIONAL
2005642411	TRIT	TRITURUS	SPECIES		NEWT	LIV	60	NO	HK	HK	T	C	C	C	10/30/05	10/30/05	I	LA	NAME REMOVED
2005642422	TRIT	TRITURUS	SPECIES		NEWT	LIV	600	NO	HK	HK	T	C	C	C	10/30/05	10/30/05	I	LA	AQUATIC CONNECTION
2005642423	TRIH	TRITURUS	HONGKONGENSIS	ALPINE	NEWT	LIV	75	NO	HK	HK	T	W	C	C	10/30/05	10/30/05	I	LA	AQUACO, INC.
2005642806	CYPY	CYNOPS	PYRRHOGASTER	JAPANESE FIREBELLY	NEWT	LIV	375	NO	HK	HK	T	W	C	C	7/21/05	7/16/05	I	NY	ERDA INCORPORATED
2005643463	CYPY	CYNOPS	PYRRHOGASTER	JAPANESE FIREBELLY	NEWT	LIV	340	NO	HK	HK	T	W	C	C	6/14/05	6/11/05	I	NY	ERDA INCORPORATED
2005643487	CYPY	CYNOPS	PYRRHOGASTER	JAPANESE FIREBELLY	NEWT	LIV	300	NO	HK	HK	T	W	C	C	6/14/05	6/11/05	I	NY	TRANSSHIP DISCOUNTS , LTD.

2005643487	PACB	PACHYTRITON	BREVIPES	TSITOU	NEWT	LIV	60	NO	HK	HK	T	W	C	C	6/14/05	6/11/05	I	NY	TRANSSHIP DISCOUNTS , LTD.
2005643835	PACB	PACHYTRITON	BREVIPES	TSITOU	NEWT	LIV	60	NO	HK	HK	T	W	C	C	9/14/05	9/11/05	I	NY	TRANSSHIP DISCOUNTS , LTD.
2005643890	CYPY	CYNOPS	PYRRHOGASTER	JAPANESE FIREBELLY	NEWT	LIV	210	NO	HK	HK	T	W	C	C	9/14/05	9/10/05	I	NY	ERDA INCORPORATED
2005644516	CYPY	CYNOPS	PYRRHOGASTER	JAPANESE FIREBELLY	NEWT	LIV	150	NO	HK	HK	T	W	C	C	8/15/05	8/13/05	I	NY	ERDA INCORPORATED
2005644579	CYPY	CYNOPS	PYRRHOGASTER	JAPANESE FIREBELLY	NEWT	LIV	300	NO	HK	HK	T	C	C	C	9/20/05	8/23/05	I	NY	WORLDWIDE AQUATICS OF LONG ISLAND, INC.
2005644599	CYPY	CYNOPS	PYRRHOGASTER	JAPANESE FIREBELLY	NEWT	LIV	320	NO	HK	HK	T	W	C	C	9/30/05	9/25/05	I	NY	TRANSSHIP DISCOUNTS , LTD.
2005644646	CYPY	CYNOPS	PYRRHOGASTER	JAPANESE FIREBELLY	NEWT	LIV	60	NO	HK	HK	T	W	C	C	2/8/05	2/5/05	I	NY	TRANSSHIP DISCOUNTS , LTD.
2005644660	CYPY	CYNOPS	PYRRHOGASTER	JAPANESE FIREBELLY	NEWT	LIV	25	NO	HK	HK	T	W	C	C	2/7/05	2/5/05	I	NY	ERDA INCORPORATED
2005644743	CYPY	CYNOPS	PYRRHOGASTER	JAPANESE FIREBELLY	NEWT	LIV	550	NO	HK	HK	T	W	C	C	10/18/05	10/15/05	I	NY	ERDA INCORPORATED
2005644841	CYPY	CYNOPS	PYRRHOGASTER	JAPANESE FIREBELLY	NEWT	LIV	50	NO	HK	HK	T	W	C	C	7/24/05	7/23/05	I	NY	TRANSSHIP DISCOUNTS , LTD.
2005645040	CYPY	CYNOPS	PYRRHOGASTER	JAPANESE FIREBELLY	NEWT	LIV	20	NO	HK	HK	T	W	C	C	10/6/05	10/2/05	I	NY	TRANSSHIP DISCOUNTS , LTD.
2005645224	CYPY	CYNOPS	PYRRHOGASTER	JAPANESE FIREBELLY	NEWT	LIV	50	NO	HK	HK	T	W	C	C	8/15/05	8/15/05	I	NY	TRANSSHIP DISCOUNTS , LTD.
2005645341	CYPY	CYNOPS	PYRRHOGASTER	JAPANESE FIREBELLY	NEWT	LIV	50	NO	HK	HK	T	W	C	C	1/25/05	1/22/05	I	NY	ERDA INCORPORATED
2005645450	TRIH	TRITURUS	HONGKONGENSIS	ALPINE	NEWT	LIV	400	NO	HK	HK	T	W	C	C	5/24/05	5/8/05	I	NY	INTERNATIONAL PET RESOURCES , LLC
2005645874	CYOR	CYNOPS	ORIENTALIS	CHINESE DWARF	NEWT	LIV	200	NO	CN	CN	T	C	C	C	9/1/05	8/28/05	I	NY	FISH MART INCORPORATED
2005646486	CYOR	CYNOPS	ORIENTALIS	CHINESE DWARF	NEWT	LIV	3,000	NO	CN	CN	T	C	C	C	11/4/05	11/4/05	I	LA	Z IMPORTS INC.
2005647107	CYOR	CYNOPS	ORIENTALIS	CHINESE DWARF	NEWT	LIV	250	NO	HK	HK	T	C	C	C	11/5/05	11/5/05	I	LA	DOLPHIN INTERNATIONAL
2005647107	PACB	PACHYTRITON	BREVIPES	TSITOU	NEWT	LIV	100	NO	HK	HK	T	C	C	C	11/5/05	11/5/05	I	LA	DOLPHIN INTERNATIONAL
2005647119	TRIT	TRITURUS	SPECIES		NEWT	LIV	600	NO	HK	HK	T	C	C	C	11/5/05	11/5/05	I	LA	GLOBAL AQUATIC CONSULTING
2005647223	CYOR	CYNOPS	ORIENTALIS	CHINESE DWARF	NEWT	LIV	1,100	NO	HK	HK	T	C	C	C	11/6/05	11/6/05	I	LA	DOLPHIN INTERNATIONAL
2005647223	PACB	PACHYTRITON	BREVIPES	TSITOU	NEWT	LIV	100	NO	HK	HK	T	C	C	C	11/6/05	11/6/05	I	LA	DOLPHIN INTERNATIONAL
2005647244	TRIH	TRITURUS	HONGKONGENSIS	ALPINE	NEWT	LIV	125	NO	HK	HK	T	W	C	C	11/6/05	11/6/05	I	LA	AQUACO, INC.
2005647256	TRIT	TRITURUS	SPECIES		NEWT	LIV	600	NO	HK	HK	T	C	C	C	11/6/05	11/6/05	I	LA	AQUATIC CONNECTION
2005651691	CYPY	CYNOPS	PYRRHOGASTER	JAPANESE FIREBELLY	NEWT	LIV	800	NO	HK	HK	T	W	C	C	10/14/05	10/8/05	I	NY	ERDA INCORPORATED
2005651848	CYPY	CYNOPS	PYRRHOGASTER	JAPANESE FIREBELLY	NEWT	LIV	70	NO	HK	HK	T	W	C	C	10/17/05	10/16/05	I	NY	TRANSSHIP DISCOUNTS , LTD.
2005651967	CYOR	CYNOPS	ORIENTALIS	CHINESE DWARF	NEWT	LIV	1,500	NO	CN	CN	T	C	C	C	11/11/05	11/11/05	I	LA	Z IMPORTS INC.
2005652081	CYPY	CYNOPS	PYRRHOGASTER	JAPANESE FIREBELLY	NEWT	LIV	238	NO	HK	HK	T	W	C	C	10/24/05	10/22/05	I	NY	ERDA INCORPORATED
2005652240	CYOR	CYNOPS	ORIENTALIS	CHINESE DWARF	NEWT	LIV	250	NO	HK	HK	T	C	C	C	11/12/05	11/12/05	I	LA	DOLPHIN INTERNATIONAL
2005652258	TRIT	TRITURUS	SPECIES		NEWT	LIV	800	NO	HK	HK	T	C	C	C	11/12/05	11/12/05	I	LA	GLOBAL AQUATIC CONSULTING
2005652376	CYOR	CYNOPS	ORIENTALIS	CHINESE DWARF	NEWT	LIV	7,800	NO	HK	HK	T	C	C	C	11/12/05	11/13/05	I	LA	DOLPHIN INTERNATIONAL
2005652376	PACB	PACHYTRITON	BREVIPES	TSITOU	NEWT	LIV	100	NO	HK	HK	T	C	C	C	11/12/05	11/13/05	I	LA	DOLPHIN INTERNATIONAL
2005652418	TRIH	TRITURUS	HONGKONGENSIS	ALPINE	NEWT	LIV	25	NO	HK	HK	T	W	C	C	11/13/05	11/13/05	I	LA	AQUACO, INC.
2005652436	TRIT	TRITURUS	SPECIES		NEWT	LIV	300	NO	HK	HK	T	C	C	C	11/13/05	11/13/05	I	LA	AQUATIC CONNECTION
2005653280	CYPY	CYNOPS	PYRRHOGASTER	JAPANESE FIREBELLY	NEWT	LIV	200	NO	CN	CN	T	C	C	C	10/21/05	10/16/05	I	NY	FISH MART INCORPORATED
2005656316	CYOR	CYNOPS	ORIENTALIS	CHINESE DWARF	NEWT	LIV	4,000	NO	CN	CN	T	C	C	C	11/18/05	11/18/05	I	LA	Z IMPORTS INC.
2005656873	CYOR	CYNOPS	ORIENTALIS	CHINESE DWARF	NEWT	LIV	250	NO	HK	HK	T	C	C	C	11/19/05	11/19/05	I	LA	DOLPHIN INTERNATIONAL
2005656917	TRIT	TRITURUS	SPECIES		NEWT	LIV	400	NO	HK	HK	T	C	C	C	11/19/05	11/19/05	I	LA	GLOBAL AQUATIC CONSULTING
2005656985	TRIH	TRITURUS	HONGKONGENSIS	ALPINE	NEWT	LIV	275	NO	HK	HK	T	W	C	C	11/20/05	11/20/05	I	LA	AQUACO, INC.
2005657012	CYOR	CYNOPS	ORIENTALIS	CHINESE DWARF	NEWT	LIV	175	NO	HK	HK	T	C	C	C	11/20/05	11/20/05	I	LA	DOLPHIN INTERNATIONAL
2005657012	PACB	PACHYTRITON	BREVIPES	TSITOU	NEWT	LIV	100	NO	HK	HK	T	C	C	C	11/20/05	11/20/05	I	LA	DOLPHIN INTERNATIONAL
2005659503	CYOR	CYNOPS	ORIENTALIS	CHINESE DWARF	NEWT	LIV	350	NO	HK	HK	T	C	C	C	11/26/05	11/26/05	I	LA	DOLPHIN INTERNATIONAL
2005659503	PACB	PACHYTRITON	BREVIPES	TSITOU	NEWT	LIV	100	NO	HK	HK	T	C	C	C	11/26/05	11/26/05	I	LA	DOLPHIN INTERNATIONAL
2005659533	CYOR	CYNOPS	ORIENTALIS	CHINESE DWARF	NEWT	LIV	4,600	NO	CN	CN	T	C	C	C	11/26/05	11/26/05	I	LA	Z IMPORTS INC.
2005659605	CYOR	CYNOPS	ORIENTALIS	CHINESE DWARF	NEWT	LIV	1,450	NO	HK	HK	T	C	C	C	11/26/05	11/27/05	I	LA	DOLPHIN INTERNATIONAL
2005659605	PACB	PACHYTRITON	BREVIPES	TSITOU	NEWT	LIV	100	NO	HK	HK	T	C	C	C	11/26/05	11/27/05	I	LA	DOLPHIN INTERNATIONAL
2005659620	TRIH	TRITURUS	HONGKONGENSIS	ALPINE	NEWT	LIV	80	NO	SG	SG	T	W	C	C	11/26/05	11/27/05	I	LA	AQUACO, INC.
2005659622	TRIH	TRITURUS	HONGKONGENSIS	ALPINE	NEWT	LIV	175	NO	HK	HK	T	W	C	C	11/26/05	11/27/05	I	LA	AQUACO, INC.
2005662887	CYOR	CYNOPS	ORIENTALIS	CHINESE DWARF	NEWT	LIV	250	NO	HK	HK	T	C	C	C	12/3/05	12/3/05	I	LA	DOLPHIN INTERNATIONAL
2005662895	TRIT	TRITURUS	SPECIES		NEWT	LIV	1,000	NO	HK	HK	T	C	C	C	12/3/05	12/3/05	I	LA	GLOBAL AQUATIC CONSULTING
2005662992	CYOR	CYNOPS	ORIENTALIS	CHINESE DWARF	NEWT	LIV	550	NO	HK	HK	T	C	C	C	12/4/05	12/4/05	I	LA	DOLPHIN INTERNATIONAL
2005663001	TRIH	TRITURUS	HONGKONGENSIS	ALPINE	NEWT	LIV	450	NO	HK	HK	T	W	C	C	12/4/05	12/4/05	I	LA	AQUACO, INC.
2005667744	CYOR	CYNOPS	ORIENTALIS	CHINESE DWARF	NEWT	LIV	3,000	NO	CN	CN	T	C	C	C	12/10/05	12/10/05	I	LA	Z IMPORTS INC.
2005667833	CYOR	CYNOPS	ORIENTALIS	CHINESE DWARF	NEWT	LIV	2,100	NO	HK	HK	T	C	C	C	12/11/05	12/11/05	I	LA	DOLPHIN INTERNATIONAL
2005667833	PACB	PACHYTRITON	BREVIPES	TSITOU	NEWT	LIV	500	NO	HK	HK	T	C	C	C	12/11/05	12/11/05	I	LA	DOLPHIN INTERNATIONAL
2005667848	TRIT	TRITURUS	SPECIES		NEWT	LIV	300	NO	HK	HK	T	C	C	C	12/11/05	12/11/05	I	LA	AQUATIC CONNECTION
2005667854	TRIH	TRITURUS	HONGKONGENSIS	ALPINE	NEWT	LIV	475	NO	HK	HK	T	W	C	C	12/11/05	12/11/05	I	LA	AQUACO, INC.
2005667858	TRIH	TRITURUS	HONGKONGENSIS	ALPINE	NEWT	LIV	3,875	NO	SG	SG	T	W	C	C	12/11/05	12/11/05	I	LA	AQUACO, INC.
2005668377	CNP?	CYNOPS	SPECIES	FIREBELLY	NEWT	LIV	600	NO	HK	HK	T	C	C	C	10/25/05	10/25/05	I	SF	GOLDEN PHOENIX FISHERIES INC
2005668884	SASA	SALAMANDRA	SALAMANDRA	EUROPEAN FIRE	SALAMANDER	LIV	375	NO	GB	GB	T	W	C	C	10/12/05	10/12/05	I	MI	TWO AMIGOS IMPORT & EXPORT, INC.

2005668884	TRIT	TRITURUS	SPECIES		NEWT	LIV	10	NO	GB	GB	T	W	C	C	10/12/05	10/12/05	I	MI	TWO AMIGOS IMPORT & EXPORT, INC.
2005670728	CYOR	CYNOPS	ORIENTALIS	CHINESE DWARF	NEWT	LIV	250	NO	HK	HK	T	C	C	C	12/17/05	12/17/05	I	LA	DOLPHIN INTERNATIONAL
2005670737	TRIT	TRITURUS	SPECIES		NEWT	LIV	800	NO	HK	HK	T	C	C	C	12/17/05	12/17/05	I	LA	GLOBAL AQUATIC CONSULTING
2005670818	TRIT	TRITURUS	SPECIES		NEWT	LIV	300	NO	HK	HK	T	C	C	C	12/18/05	12/18/05	I	LA	AQUATIC CONNECTION
2005670819	CYOR	CYNOPS	ORIENTALIS	CHINESE DWARF	NEWT	LIV	920	NO	HK	HK	T	C	C	C	12/18/05	12/18/05	I	LA	DOLPHIN INTERNATIONAL
2005670833	TRIH	TRITURUS	HONGKONGENSIS	ALPINE	NEWT	LIV	125	NO	HK	HK	T	C	C	C	12/18/05	12/18/05	I	LA	AQUACO, INC.
2005672396	SAL?	SALAMANDRA	SPECIES	FIRE	SALAMANDER	LIV	200	NO	SI	SI	T	C	C	C	12/21/05	12/21/05	I	LA	L. A. REPTILE
2005672458	SAL?	SALAMANDRA	SPECIES	FIRE	SALAMANDER	LIV	5	NO	DE	DE	T	C	C	C	11/11/05	11/11/05	I	CH	NAME REMOVED
2005672458	SAL?	SALAMANDRA	SPECIES	FIRE	SALAMANDER	LIV	2	NO	DE	DE	T	C	C	C	11/11/05	11/11/05	I	CH	NAME REMOVED
2005672458	SAL?	SALAMANDRA	SPECIES	FIRE	SALAMANDER	LIV	5	NO	DE	DE	T	C	C	C	11/11/05	11/11/05	I	CH	NAME REMOVED
2005672997	CYPY	CYNOPS	PYRRHOGASTER	JAPANESE FIREBELLY	NEWT	LIV	200	NO	HK	HK	T	W	C	C	11/15/05	11/12/05	I	NY	TRANSSHIP DISCOUNTS , LTD.
2005673113	CYPY	CYNOPS	PYRRHOGASTER	JAPANESE FIREBELLY	NEWT	LIV	110	NO	HK	HK	T	W	C	C	12/6/05	12/3/05	I	NY	TRANSSHIP DISCOUNTS , LTD.
2005673251	CYPY	CYNOPS	PYRRHOGASTER	JAPANESE FIREBELLY	NEWT	LIV	220	NO	HK	HK	T	W	C	C	11/7/05	11/6/05	I	NY	TRANSSHIP DISCOUNTS , LTD.
2005673460	CYPY	CYNOPS	PYRRHOGASTER	JAPANESE FIREBELLY	NEWT	LIV	150	NO	HK	HK	T	W	C	C	11/7/05	11/5/05	I	NY	ERDA INCORPORATED
2005673551	CYPY	CYNOPS	PYRRHOGASTER	JAPANESE FIREBELLY	NEWT	LIV	250	NO	CN	CN	T	C	C	C	11/4/05	10/30/05	I	NY	FISH MART INCORPORATED
2005673883	CYOR	CYNOPS	ORIENTALIS	CHINESE DWARF	NEWT	LIV	2,000	NO	CN	CN	T	C	C	C	12/23/05	12/23/05	I	LA	Z IMPORTS INC.
2005674135	CYOR	CYNOPS	ORIENTALIS	CHINESE DWARF	NEWT	LIV	1,100	NO	HK	HK	T	C	C	C	12/23/05	12/24/05	I	LA	DOLPHIN INTERNATIONAL
2005674156	TRIT	TRITURUS	SPECIES		NEWT	LIV	800	NO	HK	HK	T	C	C	C	12/24/05	12/24/05	I	LA	GLOBAL AQUATIC CONSULTING
2005674181	CYOR	CYNOPS	ORIENTALIS	CHINESE DWARF	NEWT	LIV	500	NO	HK	HK	T	C	C	C	12/25/05	12/25/05	I	LA	DOLPHIN INTERNATIONAL
2005674181	PACB	PACHYTRITON	BREVIPES	TSITOU	NEWT	LIV	100	NO	HK	HK	T	C	C	C	12/25/05	12/25/05	I	LA	DOLPHIN INTERNATIONAL
2005674749	CYPY	CYNOPS	PYRRHOGASTER	JAPANESE FIREBELLY	NEWT	LIV	200	NO	CN	CN	T	C	C	C	10/26/05	10/23/05	I	NY	FISH MART INCORPORATED
2005674752	TRIT	TRITURUS	SPECIES		NEWT	LIV	700	NO	HK	HK	T	W	C	C	10/26/05	9/9/05	I	NY	INTERNATIONAL PET RESOURCES , LLC
2005675785	CYPY	CYNOPS	PYRRHOGASTER	JAPANESE FIREBELLY	NEWT	LIV	50	NO	HK	HK	T	W	C	C	11/1/05	10/30/05	I	NY	TRANSSHIP DISCOUNTS , LTD.
2005675861	CYPY	CYNOPS	PYRRHOGASTER	JAPANESE FIREBELLY	NEWT	LIV	70	NO	HK	HK	T	W	C	C	11/28/05	11/27/05	I	NY	TRANSSHIP DISCOUNTS , LTD.
2005676767	CYOR	CYNOPS	ORIENTALIS	CHINESE DWARF	NEWT	LIV	350	NO	HK	HK	T	C	C	C	12/31/05	12/31/05	I	LA	DOLPHIN INTERNATIONAL
2005676777	TRIT	TRITURUS	SPECIES		NEWT	LIV	1,200	NO	HK	HK	T	C	C	C	12/31/05	12/31/05	I	LA	GLOBAL AQUATIC CONSULTING
2006676822	TRIT	TRITURUS	SPECIES		NEWT	LIV	300	NO	HK	HK	T	C	C	C	1/1/06	1/1/06	I	LA	AQUATIC CONNECTION
2006676827	CYOR	CYNOPS	ORIENTALIS	CHINESE DWARF	NEWT	LIV	2,450	NO	HK	HK	T	C	C	C	1/1/06	1/1/06	I	LA	DOLPHIN INTERNATIONAL
2006676840	TRIH	TRITURUS	HONGKONGENSIS	ALPINE	NEWT	LIV	475	NO	HK	HK	T	W	C	C	1/1/06	1/2/06	I	LA	AQUACO, INC.
2006677171	CYPY	CYNOPS	PYRRHOGASTER	JAPANESE FIREBELLY	NEWT	LIV	80	NO	HK	HK	T	W	C	C	11/23/05	11/20/05	I	NY	TRANSSHIP DISCOUNTS , LTD.
2006677171	PACB	PACHYTRITON	BREVIPES	TSITOU	NEWT	LIV	60	NO	HK	HK	T	W	C	C	11/23/05	11/20/05	I	NY	TRANSSHIP DISCOUNTS , LTD.
2006677172	NEW?	PACHYTRITON	SPECIES	CHINESE	NEWT	LIV	200	NO	CN	CN	T	C	C	C	1/3/06	1/3/06	I	SF	Golden Phoenix Fisheries, USA
2006677186	CYPY	CYNOPS	PYRRHOGASTER	JAPANESE FIREBELLY	NEWT	LIV	150	NO	HK	HK	T	W	C	C	11/23/05	11/19/05	I	NY	ERDA INCORPORATED
2006677249	TRIH	TRITURUS	HONGKONGENSIS	ALPINE	NEWT	LIV	400	NO	HK	HK	T	W	C	C	11/18/05	10/30/05	I	NY	INTERNATIONAL PET RESOURCES , LLC
2006679247	CYOR	CYNOPS	ORIENTALIS	CHINESE DWARF	NEWT	LIV	500	NO	HK	HK	T	C	C	C	1/7/06	1/7/06	I	LA	DOLPHIN INTERNATIONAL
2006679355	CYOR	CYNOPS	ORIENTALIS	CHINESE DWARF	NEWT	LIV	2,400	NO	HK	HK	T	C	C	C	1/8/06	1/8/06	I	LA	DOLPHIN INTERNATIONAL
2006679355	PACB	PACHYTRITON	BREVIPES	TSITOU	NEWT	LIV	300	NO	HK	HK	T	C	C	C	1/8/06	1/8/06	I	LA	DOLPHIN INTERNATIONAL
2006679369	TRIH	TRITURUS	HONGKONGENSIS	ALPINE	NEWT	LIV	70	NO	HK	HK	T	W	C	C	1/8/06	1/8/06	I	LA	AQUACO, INC.
2006679408	TRIT	TRITURUS	SPECIES		NEWT	LIV	30	NO	HK	HK	T	C	C	C	1/8/06	1/8/06	I	LA	NAME REMOVED
2006679802	NEW?	PACHYTRITON	SPECIES	CHINESE	NEWT	LIV	200	NO	CN	CN	T	C	C	C	1/9/06	1/10/06	I	SF	Golden Phoenix Fisheries, USA
2006681546	CYOR	CYNOPS	ORIENTALIS	CHINESE DWARF	NEWT	LIV	3,000	NO	CN	CN	T	C	C	C	1/13/06	1/13/06	I	LA	Z IMPORTS INC.
2006681772	NEW?	PACHYTRITON	SPECIES	CHINESE	NEWT	LIV	100	NO	HK	HK	T	C	C	C	1/14/06	1/14/06	I	LA	DOLPHIN INTERNATIONAL
2006681799	TRIT	TRITURUS	SPECIES		NEWT	LIV	800	NO	HK	HK	T	C	C	C	1/14/06	1/14/06	I	LA	GLOBAL AQUATIC CONSULTING
2006681867	CYOR	CYNOPS	ORIENTALIS	CHINESE DWARF	NEWT	LIV	2,100	NO	HK	HK	T	C	C	C	1/14/06	1/15/06	I	LA	DOLPHIN INTERNATIONAL
2006681867	PACB	PACHYTRITON	BREVIPES	TSITOU	NEWT	LIV	200	NO	HK	HK	T	C	C	C	1/14/06	1/15/06	I	LA	DOLPHIN INTERNATIONAL
2006681908	TRIT	TRITURUS	SPECIES		NEWT	LIV	600	NO	HK	HK	T	C	C	C	1/15/06	1/15/06	I	LA	AQUATIC CONNECTION
2006682900	SASA	SALAMANDRA	SALAMANDRA	EUROPEAN FIRE	SALAMANDER	LIV	500	NO	SI	SI	T	D	C	C	10/19/05	10/14/05	I	MI	GLOBAL TROPICAL IMP/EXP
2006683455	BODO	BOLITOGLOSSA	DOFLEINI	ALTA VERAPAZ	SALAMANDER	LIV	10	NO	GT	GT	T	W	C	C	10/6/05	10/6/05	I	MI	DHAR TROPICAL LLC
2006684361	BODO	BOLITOGLOSSA	DOFLEINI	ALTA VERAPAZ	SALAMANDER	LIV	6	NO	GT	GT	T	W	C	C	6/30/05	6/30/05	I	MI	DHAR TROPICAL LLC
2006685073	BODO	BOLITOGLOSSA	DOFLEINI	ALTA VERAPAZ	SALAMANDER	LIV	13	NO	HN	HN	T	W	C	C	11/9/05	11/8/05	I	MI	STRICTLY REPTILES, INC.
2006685458	CYOR	CYNOPS	ORIENTALIS	CHINESE DWARF	NEWT	LIV	3,000	NO	CN	CN	T	C	C	C	1/20/06	1/20/06	I	LA	Z IMPORTS INC.
2006685824	CYOR	CYNOPS	ORIENTALIS	CHINESE DWARF	NEWT	LIV	600	NO	HK	HK	T	C	C	C	1/21/06	1/21/06	I	LA	DOLPHIN INTERNATIONAL
2006685824	PACB	PACHYTRITON	BREVIPES	TSITOU	NEWT	LIV	100	NO	HK	HK	T	C	C	C	1/21/06	1/21/06	I	LA	DOLPHIN INTERNATIONAL
2006685828	TRIT	TRITURUS	SPECIES		NEWT	LIV	700	NO	HK	HK	T	C	C	C	1/21/06	1/21/06	I	LA	GLOBAL AQUATIC CONSULTING
2006685932	TRIT	TRITURUS	SPECIES		NEWT	LIV	60	NO	HK	HK	T	C	C	C	1/22/06	1/22/06	I	LA	NAME REMOVED
2006685959	TRIH	TRITURUS	HONGKONGENSIS	ALPINE	NEWT	LIV	200	NO	HK	HK	T	W	C	C	1/22/06	1/22/06	I	LA	AQUACO, INC.
2006685991	SAL?	SALAMANDRA	SPECIES	FIRE	SALAMANDER	LIV	281	NO	GB	GB	T	W	C	C	11/16/05	11/15/05	I	MI	TWO AMIGOS IMPORTS, INC.
2006685991	TRIT	TRITURUS	SPECIES		NEWT	LIV	50	NO	GB	GB	T	W	C	C	11/16/05	11/15/05	I	MI	TWO AMIGOS IMPORTS, INC.
2006685991	SAL?	SALAMANDRA	SPECIES	FIRE	SALAMANDER	LIV	20	NO	GB	GB	T	W	C	C	11/16/05	11/15/05	I	MI	TWO AMIGOS IMPORTS, INC.
2006686239	CYOR	CYNOPS	ORIENTALIS	CHINESE DWARF	NEWT	LIV	250	NO	HK	HK	T	C	C	C	1/23/06	1/23/06	I	LA	DOLPHIN INTERNATIONAL

2006687947	SASA	SALAMANDRA	SALAMANDRA	EUROPEAN FIRE	SALAMANDER	LIV	20	NO	DE	DE	T	C	C	C	1/25/06	1/25/06	I	DF	U.S. GLOBAL EXOTICS, INC.
2006687947	TCRI	TRITURUS	CRISTATUS	NORTHERN CRESTED	NEWT	LIV	23	NO	RU	DE	T	W	C	C	1/25/06	1/25/06	I	DF	U.S. GLOBAL EXOTICS, INC.
2006687947	TRIT	TRITURUS	SPECIES		NEWT	LIV	67	NO	RU	DE	T	W	C	C	1/25/06	1/25/06	I	DF	U.S. GLOBAL EXOTICS, INC.
2006689321	BODO	BOLITOGLOSSA	DOFLEINI	ALTA VERAPAZ	SALAMANDER	LIV	34	NO	GT	GT	T	W	C	C	11/3/05	11/3/05	I	MI	DHAR TROPICAL LLC
2006689321	BMEX	BOLITOGLOSSA	MEXICANA	MEX MUSHROOMTONGUE	SALAMANDER	LIV	2	NO	GT	GT	T	W	C	C	11/3/05	11/3/05	I	MI	DHAR TROPICAL LLC
2006690768	TRIT	TRITURUS	SPECIES		NEWT	LIV	800	NO	HK	HK	T	C	C	C	1/28/06	1/28/06	I	LA	GLOBAL AQUATIC CONSULTING
2006690776	TRIT	TRITURUS	SPECIES		NEWT	LIV	300	NO	HK	HK	T	C	C	C	1/28/06	1/28/06	I	LA	AQUATIC CONNECTION
2006690791	CYOR	CYNOPS	ORIENTALIS	CHINESE DWARF	NEWT	LIV	500	NO	HK	HK	T	C	C	C	1/28/06	1/28/06	I	LA	DOLPHIN INTERNATIONAL
2006690847	CYOR	CYNOPS	ORIENTALIS	CHINESE DWARF	NEWT	LIV	950	NO	HK	HK	T	C	C	C	1/28/06	1/29/06	I	LA	DOLPHIN INTERNATIONAL
2006690847	PACB	PACHYTRITON	BREVIPES	TSITOU	NEWT	LIV	200	NO	HK	HK	T	C	C	C	1/28/06	1/29/06	I	LA	DOLPHIN INTERNATIONAL
2006690859	TRIH	TRITURUS	HONGKONGENSIS	ALPINE	NEWT	LIV	325	NO	HK	HK	T	W	C	C	1/28/06	1/29/06	I	LA	AQUACO, INC.
2006692451	SASA	SALAMANDRA	SALAMANDRA	EUROPEAN FIRE	SALAMANDER	LIV	200	NO	GB	GB	T	W	C	C	2/1/06	2/1/06	I	MI	TWO AMIGOS IMPORT & EXPORT, INC.
2006693592	TRIT	TRITURUS	SPECIES		NEWT	LIV	400	NO	HK	HK	T	C	C	C	2/4/06	2/4/06	I	LA	GLOBAL AQUATIC CONSULTING
2006693607	CYOR	CYNOPS	ORIENTALIS	CHINESE DWARF	NEWT	LIV	500	NO	HK	HK	T	C	C	C	2/4/06	2/4/06	I	LA	DOLPHIN INTERNATIONAL
2006693662	TRIH	TRITURUS	HONGKONGENSIS	ALPINE	NEWT	LIV	425	NO	HK	HK	T	W	C	C	2/5/06	2/5/06	I	LA	AQUACO, INC.
2006693680	CYOR	CYNOPS	ORIENTALIS	CHINESE DWARF	NEWT	LIV	2,550	NO	HK	HK	T	C	C	C	2/5/06	2/5/06	I	LA	DOLPHIN INTERNATIONAL
2006693680	PACB	PACHYTRITON	BREVIPES	TSITOU	NEWT	LIV	100	NO	HK	HK	T	C	C	C	2/5/06	2/5/06	I	LA	DOLPHIN INTERNATIONAL
2006693695	TRIT	TRITURUS	SPECIES		NEWT	LIV	300	NO	HK	HK	T	C	C	C	2/5/06	2/5/06	I	LA	AQUATIC CONNECTION
2006695048	BODO	BOLITOGLOSSA	DOFLEINI	ALTA VERAPAZ	SALAMANDER	LIV	10	NO	HN	HN	T	W	C	C	9/13/05	9/13/05	I	MI	STRICTLY REPTILES, INC.
2006696314	PSHG	PACHYHYNOBIUS	SHANGCHENGENSIS	SHANGCHENG	SALAMANDER	LIV	20	NO	CN	HK	T	W	C	C	2/9/06	2/9/06	I	DN	Bushmaster Reptiles Inc
2006696314	CNP?	CYNOPS	SPECIES	FIREBELLY	NEWT	LIV	55	NO	CN	HK	T	W	C	C	2/9/06	2/9/06	I	DN	Bushmaster Reptiles Inc
2006697468	CYOR	CYNOPS	ORIENTALIS	CHINESE DWARF	NEWT	LIV	250	NO	HK	HK	T	C	C	C	2/11/06	2/11/06	I	LA	DOLPHIN INTERNATIONAL
2006697534	CYOR	CYNOPS	ORIENTALIS	CHINESE DWARF	NEWT	LIV	1,700	NO	HK	HK	T	C	C	C	2/11/06	2/12/06	I	LA	DOLPHIN INTERNATIONAL
2006697548	TRIH	TRITURUS	HONGKONGENSIS	ALPINE	NEWT	LIV	200	NO	HK	HK	T	W	C	C	2/11/06	2/12/06	I	LA	AQUACO, INC.
2006700568	PLAB	PACHYTRITON	LABIATUS	UNTERSTEIN'S	NEWT	LIV	25	NO	CN	HK	T	W	C	C	2/16/06	2/15/06	I	DN	Bushmaster Reptiles Inc
2006700568	PSHG	PACHYHYNOBIUS	SHANGCHENGENSIS	SHANGCHENG	SALAMANDER	LIV	110	NO	CN	HK	T	W	C	C	2/16/06	2/15/06	I	DN	Bushmaster Reptiles Inc
2006700568	CNP?	CYNOPS	SPECIES	FIREBELLY	NEWT	LIV	100	NO	CN	HK	T	W	C	C	2/16/06	2/15/06	I	DN	Bushmaster Reptiles Inc
2006701238	CYOR	CYNOPS	ORIENTALIS	CHINESE DWARF	NEWT	LIV	750	NO	HK	HK	T	C	C	C	2/18/06	2/18/06	I	LA	DOLPHIN INTERNATIONAL
2006701356	CYOR	CYNOPS	ORIENTALIS	CHINESE DWARF	NEWT	LIV	1,450	NO	HK	HK	T	C	C	C	2/19/06	2/19/06	I	LA	DOLPHIN INTERNATIONAL
2006701382	TRIH	TRITURUS	HONGKONGENSIS	ALPINE	NEWT	LIV	250	NO	HK	HK	T	W	C	C	2/19/06	2/19/06	I	LA	AQUACO, INC.
2006702536	PSHG	PACHYHYNOBIUS	SHANGCHENGENSIS	SHANGCHENG	SALAMANDER	LIV	50	NO	HK	HK	T	W	C	C	2/22/06	1/18/06	I	MI	TWO AMIGOS IMPORT & EXPORT, INC.
2006702536	PCHI	PARAMESOTRITON	CHINENSIS	CHINESE WARTY	NEWT	LIV	89	NO	HK	HK	T	W	C	C	2/22/06	1/18/06	I	MI	TWO AMIGOS IMPORT & EXPORT, INC.
2006702536	PLAB	PACHYTRITON	LABIATUS	UNTERSTEIN'S	NEWT	LIV	100	NO	HK	HK	T	W	C	C	2/22/06	1/18/06	I	MI	TWO AMIGOS IMPORT & EXPORT, INC.
2006702536	PACB	PACHYTRITON	BREVIPES	TSITOU	NEWT	LIV	50	NO	HK	HK	T	W	C	C	2/22/06	1/18/06	I	MI	TWO AMIGOS IMPORT & EXPORT, INC.
2006702536	NEW?	PACHYTRITON	SPECIES	CHINESE	NEWT	LIV	12	NO	HK	HK	T	W	C	C	2/22/06	1/18/06	I	MI	TWO AMIGOS IMPORT & EXPORT, INC.
2006702536	CNP?	CYNOPS	SPECIES	FIREBELLY	NEWT	LIV	50	NO	HK	HK	T	W	C	C	2/22/06	1/18/06	I	MI	TWO AMIGOS IMPORT & EXPORT, INC.
2006702536	CYOR	CYNOPS	ORIENTALIS	CHINESE DWARF	NEWT	LIV	500	NO	HK	HK	T	W	C	C	2/22/06	1/18/06	I	MI	TWO AMIGOS IMPORT & EXPORT, INC.
2006703525	CYOR	CYNOPS	ORIENTALIS	CHINESE DWARF	NEWT	LIV	3,000	NO	CN	CN	T	W	C	C	2/24/06	2/24/06	I	LA	Z IMPORTS INC.
2006703858	CYOR	CYNOPS	ORIENTALIS	CHINESE DWARF	NEWT	LIV	500	NO	HK	HK	T	C	C	C	2/25/06	2/25/06	I	LA	DOLPHIN INTERNATIONAL
2006703874	TRIT	TRITURUS	SPECIES		NEWT	LIV	1,400	NO	HK	HK	T	C	C	C	2/25/06	2/25/06	I	LA	GLOBAL AQUATIC CONSULTING
2006703947	CYOR	CYNOPS	ORIENTALIS	CHINESE DWARF	NEWT	LIV	2,750	NO	HK	HK	T	C	C	C	2/26/06	2/26/06	I	LA	DOLPHIN INTERNATIONAL
2006703947	PACB	PACHYTRITON	BREVIPES	TSITOU	NEWT	LIV	300	NO	HK	HK	T	C	C	C	2/26/06	2/26/06	I	LA	DOLPHIN INTERNATIONAL
2006703964	TRIH	TRITURUS	HONGKONGENSIS	ALPINE	NEWT	LIV	425	NO	HK	HK	T	W	C	C	2/26/06	2/26/06	I	LA	AQUACO, INC.
2006703990	TRIT	TRITURUS	SPECIES		NEWT	LIV	100	NO	HK	HK	T	C	C	C	2/26/06	2/26/06	I	LA	AQUATIC CONNECTION
2006704263	SASA	SALAMANDRA	SALAMANDRA	EUROPEAN FIRE	SALAMANDER	LIV	4	NO	DE	DE	P	C	R	S	2/24/06	2/24/06	I	CH	Exemptions 6 and 7(C)
2006707095	CYOR	CYNOPS	ORIENTALIS	CHINESE DWARF	NEWT	LIV	2,000	NO	CN	CN	T	W	C	C	3/3/06	3/3/06	I	LA	Z IMPORTS INC.
2006707453	TRIT	TRITURUS	SPECIES		NEWT	LIV	600	NO	HK	HK	T	C	C	C	3/4/06	3/4/06	I	LA	GLOBAL AQUATIC CONSULTING
2006707526	CYOR	CYNOPS	ORIENTALIS	CHINESE DWARF	NEWT	LIV	1,500	NO	HK	HK	T	C	C	C	3/5/06	3/5/06	I	LA	DOLPHIN INTERNATIONAL
2006707526	PACB	PACHYTRITON	BREVIPES	TSITOU	NEWT	LIV	100	NO	HK	HK	T	C	C	C	3/5/06	3/5/06	I	LA	DOLPHIN INTERNATIONAL
2006707566	TRIT	TRITURUS	SPECIES		NEWT	LIV	300	NO	HK	HK	T	C	C	C	3/5/06	3/5/06	I	LA	AQUATIC CONNECTION
2006707569	TRIH	TRITURUS	HONGKONGENSIS	ALPINE	NEWT	LIV	500	NO	HK	HK	T	W	C	C	3/5/06	3/5/06	I	LA	AQUACO, INC.
2006710081	TRIT	TRITURUS	SPECIES		NEWT	LIV	1,200	NO	HK	HK	T	C	C	C	3/11/06	3/11/06	I	LA	GLOBAL AQUATIC CONSULTING
2006710085	CYOR	CYNOPS	ORIENTALIS	CHINESE DWARF	NEWT	LIV	250	NO	HK	HK	T	C	C	C	3/11/06	3/11/06	I	LA	DOLPHIN INTERNATIONAL
2006710141	CYOR	CYNOPS	ORIENTALIS	CHINESE DWARF	NEWT	LIV	1,850	NO	HK	HK	T	C	C	C	3/12/06	3/12/06	I	LA	DOLPHIN INTERNATIONAL
2006710141	PACB	PACHYTRITON	BREVIPES	TSITOU	NEWT	LIV	100	NO	HK	HK	T	C	C	C	3/12/06	3/12/06	I	LA	DOLPHIN INTERNATIONAL
2006710169	TRIH	TRITURUS	HONGKONGENSIS	ALPINE	NEWT	LIV	50	NO	HK	HK	T	W	C	C	3/12/06	3/12/06	I	LA	AQUACO, INC.
2006710180	TRIT	TRITURUS	SPECIES		NEWT	LIV	200	NO	HK	HK	T	C	C	C	3/12/06	3/12/06	I	LA	AQUATIC CONNECTION
2006710972	NEW?	PACHYTRITON	SPECIES	CHINESE	NEWT	LIV	250	NO	CN	CN	T	C	C	C	3/14/06	3/14/06	I	SF	Golden Phoenix Fisheries, USA
2006713281	PLAB	PACHYTRITON	LABIATUS	UNTERSTEIN'S	NEWT	LIV	504	NO	CN	HK	T	W	C	C	3/16/06	3/16/06	I	DN	Bushmaster Reptiles Inc
2006713281	CNP?	CYNOPS	SPECIES	FIREBELLY	NEWT	LIV	1,116	NO	CN	HK	T	W	C	C	3/16/06	3/16/06	I	DN	Bushmaster Reptiles Inc

2006713281	PSHG	PACHYHYNOBIUS	SHANGCHENGENSIS	SHANGCHENG	SALAMANDER	LIV	100	NO	CN	HK	T	W	C	C	3/16/06	3/16/06	I	DN	Bushmaster Reptiles Inc
2006713281	PCHI	PARAMESOTRITON	CHINENSIS	CHINESE WARTY	NEWT	LIV	200	NO	CN	HK	T	W	C	C	3/16/06	3/16/06	I	DN	Bushmaster Reptiles Inc
2006714066	CYOR	CYNOPS	ORIENTALIS	CHINESE DWARF	NEWT	LIV	250	NO	HK	HK	T	C	C	C	3/18/06	3/18/06	I	LA	DOLPHIN INTERNATIONAL
2006714100	TRIT	TRITURUS	SPECIES		NEWT	LIV	1,000	NO	HK	HK	T	C	C	C	3/18/06	3/18/06	I	LA	GLOBAL AQUATIC CONSULTING
2006714401	TRIH	TRITURUS	HONGKONGENSIS	ALPINE	NEWT	LIV	650	NO	HK	HK	T	W	C	C	3/20/06	3/19/06	I	LA	AQUACO, INC.
2006714450	CYOR	CYNOPS	ORIENTALIS	CHINESE DWARF	NEWT	LIV	2,250	NO	HK	HK	T	C	C	C	3/20/06	3/19/06	I	LA	DOLPHIN INTERNATIONAL
2006714450	PACB	PACHYTRITON	BREVIPES	TSITOU	NEWT	LIV	100	NO	HK	HK	T	C	C	C	3/20/06	3/19/06	I	LA	DOLPHIN INTERNATIONAL
2006714484	TRIT	TRITURUS	SPECIES		NEWT	LIV	300	NO	HK	HK	T	C	C	C	3/20/06	3/19/06	I	LA	AQUATIC CONNECTION
2006715803	CNP?	CYNOPS	SPECIES	FIREBELLY	NEWT	LIV	350	NO	HK	HK	T	W	C	C	2/13/06	2/11/06	I	NY	ERDA INCORPORATED
2006715952	CYPY	CYNOPS	PYRRHOGASTER	JAPANESE FIREBELLY	NEWT	LIV	100	NO	CN	CN	T	C	C	C	2/1/06	1/27/06	I	NY	FISH MART INCORPORATED
2006715952	CNP?	CYNOPS	SPECIES	FIREBELLY	NEWT	LIV	75	NO	CN	CN	T	C	C	C	2/1/06	1/27/06	I	NY	FISH MART INCORPORATED
2006716512	CYOR	CYNOPS	ORIENTALIS	CHINESE DWARF	NEWT	LIV	100	NO	SG	SG	T	U	C	C	1/26/06	12/24/05	I	NY	INTERNATIONAL PET RESOURCES , LLC
2006716859	CYPY	CYNOPS	PYRRHOGASTER	JAPANESE FIREBELLY	NEWT	LIV	170	NO	HK	HK	T	W	C	C	1/17/06	1/15/06	I	NY	TRANSSHIP DISCOUNTS , LTD.
2006716859	PACB	PACHYTRITON	BREVIPES	TSITOU	NEWT	LIV	60	NO	HK	HK	T	W	C	C	1/17/06	1/15/06	I	NY	TRANSSHIP DISCOUNTS , LTD.
2006716960	CYPY	CYNOPS	PYRRHOGASTER	JAPANESE FIREBELLY	NEWT	LIV	525	NO	HK	HK	T	W	C	C	3/2/06	2/25/06	I	NY	ERDA INCORPORATED
2006717653	CYPY	CYNOPS	PYRRHOGASTER	JAPANESE FIREBELLY	NEWT	LIV	260	NO	HK	HK	T	W	C	C	1/1/06	12/23/05	I	NY	TRANSSHIP DISCOUNTS , LTD.
2006717828	CYPY	CYNOPS	PYRRHOGASTER	JAPANESE FIREBELLY	NEWT	LIV	70	NO	HK	HK	T	W	C	C	1/9/06	1/8/06	I	NY	TRANSSHIP DISCOUNTS , LTD.
2006717861	CYOR	CYNOPS	ORIENTALIS	CHINESE DWARF	NEWT	LIV	3,000	NO	CN	CN	T	C	C	C	3/24/06	3/24/06	I	LA	Z IMPORTS INC.
2006717966	PLET	PLETHODON	SPECIES		SALAMANDER	LIV	100	NO	US	DE	S	W	C	C	10/20/05	10/12/05	I	PT	OREGON STATE UNIVERSITY
2006718378	CYOR	CYNOPS	ORIENTALIS	CHINESE DWARF	NEWT	LIV	500	NO	HK	HK	T	C	C	C	3/25/06	3/25/06	I	LA	DOLPHIN INTERNATIONAL
2006718416	TRIT	TRITURUS	SPECIES		NEWT	LIV	600	NO	HK	HK	T	C	C	C	3/25/06	3/25/06	I	LA	GLOBAL AQUATIC CONSULTING
2006718479	TRIH	TRITURUS	HONGKONGENSIS	ALPINE	NEWT	LIV	575	NO	HK	HK	T	W	C	C	3/25/06	3/26/06	I	LA	AQUACO, INC.
2006718502	CYOR	CYNOPS	ORIENTALIS	CHINESE DWARF	NEWT	LIV	2,350	NO	HK	HK	T	C	C	C	3/25/06	3/26/06	I	LA	DOLPHIN INTERNATIONAL
2006718502	PACB	PACHYTRITON	BREVIPES	TSITOU	NEWT	LIV	100	NO	HK	HK	T	C	C	C	3/25/06	3/26/06	I	LA	DOLPHIN INTERNATIONAL
2006718631	CYPY	CYNOPS	PYRRHOGASTER	JAPANESE FIREBELLY	NEWT	LIV	225	NO	HK	HK	T	W	C	C	1/17/06	1/14/06	I	NY	ERDA INCORPORATED
2006718885	CYPY	CYNOPS	PYRRHOGASTER	JAPANESE FIREBELLY	NEWT	LIV	100	NO	CN	CN	T	C	C	C	1/9/06	1/1/06	I	NY	FISH MART INCORPORATED
2006718885	CNP?	CYNOPS	SPECIES	FIREBELLY	NEWT	LIV	50	NO	CN	CN	T	C	C	C	1/9/06	1/1/06	I	NY	FISH MART INCORPORATED
2006718993	CYPY	CYNOPS	PYRRHOGASTER	JAPANESE FIREBELLY	NEWT	LIV	600	NO	HK	HK	T	W	C	C	1/10/06	1/7/06	I	NY	ERDA INCORPORATED
2006719413	CYPY	CYNOPS	PYRRHOGASTER	JAPANESE FIREBELLY	NEWT	LIV	525	NO	HK	HK	T	W	C	C	12/13/05	12/10/05	I	NY	ERDA INCORPORATED
2006719926	CYPY	CYNOPS	PYRRHOGASTER	JAPANESE FIREBELLY	NEWT	LIV	50	NO	HK	HK	T	W	C	C	12/13/05	12/10/05	I	NY	TRANSSHIP DISCOUNTS , LTD.
2006720381	CYOR	CYNOPS	ORIENTALIS	CHINESE DWARF	NEWT	LIV	200	NO	CN	CN	T	F	C	C	12/7/05	11/30/05	I	NY	MIN HUA ENTERPRISE, INC.
2006720382	CYPY	CYNOPS	PYRRHOGASTER	JAPANESE FIREBELLY	NEWT	LIV	30	NO	HK	HK	T	C	C	C	10/6/05	10/1/05	I	NY	ERDA INCORPORATED
2006720641	CYPY	CYNOPS	PYRRHOGASTER	JAPANESE FIREBELLY	NEWT	LIV	300	NO	HK	HK	T	C	C	C	1/3/06	12/29/05	I	NY	ERDA INCORPORATED
2006720779	HYN?	HYNOBIUS	SPECIES		SALAMANDER	LIV	4	NO	KE	KE	E	C	C	C	10/17/05	10/15/05	I	NY	AMERICAN MUSEUM OF NATURAL HISTORY
2006720823	CYPY	CYNOPS	PYRRHOGASTER	JAPANESE FIREBELLY	NEWT	LIV	430	NO	HK	HK	T	W	C	C	10/14/05	10/9/05	I	NY	TRANSSHIP DISCOUNTS LIMITED
2006721093	PACB	PACHYTRITON	BREVIPES	TSITOU	NEWT	LIV	30	NO	HK	HK	T	W	C	C	1/3/06	12/30/05	I	NY	TRANSSHIP DISCOUNTS LIMITED
2006721344	CYPY	CYNOPS	PYRRHOGASTER	JAPANESE FIREBELLY	NEWT	LIV	150	NO	HK	HK	T	W	C	C	1/25/06	1/22/06	I	NY	TRANSSHIP DISCOUNTS LIMITED
2006721801	CYPY	CYNOPS	PYRRHOGASTER	JAPANESE FIREBELLY	NEWT	LIV	100	NO	CN	CN	T	C	C	C	12/23/05	12/18/05	I	NY	FISH MART INCORPORATED
2006721801	CNP?	CYNOPS	SPECIES	FIREBELLY	NEWT	LIV	50	NO	CN	CN	T	C	C	C	12/23/05	12/18/05	I	NY	FISH MART INCORPORATED
2006722404	TRIT	TRITURUS	SPECIES		NEWT	LIV	600	NO	HK	HK	T	C	C	C	4/1/06	4/1/06	I	LA	GLOBAL AQUATIC CONSULTING
2006722437	TRIH	TRITURUS	HONGKONGENSIS	ALPINE	NEWT	LIV	25	NO	HK	HK	T	W	C	C	4/2/06	4/2/06	I	LA	AQUACO, INC.
2006722453	CYOR	CYNOPS	ORIENTALIS	CHINESE DWARF	NEWT	LIV	3,000	NO	HK	HK	T	C	C	C	4/2/06	4/2/06	I	LA	DOLPHIN INTERNATIONAL
2006722453	PACB	PACHYTRITON	BREVIPES	TSITOU	NEWT	LIV	200	NO	HK	HK	T	C	C	C	4/2/06	4/2/06	I	LA	DOLPHIN INTERNATIONAL
2006723301	PACB	PACHYTRITON	BREVIPES	TSITOU	NEWT	LIV	30	NO	HK	HK	T	W	C	C	12/20/05	12/18/05	I	NY	TRANSSHIP DISCOUNTS LIMITED
2006724011	CYPY	CYNOPS	PYRRHOGASTER	JAPANESE FIREBELLY	NEWT	LIV	650	NO	HK	HK	T	C	C	C	12/20/05	12/17/05	I	NY	ERDA INCORPORATED
2006726839	CYOR	CYNOPS	ORIENTALIS	CHINESE DWARF	NEWT	LIV	250	NO	HK	HK	T	C	C	C	4/8/06	4/8/06	I	LA	DOLPHIN INTERNATIONAL
2006726855	TRIT	TRITURUS	SPECIES		NEWT	LIV	400	NO	HK	HK	T	C	C	C	4/8/06	4/8/06	I	LA	GLOBAL AQUATIC CONSULTING
2006726908	CYOR	CYNOPS	ORIENTALIS	CHINESE DWARF	NEWT	LIV	2,350	NO	HK	HK	T	C	C	C	4/9/06	4/9/06	I	LA	DOLPHIN INTERNATIONAL
2006726908	PACB	PACHYTRITON	BREVIPES	TSITOU	NEWT	LIV	200	NO	HK	HK	T	C	C	C	4/9/06	4/9/06	I	LA	DOLPHIN INTERNATIONAL
2006726927	TRIH	TRITURUS	HONGKONGENSIS	ALPINE	NEWT	LIV	200	NO	HK	HK	T	W	C	C	4/9/06	4/9/06	I	LA	AQUACO, INC.
2006729115	CYOR	CYNOPS	ORIENTALIS	CHINESE DWARF	NEWT	LIV	4,000	NO	CN	CN	T	C	C	C	4/14/06	4/14/06	I	LA	Z IMPORTS INC.
2006729608	TRIH	TRITURUS	HONGKONGENSIS	ALPINE	NEWT	LIV	200	NO	HK	HK	T	W	C	C	4/17/06	4/16/06	I	LA	AQUACO, INC.
2006729642	CYOR	CYNOPS	ORIENTALIS	CHINESE DWARF	NEWT	LIV	1,500	NO	HK	HK	T	C	C	C	4/17/06	4/16/06	I	LA	DOLPHIN INTERNATIONAL
2006729642	PACB	PACHYTRITON	BREVIPES	TSITOU	NEWT	LIV	200	NO	HK	HK	T	C	C	C	4/17/06	4/16/06	I	LA	DOLPHIN INTERNATIONAL
2006729665	CYOR	CYNOPS	ORIENTALIS	CHINESE DWARF	NEWT	LIV	250	NO	HK	HK	T	C	C	C	4/17/06	4/15/06	I	LA	DOLPHIN INTERNATIONAL
2006730140	TRIT	TRITURUS	SPECIES		NEWT	LIV	800	NO	HK	HK	T	C	C	C	4/18/06	4/15/06	I	LA	GLOBAL AQUATIC CONSULTING
2006731777	TRIT	TRITURUS	SPECIES		NEWT	LIV	800	NO	HK	HK	T	C	C	C	4/22/06	4/22/06	I	LA	GLOBAL AQUATIC CONSULTING
2006731865	TRIH	TRITURUS	HONGKONGENSIS	ALPINE	NEWT	LIV	400	NO	HK	PE	T	W	C	C	4/23/06	4/23/06	I	LA	AQUACO, INC.
2006731884	CYOR	CYNOPS	ORIENTALIS	CHINESE DWARF	NEWT	LIV	1,750	NO	HK	HK	T	C	C	C	4/23/06	4/23/06	I	LA	DOLPHIN INTERNATIONAL

2006731884	PACB	PACHYTRITON	BREVIPES	TSITOU	NEWT	LIV	200	NO	HK	HK	T	C	C	C	4/23/06	4/23/06	I	LA	DOLPHIN INTERNATIONAL
2006731890	TRIT	TRITURUS	SPECIES		NEWT	LIV	100	NO	HK	HK	T	C	C	C	4/23/06	4/23/06	I	LA	AQUATIC CONNECTION
2006733800	CYOR	CYNOPS	ORIENTALIS	CHINESE DWARF	NEWT	LIV	2,500	NO	CN	CN	T	W	C	C	4/28/06	4/28/06	I	LA	Z IMPORTS INC.
2006734080	CYOR	CYNOPS	ORIENTALIS	CHINESE DWARF	NEWT	LIV	750	NO	HK	HK	T	C	C	C	4/29/06	4/29/06	I	LA	DOLPHIN INTERNATIONAL
2006734080	PACB	PACHYTRITON	BREVIPES	TSITOU	NEWT	LIV	200	NO	HK	HK	T	C	C	C	4/29/06	4/29/06	I	LA	DOLPHIN INTERNATIONAL
2006734101	TRIT	TRITURUS	SPECIES		NEWT	LIV	400	NO	HK	HK	T	C	C	C	4/29/06	4/29/06	I	LA	GLOBAL AQUATIC CONSULTING
2006734166	TRIH	TRITURUS	HONGKONGENSIS	ALPINE	NEWT	LIV	150	NO	HK	HK	T	W	C	C	4/29/06	4/30/06	I	LA	AQUACO, INC.
2006734170	CYOR	CYNOPS	ORIENTALIS	CHINESE DWARF	NEWT	LIV	1,700	NO	HK	HK	T	C	C	C	4/29/06	4/30/06	I	LA	DOLPHIN INTERNATIONAL
2006734170	PACB	PACHYTRITON	BREVIPES	TSITOU	NEWT	LIV	100	NO	HK	HK	T	C	C	C	4/29/06	4/30/06	I	LA	DOLPHIN INTERNATIONAL
2006735472	PLAB	PACHYTRITON	LABIATUS	UNTERSTEIN'S	NEWT	LIV	200	NO	CN	HK	T	W	C	C	5/3/06	5/3/06	I	DN	Bushmaster Reptiles Inc
2006735472	CNP?	CYNOPS	SPECIES	FIREBELLY	NEWT	LIV	79	NO	CN	HK	T	W	C	C	5/3/06	5/3/06	I	DN	Bushmaster Reptiles Inc
2006735472	CNP?	CYNOPS	SPECIES	FIREBELLY	NEWT	LIV	1,000	NO	CN	HK	T	W	C	C	5/3/06	5/3/06	I	DN	Bushmaster Reptiles Inc
2006735472	PSHG	PACHYHYNOBIUS	SHANGCHENGENSIS	SHANGCHENG	SALAMANDER	LIV	51	NO	CN	HK	T	W	C	C	5/3/06	5/3/06	I	DN	Bushmaster Reptiles Inc
2006735472	PCHI	PARAMESOTRITON	CHINENSIS	CHINESE WARTY	NEWT	LIV	200	NO	CN	HK	T	W	C	C	5/3/06	5/3/06	I	DN	Bushmaster Reptiles Inc
2006736631	CYOR	CYNOPS	ORIENTALIS	CHINESE DWARF	NEWT	LIV	250	NO	HK	HK	T	C	C	C	5/6/06	5/6/06	I	LA	DOLPHIN INTERNATIONAL
2006736652	TRIT	TRITURUS	SPECIES		NEWT	LIV	400	NO	HK	HK	T	C	C	C	5/6/06	5/6/06	I	LA	GLOBAL AQUATIC CONSULTING
2006736721	CYOR	CYNOPS	ORIENTALIS	CHINESE DWARF	NEWT	LIV	3,550	NO	HK	HK	T	C	C	C	5/7/06	5/7/06	I	LA	DOLPHIN INTERNATIONAL
2006736721	PACB	PACHYTRITON	BREVIPES	TSITOU	NEWT	LIV	300	NO	HK	HK	T	C	C	C	5/7/06	5/7/06	I	LA	DOLPHIN INTERNATIONAL
2006736722	TRIH	TRITURUS	HONGKONGENSIS	ALPINE	NEWT	LIV	200	NO	HK	HK	T	W	C	C	5/7/06	5/7/06	I	LA	AQUACO, INC.
2006736727	TRIT	TRITURUS	SPECIES		NEWT	LIV	200	NO	HK	HK	T	C	C	C	5/7/06	5/7/06	I	LA	AQUATIC CONNECTION
2006736848	TRIT	TRITURUS	SPECIES		NEWT	LIV	500	NO	HK	HK	T	C	C	C	5/8/06	5/8/06	I	AT	SUN PET LTD.
2006739026	SASA	SALAMANDRA	SALAMANDRA	EUROPEAN FIRE	SALAMANDER	LIV	400	NO	SI	SI	T	W	C	C	5/11/06	5/11/06	I	LA	L. A. REPTILE
2006739069	CYOR	CYNOPS	ORIENTALIS	CHINESE DWARF	NEWT	LIV	2,000	NO	CN	CN	T	C	C	C	5/11/06	5/11/06	I	LA	Z IMPORTS INC.
2006740165	CYOR	CYNOPS	ORIENTALIS	CHINESE DWARF	NEWT	LIV	1,000	NO	HK	HK	T	C	C	C	5/13/06	5/13/06	I	LA	DOLPHIN INTERNATIONAL
2006740175	TRIT	TRITURUS	SPECIES		NEWT	LIV	1,000	NO	HK	HK	T	C	C	C	5/13/06	5/13/06	I	LA	GLOBAL AQUATIC CONSULTING
2006740248	CYOR	CYNOPS	ORIENTALIS	CHINESE DWARF	NEWT	LIV	2,300	NO	HK	HK	T	C	C	C	5/13/06	5/14/06	I	LA	DOLPHIN INTERNATIONAL
2006740258	TRIH	TRITURUS	HONGKONGENSIS	ALPINE	NEWT	LIV	900	NO	HK	PE	T	W	C	C	5/13/06	5/14/06	I	LA	AQUACO, INC.
2006740293	TRIT	TRITURUS	SPECIES		NEWT	LIV	200	NO	HK	HK	T	C	C	C	5/14/06	5/14/06	I	LA	AQUATIC CONNECTION
2006743177	CYPY	CYNOPS	PYRRHOGASTER	JAPANESE FIREBELLY	NEWT	LIV	55	NO	HK	HK	T	W	C	C	4/17/06	4/16/06	I	NY	TRANSSHIP DISCOUNTS LIMITED
2006743710	CYPY	CYNOPS	PYRRHOGASTER	JAPANESE FIREBELLY	NEWT	LIV	200	NO	CN	CN	T	C	C	C	2/15/06	2/12/06	I	NY	FISH MART INCORPORATED
2006743710	CNP?	CYNOPS	SPECIES	FIREBELLY	NEWT	LIV	75	NO	CN	CN	T	C	C	C	2/15/06	2/12/06	I	NY	FISH MART INCORPORATED
2006743783	CYPY	CYNOPS	PYRRHOGASTER	JAPANESE FIREBELLY	NEWT	LIV	450	NO	HK	HK	T	W	C	C	1/4/06	11/12/05	I	NY	ERDA INCORPORATED
2006743964	TRIT	TRITURUS	SPECIES		NEWT	LIV	800	NO	HK	HK	T	C	C	C	5/20/06	5/20/06	I	LA	GLOBAL AQUATIC CONSULTING
2006744058	CYOR	CYNOPS	ORIENTALIS	CHINESE DWARF	NEWT	LIV	1,750	NO	HK	HK	T	C	C	C	5/21/06	5/21/06	I	LA	DOLPHIN INTERNATIONAL
2006744058	PACB	PACHYTRITON	BREVIPES	TSITOU	NEWT	LIV	200	NO	HK	HK	T	C	C	C	5/21/06	5/21/06	I	LA	DOLPHIN INTERNATIONAL
2006744075	TRIH	TRITURUS	HONGKONGENSIS	ALPINE	NEWT	LIV	100	NO	HK	HK	T	W	C	C	5/21/06	5/21/06	I	LA	AQUACO, INC.
2006744152	CYPY	CYNOPS	PYRRHOGASTER	JAPANESE FIREBELLY	NEWT	LIV	50	NO	HK	HK	T	W	C	C	2/15/06	2/5/06	I	NY	TRANSSHIP DISCOUNTS LIMITED
2006744605	CYPY	CYNOPS	PYRRHOGASTER	JAPANESE FIREBELLY	NEWT	LIV	50	NO	HK	HK	T	W	C	C	2/13/06	2/12/06	I	NY	TRANSSHIP DISCOUNTS LIMITED
2006745006	TRIH	TRITURUS	HONGKONGENSIS	ALPINE	NEWT	LIV	100	NO	HK	HK	T	W	C	C	3/2/06	2/12/06	I	NY	INTERNATIONAL PET RESOURCES , LLC
2006745303	CYPY	CYNOPS	PYRRHOGASTER	JAPANESE FIREBELLY	NEWT	LIV	450	NO	HK	HK	T	W	C	C	11/21/05	11/12/05	I	NY	ERDA INCORPORATED
2006745321	CYPY	CYNOPS	PYRRHOGASTER	JAPANESE FIREBELLY	NEWT	LIV	450	NO	HK	HK	T	W	C	C	12/6/05	12/3/05	I	NY	ERDA INCORPORATED
2006745371	CYPY	CYNOPS	PYRRHOGASTER	JAPANESE FIREBELLY	NEWT	LIV	675	NO	HK	HK	T	W	C	C	4/17/06	4/15/06	I	NY	ERDA INCORPORATED
2006745799	CYPY	CYNOPS	PYRRHOGASTER	JAPANESE FIREBELLY	NEWT	LIV	1,700	NO	HK	HK	T	W	C	C	3/13/06	3/11/06	I	NY	ERDA INCORPORATED
2006746478	PLAB	PACHYTRITON	LABIATUS	UNTERSTEIN'S	NEWT	LIV	90	NO	CN	HK	T	W	C	C	5/25/06	5/25/06	I	DN	Bushmaster Reptiles Inc
2006746478	CNP?	CYNOPS	SPECIES	FIREBELLY	NEWT	LIV	100	NO	CN	HK	T	W	C	C	5/25/06	5/25/06	I	DN	Bushmaster Reptiles Inc
2006746478	CNP?	CYNOPS	SPECIES	FIREBELLY	NEWT	LIV	968	NO	CN	HK	T	W	C	C	5/25/06	5/25/06	I	DN	Bushmaster Reptiles Inc
2006746478	TYKW	TYLOTOTRITON	KWEICHOWENSIS	KWEICHOW CROCODILE	NEWT	LIV	100	NO	CN	HK	T	W	C	C	5/25/06	5/25/06	I	DN	Bushmaster Reptiles Inc
2006746523	CYPY	CYNOPS	PYRRHOGASTER	JAPANESE FIREBELLY	NEWT	LIV	200	NO	HK	HK	T	W	C	C	3/7/06	3/5/06	I	NY	TRANSSHIP DISCOUNTS LIMITED
2006746574	CYPY	CYNOPS	PYRRHOGASTER	JAPANESE FIREBELLY	NEWT	LIV	200	NO	HK	HK	T	W	C	C	3/13/06	3/12/06	I	NY	TRANSSHIP DISCOUNTS LIMITED
2006746574	PACB	PACHYTRITON	BREVIPES	TSITOU	NEWT	LIV	30	NO	HK	HK	T	W	C	C	3/13/06	3/12/06	I	NY	TRANSSHIP DISCOUNTS LIMITED
2006746596	CYOR	CYNOPS	ORIENTALIS	CHINESE DWARF	NEWT	LIV	3,000	NO	CN	CN	T	W	C	C	5/25/06	5/25/06	I	LA	Z IMPORTS INC.
2006747088	CYPY	CYNOPS	PYRRHOGASTER	JAPANESE FIREBELLY	NEWT	LIV	700	NO	HK	HK	T	W	C	C	3/7/06	3/4/06	I	NY	ERDA INCORPORATED
2006747098	CYPY	CYNOPS	PYRRHOGASTER	JAPANESE FIREBELLY	NEWT	LIV	500	NO	HK	HK	T	C	C	C	10/31/05	10/20/05	I	NY	ERDA INCORPORATED
2006747118	SASA	SALAMANDRA	SALAMANDRA	EUROPEAN FIRE	SALAMANDER	LIV	64	NO	GB	GB	T	W	C	C	5/24/06	5/24/06	I	MI	TWO AMIGOS IMPORT & EXPORT, INC.
2006747118	PLWA	PLEURODELES	WALTL	IBERIAN RIBBED	NEWT	LIV	20	NO	GB	GB	T	W	C	C	5/24/06	5/24/06	I	MI	TWO AMIGOS IMPORT & EXPORT, INC.
2006747118	SAL?	SALAMANDRA	SPECIES	FIRE	SALAMANDER	LIV	20	NO	GB	GB	T	W	C	C	5/24/06	5/24/06	I	MI	TWO AMIGOS IMPORT & EXPORT, INC.
2006747118	TRIT	TRITURUS	SPECIES		NEWT	LIV	60	NO	GB	GB	T	W	C	C	5/24/06	5/24/06	I	MI	TWO AMIGOS IMPORT & EXPORT, INC.
2006747675	CYOR	CYNOPS	ORIENTALIS	CHINESE DWARF	NEWT	LIV	500	NO	HK	HK	T	C	C	C	5/27/06	5/27/06	I	LA	DOLPHIN INTERNATIONAL
2006747675	PACB	PACHYTRITON	BREVIPES	TSITOU	NEWT	LIV	100	NO	HK	HK	T	C	C	C	5/27/06	5/27/06	I	LA	DOLPHIN INTERNATIONAL
2006747700	TRIT	TRITURUS	SPECIES		NEWT	LIV	200	NO	HK	HK	T	C	C	C	5/27/06	5/27/06	I	LA	GLOBAL AQUATIC CONSULTING

2006747792	CYOR	CYNOPS	ORIENTALIS	CHINESE DWARF	NEWT	LIV	500	NO	HK	HK	T	C	C	C	5/28/06	5/28/06	I	LA	DOLPHIN INTERNATIONAL
2006747792	PACB	PACHYTRITON	BREVIPES	TSITOU	NEWT	LIV	100	NO	HK	HK	T	C	C	C	5/28/06	5/28/06	I	LA	DOLPHIN INTERNATIONAL
2006747796	TRIH	TRITURUS	HONGKONGENSIS	ALPINE	NEWT	LIV	200	NO	HK	PE	T	W	C	C	5/28/06	5/28/06	I	LA	AQUACO, INC.
2006749814	PSHG	PACHYHYNOBIUS	SHANGCHENGENSIS	SHANGCHENG	SALAMANDER	LIV	12	NO	HK	HK	T	W	C	C	6/1/06	6/1/06	I	LA	NAME REMOVED
2006749814	CNP?	CYNOPS	SPECIES	FIREBELLY	NEWT	LIV	92	NO	HK	HK	T	W	C	C	6/1/06	6/1/06	I	LA	NAME REMOVED
2006749814	BTC?	BATRACHUPERUS	SPECIES	MOUNTAIN	SALAMANDER	LIV	76	NO	HK	HK	T	W	C	C	6/1/06	6/1/06	I	LA	NAME REMOVED
2006749814	TYKW	TYLOTOTRITON	KWEICHOWENSIS	KWEICHOW CROCODILE	NEWT	LIV	139	NO	HK	HK	T	W	C	C	6/1/06	6/1/06	I	LA	NAME REMOVED
2006749814	TYL?	TYLOTOTRITON	SPECIES	CROCODILE	NEWT	LIV	6	NO	HK	HK	T	W	C	C	6/1/06	6/1/06	I	LA	NAME REMOVED
2006749814	PCHI	PARAMESOTRITON	CHINENSIS	CHINESE WARTY	NEWT	LIV	2	NO	HK	HK	T	W	C	C	6/1/06	6/1/06	I	LA	NAME REMOVED
2006749919	CYPY	CYNOPS	PYRRHOGASTER	JAPANESE FIREBELLY	NEWT	LIV	50	NO	HK	HK	T	W	C	C	3/27/06	3/25/06	I	NY	TRANSSHIP DISCOUNTS LIMITED
2006749919	PACB	PACHYTRITON	BREVIPES	TSITOU	NEWT	LIV	30	NO	HK	HK	T	W	C	C	3/27/06	3/25/06	I	NY	TRANSSHIP DISCOUNTS LIMITED
2006750086	CYPY	CYNOPS	PYRRHOGASTER	JAPANESE FIREBELLY	NEWT	LIV	510	NO	HK	HK	T	W	C	C	3/25/06	3/25/06	I	NY	ERDA INCORPORATED
2006750553	CYPY	CYNOPS	PYRRHOGASTER	JAPANESE FIREBELLY	NEWT	LIV	150	NO	HK	HK	T	W	C	C	3/18/06	3/18/06	I	NY	ERDA INCORPORATED
2006750851	CYOR	CYNOPS	ORIENTALIS	CHINESE DWARF	NEWT	LIV	250	NO	HK	HK	T	C	C	C	6/3/06	6/3/06	I	LA	DOLPHIN INTERNATIONAL
2006750895	TRIT	TRITURUS	SPECIES		NEWT	LIV	800	NO	HK	HK	T	C	C	C	6/3/06	6/3/06	I	LA	GLOBAL AQUATIC CONSULTING
2006750962	TRIH	TRITURUS	HONGKONGENSIS	ALPINE	NEWT	LIV	200	NO	HK	HK	T	W	C	C	6/4/06	6/4/06	I	LA	AQUACO, INC.
2006750963	CYOR	CYNOPS	ORIENTALIS	CHINESE DWARF	NEWT	LIV	2,350	NO	HK	HK	T	C	C	C	6/4/06	6/4/06	I	LA	DOLPHIN INTERNATIONAL
2006753159	TRIT	TRITURUS	SPECIES		NEWT	LIV	600	NO	HK	HK	T	C	C	C	6/10/06	6/10/06	I	LA	GLOBAL AQUATIC CONSULTING
2006753214	TRIH	TRITURUS	HONGKONGENSIS	ALPINE	NEWT	LIV	550	NO	HK	HK	T	W	C	C	6/11/06	6/11/06	I	LA	AQUACO, INC.
2006753226	PACB	PACHYTRITON	BREVIPES	TSITOU	NEWT	LIV	300	NO	HK	HK	T	C	C	C	6/11/06	6/11/06	I	LA	DOLPHIN INTERNATIONAL
2006753690	CYPY	CYNOPS	PYRRHOGASTER	JAPANESE FIREBELLY	NEWT	LIV	100	NO	CN	CN	T	C	C	C	3/1/06	2/19/06	I	NY	FISH MART INCORPORATED
2006753690	CNP?	CYNOPS	SPECIES	FIREBELLY	NEWT	LIV	50	NO	CN	CN	T	C	C	C	3/1/06	2/19/06	I	NY	FISH MART INCORPORATED
2006753729	CYPY	CYNOPS	PYRRHOGASTER	JAPANESE FIREBELLY	NEWT	LIV	50	NO	HK	HK	T	W	C	C	4/10/06	4/9/06	I	NY	TRANSSHIP DISCOUNTS LIMITED
2006753907	CYPY	CYNOPS	PYRRHOGASTER	JAPANESE FIREBELLY	NEWT	LIV	100	NO	CN	CN	T	C	C	C	3/16/06	3/12/06	I	NY	FISH MART INCORPORATED
2006753907	CNP?	CYNOPS	SPECIES	FIREBELLY	NEWT	LIV	50	NO	CN	CN	T	C	C	C	3/16/06	3/12/06	I	NY	FISH MART INCORPORATED
2006753926	CYPY	CYNOPS	PYRRHOGASTER	JAPANESE FIREBELLY	NEWT	LIV	200	NO	HK	HK	T	W	C	C	4/25/06	4/23/06	I	NY	TRANSSHIP DISCOUNTS LIMITED
2006754544	CYPY	CYNOPS	PYRRHOGASTER	JAPANESE FIREBELLY	NEWT	LIV	125	NO	CN	CN	T	C	C	C	4/20/06	4/16/06	I	NY	FISH MART INCORPORATED
2006754544	CNP?	CYNOPS	SPECIES	FIREBELLY	NEWT	LIV	75	NO	CN	CN	T	C	C	C	4/20/06	4/16/06	I	NY	FISH MART INCORPORATED
2006754557	CYPY	CYNOPS	PYRRHOGASTER	JAPANESE FIREBELLY	NEWT	LIV	250	NO	HK	HK	T	W	C	C	1/25/06	1/21/06	I	NY	ERDA INCORPORATED
2006754900	CYPY	CYNOPS	PYRRHOGASTER	JAPANESE FIREBELLY	NEWT	LIV	150	NO	HK	HK	T	W	C	C	5/2/06	4/29/06	I	NY	TRANSSHIP DISCOUNTS LIMITED
2006755241	CYPY	CYNOPS	PYRRHOGASTER	JAPANESE FIREBELLY	NEWT	LIV	50	NO	HK	HK	T	W	C	C	4/1/06	4/1/06	I	NY	TRANSSHIP DISCOUNTS LIMITED
2006755324	CNP?	CYNOPS	SPECIES	FIREBELLY	NEWT	LIV	205	NO	CN	HK	T	W	C	C	6/15/06	6/15/06	I	DN	Bushmaster Reptiles Inc
2006755324	CNP?	CYNOPS	SPECIES	FIREBELLY	NEWT	LIV	1,000	NO	CN	HK	T	W	C	C	6/15/06	6/15/06	I	DN	Bushmaster Reptiles Inc
2006755399	CYOR	CYNOPS	ORIENTALIS	CHINESE DWARF	NEWT	LIV	2,000	NO	CN	CN	T	W	C	C	6/15/06	6/15/06	I	LA	Z IMPORTS INC.
2006755566	CYOR	CYNOPS	ORIENTALIS	CHINESE DWARF	NEWT	LIV	200	NO	CN	CN	T	F	C	C	3/14/06	3/3/06	I	NY	MIN HUA ENTERPRISE, INC.
2006755658	CYPY	CYNOPS	PYRRHOGASTER	JAPANESE FIREBELLY	NEWT	LIV	100	NO	CN	CN	T	C	C	C	3/6/06	2/26/06	I	NY	FISH MART INCORPORATED
2006755658	CNP?	CYNOPS	SPECIES	FIREBELLY	NEWT	LIV	50	NO	CN	CN	T	C	C	C	3/6/06	2/26/06	I	NY	FISH MART INCORPORATED
2006756039	CYPY	CYNOPS	PYRRHOGASTER	JAPANESE FIREBELLY	NEWT	LIV	550	NO	HK	HK	T	W	C	C	2/21/06	2/18/06	I	NY	ERDA INCORPORATED
2006756261	CYPY	CYNOPS	PYRRHOGASTER	JAPANESE FIREBELLY	NEWT	LIV	190	NO	HK	HK	T	W	C	C	1/30/06	1/29/06	I	NY	TRANSSHIP DISCOUNTS LIMITED
2006756261	PACB	PACHYTRITON	BREVIPES	TSITOU	NEWT	LIV	30	NO	HK	HK	T	W	C	C	1/30/06	1/29/06	I	NY	TRANSSHIP DISCOUNTS LIMITED
2006756351	CYPY	CYNOPS	PYRRHOGASTER	JAPANESE FIREBELLY	NEWT	LIV	190	NO	HK	HK	T	W	C	C	2/21/06	2/18/06	I	NY	TRANSSHIP DISCOUNTS LIMITED
2006756385	CYPY	CYNOPS	PYRRHOGASTER	JAPANESE FIREBELLY	NEWT	LIV	300	NO	HK	HK	T	C	C	C	3/29/06	3/15/06	I	NY	WORLDWIDE AQUATICS OF LONG ISLAND, INC.
2006756636	TRIT	TRITURUS	SPECIES		NEWT	LIV	800	NO	HK	HK	T	C	C	C	6/17/06	6/17/06	I	LA	GLOBAL AQUATIC CONSULTING
2006756655	CYOR	CYNOPS	ORIENTALIS	CHINESE DWARF	NEWT	LIV	750	NO	HK	HK	T	C	C	C	6/17/06	6/17/06	I	LA	DOLPHIN INTERNATIONAL
2006756730	PACB	PACHYTRITON	BREVIPES	TSITOU	NEWT	LIV	400	NO	HK	HK	T	C	C	C	6/18/06	6/18/06	I	LA	DOLPHIN INTERNATIONAL
2006756730	CYOR	CYNOPS	ORIENTALIS	CHINESE DWARF	NEWT	LIV	2,000	NO	HK	HK	T	C	C	C	6/18/06	6/18/06	I	LA	DOLPHIN INTERNATIONAL
2006756771	TRIT	TRITURUS	SPECIES		NEWT	LIV	98	NO	SG	SG	T	W	C	C	6/18/06	6/18/06	I	LA	TROPICAL FISH CONSOLIDATORS LTD.
2006756776	CNP?	CYNOPS	SPECIES	FIREBELLY	NEWT	LIV	400	NO	HK	HK	T	C	C	C	6/19/06	6/19/06	I	AT	SUN PET LTD.
2006758128	TRIH	TRITURUS	HONGKONGENSIS	ALPINE	NEWT	LIV	500	NO	HK	HK	T	W	C	C	5/9/06	4/30/06	I	NY	INTERNATIONAL PET RESOURCES , LLC
2006759864	CYOR	CYNOPS	ORIENTALIS	CHINESE DWARF	NEWT	LIV	3,000	NO	CN	CN	T	W	C	C	6/22/06	6/22/06	I	LA	Z IMPORTS INC.
2006760943	SASA	SALAMANDRA	SALAMANDRA	EUROPEAN FIRE	SALAMANDER	LIV	31	NO	ES	ES	S	W	C	C	5/30/06	5/24/06	I	SF	MUSEUM VERTEBRATE ZOOLOGY
2006761131	CYOR	CYNOPS	ORIENTALIS	CHINESE DWARF	NEWT	LIV	250	NO	HK	HK	T	C	C	C	6/24/06	6/24/06	I	LA	DOLPHIN INTERNATIONAL
2006761132	TRIT	TRITURUS	SPECIES		NEWT	LIV	1,000	NO	HK	HK	T	C	C	C	6/24/06	6/24/06	I	LA	GLOBAL AQUATIC CONSULTING
2006761201	PACB	PACHYTRITON	BREVIPES	TSITOU	NEWT	LIV	200	NO	HK	HK	T	C	C	C	6/25/06	6/25/06	I	LA	DOLPHIN INTERNATIONAL
2006761201	CYOR	CYNOPS	ORIENTALIS	CHINESE DWARF	NEWT	LIV	1,600	NO	HK	HK	T	C	C	C	6/25/06	6/25/06	I	LA	DOLPHIN INTERNATIONAL
2006761216	TRIH	TRITURUS	HONGKONGENSIS	ALPINE	NEWT	LIV	50	NO	HK	HK	T	W	C	C	6/25/06	6/25/06	I	LA	AQUACO, INC.
2006761275	CNP?	CYNOPS	SPECIES	FIREBELLY	NEWT	LIV	400	NO	HK	HK	T	C	C	C	6/26/06	6/26/06	I	AT	SUN PET LTD.
2006764978	TRIH	TRITURUS	HONGKONGENSIS	ALPINE	NEWT	LIV	50	NO	HK	HK	T	W	C	C	7/2/06	7/2/06	I	LA	AQUACO, INC.
2006764987	PACB	PACHYTRITON	BREVIPES	TSITOU	NEWT	LIV	100	NO	HK	HK	T	C	C	C	7/2/06	7/2/06	I	LA	DOLPHIN INTERNATIONAL

2006764987	CYOR	CYNOPS	ORIENTALIS	CHINESE DWARF	NEWT	LIV	1,950	NO	HK	HK	T	C	C	C	7/2/06	7/2/06	I	LA	DOLPHIN INTERNATIONAL
2006767266	CYOR	CYNOPS	ORIENTALIS	CHINESE DWARF	NEWT	LIV	250	NO	HK	HK	T	C	C	C	7/8/06	7/8/06	I	LA	DOLPHIN INTERNATIONAL
2006767266	PACB	PACHYTRITON	BREVIPES	TSITOU	NEWT	LIV	100	NO	HK	HK	T	C	C	C	7/8/06	7/8/06	I	LA	DOLPHIN INTERNATIONAL
2006767352	TRIH	TRITURUS	HONGKONGENSIS	ALPINE	NEWT	LIV	350	NO	HK	HK	T	W	C	C	7/9/06	7/9/06	I	LA	AQUACO, INC.
2006767370	PACB	PACHYTRITON	BREVIPES	TSITOU	NEWT	LIV	200	NO	HK	HK	T	C	C	C	7/9/06	7/9/06	I	LA	DOLPHIN INTERNATIONAL
2006767370	CYOR	CYNOPS	ORIENTALIS	CHINESE DWARF	NEWT	LIV	1,000	NO	HK	HK	T	C	C	C	7/9/06	7/9/06	I	LA	DOLPHIN INTERNATIONAL
2006768465	NEW?	PACHYTRITON	SPECIES	CHINESE	NEWT	LIV	200	NO	CN	CN	T	C	C	C	7/11/06	7/11/06	I	SF	Golden Phoenix Fisheries, USA
2006771668	TRIT	TRITURUS	SPECIES		NEWT	LIV	1,800	NO	HK	HK	T	W	C	C	7/15/06	7/15/06	I	LA	GLOBAL AQUATIC CONSULTING
2006771740	PACB	PACHYTRITON	BREVIPES	TSITOU	NEWT	LIV	200	NO	HK	HK	T	C	C	C	7/16/06	7/16/06	I	LA	DOLPHIN INTERNATIONAL
2006771740	CYOR	CYNOPS	ORIENTALIS	CHINESE DWARF	NEWT	LIV	4,150	NO	HK	HK	T	C	C	C	7/16/06	7/16/06	I	LA	DOLPHIN INTERNATIONAL
2006771772	TRIH	TRITURUS	HONGKONGENSIS	ALPINE	NEWT	LIV	100	NO	HK	HK	T	W	C	C	7/16/06	7/16/06	I	LA	AQUACO, INC.
2006771791	CNP?	CYNOPS	SPECIES	FIREBELLY	NEWT	LIV	60	NO	SG	SG	T	W	C	C	7/16/06	7/16/06	I	LA	TROPICAL FISH CONSOLIDATORS LTD.
2006771802	CNP?	CYNOPS	SPECIES	FIREBELLY	NEWT	LIV	790	NO	HK	HK	T	C	C	C	7/17/06	7/17/06	I	AT	SUN PET LTD.
2006774451	BODO	BOLITOGLOSSA	DOFLEINI	ALTA VERAPAZ	SALAMANDER	LIV	2	NO	HN	HN	T	W	C	C	1/12/05	1/12/05	I	MI	STRICTLY REPTILES, INC.
2006775400	CYOR	CYNOPS	ORIENTALIS	CHINESE DWARF	NEWT	LIV	3,000	NO	CN	CN	T	W	C	C	7/21/06	7/21/06	I	LA	Z IMPORTS INC.
2006775605	TRIT	TRITURUS	SPECIES		NEWT	LIV	1,600	NO	HK	HK	T	W	C	C	7/22/06	7/22/06	I	LA	GLOBAL AQUATIC CONSULTING
2006775670	PACB	PACHYTRITON	BREVIPES	TSITOU	NEWT	LIV	100	NO	HK	HK	T	C	C	C	7/23/06	7/23/06	I	LA	DOLPHIN INTERNATIONAL
2006775670	CYOR	CYNOPS	ORIENTALIS	CHINESE DWARF	NEWT	LIV	1,800	NO	HK	HK	T	C	C	C	7/23/06	7/23/06	I	LA	DOLPHIN INTERNATIONAL
2006775687	TRIH	TRITURUS	HONGKONGENSIS	ALPINE	NEWT	LIV	200	NO	HK	HK	T	W	C	C	7/23/06	7/23/06	I	LA	AQUACO, INC.
2006778500	CYPY	CYNOPS	PYRRHOGASTER	JAPANESE FIREBELLY	NEWT	LIV	225	NO	HK	HK	T	W	C	C	4/4/06	4/1/06	I	NY	ERDA INCORPORATED
2006778595	CYPY	CYNOPS	PYRRHOGASTER	JAPANESE FIREBELLY	NEWT	LIV	200	NO	CN	CN	T	C	C	C	3/29/06	3/26/06	I	NY	FISH MART INCORPORATED
2006778595	CNP?	CYNOPS	SPECIES	FIREBELLY	NEWT	LIV	75	NO	CN	CN	T	C	C	C	3/29/06	3/26/06	I	NY	FISH MART INCORPORATED
2006778669	CYOR	CYNOPS	ORIENTALIS	CHINESE DWARF	NEWT	LIV	2,500	NO	CN	CN	T	W	C	C	7/28/06	7/28/06	I	LA	Z IMPORTS INC.
2006778858	SAL?	SALAMANDRA	SPECIES	FIRE	SALAMANDER	LIV	27	NO	GB	GB	T	C	C	C	7/28/06	7/28/06	I	MI	TWO AMIGOS IMPORT & EXPORT, INC.
2006779123	CYOR	CYNOPS	ORIENTALIS	CHINESE DWARF	NEWT	LIV	750	NO	HK	HK	T	C	C	C	7/29/06	7/29/06	I	LA	DOLPHIN INTERNATIONAL
2006779148	TRIT	TRITURUS	SPECIES		NEWT	LIV	1,200	NO	HK	HK	T	W	C	C	7/29/06	7/29/06	I	LA	GLOBAL AQUATIC CONSULTING
2006779229	TRIH	TRITURUS	HONGKONGENSIS	ALPINE	NEWT	LIV	400	NO	HK	HK	T	W	C	C	7/30/06	7/30/06	I	LA	AQUACO, INC.
2006779231	CNP?	CYNOPS	SPECIES	FIREBELLY	NEWT	LIV	25	NO	SG	SG	T	W	C	C	7/30/06	7/30/06	I	LA	TROPICAL FISH CONSOLIDATORS LTD.
2006779241	PACB	PACHYTRITON	BREVIPES	TSITOU	NEWT	LIV	200	NO	HK	HK	T	C	C	C	7/30/06	7/30/06	I	LA	DOLPHIN INTERNATIONAL
2006779241	CYOR	CYNOPS	ORIENTALIS	CHINESE DWARF	NEWT	LIV	250	NO	HK	HK	T	C	C	C	7/30/06	7/30/06	I	LA	DOLPHIN INTERNATIONAL
2006780983	CYOR	CYNOPS	ORIENTALIS	CHINESE DWARF	NEWT	LIV	100	NO	SG	SG	T	C	C	C	6/20/06	6/11/06	I	NY	INTERNATIONAL PET RESOURCES , LLC
2006781027	CYPY	CYNOPS	PYRRHOGASTER	JAPANESE FIREBELLY	NEWT	LIV	200	NO	HK	HK	*	W	C	C	6/19/06	6/17/06	I	NY	TRANSSHIP DISCOUNTS LIMITED
2006781027	PACB	PACHYTRITON	BREVIPES	TSITOU	NEWT	LIV	30	NO	HK	HK	*	W	C	C	6/19/06	6/17/06	I	NY	TRANSSHIP DISCOUNTS LIMITED
2006782662	CYOR	CYNOPS	ORIENTALIS	CHINESE DWARF	NEWT	LIV	2,500	NO	CN	CN	T	W	C	C	8/4/06	8/4/06	I	LA	Z IMPORTS INC.
2006783086	CYOR	CYNOPS	ORIENTALIS	CHINESE DWARF	NEWT	LIV	250	NO	HK	HK	T	C	C	C	8/5/06	8/5/06	I	LA	DOLPHIN INTERNATIONAL
2006783093	TRIT	TRITURUS	SPECIES		NEWT	LIV	800	NO	HK	HK	T	W	C	C	8/5/06	8/5/06	I	LA	GLOBAL AQUATIC CONSULTING
2006783147	TRIH	TRITURUS	HONGKONGENSIS	ALPINE	NEWT	LIV	250	NO	HK	HK	T	W	C	C	8/5/06	8/6/06	I	LA	AQUACO, INC.
2006783161	PACB	PACHYTRITON	BREVIPES	TSITOU	NEWT	LIV	100	NO	HK	HK	T	C	C	C	8/5/06	8/6/06	I	LA	DOLPHIN INTERNATIONAL
2006783161	CYOR	CYNOPS	ORIENTALIS	CHINESE DWARF	NEWT	LIV	1,000	NO	HK	HK	T	C	C	C	8/5/06	8/6/06	I	LA	DOLPHIN INTERNATIONAL
2006783220	CNP?	CYNOPS	SPECIES	FIREBELLY	NEWT	LIV	400	NO	HK	HK	T	C	C	C	8/7/06	8/7/06	I	AT	SUN PET LTD.
2006784377	CYPY	CYNOPS	PYRRHOGASTER	JAPANESE FIREBELLY	NEWT	LIV	330	NO	HK	HK	T	C	C	C	6/7/06	6/3/06	I	NY	ERDA INCORPORATED
2006784612	TRIH	TRITURUS	HONGKONGENSIS	ALPINE	NEWT	LIV	800	NO	HK	HK	T	C	C	C	8/8/06	8/8/06	I	TP	5-D TROPICAL, INC.
2006784633	CYPY	CYNOPS	PYRRHOGASTER	JAPANESE FIREBELLY	NEWT	LIV	200	NO	HK	HK	T	W	C	C	5/22/06	5/21/06	I	NY	TRANSSHIP DISCOUNTS LIMITED
2006784658	NEW?	PACHYTRITON	SPECIES	CHINESE	NEWT	LIV	125	NO	CN	CN	T	C	C	C	5/26/06	5/21/06	I	NY	FISH MART INCORPORATED
2006784658	CNP?	CYNOPS	SPECIES	FIREBELLY	NEWT	LIV	75	NO	CN	CN	T	C	C	C	5/26/06	5/21/06	I	NY	FISH MART INCORPORATED
2006784728	CYPY	CYNOPS	PYRRHOGASTER	JAPANESE FIREBELLY	NEWT	LIV	150	NO	HK	HK	T	W	C	C	6/26/06	6/24/06	I	NY	ERDA INCORPORATED
2006785016	TRIH	TRITURUS	HONGKONGENSIS	ALPINE	NEWT	LIV	500	NO	HK	HK	T	W	C	C	6/27/06	6/19/06	I	NY	INTERNATIONAL PET RESOURCES , LLC
2006787449	TRIT	TRITURUS	SPECIES		NEWT	LIV	1,600	NO	HK	HK	T	W	C	C	8/12/06	8/12/06	I	LA	GLOBAL AQUATIC CONSULTING
2006787456	CYOR	CYNOPS	ORIENTALIS	CHINESE DWARF	NEWT	LIV	500	NO	HK	HK	T	C	C	C	8/12/06	8/13/06	I	LA	DOLPHIN INTERNATIONAL
2006787517	PACB	PACHYTRITON	BREVIPES	TSITOU	NEWT	LIV	100	NO	HK	HK	T	C	C	C	8/13/06	8/13/06	I	LA	DOLPHIN INTERNATIONAL
2006787517	CYOR	CYNOPS	ORIENTALIS	CHINESE DWARF	NEWT	LIV	1,250	NO	HK	HK	T	C	C	C	8/13/06	8/13/06	I	LA	DOLPHIN INTERNATIONAL
2006787519	TRIH	TRITURUS	HONGKONGENSIS	ALPINE	NEWT	LIV	75	NO	HK	HK	T	W	C	C	8/13/06	8/13/06	I	LA	AQUACO, INC.
2006787552	CNP?	CYNOPS	SPECIES	FIREBELLY	NEWT	LIV	400	NO	HK	HK	T	C	C	C	8/14/06	8/14/06	I	AT	SUN PET LTD.
2006788646	HYN?	HYNOBIUS	SPECIES		SALAMANDER	LIV	110	NO	JP	JP	T	W	C	C	8/15/06	8/15/06	I	LA	U.S. GLOBAL EXOTICS, INC.
2006788646	CYPY	CYNOPS	PYRRHOGASTER	JAPANESE FIREBELLY	NEWT	LIV	700	NO	JP	JP	T	W	C	C	8/15/06	8/15/06	I	LA	U.S. GLOBAL EXOTICS, INC.
2006788646	CNP?	CYNOPS	SPECIES	FIREBELLY	NEWT	LIV	50	NO	JP	JP	T	W	C	C	8/15/06	8/15/06	I	LA	U.S. GLOBAL EXOTICS, INC.
2006789801	SASA	SALAMANDRA	SALAMANDRA	EUROPEAN FIRE	SALAMANDER	LIV	30	NO	DE	DE	T	C	C	C	8/16/06	8/16/06	I	DF	U.S. GLOBAL EXOTICS, INC.
2006789926	CYPY	CYNOPS	PYRRHOGASTER	JAPANESE FIREBELLY	NEWT	LIV	630	NO	HK	HK	T	W	C	C	7/5/06	7/1/06	I	NY	ERDA INCORPORATED
2006790173	TYL?	TYLOTOTRITON	SPECIES	CROCODILE	NEWT	LIV	10	NO	HK	HK	T	W	C	C	8/17/06	8/17/06	I	LA	U.S. GLOBAL EXOTICS, INC.
2006790212	CYPY	CYNOPS	PYRRHOGASTER	JAPANESE FIREBELLY	NEWT	LIV	50	NO	HK	HK	T	W	C	C	5/26/06	5/27/06	I	NY	TRANSSHIP DISCOUNTS LIMITED

2006791146	CYOR	CYNOPS	ORIENTALIS	CHINESE DWARF	NEWT	LIV	250	NO	HK	HK	T	C	C	C	8/19/06	8/19/06	I	LA	DOLPHIN INTERNATIONAL
2006791153	TRIT	TRITURUS	SPECIES		NEWT	LIV	1,600	NO	HK	HK	T	W	C	C	8/19/06	8/19/06	I	LA	GLOBAL AQUATIC CONSULTING
2006791240	PACB	PACHYTRITON	BREVIPES	TSITOU	NEWT	LIV	200	NO	HK	HK	T	C	C	C	8/20/06	8/20/06	I	LA	DOLPHIN INTERNATIONAL
2006791240	CYOR	CYNOPS	ORIENTALIS	CHINESE DWARF	NEWT	LIV	1,000	NO	HK	HK	T	C	C	C	8/20/06	8/20/06	I	LA	DOLPHIN INTERNATIONAL
2006791256	TRIH	TRITURUS	HONGKONGENSIS	ALPINE	NEWT	LIV	300	NO	HK	HK	T	W	C	C	8/20/06	8/20/06	I	LA	AQUACO, INC.
2006791279	TRIT	TRITURUS	SPECIES		NEWT	LIV	200	NO	HK	HK	T	C	C	C	8/20/06	8/20/06	I	LA	AQUATIC CONNECTION
2006791306	CNP?	CYNOPS	SPECIES	FIREBELLY	NEWT	LIV	400	NO	HK	HK	T	C	C	C	8/21/06	8/21/06	I	AT	SUN PET LTD.
2006791382	CYPY	CYNOPS	PYRRHOGASTER	JAPANESE FIREBELLY	NEWT	LIV	125	NO	CN	CN	T	C	C	C	7/10/06	7/2/06	I	NY	FISH MART INCORPORATED
2006791406	CYPY	CYNOPS	PYRRHOGASTER	JAPANESE FIREBELLY	NEWT	LIV	150	NO	HK	HK	T	W	C	C	5/22/06	5/20/06	I	NY	ERDA INCORPORATED
2006791523	CYPY	CYNOPS	PYRRHOGASTER	JAPANESE FIREBELLY	NEWT	LIV	200	NO	HK	HK	T	W	C	C	6/15/06	6/10/06	I	NY	ERDA INCORPORATED
2006791566	PHNG	PARAMESOTRITON	HONGKONGENSIS	HONG KONG WARTY	NEWT	LIV	1,600	NO	HK	HK	T	C	C	C	8/21/06	8/23/06	I	TP	5-D TROPICAL, INC.
2006791653	CYOR	CYNOPS	ORIENTALIS	CHINESE DWARF	NEWT	LIV	100	NO	SG	SG	T	W	C	C	5/31/06	5/21/06	I	NY	INTERNATIONAL PET RESOURCES , LLC
2006791667	CYPY	CYNOPS	PYRRHOGASTER	JAPANESE FIREBELLY	NEWT	LIV	50	NO	HK	HK	*	W	C	C	6/13/06	6/11/06	I	NY	TRANSSHIP DISCOUNTS LIMITED
2006791667	PACB	PACHYTRITON	BREVIPES	TSITOU	NEWT	LIV	90	NO	HK	HK	*	W	C	C	6/13/06	6/11/06	I	NY	TRANSSHIP DISCOUNTS LIMITED
2006791671	CYPY	CYNOPS	PYRRHOGASTER	JAPANESE FIREBELLY	NEWT	LIV	750	NO	HK	HK	T	W	C	C	5/31/06	5/27/06	I	NY	ERDA INCORPORATED
2006792541	CYPY	CYNOPS	PYRRHOGASTER	JAPANESE FIREBELLY	NEWT	LIV	50	NO	HK	HK	T	W	C	C	5/15/06	5/14/06	I	NY	TRANSSHIP DISCOUNTS LIMITED
2006792541	PACB	PACHYTRITON	BREVIPES	TSITOU	NEWT	LIV	30	NO	HK	HK	T	W	C	C	5/15/06	5/14/06	I	NY	TRANSSHIP DISCOUNTS LIMITED
2006794817	CYOR	CYNOPS	ORIENTALIS	CHINESE DWARF	NEWT	LIV	3,000	NO	CN	CN	T	W	C	C	8/25/06	8/25/06	I	LA	Z IMPORTS INC.
2006794930	CYOR	CYNOPS	ORIENTALIS	CHINESE DWARF	NEWT	LIV	500	NO	HK	HK	T	C	C	C	8/26/06	8/26/06	I	LA	DOLPHIN INTERNATIONAL
2006794968	TRIT	TRITURUS	SPECIES		NEWT	LIV	1,600	NO	HK	HK	T	W	C	C	8/26/06	8/26/06	I	LA	GLOBAL AQUATIC CONSULTING
2006795069	TRIH	TRITURUS	HONGKONGENSIS	ALPINE	NEWT	LIV	50	NO	HK	HK	T	W	C	C	8/27/06	8/27/06	I	LA	AQUACO, INC.
2006795073	PACB	PACHYTRITON	BREVIPES	TSITOU	NEWT	LIV	100	NO	HK	HK	T	C	C	C	8/27/06	8/27/06	I	LA	DOLPHIN INTERNATIONAL
2006795073	CYOR	CYNOPS	ORIENTALIS	CHINESE DWARF	NEWT	LIV	1,250	NO	HK	HK	T	C	C	C	8/27/06	8/27/06	I	LA	DOLPHIN INTERNATIONAL
2006795086	TRIT	TRITURUS	SPECIES		NEWT	LIV	200	NO	HK	HK	T	C	C	C	8/27/06	8/27/06	I	LA	AQUATIC CONNECTION
2006795275	PHNG	PARAMESOTRITON	HONGKONGENSIS	HONG KONG WARTY	NEWT	LIV	1,600	NO	HK	HK	T	C	C	C	8/28/06	8/29/06	I	TP	5-D TROPICAL, INC.
2006798628	CYOR	CYNOPS	ORIENTALIS	CHINESE DWARF	NEWT	LIV	500	NO	HK	HK	T	C	C	C	9/2/06	9/2/06	I	LA	DOLPHIN INTERNATIONAL
2006798647	TRIT	TRITURUS	SPECIES		NEWT	LIV	1,000	NO	HK	HK	T	W	C	C	9/2/06	9/2/06	I	LA	GLOBAL AQUATIC CONSULTING
2006798710	CYOR	CYNOPS	ORIENTALIS	CHINESE DWARF	NEWT	LIV	1,100	NO	HK	HK	T	C	C	C	9/3/06	9/3/06	I	LA	DOLPHIN INTERNATIONAL
2006798743	TRIH	TRITURUS	HONGKONGENSIS	ALPINE	NEWT	LIV	50	NO	HK	HK	T	W	C	C	9/3/06	9/3/06	I	LA	AQUACO, INC.
2006798751	TRIT	TRITURUS	SPECIES		NEWT	LIV	600	NO	HK	HK	T	C	C	C	9/3/06	9/3/06	I	LA	AQUATIC CONNECTION
2006798759	CNP?	CYNOPS	SPECIES	FIREBELLY	NEWT	LIV	400	NO	HK	HK	T	C	C	C	9/4/06	9/4/06	I	AT	SUN PET LTD.
2006799041	CNP?	CYNOPS	SPECIES	FIREBELLY	NEWT	LIV	300	NO	CN	CN	T	C	C	C	8/5/06	7/30/06	I	NY	FISH MART INCORPORATED
2006800353	CYOR	CYNOPS	ORIENTALIS	CHINESE DWARF	NEWT	LIV	90	NO	SG	SG	T	W	C	C	7/20/06	7/9/06	I	NY	INTERNATIONAL PET RESOURCES , LLC
2006800457	HDM?	HYDROMANTES	SPECIES		SALAMANDER	LIV	25	NO	CA	CA	T	C	C	C	8/21/06	8/21/06	I	BN	Exemptions 6 and 7(C)
2006800457	HDM?	HYDROMANTES	SPECIES		SALAMANDER	LIV	10	NO	CA	CA	T	C	C	C	8/21/06	8/21/06	I	BN	Exemptions 6 and 7(C)
2006800457	SAL?	SALAMANDRA	SPECIES	FIRE	SALAMANDER	LIV	15	NO	CA	CA	T	C	C	C	8/21/06	8/21/06	I	BN	Exemptions 6 and 7(C)
2006800457	SAL?	SALAMANDRA	SPECIES	FIRE	SALAMANDER	LIV	20	NO	CA	CA	T	C	C	C	8/21/06	8/21/06	I	BN	Exemptions 6 and 7(C)
2006801324	CNP?	CYNOPS	SPECIES	FIREBELLY	NEWT	LIV	100	NO	CN	CN	T	C	C	C	7/25/06	7/16/06	I	NY	FISH MART INCORPORATED
2006801328	CYOR	CYNOPS	ORIENTALIS	CHINESE DWARF	NEWT	LIV	3,000	NO	CN	CN	T	W	C	C	9/8/06	9/8/06	I	LA	Z IMPORTS INC.
2006801859	CYOR	CYNOPS	ORIENTALIS	CHINESE DWARF	NEWT	LIV	350	NO	HK	HK	T	C	C	C	9/9/06	9/9/06	I	LA	DOLPHIN INTERNATIONAL
2006801876	TRIT	TRITURUS	SPECIES		NEWT	LIV	200	NO	HK	HK	T	W	C	C	9/9/06	9/9/06	I	LA	GLOBAL AQUATIC CONSULTING
2006801945	CYOR	CYNOPS	ORIENTALIS	CHINESE DWARF	NEWT	LIV	850	NO	HK	HK	T	C	C	C	9/10/06	9/10/06	I	LA	DOLPHIN INTERNATIONAL
2006801945	PACB	PACHYTRITON	BREVIPES	TSITOU	NEWT	LIV	300	NO	HK	HK	T	C	C	C	9/10/06	9/10/06	I	LA	DOLPHIN INTERNATIONAL
2006801976	TRIH	TRITURUS	HONGKONGENSIS	ALPINE	NEWT	LIV	250	NO	HK	HK	T	W	C	C	9/10/06	9/10/06	I	LA	AQUACO, INC.
2006801989	TRIT	TRITURUS	SPECIES		NEWT	LIV	600	NO	HK	HK	T	C	C	C	9/10/06	9/10/06	I	LA	AQUATIC CONNECTION
2006802005	CNP?	CYNOPS	SPECIES	FIREBELLY	NEWT	LIV	400	NO	HK	HK	T	C	C	C	9/10/06	9/11/06	I	AT	SUN PET LTD.
2006802888	NEW?	PACHYTRITON	SPECIES	CHINESE	NEWT	LIV	200	NO	CN	CN	T	C	C	C	9/12/06	9/12/06	I	SF	Golden Phoenix Fisheries, USA
2006805078	TRIH	TRITURUS	HONGKONGENSIS	ALPINE	NEWT	LIV	1,600	NO	HK	HK	T	C	C	C	9/15/06	9/12/06	I	TP	5-D TROPICAL, INC.
2006805542	CYOR	CYNOPS	ORIENTALIS	CHINESE DWARF	NEWT	LIV	250	NO	HK	HK	T	C	C	C	9/16/06	9/16/06	I	LA	DOLPHIN INTERNATIONAL
2006805558	TRIT	TRITURUS	SPECIES		NEWT	LIV	200	NO	HK	HK	T	W	C	C	9/16/06	9/16/06	I	LA	GLOBAL AQUATIC CONSULTING
2006805605	CYOR	CYNOPS	ORIENTALIS	CHINESE DWARF	NEWT	LIV	1,350	NO	HK	HK	T	C	C	C	9/17/06	9/17/06	I	LA	DOLPHIN INTERNATIONAL
2006805605	PACB	PACHYTRITON	BREVIPES	TSITOU	NEWT	LIV	300	NO	HK	HK	T	C	C	C	9/17/06	9/17/06	I	LA	DOLPHIN INTERNATIONAL
2006805628	TRIH	TRITURUS	HONGKONGENSIS	ALPINE	NEWT	LIV	100	NO	HK	HK	T	W	C	C	9/17/06	9/17/06	I	LA	AQUACO, INC.
2006805648	TRIT	TRITURUS	SPECIES		NEWT	LIV	200	NO	VS	HK	T	W	C	C	9/17/06	9/17/06	I	LA	AQUATIC CONNECTION
2006805678	CNP?	CYNOPS	SPECIES	FIREBELLY	NEWT	LIV	400	NO	HK	HK	T	C	C	C	9/18/06	9/18/06	I	AT	SUN PET LTD.
2006806454	PHNG	PARAMESOTRITON	HONGKONGENSIS	HONG KONG WARTY	NEWT	LIV	1,600	NO	HK	HK	T	C	C	C	9/18/06	9/19/06	I	TP	5-D TROPICAL, INC.
2006807813	BMEX	BOLITOGLOSSA	MEXICANA	MEX MUSHROOMTONGUE	SALAMANDER	LIV	1	NO	HN	HN	T	W	C	C	1/17/06	1/17/06	I	MI	STRICTLY REPTILES, INC.
2006808886	SASA	SALAMANDRA	SALAMANDRA	EUROPEAN FIRE	SALAMANDER	LIV	200	NO	SI	SI	T	D	C	C	12/22/05	12/21/05	I	MI	GLOBAL TROPICAL IMP/EXP
2006809531	CYOR	CYNOPS	ORIENTALIS	CHINESE DWARF	NEWT	LIV	3,000	NO	CN	CN	T	W	C	C	9/22/06	9/22/06	I	LA	Z IMPORTS INC.
2006809957	CYOR	CYNOPS	ORIENTALIS	CHINESE DWARF	NEWT	LIV	250	NO	HK	HK	T	C	C	C	9/23/06	9/23/06	I	LA	DOLPHIN INTERNATIONAL

2006809970	TRIT	TRITURUS	SPECIES		NEWT	LIV	600	NO	HK	HK	T	W	C	C	9/23/06	9/23/06	I	LA	GLOBAL AQUATIC CONSULTING
2006810024	CYOR	CYNOPS	ORIENTALIS	CHINESE DWARF	NEWT	LIV	1,870	NO	HK	HK	T	C	C	C	9/24/06	9/24/06	I	LA	DOLPHIN INTERNATIONAL
2006810024	PACB	PACHYTRITON	BREVIPES	TSITOU	NEWT	LIV	100	NO	HK	HK	T	C	C	C	9/24/06	9/24/06	I	LA	DOLPHIN INTERNATIONAL
2006810044	TRIH	TRITURUS	HONGKONGENSIS	ALPINE	NEWT	LIV	50	NO	HK	HK	T	W	C	C	9/24/06	9/24/06	I	LA	AQUACO, INC.
2006810046	TRIT	TRITURUS	SPECIES		NEWT	LIV	800	NO	HK	HK	T	W	C	C	9/24/06	9/24/06	I	LA	AQUATIC CONNECTION
2006810076	CNP?	CYNOPS	SPECIES	FIREBELLY	NEWT	LIV	400	NO	HK	HK	T	C	C	C	9/25/06	9/25/06	I	AT	SUN PET LTD.
2006810164	BODO	BOLITOGLOSSA	DOFLEINI	ALTA VERAPAZ	SALAMANDER	LIV	3	NO	GT	GT	T	W	C	C	1/30/06	1/30/06	I	MI	DHAR TROPICAL LLC
2006810243	BODO	BOLITOGLOSSA	DOFLEINI	ALTA VERAPAZ	SALAMANDER	LIV	4	NO	GT	GT	T	W	C	C	6/25/06	6/25/06	I	MI	DHAR TROPICAL LLC
2006810645	CYPY	CYNOPS	PYRRHOGASTER	JAPANESE FIREBELLY	NEWT	LIV	150	NO	CN	CN	T	C	C	C	1/11/06	1/8/06	I	NY	FISH MART INCORPORATED
2006810645	CNP?	CYNOPS	SPECIES	FIREBELLY	NEWT	LIV	75	NO	CN	CN	T	C	C	C	1/11/06	1/8/06	I	NY	FISH MART INCORPORATED
2006811024	TRIH	TRITURUS	HONGKONGENSIS	ALPINE	NEWT	LIV	245	NO	HK	HK	T	C	C	C	8/1/06	7/24/06	I	NY	TRI-COUNTY TROPICALS, INC.
2006811262	CYPY	CYNOPS	PYRRHOGASTER	JAPANESE FIREBELLY	NEWT	LIV	60	NO	HK	HK	T	W	C	C	8/9/06	8/6/06	I	NY	ERDA INCORPORATED
2006812813	CYPY	CYNOPS	PYRRHOGASTER	JAPANESE FIREBELLY	NEWT	LIV	810	NO	HK	HK	T	W	C	C	7/19/06	7/15/06	I	NY	ERDA INCORPORATED
2006812880	TRIT	TRITURUS	SPECIES		NEWT	LIV	400	NO	HK	HK	T	W	C	C	9/28/06	9/28/06	I	LA	AQUATIC CONNECTION
2006812899	CYOR	CYNOPS	ORIENTALIS	CHINESE DWARF	NEWT	LIV	4,000	NO	CN	CN	T	W	C	C	9/28/06	9/28/06	I	LA	Z IMPORTS INC.
2006813341	CYPY	CYNOPS	PYRRHOGASTER	JAPANESE FIREBELLY	NEWT	LIV	510	NO	HK	HK	T	W	C	C	8/31/06	8/26/06	I	NY	ERDA INCORPORATED
2006813455	CNP?	CYNOPS	SPECIES	FIREBELLY	NEWT	LIV	100	NO	CN	CN	T	C	C	C	8/22/06	8/13/06	I	NY	FISH MART INCORPORATED
2006814049	CYOR	CYNOPS	ORIENTALIS	CHINESE DWARF	NEWT	LIV	250	NO	HK	HK	T	C	C	C	9/30/06	9/30/06	I	LA	DOLPHIN INTERNATIONAL
2006814049	PACB	PACHYTRITON	BREVIPES	TSITOU	NEWT	LIV	100	NO	HK	HK	T	C	C	C	9/30/06	9/30/06	I	LA	DOLPHIN INTERNATIONAL
2006814108	CYOR	CYNOPS	ORIENTALIS	CHINESE DWARF	NEWT	LIV	2,450	NO	HK	HK	T	C	C	C	10/1/06	10/1/06	I	LA	DOLPHIN INTERNATIONAL
2006814108	PACB	PACHYTRITON	BREVIPES	TSITOU	NEWT	LIV	200	NO	HK	HK	T	C	C	C	10/1/06	10/1/06	I	LA	DOLPHIN INTERNATIONAL
2006814153	TRIH	TRITURUS	HONGKONGENSIS	ALPINE	NEWT	LIV	225	NO	HK	HK	T	W	C	C	10/1/06	10/1/06	I	LA	AQUACO, INC.
2006815725	PHNG	PARAMESOTRITON	HONGKONGENSIS	HONG KONG WARTY	NEWT	LIV	1,600	NO	HK	HK	T	C	C	C	10/3/06	10/3/06	I	TP	5-D TROPICAL, INC.
2006816696	BODO	BOLITOGLOSSA	DOFLEINI	ALTA VERAPAZ	SALAMANDER	LIV	17	NO	GT	GT	T	W	C	C	7/17/06	7/16/06	I	MI	DHAR TROPICAL LLC
2006817906	CYPY	CYNOPS	PYRRHOGASTER	JAPANESE FIREBELLY	NEWT	LIV	60	NO	HK	HK	T	W	C	C	8/24/06	8/19/06	I	NY	ERDA INCORPORATED
2006818323	CYOR	CYNOPS	ORIENTALIS	CHINESE DWARF	NEWT	LIV	750	NO	HK	HK	T	C	C	C	10/7/06	10/7/06	I	LA	DOLPHIN INTERNATIONAL
2006818369	TRIT	TRITURUS	SPECIES		NEWT	LIV	580	NO	HK	HK	T	W	C	C	10/7/06	10/7/06	I	LA	GLOBAL AQUATIC CONSULTING
2006818446	CYOR	CYNOPS	ORIENTALIS	CHINESE DWARF	NEWT	LIV	600	NO	HK	HK	T	C	C	C	10/8/06	10/8/06	I	LA	DOLPHIN INTERNATIONAL
2006818446	PACB	PACHYTRITON	BREVIPES	TSITOU	NEWT	LIV	200	NO	HK	HK	T	C	C	C	10/8/06	10/8/06	I	LA	DOLPHIN INTERNATIONAL
2006818555	CYPY	CYNOPS	PYRRHOGASTER	JAPANESE FIREBELLY	NEWT	LIV	5	NO	HK	HK	T	W	C	C	9/5/06	9/3/06	I	NY	TRANSSHIP DISCOUNTS LIMITED
2006818555	PACB	PACHYTRITON	BREVIPES	TSITOU	NEWT	LIV	75	NO	HK	HK	T	W	C	C	9/5/06	9/3/06	I	NY	TRANSSHIP DISCOUNTS LIMITED
2006818721	CYPY	CYNOPS	PYRRHOGASTER	JAPANESE FIREBELLY	NEWT	LIV	150	NO	CN	CN	T	C	C	C	9/1/06	8/27/06	I	NY	FISH MART INCORPORATED
2006818759	TRIH	TRITURUS	HONGKONGENSIS	ALPINE	NEWT	LIV	50	NO	HK	HK	T	W	C	C	8/21/06	8/21/06	I	NY	INTERNATIONAL PET RESOURCES , LLC
2006820828	CYOR	CYNOPS	ORIENTALIS	CHINESE DWARF	NEWT	LIV	200	NO	CN	CN	T	F	C	C	9/25/06	5/9/06	I	NY	MIN HUA ENTERPRISE, INC.
2006821046	CYPY	CYNOPS	PYRRHOGASTER	JAPANESE FIREBELLY	NEWT	LIV	450	NO	HK	HK	T	W	C	C	9/12/06	9/9/06	I	NY	ERDA INCORPORATED
2006821427	CYPY	CYNOPS	PYRRHOGASTER	JAPANESE FIREBELLY	NEWT	LIV	540	NO	HK	HK	T	W	C	C	8/15/06	8/12/06	I	NY	ERDA INCORPORATED
2006821444	CYPY	CYNOPS	PYRRHOGASTER	JAPANESE FIREBELLY	NEWT	LIV	100	NO	CN	CN	T	C	C	C	9/15/06	9/10/06	I	NY	FISH MART INCORPORATED
2006821476	CYPY	CYNOPS	PYRRHOGASTER	JAPANESE FIREBELLY	NEWT	LIV	150	NO	HK	HK	T	W	C	C	9/12/06	9/10/06	I	NY	TRANSSHIP DISCOUNTS LIMITED
2006821476	PACB	PACHYTRITON	BREVIPES	TSITOU	NEWT	LIV	60	NO	HK	HK	T	W	C	C	9/12/06	9/10/06	I	NY	TRANSSHIP DISCOUNTS LIMITED
2006821630	CYPY	CYNOPS	PYRRHOGASTER	JAPANESE FIREBELLY	NEWT	LIV	510	NO	HK	HK	T	W	C	C	9/20/06	9/16/06	I	NY	ERDA INCORPORATED
2006821642	CYPY	CYNOPS	PYRRHOGASTER	JAPANESE FIREBELLY	NEWT	LIV	350	NO	HK	HK	T	W	C	C	8/30/06	8/27/06	I	NY	TRANSSHIP DISCOUNTS LIMITED
2006821700	CYPY	CYNOPS	PYRRHOGASTER	JAPANESE FIREBELLY	NEWT	LIV	50	NO	HK	HK	T	W	C	C	6/7/06	6/4/06	I	NY	TRANSSHIP DISCOUNTS LIMITED
2006821700	PACB	PACHYTRITON	BREVIPES	TSITOU	NEWT	LIV	30	NO	HK	HK	T	W	C	C	6/7/06	6/4/06	I	NY	TRANSSHIP DISCOUNTS LIMITED
2006821882	CYOR	CYNOPS	ORIENTALIS	CHINESE DWARF	NEWT	LIV	2,000	NO	CN	CN	T	W	C	C	10/13/06	10/13/06	I	LA	Z IMPORTS INC.
2006822306	CYOR	CYNOPS	ORIENTALIS	CHINESE DWARF	NEWT	LIV	500	NO	HK	HK	T	C	C	C	10/14/06	10/14/06	I	LA	DOLPHIN INTERNATIONAL
2006822316	TRIT	TRITURUS	SPECIES		NEWT	LIV	1,600	NO	HK	HK	T	W	C	C	10/14/06	10/14/06	I	LA	GLOBAL AQUATIC CONSULTING
2006822364	TRIH	TRITURUS	HONGKONGENSIS	ALPINE	NEWT	LIV	300	NO	HK	HK	T	W	C	C	10/14/06	10/15/06	I	LA	AQUACO, INC.
2006822425	CYOR	CYNOPS	ORIENTALIS	CHINESE DWARF	NEWT	LIV	750	NO	HK	HK	T	C	C	C	10/15/06	10/15/06	I	LA	DOLPHIN INTERNATIONAL
2006822425	PACB	PACHYTRITON	BREVIPES	TSITOU	NEWT	LIV	200	NO	HK	HK	T	C	C	C	10/15/06	10/15/06	I	LA	DOLPHIN INTERNATIONAL
2006822441	TRIT	TRITURUS	SPECIES		NEWT	LIV	200	NO	HK	HK	T	W	C	C	10/15/06	10/15/06	I	LA	AQUATIC CONNECTION
2006825951	SASA	SALAMANDRA	SALAMANDRA	EUROPEAN FIRE	SALAMANDER	LIV	101	NO	GB	GB	T	C	C	C	10/19/06	10/19/06	I	MI	TWO AMIGOS IMPORT & EXPORT, INC.
2006826206	CYOR	CYNOPS	ORIENTALIS	CHINESE DWARF	NEWT	LIV	3,000	NO	CN	CN	T	W	C	C	10/20/06	10/20/06	I	LA	Z IMPORTS INC.
2006826600	CYOR	CYNOPS	ORIENTALIS	CHINESE DWARF	NEWT	LIV	250	NO	HK	HK	T	C	C	C	10/21/06	10/21/06	I	LA	DOLPHIN INTERNATIONAL
2006826610	TRIT	TRITURUS	SPECIES		NEWT	LIV	600	NO	HK	HK	T	W	C	C	10/21/06	10/21/06	I	LA	GLOBAL AQUATIC CONSULTING
2006826709	CYOR	CYNOPS	ORIENTALIS	CHINESE DWARF	NEWT	LIV	1,200	NO	HK	HK	T	C	C	C	10/22/06	10/22/06	I	LA	DOLPHIN INTERNATIONAL
2006826709	PACB	PACHYTRITON	BREVIPES	TSITOU	NEWT	LIV	300	NO	HK	HK	T	C	C	C	10/22/06	10/22/06	I	LA	DOLPHIN INTERNATIONAL
2006826735	TRIT	TRITURUS	SPECIES		NEWT	LIV	25	NO	SG	SG	T	W	C	C	10/22/06	10/22/06	I	LA	TROPICAL FISH CONSOLIDATORS LTD.
2006826737	TRIH	TRITURUS	HONGKONGENSIS	ALPINE	NEWT	LIV	500	NO	HK	HK	T	W	C	C	10/22/06	10/22/06	I	LA	AQUACO, INC.
2006826742	TRIT	TRITURUS	SPECIES		NEWT	LIV	200	NO	HK	HK	T	W	C	C	10/22/06	10/22/06	I	LA	AQUATIC CONNECTION
2006827853	TRIH	TRITURUS	HONGKONGENSIS	ALPINE	NEWT	LIV	1,600	NO	HK	HK	T	C	C	C	10/24/06	10/24/06	I	TP	5-D TROPICAL, INC.

2006829403	CYOR	CYNOPS	ORIENTALIS	CHINESE DWARF	NEWT	LIV	3,000	NO	CN	CN	T	W	C	C	10/27/06	10/27/06	I	LA	Z IMPORTS INC.
2006829777	CYOR	CYNOPS	ORIENTALIS	CHINESE DWARF	NEWT	LIV	500	NO	HK	HK	T	C	C	C	10/28/06	10/28/06	I	LA	DOLPHIN INTERNATIONAL
2006829837	TRIT	TRITURUS	SPECIES		NEWT	LIV	1,200	NO	HK	HK	T	W	C	C	10/28/06	10/28/06	I	LA	GLOBAL AQUATIC CONSULTING
2006829877	CYOR	CYNOPS	ORIENTALIS	CHINESE DWARF	NEWT	LIV	1,350	NO	HK	HK	T	C	C	C	10/29/06	10/29/06	I	LA	DOLPHIN INTERNATIONAL
2006829877	PACB	PACHYTRITON	BREVIPES	TSITOU	NEWT	LIV	300	NO	HK	HK	T	C	C	C	10/29/06	10/29/06	I	LA	DOLPHIN INTERNATIONAL
2006829900	TRIH	TRITURUS	HONGKONGENSIS	ALPINE	NEWT	LIV	150	NO	HK	HK	T	W	C	C	10/29/06	10/29/06	I	LA	AQUACO, INC.
2006829921	TRIT	TRITURUS	SPECIES		NEWT	LIV	200	NO	HK	HK	T	W	C	C	10/29/06	10/29/06	I	LA	AQUATIC CONNECTION
2006829939	CNP?	CYNOPS	SPECIES	FIREBELLY	NEWT	LIV	400	NO	HK	HK	T	C	C	C	10/30/06	10/30/06	I	AT	SUN PET LTD.
2006833135	TRIT	TRITURUS	SPECIES		NEWT	LIV	400	NO	HK	HK	T	W	C	C	11/4/06	11/4/06	I	LA	GLOBAL AQUATIC CONSULTING
2006833196	TRIH	TRITURUS	HONGKONGENSIS	ALPINE	NEWT	LIV	150	NO	HK	HK	T	W	C	C	11/5/06	11/5/06	I	LA	AQUACO, INC.
2006833220	CYOR	CYNOPS	ORIENTALIS	CHINESE DWARF	NEWT	LIV	600	NO	HK	HK	T	C	C	C	11/5/06	11/5/06	I	LA	DOLPHIN INTERNATIONAL
2006833220	PACB	PACHYTRITON	BREVIPES	TSITOU	NEWT	LIV	100	NO	HK	HK	T	C	C	C	11/5/06	11/5/06	I	LA	DOLPHIN INTERNATIONAL
2006835196	CYPY	CYNOPS	PYRRHOGASTER	JAPANESE FIREBELLY	NEWT	LIV	314	NO	HK	HK	T	W	C	C	9/7/06	9/2/06	I	NY	ERDA INCORPORATED
2006836000	CNP?	CYNOPS	SPECIES	FIREBELLY	NEWT	LIV	210	NO	HK	HK	T	W	C	C	10/12/06	10/7/06	I	NY	ERDA INCORPORATED
2006836360	SASA	SALAMANDRA	SALAMANDRA	EUROPEAN FIRE	SALAMANDER	LIV	116	NO	GB	GB	T	C	C	C	11/11/06	11/11/06	I	MI	TWO AMIGOS IMPORT & EXPORT, INC.
2006836360	TRIT	TRITURUS	SPECIES		NEWT	LIV	24	NO	GB	GB	T	C	C	C	11/11/06	11/11/06	I	MI	TWO AMIGOS IMPORT & EXPORT, INC.
2006836407	CYOR	CYNOPS	ORIENTALIS	CHINESE DWARF	NEWT	LIV	250	NO	HK	HK	T	C	C	C	11/11/06	11/11/06	I	LA	DOLPHIN INTERNATIONAL
2006836471	CYOR	CYNOPS	ORIENTALIS	CHINESE DWARF	NEWT	LIV	1,100	NO	HK	HK	T	C	C	C	11/12/06	11/12/06	I	LA	DOLPHIN INTERNATIONAL
2006836472	CYOR	CYNOPS	ORIENTALIS	CHINESE DWARF	NEWT	LIV	350	NO	HK	HK	T	C	C	C	11/12/06	11/12/06	I	LA	DOLPHIN INTERNATIONAL
2006836543	CNP?	CYNOPS	SPECIES	FIREBELLY	NEWT	LIV	400	NO	HK	HK	T	C	C	C	11/13/06	11/13/06	I	AT	SUN PET LTD.
2006836595	CYPY	CYNOPS	PYRRHOGASTER	JAPANESE FIREBELLY	NEWT	LIV	450	NO	HK	HK	T	W	C	C	10/4/06	9/30/06	I	NY	ERDA INCORPORATED
2006837000	CYPY	CYNOPS	PYRRHOGASTER	JAPANESE FIREBELLY	NEWT	LIV	200	NO	HK	HK	T	W	C	C	9/26/06	9/24/06	I	NY	TRANSSHIP DISCOUNTS LIMITED
2006837481	CYPY	CYNOPS	PYRRHOGASTER	JAPANESE FIREBELLY	NEWT	LIV	50	NO	HK	HK	T	W	C	C	10/2/06	10/1/06	I	NY	TRANSSHIP DISCOUNTS LIMITED
2006838158	CYOR	CYNOPS	ORIENTALIS	CHINESE DWARF	NEWT	LIV	40	NO	SG	SG	T	W	C	C	9/17/06	9/17/06	I	NY	INTERNATIONAL PET RESOURCES , LLC
2006838201	CNP?	CYNOPS	SPECIES	FIREBELLY	NEWT	LIV	200	NO	CN	CN	T	C	C	C	9/27/06	9/24/06	I	NY	FISH MART INCORPORATED
2006839856	CYPY	CYNOPS	PYRRHOGASTER	JAPANESE FIREBELLY	NEWT	LIV	2,200	NO	JP	JP	T	C	C	C	11/16/06	11/16/06	I	DF	U.S. GLOBAL EXOTICS, INC.
2006839856	CNP?	CYNOPS	SPECIES	FIREBELLY	NEWT	LIV	510	NO	JP	JP	T	C	C	C	11/16/06	11/16/06	I	DF	U.S. GLOBAL EXOTICS, INC.
2006840571	CYOR	CYNOPS	ORIENTALIS	CHINESE DWARF	NEWT	LIV	3,000	NO	CH	CN	T	W	C	C	11/17/06	11/17/06	I	LA	Z IMPORTS INC.
2006841130	TRIH	TRITURUS	HONGKONGENSIS	ALPINE	NEWT	LIV	50	NO	HK	HK	T	W	C	C	11/18/06	11/19/06	I	LA	AQUACO, INC.
2006841175	CYOR	CYNOPS	ORIENTALIS	CHINESE DWARF	NEWT	LIV	850	NO	HK	HK	T	C	C	C	11/19/06	11/19/06	I	LA	DOLPHIN INTERNATIONAL
2006841175	PACB	PACHYTRITON	BREVIPES	TSITOU	NEWT	LIV	500	NO	HK	HK	T	C	C	C	11/19/06	11/19/06	I	LA	DOLPHIN INTERNATIONAL
2006842447	CYPY	CYNOPS	PYRRHOGASTER	JAPANESE FIREBELLY	NEWT	LIV	60	NO	HK	HK	T	W	C	C	10/19/06	10/15/06	I	NY	TRANSSHIP DISCOUNTS LIMITED
2006842545	CYPY	CYNOPS	PYRRHOGASTER	JAPANESE FIREBELLY	NEWT	LIV	625	NO	HK	HK	T	W	C	C	4/11/06	4/8/06	I	NY	ERDA INCORPORATED
2006843164	CYPY	CYNOPS	PYRRHOGASTER	JAPANESE FIREBELLY	NEWT	LIV	400	NO	HK	HK	T	W	C	C	11/2/06	10/28/06	I	NY	ERDA INCORPORATED
2006844159	CYOR	CYNOPS	ORIENTALIS	CHINESE DWARF	NEWT	LIV	3,000	NO	CH	CN	T	W	C	C	11/24/06	11/24/06	I	LA	Z IMPORTS INC.
2006844270	CYOR	CYNOPS	ORIENTALIS	CHINESE DWARF	NEWT	LIV	500	NO	HK	HK	T	C	C	C	11/25/06	11/25/06	I	LA	DOLPHIN INTERNATIONAL
2006844270	PACB	PACHYTRITON	BREVIPES	TSITOU	NEWT	LIV	100	NO	HK	HK	T	C	C	C	11/25/06	11/25/06	I	LA	DOLPHIN INTERNATIONAL
2006844308	TRIT	TRITURUS	SPECIES		NEWT	LIV	200	NO	HK	HK	T	C	C	C	11/25/06	11/25/06	I	LA	GLOBAL AQUATIC CONSULTING
2006844378	TRIH	TRITURUS	HONGKONGENSIS	ALPINE	NEWT	LIV	450	NO	HK	HK	T	W	C	C	11/26/06	11/26/06	I	LA	AQUACO, INC.
2006844382	CYOR	CYNOPS	ORIENTALIS	CHINESE DWARF	NEWT	LIV	1,100	NO	HK	HK	T	C	C	C	11/26/06	11/26/06	I	LA	DOLPHIN INTERNATIONAL
2006844382	PACB	PACHYTRITON	BREVIPES	TSITOU	NEWT	LIV	500	NO	HK	HK	T	C	C	C	11/26/06	11/26/06	I	LA	DOLPHIN INTERNATIONAL
2006846803	PDE?	PSEUDOEURYCEA	SPECIES	FALSE BROOK	SALAMANDER	LIV	6	NO	MX	MX	S	W	C	C	9/5/06	9/5/06	I	LR	SAN ANTONIO ZOOLOGICAL GARDENS
2006847449	TRIH	TRITURUS	HONGKONGENSIS	ALPINE	NEWT	LIV	222	NO	HK	HK	T	C	C	C	5/16/06	5/15/06	I	NY	TRI-COUNTY TROPICALS, INC.
2006848571	CYOR	CYNOPS	ORIENTALIS	CHINESE DWARF	NEWT	LIV	3,000	NO	CH	CN	T	W	C	C	12/1/06	12/1/06	I	LA	Z IMPORTS INC.
2006849043	SASA	SALAMANDRA	SALAMANDRA	EUROPEAN FIRE	SALAMANDER	LIV	66	NO	DE	DE	P	C	C	C	11/15/06	11/15/06	I	SF	Exemptions 6 and 7(C)
2006849128	TRIT	TRITURUS	SPECIES		NEWT	LIV	600	NO	VS	HK	T	W	C	C	12/2/06	12/2/06	I	LA	GLOBAL AQUATIC CONSULTING
2006849168	TRIH	TRITURUS	HONGKONGENSIS	ALPINE	NEWT	LIV	100	NO	HK	HK	T	W	C	C	12/3/06	12/3/06	I	LA	AQUACO, INC.
2006849195	CYOR	CYNOPS	ORIENTALIS	CHINESE DWARF	NEWT	LIV	835	NO	HK	HK	T	C	C	C	12/3/06	12/3/06	I	LA	DOLPHIN INTERNATIONAL
2006849195	PACB	PACHYTRITON	BREVIPES	TSITOU	NEWT	LIV	500	NO	HK	HK	T	C	C	C	12/3/06	12/3/06	I	LA	DOLPHIN INTERNATIONAL
2006849226	CNP?	CYNOPS	SPECIES	FIREBELLY	NEWT	LIV	400	NO	HK	HK	T	C	C	C	12/4/06	12/4/06	I	AT	SUN PET LTD.
2006849407	CYPY	CYNOPS	PYRRHOGASTER	JAPANESE FIREBELLY	NEWT	LIV	150	NO	HK	HK	T	W	C	C	10/11/06	10/8/06	I	NY	TRANSSHIP DISCOUNTS LIMITED
2006850175	CYPY	CYNOPS	PYRRHOGASTER	JAPANESE FIREBELLY	NEWT	LIV	275	NO	HK	HK	T	C	C	C	10/18/06	10/14/06	I	NY	ERDA INCORPORATED
2006850814	PACB	PACHYTRITON	BREVIPES	TSITOU	NEWT	LIV	60	NO	HK	HK	T	W	C	C	7/5/06	7/2/06	I	NY	TRANSSHIP DISCOUNTS LIMITED
2006851400	CNP?	CYNOPS	SPECIES	FIREBELLY	NEWT	LIV	200	NO	CN	CN	T	C	C	C	10/26/06	10/22/06	I	NY	FISH MART INCORPORATED
2006851615	CYPY	CYNOPS	PYRRHOGASTER	JAPANESE FIREBELLY	NEWT	LIV	150	NO	HK	HK	T	C	C	C	10/24/06	10/21/06	I	NY	ERDA INC
2006851647	CYPY	CYNOPS	PYRRHOGASTER	JAPANESE FIREBELLY	NEWT	LIV	150	NO	HK	HK	T	C	C	C	7/27/06	7/22/06	I	NY	ERDA INCORPORATED
2006851915	CYPY	CYNOPS	PYRRHOGASTER	JAPANESE FIREBELLY	NEWT	LIV	50	NO	HK	HK	T	W	C	C	7/12/06	7/9/06	I	NY	TRANSSHIP DISCOUNTS LIMITED
2006852093	CYPY	CYNOPS	PYRRHOGASTER	JAPANESE FIREBELLY	NEWT	LIV	60	NO	HK	HK	T	W	C	C	8/2/06	7/29/06	I	NY	ERDA INCORPORATED
2006852565	PHNG	PARAMESOTRITON	HONGKONGENSIS	HONG KONG WARTY	NEWT	LIV	300	NO	HK	HK	T	C	C	C	12/7/06	12/7/06	I	TP	SEGREST FARMS
2006853011	CYOR	CYNOPS	ORIENTALIS	CHINESE DWARF	NEWT	LIV	3,000	NO	CN	CN	T	W	C	C	12/8/06	12/8/06	I	LA	Z IMPORTS INC.

2006853265	CYOR	CYNOPS	ORIENTALIS	CHINESE DWARF	NEWT	LIV	500	NO	HK	HK	T	C	C	C	12/9/06	12/9/06	I	LA	DOLPHIN INTERNATIONAL
2006853284	TRIT	TRITURUS	SPECIES		NEWT	LIV	800	NO	HK	HK	T	C	C	C	12/9/06	12/9/06	I	LA	GLOBAL AQUATIC CONSULTING
2006853384	CYOR	CYNOPS	ORIENTALIS	CHINESE DWARF	NEWT	LIV	850	NO	HK	HK	T	C	C	C	12/10/06	12/10/06	I	LA	DOLPHIN INTERNATIONAL
2006853384	PACB	PACHYTRITON	BREVIPES	TSITOU	NEWT	LIV	100	NO	HK	HK	T	C	C	C	12/10/06	12/10/06	I	LA	DOLPHIN INTERNATIONAL
2006853385	TRIH	TRITURUS	HONGKONGENSIS	ALPINE	NEWT	LIV	100	NO	HK	HK	T	W	C	C	12/10/06	12/10/06	I	LA	AQUACO, INC.
2006856729	PHNG	PARAMESOTRITON	HONGKONGENSIS	HONG KONG WARTY	NEWT	LIV	300	NO	HK	HK	T	C	C	C	12/14/06	12/14/06	I	TP	SEGREST FARMS
2006858244	CYOR	CYNOPS	ORIENTALIS	CHINESE DWARF	NEWT	LIV	750	NO	HK	HK	T	C	C	C	12/16/06	12/16/06	I	LA	DOLPHIN INTERNATIONAL
2006858302	TRIT	TRITURUS	SPECIES		NEWT	LIV	800	NO	HK	HK	T	C	C	C	12/16/06	12/16/06	I	LA	GLOBAL AQUATIC CONSULTING
2006858323	TRIH	TRITURUS	HONGKONGENSIS	ALPINE	NEWT	LIV	150	NO	HK	HK	T	W	C	C	12/16/06	12/17/06	I	LA	AQUACO, INC.
2006858362	CYOR	CYNOPS	ORIENTALIS	CHINESE DWARF	NEWT	LIV	750	NO	HK	HK	T	C	C	C	12/17/06	12/17/06	I	LA	DOLPHIN INTERNATIONAL
2006858362	PACB	PACHYTRITON	BREVIPES	TSITOU	NEWT	LIV	600	NO	HK	HK	T	C	C	C	12/17/06	12/17/06	I	LA	DOLPHIN INTERNATIONAL
2006860638	CYOR	CYNOPS	ORIENTALIS	CHINESE DWARF	NEWT	LIV	500	NO	HK	HK	T	C	C	C	12/20/06	12/20/06	I	LA	DOLPHIN INTERNATIONAL
2006863175	CYOR	CYNOPS	ORIENTALIS	CHINESE DWARF	NEWT	LIV	500	NO	HK	HK	T	C	C	C	12/26/06	12/26/06	I	LA	DOLPHIN INTERNATIONAL
2006863175	PACB	PACHYTRITON	BREVIPES	TSITOU	NEWT	LIV	100	NO	HK	HK	T	C	C	C	12/26/06	12/26/06	I	LA	DOLPHIN INTERNATIONAL
2006863961	PHNG	PARAMESOTRITON	HONGKONGENSIS	HONG KONG WARTY	NEWT	LIV	1,600	NO	HK	HK	T	C	C	C	12/27/06	12/27/06	I	TP	5-D TROPICAL, INC.
2006864553	CYPY	CYNOPS	PYRRHOGASTER	JAPANESE FIREBELLY	NEWT	LIV	200	NO	HK	HK	T	W	C	C	9/26/06	9/23/06	I	NY	ERDA INCORPORATED
2006865599	CYOR	CYNOPS	ORIENTALIS	CHINESE DWARF	NEWT	LIV	250	NO	HK	HK	T	C	C	C	12/30/06	12/30/06	I	LA	DOLPHIN INTERNATIONAL
2006865619	CYOR	CYNOPS	ORIENTALIS	CHINESE DWARF	NEWT	LIV	840	NO	HK	HK	T	C	C	C	12/31/06	12/31/06	I	LA	DOLPHIN INTERNATIONAL
2006865619	PACB	PACHYTRITON	BREVIPES	TSITOU	NEWT	LIV	300	NO	HK	HK	T	C	C	C	12/31/06	12/31/06	I	LA	DOLPHIN INTERNATIONAL
2007100526	CYOR	CYNOPS	ORIENTALIS	CHINESE DWARF	NEWT	LIV	3,000	NO	CN	CN	T	W	C	C	9/14/07	9/14/07	I	LA	Z IMPORTS INC.
2007101028	CYOR	CYNOPS	ORIENTALIS	CHINESE DWARF	NEWT	LIV	500	NO	HK	HK	T	C	C	C	9/15/07	9/15/07	I	LA	DOLPHIN INTERNATIONAL
2007101097	CYOR	CYNOPS	ORIENTALIS	CHINESE DWARF	NEWT	LIV	750	NO	HK	HK	T	C	C	C	9/15/07	9/16/07	I	LA	DOLPHIN INTERNATIONAL
2007101097	PACB	PACHYTRITON	BREVIPES	TSITOU	NEWT	LIV	100	NO	HK	HK	T	C	C	C	9/15/07	9/16/07	I	LA	DOLPHIN INTERNATIONAL
2007101128	TRIH	TRITURUS	HONGKONGENSIS	ALPINE	NEWT	LIV	475	NO	VS	HK	T	W	C	C	9/15/07	9/16/07	I	LA	AQUACO, INC.
2007101131	CYOR	CYNOPS	ORIENTALIS	CHINESE DWARF	NEWT	LIV	2,100	NO	CN	CN	T	W	C	C	9/15/07	9/16/07	I	LA	AQUA-NAUTIC SPECIALIST
2007101156	PLAB	PACHYTRITON	LABIATUS	UNTERSTEIN'S	NEWT	LIV	80	NO	CN	CN	T	C	C	C	9/17/07	9/17/07	I	AT	SUN PET LTD.
2007101868	CYPY	CYNOPS	PYRRHOGASTER	JAPANESE FIREBELLY	NEWT	LIV	300	NO	HK	HK	T	W	C	C	4/17/07	4/15/07	I	NY	TRANSSHIP DISCOUNTS LIMITED
2007101875	CYPY	CYNOPS	PYRRHOGASTER	JAPANESE FIREBELLY	NEWT	LIV	35	NO	HK	HK	T	W	C	C	4/17/07	4/14/07	I	NY	ERDA INCORPORATED
2007102108	TRIH	TRITURUS	HONGKONGENSIS	ALPINE	NEWT	LIV	1,460	NO	HK	HK	T	C	C	C	9/18/07	9/19/07	I	TP	5-D TROPICAL, INC.
2007102459	TRIH	TRITURUS	HONGKONGENSIS	ALPINE	NEWT	LIV	400	NO	HK	HK	T	W	C	C	4/23/07	4/15/07	I	NY	INTERNATIONAL PET RESOURCES , LLC
2007103263	CYPY	CYNOPS	PYRRHOGASTER	JAPANESE FIREBELLY	NEWT	LIV	50	NO	HK	HK	T	W	C	C	6/24/07	6/24/07	I	NY	TRANSSHIP DISCOUNTS LIMITED
2007103263	CYPY	CYNOPS	PYRRHOGASTER	JAPANESE FIREBELLY	NEWT	LIV	540	NO	HK	HK	T	W	C	C	6/24/07	6/24/07	I	NY	TRANSSHIP DISCOUNTS LIMITED
2007103326	CYPY	CYNOPS	PYRRHOGASTER	JAPANESE FIREBELLY	NEWT	LIV	300	NO	HK	HK	T	W	C	C	5/30/07	5/27/07	I	NY	TRANSSHIP DISCOUNTS LIMITED
2007103370	CYPY	CYNOPS	PYRRHOGASTER	JAPANESE FIREBELLY	NEWT	LIV	220	NO	HK	HK	T	W	C	C	8/13/07	8/11/07	I	NY	ERDA INCORPORATED
2007103384	CYPY	CYNOPS	PYRRHOGASTER	JAPANESE FIREBELLY	NEWT	LIV	150	NO	HK	HK	T	W	C	C	8/13/07	8/12/07	I	NY	TRANSSHIP DISCOUNTS LIMITED
2007103597	CYPY	CYNOPS	PYRRHOGASTER	JAPANESE FIREBELLY	NEWT	LIV	400	NO	HK	HK	T	W	C	C	9/19/07	9/16/07	I	NY	Transship Discounts Ltd.
2007103775	CYPY	CYNOPS	PYRRHOGASTER	JAPANESE FIREBELLY	NEWT	LIV	150	NO	HK	HK	T	W	C	C	7/24/07	7/22/07	I	NY	TRANSSHIP DISCOUNTS LIMITED
2007103784	CYPY	CYNOPS	PYRRHOGASTER	JAPANESE FIREBELLY	NEWT	LIV	290	NO	HK	HK	T	W	C	C	7/23/07	7/21/07	I	NY	ERDA INCORPORATED
2007103793	CYPY	CYNOPS	PYRRHOGASTER	JAPANESE FIREBELLY	NEWT	LIV	100	NO	HK	HK	T	W	C	C	6/4/07	6/3/07	I	NY	TRANSSHIP DISCOUNTS LIMITED
2007103793	CYPY	CYNOPS	PYRRHOGASTER	JAPANESE FIREBELLY	NEWT	LIV	90	NO	HK	HK	T	W	C	C	6/4/07	6/3/07	I	NY	TRANSSHIP DISCOUNTS LIMITED
2007103815	CYPY	CYNOPS	PYRRHOGASTER	JAPANESE FIREBELLY	NEWT	LIV	515	NO	HK	HK	T	W	C	C	6/4/07	6/2/07	I	NY	ERDA INCORPORATED
2007104905	TYVE	TYLOTOTRITON	VERRUCOSUS	CROCODILE	NEWT	LIV	20	NO	HK	HK	T	C	C	C	9/21/07	9/21/07	I	SF	A.F. Exotics
2007105229	PACB	PACHYTRITON	BREVIPES	TSITOU	NEWT	LIV	100	NO	HK	HK	T	C	C	C	9/22/07	9/22/07	I	LA	DOLPHIN INTERNATIONAL
2007105244	TRIT	TRITURUS	SPECIES		NEWT	LIV	400	NO	HK	HK	T	C	C	C	9/22/07	9/23/07	I	LA	AQUATIC CONNECTION
2007105247	CYOR	CYNOPS	ORIENTALIS	CHINESE DWARF	NEWT	LIV	600	NO	CN	CN	T	W	C	C	9/22/07	9/23/07	I	LA	AQUA-NAUTIC SPECIALIST
2007105251	TRIH	TRITURUS	HONGKONGENSIS	ALPINE	NEWT	LIV	175	NO	HK	HK	T	W	C	C	9/22/07	9/23/07	I	LA	AQUACO, INC.
2007105277	CYOR	CYNOPS	ORIENTALIS	CHINESE DWARF	NEWT	LIV	800	NO	HK	HK	T	C	C	C	9/22/07	9/23/07	I	LA	DOLPHIN INTERNATIONAL
2007105277	PACB	PACHYTRITON	BREVIPES	TSITOU	NEWT	LIV	100	NO	HK	HK	T	C	C	C	9/22/07	9/23/07	I	LA	DOLPHIN INTERNATIONAL
2007105314	CNP?	CYNOPS	SPECIES	FIREBELLY	NEWT	LIV	300	NO	CN	CN	T	C	C	C	9/24/07	9/24/07	I	AT	SUN PET LTD.
2007105702	BODO	BOLITOGLOSSA	DOFLEINI	ALTA VERAPAZ	SALAMANDER	LIV	36	NO	GT	GT	T	W	C	C	9/24/07	9/24/07	I	MI	D.H.A.R. TROPICALS LLC
2007106613	CYPY	CYNOPS	PYRRHOGASTER	JAPANESE FIREBELLY	NEWT	LIV	134	NO	HK	HK	T	W	C	C	9/26/07	9/23/07	I	NY	Transship Discounts Ltd.
2007106864	TYVE	TYLOTOTRITON	VERRUCOSUS	CROCODILE	NEWT	LIV	500	NO	CN	CN	T	W	C	C	9/26/07	9/26/07	I	LA	GOLDEN POND
2007107963	TRIT	TRITURUS	SPECIES		NEWT	LIV	52	NO	CA	CA	T	C	C	C	9/28/07	9/28/07	I	NY	Exemptions 6 and 7(C)
2007107963	NRR?	NEURERGUS	SPECIES		NEWT	LIV	4	NO	CA	CA	T	C	C	C	9/28/07	9/28/07	I	NY	Exemptions 6 and 7(C)
2007107963	PCAU	PARAMESOTRITON	CAUDOPUNCTATUS	GUIZHOU WARTY	NEWT	LIV	22	NO	CA	CA	T	C	C	C	9/28/07	9/28/07	I	NY	Exemptions 6 and 7(C)
2007107963	SASA	SALAMANDRA	SALAMANDRA	EUROPEAN FIRE	SALAMANDER	LIV	1	NO	CA	CA	T	C	C	C	9/28/07	9/28/07	I	NY	Exemptions 6 and 7(C)
2007107963	CNP?	CYNOPS	SPECIES	FIREBELLY	NEWT	LIV	6	NO	CA	CA	T	C	C	C	9/28/07	9/28/07	I	NY	Exemptions 6 and 7(C)
2007108362	CYOR	CYNOPS	ORIENTALIS	CHINESE DWARF	NEWT	LIV	4,000	NO	CN	CN	T	W	C	C	9/28/07	9/28/07	I	LA	Z IMPORTS INC.
2007108362	TYVE	TYLOTOTRITON	VERRUCOSUS	CROCODILE	NEWT	LIV	260	NO	CN	CN	T	W	C	C	9/28/07	9/28/07	I	LA	Z IMPORTS INC.
2007108482	CYOR	CYNOPS	ORIENTALIS	CHINESE DWARF	NEWT	LIV	500	NO	HK	HK	T	C	C	C	9/29/07	9/29/07	I	LA	DOLPHIN INTERNATIONAL

2007108482	PACB	PACHYTRITON	BREVIPES	TSITOU	NEWT	LIV	100	NO	HK	HK	T	C	C	C	9/29/07	9/29/07	I	LA	DOLPHIN INTERNATIONAL
2007108564	CYOR	CYNOPS	ORIENTALIS	CHINESE DWARF	NEWT	LIV	1,450	NO	HK	HK	T	C	C	C	9/29/07	9/30/07	I	LA	DOLPHIN INTERNATIONAL
2007108592	TRIH	TRITURUS	HONGKONGENSIS	ALPINE	NEWT	LIV	350	NO	HK	HK	T	W	C	C	9/30/07	9/30/07	I	LA	AQUACO, INC.
2007108594	CYOR	CYNOPS	ORIENTALIS	CHINESE DWARF	NEWT	LIV	300	NO	CN	CN	T	W	C	C	9/30/07	9/30/07	I	LA	AQUA-NAUTIC SPECIALIST
2007108654	CNP?	CYNOPS	SPECIES	FIREBELLY	NEWT	LIV	300	NO	CN	CN	T	C	C	C	10/1/07	10/1/07	I	AT	SUN PET LTD.
2007109832	CYPY	CYNOPS	PYRRHOGASTER	JAPANESE FIREBELLY	NEWT	LIV	210	NO	HK	HK	T	W	C	C	10/2/07	9/30/07	I	NY	Transship Discounts Ltd.
2007110063	CYOR	CYNOPS	ORIENTALIS	CHINESE DWARF	NEWT	LIV	300	NO	HK	HK	T	W	C	C	10/3/07	10/3/07	I	CH	AQUATICS, INC.
2007111628	CYOR	CYNOPS	ORIENTALIS	CHINESE DWARF	NEWT	LIV	500	NO	HK	HK	T	C	C	C	10/6/07	10/6/07	I	LA	DOLPHIN INTERNATIONAL
2007111723	CYOR	CYNOPS	ORIENTALIS	CHINESE DWARF	NEWT	LIV	1,000	NO	HK	HK	T	C	C	C	10/7/07	10/7/07	I	LA	DOLPHIN INTERNATIONAL
2007111723	PACB	PACHYTRITON	BREVIPES	TSITOU	NEWT	LIV	100	NO	HK	HK	T	C	C	C	10/7/07	10/7/07	I	LA	DOLPHIN INTERNATIONAL
2007111733	TRIH	TRITURUS	HONGKONGENSIS	ALPINE	NEWT	LIV	150	NO	HK	HK	T	W	C	C	10/7/07	10/7/07	I	LA	AQUACO, INC.
2007111735	TRIT	TRITURUS	SPECIES		NEWT	LIV	400	NO	HK	HK	T	C	C	C	10/7/07	10/7/07	I	LA	AQUATIC CONNECTION
2007111747	CYOR	CYNOPS	ORIENTALIS	CHINESE DWARF	NEWT	LIV	600	NO	HK	CN	T	W	C	C	10/7/07	10/7/07	I	LA	AQUA-NAUTIC SPECIALIST
2007111752	PLAB	PACHYTRITON	LABIATUS	UNTERSTEIN'S	NEWT	LIV	80	NO	CN	CN	T	C	C	C	10/8/07	10/8/07	I	AT	SUN PET LTD.
2007112937	CYPY	CYNOPS	PYRRHOGASTER	JAPANESE FIREBELLY	NEWT	LIV	160	NO	HK	HK	T	W	C	C	10/10/07	10/6/07	I	NY	Transship Discounts Ltd.
2007114194	CYOR	CYNOPS	ORIENTALIS	CHINESE DWARF	NEWT	LIV	3,000	NO	CN	CN	T	W	C	C	10/12/07	10/12/07	I	LA	Z IMPORTS INC.
2007114194	TYVE	TYLOTOTRITON	VERRUCOSUS	CROCODILE	NEWT	LIV	300	NO	CN	CN	T	W	C	C	10/12/07	10/12/07	I	LA	Z IMPORTS INC.
2007114415	CYOR	CYNOPS	ORIENTALIS	CHINESE DWARF	NEWT	LIV	250	NO	HK	HK	T	C	C	C	10/12/07	10/13/07	I	LA	DOLPHIN INTERNATIONAL
2007114590	TRIT	TRITURUS	SPECIES		NEWT	LIV	800	NO	HK	HK	T	W	C	C	10/13/07	10/13/07	I	LA	GLOBAL AQUATIC CONSULTING
2007114655	CYOR	CYNOPS	ORIENTALIS	CHINESE DWARF	NEWT	LIV	557	NO	CN	CN	T	W	C	C	10/13/07	10/14/07	I	LA	AQUA-NAUTIC SPECIALIST
2007114664	CYOR	CYNOPS	ORIENTALIS	CHINESE DWARF	NEWT	LIV	850	NO	HK	HK	T	C	C	C	10/13/07	10/14/07	I	LA	DOLPHIN INTERNATIONAL
2007115589	CYPY	CYNOPS	PYRRHOGASTER	JAPANESE FIREBELLY	NEWT	LIV	210	NO	HK	HK	T	W	C	C	10/16/07	10/14/07	I	NY	Transship Discounts Ltd.
2007117560	CYOR	CYNOPS	ORIENTALIS	CHINESE DWARF	NEWT	LIV	250	NO	HK	HK	T	C	C	C	10/19/07	10/20/07	I	LA	DOLPHIN INTERNATIONAL
2007117877	TRIH	TRITURUS	HONGKONGENSIS	ALPINE	NEWT	LIV	50	NO	HK	HK	T	W	C	C	10/20/07	10/21/07	I	LA	AQUACO, INC.
2007117880	CYOR	CYNOPS	ORIENTALIS	CHINESE DWARF	NEWT	LIV	2,000	NO	HK	HK	T	C	C	C	10/20/07	10/21/07	I	LA	DOLPHIN INTERNATIONAL
2007117880	PACB	PACHYTRITON	BREVIPES	TSITOU	NEWT	LIV	100	NO	HK	HK	T	C	C	C	10/20/07	10/21/07	I	LA	DOLPHIN INTERNATIONAL
2007117957	CNP?	CYNOPS	SPECIES	FIREBELLY	NEWT	LIV	300	NO	CN	CN	T	C	C	C	10/22/07	10/22/07	I	AT	SUN PET LTD.
2007118000	CYPY	CYNOPS	PYRRHOGASTER	JAPANESE FIREBELLY	NEWT	LIV	150	NO	HK	HK	T	W	C	C	5/1/07	4/29/07	I	NY	TRANSSHIP DISCOUNTS LIMITED
2007118000	NEW?	PACHYTRITON	SPECIES	CHINESE	NEWT	LIV	60	NO	HK	HK	T	W	C	C	5/1/07	4/29/07	I	NY	TRANSSHIP DISCOUNTS LIMITED
2007118102	CYPY	CYNOPS	PYRRHOGASTER	JAPANESE FIREBELLY	NEWT	LIV	200	NO	HK	HK	T	W	C	C	4/25/07	4/21/07	I	NY	ERDA INCORPORATED
2007118266	CYPY	CYNOPS	PYRRHOGASTER	JAPANESE FIREBELLY	NEWT	LIV	110	NO	HK	HK	T	W	C	C	10/22/07	10/21/07	I	NY	Transship Discounts Ltd.
2007118277	CYOR	CYNOPS	ORIENTALIS	CHINESE DWARF	NEWT	LIV	500	NO	CN	CN	T	W	C	C	10/22/07	10/22/07	I	LA	WORLD WIDE TRADING INC.
2007118277	PLAB	PACHYTRITON	LABIATUS	UNTERSTEIN'S	NEWT	LIV	500	NO	CN	CN	T	W	C	C	10/22/07	10/22/07	I	LA	WORLD WIDE TRADING INC.
2007118305	CYPY	CYNOPS	PYRRHOGASTER	JAPANESE FIREBELLY	NEWT	LIV	150	NO	HK	HK	T	W	C	C	5/14/07	5/13/07	I	NY	ERDA INCORPORATED
2007118321	CYPY	CYNOPS	PYRRHOGASTER	JAPANESE FIREBELLY	NEWT	LIV	210	NO	HK	HK	T	W	C	C	5/14/07	5/13/07	I	NY	TRANSSHIP DISCOUNTS LIMITED
2007118744	TYVE	TYLOTOTRITON	VERRUCOSUS	CROCODILE	NEWT	LIV	80	NO	SG	SG	T	W	C	C	3/21/07	3/4/07	I	NY	INTERNATIONAL PET RESOURCES , LLC
2007118821	NEW?	PACHYTRITON	SPECIES	CHINESE	NEWT	LIV	300	NO	CN	CN	T	C	C	C	10/23/07	10/23/07	I	SF	Golden Phoenix Fisheries, USA
2007119422	BODO	BOLITOGLOSSA	DOFLEINI	ALTA VERAPAZ	SALAMANDER	LIV	49	NO	GT	GT	T	W	C	C	10/24/07	9/2/07	I	MI	D.H.A.R. TROPICALS LLC
2007119532	CYPY	CYNOPS	PYRRHOGASTER	JAPANESE FIREBELLY	NEWT	LIV	250	NO	HK	HK	T	W	C	C	5/22/07	5/20/07	I	NY	TRANSSHIP DISCOUNTS LIMITED
2007120268	TRIH	TRITURUS	HONGKONGENSIS	ALPINE	NEWT	LIV	100	NO	HK	HK	T	W	C	C	3/21/07	3/2/07	I	NY	INTERNATIONAL PET RESOURCES , LLC
2007120955	TRIH	TRITURUS	HONGKONGENSIS	ALPINE	NEWT	LIV	1,600	NO	HK	HK	T	C	C	C	10/26/07	10/16/07	I	TP	5-D TROPICAL, INC.
2007121152	CYOR	CYNOPS	ORIENTALIS	CHINESE DWARF	NEWT	LIV	150	NO	HK	HK	T	W	C	C	6/26/07	6/23/07	I	NY	ERDA INCORPORATED
2007121575	CYOR	CYNOPS	ORIENTALIS	CHINESE DWARF	NEWT	LIV	600	NO	HK	HK	T	C	C	C	10/27/07	10/28/07	I	LA	DOLPHIN INTERNATIONAL
2007121623	CYOR	CYNOPS	ORIENTALIS	CHINESE DWARF	NEWT	LIV	1,297	NO	CN	CN	T	W	C	C	10/28/07	10/28/07	I	LA	AQUA-NAUTIC SPECIALIST
2007122978	CYPY	CYNOPS	PYRRHOGASTER	JAPANESE FIREBELLY	NEWT	LIV	150	NO	HK	HK	T	W	C	C	7/3/07	7/1/07	I	NY	TRANSSHIP DISCOUNTS LIMITED
2007122978	CYPY	CYNOPS	PYRRHOGASTER	JAPANESE FIREBELLY	NEWT	LIV	120	NO	HK	HK	T	W	C	C	7/3/07	7/1/07	I	NY	TRANSSHIP DISCOUNTS LIMITED
2007123000	CYPY	CYNOPS	PYRRHOGASTER	JAPANESE FIREBELLY	NEWT	LIV	300	NO	HK	HK	T	W	C	C	7/18/07	7/14/07	I	NY	ERDA INCORPORATED
2007123462	CYPY	CYNOPS	PYRRHOGASTER	JAPANESE FIREBELLY	NEWT	LIV	60	NO	HK	HK	T	W	C	C	7/18/07	7/15/07	I	NY	TRANSSHIP DISCOUNTS LIMITED
2007123604	NEBE	NECTURUS	BEYERI	GULF COAST	WATERDOG	LIV	10	NO	US	JP	T	C	C	C	5/23/07	5/23/07	I	MI	NAME REMOVED
2007123604	AMBY	AMBYSTOMA	SPECIES	MOLE	SALAMANDER	LIV	1	NO	US	JP	T	C	C	C	5/23/07	5/23/07	I	MI	NAME REMOVED
2007123752	CYOR	CYNOPS	ORIENTALIS	CHINESE DWARF	NEWT	LIV	150	NO	HK	HK	T	W	C	C	10/31/07	10/31/07	I	CH	AQUATICS, INC.
2007124765	CYOR	CYNOPS	ORIENTALIS	CHINESE DWARF	NEWT	LIV	5,000	NO	HK	CN	T	W	C	C	11/2/07	11/2/07	I	LA	Z IMPORTS INC.
2007124782	CYOR	CYNOPS	ORIENTALIS	CHINESE DWARF	NEWT	LIV	500	NO	CN	CN	T	W	C	C	11/2/07	11/2/07	I	LA	WORLD WIDE TRADING INC.
2007124782	PLAB	PACHYTRITON	LABIATUS	UNTERSTEIN'S	NEWT	LIV	500	NO	CN	CN	T	W	C	C	11/2/07	11/2/07	I	LA	WORLD WIDE TRADING INC.
2007125204	CYOR	CYNOPS	ORIENTALIS	CHINESE DWARF	NEWT	LIV	500	NO	HK	HK	T	C	C	C	11/3/07	11/3/07	I	LA	DOLPHIN INTERNATIONAL
2007125300	TRIH	TRITURUS	HONGKONGENSIS	ALPINE	NEWT	LIV	50	NO	HK	HK	T	W	C	C	11/3/07	11/4/07	I	LA	AQUACO, INC.
2007125308	CYOR	CYNOPS	ORIENTALIS	CHINESE DWARF	NEWT	LIV	600	NO	CN	CN	T	W	C	C	11/3/07	11/4/07	I	LA	AQUA-NAUTIC SPECIALIST
2007125309	CYOR	CYNOPS	ORIENTALIS	CHINESE DWARF	NEWT	LIV	1,000	NO	HK	HK	T	C	C	C	11/3/07	11/4/07	I	LA	DOLPHIN INTERNATIONAL
2007125309	PACB	PACHYTRITON	BREVIPES	TSITOU	NEWT	LIV	100	NO	HK	HK	T	C	C	C	11/3/07	11/4/07	I	LA	DOLPHIN INTERNATIONAL
2007125353	CNP?	CYNOPS	SPECIES	FIREBELLY	NEWT	LIV	300	NO	CN	CN	T	C	C	C	11/5/07	11/5/07	I	AT	SUN PET LTD.

2007126065	CYPY	CYNOPS	PYRRHOGASTER	JAPANESE FIREBELLY	NEWT	LIV	60	NO	HK	HK	T	W	C	C	11/5/07	11/4/07	I	NY	Transship Discounts Ltd.
2007126401	CYPY	CYNOPS	PYRRHOGASTER	JAPANESE FIREBELLY	NEWT	LIV	590	NO	HK	HK	T	W	C	C	6/12/07	6/9/07	I	NY	ERDA INCORPORATED
2007126525	CYOR	CYNOPS	ORIENTALIS	CHINESE DWARF	NEWT	LIV	200	NO	SG	SG	T	W	C	C	6/20/07	6/10/07	I	NY	INTERNATIONAL PET RESOURCES , LLC
2007126964	CYPY	CYNOPS	PYRRHOGASTER	JAPANESE FIREBELLY	NEWT	LIV	300	NO	HK	HK	T	W	C	C	6/13/07	6/10/07	I	NY	TRANSSHIP DISCOUNTS LIMITED
2007126964	CYPY	CYNOPS	PYRRHOGASTER	JAPANESE FIREBELLY	NEWT	LIV	90	NO	HK	HK	T	W	C	C	6/13/07	6/10/07	I	NY	TRANSSHIP DISCOUNTS LIMITED
2007127420	ONJA	ONYCHODACTYLUS	JAPONICUS	JAPANESE CLAWED	SALAMANDER	LIV	12	NO	JP	JP	T	W	C	C	11/7/07	11/7/07	I	LA	NAME REMOVED
2007127420	CYPY	CYNOPS	PYRRHOGASTER	JAPANESE FIREBELLY	NEWT	LIV	200	NO	JP	JP	T	W	C	C	11/7/07	11/7/07	I	LA	NAME REMOVED
2007128408	CYOR	CYNOPS	ORIENTALIS	CHINESE DWARF	NEWT	LIV	40	NO	SG	SG	T	W	C	C	3/14/07	2/25/07	I	NY	INTERNATIONAL PET RESOURCES , LLC
2007128413	CYOR	CYNOPS	ORIENTALIS	CHINESE DWARF	NEWT	LIV	200	NO	SG	SG	T	C	C	C	3/14/07	2/17/07	I	NY	INTERNATIONAL PET RESOURCES , LLC
2007128540	TRIH	TRITURUS	HONGKONGENSIS	ALPINE	NEWT	LIV	100	NO	HK	HK	T	W	C	C	3/14/07	2/25/07	I	NY	INTERNATIONAL PET RESOURCES , LLC
2007128873	PACB	PACHYTRITON	BREVIPES	TSITOU	NEWT	LIV	100	NO	HK	HK	T	C	C	C	11/10/07	11/10/07	I	LA	DOLPHIN INTERNATIONAL
2007128989	CYOR	CYNOPS	ORIENTALIS	CHINESE DWARF	NEWT	LIV	1,150	NO	HK	HK	T	C	C	C	11/10/07	11/11/07	I	LA	DOLPHIN INTERNATIONAL
2007128989	PACB	PACHYTRITON	BREVIPES	TSITOU	NEWT	LIV	100	NO	HK	HK	T	C	C	C	11/10/07	11/11/07	I	LA	DOLPHIN INTERNATIONAL
2007129000	TRIH	TRITURUS	HONGKONGENSIS	ALPINE	NEWT	LIV	100	NO	HK	HK	T	W	C	C	11/10/07	11/11/07	I	LA	AQUACO, INC.
2007129071	PLAB	PACHYTRITON	LABIATUS	UNTERSTEIN'S	NEWT	LIV	99	NO	CN	CN	T	C	C	C	11/12/07	11/12/07	I	AT	SUN PET LTD.
2007129359	CYOR	CYNOPS	ORIENTALIS	CHINESE DWARF	NEWT	LIV	200	NO	SG	SG	T	W	C	C	2/27/07	2/11/07	I	NY	INTERNATIONAL PET RESOURCES , LLC
2007130970	CYPY	CYNOPS	PYRRHOGASTER	JAPANESE FIREBELLY	NEWT	LIV	50	NO	HK	HK	T	W	C	C	11/15/07	11/11/07	I	NY	Transship Discounts Ltd.
2007131853	CYOR	CYNOPS	ORIENTALIS	CHINESE DWARF	NEWT	LIV	500	NO	CN	CN	T	W	C	C	11/16/07	11/16/07	I	LA	Z IMPORTS INC.
2007132224	CYOR	CYNOPS	ORIENTALIS	CHINESE DWARF	NEWT	LIV	750	NO	HK	HK	T	C	C	C	11/17/07	11/17/07	I	LA	DOLPHIN INTERNATIONAL
2007132334	TRIH	TRITURUS	HONGKONGENSIS	ALPINE	NEWT	LIV	100	NO	HK	HK	T	W	C	C	11/18/07	11/18/07	I	LA	AQUACO, INC.
2007132337	CYOR	CYNOPS	ORIENTALIS	CHINESE DWARF	NEWT	LIV	300	NO	CN	CN	T	W	C	C	11/18/07	11/18/07	I	LA	AQUA-NAUTIC SPECIALIST
2007132347	CYOR	CYNOPS	ORIENTALIS	CHINESE DWARF	NEWT	LIV	750	NO	HK	HK	T	C	C	C	11/18/07	11/18/07	I	LA	DOLPHIN INTERNATIONAL
2007132347	PACB	PACHYTRITON	BREVIPES	TSITOU	NEWT	LIV	200	NO	HK	HK	T	C	C	C	11/18/07	11/18/07	I	LA	DOLPHIN INTERNATIONAL
2007132431	CNP?	CYNOPS	SPECIES	FIREBELLY	NEWT	LIV	300	NO	CN	CN	T	C	C	C	11/19/07	11/19/07	I	AT	SUN PET LTD.
2007133510	TYVE	TYLOTOTRITON	VERRUCOSUS	CROCODILE	NEWT	LIV	300	NO	CN	CN	T	W	C	C	11/20/07	11/20/07	I	LA	GOLDEN POND
2007134196	NRR?	NEURERGUS	SPECIES		NEWT	LIV	21	NO	DE	DE	T	C	C	C	11/21/07	11/21/07	I	DF	U.S. GLOBAL EXOTICS, INC.
2007134569	CYOR	CYNOPS	ORIENTALIS	CHINESE DWARF	NEWT	LIV	500	NO	CN	CN	T	W	C	C	11/23/07	11/23/07	I	LA	WORLD WIDE TRADING INC.
2007134569	PLAB	PACHYTRITON	LABIATUS	UNTERSTEIN'S	NEWT	LIV	1,000	NO	CN	CN	T	W	C	C	11/23/07	11/23/07	I	LA	WORLD WIDE TRADING INC.
2007134920	CYOR	CYNOPS	ORIENTALIS	CHINESE DWARF	NEWT	LIV	500	NO	HK	HK	T	C	C	C	11/24/07	11/24/07	I	LA	DOLPHIN INTERNATIONAL
2007134920	PACB	PACHYTRITON	BREVIPES	TSITOU	NEWT	LIV	100	NO	HK	HK	T	C	C	C	11/24/07	11/24/07	I	LA	DOLPHIN INTERNATIONAL
2007135022	TRIH	TRITURUS	HONGKONGENSIS	ALPINE	NEWT	LIV	50	NO	HK	HK	T	W	C	C	11/24/07	11/25/07	I	LA	AQUACO, INC.
2007135024	CYOR	CYNOPS	ORIENTALIS	CHINESE DWARF	NEWT	LIV	500	NO	HK	HK	T	C	C	C	11/24/07	11/25/07	I	LA	DOLPHIN INTERNATIONAL
2007135024	PACB	PACHYTRITON	BREVIPES	TSITOU	NEWT	LIV	100	NO	HK	HK	T	C	C	C	11/24/07	11/25/07	I	LA	DOLPHIN INTERNATIONAL
2007135052	CYOR	CYNOPS	ORIENTALIS	CHINESE DWARF	NEWT	LIV	1,695	NO	CN	CN	T	W	C	C	11/25/07	11/25/07	I	LA	AQUA-NAUTIC SPECIALIST
2007135616	CYPY	CYNOPS	PYRRHOGASTER	JAPANESE FIREBELLY	NEWT	LIV	170	NO	HK	HK	T	W	C	C	11/26/07	11/18/07	I	NY	Transship Discounts Ltd.
2007138680	CYOR	CYNOPS	ORIENTALIS	CHINESE DWARF	NEWT	LIV	346	NO	CN	CN	T	W	C	C	12/1/07	12/2/07	I	LA	AQUA-NAUTIC SPECIALIST
2007138683	TRIH	TRITURUS	HONGKONGENSIS	ALPINE	NEWT	LIV	225	NO	HK	HK	T	W	C	C	12/1/07	12/2/07	I	LA	AQUACO, INC.
2007138688	CYOR	CYNOPS	ORIENTALIS	CHINESE DWARF	NEWT	LIV	250	NO	HK	HK	T	C	C	C	12/1/07	12/2/07	I	LA	DOLPHIN INTERNATIONAL
2007138688	PACB	PACHYTRITON	BREVIPES	TSITOU	NEWT	LIV	300	NO	HK	HK	T	C	C	C	12/1/07	12/2/07	I	LA	DOLPHIN INTERNATIONAL
2007138818	CNP?	CYNOPS	SPECIES	FIREBELLY	NEWT	LIV	300	NO	CN	CN	T	C	C	C	12/3/07	12/3/07	I	AT	SUN PET LTD.
2007140919	CYPY	CYNOPS	PYRRHOGASTER	JAPANESE FIREBELLY	NEWT	LIV	210	NO	HK	HK	T	W	C	C	12/6/07	12/2/07	I	NY	Transship Discounts Ltd.
2007140919	PACB	PACHYTRITON	BREVIPES	TSITOU	NEWT	LIV	60	NO	HK	HK	T	W	C	C	12/6/07	12/2/07	I	NY	Transship Discounts Ltd.
2007140921	CYPY	CYNOPS	PYRRHOGASTER	JAPANESE FIREBELLY	NEWT	LIV	200	NO	HK	HK	T	W	C	C	12/6/07	11/25/07	I	NY	Transship Discounts Ltd.
2007140981	CNP?	CYNOPS	SPECIES	FIREBELLY	NEWT	LIV	9	NO	CA	CA	T	C	C	C	9/17/07	9/17/07	I	BN	NAME REMOVED
2007140981	PCAU	PARAMESOTRITON	CAUDOPUNCTATUS	GUIZHOU WARTY	NEWT	LIV	8	NO	CA	CA	T	C	C	C	9/17/07	9/17/07	I	BN	NAME REMOVED
2007140981	TRIT	TRITURUS	SPECIES		NEWT	LIV	36	NO	CA	CA	T	C	C	C	9/17/07	9/17/07	I	BN	NAME REMOVED
2007142051	CYOR	CYNOPS	ORIENTALIS	CHINESE DWARF	NEWT	LIV	750	NO	HK	HK	T	C	C	C	12/8/07	12/8/07	I	LA	DOLPHIN INTERNATIONAL
2007142051	PACB	PACHYTRITON	BREVIPES	TSITOU	NEWT	LIV	100	NO	HK	HK	T	C	C	C	12/8/07	12/8/07	I	LA	DOLPHIN INTERNATIONAL
2007142153	CYOR	CYNOPS	ORIENTALIS	CHINESE DWARF	NEWT	LIV	1,300	NO	HK	HK	T	C	C	C	12/8/07	12/9/07	I	LA	DOLPHIN INTERNATIONAL
2007142172	TRIH	TRITURUS	HONGKONGENSIS	ALPINE	NEWT	LIV	25	NO	HK	HK	T	W	C	C	12/8/07	12/9/07	I	LA	AQUACO, INC.
2007143364	TRIH	TRITURUS	HONGKONGENSIS	ALPINE	NEWT	LIV	1,600	NO	HK	HK	T	C	C	C	12/11/07	12/11/07	I	TP	5-D TROPICAL INC.
2007143519	CYPY	CYNOPS	PYRRHOGASTER	JAPANESE FIREBELLY	NEWT	LIV	100	NO	HK	HK	T	W	C	C	12/11/07	12/9/07	I	NY	Transship Discounts Ltd.
2007145812	CYOR	CYNOPS	ORIENTALIS	CHINESE DWARF	NEWT	LIV	100	NO	HK	HK	T	C	C	C	12/16/07	12/16/07	I	LA	DOLPHIN INTERNATIONAL
2007145812	PACB	PACHYTRITON	BREVIPES	TSITOU	NEWT	LIV	200	NO	HK	HK	T	C	C	C	12/16/07	12/16/07	I	LA	DOLPHIN INTERNATIONAL
2007145842	PLAB	PACHYTRITON	LABIATUS	UNTERSTEIN'S	NEWT	LIV	100	NO	CN	CN	T	C	C	C	12/17/07	12/17/07	I	AT	SUN PET LTD.
2007149263	CYOR	CYNOPS	ORIENTALIS	CHINESE DWARF	NEWT	LIV	500	NO	HK	HK	T	C	C	C	12/22/07	12/22/07	I	LA	DOLPHIN INTERNATIONAL
2007149924	CYPY	CYNOPS	PYRRHOGASTER	JAPANESE FIREBELLY	NEWT	LIV	120	NO	HK	HK	T	W	C	C	12/26/07	12/23/07	I	NY	Transship Discounts Ltd.
2007149924	PACB	PACHYTRITON	BREVIPES	TSITOU	NEWT	LIV	60	NO	HK	HK	T	W	C	C	12/26/07	12/23/07	I	NY	Transship Discounts Ltd.
2007151190	CYOR	CYNOPS	ORIENTALIS	CHINESE DWARF	NEWT	LIV	250	NO	HK	HK	T	C	C	C	12/29/07	12/29/07	I	LA	DOLPHIN INTERNATIONAL
2007151190	PACB	PACHYTRITON	BREVIPES	TSITOU	NEWT	LIV	100	NO	HK	HK	T	C	C	C	12/29/07	12/29/07	I	LA	DOLPHIN INTERNATIONAL

2007151202	CYOR	CYNOPS	ORIENTALIS	CHINESE DWARF	NEWT	LIV	600	NO	CN	CN	T	W	C	C	12/29/07	12/29/07	I	LA	AQUA-NAUTIC SPECIALIST
2007151276	CYOR	CYNOPS	ORIENTALIS	CHINESE DWARF	NEWT	LIV	1,000	NO	HK	HK	T	C	C	C	12/29/07	12/30/07	I	LA	DOLPHIN INTERNATIONAL
2007867707	TRIT	TRITURUS	SPECIES		NEWT	LIV	200	NO	HK	HK	T	C	C	C	1/6/07	1/6/07	I	LA	GLOBAL AQUATIC CONSULTING
2007867786	TRIH	TRITURUS	HONGKONGENSIS	ALPINE	NEWT	LIV	100	NO	HK	HK	T	W	C	C	1/7/07	1/7/07	I	LA	AQUACO, INC.
2007867817	PACB	PACHYTRITON	BREVIPES	TSITOU	NEWT	LIV	900	NO	HK	HK	T	C	C	C	1/7/07	1/7/07	I	LA	DOLPHIN INTERNATIONAL
2007868093	CNP?	CYNOPS	SPECIES	FIREBELLY	NEWT	LIV	400	NO	HK	HK	T	C	C	C	1/8/07	1/8/07	I	AT	SUN PET LTD.
2007869226	NEW?	PACHYTRITON	SPECIES	CHINESE	NEWT	LIV	250	NO	CN	CN	T	C	C	C	1/10/07	1/9/07	I	SF	Golden Phoenix Fisheries, USA
2007870496	TRIH	TRITURUS	HONGKONGENSIS	ALPINE	NEWT	LIV	200	NO	HK	PE	T	W	C	C	1/13/07	1/14/07	I	LA	AQUACO, INC.
2007870507	PACB	PACHYTRITON	BREVIPES	TSITOU	NEWT	LIV	500	NO	HK	HK	T	C	C	C	1/13/07	1/14/07	I	LA	DOLPHIN INTERNATIONAL
2007870507	CYOR	CYNOPS	ORIENTALIS	CHINESE DWARF	NEWT	LIV	90	NO	HK	HK	T	C	C	C	1/13/07	1/14/07	I	LA	DOLPHIN INTERNATIONAL
2007873756	TRIH	TRITURUS	HONGKONGENSIS	ALPINE	NEWT	LIV	150	NO	HK	HK	T	W	C	C	1/21/07	1/21/07	I	LA	AQUACO, INC.
2007873768	CYOR	CYNOPS	ORIENTALIS	CHINESE DWARF	NEWT	LIV	90	NO	HK	HK	T	C	C	C	1/21/07	1/21/07	I	LA	DOLPHIN INTERNATIONAL
2007879865	TRIH	TRITURUS	HONGKONGENSIS	ALPINE	NEWT	LIV	200	NO	HK	HK	T	W	C	C	2/4/07	2/4/07	I	LA	AQUACO, INC.
2007879868	CYOR	CYNOPS	ORIENTALIS	CHINESE DWARF	NEWT	LIV	90	NO	HK	HK	T	C	C	C	2/4/07	2/4/07	I	LA	DOLPHIN INTERNATIONAL
2007883528	CYOR	CYNOPS	ORIENTALIS	CHINESE DWARF	NEWT	LIV	500	NO	HK	HK	T	C	C	C	2/10/07	2/10/07	I	LA	DOLPHIN INTERNATIONAL
2007883613	TRIH	TRITURUS	HONGKONGENSIS	ALPINE	NEWT	LIV	550	NO	HK	HK	T	W	C	C	2/11/07	2/11/07	I	LA	AQUACO, INC.
2007883615	CYOR	CYNOPS	ORIENTALIS	CHINESE DWARF	NEWT	LIV	950	NO	HK	HK	T	C	C	C	2/11/07	2/11/07	I	LA	DOLPHIN INTERNATIONAL
2007883807	TRIT	TRITURUS	SPECIES		NEWT	LIV	400	NO	HK	HK	T	W	C	C	11/20/06	11/18/06	I	LA	GLOBAL AQUATIC CONSULTING
2007887854	TRIH	TRITURUS	HONGKONGENSIS	ALPINE	NEWT	LIV	100	NO	HK	HK	T	W	C	C	2/18/07	2/18/07	I	LA	AQUACO, INC.
2007888062	BODO	BOLITOGLOSSA	DOFLEINI	ALTA VERAPAZ	SALAMANDER	LIV	2	NO	GT	GT	T	W	C	C	8/7/06	8/6/06	I	MI	DHAR TROPICAL LLC
2007888185	SASA	SALAMANDRA	SALAMANDRA	EUROPEAN FIRE	SALAMANDER	LIV	600	NO	SI	SI	T	D	C	C	12/8/06	12/8/06	I	MI	GLOBAL TROPICAL IMP/EXP
2007889718	SASA	SALAMANDRA	SALAMANDRA	EUROPEAN FIRE	SALAMANDER	LIV	1,300	NO	SI	SI	T	C	C	C	9/29/06	9/29/06	I	MI	GLOBAL TROPICAL IMP/EXP
2007889937	BODO	BOLITOGLOSSA	DOFLEINI	ALTA VERAPAZ	SALAMANDER	LIV	15	NO	HN	HN	T	W	C	C	6/23/04	6/23/04	I	MI	STRICTLY REPTILES, INC.
2007891878	TRIH	TRITURUS	HONGKONGENSIS	ALPINE	NEWT	LIV	150	NO	HK	HK	T	W	C	C	2/25/07	2/25/07	I	LA	AQUACO, INC.
2007891891	CYOR	CYNOPS	ORIENTALIS	CHINESE DWARF	NEWT	LIV	820	NO	CN	CN	T	W	C	C	2/25/07	2/25/07	I	LA	AQUA-NAUTIC SPECIALIST
2007892249	CNP?	CYNOPS	SPECIES	FIREBELLY	NEWT	LIV	300	NO	CN	CN	T	C	C	C	2/26/07	2/26/07	I	AT	SUN PET LTD.
2007892428	SASA	SALAMANDRA	SALAMANDRA	EUROPEAN FIRE	SALAMANDER	LIV	1,500	NO	SI	SI	T	C	C	C	2/26/07	2/26/07	I	DF	U.S. GLOBAL EXOTICS, INC.
2007892428	SAL?	SALAMANDRA	SPECIES	FIRE	SALAMANDER	LIV	50	NO	SI	SI	T	C	C	C	2/26/07	2/26/07	I	DF	U.S. GLOBAL EXOTICS, INC.
2007895275	CYOR	CYNOPS	ORIENTALIS	CHINESE DWARF	NEWT	LIV	750	NO	HK	HK	T	C	C	C	3/3/07	3/3/07	I	LA	DOLPHIN INTERNATIONAL
2007895316	TRIH	TRITURUS	HONGKONGENSIS	ALPINE	NEWT	LIV	50	NO	HK	HK	T	W	C	C	3/3/07	3/4/07	I	LA	AQUACO, INC.
2007895323	CYOR	CYNOPS	ORIENTALIS	CHINESE DWARF	NEWT	LIV	2,450	NO	HK	HK	T	C	C	C	3/3/07	3/4/07	I	LA	DOLPHIN INTERNATIONAL
2007895335	CYOR	CYNOPS	ORIENTALIS	CHINESE DWARF	NEWT	LIV	200	NO	SG	SG	T	W	C	C	3/3/07	3/4/07	I	LA	TROPICAL FISH CONSOLIDATORS LTD.
2007895338	CYOR	CYNOPS	ORIENTALIS	CHINESE DWARF	NEWT	LIV	1,200	NO	CN	CN	T	W	C	C	3/3/07	3/4/07	I	LA	AQUA-NAUTIC SPECIALIST
2007895369	CNP?	CYNOPS	SPECIES	FIREBELLY	NEWT	LIV	600	NO	CN	CN	T	C	C	C	3/5/07	3/5/07	I	AT	SUN PET LTD.
2007895369	PLAB	PACHYTRITON	LABIATUS	UNTERSTEIN'S	NEWT	LIV	50	NO	CN	CN	T	C	C	C	3/5/07	3/5/07	I	AT	SUN PET LTD.
2007898036	SAL?	SALAMANDRA	SPECIES	FIRE	SALAMANDER	LIV	115	NO	GB	GB	T	W	C	C	3/8/07	12/14/06	I	MI	TWO AMIGOS IMPORT & EXPORT, INC.
2007898036	TRIT	TRITURUS	SPECIES		NEWT	LIV	24	NO	GB	GB	T	W	C	C	3/8/07	12/14/06	I	MI	TWO AMIGOS IMPORT & EXPORT, INC.
2007898959	CYPY	CYNOPS	PYRRHOGASTER	JAPANESE FIREBELLY	NEWT	LIV	200	NO	CN	CN	T	C	C	C	10/12/06	10/8/06	I	NY	FISH MART INCORPORATED
2007899460	CYOR	CYNOPS	ORIENTALIS	CHINESE DWARF	NEWT	LIV	4,000	NO	CN	CN	T	W	C	C	3/9/07	3/9/07	I	LA	Z IMPORTS INC.
2007899778	TRIT	TRITURUS	SPECIES		NEWT	LIV	400	NO	HK	HK	T	W	C	C	3/10/07	3/10/07	I	LA	GLOBAL AQUATIC CONSULTING
2007899826	TRIH	TRITURUS	HONGKONGENSIS	ALPINE	NEWT	LIV	50	NO	HK	HK	T	W	C	C	3/10/07	3/11/07	I	LA	AQUACO, INC.
2007899830	CYOR	CYNOPS	ORIENTALIS	CHINESE DWARF	NEWT	LIV	1,650	NO	CN	CN	T	W	C	C	3/10/07	3/11/07	I	LA	AQUA-NAUTIC SPECIALIST
2007899863	CYOR	CYNOPS	ORIENTALIS	CHINESE DWARF	NEWT	LIV	1,500	NO	HK	HK	T	C	C	C	3/11/07	3/11/07	I	LA	DOLPHIN INTERNATIONAL
2007899882	TRIT	TRITURUS	SPECIES		NEWT	LIV	800	NO	HK	HK	T	C	C	C	3/11/07	3/11/07	I	LA	AQUATIC CONNECTION
2007900947	CYPY	CYNOPS	PYRRHOGASTER	JAPANESE FIREBELLY	NEWT	LIV	50	NO	HK	CN	T	W	C	C	1/3/07	12/29/06	I	NY	ERDA INCORPORATED
2007901210	CYPY	CYNOPS	PYRRHOGASTER	JAPANESE FIREBELLY	NEWT	LIV	150	NO	HK	HK	T	W	C	C	12/29/06	12/24/06	I	NY	TRANSSHIP DISCOUNTS LIMITED
2007902445	CYOR	CYNOPS	ORIENTALIS	CHINESE DWARF	NEWT	LIV	150	NO	HK	HK	T	C	C	C	3/14/07	3/14/07	I	CH	AQUATICS, INC.
2007903200	CYPY	CYNOPS	PYRRHOGASTER	JAPANESE FIREBELLY	NEWT	LIV	50	NO	HK	HK	T	W	C	C	12/14/06	12/10/06	I	NY	TRANSSHIP DISCOUNTS LIMITED
2007904179	CYOR	CYNOPS	ORIENTALIS	CHINESE DWARF	NEWT	LIV	4,000	NO	CN	CN	T	W	C	C	3/16/07	3/16/07	I	LA	Z IMPORTS INC.
2007904521	TRIT	TRITURUS	SPECIES		NEWT	LIV	200	NO	HK	HK	T	W	C	C	3/17/07	3/17/07	I	LA	GLOBAL AQUATIC CONSULTING
2007904539	CYOR	CYNOPS	ORIENTALIS	CHINESE DWARF	NEWT	LIV	1,350	NO	CN	CN	T	W	C	C	3/17/07	3/18/07	I	LA	AQUA-NAUTIC SPECIALIST
2007904544	TRIH	TRITURUS	HONGKONGENSIS	ALPINE	NEWT	LIV	300	NO	HK	HK	T	W	C	C	3/17/07	3/18/07	I	LA	AQUACO, INC.
2007904566	CYOR	CYNOPS	ORIENTALIS	CHINESE DWARF	NEWT	LIV	100	NO	SG	SG	T	W	C	C	3/17/07	3/18/07	I	LA	TROPICAL FISH CONSOLIDATORS LTD.
2007904602	CYOR	CYNOPS	ORIENTALIS	CHINESE DWARF	NEWT	LIV	660	NO	HK	HK	T	C	C	C	3/18/07	3/18/07	I	LA	DOLPHIN INTERNATIONAL
2007904618	TRIT	TRITURUS	SPECIES		NEWT	LIV	400	NO	HK	HK	T	C	C	C	3/18/07	3/18/07	I	LA	AQUATIC CONNECTION
2007904637	CYPY	CYNOPS	PYRRHOGASTER	JAPANESE FIREBELLY	NEWT	LIV	300	NO	CN	CN	T	C	C	C	3/19/07	3/19/07	I	AT	SUN PET LTD.
2007905200	CYPY	CYNOPS	PYRRHOGASTER	JAPANESE FIREBELLY	NEWT	LIV	300	NO	HK	HK	T	W	C	C	11/30/06	11/26/06	I	NY	TRANSSHIP DISCOUNTS LIMITED
2007905200	PACB	PACHYTRITON	BREVIPES	TSITOU	NEWT	LIV	30	NO	HK	HK	T	W	C	C	11/30/06	11/26/06	I	NY	TRANSSHIP DISCOUNTS LIMITED
2007905500	CYOR	CYNOPS	ORIENTALIS	CHINESE DWARF	NEWT	LIV	100	NO	SG	SG	T	W	C	C	11/30/06	11/19/06	I	NY	INTERNATIONAL PET RESOURCES , LLC
2007907825	CYPY	CYNOPS	PYRRHOGASTER	JAPANESE FIREBELLY	NEWT	LIV	100	NO	HK	HK	T	W	C	C	1/4/07	12/31/06	I	NY	TRANSSHIP DISCOUNTS LIMITED

2007908211	CYPY	CYNOPS	PYRRHOGASTER	JAPANESE FIREBELLY	NEWT	LIV	160	NO	HK	HK	T	W	C	C	11/6/06	11/5/06	I	NY	TRANSSHIP DISCOUNTS LIMITED
2007908434	SASA	SALAMANDRA	SALAMANDRA	EUROPEAN FIRE	SALAMANDER	LIV	123	NO	GB	GB	T	C	C	C	3/22/07	3/22/07	I	MI	TWO AMIGOS IMPORT & EXPORT, INC.
2007908635	PCHI	PARAMESOTRITON	CHINENSIS	CHINESE WARTY	NEWT	LIV	200	NO	CN	HK	T	W	C	C	3/23/07	3/23/07	I	DN	Bushmaster Reptiles Inc
2007909192	TRIT	TRITURUS	SPECIES		NEWT	LIV	200	NO	HK	HK	T	W	C	C	3/24/07	3/24/07	I	LA	GLOBAL AQUATIC CONSULTING
2007909269	TRIH	TRITURUS	HONGKONGENSIS	ALPINE	NEWT	LIV	50	NO	HK	HK	T	W	C	C	3/24/07	3/25/07	I	LA	AQUACO, INC.
2007909289	CYOR	CYNOPS	ORIENTALIS	CHINESE DWARF	NEWT	LIV	1,200	NO	CN	CN	T	W	C	C	3/25/07	3/25/07	I	LA	AQUA-NAUTIC SPECIALIST
2007909295	CNP?	CYNOPS	SPECIES	FIREBELLY	NEWT	LIV	300	NO	CN	CN	T	C	C	C	3/26/07	3/26/07	I	AT	SUN PET LTD.
2007909365	TRIH	TRITURUS	HONGKONGENSIS	ALPINE	NEWT	LIV	50	NO	HK	HK	T	W	C	C	1/30/07	1/19/07	I	NY	INTERNATIONAL PET RESOURCES , LLC
2007909479	CYPY	CYNOPS	PYRRHOGASTER	JAPANESE FIREBELLY	NEWT	LIV	50	NO	HK	HK	T	W	C	C	1/9/07	1/7/07	I	NY	TRANSSHIP DISCOUNTS LIMITED
2007910180	NEW?	PACHYTRITON	SPECIES	CHINESE	NEWT	LIV	180	NO	CN	CN	T	C	C	C	3/27/07	3/27/07	I	SF	Golden Phoenix Fisheries, USA
2007910920	CNP?	CYNOPS	SPECIES	FIREBELLY	NEWT	LIV	400	NO	HK	HK	T	C	C	C	1/8/07	1/8/07	I	AT	SUN PET LTD.
2007911079	CYPY	CYNOPS	PYRRHOGASTER	JAPANESE FIREBELLY	NEWT	LIV	175	NO	HK	HK	T	W	C	C	12/5/06	12/2/06	I	NY	ERDA INCORPORATED
2007911171	TRIT	TRITURUS	SPECIES		NEWT	LIV	150	NO	HK	HK	T	W	C	C	12/5/06	11/26/06	I	NY	INTERNATIONAL PET RESOURCES , LLC
2007911274	CYPY	CYNOPS	PYRRHOGASTER	JAPANESE FIREBELLY	NEWT	LIV	50	NO	HK	HK	T	W	C	C	12/5/06	12/3/06	I	NY	TRANSSHIP DISCOUNTS LIMITED
2007911304	CYPY	CYNOPS	PYRRHOGASTER	JAPANESE FIREBELLY	NEWT	LIV	200	NO	HK	HK	T	W	C	C	12/18/06	12/17/06	I	NY	TRANSSHIP DISCOUNTS LIMITED
2007911342	CYPY	CYNOPS	PYRRHOGASTER	JAPANESE FIREBELLY	NEWT	LIV	75	NO	HK	HK	T	C	C	C	12/18/06	12/16/06	I	NY	ERDA INCORPORATED
2007911908	CNP?	CYNOPS	SPECIES	FIREBELLY	NEWT	LIV	400	NO	HK	HK	T	W	C	C	12/11/06	12/10/06	I	NY	ERDA INCORPORATED
2007911911	CYPY	CYNOPS	PYRRHOGASTER	JAPANESE FIREBELLY	NEWT	LIV	585	NO	HK	HK	T	W	C	C	5/15/06	5/13/06	I	NY	ERDA INCORPORATED
2007912112	CYPY	CYNOPS	PYRRHOGASTER	JAPANESE FIREBELLY	NEWT	LIV	350	NO	HK	HK	T	W	C	C	5/9/06	5/6/06	I	NY	ERDA INCORPORATED
2007912131	CYPY	CYNOPS	PYRRHOGASTER	JAPANESE FIREBELLY	NEWT	LIV	50	NO	HK	HK	T	W	C	C	11/21/06	11/19/06	I	NY	TRANSSHIP DISCOUNTS LIMITED
2007912323	TRIH	TRITURUS	HONGKONGENSIS	ALPINE	NEWT	LIV	150	NO	HK	HK	T	W	C	C	11/6/06	10/29/06	I	NY	INTERNATIONAL PET RESOURCES , LLC
2007912970	CYPY	CYNOPS	PYRRHOGASTER	JAPANESE FIREBELLY	NEWT	LIV	150	NO	HK	HK	T	W	C	C	11/20/06	11/18/06	I	NY	ERDA INCORPORATED
2007913245	SIRL	SIREN	LACERTINA	GREATER	SIREN	LIV	12	NO	US	JP	T	C	C	C	3/29/07	3/29/07	I	MI	NAME REMOVED
2007913523	TRIT	TRITURUS	SPECIES		NEWT	LIV	200	NO	HK	HK	T	W	C	C	3/31/07	3/31/07	I	LA	GLOBAL AQUATIC CONSULTING
2007913592	TRIH	TRITURUS	HONGKONGENSIS	ALPINE	NEWT	LIV	90	NO	HK	HK	T	W	C	C	3/31/07	4/1/07	I	LA	AQUACO, INC.
2007913598	CNP?	CYNOPS	SPECIES	FIREBELLY	NEWT	LIV	37	NO	SG	SG	T	W	C	C	3/31/07	4/1/07	I	LA	TROPICAL FISH CONSOLIDATORS LTD.
2007913616	CYOR	CYNOPS	ORIENTALIS	CHINESE DWARF	NEWT	LIV	2,900	NO	HK	HK	T	C	C	C	4/1/07	4/1/07	I	LA	DOLPHIN INTERNATIONAL
2007913616	PACB	PACHYTRITON	BREVIPES	TSITOU	NEWT	LIV	300	NO	HK	HK	T	C	C	C	4/1/07	4/1/07	I	LA	DOLPHIN INTERNATIONAL
2007913640	TRIT	TRITURUS	SPECIES		NEWT	LIV	200	NO	HK	HK	T	C	C	C	4/1/07	4/1/07	I	LA	AQUATIC CONNECTION
2007917021	NOTO	NOTOPHTHALMUS	SPECIES		NEWT	LIV	1	NO	TH	TH	T	C	C	C	4/6/07	4/7/07	I	DU	PSaltwater LLC
2007917260	CYOR	CYNOPS	ORIENTALIS	CHINESE DWARF	NEWT	LIV	3,000	NO	CN	CN	T	W	C	C	4/6/07	4/6/07	I	LA	Z IMPORTS INC.
2007917384	CYOR	CYNOPS	ORIENTALIS	CHINESE DWARF	NEWT	LIV	250	NO	HK	HK	T	C	C	C	4/6/07	4/7/07	I	LA	DOLPHIN INTERNATIONAL
2007917587	CYOR	CYNOPS	ORIENTALIS	CHINESE DWARF	NEWT	LIV	2,050	NO	HK	HK	T	C	C	C	4/8/07	4/8/07	I	LA	DOLPHIN INTERNATIONAL
2007917587	PACB	PACHYTRITON	BREVIPES	TSITOU	NEWT	LIV	100	NO	HK	HK	T	C	C	C	4/8/07	4/8/07	I	LA	DOLPHIN INTERNATIONAL
2007917609	CYOR	CYNOPS	ORIENTALIS	CHINESE DWARF	NEWT	LIV	900	NO	CN	CN	T	W	C	C	4/8/07	4/8/07	I	LA	AQUA-NAUTIC SPECIALIST
2007917626	CNP?	CYNOPS	SPECIES	FIREBELLY	NEWT	LIV	300	NO	CN	CN	T	C	C	C	4/9/07	4/9/07	I	AT	SUN PET LTD.
2007921724	CYOR	CYNOPS	ORIENTALIS	CHINESE DWARF	NEWT	LIV	500	NO	HK	HK	T	C	C	C	4/13/07	4/14/07	I	LA	DOLPHIN INTERNATIONAL
2007921724	PACB	PACHYTRITON	BREVIPES	TSITOU	NEWT	LIV	200	NO	HK	HK	T	C	C	C	4/13/07	4/14/07	I	LA	DOLPHIN INTERNATIONAL
2007921840	TRIT	TRITURUS	SPECIES		NEWT	LIV	400	NO	HK	HK	T	C	C	C	4/14/07	4/14/07	I	LA	GLOBAL AQUATIC CONSULTING
2007921878	CYOR	CYNOPS	ORIENTALIS	CHINESE DWARF	NEWT	LIV	2,060	NO	CN	CN	T	W	C	C	4/14/07	4/15/07	I	LA	AQUA-NAUTIC SPECIALIST
2007921886	TRIH	TRITURUS	HONGKONGENSIS	ALPINE	NEWT	LIV	200	NO	HK	HK	T	W	C	C	4/14/07	4/15/07	I	LA	AQUACO, INC.
2007921888	CYOR	CYNOPS	ORIENTALIS	CHINESE DWARF	NEWT	LIV	5,499	NO	HK	HK	T	C	C	C	4/14/07	4/15/07	I	LA	DOLPHIN INTERNATIONAL
2007921888	PACB	PACHYTRITON	BREVIPES	TSITOU	NEWT	LIV	200	NO	HK	HK	T	C	C	C	4/14/07	4/15/07	I	LA	DOLPHIN INTERNATIONAL
2007921958	CNP?	CYNOPS	SPECIES	FIREBELLY	NEWT	LIV	300	NO	CN	CN	T	C	C	C	4/16/07	4/16/07	I	AT	SUN PET LTD.
2007923088	PDT?	PSEUDOTRITON	SPECIES	RED	SALAMANDER	LIV	2	NO	AT	AT	T	C	C	C	9/21/06	9/20/06	I	MI	NAME REMOVED
2007923088	PDT?	PSEUDOTRITON	SPECIES	RED	SALAMANDER	LIV	4	NO	AT	AT	T	C	C	C	9/21/06	9/20/06	I	MI	NAME REMOVED
2007923644	CYOR	CYNOPS	ORIENTALIS	CHINESE DWARF	NEWT	LIV	150	NO	HK	HK	T	C	C	C	4/18/07	4/18/07	I	CH	AQUATICS, INC.
2007924405	TRIH	TRITURUS	HONGKONGENSIS	ALPINE	NEWT	LIV	1,600	NO	HK	HK	T	C	C	C	4/18/07	4/17/07	I	TP	5-D TROPICAL, INC.
2007926187	TRIT	TRITURUS	SPECIES		NEWT	LIV	200	NO	HK	HK	T	C	C	C	4/21/07	4/21/07	I	LA	GLOBAL AQUATIC CONSULTING
2007926264	CNP?	CYNOPS	SPECIES	FIREBELLY	NEWT	LIV	80	NO	SG	SG	T	W	C	C	4/21/07	4/22/07	I	LA	TROPICAL FISH CONSOLIDATORS LTD.
2007926300	CYOR	CYNOPS	ORIENTALIS	CHINESE DWARF	NEWT	LIV	1,418	NO	HK	HK	T	C	C	C	4/22/07	4/22/07	I	LA	DOLPHIN INTERNATIONAL
2007926300	PACB	PACHYTRITON	BREVIPES	TSITOU	NEWT	LIV	100	NO	HK	HK	T	C	C	C	4/22/07	4/22/07	I	LA	DOLPHIN INTERNATIONAL
2007926316	TRIH	TRITURUS	HONGKONGENSIS	ALPINE	NEWT	LIV	250	NO	HK	HK	T	W	C	C	4/22/07	4/22/07	I	LA	AQUACO, INC.
2007926328	TRIH	TRITURUS	HONGKONGENSIS	ALPINE	NEWT	LIV	900	NO	CN	CN	T	W	C	C	4/22/07	4/22/07	I	LA	AQUA-NAUTIC SPECIALIST
2007926354	CNP?	CYNOPS	SPECIES	FIREBELLY	NEWT	LIV	300	NO	CN	CN	T	C	C	C	4/23/07	4/23/07	I	AT	SUN PET LTD.
2007929196	CYPY	CYNOPS	PYRRHOGASTER	JAPANESE FIREBELLY	NEWT	LIV	300	NO	HK	CN	T	W	C	C	2/27/07	2/24/07	I	NY	ERDA INCORPORATED
2007929239	CYPY	CYNOPS	PYRRHOGASTER	JAPANESE FIREBELLY	NEWT	LIV	150	NO	HK	HK	T	W	C	C	4/4/07	4/1/07	I	NY	TRANSSHIP DISCOUNTS LIMITED
2007929267	CYPY	CYNOPS	PYRRHOGASTER	JAPANESE FIREBELLY	NEWT	LIV	50	NO	HK	CN	T	W	C	C	3/7/07	3/3/07	I	NY	ERDA INCORPORATED
2007929354	CYPY	CYNOPS	PYRRHOGASTER	JAPANESE FIREBELLY	NEWT	LIV	50	NO	HK	HK	T	W	C	C	3/6/07	3/4/07	I	NY	TRANSSHIP DISCOUNTS LIMITED
2007929531	CYPY	CYNOPS	PYRRHOGASTER	JAPANESE FIREBELLY	NEWT	LIV	300	NO	HK	HK	T	W	C	C	3/14/07	3/11/07	I	NY	TRANSSHIP DISCOUNTS LIMITED

2007929557	CYPY	CYNOPS	PYRRHOGASTER	JAPANESE FIREBELLY	NEWT	LIV	150	NO	HK	CN	T	C	C	C	3/14/07	3/10/07	I	NY	ERDA INCORPORATED
2007929579	CYPY	CYNOPS	PYRRHOGASTER	JAPANESE FIREBELLY	NEWT	LIV	250	NO	HK	HK	T	W	C	C	3/18/07	3/18/07	I	NY	TRANSSHIP DISCOUNTS LIMITED
2007930008	TRIH	TRITURUS	HONGKONGENSIS	ALPINE	NEWT	LIV	200	NO	HK	HK	T	C	C	C	3/6/07	3/5/07	I	NY	TRI-COUNTY TROPICALS, INC.
2007930078	CYPY	CYNOPS	PYRRHOGASTER	JAPANESE FIREBELLY	NEWT	LIV	210	NO	HK	HK	T	W	C	C	4/4/07	3/31/07	I	NY	ERDA INCORPORATED
2007930089	CYPY	CYNOPS	PYRRHOGASTER	JAPANESE FIREBELLY	NEWT	LIV	45	NO	HK	HK	T	W	C	C	2/27/07	2/25/07	I	NY	TRANSSHIP DISCOUNTS LIMITED
2007930121	CYPY	CYNOPS	PYRRHOGASTER	JAPANESE FIREBELLY	NEWT	LIV	200	NO	HK	HK	T	W	C	C	1/24/07	1/20/07	I	NY	ERDA INCORPORATED
2007930272	CYPY	CYNOPS	PYRRHOGASTER	JAPANESE FIREBELLY	NEWT	LIV	50	NO	HK	HK	T	W	C	C	2/14/07	2/11/07	I	NY	TRANSSHIP DISCOUNTS LIMITED
2007930295	CYPY	CYNOPS	PYRRHOGASTER	JAPANESE FIREBELLY	NEWT	LIV	171	NO	HK	HK	T	W	C	C	3/28/07	3/24/07	I	NY	ERDA INCORPORATED
2007930509	TRIT	TRITURUS	SPECIES		NEWT	LIV	200	NO	HK	HK	T	C	C	C	4/28/07	4/28/07	I	LA	GLOBAL AQUATIC CONSULTING
2007930529	CYOR	CYNOPS	ORIENTALIS	CHINESE DWARF	NEWT	LIV	250	NO	HK	HK	T	C	C	C	4/28/07	4/28/07	I	LA	DOLPHIN INTERNATIONAL
2007930603	TRIH	TRITURUS	HONGKONGENSIS	ALPINE	NEWT	LIV	150	NO	HK	HK	T	W	C	C	4/28/07	4/29/07	I	LA	AQUACO, INC.
2007930606	TRIH	TRITURUS	HONGKONGENSIS	ALPINE	NEWT	LIV	1,200	NO	CN	CN	T	W	C	C	4/28/07	4/29/07	I	LA	AQUA-NAUTIC SPECIALIST
2007930610	CNP?	CYNOPS	SPECIES	FIREBELLY	NEWT	LIV	155	NO	SG	SG	T	W	C	C	4/28/07	4/29/07	I	LA	TROPICAL FISH CONSOLIDATORS LTD.
2007930640	CYOR	CYNOPS	ORIENTALIS	CHINESE DWARF	NEWT	LIV	2,150	NO	HK	HK	T	C	C	C	4/29/07	4/29/07	I	LA	DOLPHIN INTERNATIONAL
2007930640	PACB	PACHYTRITON	BREVIPES	TSITOU	NEWT	LIV	100	NO	HK	HK	T	C	C	C	4/29/07	4/29/07	I	LA	DOLPHIN INTERNATIONAL
2007930708	TRIH	TRITURUS	HONGKONGENSIS	ALPINE	NEWT	LIV	300	NO	HK	HK	T	W	C	C	3/27/07	3/18/07	I	NY	INTERNATIONAL PET RESOURCES , LLC
2007930739	CYPY	CYNOPS	PYRRHOGASTER	JAPANESE FIREBELLY	NEWT	LIV	210	NO	HK	HK	T	W	C	C	2/21/07	2/18/07	I	NY	TRANSSHIP DISCOUNTS LIMITED
2007930752	CYPY	CYNOPS	PYRRHOGASTER	JAPANESE FIREBELLY	NEWT	LIV	50	NO	HK	HK	T	W	C	C	2/7/07	2/2/07	I	NY	ERDA INCORPORATED
2007931033	CYPY	CYNOPS	PYRRHOGASTER	JAPANESE FIREBELLY	NEWT	LIV	150	NO	HK	HK	T	W	C	C	1/19/07	1/14/07	I	NY	TRANSSHIP DISCOUNTS LIMITED
2007931076	CYPY	CYNOPS	PYRRHOGASTER	JAPANESE FIREBELLY	NEWT	LIV	100	NO	HK	HK	T	C	C	C	2/14/07	2/10/07	I	NY	ERDA INCORPORATED
2007931181	TRIH	TRITURUS	HONGKONGENSIS	ALPINE	NEWT	LIV	50	NO	HK	HK	T	C	C	C	1/24/07	1/22/07	I	NY	TRI-COUNTY TROPICALS, INC.
2007931972	PHNG	PARAMESOTRITON	HONGKONGENSIS	HONG KONG WARTY	NEWT	LIV	2,600	NO	HK	HK	T	C	C	C	5/1/07	4/30/07	I	TP	5-D TROPICAL, INC.
2007932342	CYPY	CYNOPS	PYRRHOGASTER	JAPANESE FIREBELLY	NEWT	LIV	250	NO	HK	HK	T	C	C	C	1/17/07	1/14/07	I	NY	ERDA INCORPORATED
2007932394	NOVI	NOTOPHTHALMUS	VIRIDESCENS	EASTERN	NEWT	LIV	5	NO	US	CA	T	U	C	C	3/14/07	3/14/07	I	BN	SCIENCE KIT
2007932397	NOTO	NOTOPHTHALMUS	SPECIES		NEWT	LIV	4	NO	US	CA	T	U	C	C	3/12/07	3/12/07	I	BN	SCIENCE KIT
2007932596	NOTO	NOTOPHTHALMUS	SPECIES		NEWT	LIV	1	NO	US	CA	T	U	C	C	3/28/07	3/28/07	I	BN	SCIENCE KIT
2007932647	NOTO	NOTOPHTHALMUS	SPECIES		NEWT	LIV	9	NO	US	CA	T	U	C	C	4/11/07	4/9/07	I	BN	SCIENCE KIT
2007934137	CYOR	CYNOPS	ORIENTALIS	CHINESE DWARF	NEWT	LIV	4,000	NO	CN	CN	T	W	C	C	5/4/07	5/4/07	I	LA	Z IMPORTS INC.
2007934760	CYOR	CYNOPS	ORIENTALIS	CHINESE DWARF	NEWT	LIV	1,000	NO	HK	HK	T	C	C	C	5/5/07	5/5/07	I	LA	DOLPHIN INTERNATIONAL
2007934778	TRIT	TRITURUS	SPECIES		NEWT	LIV	200	NO	HK	HK	T	C	C	C	5/5/07	5/5/07	I	LA	GLOBAL AQUATIC CONSULTING
2007934829	TRIH	TRITURUS	HONGKONGENSIS	ALPINE	NEWT	LIV	150	NO	HK	HK	T	W	C	C	5/5/07	5/6/07	I	LA	AQUACO, INC.
2007934856	CYOR	CYNOPS	ORIENTALIS	CHINESE DWARF	NEWT	LIV	1,400	NO	HK	HK	T	C	C	C	5/6/07	5/6/07	I	LA	DOLPHIN INTERNATIONAL
2007934856	PACB	PACHYTRITON	BREVIPES	TSITOU	NEWT	LIV	200	NO	HK	HK	T	C	C	C	5/6/07	5/6/07	I	LA	DOLPHIN INTERNATIONAL
2007934866	TRIT	TRITURUS	SPECIES		NEWT	LIV	200	NO	HK	HK	T	C	C	C	5/6/07	5/6/07	I	LA	AQUATIC CONNECTION
2007934867	CYOR	CYNOPS	ORIENTALIS	CHINESE DWARF	NEWT	LIV	599	NO	CN	CN	T	W	C	C	5/6/07	5/6/07	I	LA	AQUA-NAUTIC SPECIALIST
2007934882	CNP?	CYNOPS	SPECIES	FIREBELLY	NEWT	LIV	300	NO	CN	CN	T	C	C	C	5/7/07	5/7/07	I	AT	SUN PET LTD.
2007936137	NEW?	PACHYTRITON	SPECIES	CHINESE	NEWT	LIV	400	NO	CN	CN	T	C	C	C	5/8/07	5/8/07	I	SF	Golden Phoenix Fisheries, USA
2007937975	CYOR	CYNOPS	ORIENTALIS	CHINESE DWARF	NEWT	LIV	4,000	NO	CN	CN	T	W	C	C	5/10/07	5/10/07	I	LA	Z IMPORTS INC.
2007938748	CYOR	CYNOPS	ORIENTALIS	CHINESE DWARF	NEWT	LIV	500	NO	HK	HK	T	C	C	C	5/12/07	5/12/07	I	LA	DOLPHIN INTERNATIONAL
2007938751	TRIT	TRITURUS	SPECIES		NEWT	LIV	200	NO	HK	HK	T	C	C	C	5/12/07	5/12/07	I	LA	GLOBAL AQUATIC CONSULTING
2007938814	TRIH	TRITURUS	HONGKONGENSIS	ALPINE	NEWT	LIV	250	NO	HK	HK	T	W	C	C	5/12/07	5/13/07	I	LA	AQUACO, INC.
2007938820	CYOR	CYNOPS	ORIENTALIS	CHINESE DWARF	NEWT	LIV	417	NO	CN	CN	T	W	C	C	5/12/07	5/13/07	I	LA	AQUA-NAUTIC SPECIALIST
2007938851	CYOR	CYNOPS	ORIENTALIS	CHINESE DWARF	NEWT	LIV	500	NO	HK	HK	T	C	C	C	5/13/07	5/13/07	I	LA	DOLPHIN INTERNATIONAL
2007938851	PACB	PACHYTRITON	BREVIPES	TSITOU	NEWT	LIV	100	NO	HK	HK	T	C	C	C	5/13/07	5/13/07	I	LA	DOLPHIN INTERNATIONAL
2007939868	PHNG	PARAMESOTRITON	HONGKONGENSIS	HONG KONG WARTY	NEWT	LIV	3,200	NO	HK	HK	T	C	C	C	5/15/07	5/15/07	I	TP	5-D TROPICAL, INC.
2007939868	TRIH	TRITURUS	HONGKONGENSIS	ALPINE	NEWT	LIV	300	NO	HK	HK	T	C	C	C	5/15/07	5/15/07	I	TP	5-D TROPICAL, INC.
2007941202	CYOR	CYNOPS	ORIENTALIS	CHINESE DWARF	NEWT	LIV	5,000	NO	CN	CN	T	W	C	C	5/17/07	5/17/07	I	LA	Z IMPORTS INC.
2007941981	CYOR	CYNOPS	ORIENTALIS	CHINESE DWARF	NEWT	LIV	2,150	NO	HK	HK	T	C	C	C	5/21/07	5/20/07	I	LA	DOLPHIN INTERNATIONAL
2007941981	PACB	PACHYTRITON	BREVIPES	TSITOU	NEWT	LIV	200	NO	HK	HK	T	C	C	C	5/21/07	5/20/07	I	LA	DOLPHIN INTERNATIONAL
2007942001	TRIH	TRITURUS	HONGKONGENSIS	ALPINE	NEWT	LIV	300	NO	CN	CN	T	W	C	C	5/21/07	5/20/07	I	LA	AQUA-NAUTIC SPECIALIST
2007942034	TRIT	TRITURUS	SPECIES		NEWT	LIV	400	NO	HK	HK	T	C	C	C	5/21/07	5/19/07	I	LA	GLOBAL AQUATIC CONSULTING
2007942081	CYOR	CYNOPS	ORIENTALIS	CHINESE DWARF	NEWT	LIV	250	NO	HK	HK	T	C	C	C	5/21/07	5/19/07	I	LA	DOLPHIN INTERNATIONAL
2007942081	PACB	PACHYTRITON	BREVIPES	TSITOU	NEWT	LIV	100	NO	HK	HK	T	C	C	C	5/21/07	5/19/07	I	LA	DOLPHIN INTERNATIONAL
2007942540	TRIH	TRITURUS	HONGKONGENSIS	ALPINE	NEWT	LIV	100	NO	HK	HK	T	W	C	C	5/21/07	5/20/07	I	LA	AQUACO, INC.
2007943443	TRIH	TRITURUS	HONGKONGENSIS	ALPINE	NEWT	LIV	150	NO	HK	HK	T	C	C	C	5/23/07	5/23/07	I	CH	AQUATICS, INC.
2007943640	TYVE	TYLOTOTRITON	VERRUCOSUS	CROCODILE	NEWT	LIV	120	NO	SG	SG	T	W	C	C	4/4/07	4/1/07	I	NY	INTERNATIONAL PET RESOURCES , LLC
2007943662	TYVE	TYLOTOTRITON	VERRUCOSUS	CROCODILE	NEWT	LIV	120	NO	SG	SG	T	W	C	C	4/16/07	4/1/07	I	NY	INTERNATIONAL PET RESOURCES , LLC
2007944030	TRIH	TRITURUS	HONGKONGENSIS	ALPINE	NEWT	LIV	150	NO	HK	HK	T	W	C	C	5/1/07	4/22/07	I	NY	INTERNATIONAL PET RESOURCES , LLC
2007945148	CYPY	CYNOPS	PYRRHOGASTER	JAPANESE FIREBELLY	NEWT	LIV	100	NO	SG	SG	T	W	C	C	4/9/07	4/8/07	I	NY	TRANSSHIP DISCOUNTS LIMITED
2007945303	CYOR	CYNOPS	ORIENTALIS	CHINESE DWARF	NEWT	LIV	500	NO	HK	HK	T	C	C	C	5/26/07	5/26/07	I	LA	DOLPHIN INTERNATIONAL

2007945379	TRIH	TRITURUS	HONGKONGENSIS	ALPINE	NEWT	LIV	50	NO	HK	HK	T	W	C	C	5/27/07	5/27/07	I	LA	AQUACO, INC.
2007945391	TRIH	TRITURUS	HONGKONGENSIS	ALPINE	NEWT	LIV	1,739	NO	CN	CN	T	W	C	C	5/27/07	5/27/07	I	LA	AQUA-NAUTIC SPECIALIST
2007945393	CYOR	CYNOPS	ORIENTALIS	CHINESE DWARF	NEWT	LIV	500	NO	HK	HK	T	C	C	C	5/27/07	5/27/07	I	LA	DOLPHIN INTERNATIONAL
2007945393	PACB	PACHYTRITON	BREVIPES	TSITOU	NEWT	LIV	200	NO	HK	HK	T	C	C	C	5/27/07	5/27/07	I	LA	DOLPHIN INTERNATIONAL
2007945412	CNP?	CYNOPS	SPECIES	FIREBELLY	NEWT	LIV	300	NO	CN	CN	T	C	C	C	5/28/07	5/28/07	I	AT	SUN PET LTD.
2007945592	CYOR	CYNOPS	ORIENTALIS	CHINESE DWARF	NEWT	LIV	40	NO	SG	SG	T	W	C	C	2/8/07	1/28/07	I	NY	INTERNATIONAL PET RESOURCES , LLC
2007945592	TYVE	TYLOTOTRITON	VERRUCOSUS	CROCODILE	NEWT	LIV	100	NO	SG	SG	T	W	C	C	2/8/07	1/28/07	I	NY	INTERNATIONAL PET RESOURCES , LLC
2007945918	CYOR	CYNOPS	ORIENTALIS	CHINESE DWARF	NEWT	LIV	40	NO	SG	SG	T	W	C	C	4/24/07	4/15/07	I	NY	INTERNATIONAL PET RESOURCES , LLC
2007946478	CYPY	CYNOPS	PYRRHOGASTER	JAPANESE FIREBELLY	NEWT	LIV	150	NO	HK	CN	T	W	C	C	2/20/07	2/17/07	I	NY	ERDA INCORPORATED
2007946523	TRIH	TRITURUS	HONGKONGENSIS	ALPINE	NEWT	LIV	50	NO	HK	HK	T	W	C	C	2/13/07	2/4/07	I	NY	INTERNATIONAL PET RESOURCES , LLC
2007946531	PHNG	PARAMESOTRITON	HONGKONGENSIS	HONG KONG WARTY	NEWT	LIV	270	NO	HK	HK	T	C	C	C	5/30/07	5/29/07	I	TP	5-D TROPICAL, INC.
2007946542	TRIH	TRITURUS	HONGKONGENSIS	ALPINE	NEWT	LIV	50	NO	HK	HK	T	W	C	C	2/8/07	1/26/07	I	NY	INTERNATIONAL PET RESOURCES , LLC
2007948503	CYOR	CYNOPS	ORIENTALIS	CHINESE DWARF	NEWT	LIV	500	NO	HK	HK	T	C	C	C	6/2/07	6/2/07	I	LA	DOLPHIN INTERNATIONAL
2007948503	PACB	PACHYTRITON	BREVIPES	TSITOU	NEWT	LIV	100	NO	HK	HK	T	C	C	C	6/2/07	6/2/07	I	LA	DOLPHIN INTERNATIONAL
2007948534	TRIT	TRITURUS	SPECIES		NEWT	LIV	400	NO	HK	HK	T	C	C	C	6/2/07	6/2/07	I	LA	GLOBAL AQUATIC CONSULTING
2007948576	CNP?	CYNOPS	SPECIES	FIREBELLY	NEWT	LIV	80	NO	SG	SG	T	W	C	C	6/2/07	6/3/07	I	LA	TROPICAL FISH CONSOLIDATORS LTD.
2007948584	CYOR	CYNOPS	ORIENTALIS	CHINESE DWARF	NEWT	LIV	1,200	NO	CN	CN	T	W	C	C	6/2/07	6/3/07	I	LA	AQUA-NAUTIC SPECIALIST
2007948593	TRIH	TRITURUS	HONGKONGENSIS	ALPINE	NEWT	LIV	275	NO	HK	HK	T	W	C	C	6/2/07	6/3/07	I	LA	AQUACO, INC.
2007948618	CYOR	CYNOPS	ORIENTALIS	CHINESE DWARF	NEWT	LIV	500	NO	HK	HK	T	C	C	C	6/3/07	6/3/07	I	LA	DOLPHIN INTERNATIONAL
2007948618	PACB	PACHYTRITON	BREVIPES	TSITOU	NEWT	LIV	100	NO	HK	HK	T	C	C	C	6/3/07	6/3/07	I	LA	DOLPHIN INTERNATIONAL
2007950111	CNP?	CYNOPS	SPECIES	FIREBELLY	NEWT	LIV	200	NO	CN	HK	T	W	C	C	6/6/07	6/6/07	I	DN	Bushmaster Reptiles Inc
2007951309	CYPY	CYNOPS	PYRRHOGASTER	JAPANESE FIREBELLY	NEWT	LIV	80	NO	SG	HK	T	C	C	C	3/18/07	4/23/07	I	LA	CITIZEN WATCH CO. OF AMERICA, INC.
2007951712	CYOR	CYNOPS	ORIENTALIS	CHINESE DWARF	NEWT	LIV	250	NO	HK	HK	T	C	C	C	6/9/07	6/9/07	I	LA	DOLPHIN INTERNATIONAL
2007951722	TRIT	TRITURUS	SPECIES		NEWT	LIV	400	NO	HK	HK	T	C	C	C	6/9/07	6/9/07	I	LA	GLOBAL AQUATIC CONSULTING
2007951771	CYOR	CYNOPS	ORIENTALIS	CHINESE DWARF	NEWT	LIV	2,400	NO	HK	HK	T	C	C	C	6/9/07	6/10/07	I	LA	DOLPHIN INTERNATIONAL
2007951771	PACB	PACHYTRITON	BREVIPES	TSITOU	NEWT	LIV	200	NO	HK	HK	T	C	C	C	6/9/07	6/10/07	I	LA	DOLPHIN INTERNATIONAL
2007951790	TRIH	TRITURUS	HONGKONGENSIS	ALPINE	NEWT	LIV	150	NO	HK	HK	T	W	C	C	6/9/07	6/10/07	I	LA	AQUACO, INC.
2007951797	CYOR	CYNOPS	ORIENTALIS	CHINESE DWARF	NEWT	LIV	1,797	NO	CN	CN	T	W	C	C	6/9/07	6/10/07	I	LA	AQUA-NAUTIC SPECIALIST
2007951800	TRIT	TRITURUS	SPECIES		NEWT	LIV	200	NO	HK	HK	T	W	C	C	6/9/07	6/10/07	I	LA	AQUATIC CONNECTION
2007951832	CNP?	CYNOPS	SPECIES	FIREBELLY	NEWT	LIV	578	NO	CN	CN	T	C	C	C	6/11/07	6/11/07	I	AT	SUN PET LTD.
2007951832	PLAB	PACHYTRITON	LABIATUS	UNTERSTEIN'S	NEWT	LIV	100	NO	CN	CN	T	C	C	C	6/11/07	6/11/07	I	AT	SUN PET LTD.
2007952785	NEW?	PACHYTRITON	SPECIES	CHINESE	NEWT	LIV	200	NO	CN	CN	T	C	C	C	6/12/07	6/12/07	I	SF	Golden Phoenix Fisheries, USA
2007954783	CYOR	CYNOPS	ORIENTALIS	CHINESE DWARF	NEWT	LIV	3,000	NO	CN	CN	T	W	C	C	6/15/07	6/15/07	I	LA	Z IMPORTS INC.
2007955161	TRIT	TRITURUS	SPECIES		NEWT	LIV	200	NO	HK	HK	T	C	C	C	6/16/07	6/16/07	I	LA	GLOBAL AQUATIC CONSULTING
2007955216	CYOR	CYNOPS	ORIENTALIS	CHINESE DWARF	NEWT	LIV	1,750	NO	HK	HK	T	C	C	C	6/16/07	6/17/07	I	LA	DOLPHIN INTERNATIONAL
2007955216	PACB	PACHYTRITON	BREVIPES	TSITOU	NEWT	LIV	200	NO	HK	HK	T	C	C	C	6/16/07	6/17/07	I	LA	DOLPHIN INTERNATIONAL
2007955249	TRIH	TRITURUS	HONGKONGENSIS	ALPINE	NEWT	LIV	150	NO	HK	HK	T	W	C	C	6/16/07	6/17/07	I	LA	AQUACO, INC.
2007955250	CYOR	CYNOPS	ORIENTALIS	CHINESE DWARF	NEWT	LIV	1,020	NO	CN	CN	T	W	C	C	6/16/07	6/17/07	I	LA	AQUA-NAUTIC SPECIALIST
2007955284	CNP?	CYNOPS	SPECIES	FIREBELLY	NEWT	LIV	300	NO	CN	CN	T	C	C	C	6/18/07	6/18/07	I	AT	SUN PET LTD.
2007958964	CYOR	CYNOPS	ORIENTALIS	CHINESE DWARF	NEWT	LIV	3,000	NO	CN	CN	T	W	C	C	6/22/07	6/22/07	I	LA	Z IMPORTS INC.
2007959394	CYOR	CYNOPS	ORIENTALIS	CHINESE DWARF	NEWT	LIV	750	NO	HK	HK	T	C	C	C	6/23/07	6/23/07	I	LA	DOLPHIN INTERNATIONAL
2007959394	PACB	PACHYTRITON	BREVIPES	TSITOU	NEWT	LIV	100	NO	HK	HK	T	C	C	C	6/23/07	6/23/07	I	LA	DOLPHIN INTERNATIONAL
2007959472	TRIT	TRITURUS	SPECIES		NEWT	LIV	50	NO	HK	HK	T	W	C	C	6/23/07	6/24/07	I	LA	AQUACO, INC.
2007959493	CYOR	CYNOPS	ORIENTALIS	CHINESE DWARF	NEWT	LIV	500	NO	HK	HK	T	C	C	C	6/23/07	6/24/07	I	LA	DOLPHIN INTERNATIONAL
2007959493	PACB	PACHYTRITON	BREVIPES	TSITOU	NEWT	LIV	100	NO	HK	HK	T	C	C	C	6/23/07	6/24/07	I	LA	DOLPHIN INTERNATIONAL
2007959510	CYOR	CYNOPS	ORIENTALIS	CHINESE DWARF	NEWT	LIV	600	NO	CN	CN	T	W	C	C	6/24/07	6/24/07	I	LA	AQUA-NAUTIC SPECIALIST
2007959518	TRIT	TRITURUS	SPECIES		NEWT	LIV	200	NO	HK	HK	T	W	C	C	6/24/07	6/24/07	I	LA	AQUATIC CONNECTION
2007960385	BODO	BOLITOGLOSSA	DOFLEINI	ALTA VERAPAZ	SALAMANDER	LIV	13	NO	GT	GT	T	W	C	C	6/26/07	6/25/07	I	MI	D.H.A.R. TROPICALS LLC
2007960444	TYVE	TYLOTOTRITON	VERRUCOSUS	CROCODILE	NEWT	LIV	20	NO	HK	HK	T	C	C	C	6/26/07	6/25/07	I	MI	NAME REMOVED
2007962727	TYVE	TYLOTOTRITON	VERRUCOSUS	CROCODILE	NEWT	LIV	1,000	NO	HK	HK	T	W	C	C	6/29/07	6/29/07	I	LA	CALIFORNIA ZOOLOGICAL SUPPLY
2007962727	TYL?	TYLOTOTRITON	SPECIES	CROCODILE	NEWT	LIV	100	NO	HK	HK	T	W	C	C	6/29/07	6/29/07	I	LA	CALIFORNIA ZOOLOGICAL SUPPLY
2007963116	CYOR	CYNOPS	ORIENTALIS	CHINESE DWARF	NEWT	LIV	250	NO	HK	HK	T	C	C	C	6/30/07	6/30/07	I	LA	DOLPHIN INTERNATIONAL
2007963125	TRIT	TRITURUS	SPECIES		NEWT	LIV	200	NO	HK	HK	T	W	C	C	6/30/07	6/30/07	I	LA	GLOBAL AQUATIC CONSULTING
2007963175	TRIH	TRITURUS	HONGKONGENSIS	ALPINE	NEWT	LIV	400	NO	HK	HK	T	W	C	C	6/30/07	7/1/07	I	LA	AQUACO, INC.
2007963205	CYOR	CYNOPS	ORIENTALIS	CHINESE DWARF	NEWT	LIV	750	NO	HK	HK	T	C	C	C	7/1/07	7/1/07	I	LA	DOLPHIN INTERNATIONAL
2007963205	PACB	PACHYTRITON	BREVIPES	TSITOU	NEWT	LIV	600	NO	HK	HK	T	C	C	C	7/1/07	7/1/07	I	LA	DOLPHIN INTERNATIONAL
2007963224	CYOR	CYNOPS	ORIENTALIS	CHINESE DWARF	NEWT	LIV	300	NO	CN	CN	T	W	C	C	7/1/07	7/1/07	I	LA	AQUA-NAUTIC SPECIALIST
2007963227	CNP?	CYNOPS	SPECIES	FIREBELLY	NEWT	LIV	300	NO	CN	CN	T	C	C	C	7/2/07	7/2/07	I	AT	SUN PET LTD.
2007963924	TYKW	TYLOTOTRITON	KWEICHOWENSIS	KWEICHOW CROCODILE	NEWT	LIV	520	NO	CN	HK	T	W	C	C	7/3/07	7/3/07	I	DN	Bushmaster Reptiles Inc
2007965457	CYOR	CYNOPS	ORIENTALIS	CHINESE DWARF	NEWT	LIV	2,000	NO	CN	CN	T	W	C	C	7/6/07	7/6/07	I	LA	Z IMPORTS INC.

2007965457	TYVE	TYLOTOTRITON	VERRUCOSUS	CROCODILE	NEWT	LIV	600	NO	CN	CN	T	W	C	C	7/6/07	7/6/07	I	LA	Z IMPORTS INC.
2007965833	CYOR	CYNOPS	ORIENTALIS	CHINESE DWARF	NEWT	LIV	250	NO	HK	HK	T	C	C	C	7/6/07	7/7/07	I	LA	DOLPHIN INTERNATIONAL
2007966010	TRIT	TRITURUS	SPECIES		NEWT	LIV	200	NO	HK	HK	T	W	C	C	7/7/07	7/7/07	I	LA	GLOBAL AQUATIC CONSULTING
2007966010	CYOR	CYNOPS	ORIENTALIS	CHINESE DWARF	NEWT	LIV	100	NO	HK	HK	T	W	C	C	7/7/07	7/7/07	I	LA	GLOBAL AQUATIC CONSULTING
2007966073	CYOR	CYNOPS	ORIENTALIS	CHINESE DWARF	NEWT	LIV	750	NO	HK	HK	T	C	C	C	7/7/07	7/8/07	I	LA	DOLPHIN INTERNATIONAL
2007966073	PACB	PACHYTRITON	BREVIPES	TSITOU	NEWT	LIV	100	NO	HK	HK	T	C	C	C	7/7/07	7/8/07	I	LA	DOLPHIN INTERNATIONAL
2007966114	TRIT	TRITURUS	SPECIES		NEWT	LIV	200	NO	HK	HK	T	W	C	C	7/8/07	7/8/07	I	LA	AQUATIC CONNECTION
2007966133	CNP?	CYNOPS	SPECIES	FIREBELLY	NEWT	LIV	300	NO	CN	CN	T	C	C	C	7/9/07	7/9/07	I	AT	SUN PET LTD.
2007966133	PLAB	PACHYTRITON	LABIATUS	UNTERSTEIN'S	NEWT	LIV	33	NO	CN	CN	T	C	C	C	7/9/07	7/9/07	I	AT	SUN PET LTD.
2007968425	TYVE	TYLOTOTRITON	VERRUCOSUS	CROCODILE	NEWT	LIV	120	NO	HK	HK	T	W	C	C	7/11/07	7/11/07	I	MI	TWO AMIGOS IMPORT & EXPORT, INC.
2007970134	CYOR	CYNOPS	ORIENTALIS	CHINESE DWARF	NEWT	LIV	250	NO	HK	HK	T	C	C	C	7/13/07	7/14/07	I	LA	DOLPHIN INTERNATIONAL
2007970232	TRIT	TRITURUS	SPECIES		NEWT	LIV	200	NO	HK	HK	T	W	C	C	7/14/07	7/14/07	I	LA	GLOBAL AQUATIC CONSULTING
2007970302	CNP?	CYNOPS	SPECIES	FIREBELLY	NEWT	LIV	50	NO	SG	SG	T	W	C	C	7/14/07	7/15/07	I	LA	TROPICAL FISH CONSOLIDATORS LTD.
2007970312	CYOR	CYNOPS	ORIENTALIS	CHINESE DWARF	NEWT	LIV	250	NO	HK	HK	T	C	C	C	7/14/07	7/15/07	I	LA	DOLPHIN INTERNATIONAL
2007970312	PACB	PACHYTRITON	BREVIPES	TSITOU	NEWT	LIV	100	NO	HK	HK	T	C	C	C	7/14/07	7/15/07	I	LA	DOLPHIN INTERNATIONAL
2007970330	TRIH	TRITURUS	HONGKONGENSIS	ALPINE	NEWT	LIV	100	NO	HK	HK	T	C	C	C	7/14/07	7/15/07	I	LA	AQUACO, INC.
2007970387	CNP?	CYNOPS	SPECIES	FIREBELLY	NEWT	LIV	300	NO	CN	CN	T	C	C	C	7/16/07	7/16/07	I	AT	SUN PET LTD.
2007971653	TRIH	TRITURUS	HONGKONGENSIS	ALPINE	NEWT	LIV	1,600	NO	HK	HK	T	C	C	C	7/17/07	7/17/07	I	TP	5-D TROPICAL, INC.
2007971891	CYPY	CYNOPS	PYRRHOGASTER	JAPANESE FIREBELLY	NEWT	LIV	20	NO	JP	JP	S	W	C	C	7/18/07	7/18/07	I	SF	UNIVERSITY OF CALIFORNIA DAVIS
2007972045	CYOR	CYNOPS	ORIENTALIS	CHINESE DWARF	NEWT	LIV	150	NO	HK	HK	T	W	C	C	7/18/07	7/18/07	I	CH	AQUATICS, INC.
2007973755	CYOR	CYNOPS	ORIENTALIS	CHINESE DWARF	NEWT	LIV	2,000	NO	CN	CN	T	W	C	C	7/20/07	7/20/07	I	LA	Z IMPORTS INC.
2007974138	CYOR	CYNOPS	ORIENTALIS	CHINESE DWARF	NEWT	LIV	500	NO	HK	HK	T	C	C	C	7/20/07	7/21/07	I	LA	DOLPHIN INTERNATIONAL
2007974138	PACB	PACHYTRITON	BREVIPES	TSITOU	NEWT	LIV	200	NO	HK	HK	T	C	C	C	7/20/07	7/21/07	I	LA	DOLPHIN INTERNATIONAL
2007974228	TRIT	TRITURUS	SPECIES		NEWT	LIV	400	NO	HK	HK	T	W	C	C	7/21/07	7/21/07	I	LA	GLOBAL AQUATIC CONSULTING
2007974333	TRIT	TRITURUS	SPECIES		NEWT	LIV	400	NO	HK	HK	T	W	C	C	7/21/07	7/22/07	I	LA	AQUATIC CONNECTION
2007974339	CYOR	CYNOPS	ORIENTALIS	CHINESE DWARF	NEWT	LIV	1,750	NO	HK	HK	T	C	C	C	7/21/07	7/22/07	I	LA	DOLPHIN INTERNATIONAL
2007974339	PACB	PACHYTRITON	BREVIPES	TSITOU	NEWT	LIV	200	NO	HK	HK	T	C	C	C	7/21/07	7/22/07	I	LA	DOLPHIN INTERNATIONAL
2007974361	TRIH	TRITURUS	HONGKONGENSIS	ALPINE	NEWT	LIV	78	NO	HK	HK	T	W	C	C	7/21/07	7/22/07	I	LA	AQUACO, INC.
2007974395	CYOR	CYNOPS	ORIENTALIS	CHINESE DWARF	NEWT	LIV	900	NO	CN	CN	T	C	C	C	7/22/07	7/22/07	I	LA	AQUA-NAUTIC SPECIALIST
2007975933	CYPY	CYNOPS	PYRRHOGASTER	JAPANESE FIREBELLY	NEWT	LIV	20	NO	JP	JP	M	W	C	C	7/25/07	7/25/07	I	SF	UNIVERSITY OF CALIFORNIA DAVIS
2007977331	CYOR	CYNOPS	ORIENTALIS	CHINESE DWARF	NEWT	LIV	3,600	NO	CN	CN	T	W	C	C	7/27/07	7/27/07	I	LA	Z IMPORTS INC.
2007977685	CYOR	CYNOPS	ORIENTALIS	CHINESE DWARF	NEWT	LIV	500	NO	HK	HK	T	C	C	C	7/27/07	7/28/07	I	LA	DOLPHIN INTERNATIONAL
2007977685	PACB	PACHYTRITON	BREVIPES	TSITOU	NEWT	LIV	100	NO	HK	HK	T	C	C	C	7/27/07	7/28/07	I	LA	DOLPHIN INTERNATIONAL
2007977785	TRIT	TRITURUS	SPECIES		NEWT	LIV	200	NO	HK	HK	T	W	C	C	7/28/07	7/28/07	I	LA	GLOBAL AQUATIC CONSULTING
2007977862	CYOR	CYNOPS	ORIENTALIS	CHINESE DWARF	NEWT	LIV	100	NO	SG	SG	T	W	C	C	7/28/07	7/29/07	I	LA	TROPICAL FISH CONSOLIDATORS LTD.
2007977880	TRIH	TRITURUS	HONGKONGENSIS	ALPINE	NEWT	LIV	450	NO	HK	HK	T	W	C	C	7/28/07	7/29/07	I	LA	AQUACO, INC.
2007977885	CYOR	CYNOPS	ORIENTALIS	CHINESE DWARF	NEWT	LIV	1,000	NO	HK	HK	T	C	C	C	7/28/07	7/29/07	I	LA	DOLPHIN INTERNATIONAL
2007977901	TRIH	TRITURUS	HONGKONGENSIS	ALPINE	NEWT	LIV	20	NO	CN	CN	T	C	C	C	7/29/07	7/29/07	I	LA	AQUA-NAUTIC SPECIALIST
2007977938	PLAB	PACHYTRITON	LABIATUS	UNTERSTEIN'S	NEWT	LIV	80	NO	CN	CN	T	C	C	C	7/30/07	7/30/07	I	AT	SUN PET LTD.
2007978754	BODO	BOLITOGLOSSA	DOFLEINI	ALTA VERAPAZ	SALAMANDER	LIV	14	NO	GT	GT	T	W	C	C	7/30/07	7/29/07	I	MI	D.H.A.R. TROPICALS LLC
2007981556	CYOR	CYNOPS	ORIENTALIS	CHINESE DWARF	NEWT	LIV	500	NO	HK	HK	T	C	C	C	8/3/07	8/4/07	I	LA	DOLPHIN INTERNATIONAL
2007981652	TRIT	TRITURUS	SPECIES		NEWT	LIV	200	NO	HK	HK	T	W	C	C	8/4/07	8/4/07	I	LA	GLOBAL AQUATIC CONSULTING
2007981708	CYOR	CYNOPS	ORIENTALIS	CHINESE DWARF	NEWT	LIV	4,750	NO	HK	HK	T	C	C	C	8/4/07	8/5/07	I	LA	DOLPHIN INTERNATIONAL
2007981708	PACB	PACHYTRITON	BREVIPES	TSITOU	NEWT	LIV	100	NO	HK	HK	T	C	C	C	8/4/07	8/5/07	I	LA	DOLPHIN INTERNATIONAL
2007981735	TRIH	TRITURUS	HONGKONGENSIS	ALPINE	NEWT	LIV	46	NO	HK	HK	T	W	C	C	8/4/07	8/5/07	I	LA	AQUACO, INC.
2007984767	CYOR	CYNOPS	ORIENTALIS	CHINESE DWARF	NEWT	LIV	3,000	NO	CN	CN	T	W	C	C	8/10/07	8/10/07	I	LA	Z IMPORTS INC.
2007984769	CYPY	CYNOPS	PYRRHOGASTER	JAPANESE FIREBELLY	NEWT	LIV	20	NO	JP	JP	M	W	C	C	8/10/07	8/10/07	I	SF	UNIVERSITY OF CALIFORNIA DAVIS
2007985134	CYOR	CYNOPS	ORIENTALIS	CHINESE DWARF	NEWT	LIV	500	NO	HK	HK	T	C	C	C	8/11/07	8/11/07	I	LA	DOLPHIN INTERNATIONAL
2007985151	TRIT	TRITURUS	SPECIES		NEWT	LIV	200	NO	HK	HK	T	W	C	C	8/11/07	8/11/07	I	LA	GLOBAL AQUATIC CONSULTING
2007985247	CYOR	CYNOPS	ORIENTALIS	CHINESE DWARF	NEWT	LIV	1,750	NO	HK	HK	T	C	C	C	8/11/07	8/12/07	I	LA	DOLPHIN INTERNATIONAL
2007985247	PACB	PACHYTRITON	BREVIPES	TSITOU	NEWT	LIV	200	NO	HK	HK	T	C	C	C	8/11/07	8/12/07	I	LA	DOLPHIN INTERNATIONAL
2007985264	TRIT	TRITURUS	SPECIES		NEWT	LIV	200	NO	HK	HK	T	C	C	C	8/11/07	8/12/07	I	LA	AQUATIC CONNECTION
2007985268	TRIH	TRITURUS	HONGKONGENSIS	ALPINE	NEWT	LIV	150	NO	CN	CN	T	C	C	C	8/11/07	8/12/07	I	LA	AQUA-NAUTIC SPECIALIST
2007985275	TRIH	TRITURUS	HONGKONGENSIS	ALPINE	NEWT	LIV	50	NO	HK	HK	T	C	C	C	8/11/07	8/12/07	I	LA	AQUACO, INC.
2007985842	CYOR	CYNOPS	ORIENTALIS	CHINESE DWARF	NEWT	LIV	1,000	NO	CN	CN	T	W	C	C	8/13/07	8/13/07	I	LA	WORLD WIDE TRADING INC.
2007985842	PLAB	PACHYTRITON	LABIATUS	UNTERSTEIN'S	NEWT	LIV	200	NO	CN	CN	T	W	C	C	8/13/07	8/13/07	I	LA	WORLD WIDE TRADING INC.
2007986103	NEW?	PACHYTRITON	SPECIES	CHINESE	NEWT	LIV	290	NO	CN	CN	T	C	C	C	8/14/07	8/14/07	I	SF	Golden Phoenix Fisheries, USA
2007988166	CYOR	CYNOPS	ORIENTALIS	CHINESE DWARF	NEWT	LIV	8,000	NO	CN	CN	T	W	C	C	8/17/07	8/17/07	I	LA	Z IMPORTS INC.
2007988280	CYOR	CYNOPS	ORIENTALIS	CHINESE DWARF	NEWT	LIV	500	NO	HK	HK	T	C	C	C	8/18/07	8/18/07	I	LA	DOLPHIN INTERNATIONAL
2007988387	CYOR	CYNOPS	ORIENTALIS	CHINESE DWARF	NEWT	LIV	1,250	NO	HK	HK	T	C	C	C	8/19/07	8/19/07	I	LA	DOLPHIN INTERNATIONAL

2007988387	PACB	PACHYTRITON	BREVIPES	TSITOU	NEWT	LIV	300	NO	HK	HK	T	C	C	C	8/19/07	8/19/07	I	LA	DOLPHIN INTERNATIONAL
2007988430	TRIH	TRITURUS	HONGKONGENSIS	ALPINE	NEWT	LIV	250	NO	HK	HK	T	W	C	C	8/19/07	8/19/07	I	LA	AQUACO, INC.
2007988431	CYOR	CYNOPS	ORIENTALIS	CHINESE DWARF	NEWT	LIV	1,200	NO	CN	CN	T	W	C	C	8/19/07	8/19/07	I	LA	AQUA-NAUTIC SPECIALIST
2007988445	CNP?	CYNOPS	SPECIES	FIREBELLY	NEWT	LIV	300	NO	CN	CN	T	C	C	C	8/20/07	8/20/07	I	AT	SUN PET LTD.
2007989236	TRIH	TRITURUS	HONGKONGENSIS	ALPINE	NEWT	LIV	1,600	NO	HK	HK	T	C	C	C	8/21/07	8/22/07	I	TP	5-D TROPICAL, INC.
2007990921	CYOR	CYNOPS	ORIENTALIS	CHINESE DWARF	NEWT	LIV	2,000	NO	CN	CN	T	W	C	C	8/24/07	8/24/07	I	LA	Z IMPORTS INC.
2007990921	TYVE	TYLOTOTRITON	VERRUCOSUS	CROCODILE	NEWT	LIV	300	NO	CN	CN	T	W	C	C	8/24/07	8/24/07	I	LA	Z IMPORTS INC.
2007991046	CYOR	CYNOPS	ORIENTALIS	CHINESE DWARF	NEWT	LIV	500	NO	HK	HK	T	C	C	C	8/24/07	8/25/07	I	LA	DOLPHIN INTERNATIONAL
2007991046	PACB	PACHYTRITON	BREVIPES	TSITOU	NEWT	LIV	100	NO	HK	HK	T	C	C	C	8/24/07	8/25/07	I	LA	DOLPHIN INTERNATIONAL
2007991166	TRIT	TRITURUS	SPECIES		NEWT	LIV	200	NO	HK	HK	T	W	C	C	8/25/07	8/25/07	I	LA	GLOBAL AQUATIC CONSULTING
2007991219	CYOR	CYNOPS	ORIENTALIS	CHINESE DWARF	NEWT	LIV	1,500	NO	HK	HK	T	C	C	C	8/25/07	8/26/07	I	LA	DOLPHIN INTERNATIONAL
2007991219	PACB	PACHYTRITON	BREVIPES	TSITOU	NEWT	LIV	300	NO	HK	HK	T	C	C	C	8/25/07	8/26/07	I	LA	DOLPHIN INTERNATIONAL
2007991231	CNP?	CYNOPS	SPECIES	FIREBELLY	NEWT	LIV	100	NO	SG	SG	T	W	C	C	8/25/07	8/26/07	I	LA	TROPICAL FISH CONSOLIDATORS LTD.
2007991253	CYOR	CYNOPS	ORIENTALIS	CHINESE DWARF	NEWT	LIV	2,579	NO	CN	CN	T	C	C	C	8/26/07	8/26/07	I	LA	AQUA-NAUTIC SPECIALIST
2007991261	TRIH	TRITURUS	HONGKONGENSIS	ALPINE	NEWT	LIV	150	NO	VS	HK	T	W	C	C	8/26/07	8/26/07	I	LA	AQUACO, INC.
2007991291	PLAB	PACHYTRITON	LABIATUS	UNTERSTEIN'S	NEWT	LIV	80	NO	CN	CN	T	C	C	C	8/27/07	8/27/07	I	AT	SUN PET LTD.
2007992531	CYOR	CYNOPS	ORIENTALIS	CHINESE DWARF	NEWT	LIV	150	NO	HK	HK	T	W	C	C	8/29/07	8/29/07	I	CH	AQUATICS, INC.
2007993798	CYOR	CYNOPS	ORIENTALIS	CHINESE DWARF	NEWT	LIV	250	NO	HK	HK	T	C	C	C	9/1/07	9/1/07	I	LA	DOLPHIN INTERNATIONAL
2007993798	PACB	PACHYTRITON	BREVIPES	TSITOU	NEWT	LIV	200	NO	HK	HK	T	C	C	C	9/1/07	9/1/07	I	LA	DOLPHIN INTERNATIONAL
2007993822	TRIH	TRITURUS	HONGKONGENSIS	ALPINE	NEWT	LIV	150	NO	VS	HK	T	W	C	C	9/2/07	9/2/07	I	LA	AQUACO, INC.
2007993827	CYOR	CYNOPS	ORIENTALIS	CHINESE DWARF	NEWT	LIV	447	NO	CN	CN	T	C	C	C	9/2/07	9/2/07	I	LA	AQUA-NAUTIC SPECIALIST
2007993835	CYOR	CYNOPS	ORIENTALIS	CHINESE DWARF	NEWT	LIV	1,600	NO	HK	HK	T	C	C	C	9/2/07	9/2/07	I	LA	DOLPHIN INTERNATIONAL
2007993835	PACB	PACHYTRITON	BREVIPES	TSITOU	NEWT	LIV	100	NO	HK	HK	T	C	C	C	9/2/07	9/2/07	I	LA	DOLPHIN INTERNATIONAL
2007993852	CNP?	CYNOPS	SPECIES	FIREBELLY	NEWT	LIV	300	NO	CN	CN	T	C	C	C	9/3/07	9/3/07	I	AT	SUN PET LTD.
2007994303	PHNG	PARAMESOTRITON	HONGKONGENSIS	HONG KONG WARTY	NEWT	LIV	1,600	NO	HK	HK	T	C	C	C	9/4/07	9/5/07	I	TP	5-D TROPICAL, INC.
2007995439	SAL?	SALAMANDRA	SPECIES	FIRE	SALAMANDER	LIV	500	NO	CN	CN	T	W	C	C	9/6/07	9/1/07	I	LA	FISH IN THE BAG, INC.
2007995574	CYOR	CYNOPS	ORIENTALIS	CHINESE DWARF	NEWT	LIV	200	NO	SG	SG	T	W	C	C	6/13/07	6/3/07	I	NY	INTERNATIONAL PET RESOURCES , LLC
2007996319	CYOR	CYNOPS	ORIENTALIS	CHINESE DWARF	NEWT	LIV	100	NO	SG	SG	T	W	C	C	7/12/07	6/17/07	I	NY	INTERNATIONAL PET RESOURCES , LLC
2007996372	CYPY	CYNOPS	PYRRHOGASTER	JAPANESE FIREBELLY	NEWT	LIV	440	NO	HK	HK	T	W	C	C	7/5/07	6/30/07	I	NY	ERDA INCORPORATED
2007996585	TRIT	TRITURUS	SPECIES		NEWT	LIV	400	NO	HK	HK	T	W	C	C	9/8/07	9/8/07	I	LA	GLOBAL AQUATIC CONSULTING
2007996603	CYOR	CYNOPS	ORIENTALIS	CHINESE DWARF	NEWT	LIV	250	NO	HK	HK	T	C	C	C	9/8/07	9/8/07	I	LA	DOLPHIN INTERNATIONAL
2007996679	CYOR	CYNOPS	ORIENTALIS	CHINESE DWARF	NEWT	LIV	1,850	NO	HK	HK	T	C	C	C	9/9/07	9/9/07	I	LA	DOLPHIN INTERNATIONAL
2007996679	PACB	PACHYTRITON	BREVIPES	TSITOU	NEWT	LIV	100	NO	HK	HK	T	C	C	C	9/9/07	9/9/07	I	LA	DOLPHIN INTERNATIONAL
2007996697	TRIH	TRITURUS	HONGKONGENSIS	ALPINE	NEWT	LIV	300	NO	VS	HK	T	W	C	C	9/9/07	9/9/07	I	LA	AQUACO, INC.
2007996719	CNP?	CYNOPS	SPECIES	FIREBELLY	NEWT	LIV	300	NO	CN	CN	T	C	C	C	9/10/07	9/10/07	I	AT	SUN PET LTD.
2007996879	CYPY	CYNOPS	PYRRHOGASTER	JAPANESE FIREBELLY	NEWT	LIV	140	NO	HK	HK	T	W	C	C	4/10/07	4/7/07	I	NY	ERDA INCORPORATED
2007996902	NRR?	NEURERGUS	SPECIES		NEWT	LIV	20	NO	FR	FR	T	C	C	C	8/15/07	8/15/07	I	MI	TWO AMIGOS IMPORT & EXPORT, INC.
2007996902	SASA	SALAMANDRA	SALAMANDRA	EUROPEAN FIRE	SALAMANDER	LIV	5	NO	FR	FR	T	C	C	C	8/15/07	8/15/07	I	MI	TWO AMIGOS IMPORT & EXPORT, INC.
2007997183	CYPY	CYNOPS	PYRRHOGASTER	JAPANESE FIREBELLY	NEWT	LIV	50	NO	HK	HK	T	W	C	C	6/17/07	6/17/07	I	NY	TRANSSHIP DISCOUNTS LIMITED
2007997212	CYOR	CYNOPS	ORIENTALIS	CHINESE DWARF	NEWT	LIV	300	NO	SG	SG	T	W	C	C	5/27/07	5/27/07	I	NY	INTERNATIONAL PET RESOURCES , LLC
2007997290	CYPY	CYNOPS	PYRRHOGASTER	JAPANESE FIREBELLY	NEWT	LIV	150	NO	HK	HK	T	W	C	C	8/9/07	8/5/07	I	NY	TRANSSHIP DISCOUNTS LIMITED
2007997768	CYOR	CYNOPS	ORIENTALIS	CHINESE DWARF	NEWT	LIV	400	NO	SG	SG	T	C	C	C	5/30/07	5/20/07	I	NY	INTERNATIONAL PET RESOURCES , LLC
2007997824	CYPY	CYNOPS	PYRRHOGASTER	JAPANESE FIREBELLY	NEWT	LIV	210	NO	HK	HK	T	W	C	C	8/7/07	8/4/07	I	NY	ERDA INCORPORATED
2007998428	CYPY	CYNOPS	PYRRHOGASTER	JAPANESE FIREBELLY	NEWT	LIV	50	NO	HK	HK	T	W	C	C	4/10/07	4/8/07	I	NY	TRANSSHIP DISCOUNTS LIMITED
2007999322	TYVE	TYLOTOTRITON	VERRUCOSUS	CROCODILE	NEWT	LIV	400	NO	CN	CN	T	W	C	C	6/13/07	6/13/07	I	LA	GOLDEN POND
2007999466	CYPY	CYNOPS	PYRRHOGASTER	JAPANESE FIREBELLY	NEWT	LIV	220	NO	HK	HK	T	W	C	C	6/1/07	5/19/07	I	NY	ERDA INCORPORATED
2007999686	CYOR	CYNOPS	ORIENTALIS	CHINESE DWARF	NEWT	LIV	1,000	NO	CN	CN	T	W	C	C	9/13/07	9/13/07	I	LA	WORLD WIDE TRADING INC.
2008151818	CYPY	CYNOPS	PYRRHOGASTER	JAPANESE FIREBELLY	NEWT	LIV	60	NO	HK	HK	T	W	C	C	1/2/08	12/30/07	I	NY	Transship Discounts Ltd.
2008153140	CYOR	CYNOPS	ORIENTALIS	CHINESE DWARF	NEWT	LIV	500	NO	HK	HK	T	C	C	C	1/5/08	1/5/08	I	LA	DOLPHIN INTERNATIONAL
2008153216	TRIH	TRITURUS	HONGKONGENSIS	ALPINE	NEWT	LIV	250	NO	HK	HK	T	W	C	C	1/5/08	1/6/08	I	LA	AQUACO, INC.
2008153232	CYOR	CYNOPS	ORIENTALIS	CHINESE DWARF	NEWT	LIV	850	NO	HK	HK	T	C	C	C	1/5/08	1/6/08	I	LA	DOLPHIN INTERNATIONAL
2008154019	CYPY	CYNOPS	PYRRHOGASTER	JAPANESE FIREBELLY	NEWT	LIV	50	NO	HK	HK	T	W	C	C	1/8/08	1/6/08	I	NY	Transship Discounts Ltd.
2008156278	CYOR	CYNOPS	ORIENTALIS	CHINESE DWARF	NEWT	LIV	1,380	NO	HK	HK	T	C	C	C	1/13/08	1/13/08	I	LA	DOLPHIN INTERNATIONAL
2008156278	PACB	PACHYTRITON	BREVIPES	TSITOU	NEWT	LIV	300	NO	HK	HK	T	C	C	C	1/13/08	1/13/08	I	LA	DOLPHIN INTERNATIONAL
2008156294	TRIH	TRITURUS	HONGKONGENSIS	ALPINE	NEWT	LIV	50	NO	ID	HK	T	W	C	C	1/13/08	1/13/08	I	LA	AQUACO, INC.
2008156297	CYOR	CYNOPS	ORIENTALIS	CHINESE DWARF	NEWT	LIV	1,500	NO	HK	HK	T	W	C	C	1/13/08	1/13/08	I	LA	AQUA-NAUTIC SPECIALIST
2008156320	CYPY	CYNOPS	PYRRHOGASTER	JAPANESE FIREBELLY	NEWT	LIV	300	NO	CN	CN	T	C	C	C	1/14/08	1/14/08	I	AT	SUN PET LTD.
2008157114	TRIH	TRITURUS	HONGKONGENSIS	ALPINE	NEWT	LIV	1,600	NO	HK	HK	T	C	C	C	1/15/08	1/15/08	I	TP	5-D TROPICAL INC.
2008157561	CYPY	CYNOPS	PYRRHOGASTER	JAPANESE FIREBELLY	NEWT	LIV	70	NO	HK	HK	T	W	C	C	1/14/08	1/13/08	I	NY	Transship Discounts Ltd.
2008159232	CYOR	CYNOPS	ORIENTALIS	CHINESE DWARF	NEWT	LIV	250	NO	HK	HK	T	C	C	C	1/19/08	1/19/08	I	LA	DOLPHIN INTERNATIONAL

2008159315	CYOR	CYNOPS	ORIENTALIS	CHINESE DWARF	NEWT	LIV	1,040	NO	HK	HK	T	W	C	C	1/19/08	1/20/08	I	LA	AQUA-NAUTIC SPECIALIST
2008159322	CYOR	CYNOPS	ORIENTALIS	CHINESE DWARF	NEWT	LIV	1,940	NO	HK	HK	T	C	C	C	1/19/08	1/20/08	I	LA	DOLPHIN INTERNATIONAL
2008159322	PACB	PACHYTRITON	BREVIPES	TSITOU	NEWT	LIV	100	NO	HK	HK	T	C	C	C	1/19/08	1/20/08	I	LA	DOLPHIN INTERNATIONAL
2008159381	CYPY	CYNOPS	PYRRHOGASTER	JAPANESE FIREBELLY	NEWT	LIV	300	NO	CN	CN	T	C	C	C	1/21/08	1/21/08	I	AT	SUN PET LTD.
2008160481	CYPY	CYNOPS	PYRRHOGASTER	JAPANESE FIREBELLY	NEWT	LIV	60	NO	HK	HK	T	W	C	C	1/22/08	1/20/08	I	NY	Transship Discounts Ltd.
2008160481	PACB	PACHYTRITON	BREVIPES	TSITOU	NEWT	LIV	60	NO	HK	HK	T	W	C	C	1/22/08	1/20/08	I	NY	Transship Discounts Ltd.
2008160506	CYPY	CYNOPS	PYRRHOGASTER	JAPANESE FIREBELLY	NEWT	LIV	50	NO	HK	HK	T	W	C	C	9/5/07	9/2/07	I	NY	TRANSSHIP DISCOUNTS LIMITED
2008160527	CYOR	CYNOPS	ORIENTALIS	CHINESE DWARF	NEWT	LIV	100	NO	SG	SG	T	C	C	C	7/25/07	7/15/07	I	NY	INTERNATIONAL PET RESOURCES , LLC
2008160556	CYOR	CYNOPS	ORIENTALIS	CHINESE DWARF	NEWT	LIV	100	NO	HK	HK	T	W	C	C	9/17/07	9/15/07	I	NY	ERDA INC.
2008160649	WNEW	PARAMESOTRITON	SPECIES	WARTY	NEWT	LIV	100	NO	HK	HK	T	W	C	C	1/22/08	1/22/08	I	LA	DM EXOTICS
2008160649	PCHI	PARAMESOTRITON	CHINENSIS	CHINESE WARTY	NEWT	LIV	500	NO	HK	HK	T	W	C	C	1/22/08	1/22/08	I	LA	DM EXOTICS
2008160649	PLAB	PACHYTRITON	LABIATUS	UNTERSTEIN'S	NEWT	LIV	300	NO	HK	HK	T	W	C	C	1/22/08	1/22/08	I	LA	DM EXOTICS
2008161116	CYPY	CYNOPS	PYRRHOGASTER	JAPANESE FIREBELLY	NEWT	LIV	50	NO	HK	HK	T	C	C	C	9/11/07	9/9/07	I	NY	ERDA INCORPORATED
2008161121	CYPY	CYNOPS	PYRRHOGASTER	JAPANESE FIREBELLY	NEWT	LIV	70	NO	HK	HK	T	W	C	C	9/11/07	9/9/07	I	NY	ERDA INCORPORATED
2008161363	CYOR	CYNOPS	ORIENTALIS	CHINESE DWARF	NEWT	LIV	300	NO	HK	HK	T	W	C	C	1/23/08	1/23/08	I	CH	AQUATICS INC
2008161420	CYOR	CYNOPS	ORIENTALIS	CHINESE DWARF	NEWT	LIV	200	NO	SG	SG	T	W	C	C	9/19/07	9/2/07	I	NY	INTERNATIONAL PET RESOURCES , LLC
2008161590	CYPY	CYNOPS	PYRRHOGASTER	JAPANESE FIREBELLY	NEWT	LIV	290	NO	HK	HK	T	W	C	C	8/29/07	8/25/07	I	NY	ERDA INCORPORATED
2008161642	CYPY	CYNOPS	PYRRHOGASTER	JAPANESE FIREBELLY	NEWT	LIV	300	NO	HK	HK	T	W	C	C	9/5/07	9/1/07	I	NY	ERDA INCORPORATED
2008161979	CYPY	CYNOPS	PYRRHOGASTER	JAPANESE FIREBELLY	NEWT	LIV	370	NO	HK	HK	T	W	C	C	5/8/07	5/6/07	I	NY	ERDA INCORPORATED
2008162046	TRIH	TRITURUS	HONGKONGENSIS	ALPINE	NEWT	LIV	750	NO	HK	HK	T	C	C	C	5/18/07	5/14/07	I	NY	TRI-COUNTY TROPICALS, INC.
2008163445	CYOR	CYNOPS	ORIENTALIS	CHINESE DWARF	NEWT	LIV	250	NO	HK	HK	T	C	C	C	1/25/08	1/26/08	I	LA	DOLPHIN INTERNATIONAL
2008163445	PACB	PACHYTRITON	BREVIPES	TSITOU	NEWT	LIV	200	NO	HK	HK	T	C	C	C	1/25/08	1/26/08	I	LA	DOLPHIN INTERNATIONAL
2008163657	TRIH	TRITURUS	HONGKONGENSIS	ALPINE	NEWT	LIV	34	NO	HK	HK	T	W	C	C	1/26/08	1/27/08	I	LA	AQUACO, INC.
2008163674	CYOR	CYNOPS	ORIENTALIS	CHINESE DWARF	NEWT	LIV	450	NO	HK	HK	T	C	C	C	1/26/08	1/27/08	I	LA	DOLPHIN INTERNATIONAL
2008164158	CNP?	CYNOPS	SPECIES	FIREBELLY	NEWT	LIV	270	NO	HK	HK	T	W	C	C	3/19/07	3/17/07	I	NY	ERDA INCORPORATED
2008165712	CYPY	CYNOPS	PYRRHOGASTER	JAPANESE FIREBELLY	NEWT	LIV	110	NO	HK	HK	T	W	C	C	1/30/08	1/27/08	I	NY	Transship Discounts Ltd.
2008166946	PLAB	PACHYTRITON	LABIATUS	UNTERSTEIN'S	NEWT	LIV	1,000	NO	CN	CN	T	W	C	C	1/31/08	1/31/08	I	LA	WORLD WIDE TRADING INC.
2008168278	CYOR	CYNOPS	ORIENTALIS	CHINESE DWARF	NEWT	LIV	250	NO	HK	HK	T	C	C	C	2/1/08	2/2/08	I	LA	DOLPHIN INTERNATIONAL
2008168483	CYOR	CYNOPS	ORIENTALIS	CHINESE DWARF	NEWT	LIV	750	NO	HK	HK	T	C	C	C	2/2/08	2/3/08	I	LA	DOLPHIN INTERNATIONAL
2008168495	CYOR	CYNOPS	ORIENTALIS	CHINESE DWARF	NEWT	LIV	757	NO	HK	HK	T	W	C	C	2/2/08	2/3/08	I	LA	AQUA-NAUTIC SPECIALIST
2008168500	TRIH	TRITURUS	HONGKONGENSIS	ALPINE	NEWT	LIV	100	NO	HK	HK	T	W	C	C	2/2/08	2/3/08	I	LA	AQUACO, INC.
2008168532	CNP?	CYNOPS	SPECIES	FIREBELLY	NEWT	LIV	200	NO	CN	CN	T	C	C	C	2/4/08	2/4/08	I	AT	SUN PET LTD.
2008171318	CNP?	CYNOPS	SPECIES	FIREBELLY	NEWT	LIV	150	NO	CN	CN	T	C	C	C	8/31/07	8/26/07	I	NY	FISH MART INCORPORATED
2008171318	CNP?	CYNOPS	SPECIES	FIREBELLY	NEWT	LIV	0	NO	CN	CN	T	C	C	C	8/31/07	8/26/07	I	NY	FISH MART INCORPORATED
2008172591	TYVE	TYLOTOTRITON	VERRUCOSUS	CROCODILE	NEWT	LIV	300	NO	CN	CN	T	W	C	C	2/7/08	2/7/08	I	LA	GOLDEN POND
2008172793	CYPY	CYNOPS	PYRRHOGASTER	JAPANESE FIREBELLY	NEWT	LIV	170	NO	HK	HK	T	W	C	C	2/7/08	2/3/08	I	NY	Transship Discounts Ltd.
2008177198	CYPY	CYNOPS	PYRRHOGASTER	JAPANESE FIREBELLY	NEWT	LIV	150	NO	HK	HK	T	W	C	C	5/8/07	5/6/07	I	NY	TRANSSHIP DISCOUNTS LIMITED
2008177198	PACB	PACHYTRITON	BREVIPES	TSITOU	NEWT	LIV	60	NO	HK	HK	T	W	C	C	5/8/07	5/6/07	I	NY	TRANSSHIP DISCOUNTS LIMITED
2008177354	CYPY	CYNOPS	PYRRHOGASTER	JAPANESE FIREBELLY	NEWT	LIV	70	NO	HK	HK	T	W	C	C	4/30/07	4/28/07	I	NY	ERDA INCORPORATED
2008178862	CYOR	CYNOPS	ORIENTALIS	CHINESE DWARF	NEWT	LIV	300	NO	HK	HK	T	C	C	C	2/17/08	2/17/08	I	LA	DOLPHIN INTERNATIONAL
2008178862	PACB	PACHYTRITON	BREVIPES	TSITOU	NEWT	LIV	200	NO	HK	HK	T	C	C	C	2/17/08	2/17/08	I	LA	DOLPHIN INTERNATIONAL
2008178901	CNP?	CYNOPS	SPECIES	FIREBELLY	NEWT	LIV	300	NO	CN	CN	T	C	C	C	2/18/08	2/18/08	I	AT	SUN PET LTD.
2008181399	CYPY	CYNOPS	PYRRHOGASTER	JAPANESE FIREBELLY	NEWT	LIV	120	NO	HK	HK	T	W	C	C	2/21/08	2/17/08	I	NY	Transship Discounts Ltd.
2008182998	PACB	PACHYTRITON	BREVIPES	TSITOU	NEWT	LIV	100	NO	HK	HK	T	C	C	C	2/23/08	2/23/08	I	LA	DOLPHIN INTERNATIONAL
2008183081	PLAB	PACHYTRITON	LABIATUS	UNTERSTEIN'S	NEWT	LIV	90	NO	HK	HK	T	W	C	C	2/23/08	2/24/08	I	LA	AQUA-NAUTIC SPECIALIST
2008183083	TRIH	TRITURUS	HONGKONGENSIS	ALPINE	NEWT	LIV	250	NO	HK	HK	T	W	C	C	2/23/08	2/24/08	I	LA	AQUACO, INC.
2008183103	CYOR	CYNOPS	ORIENTALIS	CHINESE DWARF	NEWT	LIV	200	NO	HK	HK	T	C	C	C	2/24/08	2/24/08	I	LA	DOLPHIN INTERNATIONAL
2008183103	PACB	PACHYTRITON	BREVIPES	TSITOU	NEWT	LIV	200	NO	HK	HK	T	C	C	C	2/24/08	2/24/08	I	LA	DOLPHIN INTERNATIONAL
2008183317	TVIT	TRITURUS	VITTATUS	BANDED	NEWT	LIV	25	NO	DE	DE	T	C	C	C	9/18/07	9/13/07	I	SF	NAME REMOVED
2008183686	CYPY	CYNOPS	PYRRHOGASTER	JAPANESE FIREBELLY	NEWT	LIV	110	NO	HK	HK	T	W	C	C	2/25/08	2/24/08	I	NY	Transship Discounts Ltd.
2008184577	CYPY	CYNOPS	PYRRHOGASTER	JAPANESE FIREBELLY	NEWT	LIV	625	NO	HK	HK	T	C	C	C	10/22/07	10/20/07	I	NY	ERDA INCORPORATED
2008187381	PACB	PACHYTRITON	BREVIPES	TSITOU	NEWT	LIV	100	NO	HK	HK	T	C	C	C	3/1/08	3/1/08	I	LA	DOLPHIN INTERNATIONAL
2008187454	TRIH	TRITURUS	HONGKONGENSIS	ALPINE	NEWT	LIV	10	NO	HK	HK	T	W	C	C	3/1/08	3/2/08	I	LA	AQUACO, INC.
2008187456	CYOR	CYNOPS	ORIENTALIS	CHINESE DWARF	NEWT	LIV	600	NO	HK	HK	T	W	C	C	3/1/08	3/2/08	I	LA	AQUA-NAUTIC SPECIALIST
2008187460	CYOR	CYNOPS	ORIENTALIS	CHINESE DWARF	NEWT	LIV	100	NO	HK	HK	T	C	C	C	3/1/08	3/2/08	I	LA	DOLPHIN INTERNATIONAL
2008187460	PACB	PACHYTRITON	BREVIPES	TSITOU	NEWT	LIV	300	NO	HK	HK	T	C	C	C	3/1/08	3/2/08	I	LA	DOLPHIN INTERNATIONAL
2008187494	CNP?	CYNOPS	SPECIES	FIREBELLY	NEWT	LIV	300	NO	CN	CN	T	C	C	C	3/3/08	3/3/08	I	AT	SUN PET LTD.
2008188532	CYPY	CYNOPS	PYRRHOGASTER	JAPANESE FIREBELLY	NEWT	LIV	120	NO	HK	HK	T	W	C	C	3/4/08	3/2/08	I	NY	Transship Discounts Ltd.
2008188557	PLET	PLETHODON	SPECIES		SALAMANDER	LIV	150	NO	US	DE	S	W	C	C	3/3/08	3/3/08	I	PT	OREGON STATE UNIVERSITY
2008189409	CYPY	CYNOPS	PYRRHOGASTER	JAPANESE FIREBELLY	NEWT	LIV	375	NO	HK	HK	T	W	C	C	10/9/07	10/6/07	I	NY	ERDA INC

2008190650	CYOR	CYNOPS	ORIENTALIS	CHINESE DWARF	NEWT	LIV	100	NO	SA	SG	T	W	C	C	10/10/07	9/23/07	I	NY	INTERNATIONAL PET RESOURCES , LLC
2008190981	CYOR	CYNOPS	ORIENTALIS	CHINESE DWARF	NEWT	LIV	100	NO	SG	SG	T	W	C	C	12/12/07	11/25/07	I	NY	INTERNATIONAL PET RESOURCES , LLC
2008190998	CYPY	CYNOPS	PYRRHOGASTER	JAPANESE FIREBELLY	NEWT	LIV	75	NO	HK	HK	T	W	C	C	12/12/07	12/8/07	I	NY	ERDA INCORPORATED
2008191072	CYPY	CYNOPS	PYRRHOGASTER	JAPANESE FIREBELLY	NEWT	LIV	50	NO	HK	HK	T	W	C	C	12/6/07	12/1/07	I	NY	ERDA INCORPORATED
2008191134	CYPY	CYNOPS	PYRRHOGASTER	JAPANESE FIREBELLY	NEWT	LIV	400	NO	HK	HK	T	W	C	C	1/3/08	12/29/07	I	NY	ERDA INCORPORATED
2008191381	CYOR	CYNOPS	ORIENTALIS	CHINESE DWARF	NEWT	LIV	860	NO	CN	SG	T	W	C	C	3/8/08	3/9/08	I	LA	AQUA-NAUTIC SPECIALIST
2008191382	CYOR	CYNOPS	ORIENTALIS	CHINESE DWARF	NEWT	LIV	860	NO	CN	HK	T	W	C	C	3/8/08	3/9/08	I	LA	AQUA-NAUTIC SPECIALIST
2008191405	CYOR	CYNOPS	ORIENTALIS	CHINESE DWARF	NEWT	LIV	200	NO	HK	HK	T	C	C	C	3/9/08	3/9/08	I	LA	DOLPHIN INTERNATIONAL
2008191480	CNP?	CYNOPS	SPECIES	FIREBELLY	NEWT	LIV	300	NO	CN	CN	T	C	C	C	3/10/08	3/10/08	I	AT	SUN PET LTD.
2008191736	CYOR	CYNOPS	ORIENTALIS	CHINESE DWARF	NEWT	LIV	100	NO	SG	SG	T	C	C	C	11/20/07	11/4/07	I	NY	INTERNATIONAL PET RESOURCES , LLC
2008192375	CNP?	CYNOPS	SPECIES	FIREBELLY	NEWT	LIV	125	NO	CN	CN	T	C	C	C	9/28/07	9/23/07	I	NY	FISH MART INCORPORATED
2008192375	CNP?	CYNOPS	SPECIES	FIREBELLY	NEWT	LIV	0	NO	CN	CN	T	C	C	C	9/28/07	9/23/07	I	NY	FISH MART INCORPORATED
2008192594	CYOR	CYNOPS	ORIENTALIS	CHINESE DWARF	NEWT	LIV	200	NO	SG	SG	T	C	C	C	8/1/07	7/22/07	I	NY	INTERNATIONAL PET RESOURCES , LLC
2008192730	CYPY	CYNOPS	PYRRHOGASTER	JAPANESE FIREBELLY	NEWT	LIV	200	NO	HK	HK	T	C	C	C	8/1/07	7/28/07	I	NY	ERDA INCORPORATED
2008192848	CYOR	CYNOPS	ORIENTALIS	CHINESE DWARF	NEWT	LIV	100	NO	SG	SG	T	W	C	C	1/3/08	12/16/07	I	NY	INTERNATIONAL PET RESOURCES , LLC
2008192905	CYPY	CYNOPS	PYRRHOGASTER	JAPANESE FIREBELLY	NEWT	LIV	50	NO	HK	HK	T	W	C	C	8/1/07	7/29/07	I	NY	TRANSSHIP DISCOUNTS LIMITED
2008192905	CYPY	CYNOPS	PYRRHOGASTER	JAPANESE FIREBELLY	NEWT	LIV	30	NO	HK	HK	T	W	C	C	8/1/07	7/29/07	I	NY	TRANSSHIP DISCOUNTS LIMITED
2008194447	CYOR	CYNOPS	ORIENTALIS	CHINESE DWARF	NEWT	LIV	410	NO	HK	HK	T	W	C	C	3/13/08	3/13/08	I	MI	TWO AMIGOS IMPORT & EXPORT, INC.
2008194825	CYPY	CYNOPS	PYRRHOGASTER	JAPANESE FIREBELLY	NEWT	LIV	805	NO	HK	HK	T	C	C	C	8/22/07	8/18/07	I	NY	ERDA INCORPORATED
2008194853	CYPY	CYNOPS	PYRRHOGASTER	JAPANESE FIREBELLY	NEWT	LIV	50	NO	HK	HK	T	W	C	C	8/22/07	8/19/07	I	NY	TRANSSHIP DISCOUNTS LIMITED
2008196154	CYOR	CYNOPS	ORIENTALIS	CHINESE DWARF	NEWT	LIV	750	NO	HK	HK	T	C	C	C	3/15/08	3/15/08	I	LA	DOLPHIN INTERNATIONAL
2008196236	CYOR	CYNOPS	ORIENTALIS	CHINESE DWARF	NEWT	LIV	1,565	NO	SG	SG	T	W	C	C	3/15/08	3/16/08	I	LA	AQUA-NAUTIC SPECIALIST
2008196237	TRIH	TRITURUS	HONGKONGENSIS	ALPINE	NEWT	LIV	100	NO	HK	HK	T	W	C	C	3/15/08	3/16/08	I	LA	AQUACO, INC.
2008196248	PACB	PACHYTRITON	BREVIPES	TSITOU	NEWT	LIV	100	NO	HK	HK	T	C	C	C	3/15/08	3/16/08	I	LA	DOLPHIN INTERNATIONAL
2008196248	CYOR	CYNOPS	ORIENTALIS	CHINESE DWARF	NEWT	LIV	900	NO	HK	HK	T	C	C	C	3/15/08	3/16/08	I	LA	DOLPHIN INTERNATIONAL
2008196285	CYOR	CYNOPS	ORIENTALIS	CHINESE DWARF	NEWT	LIV	1,565	NO	CN	CN	T	W	C	C	3/16/08	3/16/08	I	LA	AQUA-NAUTIC SPECIALIST
2008196337	CNP?	CYNOPS	SPECIES	FIREBELLY	NEWT	LIV	300	NO	CN	CN	T	C	C	C	3/17/08	3/17/08	I	AT	SUN PET LTD.
2008196337	PLAB	PACHYTRITON	LABIATUS	UNTERSTEIN'S	NEWT	LIV	100	NO	CN	CN	T	C	C	C	3/17/08	3/17/08	I	AT	SUN PET LTD.
2008196515	CNP?	CYNOPS	SPECIES	FIREBELLY	NEWT	LIV	1	NO	US	CA	P	C	C	C	8/20/07	8/20/07	I	PL	Exemptions 6 and 7(C)
2008198011	CYPY	CYNOPS	PYRRHOGASTER	JAPANESE FIREBELLY	NEWT	LIV	50	NO	HK	HK	T	W	C	C	3/18/08	3/16/08	I	NY	Transship Discounts Ltd.
2008198011	PACB	PACHYTRITON	BREVIPES	TSITOU	NEWT	LIV	120	NO	HK	HK	T	W	C	C	3/18/08	3/16/08	I	NY	Transship Discounts Ltd.
2008198556	TRIH	TRITURUS	HONGKONGENSIS	ALPINE	NEWT	LIV	1,500	NO	HK	HK	T	C	C	C	3/18/08	3/18/08	I	TP	5-D TROPICAL INC.
2008198655	CYPY	CYNOPS	PYRRHOGASTER	JAPANESE FIREBELLY	NEWT	LIV	20	NO	HK	HK	T	W	C	C	3/18/08	3/9/08	I	NY	Transship Discounts Ltd.
2008198711	PLWA	PLEURODELES	WALTL	IBERIAN RIBBED	NEWT	LIV	57	NO	DE	DE	B	C	C	C	3/18/08	3/17/08	I	AT	NAME REMOVED
2008200307	CYPY	CYNOPS	PYRRHOGASTER	JAPANESE FIREBELLY	NEWT	LIV	450	NO	HK	HK	T	W	C	C	11/19/07	11/17/07	I	NY	ERDA INCORPORATED
2008200315	CYOR	CYNOPS	ORIENTALIS	CHINESE DWARF	NEWT	LIV	100	NO	SG	SG	T	C	C	C	8/15/07	7/29/07	I	NW	INTERNATIONAL PET RESOURCES , LLC
2008200346	CYPY	CYNOPS	PYRRHOGASTER	JAPANESE FIREBELLY	NEWT	LIV	100	NO	HK	HK	T	W	C	C	11/28/07	11/24/07	I	NY	ERDA INCORPORATED
2008200357	CYOR	CYNOPS	ORIENTALIS	CHINESE DWARF	NEWT	LIV	100	NO	SG	SG	T	W	C	C	11/28/07	10/28/07	I	NY	INTERNATIONAL PET RESOURCES , LLC
2008201526	PACB	PACHYTRITON	BREVIPES	TSITOU	NEWT	LIV	100	NO	HK	HK	T	C	C	C	3/21/08	3/22/08	I	LA	DOLPHIN INTERNATIONAL
2008201614	CYOR	CYNOPS	ORIENTALIS	CHINESE DWARF	NEWT	LIV	1,250	NO	HK	HK	T	C	C	C	3/22/08	3/23/08	I	LA	DOLPHIN INTERNATIONAL
2008201614	PACB	PACHYTRITON	BREVIPES	TSITOU	NEWT	LIV	100	NO	HK	HK	T	C	C	C	3/22/08	3/23/08	I	LA	DOLPHIN INTERNATIONAL
2008201630	TRIH	TRITURUS	HONGKONGENSIS	ALPINE	NEWT	LIV	100	NO	HK	HK	T	W	C	C	3/22/08	3/23/08	I	LA	AQUACO, INC.
2008201633	CYOR	CYNOPS	ORIENTALIS	CHINESE DWARF	NEWT	LIV	3,580	NO	CN	CN	T	W	C	C	3/22/08	3/23/08	I	LA	AQUA-NAUTIC SPECIALIST
2008201633	PLAB	PACHYTRITON	LABIATUS	UNTERSTEIN'S	NEWT	LIV	100	NO	CN	CN	T	W	C	C	3/22/08	3/23/08	I	LA	AQUA-NAUTIC SPECIALIST
2008201745	CNP?	CYNOPS	SPECIES	FIREBELLY	NEWT	LIV	150	NO	CN	CN	T	W	C	C	11/8/07	11/4/07	I	NY	FISH MART INCORPORATED
2008201745	CNP?	CYNOPS	SPECIES	FIREBELLY	NEWT	LIV	0	NO	CN	CN	T	W	C	C	11/8/07	11/4/07	I	NY	FISH MART INCORPORATED
2008203413	CYPY	CYNOPS	PYRRHOGASTER	JAPANESE FIREBELLY	NEWT	LIV	260	NO	HK	HK	T	W	C	C	3/27/08	3/23/08	I	NY	Transship Discounts Ltd.
2008204006	CYOR	CYNOPS	ORIENTALIS	CHINESE DWARF	NEWT	LIV	5,000	NO	CN	CN	T	W	C	C	3/28/08	3/28/08	I	LA	Z IMPORTS INC.
2008204400	PACB	PACHYTRITON	BREVIPES	TSITOU	NEWT	LIV	100	NO	HK	HK	T	C	C	C	3/29/08	3/29/08	I	LA	DOLPHIN INTERNATIONAL
2008204467	TRIH	TRITURUS	HONGKONGENSIS	ALPINE	NEWT	LIV	350	NO	HK	HK	T	W	C	C	3/29/08	3/30/08	I	LA	AQUACO, INC.
2008204473	CYOR	CYNOPS	ORIENTALIS	CHINESE DWARF	NEWT	LIV	1,200	NO	CN	CN	T	W	C	C	3/29/08	3/30/08	I	LA	AQUA-NAUTIC SPECIALIST
2008204480	CYOR	CYNOPS	ORIENTALIS	CHINESE DWARF	NEWT	LIV	750	NO	HK	HK	T	C	C	C	3/30/08	3/30/08	I	LA	DOLPHIN INTERNATIONAL
2008204480	PACB	PACHYTRITON	BREVIPES	TSITOU	NEWT	LIV	100	NO	HK	HK	T	C	C	C	3/30/08	3/30/08	I	LA	DOLPHIN INTERNATIONAL
2008204528	CNP?	CYNOPS	SPECIES	FIREBELLY	NEWT	LIV	300	NO	CN	CN	T	C	C	C	3/31/08	3/31/08	I	AT	SUN PET LTD.
2008205553	TRIH	TRITURUS	HONGKONGENSIS	ALPINE	NEWT	LIV	1,600	NO	HK	HK	T	C	C	C	4/1/08	4/1/08	I	TP	5-D TROPICAL INC.
2008205553	CYOR	CYNOPS	ORIENTALIS	CHINESE DWARF	NEWT	LIV	300	NO	HK	HK	T	C	C	C	4/1/08	4/1/08	I	TP	5-D TROPICAL INC.
2008205679	BOG?	BOLITOGLOSSA	SPECIES	MUSHROOMTONGUE	SALAMANDER	LIV	24	NO	MX	MX	S	W	C	C	4/1/08	4/1/08	I	SF	Museum of Vertebrate Zoology
2008205679	BOG?	BOLITOGLOSSA	SPECIES	MUSHROOMTONGUE	SALAMANDER	LIV	1	NO	MX	MX	S	W	C	C	4/1/08	4/1/08	I	SF	Museum of Vertebrate Zoology
2008206358	CYPY	CYNOPS	PYRRHOGASTER	JAPANESE FIREBELLY	NEWT	LIV	220	NO	HK	HK	T	W	C	C	4/3/08	3/30/08	I	NY	Transship Discounts Ltd.
2008207488	CYOR	CYNOPS	ORIENTALIS	CHINESE DWARF	NEWT	LIV	500	NO	HK	HK	T	C	C	C	4/4/08	4/5/08	I	LA	DOLPHIN INTERNATIONAL

2008207488	PACB	PACHYTRITON	BREVIPES	TSITOU	NEWT	LIV	100	NO	HK	HK	T	C	C	C	4/4/08	4/5/08	I	LA	DOLPHIN INTERNATIONAL
2008207647	CYOR	CYNOPS	ORIENTALIS	CHINESE DWARF	NEWT	LIV	350	NO	HK	HK	T	C	C	C	4/5/08	4/6/08	I	LA	DOLPHIN INTERNATIONAL
2008207647	PACB	PACHYTRITON	BREVIPES	TSITOU	NEWT	LIV	100	NO	HK	HK	T	C	C	C	4/5/08	4/6/08	I	LA	DOLPHIN INTERNATIONAL
2008207664	TRIH	TRITURUS	HONGKONGENSIS	ALPINE	NEWT	LIV	350	NO	HK	HK	T	W	C	C	4/5/08	4/6/08	I	LA	AQUACO, INC.
2008207669	CYOR	CYNOPS	ORIENTALIS	CHINESE DWARF	NEWT	LIV	900	NO	CN	CN	T	W	C	C	4/5/08	4/6/08	I	LA	AQUA-NAUTIC SPECIALIST
2008208024	CYPY	CYNOPS	PYRRHOGASTER	JAPANESE FIREBELLY	NEWT	LIV	200	NO	HK	HK	T	W	C	C	4/7/08	4/6/08	I	NY	Transship Discounts Ltd.
2008208024	PACB	PACHYTRITON	BREVIPES	TSITOU	NEWT	LIV	65	NO	HK	HK	T	W	C	C	4/7/08	4/6/08	I	NY	Transship Discounts Ltd.
2008209194	CYOR	CYNOPS	ORIENTALIS	CHINESE DWARF	NEWT	LIV	150	NO	HK	HK	T	W	C	C	4/9/08	4/9/08	I	CH	AQUATICS INC
2008210313	CYOR	CYNOPS	ORIENTALIS	CHINESE DWARF	NEWT	LIV	250	NO	HK	HK	T	C	C	C	4/11/08	4/12/08	I	LA	DOLPHIN INTERNATIONAL
2008210487	CYOR	CYNOPS	ORIENTALIS	CHINESE DWARF	NEWT	LIV	1,640	NO	CN	CN	T	W	C	C	4/12/08	4/13/08	I	LA	AQUA-NAUTIC SPECIALIST
2008210498	CYOR	CYNOPS	ORIENTALIS	CHINESE DWARF	NEWT	LIV	800	NO	HK	HK	T	C	C	C	4/13/08	4/13/08	I	LA	DOLPHIN INTERNATIONAL
2008210529	CNP?	CYNOPS	SPECIES	FIREBELLY	NEWT	LIV	300	NO	CN	CN	T	C	C	C	4/14/08	4/14/08	I	AT	SUN PET LTD.
2008210529	PLAB	PACHYTRITON	LABIATUS	UNTERSTEIN'S	NEWT	LIV	100	NO	CN	CN	T	C	C	C	4/14/08	4/14/08	I	AT	SUN PET LTD.
2008213356	CYOR	CYNOPS	ORIENTALIS	CHINESE DWARF	NEWT	LIV	100	NO	HK	HK	T	C	C	C	4/18/08	4/19/08	I	LA	DOLPHIN INTERNATIONAL
2008213356	PACB	PACHYTRITON	BREVIPES	TSITOU	NEWT	LIV	100	NO	HK	HK	T	C	C	C	4/18/08	4/19/08	I	LA	DOLPHIN INTERNATIONAL
2008213657	TRIH	TRITURUS	HONGKONGENSIS	ALPINE	NEWT	LIV	675	NO	HK	HK	T	W	C	C	4/19/08	4/20/08	I	LA	AQUACO, INC.
2008213661	CYOR	CYNOPS	ORIENTALIS	CHINESE DWARF	NEWT	LIV	1,440	NO	CN	CN	T	W	C	C	4/19/08	4/20/08	I	LA	AQUA-NAUTIC SPECIALIST
2008213682	CYOR	CYNOPS	ORIENTALIS	CHINESE DWARF	NEWT	LIV	1,000	NO	HK	HK	T	C	C	C	4/20/08	4/20/08	I	LA	DOLPHIN INTERNATIONAL
2008213682	PACB	PACHYTRITON	BREVIPES	TSITOU	NEWT	LIV	100	NO	HK	HK	T	C	C	C	4/20/08	4/20/08	I	LA	DOLPHIN INTERNATIONAL
2008214397	TRIH	TRITURUS	HONGKONGENSIS	ALPINE	NEWT	LIV	1,600	NO	HK	HK	T	C	C	C	4/22/08	4/22/08	I	TP	5-D TROPICAL INC.
2008216390	CYOR	CYNOPS	ORIENTALIS	CHINESE DWARF	NEWT	LIV	3,000	NO	CN	CN	T	W	C	C	4/25/08	4/25/08	I	LA	Z IMPORTS INC.
2008216467	CYOR	CYNOPS	ORIENTALIS	CHINESE DWARF	NEWT	LIV	350	NO	HK	HK	T	C	C	C	4/25/08	4/26/08	I	LA	DOLPHIN INTERNATIONAL
2008216467	PACB	PACHYTRITON	BREVIPES	TSITOU	NEWT	LIV	100	NO	HK	HK	T	C	C	C	4/25/08	4/26/08	I	LA	DOLPHIN INTERNATIONAL
2008216651	CYOR	CYNOPS	ORIENTALIS	CHINESE DWARF	NEWT	LIV	450	NO	HK	HK	T	C	C	C	4/26/08	4/27/08	I	LA	DOLPHIN INTERNATIONAL
2008216663	TRIH	TRITURUS	HONGKONGENSIS	ALPINE	NEWT	LIV	200	NO	HK	HK	T	W	C	C	4/26/08	4/27/08	I	LA	AQUACO, INC.
2008216665	CYOR	CYNOPS	ORIENTALIS	CHINESE DWARF	NEWT	LIV	5,980	NO	CN	CN	T	W	C	C	4/26/08	4/27/08	I	LA	AQUA-NAUTIC SPECIALIST
2008219226	CYPY	CYNOPS	PYRRHOGASTER	JAPANESE FIREBELLY	NEWT	LIV	100	NO	HK	HK	T	W	C	C	5/2/08	4/27/08	I	NY	Transship Discounts Ltd.
2008219796	TRIH	TRITURUS	HONGKONGENSIS	ALPINE	NEWT	LIV	650	NO	HK	HK	T	W	C	C	5/3/08	5/4/08	I	LA	AQUACO, INC.
2008219809	CNP?	CYNOPS	SPECIES	FIREBELLY	NEWT	LIV	50	NO	SG	SG	T	W	C	C	5/3/08	5/4/08	I	LA	TROPICAL FISH CONSOLIDATORS LTD.
2008219837	CYOR	CYNOPS	ORIENTALIS	CHINESE DWARF	NEWT	LIV	500	NO	HK	HK	T	C	C	C	5/4/08	5/4/08	I	LA	DOLPHIN INTERNATIONAL
2008219850	CYOR	CYNOPS	ORIENTALIS	CHINESE DWARF	NEWT	LIV	1,800	NO	CN	CN	T	W	C	C	5/4/08	5/4/08	I	LA	AQUA-NAUTIC SPECIALIST
2008219861	CNP?	CYNOPS	SPECIES	FIREBELLY	NEWT	LIV	300	NO	CN	CN	T	C	C	C	5/5/08	5/5/08	I	AT	SUN PET LTD.
2008220082	CYOR	CYNOPS	ORIENTALIS	CHINESE DWARF	NEWT	LIV	500	NO	CN	CN	T	W	C	C	5/5/08	5/5/08	I	LA	WORLD WIDE TRADING INC.
2008220082	PLAB	PACHYTRITON	LABIATUS	UNTERSTEIN'S	NEWT	LIV	500	NO	CN	CN	T	W	C	C	5/5/08	5/5/08	I	LA	WORLD WIDE TRADING INC.
2008220331	CYPY	CYNOPS	PYRRHOGASTER	JAPANESE FIREBELLY	NEWT	LIV	150	NO	HK	HK	T	W	C	C	5/6/08	5/4/08	I	NY	Transship Discounts Ltd.
2008222629	CYOR	CYNOPS	ORIENTALIS	CHINESE DWARF	NEWT	LIV	3,000	NO	CN	CN	T	W	C	C	5/9/08	5/9/08	I	LA	Z IMPORTS INC.
2008222776	CYPY	CYNOPS	PYRRHOGASTER	JAPANESE FIREBELLY	NEWT	LIV	250	NO	HK	HK	T	W	C	C	4/24/07	4/22/07	I	NY	TRANSSHIP DISCOUNTS LIMITED
2008222997	PACB	PACHYTRITON	BREVIPES	TSITOU	NEWT	LIV	100	NO	HK	HK	T	C	C	C	5/10/08	5/10/08	I	LA	DOLPHIN INTERNATIONAL
2008223073	CYOR	CYNOPS	ORIENTALIS	CHINESE DWARF	NEWT	LIV	1,040	NO	CN	CN	T	W	C	C	5/10/08	5/11/08	I	LA	AQUA-NAUTIC SPECIALIST
2008223090	CYOR	CYNOPS	ORIENTALIS	CHINESE DWARF	NEWT	LIV	500	NO	HK	HK	T	C	C	C	5/10/08	5/11/08	I	LA	DOLPHIN INTERNATIONAL
2008224493	CYOR	CYNOPS	ORIENTALIS	CHINESE DWARF	NEWT	LIV	100	NO	SG	SG	T	W	C	C	8/29/07	8/19/07	I	NY	INTERNATIONAL PET RESOURCES , LLC
2008224637	CYPY	CYNOPS	PYRRHOGASTER	JAPANESE FIREBELLY	NEWT	LIV	150	NO	HK	HK	T	W	C	C	7/10/07	7/7/07	I	NY	ERDA INCORPORATED
2008224694	TRIH	TRITURUS	HONGKONGENSIS	ALPINE	NEWT	LIV	200	NO	HK	HK	T	W	C	C	5/14/08	5/14/08	I	CH	AQUATICS INC
2008224861	CYPY	CYNOPS	PYRRHOGASTER	JAPANESE FIREBELLY	NEWT	LIV	50	NO	HK	HK	T	W	C	C	10/30/07	10/27/07	I	NY	ERDA INCORPORATED
2008224906	CNP?	CYNOPS	SPECIES	FIREBELLY	NEWT	LIV	150	NO	CN	CN	T	C	C	C	9/14/07	9/9/07	I	NY	FISH MART INCORPORATED
2008224933	CYOR	CYNOPS	ORIENTALIS	CHINESE DWARF	NEWT	LIV	100	NO	SG	SG	T	W	C	C	8/23/07	8/12/07	I	NY	INTERNATIONAL PET RESOURCES , LLC
2008224936	CYOR	CYNOPS	ORIENTALIS	CHINESE DWARF	NEWT	LIV	100	NO	SG	SG	T	W	C	C	8/23/07	8/5/07	I	NY	INTERNATIONAL PET RESOURCES , LLC
2008225027	CYPY	CYNOPS	PYRRHOGASTER	JAPANESE FIREBELLY	NEWT	LIV	150	NO	HK	HK	T	W	C	C	5/14/08	5/11/08	I	NY	Transship Discounts Ltd.
2008225027	PACB	PACHYTRITON	BREVIPES	TSITOU	NEWT	LIV	60	NO	HK	HK	T	W	C	C	5/14/08	5/11/08	I	NY	Transship Discounts Ltd.
2008225987	CYOR	CYNOPS	ORIENTALIS	CHINESE DWARF	NEWT	LIV	100	NO	SG	SG	T	W	C	C	10/1/07	9/9/07	I	NY	INTERNATIONAL PET RESOURCES , LLC
2008225994	CYOR	CYNOPS	ORIENTALIS	CHINESE DWARF	NEWT	LIV	100	NO	SG	SG	T	W	C	C	9/28/07	9/16/07	I	NY	INTERNATIONAL PET RESOURCES , LLC
2008226114	CYOR	CYNOPS	ORIENTALIS	CHINESE DWARF	NEWT	LIV	250	NO	HK	HK	T	C	C	C	5/16/08	5/17/08	I	LA	DOLPHIN INTERNATIONAL
2008226266	CYOR	CYNOPS	ORIENTALIS	CHINESE DWARF	NEWT	LIV	2,400	NO	CN	CN	T	W	C	C	5/17/08	5/18/08	I	LA	AQUA-NAUTIC SPECIALIST
2008226294	CYOR	CYNOPS	ORIENTALIS	CHINESE DWARF	NEWT	LIV	950	NO	HK	HK	T	C	C	C	5/18/08	5/18/08	I	LA	DOLPHIN INTERNATIONAL
2008226407	CNP?	CYNOPS	SPECIES	FIREBELLY	NEWT	LIV	300	NO	CN	CN	T	C	C	C	5/19/08	5/19/08	I	AT	SUN PET LTD.
2008226959	CYPY	CYNOPS	PYRRHOGASTER	JAPANESE FIREBELLY	NEWT	LIV	200	NO	HK	HK	T	W	C	C	5/19/08	5/18/08	I	NY	Transship Discounts Ltd.
2008227806	TRIH	TRITURUS	HONGKONGENSIS	ALPINE	NEWT	LIV	1,600	NO	HK	HK	T	C	C	C	5/20/08	5/20/08	I	TP	5-D TROPICAL INC.
2008229364	CYOR	CYNOPS	ORIENTALIS	CHINESE DWARF	NEWT	LIV	1,000	NO	CN	CN	T	W	C	C	5/22/08	5/22/08	I	LA	WORLD WIDE TRADING INC.
2008229364	PLAB	PACHYTRITON	LABIATUS	UNTERSTEIN'S	NEWT	LIV	1,000	NO	CN	CN	T	W	C	C	5/22/08	5/22/08	I	LA	WORLD WIDE TRADING INC.
2008229962	PACB	PACHYTRITON	BREVIPES	TSITOU	NEWT	LIV	100	NO	HK	HK	T	C	C	C	5/23/08	5/24/08	I	LA	DOLPHIN INTERNATIONAL

2008230119	CYOR	CYNOPS	ORIENTALIS	CHINESE DWARF	NEWT	LIV	2,320	NO	CN	CN	T	W	C	C	5/24/08	5/25/08	I	LA	AQUA-NAUTIC SPECIALIST
2008230121	TRIH	TRITURUS	HONGKONGENSIS	ALPINE	NEWT	LIV	300	NO	HK	HK	T	W	C	C	5/24/08	5/25/08	I	LA	AQUACO, INC.
2008230136	CYOR	CYNOPS	ORIENTALIS	CHINESE DWARF	NEWT	LIV	1,200	NO	HK	HK	T	C	C	C	5/24/08	5/25/08	I	LA	DOLPHIN INTERNATIONAL
2008230163	PLAB	PACHYTRITON	LABIATUS	UNTERSTEIN'S	NEWT	LIV	100	NO	CN	CN	T	C	C	C	5/26/08	5/26/08	I	AT	SUN PET LTD.
2008232827	CYPY	CYNOPS	PYRRHOGASTER	JAPANESE FIREBELLY	NEWT	LIV	75	NO	HK	HK	T	W	C	C	11/5/07	11/3/07	I	NY	ERDA INCORPORATED
2008233393	CYOR	CYNOPS	ORIENTALIS	CHINESE DWARF	NEWT	LIV	250	NO	HK	HK	T	C	C	C	5/30/08	5/31/08	I	LA	DOLPHIN INTERNATIONAL
2008233569	TRIH	TRITURUS	HONGKONGENSIS	ALPINE	NEWT	LIV	200	NO	HK	HK	T	W	C	C	6/1/08	6/1/08	I	LA	AQUACO, INC.
2008233572	CYOR	CYNOPS	ORIENTALIS	CHINESE DWARF	NEWT	LIV	600	NO	CN	CN	T	W	C	C	6/1/08	6/1/08	I	LA	AQUA-NAUTIC SPECIALIST
2008233591	PACB	PACHYTRITON	BREVIPES	TSITOU	NEWT	LIV	100	NO	HK	HK	T	C	C	C	6/1/08	6/1/08	I	LA	DOLPHIN INTERNATIONAL
2008233606	CNP?	CYNOPS	SPECIES	FIREBELLY	NEWT	LIV	300	NO	CN	CN	T	C	C	C	6/2/08	6/2/08	I	AT	SUN PET LTD.
2008233704	CNP?	CYNOPS	SPECIES	FIREBELLY	NEWT	LIV	150	NO	CN	CN	T	W	C	C	10/26/07	10/21/07	I	NY	FISH MART INCORPORATED
2008233880	CYPY	CYNOPS	PYRRHOGASTER	JAPANESE FIREBELLY	NEWT	LIV	315	NO	HK	HK	T	W	C	C	6/2/08	6/1/08	I	NY	Transship Discounts Ltd.
2008234663	TRIH	TRITURUS	HONGKONGENSIS	ALPINE	NEWT	LIV	1,600	NO	HK	HK	T	C	C	C	6/3/08	6/3/08	I	TP	5-D TROPICAL INC.
2008236768	CYOR	CYNOPS	ORIENTALIS	CHINESE DWARF	NEWT	LIV	1,640	NO	CN	CN	T	W	C	C	6/7/08	6/8/08	I	LA	AQUA-NAUTIC SPECIALIST
2008236793	CYOR	CYNOPS	ORIENTALIS	CHINESE DWARF	NEWT	LIV	2,600	NO	HK	HK	T	C	C	C	6/8/08	6/8/08	I	LA	DOLPHIN INTERNATIONAL
2008236835	CNP?	CYNOPS	SPECIES	FIREBELLY	NEWT	LIV	300	NO	CN	CN	T	C	C	C	6/9/08	6/9/08	I	AT	SUN PET LTD.
2008238312	CYPY	CYNOPS	PYRRHOGASTER	JAPANESE FIREBELLY	NEWT	LIV	210	NO	HK	HK	T	W	C	C	6/11/08	6/8/08	I	NY	Transship Discounts Ltd.
2008238830	CYOR	CYNOPS	ORIENTALIS	CHINESE DWARF	NEWT	LIV	1,000	NO	CN	CN	T	W	C	C	6/12/08	6/12/08	I	LA	WORLD WIDE TRADING INC.
2008239611	CYOR	CYNOPS	ORIENTALIS	CHINESE DWARF	NEWT	LIV	500	NO	HK	HK	T	C	C	C	6/14/08	6/14/08	I	LA	DOLPHIN INTERNATIONAL
2008239611	PACB	PACHYTRITON	BREVIPES	TSITOU	NEWT	LIV	100	NO	HK	HK	T	C	C	C	6/14/08	6/14/08	I	LA	DOLPHIN INTERNATIONAL
2008239696	TRIH	TRITURUS	HONGKONGENSIS	ALPINE	NEWT	LIV	200	NO	HK	HK	T	W	C	C	6/14/08	6/15/08	I	LA	AQUACO, INC.
2008239706	CYOR	CYNOPS	ORIENTALIS	CHINESE DWARF	NEWT	LIV	580	NO	CN	CN	T	W	C	C	6/14/08	6/15/08	I	LA	AQUA-NAUTIC SPECIALIST
2008239712	CYOR	CYNOPS	ORIENTALIS	CHINESE DWARF	NEWT	LIV	600	NO	HK	HK	T	C	C	C	6/14/08	6/15/08	I	LA	DOLPHIN INTERNATIONAL
2008239712	PACB	PACHYTRITON	BREVIPES	TSITOU	NEWT	LIV	100	NO	HK	HK	T	C	C	C	6/14/08	6/15/08	I	LA	DOLPHIN INTERNATIONAL
2008240458	TRIH	TRITURUS	HONGKONGENSIS	ALPINE	NEWT	LIV	1,600	NO	HK	HK	T	C	C	C	6/17/08	6/17/08	I	TP	5-D TROPICAL INC.
2008240998	CYOR	CYNOPS	ORIENTALIS	CHINESE DWARF	NEWT	LIV	250	NO	HK	HK	T	W	C	C	6/18/08	6/18/08	I	CH	AQUATICS INC
2008241665	PACB	PACHYTRITON	BREVIPES	TSITOU	NEWT	LIV	60	NO	HK	HK	T	W	C	C	6/19/08	6/15/08	I	NY	Transship Discounts Ltd.
2008242200	CYOR	CYNOPS	ORIENTALIS	CHINESE DWARF	NEWT	LIV	2,000	NO	CN	CN	T	W	C	C	6/20/08	6/20/08	I	LA	Z IMPORTS INC.
2008242624	CYOR	CYNOPS	ORIENTALIS	CHINESE DWARF	NEWT	LIV	1,200	NO	HK	HK	T	C	C	C	6/21/08	6/22/08	I	LA	DOLPHIN INTERNATIONAL
2008243775	TRIH	TRITURUS	HONGKONGENSIS	ALPINE	NEWT	LIV	1,600	NO	HK	HK	T	C	C	C	6/24/08	6/24/08	I	TP	5-D TROPICAL INC.
2008244280	CYPY	CYNOPS	PYRRHOGASTER	JAPANESE FIREBELLY	NEWT	LIV	105	NO	HK	HK	T	W	C	C	6/25/08	6/22/08	I	NY	Transship Discounts Ltd.
2008245608	CYOR	CYNOPS	ORIENTALIS	CHINESE DWARF	NEWT	LIV	500	NO	HK	HK	T	C	C	C	6/28/08	6/28/08	I	LA	DOLPHIN INTERNATIONAL
2008245639	CYOR	CYNOPS	ORIENTALIS	CHINESE DWARF	NEWT	LIV	1,000	NO	HK	HK	T	C	C	C	6/29/08	6/29/08	I	LA	DOLPHIN INTERNATIONAL
2008245660	CYPY	CYNOPS	PYRRHOGASTER	JAPANESE FIREBELLY	NEWT	LIV	300	NO	CN	CN	T	C	C	C	6/30/08	6/30/08	I	AT	SUN PET LTD.
2008247288	TRIH	TRITURUS	HONGKONGENSIS	ALPINE	NEWT	LIV	1,600	NO	HK	HK	T	C	C	C	7/1/08	7/1/08	I	TP	5-D TROPICAL INC.
2008247627	CYPY	CYNOPS	PYRRHOGASTER	JAPANESE FIREBELLY	NEWT	LIV	400	NO	CN	CN	T	C	C	C	8/21/07	8/12/07	I	NY	FISH MART INCORPORATED
2008248760	CYOR	CYNOPS	ORIENTALIS	CHINESE DWARF	NEWT	LIV	100	NO	SG	SG	T	W	C	C	10/29/07	9/23/07	I	NY	INTERNATIONAL PET RESOURCES , LLC
2008249327	CNP?	CYNOPS	SPECIES	FIREBELLY	NEWT	LIV	200	NO	CN	CN	T	W	C	C	12/2/07	12/2/07	I	NY	FISH MART INCORPORATED
2008249672	TRIH	TRITURUS	HONGKONGENSIS	ALPINE	NEWT	LIV	1,200	NO	HK	HK	T	W	C	C	7/5/08	7/6/08	I	LA	AQUACO, INC.
2008249681	CYOR	CYNOPS	ORIENTALIS	CHINESE DWARF	NEWT	LIV	1,200	NO	CN	CN	T	W	C	C	7/5/08	7/6/08	I	LA	AQUA-NAUTIC SPECIALIST
2008249689	TRIH	TRITURUS	HONGKONGENSIS	ALPINE	NEWT	LIV	1,200	NO	HK	HK	T	W	C	C	7/5/08	7/6/08	I	LA	AQUACO, INC.
2008249719	CYOR	CYNOPS	ORIENTALIS	CHINESE DWARF	NEWT	LIV	1,200	NO	HK	HK	T	C	C	C	7/6/08	7/6/08	I	LA	DOLPHIN INTERNATIONAL
2008249773	TRIT	TRITURUS	SPECIES		NEWT	LIV	5	NO	GE	DE	T	C	C	C	4/1/08	3/26/08	I	SF	Exemptions 6 and 7(C)
2008249773	TRIT	TRITURUS	SPECIES		NEWT	LIV	6	NO	GE	DE	T	C	C	C	4/1/08	3/26/08	I	SF	Exemptions 6 and 7(C)
2008249773	SASA	SALAMANDRA	SALAMANDRA	EUROPEAN FIRE	SALAMANDER	LIV	16	NO	GE	DE	T	C	C	C	4/1/08	3/26/08	I	SF	Exemptions 6 and 7(C)
2008249773	SAL?	SALAMANDRA	SPECIES	FIRE	SALAMANDER	LIV	6	NO	GE	DE	T	C	C	C	4/1/08	3/26/08	I	SF	Exemptions 6 and 7(C)
2008249773	NRR?	NEURERGUS	SPECIES		NEWT	LIV	5	NO	GE	DE	T	C	C	C	4/1/08	3/26/08	I	SF	Exemptions 6 and 7(C)
2008249773	TRIT	TRITURUS	SPECIES		NEWT	LIV	5	NO	DE	DE	T	C	C	C	4/1/08	3/26/08	I	SF	Exemptions 6 and 7(C)
2008249773	SASA	SALAMANDRA	SALAMANDRA	EUROPEAN FIRE	SALAMANDER	LIV	25	NO	GE	DE	T	C	C	C	4/1/08	3/26/08	I	SF	Exemptions 6 and 7(C)
2008249773	TRIT	TRITURUS	SPECIES		NEWT	LIV	2	NO	GE	DE	T	C	C	C	4/1/08	3/26/08	I	SF	Exemptions 6 and 7(C)
2008249917	CYOR	CYNOPS	ORIENTALIS	CHINESE DWARF	NEWT	LIV	100	NO	SG	SG	T	W	C	C	12/7/07	11/11/07	I	NY	INTERNATIONAL PET RESOURCES , LLC
2008251673	CYPY	CYNOPS	PYRRHOGASTER	JAPANESE FIREBELLY	NEWT	LIV	315	NO	HK	HK	T	W	C	C	7/9/08	7/6/08	I	NY	Transship Discounts Ltd.
2008253418	CYOR	CYNOPS	ORIENTALIS	CHINESE DWARF	NEWT	LIV	250	NO	HK	HK	T	C	C	C	7/11/08	7/12/08	I	LA	DOLPHIN INTERNATIONAL
2008253558	PACB	PACHYTRITON	BREVIPES	TSITOU	NEWT	LIV	100	NO	HK	HK	T	C	C	C	7/12/08	7/13/08	I	LA	DOLPHIN INTERNATIONAL
2008253568	CYOR	CYNOPS	ORIENTALIS	CHINESE DWARF	NEWT	LIV	740	NO	CN	CN	T	W	C	C	7/12/08	7/13/08	I	LA	AQUA-NAUTIC SPECIALIST
2008253619	CNP?	CYNOPS	SPECIES	FIREBELLY	NEWT	LIV	300	NO	CN	CN	T	C	C	C	7/14/08	7/14/08	I	AT	SUN PET LTD.
2008256086	CYOR	CYNOPS	ORIENTALIS	CHINESE DWARF	NEWT	LIV	2,000	NO	CN	CN	T	W	C	C	7/18/08	7/18/08	I	LA	Z IMPORTS INC.
2008256530	TRIH	TRITURUS	HONGKONGENSIS	ALPINE	NEWT	LIV	100	NO	SD	SG	T	W	C	C	7/19/08	7/20/08	I	LA	AQUACO, INC.
2008256543	PACB	PACHYTRITON	BREVIPES	TSITOU	NEWT	LIV	100	NO	HK	HK	T	C	C	C	7/19/08	7/20/08	I	LA	DOLPHIN INTERNATIONAL
2008256543	CYOR	CYNOPS	ORIENTALIS	CHINESE DWARF	NEWT	LIV	1,000	NO	HK	HK	T	C	C	C	7/19/08	7/20/08	I	LA	DOLPHIN INTERNATIONAL

2008256553	CYPY	CYNOPS	PYRRHOGASTER	JAPANESE FIREBELLY	NEWT	LIV	100	NO	SG	SG	T	W	C	C	7/19/08	7/20/08	I	LA	TROPICAL FISH CONSOLIDATORS LTD.
2008256575	TRIH	TRITURUS	HONGKONGENSIS	ALPINE	NEWT	LIV	150	NO	HK	HK	T	W	C	C	7/20/08	7/20/08	I	LA	AQUACO, INC.
2008256578	PLAB	PACHYTRITON	LABIATUS	UNTERSTEIN'S	NEWT	LIV	100	NO	CN	CN	T	C	C	C	7/21/08	7/21/08	I	AT	SUN PET LTD.
2008257634	TRIH	TRITURUS	HONGKONGENSIS	ALPINE	NEWT	LIV	1,600	NO	HK	HK	T	C	C	C	7/22/08	7/22/08	I	TP	5-D TROPICAL INC.
2008258067	CYPY	CYNOPS	PYRRHOGASTER	JAPANESE FIREBELLY	NEWT	LIV	105	NO	HK	HK	T	W	C	C	7/23/08	7/20/08	I	NY	Transship Discounts Ltd.
2008258255	CYOR	CYNOPS	ORIENTALIS	CHINESE DWARF	NEWT	LIV	400	NO	HK	HK	T	W	C	C	7/23/08	7/23/08	I	CH	AQUATICS INC
2008259532	CYOR	CYNOPS	ORIENTALIS	CHINESE DWARF	NEWT	LIV	250	NO	HK	HK	T	C	C	C	7/25/08	7/26/08	I	LA	DOLPHIN INTERNATIONAL
2008259681	CYPY	CYNOPS	PYRRHOGASTER	JAPANESE FIREBELLY	NEWT	LIV	140	NO	SG	SG	T	W	C	C	7/26/08	7/27/08	I	LA	TROPICAL FISH CONSOLIDATORS LTD.
2008259693	TRIH	TRITURUS	HONGKONGENSIS	ALPINE	NEWT	LIV	189	NO	HK	HK	T	W	C	C	7/26/08	7/27/08	I	LA	AQUACO, INC.
2008259694	CYOR	CYNOPS	ORIENTALIS	CHINESE DWARF	NEWT	LIV	1,850	NO	HK	HK	T	C	C	C	7/26/08	7/27/08	I	LA	DOLPHIN INTERNATIONAL
2008259746	CNP?	CYNOPS	SPECIES	FIREBELLY	NEWT	LIV	300	NO	CN	CN	T	C	C	C	7/28/08	7/28/08	I	AT	SUN PET LTD.
2008265507	CYOR	CYNOPS	ORIENTALIS	CHINESE DWARF	NEWT	LIV	500	NO	HK	HK	T	C	C	C	8/3/08	8/3/08	I	LA	DOLPHIN INTERNATIONAL
2008265507	PACB	PACHYTRITON	BREVIPES	TSITOU	NEWT	LIV	100	NO	HK	HK	T	C	C	C	8/3/08	8/3/08	I	LA	DOLPHIN INTERNATIONAL
2008265526	CYOR	CYNOPS	ORIENTALIS	CHINESE DWARF	NEWT	LIV	440	NO	CN	CN	T	W	C	C	8/3/08	8/3/08	I	LA	AQUA-NAUTIC SPECIALIST
2008265765	CYOR	CYNOPS	ORIENTALIS	CHINESE DWARF	NEWT	LIV	100	NO	SG	SG	T	C	C	C	2/6/08	1/20/08	I	NY	INTERNATIONAL PET RESOURCES , LLC
2008267295	CYPY	CYNOPS	PYRRHOGASTER	JAPANESE FIREBELLY	NEWT	LIV	150	NO	HK	HK	T	C	C	C	9/30/07	9/29/07	I	NY	ERDA INCORPORATED
2008267770	TRIH	TRITURUS	HONGKONGENSIS	ALPINE	NEWT	LIV	1,600	NO	HK	HK	T	C	C	C	8/5/08	8/5/08	I	TP	5-D TROPICAL INC.
2008268632	CNP?	CYNOPS	SPECIES	FIREBELLY	NEWT	LIV	50	NO	HK	HK	T	W	C	C	12/20/07	12/15/07	I	NY	ERDA INCORPORATED
2008269033	CYPY	CYNOPS	PYRRHOGASTER	JAPANESE FIREBELLY	NEWT	LIV	250	NO	HK	HK	T	C	C	C	9/22/07	9/22/07	I	NY	ERDA INCORPORATED
2008270840	PACB	PACHYTRITON	BREVIPES	TSITOU	NEWT	LIV	100	NO	HK	HK	T	C	C	C	8/8/08	8/9/08	I	LA	DOLPHIN INTERNATIONAL
2008270986	CYOR	CYNOPS	ORIENTALIS	CHINESE DWARF	NEWT	LIV	1,000	NO	HK	HK	T	C	C	C	8/9/08	8/10/08	I	LA	DOLPHIN INTERNATIONAL
2008270986	PACB	PACHYTRITON	BREVIPES	TSITOU	NEWT	LIV	100	NO	HK	HK	T	C	C	C	8/9/08	8/10/08	I	LA	DOLPHIN INTERNATIONAL
2008271006	TRIH	TRITURUS	HONGKONGENSIS	ALPINE	NEWT	LIV	350	NO	HK	HK	T	W	C	C	8/10/08	8/10/08	I	LA	AQUACO INC.
2008271592	CNP?	CYNOPS	SPECIES	FIREBELLY	NEWT	LIV	300	NO	CN	CN	T	C	C	C	8/11/08	8/11/08	I	AT	SUN PET LTD.
2008273886	SASA	SALAMANDRA	SALAMANDRA	EUROPEAN FIRE	SALAMANDER	LIV	21	NO	DE	DE	P	C	C	C	8/14/08	8/14/08	I	DF	Exemptions 6 and 7(C)
2008273886	SASA	SALAMANDRA	SALAMANDRA	EUROPEAN FIRE	SALAMANDER	LIV	5	NO	DE	DE	P	C	C	C	8/14/08	8/14/08	I	DF	Exemptions 6 and 7(C)
2008273886	SASA	SALAMANDRA	SALAMANDRA	EUROPEAN FIRE	SALAMANDER	LIV	2	NO	DE	DE	P	C	C	C	8/14/08	8/14/08	I	DF	Exemptions 6 and 7(C)
2008273886	SAL?	SALAMANDRA	SPECIES	FIRE	SALAMANDER	LIV	2	NO	DE	DE	P	C	C	C	8/14/08	8/14/08	I	DF	Exemptions 6 and 7(C)
2008274473	CYOR	CYNOPS	ORIENTALIS	CHINESE DWARF	NEWT	LIV	750	NO	HK	HK	T	C	C	C	8/15/08	8/16/08	I	LA	DOLPHIN INTERNATIONAL
2008274584	TRIH	TRITURUS	HONGKONGENSIS	ALPINE	NEWT	LIV	100	NO	HK	HK	T	C	C	C	8/16/08	8/17/08	I	LA	AQUACO, INC.
2008274590	CYOR	CYNOPS	ORIENTALIS	CHINESE DWARF	NEWT	LIV	740	NO	CN	CN	T	W	C	C	8/16/08	8/17/08	I	LA	AQUA-NAUTIC SPECIALIST
2008274600	CYOR	CYNOPS	ORIENTALIS	CHINESE DWARF	NEWT	LIV	1,500	NO	HK	HK	T	C	C	C	8/16/08	8/17/08	I	LA	DOLPHIN INTERNATIONAL
2008274600	PACB	PACHYTRITON	BREVIPES	TSITOU	NEWT	LIV	300	NO	HK	HK	T	C	C	C	8/16/08	8/17/08	I	LA	DOLPHIN INTERNATIONAL
2008276648	SASA	SALAMANDRA	SALAMANDRA	EUROPEAN FIRE	SALAMANDER	LIV	120	NO	DE	DE	T	C	C	C	8/21/08	8/21/08	I	DF	U.S. GLOBAL EXOTICS, INC.
2008276648	TRIT	TRITURUS	SPECIES		NEWT	LIV	70	NO	DE	DE	T	C	C	C	8/21/08	8/21/08	I	DF	U.S. GLOBAL EXOTICS, INC.
2008276648	CYPY	CYNOPS	PYRRHOGASTER	JAPANESE FIREBELLY	NEWT	LIV	200	NO	DE	DE	T	C	C	C	8/21/08	8/21/08	I	DF	U.S. GLOBAL EXOTICS, INC.
2008276648	CNP?	CYNOPS	SPECIES	FIREBELLY	NEWT	LIV	8	NO	DE	DE	T	C	C	C	8/21/08	8/21/08	I	DF	U.S. GLOBAL EXOTICS, INC.
2008276792	TRIH	TRITURUS	HONGKONGENSIS	ALPINE	NEWT	LIV	3,200	NO	HK	HK	T	C	C	C	8/22/08	8/19/08	I	TP	5-D TROPICAL INC.
2008276904	CYOR	CYNOPS	ORIENTALIS	CHINESE DWARF	NEWT	LIV	2,000	NO	CN	CN	T	W	C	C	8/22/08	8/22/08	I	LA	Z IMPORTS INC.
2008277191	TYKW	TYLOTOTRITON	KWEICHOWENSIS	KWEICHOW CROCODILE	NEWT	LIV	10	NO	CA	CA	T	C	C	C	8/22/08	8/22/08	I	NY	Exemptions 6 and 7(C)
2008277191	TYL?	TYLOTOTRITON	SPECIES	CROCODILE	NEWT	LIV	6	NO	CA	CA	T	C	C	C	8/22/08	8/22/08	I	NY	Exemptions 6 and 7(C)
2008277191	TYL?	TYLOTOTRITON	SPECIES	CROCODILE	NEWT	LIV	4	NO	CA	CA	T	C	C	C	8/22/08	8/22/08	I	NY	Exemptions 6 and 7(C)
2008277191	CNP?	CYNOPS	SPECIES	FIREBELLY	NEWT	LIV	5	NO	CA	CA	T	C	C	C	8/22/08	8/22/08	I	NY	Exemptions 6 and 7(C)
2008277191	PCAU	PARAMESOTRITON	CAUDOPUNCTATUS	GUIZHOU WARTY	NEWT	LIV	16	NO	CA	CA	T	C	C	C	8/22/08	8/22/08	I	NY	Exemptions 6 and 7(C)
2008277191	PLAB	PACHYTRITON	LABIATUS	UNTERSTEIN'S	NEWT	LIV	2	NO	CA	CA	T	C	C	C	8/22/08	8/22/08	I	NY	Exemptions 6 and 7(C)
2008277191	HYN?	HYNOBIUS	SPECIES		SALAMANDER	LIV	2	NO	CA	CA	T	C	C	C	8/22/08	8/22/08	I	NY	Exemptions 6 and 7(C)
2008277191	HYN?	HYNOBIUS	SPECIES		SALAMANDER	LIV	10	NO	CA	CA	T	C	C	C	8/22/08	8/22/08	I	NY	Exemptions 6 and 7(C)
2008277191	HYN?	HYNOBIUS	SPECIES		SALAMANDER	LIV	3	NO	CA	CA	T	C	C	C	8/22/08	8/22/08	I	NY	Exemptions 6 and 7(C)
2008277191	HYN?	HYNOBIUS	SPECIES		SALAMANDER	LIV	5	NO	CA	CA	T	C	C	C	8/22/08	8/22/08	I	NY	Exemptions 6 and 7(C)
2008277191	HYN?	HYNOBIUS	SPECIES		SALAMANDER	LIV	6	NO	CA	CA	T	C	C	C	8/22/08	8/22/08	I	NY	Exemptions 6 and 7(C)
2008277191	HYN?	HYNOBIUS	SPECIES		SALAMANDER	LIV	8	NO	CA	CA	T	C	C	C	8/22/08	8/22/08	I	NY	Exemptions 6 and 7(C)
2008277191	HYN?	HYNOBIUS	SPECIES		SALAMANDER	LIV	1	NO	CA	CA	T	C	C	C	8/22/08	8/22/08	I	NY	Exemptions 6 and 7(C)
2008277191	ONJA	ONYCHODACTYLUS	JAPONICUS	JAPANESE CLAWED	SALAMANDER	LIV	1	NO	CA	CA	T	C	C	C	8/22/08	8/22/08	I	NY	Exemptions 6 and 7(C)
2008277366	TRIH	TRITURUS	HONGKONGENSIS	ALPINE	NEWT	LIV	50	NO	HK	HK	T	W	C	C	8/23/08	8/24/08	I	LA	AQUACO, INC.
2008277380	CYPY	CYNOPS	PYRRHOGASTER	JAPANESE FIREBELLY	NEWT	LIV	50	NO	SG	SG	T	W	C	C	8/23/08	8/24/08	I	LA	TROPICAL FISH CONSOLIDATORS LTD.
2008277384	CYOR	CYNOPS	ORIENTALIS	CHINESE DWARF	NEWT	LIV	1,500	NO	HK	HK	T	C	C	C	8/23/08	8/24/08	I	LA	DOLPHIN INTERNATIONAL
2008277949	CNP?	CYNOPS	SPECIES	FIREBELLY	NEWT	LIV	300	NO	CN	CN	T	C	C	C	8/26/08	8/26/08	I	AT	SUN PET LTD.
2008278737	CYPY	CYNOPS	PYRRHOGASTER	JAPANESE FIREBELLY	NEWT	LIV	545	NO	HK	HK	T	W	C	C	8/27/08	8/24/08	I	NY	Transship Discounts Ltd.
2008278737	PACB	PACHYTRITON	BREVIPES	TSITOU	NEWT	LIV	120	NO	HK	HK	T	W	C	C	8/27/08	8/24/08	I	NY	Transship Discounts Ltd.
2008278909	CYOR	CYNOPS	ORIENTALIS	CHINESE DWARF	NEWT	LIV	200	NO	HK	HK	T	C	C	C	8/27/08	8/27/08	I	CH	AQUATICS INC

2008279141	SASA	SALAMANDRA	SALAMANDRA	EUROPEAN FIRE	SALAMANDER	LIV	1	NO	FR	FR	T	C	C	C	8/28/08	8/20/08	I	MI	TWO AMIGOS IMPORT & EXPORT , INC.
2008279632	CNP?	CYNOPS	SPECIES	FIREBELLY	NEWT	LIV	300	NO	CN	CN	T	W	C	C	1/23/08	1/13/08	I	NY	FISH MART INCORPORATED
2008279786	CYOR	CYNOPS	ORIENTALIS	CHINESE DWARF	NEWT	LIV	2,000	NO	CN	CN	T	W	C	C	8/29/08	8/29/08	I	LA	Z IMPORTS INC.
2008280176	PACB	PACHYTRITON	BREVIPES	TSITOU	NEWT	LIV	100	NO	HK	HK	T	C	C	C	8/30/08	8/30/08	I	LA	DOLPHIN INTERNATIONAL
2008280258	TRIH	TRITURUS	HONGKONGENSIS	ALPINE	NEWT	LIV	150	NO	HK	HK	T	W	C	C	8/30/08	8/31/08	I	LA	AQUACO, INC.
2008280276	CYOR	CYNOPS	ORIENTALIS	CHINESE DWARF	NEWT	LIV	250	NO	HK	HK	T	C	C	C	8/31/08	8/31/08	I	LA	DOLPHIN INTERNATIONAL
2008280276	PACB	PACHYTRITON	BREVIPES	TSITOU	NEWT	LIV	100	NO	HK	HK	T	C	C	C	8/31/08	8/31/08	I	LA	DOLPHIN INTERNATIONAL
2008280385	TRIH	TRITURUS	HONGKONGENSIS	ALPINE	NEWT	LIV	1,400	NO	HK	HK	T	C	C	C	9/2/08	9/2/08	I	TP	5-D TROPICAL INC.
2008280536	PLAB	PACHYTRITON	LABIATUS	UNTERSTEIN'S	NEWT	LIV	500	NO	CN	CN	T	W	C	C	9/2/08	9/2/08	I	LA	NAME REMOVED
2008281141	CYOR	CYNOPS	ORIENTALIS	CHINESE DWARF	NEWT	LIV	100	NO	HK	HK	T	C	C	C	2/13/08	2/9/08	I	NY	ERDA INCORPORATED
2008281184	CYPY	CYNOPS	PYRRHOGASTER	JAPANESE FIREBELLY	NEWT	LIV	100	NO	HK	HK	T	C	C	C	2/7/08	2/2/08	I	NY	ERDA INCORPORATED
2008283287	CYOR	CYNOPS	ORIENTALIS	CHINESE DWARF	NEWT	LIV	500	NO	HK	HK	T	C	C	C	9/7/08	9/7/08	I	LA	DOLPHIN INTERNATIONAL
2008283287	PACB	PACHYTRITON	BREVIPES	TSITOU	NEWT	LIV	200	NO	HK	HK	T	C	C	C	9/7/08	9/7/08	I	LA	DOLPHIN INTERNATIONAL
2008283291	TRIH	TRITURUS	HONGKONGENSIS	ALPINE	NEWT	LIV	50	NO	HK	HK	T	W	C	C	9/7/08	9/7/08	I	LA	AQUACO, INC.
2008283958	CYPY	CYNOPS	PYRRHOGASTER	JAPANESE FIREBELLY	NEWT	LIV	105	NO	HK	HK	T	W	C	C	9/8/08	9/6/08	I	NY	Transship Discounts Ltd.
2008283958	PACB	PACHYTRITON	BREVIPES	TSITOU	NEWT	LIV	90	NO	HK	HK	T	W	C	C	9/8/08	9/6/08	I	NY	Transship Discounts Ltd.
2008284480	CYOR	CYNOPS	ORIENTALIS	CHINESE DWARF	NEWT	LIV	100	NO	SG	SG	T	W	C	C	2/6/08	1/13/08	I	NY	INTERNATIONAL PET RESOURCES , LLC
2008284499	TRIH	TRITURUS	HONGKONGENSIS	ALPINE	NEWT	LIV	50	NO	HK	HK	T	C	C	C	10/21/07	10/21/07	I	NY	ERDA INCORPORATED
2008284626	CYOR	CYNOPS	ORIENTALIS	CHINESE DWARF	NEWT	LIV	100	NO	SG	SG	T	W	C	C	1/27/08	1/27/08	I	NY	INTERNATIONAL PET RESOURCES , LLC
2008284688	CYPY	CYNOPS	PYRRHOGASTER	JAPANESE FIREBELLY	NEWT	LIV	50	NO	HK	HK	T	W	C	C	1/26/08	1/26/08	I	NY	ERDA INCORPORATED
2008285389	PACB	PACHYTRITON	BREVIPES	TSITOU	NEWT	LIV	60	NO	HK	HK	T	W	C	C	9/10/08	9/7/08	I	NY	Transship Discounts Ltd.
2008285856	CYOR	CYNOPS	ORIENTALIS	CHINESE DWARF	NEWT	LIV	100	NO	SG	SG	T	W	C	C	12/17/07	12/2/07	I	NY	INTERNATIONAL PET RESOURSES
2008286028	CYOR	CYNOPS	ORIENTALIS	CHINESE DWARF	NEWT	LIV	200	NO	SG	SG	T	W	C	C	3/2/08	3/2/08	I	NY	INTERNATIONAL PET RESOURCES , LLC
2008286858	CYOR	CYNOPS	ORIENTALIS	CHINESE DWARF	NEWT	LIV	2,000	NO	CN	CN	T	W	C	C	9/12/08	9/12/08	I	LA	Z IMPORTS INC.
2008287318	CYOR	CYNOPS	ORIENTALIS	CHINESE DWARF	NEWT	LIV	500	NO	HK	HK	T	C	C	C	9/13/08	9/13/08	I	LA	DOLPHIN INTERNATIONAL
2008287363	CYOR	CYNOPS	ORIENTALIS	CHINESE DWARF	NEWT	LIV	1,000	NO	CN	CN	T	W	C	C	9/13/08	9/13/08	I	LA	WORLD WIDE TRADING INC.
2008287407	TRIH	TRITURUS	HONGKONGENSIS	ALPINE	NEWT	LIV	450	NO	HK	HK	T	W	C	C	9/13/08	9/14/08	I	LA	AQUACO, INC.
2008287430	CYOR	CYNOPS	ORIENTALIS	CHINESE DWARF	NEWT	LIV	1,000	NO	HK	HK	T	C	C	C	9/14/08	9/14/08	I	LA	DOLPHIN INTERNATIONAL
2008287979	CYOR	CYNOPS	ORIENTALIS	CHINESE DWARF	NEWT	LIV	100	NO	SG	SG	T	W	C	C	1/14/08	12/28/07	I	NY	INTERNATIONAL PET RESOURCES , LLC
2008288112	CYOR	CYNOPS	ORIENTALIS	CHINESE DWARF	NEWT	LIV	100	NO	SG	SG	T	W	C	C	1/23/08	1/6/08	I	NY	INTERNATIONAL PET RESOURCES , LLC
2008288191	CYOR	CYNOPS	ORIENTALIS	CHINESE DWARF	NEWT	LIV	100	NO	SG	SG	T	W	C	C	1/25/08	12/9/07	I	NY	INTERNATIONAL PET RESOURCES , LLC
2008289753	CYPY	CYNOPS	PYRRHOGASTER	JAPANESE FIREBELLY	NEWT	LIV	105	NO	HK	HK	T	W	C	C	9/17/08	9/14/08	I	NY	Transship Discounts Ltd.
2008290843	AMBY	AMBYSTOMA	SPECIES	MOLE	SALAMANDER	LIV	10	NO	DE	DE	T	C	C	C	9/18/08	9/16/08	I	DF	U.S. GLOBAL EXOTICS, INC.
2008290843	TYL?	TYLOTOTRITON	SPECIES	CROCODILE	NEWT	LIV	21	NO	DE	DE	T	C	C	C	9/18/08	9/16/08	I	DF	U.S. GLOBAL EXOTICS, INC.
2008290843	CNP?	CYNOPS	SPECIES	FIREBELLY	NEWT	LIV	50	NO	DE	DE	T	C	C	C	9/18/08	9/16/08	I	DF	U.S. GLOBAL EXOTICS, INC.
2008290843	TRIT	TRITURUS	SPECIES		NEWT	LIV	50	NO	DE	DE	T	C	C	C	9/18/08	9/16/08	I	DF	U.S. GLOBAL EXOTICS, INC.
2008290843	SASA	SALAMANDRA	SALAMANDRA	EUROPEAN FIRE	SALAMANDER	LIV	50	NO	DE	DE	T	C	C	C	9/18/08	9/16/08	I	DF	U.S. GLOBAL EXOTICS, INC.
2008290843	TYKW	TYLOTOTRITON	KWEICHOWENSIS	KWEICHOW CROCODILE	NEWT	LIV	22	NO	DE	DE	T	C	C	C	9/18/08	9/16/08	I	DF	U.S. GLOBAL EXOTICS, INC.
2008291327	CYOR	CYNOPS	ORIENTALIS	CHINESE DWARF	NEWT	LIV	250	NO	HK	HK	T	C	C	C	9/19/08	9/20/08	I	LA	DOLPHIN INTERNATIONAL
2008291507	CYOR	CYNOPS	ORIENTALIS	CHINESE DWARF	NEWT	LIV	2,000	NO	CN	CN	T	W	C	C	9/20/08	9/20/08	I	LA	Z IMPORTS INC.
2008291579	PACB	PACHYTRITON	BREVIPES	TSITOU	NEWT	LIV	100	NO	HK	HK	T	C	C	C	9/21/08	9/21/08	I	LA	DOLPHIN INTERNATIONAL
2008291601	TRIH	TRITURUS	HONGKONGENSIS	ALPINE	NEWT	LIV	150	NO	HK	HK	T	W	C	C	9/21/08	9/21/08	I	LA	AQUACO, INC.
2008291607	CYOR	CYNOPS	ORIENTALIS	CHINESE DWARF	NEWT	LIV	1,625	NO	CN	CN	T	W	C	C	9/21/08	9/21/08	I	LA	AQUA-NAUTIC SPECIALIST
2008291624	CNP?	CYNOPS	SPECIES	FIREBELLY	NEWT	LIV	300	NO	NO	CN	T	C	C	C	9/22/08	9/22/08	I	AT	SUN PET LTD.
2008292887	TRIH	TRITURUS	HONGKONGENSIS	ALPINE	NEWT	LIV	1,600	NO	HK	HK	T	C	C	C	9/23/08	9/22/08	I	TP	5-D TROPICAL INC.
2008294842	TRIH	TRITURUS	HONGKONGENSIS	ALPINE	NEWT	LIV	1,200	NO	HK	HK	T	W	C	C	9/27/08	9/28/08	I	LA	AQUACO, INC.
2008295104	CYOR	CYNOPS	ORIENTALIS	CHINESE DWARF	NEWT	LIV	1,250	NO	HK	HK	T	C	C	C	9/29/08	9/29/08	I	LA	DOLPHIN INTERNATIONAL
2008295104	PACB	PACHYTRITON	BREVIPES	TSITOU	NEWT	LIV	100	NO	HK	HK	T	C	C	C	9/29/08	9/29/08	I	LA	DOLPHIN INTERNATIONAL
2008297919	TRIH	TRITURUS	HONGKONGENSIS	ALPINE	NEWT	LIV	1,200	NO	HK	HK	T	W	C	C	10/4/08	10/5/08	I	LA	AQUACO, INC.
2008297937	CYOR	CYNOPS	ORIENTALIS	CHINESE DWARF	NEWT	LIV	500	NO	HK	HK	T	C	C	C	10/4/08	10/5/08	I	LA	DOLPHIN INTERNATIONAL
2008297937	PACB	PACHYTRITON	BREVIPES	TSITOU	NEWT	LIV	200	NO	HK	HK	T	C	C	C	10/4/08	10/5/08	I	LA	DOLPHIN INTERNATIONAL
2008298273	CNP?	CYNOPS	SPECIES	FIREBELLY	NEWT	LIV	300	NO	CN	CN	T	C	C	C	10/6/08	10/6/08	I	AT	SUN PET LTD.
2008298892	TRIH	TRITURUS	HONGKONGENSIS	ALPINE	NEWT	LIV	1,600	NO	HK	HK	T	C	C	C	10/7/08	10/7/08	I	TP	5-D TROPICAL INC.
2008299428	CYPY	CYNOPS	PYRRHOGASTER	JAPANESE FIREBELLY	NEWT	LIV	105	NO	HK	HK	T	W	C	C	10/8/08	10/5/08	I	NY	Transship Discounts Ltd.
2008300681	CYOR	CYNOPS	ORIENTALIS	CHINESE DWARF	NEWT	LIV	2,000	NO	CN	CN	T	W	C	C	10/10/08	10/10/08	I	LA	Z IMPORTS INC.
2008300943	PACB	PACHYTRITON	BREVIPES	TSITOU	NEWT	LIV	100	NO	HK	HK	T	C	C	C	10/10/08	10/11/08	I	LA	DOLPHIN INTERNATIONAL
2008301064	TRIH	TRITURUS	HONGKONGENSIS	ALPINE	NEWT	LIV	200	NO	HK	HK	T	W	C	C	10/12/08	10/12/08	I	LA	AQUACO, INC.
2008301092	CYOR	CYNOPS	ORIENTALIS	CHINESE DWARF	NEWT	LIV	600	NO	HK	HK	T	C	C	C	10/12/08	10/12/08	I	LA	DOLPHIN INTERNATIONAL
2008301092	PACB	PACHYTRITON	BREVIPES	TSITOU	NEWT	LIV	300	NO	HK	HK	T	C	C	C	10/12/08	10/12/08	I	LA	DOLPHIN INTERNATIONAL
2008301156	CYOR	CYNOPS	ORIENTALIS	CHINESE DWARF	NEWT	LIV	1,000	NO	CN	CN	T	W	C	C	10/13/08	10/13/08	I	LA	WORLD WIDE TRADING INC.

2008301421	PACB	PACHYTRITON	BREVIPES		TSITOU	NEWT	LIV	60	NO	HK	HK	T	W	C	C	10/14/08	10/12/08	I	NY	Transship Discounts Ltd.
2008303063	CYOR	CYNOPS	ORIENTALIS		CHINESE DWARF	NEWT	LIV	300	NO	HK	HK	T	W	C	C	10/16/08	10/15/08	I	CH	AQUATICS INC
2008303283	NOVI	NOTOPHTHALMUS	VIRIDESCENS		EASTERN	NEWT	LIV	8	NO	US	CA	T	W	C	C	10/15/08	10/15/08	I	BN	Science Kit, Inc - Boreal
2008303395	AMBY	AMBYSTOMA	SPECIES		MOLE	SALAMANDER	LIV	10	NO	GE	DE	T	C	C	C	10/14/08	10/14/08	I	PT	Exemptions 6 and 7(C)
2008303395	SAL?	SALAMANDRA	SPECIES		FIRE	SALAMANDER	LIV	3	NO	GE	DE	T	C	C	C	10/14/08	10/14/08	I	PT	Exemptions 6 and 7(C)
2008303395	SAL?	SALAMANDRA	SPECIES		FIRE	SALAMANDER	LIV	4	NO	GE	DE	T	C	C	C	10/14/08	10/14/08	I	PT	Exemptions 6 and 7(C)
2008303395	TRIT	TRITURUS	SPECIES			NEWT	LIV	11	NO	GE	DE	T	C	C	C	10/14/08	10/14/08	I	PT	Exemptions 6 and 7(C)
2008303395	SASA	SALAMANDRA	SALAMANDRA		EUROPEAN FIRE	SALAMANDER	LIV	7	NO	GE	DE	T	C	C	C	10/14/08	10/14/08	I	PT	Exemptions 6 and 7(C)
2008303395	SAL?	SALAMANDRA	SPECIES		FIRE	SALAMANDER	LIV	40	NO	GE	DE	T	C	C	C	10/14/08	10/14/08	I	PT	Exemptions 6 and 7(C)
2008303800	PLAB	PACHYTRITON	LABIATUS		UNTERSTEIN'S	NEWT	LIV	300	NO	HK	HK	T	W	C	C	10/17/08	10/18/08	I	DF	U.S. GLOBAL EXOTICS, INC.
2008304245	TRIH	TRITURUS	HONGKONGENSIS		ALPINE	NEWT	LIV	150	NO	HK	HK	T	C	C	C	10/18/08	10/19/08	I	LA	AQUACO, INC.
2008304260	NEW?	PACHYTRITON	SPECIES		CHINESE	NEWT	LIV	100	NO	CN	CN	T	W	C	C	10/18/08	10/19/08	I	LA	AQUA-NAUTIC SPECIALIST
2008304277	CYOR	CYNOPS	ORIENTALIS		CHINESE DWARF	NEWT	LIV	500	NO	HK	HK	T	C	C	C	10/19/08	10/19/08	I	LA	DOLPHIN INTERNATIONAL
2008304303	CNP?	CYNOPS	SPECIES		FIREBELLY	NEWT	LIV	300	NO	CN	CN	T	C	C	C	10/20/08	10/20/08	I	AT	SUN PET LTD.
2008306427	PHNG	PARAMESOTRITON	HONGKONGENSIS		HONG KONG WARTY	NEWT	LIV	1,600	NO	HK	HK	T	C	C	C	7/25/06	7/25/06	I	TP	5-D TROPICAL, INC.
2008306565	AMTS	AMBYSTOMA	TIGRINUM	STEBBINSI	SONORA TIGER	SALAMANDER	LIV	98	NO	US	GE	T	W	C	C	10/21/08	10/21/08	I	PT	Exemptions 6 and 7(C)
2008306565	AMBY	AMBYSTOMA	SPECIES		MOLE	SALAMANDER	LIV	27	NO	US	GE	T	W	C	C	10/21/08	10/21/08	I	PT	Exemptions 6 and 7(C)
2008306565	AMMA	AMBYSTOMA	MACRODACTYLUM		LONG-TOED	SALAMANDER	LIV	5	NO	US	GE	T	W	C	C	10/21/08	10/21/08	I	PT	Exemptions 6 and 7(C)
2008306565	AMBA	AMBYSTOMA	MACULATUM		SPOTTED	SALAMANDER	LIV	4	NO	US	GE	T	W	C	C	10/21/08	10/21/08	I	PT	Exemptions 6 and 7(C)
2008306565	PLET	PLETHODON	SPECIES			SALAMANDER	LIV	24	NO	US	GE	T	W	C	C	10/21/08	10/21/08	I	PT	Exemptions 6 and 7(C)
2008306565	ALAT	AMBYSTOMA	LATERALE		BLUE-SPOTTED	SALAMANDER	LIV	1	NO	US	GE	T	W	C	C	10/21/08	10/21/08	I	PT	Exemptions 6 and 7(C)
2008306565	GSA?	DICAMPTODON	SPECIES		GIANT	SALAMANDER	LIV	7	NO	US	GE	T	W	C	C	10/21/08	10/21/08	I	PT	Exemptions 6 and 7(C)
2008306565	OLS?	RHYACOTRITON	SPECIES		OLYMPIC	SALAMANDER	LIV	6	NO	US	GE	T	W	C	C	10/21/08	10/21/08	I	PT	Exemptions 6 and 7(C)
2008306764	TRIH	TRITURUS	HONGKONGENSIS		ALPINE	NEWT	LIV	1,600	NO	HK	HK	T	C	C	C	5/31/06	5/31/06	I	TP	5-D TROPICAL, INC.
2008308562	CYOR	CYNOPS	ORIENTALIS		CHINESE DWARF	NEWT	LIV	250	NO	HK	HK	T	C	C	C	10/25/08	10/25/08	I	LA	DOLPHIN INTERNATIONAL
2008308655	TRIH	TRITURUS	HONGKONGENSIS		ALPINE	NEWT	LIV	350	NO	HK	HK	T	C	C	C	10/25/08	10/26/08	I	LA	AQUACO, INC.
2008308673	CYOR	CYNOPS	ORIENTALIS		CHINESE DWARF	NEWT	LIV	600	NO	HK	HK	T	C	C	C	10/25/08	10/26/08	I	LA	DOLPHIN INTERNATIONAL
2008308673	PACB	PACHYTRITON	BREVIPES		TSITOU	NEWT	LIV	200	NO	HK	HK	T	C	C	C	10/25/08	10/26/08	I	LA	DOLPHIN INTERNATIONAL
2008309237	CYPY	CYNOPS	PYRRHOGASTER		JAPANESE FIREBELLY	NEWT	LIV	210	NO	HK	HK	T	W	C	C	10/27/08	10/26/08	I	NY	Transship Discounts Ltd.
2008309237	PACB	PACHYTRITON	BREVIPES		TSITOU	NEWT	LIV	60	NO	HK	HK	T	W	C	C	10/27/08	10/26/08	I	NY	Transship Discounts Ltd.
2008309274	NOVI	NOTOPHTHALMUS	VIRIDESCENS		EASTERN	NEWT	LIV	7	NO	US	CA	T	W	C	C	10/27/08	10/27/08	I	BN	Science Kit, Inc - Boreal
2008309686	TRIH	TRITURUS	HONGKONGENSIS		ALPINE	NEWT	LIV	1,600	NO	HK	HK	T	C	C	C	3/15/06	3/15/06	I	TP	5-D TROPICAL, INC.
2008310038	WNEW	PARAMESOTRITON	SPECIES		WARTY	NEWT	LIV	1	NO	CN	CN	S	W	C	C	10/28/08	10/29/08	I	SF	Museum of Vertebrate Zoology
2008310733	TRIH	TRITURUS	HONGKONGENSIS		ALPINE	NEWT	LIV	1,600	NO	HK	HK	T	C	C	C	1/10/06	1/10/06	I	TP	5-D TROPICAL, INC.
2008311125	TRIH	TRITURUS	HONGKONGENSIS		ALPINE	NEWT	LIV	1,600	NO	HK	HK	T	C	C	C	10/29/08	10/29/08	I	TP	5-D TROPICAL INC.
2008311993	NOVI	NOTOPHTHALMUS	VIRIDESCENS		EASTERN	NEWT	LIV	8	NO	US	CA	T	W	C	C	10/31/08	10/20/08	I	BN	Science Kit, Inc - Boreal
2008311997	TRIH	TRITURUS	HONGKONGENSIS		ALPINE	NEWT	LIV	1,600	NO	HK	HK	T	C	C	C	2/2/06	2/2/06	I	TP	5-D TROPICAL, INC.
2008312601	CYOR	CYNOPS	ORIENTALIS		CHINESE DWARF	NEWT	LIV	250	NO	HK	HK	T	C	C	C	11/1/08	11/1/08	I	LA	DOLPHIN INTERNATIONAL
2008312714	CYOR	CYNOPS	ORIENTALIS		CHINESE DWARF	NEWT	LIV	1,840	NO	CN	CN	T	C	C	C	11/2/08	11/2/08	I	LA	AQUA-NAUTIC SPECIALIST
2008312732	CYOR	CYNOPS	ORIENTALIS		CHINESE DWARF	NEWT	LIV	800	NO	HK	HK	T	C	C	C	11/2/08	11/2/08	I	LA	DOLPHIN INTERNATIONAL
2008312732	PACB	PACHYTRITON	BREVIPES		TSITOU	NEWT	LIV	100	NO	HK	HK	T	C	C	C	11/2/08	11/2/08	I	LA	DOLPHIN INTERNATIONAL
2008312750	CNP?	CYNOPS	SPECIES		FIREBELLY	NEWT	LIV	300	NO	CN	CN	T	C	C	C	11/3/08	11/3/08	I	AT	SUN PET LTD.
2008312952	TYVE	TYLOTOTRITON	VERRUCOSUS		CROCODILE	NEWT	LIV	1	NO	XX	NL	T	U	R	S	10/30/08	10/28/08	I	NW	Exemptions 6 and 7(C)
2008312954	NRR?	NEURERGUS	SPECIES			NEWT	LIV	32	NO	CA	CA	T	C	C	C	11/3/08	11/3/08	I	NY	Exemptions 6 and 7(C)
2008312954	TYL?	TYLOTOTRITON	SPECIES		CROCODILE	NEWT	LIV	34	NO	CA	CA	T	C	C	C	11/3/08	11/3/08	I	NY	Exemptions 6 and 7(C)
2008312954	TYKW	TYLOTOTRITON	KWEICHOWENSIS		KWEICHOW CROCODILE	NEWT	LIV	11	NO	CA	CA	T	C	C	C	11/3/08	11/3/08	I	NY	Exemptions 6 and 7(C)
2008312954	HYN?	HYNOBIUS	SPECIES			SALAMANDER	LIV	6	NO	CA	CA	T	C	C	C	11/3/08	11/3/08	I	NY	Exemptions 6 and 7(C)
2008312954	HYN?	HYNOBIUS	SPECIES			SALAMANDER	LIV	8	NO	CA	CA	T	C	C	C	11/3/08	11/3/08	I	NY	Exemptions 6 and 7(C)
2008313212	TRIH	TRITURUS	HONGKONGENSIS		ALPINE	NEWT	LIV	1,600	NO	HK	HK	T	C	C	C	6/14/06	6/14/06	I	TP	5-D TROPICAL, INC.
2008313256	NOVI	NOTOPHTHALMUS	VIRIDESCENS		EASTERN	NEWT	LIV	4	NO	US	CA	T	W	C	C	11/3/08	11/3/08	I	BN	Science Kit, Inc - Boreal
2008313289	TRIH	TRITURUS	HONGKONGENSIS		ALPINE	NEWT	LIV	300	NO	HK	HK	T	C	C	C	6/22/06	6/22/06	I	TP	SEGREST FARMS
2008313327	TRIH	TRITURUS	HONGKONGENSIS		ALPINE	NEWT	LIV	400	NO	HK	HK	T	C	C	C	6/30/06	6/29/06	I	TP	SEGREST FARMS
2008313393	CYPY	CYNOPS	PYRRHOGASTER		JAPANESE FIREBELLY	NEWT	LIV	210	NO	HK	HK	T	W	C	C	11/3/08	11/2/08	I	NY	Transship Discounts Ltd.
2008313653	NOVI	NOTOPHTHALMUS	VIRIDESCENS		EASTERN	NEWT	LIV	7	NO	US	CA	T	W	C	C	10/29/08	10/29/08	I	BN	Science Kit, Inc - Boreal
2008315602	NOVI	NOTOPHTHALMUS	VIRIDESCENS		EASTERN	NEWT	LIV	3	NO	US	CA	T	W	C	C	11/6/08	11/5/08	I	BN	Science Kit, Inc - Boreal
2008316892	CYOR	CYNOPS	ORIENTALIS		CHINESE DWARF	NEWT	LIV	250	NO	HK	HK	T	C	C	C	11/8/08	11/8/08	I	LA	DOLPHIN INTERNATIONAL
2008316919	TRIH	TRITURUS	HONGKONGENSIS		ALPINE	NEWT	LIV	1,200	NO	HK	HK	T	C	C	C	11/8/08	11/9/08	I	LA	AQUACO, INC.
2008316954	CYOR	CYNOPS	ORIENTALIS		CHINESE DWARF	NEWT	LIV	850	NO	HK	HK	T	C	C	C	11/9/08	11/9/08	I	LA	DOLPHIN INTERNATIONAL
2008317745	NOVI	NOTOPHTHALMUS	VIRIDESCENS		EASTERN	NEWT	LIV	5	NO	US	CA	T	W	C	C	11/10/08	11/10/08	I	BN	Science Kit, Inc - Boreal
2008318578	CYOR	CYNOPS	ORIENTALIS		CHINESE DWARF	NEWT	LIV	100	NO	XX	SG	T	W	C	C	1/8/08	12/16/07	I	NY	INTERNATIONAL PET RESOURCES , LLC

2008318676	CYPY	CYNOPS	PYRRHOGASTER	JAPANESE FIREBELLY	NEWT	LIV	50	NO	HK	HK	T	W	C	C	2/21/08	2/16/08	I	NY	ERDA INCORPORATED
2008319462	NOVI	NOTOPHTHALMUS	VIRIDESCENS	EASTERN	NEWT	LIV	2	NO	US	CA	T	W	C	C	11/12/08	11/12/08	I	BN	Science Kit, Inc - Boreal
2008319471	CYPY	CYNOPS	PYRRHOGASTER	JAPANESE FIREBELLY	NEWT	LIV	330	NO	HK	HK	T	W	C	C	11/13/08	11/9/08	I	NY	Transship Discounts Ltd.
2008319948	CYPY	CYNOPS	PYRRHOGASTER	JAPANESE FIREBELLY	NEWT	LIV	150	NO	HK	HK	T	W	C	C	1/26/08	1/19/08	I	NY	ERDA INCORPORATED
2008320311	CNP?	CYNOPS	SPECIES	FIREBELLY	NEWT	LIV	0	NO	CN	CN	T	W	C	C	10/12/07	10/7/07	I	NY	FISH MART INCORPORATED
2008320311	CNP?	CYNOPS	SPECIES	FIREBELLY	NEWT	LIV	0	NO	CN	CN	T	W	C	C	10/12/07	10/7/07	I	NY	FISH MART INCORPORATED
2008320493	CNP?	CYNOPS	SPECIES	FIREBELLY	NEWT	LIV	50	NO	HK	HK	T	W	C	C	1/3/08	12/15/07	I	NY	ERDA INCORPORATED
2008321053	CYOR	CYNOPS	ORIENTALIS	CHINESE DWARF	NEWT	LIV	250	NO	HK	HK	T	C	C	C	11/15/08	11/15/08	I	LA	DOLPHIN INTERNATIONAL
2008321172	CYOR	CYNOPS	ORIENTALIS	CHINESE DWARF	NEWT	LIV	1,400	NO	HK	HK	T	C	C	C	11/15/08	11/16/08	I	LA	DOLPHIN INTERNATIONAL
2008321172	PACB	PACHYTRITON	BREVIPES	TSITOU	NEWT	LIV	300	NO	HK	HK	T	C	C	C	11/15/08	11/16/08	I	LA	DOLPHIN INTERNATIONAL
2008321189	CNP?	CYNOPS	SPECIES	FIREBELLY	NEWT	LIV	300	NO	CN	CN	T	C	C	C	11/17/08	11/17/08	I	AT	SUN PET LTD.
2008321437	CYPY	CYNOPS	PYRRHOGASTER	JAPANESE FIREBELLY	NEWT	LIV	100	NO	HK	HK	T	C	C	C	3/7/08	3/1/08	I	NY	ERDA INCORPORATED
2008321505	CYPY	CYNOPS	PYRRHOGASTER	JAPANESE FIREBELLY	NEWT	LIV	50	NO	HK	HK	T	W	C	C	2/29/08	2/23/08	I	NY	ERDA INCORPORATED
2008321602	CYOR	CYNOPS	ORIENTALIS	CHINESE DWARF	NEWT	LIV	100	NO	SG	SG	T	W	C	C	4/27/08	4/27/08	I	NY	INTERNATIONAL PET RESOURCES , LLC
2008321679	CYPY	CYNOPS	PYRRHOGASTER	JAPANESE FIREBELLY	NEWT	LIV	100	NO	HK	HK	T	W	C	C	5/2/08	4/27/08	I	NY	ERDA INCORPORATED
2008323170	CYOR	CYNOPS	ORIENTALIS	CHINESE DWARF	NEWT	LIV	0	NO	SG	SG	T	W	C	C	5/4/08	5/4/08	I	NY	INTERNATIONAL PET RESOURCES , LLC
2008324133	PLWA	PLEURODELES	WALTL	IBERIAN RIBBED	NEWT	LIV	247	NO	CZ	CZ	T	C	C	C	11/21/08	11/19/08	I	MI	STRICTLY REPTILES, INC.
2008324869	CYOR	CYNOPS	ORIENTALIS	CHINESE DWARF	NEWT	LIV	250	NO	HK	HK	T	C	C	C	11/22/08	11/22/08	I	LA	DOLPHIN INTERNATIONAL
2008324960	PLAB	PACHYTRITON	LABIATUS	UNTERSTEIN'S	NEWT	LIV	545	NO	CN	CN	T	C	C	C	11/22/08	11/23/08	I	LA	AQUA-NAUTIC SPECIALIST
2008324975	CYOR	CYNOPS	ORIENTALIS	CHINESE DWARF	NEWT	LIV	250	NO	HK	HK	T	C	C	C	11/23/08	11/23/08	I	LA	DOLPHIN INTERNATIONAL
2008325336	CYOR	CYNOPS	ORIENTALIS	CHINESE DWARF	NEWT	LIV	300	NO	HK	HK	T	C	C	C	11/24/08	11/24/08	I	CH	AQUATICS INC
2008326739	NOVI	NOTOPHTHALMUS	VIRIDESCENS	EASTERN	NEWT	LIV	2	NO	US	CA	T	W	C	C	11/25/08	11/24/08	I	BN	Science Kit, Inc - Boreal
2008327925	CYOR	CYNOPS	ORIENTALIS	CHINESE DWARF	NEWT	LIV	2,500	NO	CN	CN	T	W	C	C	11/28/08	11/28/08	I	LA	Z IMPORTS INC.
2008328273	PACB	PACHYTRITON	BREVIPES	TSITOU	NEWT	LIV	100	NO	HK	HK	T	C	C	C	11/29/08	11/29/08	I	LA	DOLPHIN INTERNATIONAL
2008328354	TRIH	TRITURUS	HONGKONGENSIS	ALPINE	NEWT	LIV	50	NO	HK	HK	T	C	C	C	11/29/08	11/30/08	I	LA	AQUACO, INC.
2008328360	PLAB	PACHYTRITON	LABIATUS	UNTERSTEIN'S	NEWT	LIV	200	NO	CN	CN	T	C	C	C	11/29/08	11/30/08	I	LA	AQUA-NAUTIC SPECIALIST
2008328368	CYOR	CYNOPS	ORIENTALIS	CHINESE DWARF	NEWT	LIV	450	NO	HK	HK	T	C	C	C	11/29/08	11/30/08	I	LA	DOLPHIN INTERNATIONAL
2008329577	NOVI	NOTOPHTHALMUS	VIRIDESCENS	EASTERN	NEWT	LIV	14	NO	US	CA	T	W	C	C	12/2/08	11/19/08	I	BN	Science Kit, Inc - Boreal
2008329881	CYPY	CYNOPS	PYRRHOGASTER	JAPANESE FIREBELLY	NEWT	LIV	420	NO	HK	HK	T	W	C	C	12/3/08	11/30/08	I	NY	Transship Discounts Ltd.
2008329981	TRIT	TRITURUS	SPECIES		NEWT	LIV	42	NO	NL	NL	T	C	C	C	12/3/08	12/3/08	I	DF	U.S. GLOBAL EXOTICS, INC.
2008329981	PLWA	PLEURODELES	WALTL	IBERIAN RIBBED	NEWT	LIV	614	NO	NL	NL	T	C	C	C	12/3/08	12/3/08	I	DF	U.S. GLOBAL EXOTICS, INC.
2008330220	NOVI	NOTOPHTHALMUS	VIRIDESCENS	EASTERN	NEWT	LIV	8	NO	US	CA	T	W	C	C	12/3/08	12/3/08	I	BN	Science Kit, Inc - Boreal
2008330576	CYPY	CYNOPS	PYRRHOGASTER	JAPANESE FIREBELLY	NEWT	LIV	140	NO	HK	HK	T	W	C	C	6/19/08	6/14/08	I	NY	ERDA INCORPORATED
2008331092	CNP?	CYNOPS	SPECIES	FIREBELLY	NEWT	LIV	200	NO	SG	SG	T	W	C	C	4/13/08	4/13/08	I	NY	INTERNATIONAL PET RESOURCES , LLC
2008332062	CYOR	CYNOPS	ORIENTALIS	CHINESE DWARF	NEWT	LIV	500	NO	HK	HK	T	C	C	C	12/6/08	12/6/08	I	LA	DOLPHIN INTERNATIONAL
2008332062	PACB	PACHYTRITON	BREVIPES	TSITOU	NEWT	LIV	100	NO	HK	HK	T	C	C	C	12/6/08	12/6/08	I	LA	DOLPHIN INTERNATIONAL
2008332151	TRIH	TRITURUS	HONGKONGENSIS	ALPINE	NEWT	LIV	250	NO	HK	HK	T	C	C	C	12/6/08	12/7/08	I	LA	AQUACO, INC.
2008332174	CYOR	CYNOPS	ORIENTALIS	CHINESE DWARF	NEWT	LIV	1,250	NO	HK	HK	T	C	C	C	12/6/08	12/7/08	I	LA	DOLPHIN INTERNATIONAL
2008332189	CNP?	CYNOPS	SPECIES	FIREBELLY	NEWT	LIV	300	NO	CN	CN	T	C	C	C	12/8/08	12/8/08	I	AT	SUN PET LTD.
2008333037	CYPY	CYNOPS	PYRRHOGASTER	JAPANESE FIREBELLY	NEWT	LIV	60	NO	HK	HK	T	W	C	C	12/9/08	12/7/08	I	NY	Transship Discounts Ltd.
2008333255	CNP?	CYNOPS	SPECIES	FIREBELLY	NEWT	LIV	200	NO	CN	CN	T	W	C	C	5/29/08	5/11/08	I	NY	FISH MART INCORPORATED
2008333255	CNP?	CYNOPS	SPECIES	FIREBELLY	NEWT	LIV	0	NO	CN	CN	T	W	C	C	5/29/08	5/11/08	I	NY	FISH MART INCORPORATED
2008334470	NOVI	NOTOPHTHALMUS	VIRIDESCENS	EASTERN	NEWT	LIV	2	NO	US	CA	T	W	C	C	12/10/08	12/10/08	I	BN	Science Kit, Inc - Boreal
2008335120	SASA	SALAMANDRA	SALAMANDRA	EUROPEAN FIRE	SALAMANDER	LIV	1,710	NO	SI	SI	T	C	C	C	12/11/08	12/11/08	I	DF	U.S. GLOBAL EXOTICS, INC.
2008335925	CYOR	CYNOPS	ORIENTALIS	CHINESE DWARF	NEWT	LIV	1,050	NO	HK	HK	T	C	C	C	12/13/08	12/14/08	I	LA	DOLPHIN INTERNATIONAL
2008336223	NOVI	NOTOPHTHALMUS	VIRIDESCENS	EASTERN	NEWT	LIV	1	NO	US	CA	T	W	C	C	12/15/08	12/15/08	I	BN	Science Kit, Inc - Boreal
2008336323	CYPY	CYNOPS	PYRRHOGASTER	JAPANESE FIREBELLY	NEWT	LIV	60	NO	HK	HK	T	W	C	C	12/15/08	12/14/08	I	NY	Transship Discounts Ltd.
2008338941	BOG?	BOLITOGLOSSA	SPECIES	MUSHROOMTONGUE	SALAMANDER	LIV	16	NO	MX	MX	S	W	C	C	12/19/08	11/23/08	I	DF	BELL MUSEUM OF NATURAL HISTOR, UNIV. OF MINNESOTA
2008338941	PDE?	PSEUDOEURYCEA	SPECIES	FALSE BROOK	SALAMANDER	LIV	47	NO	MX	MX	S	W	C	C	12/19/08	11/23/08	I	DF	BELL MUSEUM OF NATURAL HISTOR, UNIV. OF MINNESOTA
2008339080	CYOR	CYNOPS	ORIENTALIS	CHINESE DWARF	NEWT	LIV	600	NO	HK	HK	T	C	C	C	12/20/08	12/20/08	I	LA	DOLPHIN INTERNATIONAL
2008339192	CYOR	CYNOPS	ORIENTALIS	CHINESE DWARF	NEWT	LIV	350	NO	HK	HK	T	C	C	C	12/21/08	12/21/08	I	LA	DOLPHIN INTERNATIONAL
2008340945	CYOR	CYNOPS	ORIENTALIS	CHINESE DWARF	NEWT	LIV	100	NO	HK	HK	T	C	C	C	12/27/08	12/27/08	I	LA	DOLPHIN INTERNATIONAL
2008340945	PACB	PACHYTRITON	BREVIPES	TSITOU	NEWT	LIV	100	NO	HK	HK	T	C	C	C	12/27/08	12/27/08	I	LA	DOLPHIN INTERNATIONAL
2008341079	CYOR	CYNOPS	ORIENTALIS	CHINESE DWARF	NEWT	LIV	250	NO	HK	HK	T	C	C	C	12/27/08	12/28/08	I	LA	DOLPHIN INTERNATIONAL
2008341474	CYPY	CYNOPS	PYRRHOGASTER	JAPANESE FIREBELLY	NEWT	LIV	60	NO	HK	HK	T	W	C	C	12/29/08	12/28/08	I	NY	Transship Discounts Ltd.
2009343285	CYOR	CYNOPS	ORIENTALIS	CHINESE DWARF	NEWT	LIV	250	NO	HK	HK	T	C	C	C	1/3/09	1/3/09	I	LA	DOLPHIN INTERNATIONAL
2009343328	TRIH	TRITURUS	HONGKONGENSIS	ALPINE	NEWT	LIV	300	NO	HK	HK	T	C	C	C	1/3/09	1/4/09	I	LA	AQUACO, INC.
2009343333	CYOR	CYNOPS	ORIENTALIS	CHINESE DWARF	NEWT	LIV	940	NO	CN	CN	T	C	C	C	1/3/09	1/4/09	I	LA	AQUA-NAUTIC SPECIALIST

2009343354	CYOR	CYNOPS	ORIENTALIS	CHINESE DWARF	NEWT	LIV	1,400	NO	HK	HK	T	C	C	C	1/3/09	1/4/09	I	LA	DOLPHIN INTERNATIONAL
2009344033	CYOR	CYNOPS	ORIENTALIS	CHINESE DWARF	NEWT	LIV	100	NO	SG	SG	T	W	C	C	6/17/08	6/8/08	I	NY	INTERNATIONAL PET RESOURCES
2009344034	NOVI	NOTOPHTHALMUS	VIRIDESCENS	EASTERN	NEWT	LIV	4	NO	US	CA	T	W	C	C	1/5/09	1/5/09	I	BN	Science Kit, Inc - Boreal
2009344057	CYPY	CYNOPS	PYRRHOGASTER	JAPANESE FIREBELLY	NEWT	LIV	70	NO	HK	HK	T	C	C	C	5/27/08	5/24/08	I	NY	ERDA INCORPORATED
2009344317	CNP?	CYNOPS	SPECIES	FIREBELLY	NEWT	LIV	100	NO	SG	SG	T	W	C	C	5/23/08	5/11/08	I	NY	INTERNATIONAL PET RESOURCES , LLC
2009344388	CNP?	CYNOPS	SPECIES	FIREBELLY	NEWT	LIV	170	NO	CN	CN	T	W	C	C	6/24/08	6/22/08	I	NY	FISH MART INCORPORATED
2009344388	CNP?	CYNOPS	SPECIES	FIREBELLY	NEWT	LIV	150	NO	CN	CN	T	W	C	C	6/24/08	6/22/08	I	NY	FISH MART INCORPORATED
2009346137	TRIH	TRITURUS	HONGKONGENSIS	ALPINE	NEWT	LIV	1,600	NO	HK	HK	T	C	C	C	1/7/09	1/7/09	I	TP	5-D TROPICAL INC.
2009346158	CYPY	CYNOPS	PYRRHOGASTER	JAPANESE FIREBELLY	NEWT	LIV	165	NO	HK	HK	T	W	C	C	1/7/09	1/4/09	I	NY	Transship Discounts Ltd.
2009346158	PACB	PACHYTRITON	BREVIPES	TSITOU	NEWT	LIV	60	NO	HK	HK	T	W	C	C	1/7/09	1/4/09	I	NY	Transship Discounts Ltd.
2009346393	TYVE	TYLOTOTRITON	VERRUCOSUS	CROCODILE	NEWT	LIV	45	NO	SG	ID	T	W	C	C	6/22/08	6/22/08	I	NY	INTERNATIONAL PET RESOURCES , LLC
2009347206	CYPY	CYNOPS	PYRRHOGASTER	JAPANESE FIREBELLY	NEWT	LIV	100	NO	HK	HK	T	C	C	C	3/8/08	3/8/08	I	NY	ERDA INCORPORATED
2009347234	CYOR	CYNOPS	ORIENTALIS	CHINESE DWARF	NEWT	LIV	200	NO	SG	SG	T	W	C	C	2/17/08	2/17/08	I	NY	INTERNATIONAL PET RESOURCES , LLC
2009347928	CYOR	CYNOPS	ORIENTALIS	CHINESE DWARF	NEWT	LIV	350	NO	HK	HK	T	C	C	C	1/10/09	1/10/09	I	LA	DOLPHIN INTERNATIONAL
2009347928	PACB	PACHYTRITON	BREVIPES	TSITOU	NEWT	LIV	100	NO	HK	HK	T	C	C	C	1/10/09	1/10/09	I	LA	DOLPHIN INTERNATIONAL
2009348046	CYOR	CYNOPS	ORIENTALIS	CHINESE DWARF	NEWT	LIV	1,100	NO	HK	HK	T	C	C	C	1/10/09	1/11/09	I	LA	DOLPHIN INTERNATIONAL
2009348046	PACB	PACHYTRITON	BREVIPES	TSITOU	NEWT	LIV	200	NO	HK	HK	T	C	C	C	1/10/09	1/11/09	I	LA	DOLPHIN INTERNATIONAL
2009348060	CYOR	CYNOPS	ORIENTALIS	CHINESE DWARF	NEWT	LIV	1,240	NO	CN	CN	T	C	C	C	1/11/09	1/11/09	I	LA	AQUA-NAUTIC SPECIALIST
2009349363	NOVI	NOTOPHTHALMUS	VIRIDESCENS	EASTERN	NEWT	LIV	4	NO	US	CA	T	W	C	C	1/13/09	1/12/09	I	BN	Science Kit, Inc - Boreal
2009350114	PACB	PACHYTRITON	BREVIPES	TSITOU	NEWT	LIV	30	NO	HK	HK	T	W	C	C	1/13/09	1/11/09	I	NY	Transship Discounts Ltd.
2009351378	NOVI	NOTOPHTHALMUS	VIRIDESCENS	EASTERN	NEWT	LIV	1	NO	US	CA	T	W	C	C	1/15/09	1/14/09	I	BN	Science Kit, Inc - Boreal
2009351631	CYPY	CYNOPS	PYRRHOGASTER	JAPANESE FIREBELLY	NEWT	LIV	200	NO	HK	HK	T	C	C	C	4/6/08	4/5/07	I	NY	ERDA INCORPORATED
2009351745	CNP?	CYNOPS	SPECIES	FIREBELLY	NEWT	LIV	200	NO	CN	CN	T	W	C	C	3/30/08	3/30/08	I	NY	FISH MART INCORPORATED
2009352444	CYOR	CYNOPS	ORIENTALIS	CHINESE DWARF	NEWT	LIV	450	NO	HK	HK	T	C	C	C	1/17/09	1/17/09	I	LA	DOLPHIN INTERNATIONAL
2009352444	PACB	PACHYTRITON	BREVIPES	TSITOU	NEWT	LIV	100	NO	HK	HK	T	C	C	C	1/17/09	1/17/09	I	LA	DOLPHIN INTERNATIONAL
2009352547	TRIH	TRITURUS	HONGKONGENSIS	ALPINE	NEWT	LIV	450	NO	HK	HK	T	C	C	C	1/17/09	1/18/09	I	LA	AQUACO, INC.
2009352570	CYOR	CYNOPS	ORIENTALIS	CHINESE DWARF	NEWT	LIV	700	NO	HK	HK	T	C	C	C	1/17/09	1/18/09	I	LA	DOLPHIN INTERNATIONAL
2009352570	PACB	PACHYTRITON	BREVIPES	TSITOU	NEWT	LIV	100	NO	HK	HK	T	C	C	C	1/17/09	1/18/09	I	LA	DOLPHIN INTERNATIONAL
2009353039	CYOR	CYNOPS	ORIENTALIS	CHINESE DWARF	NEWT	LIV	200	NO	SG	SG	T	W	C	C	5/29/08	5/18/08	I	NY	INTERNATIONAL PET RESOURCES , LLC
2009353110	CNP?	CYNOPS	SPECIES	FIREBELLY	NEWT	LIV	250	NO	CN	CN	T	W	C	C	4/28/08	4/20/08	I	NY	FISH MART INCORPORATED
2009353110	CNP?	CYNOPS	SPECIES	FIREBELLY	NEWT	LIV	0	NO	CN	CN	T	W	C	C	4/28/08	4/20/08	I	NY	FISH MART INCORPORATED
2009353293	CYPY	CYNOPS	PYRRHOGASTER	JAPANESE FIREBELLY	NEWT	LIV	50	NO	HK	HK	T	W	C	C	4/17/08	4/12/08	I	NY	ERDA INCORPORATED
2009353332	CYOR	CYNOPS	ORIENTALIS	CHINESE DWARF	NEWT	LIV	100	NO	SG	SG	T	W	C	C	4/9/08	3/30/08	I	NY	INTERNATIONAL PET RESOURCES , LLC
2009353486	CYPY	CYNOPS	PYRRHOGASTER	JAPANESE FIREBELLY	NEWT	LIV	250	NO	HK	HK	T	W	C	C	4/3/08	3/30/08	I	NY	ERDA INCORPORATED
2009354164	CYPY	CYNOPS	PYRRHOGASTER	JAPANESE FIREBELLY	NEWT	LIV	120	NO	HK	HK	T	W	C	C	1/20/09	1/18/09	I	NY	Transship Discounts Ltd.
2009354556	CYOR	CYNOPS	ORIENTALIS	CHINESE DWARF	NEWT	LIV	100	NO	HK	HK	T	W	C	C	1/21/09	1/21/09	I	CH	AQUATICS INC
2009354771	NOVI	NOTOPHTHALMUS	VIRIDESCENS	EASTERN	NEWT	LIV	8	NO	US	CA	T	W	C	C	1/21/09	1/21/09	I	BN	Science Kit, Inc - Boreal
2009356360	TRIH	TRITURUS	HONGKONGENSIS	ALPINE	NEWT	LIV	250	NO	HK	HK	T	C	C	C	1/24/09	1/25/09	I	LA	AQUACO, INC.
2009356365	CYOR	CYNOPS	ORIENTALIS	CHINESE DWARF	NEWT	LIV	200	NO	CN	CN	T	C	C	C	1/24/09	1/25/09	I	LA	AQUA-NAUTIC SPECIALIST
2009356376	CYOR	CYNOPS	ORIENTALIS	CHINESE DWARF	NEWT	LIV	600	NO	HK	HK	T	C	C	C	1/24/09	1/25/09	I	LA	DOLPHIN INTERNATIONAL
2009356376	PACB	PACHYTRITON	BREVIPES	TSITOU	NEWT	LIV	100	NO	HK	HK	T	C	C	C	1/24/09	1/25/09	I	LA	DOLPHIN INTERNATIONAL
2009358501	NOVI	NOTOPHTHALMUS	VIRIDESCENS	EASTERN	NEWT	LIV	8	NO	US	CA	T	W	C	C	1/29/09	1/28/09	I	BN	Science Kit, Inc - Boreal
2009358504	NOVI	NOTOPHTHALMUS	VIRIDESCENS	EASTERN	NEWT	LIV	3	NO	US	CA	T	W	C	C	1/29/09	1/26/09	I	BN	Science Kit, Inc - Boreal
2009359377	CYOR	CYNOPS	ORIENTALIS	CHINESE DWARF	NEWT	LIV	1,250	NO	HK	HK	T	C	C	C	1/31/09	2/1/09	I	LA	DOLPHIN INTERNATIONAL
2009359392	PLAB	PACHYTRITON	LABIATUS	UNTERSTEIN'S	NEWT	LIV	100	NO	CN	CN	T	C	C	C	1/31/09	2/1/09	I	LA	AQUA-NAUTIC SPECIALIST
2009359484	PLWA	PLEURODELES	WALTL	IBERIAN RIBBED	NEWT	LIV	52	NO	CZ	CZ	T	C	C	C	2/2/09	1/21/09	I	MI	STRICTLY REPTILES, INC.
2009361048	TRIH	TRITURUS	HONGKONGENSIS	ALPINE	NEWT	LIV	1,600	NO	HK	HK	T	C	C	C	2/4/09	2/3/09	I	TP	5-D TROPICAL INC.
2009361139	NOVI	NOTOPHTHALMUS	VIRIDESCENS	EASTERN	NEWT	LIV	5	NO	US	CA	T	W	C	C	2/4/09	2/4/09	I	BN	Science Kit, Inc - Boreal
2009363217	CYPY	CYNOPS	PYRRHOGASTER	JAPANESE FIREBELLY	NEWT	LIV	90	NO	HK	HK	T	W	C	C	2/6/09	2/1/09	I	NY	Transship Discounts Ltd.
2009363278	CYOR	CYNOPS	ORIENTALIS	CHINESE DWARF	NEWT	LIV	350	NO	HK	HK	T	C	C	C	2/7/09	2/7/09	I	LA	DOLPHIN INTERNATIONAL
2009363353	TRIH	TRITURUS	HONGKONGENSIS	ALPINE	NEWT	LIV	675	NO	HK	HK	T	C	C	C	2/7/09	2/8/09	I	LA	AQUACO, INC.
2009363365	CYOR	CYNOPS	ORIENTALIS	CHINESE DWARF	NEWT	LIV	1,350	NO	HK	HK	T	C	C	C	2/7/09	2/8/09	I	LA	DOLPHIN INTERNATIONAL
2009363365	PACB	PACHYTRITON	BREVIPES	TSITOU	NEWT	LIV	500	NO	HK	HK	T	C	C	C	2/7/09	2/8/09	I	LA	DOLPHIN INTERNATIONAL
2009364089	CYPY	CYNOPS	PYRRHOGASTER	JAPANESE FIREBELLY	NEWT	LIV	165	NO	HK	HK	T	W	C	C	2/10/09	2/8/09	I	NY	Transship Discounts Ltd.
2009364397	NOVI	NOTOPHTHALMUS	VIRIDESCENS	EASTERN	NEWT	LIV	2	NO	US	CA	T	W	C	C	2/10/09	2/9/09	I	BN	Science Kit, Inc - Boreal
2009365101	NOVI	NOTOPHTHALMUS	VIRIDESCENS	EASTERN	NEWT	LIV	5	NO	US	CA	T	W	C	C	2/11/09	2/11/09	I	BN	Science Kit, Inc - Boreal
2009366562	TRIH	TRITURUS	HONGKONGENSIS	ALPINE	NEWT	LIV	50	NO	HK	HK	T	C	C	C	2/14/09	2/15/09	I	LA	AQUACO, INC.
2009366576	CYOR	CYNOPS	ORIENTALIS	CHINESE DWARF	NEWT	LIV	600	NO	HK	HK	T	C	C	C	2/15/09	2/15/09	I	LA	DOLPHIN INTERNATIONAL
2009366576	PACB	PACHYTRITON	BREVIPES	TSITOU	NEWT	LIV	100	NO	HK	HK	T	C	C	C	2/15/09	2/15/09	I	LA	DOLPHIN INTERNATIONAL
2009366599	PLAB	PACHYTRITON	LABIATUS	UNTERSTEIN'S	NEWT	LIV	100	NO	CN	CN	T	C	C	C	2/15/09	2/15/09	I	LA	AQUA-NAUTIC SPECIALIST

2009367552	PACB	PACHYTRITON	BREVIPES	TSITOU	NEWT	LIV	60	NO	HK	HK	T	W	C	C	2/17/09	2/15/09	I	NY	Transship Discounts Ltd.
2009368180	NOVI	NOTOPHTHALMUS	VIRIDESCENS	EASTERN	NEWT	LIV	11	NO	US	CA	T	W	C	C	2/18/09	2/18/09	I	BN	Science Kit, Inc - Boreal
2009368365	PDE?	PSEUDOEURYCEA	SPECIES	FALSE BROOK	SALAMANDER	LIV	10	NO	MX	MX	S	W	C	C	9/8/08	8/17/08	I	SF	MUSEUM OF VERTEBRATE ZOOLOGY
2009368746	CYOR	CYNOPS	ORIENTALIS	CHINESE DWARF	NEWT	LIV	200	NO	SG	SG	T	W	C	C	7/20/08	7/20/08	I	NY	INTERNATIONAL PET RESOURCES , LLC
2009369013	TRIH	TRITURUS	HONGKONGENSIS	ALPINE	NEWT	LIV	450	NO	HK	HK	T	C	C	C	3/26/07	3/26/07	I	NY	TRI-COUNTY TROPICALS, INC.
2009369136	CYOR	CYNOPS	ORIENTALIS	CHINESE DWARF	NEWT	LIV	2,500	NO	CN	CN	T	W	C	C	2/20/09	2/20/09	I	LA	Z IMPORTS INC.
2009369159	CYOR	CYNOPS	ORIENTALIS	CHINESE DWARF	NEWT	LIV	200	NO	SG	SG	T	W	C	C	7/6/08	7/5/08	I	NY	INTERNATIONAL PET RESOURCES , LLC
2009369602	CYOR	CYNOPS	ORIENTALIS	CHINESE DWARF	NEWT	LIV	600	NO	HK	HK	T	C	C	C	2/21/09	2/21/09	I	LA	DOLPHIN INTERNATIONAL
2009369602	PACB	PACHYTRITON	BREVIPES	TSITOU	NEWT	LIV	100	NO	HK	HK	T	C	C	C	2/21/09	2/21/09	I	LA	DOLPHIN INTERNATIONAL
2009369705	CYOR	CYNOPS	ORIENTALIS	CHINESE DWARF	NEWT	LIV	1,100	NO	HK	HK	T	C	C	C	2/22/09	2/22/09	I	LA	DOLPHIN INTERNATIONAL
2009369705	PACB	PACHYTRITON	BREVIPES	TSITOU	NEWT	LIV	100	NO	HK	HK	T	C	C	C	2/22/09	2/22/09	I	LA	DOLPHIN INTERNATIONAL
2009370023	PACB	PACHYTRITON	BREVIPES	TSITOU	NEWT	LIV	30	NO	HK	HK	T	W	C	C	2/23/09	2/22/09	I	NY	Transship Discounts Ltd.
2009370451	CYPY	CYNOPS	PYRRHOGASTER	JAPANESE FIREBELLY	NEWT	LIV	960	NO	HK	HK	T	W	C	C	6/21/06	6/17/06	I	NY	ERDA INCORPORATED
2009370910	NOVI	NOTOPHTHALMUS	VIRIDESCENS	EASTERN	NEWT	LIV	4	NO	US	CA	T	W	C	C	2/24/09	2/23/09	I	BN	Science Kit, Inc - Boreal
2009371186	CYPY	CYNOPS	PYRRHOGASTER	JAPANESE FIREBELLY	NEWT	LIV	125	NO	CN	CN	T	C	C	C	5/5/06	4/30/06	I	NY	FISH MART INCORPORATED
2009371630	TRIH	TRITURUS	HONGKONGENSIS	ALPINE	NEWT	LIV	1,600	NO	HK	HK	T	C	C	C	2/25/09	2/25/09	I	TP	5-D TROPICAL INC.
2009371857	NOVI	NOTOPHTHALMUS	VIRIDESCENS	EASTERN	NEWT	LIV	2	NO	US	CA	T	W	C	C	2/26/09	2/25/09	I	BN	Science Kit, Inc - Boreal
2009372073	CYPY	CYNOPS	PYRRHOGASTER	JAPANESE FIREBELLY	NEWT	LIV	50	NO	HK	HK	T	W	C	C	7/11/07	7/8/07	I	NY	TRANSSHIP DISCOUNTS LIMITED
2009373145	TRIH	TRITURUS	HONGKONGENSIS	ALPINE	NEWT	LIV	150	NO	HK	HK	T	C	C	C	2/28/09	3/1/09	I	LA	AQUACO, INC.
2009373155	CYOR	CYNOPS	ORIENTALIS	CHINESE DWARF	NEWT	LIV	245	NO	CN	CN	T	C	C	C	2/28/09	3/1/09	I	LA	AQUA-NAUTIC SPECIALIST
2009373174	CYOR	CYNOPS	ORIENTALIS	CHINESE DWARF	NEWT	LIV	560	NO	HK	HK	T	C	C	C	3/1/09	3/1/09	I	LA	DOLPHIN INTERNATIONAL
2009373174	PACB	PACHYTRITON	BREVIPES	TSITOU	NEWT	LIV	220	NO	HK	HK	T	C	C	C	3/1/09	3/1/09	I	LA	DOLPHIN INTERNATIONAL
2009373561	NOVI	NOTOPHTHALMUS	VIRIDESCENS	EASTERN	NEWT	LIV	2	NO	US	CA	T	W	C	C	3/2/09	3/2/09	I	BN	Science Kit, Inc - Boreal
2009374183	CYPY	CYNOPS	PYRRHOGASTER	JAPANESE FIREBELLY	NEWT	LIV	105	NO	HK	HK	T	W	C	C	3/3/09	3/1/09	I	NY	Transship Discounts Ltd.
2009377085	CYOR	CYNOPS	ORIENTALIS	CHINESE DWARF	NEWT	LIV	2,500	NO	CN	CN	T	W	C	C	3/6/09	3/6/09	I	LA	Z IMPORTS INC.
2009377311	NOVI	NOTOPHTHALMUS	VIRIDESCENS	EASTERN	NEWT	LIV	3	NO	US	CA	T	W	C	C	3/4/09	3/4/09	I	BN	Science Kit, Inc - Boreal
2009377618	CYOR	CYNOPS	ORIENTALIS	CHINESE DWARF	NEWT	LIV	670	NO	HK	HK	T	C	C	C	3/7/09	3/8/09	I	LA	DOLPHIN INTERNATIONAL
2009377618	PACB	PACHYTRITON	BREVIPES	TSITOU	NEWT	LIV	440	NO	HK	HK	T	C	C	C	3/7/09	3/8/09	I	LA	DOLPHIN INTERNATIONAL
2009377705	CYOR	CYNOPS	ORIENTALIS	CHINESE DWARF	NEWT	LIV	100	NO	SG	SG	T	C	C	C	9/7/08	9/7/08	I	NY	INTERNATIONAL PET RESOURCES , LLC
2009379010	NOVI	NOTOPHTHALMUS	VIRIDESCENS	EASTERN	NEWT	LIV	1	NO	US	CA	T	W	C	C	3/9/09	3/9/09	I	BN	Science Kit, Inc - Boreal
2009379264	CYOR	CYNOPS	ORIENTALIS	CHINESE DWARF	NEWT	LIV	200	NO	SG	SG	T	W	C	C	11/3/08	6/1/08	I	NY	INTERNATIONAL PET RESOURCES , LLC
2009379857	NOVI	NOTOPHTHALMUS	VIRIDESCENS	EASTERN	NEWT	LIV	1	NO	US	CA	T	W	C	C	3/11/09	3/11/09	I	BN	Science Kit, Inc - Boreal
2009380246	NEKA	NEURERGUS	KAISERI	LURISTAN	NEWT	LIV	230	NO	UA	UA	T	C	C	C	3/12/09	3/11/09	I	MI	EMERALD CORAL & REPTILE
2009380758	CYPY	CYNOPS	PYRRHOGASTER	JAPANESE FIREBELLY	NEWT	LIV	60	NO	HK	HK	T	W	C	C	3/13/09	3/8/09	I	NY	Transship Discounts Ltd.
2009380758	PACB	PACHYTRITON	BREVIPES	TSITOU	NEWT	LIV	30	NO	HK	HK	T	W	C	C	3/13/09	3/8/09	I	NY	Transship Discounts Ltd.
2009381723	CYOR	CYNOPS	ORIENTALIS	CHINESE DWARF	NEWT	LIV	950	NO	HK	HK	T	C	C	C	3/14/09	3/14/09	I	LA	DOLPHIN INTERNATIONAL
2009381788	TRIH	TRITURUS	HONGKONGENSIS	ALPINE	NEWT	LIV	250	NO	HK	HK	T	C	C	C	3/14/09	3/15/09	I	LA	AQUACO, INC.
2009381830	CYOR	CYNOPS	ORIENTALIS	CHINESE DWARF	NEWT	LIV	840	NO	HK	HK	T	C	C	C	3/14/09	3/15/09	I	LA	DOLPHIN INTERNATIONAL
2009381830	PACB	PACHYTRITON	BREVIPES	TSITOU	NEWT	LIV	110	NO	HK	HK	T	C	C	C	3/14/09	3/15/09	I	LA	DOLPHIN INTERNATIONAL
2009381837	CYOR	CYNOPS	ORIENTALIS	CHINESE DWARF	NEWT	LIV	280	NO	HK	HK	T	C	C	C	3/14/09	3/15/09	I	LA	DOLPHIN INTERNATIONAL
2009381934	CYPY	CYNOPS	PYRRHOGASTER	JAPANESE FIREBELLY	NEWT	LIV	160	NO	HK	HK	T	W	C	C	7/8/08	6/28/08	I	NY	ERDA INCORPORATED
2009382128	CYPY	CYNOPS	PYRRHOGASTER	JAPANESE FIREBELLY	NEWT	LIV	300	NO	HK	HK	T	W	C	C	9/30/08	9/27/08	I	NY	ERDA INCORPORATED
2009382340	CYPY	CYNOPS	PYRRHOGASTER	JAPANESE FIREBELLY	NEWT	LIV	300	NO	HK	HK	T	W	C	C	8/25/08	8/23/08	I	NY	ERDA INCORPORATED
2009382446	CNP?	CYNOPS	SPECIES	FIREBELLY	NEWT	LIV	0	NO	CN	CN	T	W	C	C	10/6/08	9/28/08	I	NY	FISH MART INCORPORATED
2009382446	CNP?	CYNOPS	SPECIES	FIREBELLY	NEWT	LIV	0	NO	CN	CN	T	W	C	C	10/6/08	9/28/08	I	NY	FISH MART INCORPORATED
2009382475	CYPY	CYNOPS	PYRRHOGASTER	JAPANESE FIREBELLY	NEWT	LIV	210	NO	HK	HK	T	C	C	C	7/23/08	7/19/08	I	NY	ERDA INCORPORATED
2009382492	CYPY	CYNOPS	PYRRHOGASTER	JAPANESE FIREBELLY	NEWT	LIV	60	NO	HK	HK	T	C	C	C	8/18/08	8/16/08	I	NY	ERDA INCORPORATED
2009382509	CYOR	CYNOPS	ORIENTALIS	CHINESE DWARF	NEWT	LIV	100	NO	SG	SG	T	W	C	C	9/14/08	9/14/08	I	NY	INTERNATIONAL PET RESOURCES , LLC
2009382556	CYPY	CYNOPS	PYRRHOGASTER	JAPANESE FIREBELLY	NEWT	LIV	90	NO	HK	HK	T	W	C	C	8/11/08	8/9/08	I	NY	ERDA INCORPORATED
2009382711	CYPY	CYNOPS	PYRRHOGASTER	JAPANESE FIREBELLY	NEWT	LIV	180	NO	HK	HK	T	W	C	C	9/17/08	9/13/08	I	NY	ERDA INCORPORATED
2009382737	CYPY	CYNOPS	PYRRHOGASTER	JAPANESE FIREBELLY	NEWT	LIV	250	NO	HK	HK	T	W	C	C	10/14/08	10/12/08	I	NY	ERDA INCORPORATED
2009383034	CYOR	CYNOPS	ORIENTALIS	CHINESE DWARF	NEWT	LIV	100	NO	SG	SG	T	W	C	C	8/27/08	8/24/08	I	NY	INTERNATIONAL PET RESOURCES , LLC
2009383439	NOVI	NOTOPHTHALMUS	VIRIDESCENS	EASTERN	NEWT	LIV	1	NO	US	CA	T	W	C	C	3/16/09	3/16/09	I	BN	Science Kit, Inc - Boreal
2009383750	CYPY	CYNOPS	PYRRHOGASTER	JAPANESE FIREBELLY	NEWT	LIV	60	NO	HK	HK	T	W	C	C	10/21/08	10/19/08	I	NY	ERDA INCORPORATED
2009383769	CYPY	CYNOPS	PYRRHOGASTER	JAPANESE FIREBELLY	NEWT	LIV	25	NO	HK	HK	T	W	C	C	10/27/08	10/25/08	I	NY	ERDA INCORPORATED
2009383824	CYOR	CYNOPS	ORIENTALIS	CHINESE DWARF	NEWT	LIV	100	NO	SG	SG	T	W	C	C	8/31/08	8/31/08	I	NY	INTERNATIONAL PET RESOURCES , LLC
2009383978	CYOR	CYNOPS	ORIENTALIS	CHINESE DWARF	NEWT	LIV	100	NO	SG	SG	T	W	C	C	8/8/08	8/8/08	I	NY	INTERNATIONAL PET RESOURCES , LLC
2009384004	CYOR	CYNOPS	ORIENTALIS	CHINESE DWARF	NEWT	LIV	100	NO	SG	SG	T	W	C	C	10/17/08	10/12/08	I	NY	INTERNATIONAL PET RESOURCES , LLC
2009384141	CYOR	CYNOPS	ORIENTALIS	CHINESE DWARF	NEWT	LIV	100	NO	SG	SG	T	W	C	C	11/2/08	11/2/08	I	NY	INTERNATIONAL PET RESOURCES , LLC
2009385469	CYOR	CYNOPS	ORIENTALIS	CHINESE DWARF	NEWT	LIV	2,000	NO	CN	CN	T	W	C	C	3/20/09	3/20/09	I	LA	Z IMPORTS INC.

2009385828	CYOR	CYNOPS	ORIENTALIS	CHINESE DWARF	NEWT	LIV	110	NO	HK	HK	T	C	C	C	3/21/09	3/21/09	I	LA	DOLPHIN INTERNATIONAL
2009385910	TRIH	TRITURUS	HONGKONGENSIS	ALPINE	NEWT	LIV	50	NO	HK	HK	T	C	C	C	3/21/09	3/22/09	I	LA	AQUACO, INC.
2009385912	PLAB	PACHYTRITON	LABIATUS	UNTERSTEIN'S	NEWT	LIV	600	NO	CN	CN	T	C	C	C	3/21/09	3/22/09	I	LA	AQUA-NAUTIC SPECIALIST
2009385930	CYOR	CYNOPS	ORIENTALIS	CHINESE DWARF	NEWT	LIV	1,230	NO	HK	HK	T	C	C	C	3/22/09	3/22/09	I	LA	DOLPHIN INTERNATIONAL
2009385930	PACB	PACHYTRITON	BREVIPES	TSITOU	NEWT	LIV	110	NO	HK	HK	T	C	C	C	3/22/09	3/22/09	I	LA	DOLPHIN INTERNATIONAL
2009386237	CYOR	CYNOPS	ORIENTALIS	CHINESE DWARF	NEWT	LIV	300	NO	HK	HK	T	C	C	C	3/23/09	3/23/09	I	CH	AQUATICS INC
2009386555	NOVI	NOTOPHTHALMUS	VIRIDESCENS	EASTERN	NEWT	LIV	6	NO	US	CA	T	W	C	C	3/24/09	3/23/09	I	BN	Science Kit, Inc - Boreal
2009387482	CYPY	CYNOPS	PYRRHOGASTER	JAPANESE FIREBELLY	NEWT	LIV	90	NO	HK	HK	T	W	C	C	3/25/09	3/22/09	I	NY	Transship Discounts Ltd.
2009387890	NOVI	NOTOPHTHALMUS	VIRIDESCENS	EASTERN	NEWT	LIV	3	NO	US	CA	T	W	C	C	3/26/09	3/25/09	I	BN	Science Kit, Inc - Boreal
2009388008	TRIT	TRITURUS	SPECIES		NEWT	LIV	109	NO	DE	DE	T	C	C	C	3/18/09	3/18/09	I	DF	U.S. GLOBAL EXOTICS, INC.
2009388008	CYPY	CYNOPS	PYRRHOGASTER	JAPANESE FIREBELLY	NEWT	LIV	100	NO	JP	DE	T	C	C	C	3/18/09	3/18/09	I	DF	U.S. GLOBAL EXOTICS, INC.
2009388220	NEKA	NEURERGUS	KAISERI	LURISTAN	NEWT	LIV	165	NO	UA	UA	T	C	C	C	3/26/09	3/26/09	I	DF	U.S. GLOBAL EXOTICS, INC.
2009388603	CNP?	CYNOPS	SPECIES	FIREBELLY	NEWT	LIV	0	NO	CN	CN	T	W	C	C	8/7/08	8/3/08	I	NY	FISH MART INCORPORATED
2009388603	CNP?	CYNOPS	SPECIES	FIREBELLY	NEWT	LIV	0	NO	CN	CN	T	W	C	C	8/7/08	8/3/08	I	NY	FISH MART INCORPORATED
2009388620	CYPY	CYNOPS	PYRRHOGASTER	JAPANESE FIREBELLY	NEWT	LIV	150	NO	HK	HK	T	W	C	C	8/7/08	8/2/08	I	NY	ERDA INCORPORATED
2009388860	CYOR	CYNOPS	ORIENTALIS	CHINESE DWARF	NEWT	LIV	560	NO	HK	HK	T	C	C	C	3/28/09	3/28/09	I	LA	DOLPHIN INTERNATIONAL
2009388926	TRIH	TRITURUS	HONGKONGENSIS	ALPINE	NEWT	LIV	150	NO	HK	HK	T	C	C	C	3/28/09	3/29/09	I	LA	AQUACO, INC.
2009388929	CYOR	CYNOPS	ORIENTALIS	CHINESE DWARF	NEWT	LIV	1,120	NO	HK	HK	T	C	C	C	3/28/09	3/29/09	I	LA	DOLPHIN INTERNATIONAL
2009388943	PLAB	PACHYTRITON	LABIATUS	UNTERSTEIN'S	NEWT	LIV	290	NO	CN	CN	T	W	C	C	3/29/09	3/29/09	I	LA	RENAISSANCE AQUATICS INC
2009389639	CYPY	CYNOPS	PYRRHOGASTER	JAPANESE FIREBELLY	NEWT	LIV	105	NO	HK	HK	T	W	C	C	3/31/09	3/29/09	I	NY	Transship Discounts Ltd.
2009389798	CYOR	CYNOPS	ORIENTALIS	CHINESE DWARF	NEWT	LIV	100	NO	SG	SG	T	W	C	C	4/20/08	4/20/08	I	NY	INTERNATIONAL PET RESOURCES , LLC
2009389973	NOVI	NOTOPHTHALMUS	VIRIDESCENS	EASTERN	NEWT	LIV	4	NO	US	CA	T	W	C	C	3/31/09	3/30/09	I	BN	Science Kit, Inc - Boreal
2009390798	NOVI	NOTOPHTHALMUS	VIRIDESCENS	EASTERN	NEWT	LIV	2	NO	US	CA	T	W	C	C	4/1/09	4/1/09	I	BN	Science Kit, Inc - Boreal
2009390918	TRIH	TRITURUS	HONGKONGENSIS	ALPINE	NEWT	LIV	1,600	NO	HK	HK	T	C	C	C	4/1/09	4/1/09	I	TP	5-D TROPICAL INC.
2009391144	CYPY	CYNOPS	PYRRHOGASTER	JAPANESE FIREBELLY	NEWT	LIV	150	NO	HK	HK	T	W	C	C	6/11/08	6/7/08	I	NY	ERDA INCORPORATED
2009391446	CYPY	CYNOPS	PYRRHOGASTER	JAPANESE FIREBELLY	NEWT	LIV	60	NO	HK	HK	T	C	C	C	5/6/08	5/3/08	I	NY	ERDA INCORPORATED
2009391880	CYOR	CYNOPS	ORIENTALIS	CHINESE DWARF	NEWT	LIV	100	NO	SG	SG	T	W	C	C	2/3/08	2/3/08	I	NY	INTERNATIONAL PET RESOURCES , LLC
2009392391	CYOR	CYNOPS	ORIENTALIS	CHINESE DWARF	NEWT	LIV	280	NO	HK	HK	T	C	C	C	4/4/09	4/4/09	I	LA	DOLPHIN INTERNATIONAL
2009392391	PACB	PACHYTRITON	BREVIPES	TSITOU	NEWT	LIV	220	NO	HK	HK	T	C	C	C	4/4/09	4/4/09	I	LA	DOLPHIN INTERNATIONAL
2009392448	TRIH	TRITURUS	HONGKONGENSIS	ALPINE	NEWT	LIV	75	NO	HK	HK	T	C	C	C	4/4/09	4/5/09	I	LA	AQUACO, INC.
2009392461	CYOR	CYNOPS	ORIENTALIS	CHINESE DWARF	NEWT	LIV	1,120	NO	HK	HK	T	C	C	C	4/4/09	4/5/09	I	LA	DOLPHIN INTERNATIONAL
2009392469	PLAB	PACHYTRITON	LABIATUS	UNTERSTEIN'S	NEWT	LIV	100	NO	CN	CN	T	W	C	C	4/5/09	4/5/09	I	LA	RENAISSANCE AQUATICS INC
2009392690	CYPY	CYNOPS	PYRRHOGASTER	JAPANESE FIREBELLY	NEWT	LIV	180	NO	HK	HK	T	W	C	C	9/2/08	8/30/08	I	NY	ERDA INCORPORATED
2009392993	CNP?	CYNOPS	SPECIES	FIREBELLY	NEWT	LIV	200	NO	CN	CN	T	W	C	C	7/22/08	7/13/08	I	NY	FISH MART INCORPORATED
2009393170	NOVI	NOTOPHTHALMUS	VIRIDESCENS	EASTERN	NEWT	LIV	2	NO	US	CA	T	W	C	C	4/6/09	4/6/09	I	BN	Science Kit, Inc - Boreal
2009394541	NOVI	NOTOPHTHALMUS	VIRIDESCENS	EASTERN	NEWT	LIV	1	NO	US	CA	T	W	C	C	4/8/09	4/8/09	I	BN	Science Kit, Inc - Boreal
2009396153	CYOR	CYNOPS	ORIENTALIS	CHINESE DWARF	NEWT	LIV	220	NO	HK	HK	T	C	C	C	4/11/09	4/11/09	I	LA	DOLPHIN INTERNATIONAL
2009396214	TRIH	TRITURUS	HONGKONGENSIS	ALPINE	NEWT	LIV	1,300	NO	HK	HK	T	C	C	C	4/11/09	4/12/09	I	LA	AQUACO, INC.
2009396231	CYOR	CYNOPS	ORIENTALIS	CHINESE DWARF	NEWT	LIV	1,120	NO	HK	HK	T	C	C	C	4/11/09	4/12/09	I	LA	DOLPHIN INTERNATIONAL
2009396231	PACB	PACHYTRITON	BREVIPES	TSITOU	NEWT	LIV	110	NO	HK	HK	T	C	C	C	4/11/09	4/12/09	I	LA	DOLPHIN INTERNATIONAL
2009397393	NOVI	NOTOPHTHALMUS	VIRIDESCENS	EASTERN	NEWT	LIV	4	NO	US	CA	T	W	C	C	4/14/09	4/13/09	I	BN	Science Kit, Inc - Boreal
2009397871	CYPY	CYNOPS	PYRRHOGASTER	JAPANESE FIREBELLY	NEWT	LIV	60	NO	HK	HK	T	W	C	C	4/15/09	4/5/09	I	NY	Transship Discounts Ltd.
2009397871	PACB	PACHYTRITON	BREVIPES	TSITOU	NEWT	LIV	120	NO	HK	HK	T	W	C	C	4/15/09	4/5/09	I	NY	Transship Discounts Ltd.
2009398331	NOVI	NOTOPHTHALMUS	VIRIDESCENS	EASTERN	NEWT	LIV	3	NO	US	CA	T	W	C	C	4/15/09	4/15/09	I	BN	Science Kit, Inc - Boreal
2009398394	TRIH	TRITURUS	HONGKONGENSIS	ALPINE	NEWT	LIV	1,600	NO	HK	HK	T	C	C	C	4/15/09	4/15/09	I	TP	5-D TROPICAL INC.
2009400315	CYOR	CYNOPS	ORIENTALIS	CHINESE DWARF	NEWT	LIV	1,400	NO	HK	HK	T	C	C	C	4/19/09	4/19/09	I	LA	DOLPHIN INTERNATIONAL
2009400315	PACB	PACHYTRITON	BREVIPES	TSITOU	NEWT	LIV	110	NO	HK	HK	T	C	C	C	4/19/09	4/19/09	I	LA	DOLPHIN INTERNATIONAL
2009400333	TRIH	TRITURUS	HONGKONGENSIS	ALPINE	NEWT	LIV	200	NO	HK	HK	T	C	C	C	4/19/09	4/19/09	I	LA	AQUACO, INC.
2009400337	CYPY	CYNOPS	PYRRHOGASTER	JAPANESE FIREBELLY	NEWT	LIV	80	NO	SG	SG	T	W	C	C	4/19/09	4/19/09	I	LA	TROPICAL FISH CONSOLIDATORS
2009400346	PLAB	PACHYTRITON	LABIATUS	UNTERSTEIN'S	NEWT	LIV	100	NO	CN	CN	T	W	C	C	4/19/09	4/19/09	I	LA	RENAISSANCE AQUATICS INC
2009400678	NMAC	NECTURUS	MACULOSUS		MUDPUPPY	LIV	1,500	NO	CA	CA	T	W	C	C	4/20/09	4/20/09	I	BN	Science Kit, Inc - Boreal
2009401286	CYPY	CYNOPS	PYRRHOGASTER	JAPANESE FIREBELLY	NEWT	LIV	195	NO	HK	HK	T	W	C	C	4/21/09	4/19/09	I	NY	Transship Discounts Ltd.
2009403349	CYPY	CYNOPS	PYRRHOGASTER	JAPANESE FIREBELLY	NEWT	LIV	800	NO	CN	CN	T	C	C	C	4/25/09	4/25/09	I	AT	SUN PET LTD.
2009403362	TRIH	TRITURUS	HONGKONGENSIS	ALPINE	NEWT	LIV	100	NO	HK	HK	T	C	C	C	4/25/09	4/26/09	I	LA	AQUACO, INC.
2009403382	CYOR	CYNOPS	ORIENTALIS	CHINESE DWARF	NEWT	LIV	950	NO	HK	HK	T	C	C	C	4/25/09	4/26/09	I	LA	DOLPHIN INTERNATIONAL
2009403382	PACB	PACHYTRITON	BREVIPES	TSITOU	NEWT	LIV	220	NO	HK	HK	T	C	C	C	4/25/09	4/26/09	I	LA	DOLPHIN INTERNATIONAL
2009403747	NOVI	NOTOPHTHALMUS	VIRIDESCENS	EASTERN	NEWT	LIV	5	NO	US	CA	T	W	C	C	4/27/09	4/27/09	I	BN	Science Kit, Inc - Boreal
2009403749	CYOR	CYNOPS	ORIENTALIS	CHINESE DWARF	NEWT	LIV	350	NO	HK	HK	T	C	C	C	4/27/09	4/27/09	I	CH	AQUATICS INC
2009404049	CYPY	CYNOPS	PYRRHOGASTER	JAPANESE FIREBELLY	NEWT	LIV	165	NO	HK	HK	T	W	C	C	4/28/09	4/26/09	I	NY	Transship Discounts Ltd.
2009404991	NOVI	NOTOPHTHALMUS	VIRIDESCENS	EASTERN	NEWT	LIV	1	NO	US	CA	T	W	C	C	4/29/09	4/29/09	I	BN	Science Kit, Inc - Boreal

2009404991	AMBA	AMBYSTOMA	MACULATUM	SPOTTED	SALAMANDER	LIV	125	NO	CA	CA	T	W	C	C	4/29/09	4/29/09	I	BN	Science Kit, Inc - Boreal
2009405186	BOG?	BOLITOGLOSSA	SPECIES	MUSHROOMTONGUE	SALAMANDER	LIV	10	NO	GT	GT	S	W	C	C	4/29/09	4/30/09	I	SF	Museum of Vertebrate Zoology
2009405186	BOG?	BOLITOGLOSSA	SPECIES	MUSHROOMTONGUE	SALAMANDER	LIV	10	NO	GT	GT	S	W	C	C	4/29/09	4/30/09	I	SF	Museum of Vertebrate Zoology
2009406365	CYOR	CYNOPS	ORIENTALIS	CHINESE DWARF	NEWT	LIV	280	NO	HK	HK	T	C	C	C	5/2/09	5/2/09	I	LA	DOLPHIN INTERNATIONAL
2009406439	TRIH	TRITURUS	HONGKONGENSIS	ALPINE	NEWT	LIV	50	NO	HK	HK	T	C	C	C	5/2/09	5/3/09	I	LA	AQUACO, INC.
2009406448	CYOR	CYNOPS	ORIENTALIS	CHINESE DWARF	NEWT	LIV	300	NO	CN	CN	T	C	C	C	5/2/09	5/3/09	I	LA	RENAISSANCE AQUATICS INC
2009406838	NOVI	NOTOPHTHALMUS	VIRIDESCENS	EASTERN	NEWT	LIV	2	NO	US	CA	T	W	C	C	5/4/09	5/4/09	I	BN	Science Kit, Inc - Boreal
2009407017	CYPY	CYNOPS	PYRRHOGASTER	JAPANESE FIREBELLY	NEWT	LIV	105	NO	HK	HK	T	W	C	C	5/5/09	5/3/09	I	NY	Transship Discounts Ltd.
2009408033	NOVI	NOTOPHTHALMUS	VIRIDESCENS	EASTERN	NEWT	LIV	3	NO	US	CA	T	W	C	C	5/7/09	5/6/09	I	BN	Science Kit, Inc - Boreal
2009409036	CYOR	CYNOPS	ORIENTALIS	CHINESE DWARF	NEWT	LIV	500	NO	HK	HK	T	C	C	C	5/9/09	5/9/09	I	LA	DOLPHIN INTERNATIONAL
2009409036	PACB	PACHYTRITON	BREVIPES	TSITOU	NEWT	LIV	110	NO	HK	HK	T	C	C	C	5/9/09	5/9/09	I	LA	DOLPHIN INTERNATIONAL
2009409167	TRIH	TRITURUS	HONGKONGENSIS	ALPINE	NEWT	LIV	100	NO	HK	HK	T	C	C	C	5/9/09	5/10/09	I	LA	AQUACO, INC.
2009409170	CYOR	CYNOPS	ORIENTALIS	CHINESE DWARF	NEWT	LIV	2,380	NO	CN	CN	T	W	C	C	5/9/09	5/10/09	I	LA	RENAISSANCE AQUATICS INC
2009409184	CYOR	CYNOPS	ORIENTALIS	CHINESE DWARF	NEWT	LIV	950	NO	HK	HK	T	C	C	C	5/9/09	5/10/09	I	LA	DOLPHIN INTERNATIONAL
2009409184	PACB	PACHYTRITON	BREVIPES	TSITOU	NEWT	LIV	110	NO	HK	HK	T	C	C	C	5/9/09	5/10/09	I	LA	DOLPHIN INTERNATIONAL
2009409195	CYOR	CYNOPS	ORIENTALIS	CHINESE DWARF	NEWT	LIV	1,060	NO	HK	HK	T	C	C	C	5/10/09	5/3/09	I	LA	DOLPHIN INTERNATIONAL
2009409195	PACB	PACHYTRITON	BREVIPES	TSITOU	NEWT	LIV	110	NO	HK	HK	T	C	C	C	5/10/09	5/3/09	I	LA	DOLPHIN INTERNATIONAL
2009410479	CYPY	CYNOPS	PYRRHOGASTER	JAPANESE FIREBELLY	NEWT	LIV	210	NO	HK	HK	T	W	C	C	5/13/09	5/10/09	I	NY	Transship Discounts Ltd.
2009410702	NOVI	NOTOPHTHALMUS	VIRIDESCENS	EASTERN	NEWT	LIV	5	NO	US	CA	T	W	C	C	5/13/09	5/13/09	I	BN	Science Kit, Inc - Boreal
2009411563	CNP?	CYNOPS	SPECIES	FIREBELLY	NEWT	LIV	100	NO	SG	SG	T	W	C	C	11/23/08	11/23/08	I	NY	INTERNATIONAL PET RESOURCES , LLC
2009412237	CNP?	CYNOPS	SPECIES	FIREBELLY	NEWT	LIV	400	NO	CN	CN	T	C	C	C	5/16/09	5/16/09	I	AT	SUN PET LTD.
2009412328	TRIH	TRITURUS	HONGKONGENSIS	ALPINE	NEWT	LIV	25	NO	HK	HK	T	C	C	C	5/16/09	5/17/09	I	LA	AQUACO, INC.
2009412338	CYOR	CYNOPS	ORIENTALIS	CHINESE DWARF	NEWT	LIV	1,790	NO	HK	HK	T	C	C	C	5/16/09	5/17/09	I	LA	DOLPHIN INTERNATIONAL
2009412350	CYOR	CYNOPS	ORIENTALIS	CHINESE DWARF	NEWT	LIV	2,840	NO	CN	CN	T	W	C	C	5/17/09	5/17/09	I	LA	RENAISSANCE AQUATICS INC
2009414314	TRIH	TRITURUS	HONGKONGENSIS	ALPINE	NEWT	LIV	1,600	NO	HK	HK	T	C	C	C	5/20/09	5/20/09	I	TP	5-D TROPICAL INC.
2009415266	CYPY	CYNOPS	PYRRHOGASTER	JAPANESE FIREBELLY	NEWT	LIV	210	NO	HK	HK	T	W	C	C	5/22/09	5/17/09	I	NY	Transship Discounts Ltd.
2009415266	CYPY	CYNOPS	PYRRHOGASTER	JAPANESE FIREBELLY	NEWT	LIV	120	NO	HK	HK	T	W	C	C	5/22/09	5/17/09	I	NY	Transship Discounts Ltd.
2009415576	SASA	SALAMANDRA	SALAMANDRA	EUROPEAN FIRE	SALAMANDER	LIV	100	NO	UA	UA	T	W	C	C	5/22/09	5/20/09	I	MI	TWO AMIGOS IMPORT & EXPORT, INC.
2009415883	CNP?	CYNOPS	SPECIES	FIREBELLY	NEWT	LIV	400	NO	CN	CN	T	C	C	C	5/23/09	5/23/09	I	AT	SUN PET LTD.
2009415904	CYOR	CYNOPS	ORIENTALIS	CHINESE DWARF	NEWT	LIV	560	NO	HK	HK	T	C	C	C	5/23/09	5/23/09	I	LA	DOLPHIN INTERNATIONAL
2009415991	TRIH	TRITURUS	HONGKONGENSIS	ALPINE	NEWT	LIV	700	NO	HK	HK	T	C	C	C	5/23/09	5/24/09	I	LA	AQUACO, INC.
2009416002	CYOR	CYNOPS	ORIENTALIS	CHINESE DWARF	NEWT	LIV	600	NO	CN	CN	T	W	C	C	5/23/09	5/24/09	I	LA	RENAISSANCE AQUATICS INC
2009416002	TRIH	TRITURUS	HONGKONGENSIS	ALPINE	NEWT	LIV	150	NO	CN	CN	T	W	C	C	5/23/09	5/24/09	I	LA	RENAISSANCE AQUATICS INC
2009416032	CYOR	CYNOPS	ORIENTALIS	CHINESE DWARF	NEWT	LIV	280	NO	HK	HK	T	C	C	C	5/24/09	5/24/09	I	LA	DOLPHIN INTERNATIONAL
2009416032	PACB	PACHYTRITON	BREVIPES	TSITOU	NEWT	LIV	220	NO	HK	HK	T	C	C	C	5/24/09	5/24/09	I	LA	DOLPHIN INTERNATIONAL
2009417481	NOVI	NOTOPHTHALMUS	VIRIDESCENS	EASTERN	NEWT	LIV	6	NO	US	CA	T	W	C	C	5/27/09	5/27/09	I	BN	Science Kit, Inc - Boreal
2009417647	TRIH	TRITURUS	HONGKONGENSIS	ALPINE	NEWT	LIV	1,600	NO	HK	HK	T	C	C	C	5/27/09	5/28/09	I	TP	5-D TROPICAL INC.
2009417672	SASA	SALAMANDRA	SALAMANDRA	EUROPEAN FIRE	SALAMANDER	LIV	8	NO	DE	DE	T	C	C	C	4/20/09	4/20/09	I	PT	NAME REMOVED
2009418545	CYOR	CYNOPS	ORIENTALIS	CHINESE DWARF	NEWT	LIV	2,500	NO	CN	CN	T	W	C	C	5/29/09	5/29/09	I	LA	Z IMPORTS INC.
2009419024	CYOR	CYNOPS	ORIENTALIS	CHINESE DWARF	NEWT	LIV	110	NO	HK	HK	T	C	C	C	5/30/09	5/30/09	I	LA	DOLPHIN INTERNATIONAL
2009419100	CYOR	CYNOPS	ORIENTALIS	CHINESE DWARF	NEWT	LIV	950	NO	HK	HK	T	C	C	C	5/31/09	5/31/09	I	LA	DOLPHIN INTERNATIONAL
2009419105	TRIH	TRITURUS	HONGKONGENSIS	ALPINE	NEWT	LIV	100	NO	HK	HK	T	C	C	C	5/31/09	5/31/09	I	LA	AQUACO, INC.
2009420327	NOVI	NOTOPHTHALMUS	VIRIDESCENS	EASTERN	NEWT	LIV	3	NO	US	CA	T	W	C	C	6/3/09	6/1/09	I	BN	Science Kit, Inc - Boreal
2009420828	NOVI	NOTOPHTHALMUS	VIRIDESCENS	EASTERN	NEWT	LIV	4	NO	US	CA	T	W	C	C	6/4/09	6/3/09	I	BN	Science Kit, Inc - Boreal
2009420919	CYPY	CYNOPS	PYRRHOGASTER	JAPANESE FIREBELLY	NEWT	LIV	165	NO	HK	HK	T	W	C	C	6/4/09	5/31/09	I	NY	Transship Discounts Ltd.
2009421249	CYPY	CYNOPS	PYRRHOGASTER	JAPANESE FIREBELLY	NEWT	LIV	135	NO	HK	HK	T	W	C	C	12/11/08	12/7/08	I	NY	ERDA INCORPORATED
2009421321	SASA	SALAMANDRA	SALAMANDRA	EUROPEAN FIRE	SALAMANDER	LIV	16	NO	DE	DE	T	C	C	C	6/4/09	6/4/09	I	DF	U.S. GLOBAL EXOTICS, INC.
2009421321	NRR?	NEURERGUS	SPECIES		NEWT	LIV	30	NO	DE	DE	T	C	C	C	6/4/09	6/4/09	I	DF	U.S. GLOBAL EXOTICS, INC.
2009421321	AMBY	AMBYSTOMA	SPECIES	MOLE	SALAMANDER	LIV	10	NO	DE	DE	T	C	C	C	6/4/09	6/4/09	I	DF	U.S. GLOBAL EXOTICS, INC.
2009422106	CYOR	CYNOPS	ORIENTALIS	CHINESE DWARF	NEWT	LIV	110	NO	HK	HK	T	C	C	C	6/6/09	6/6/09	I	LA	DOLPHIN INTERNATIONAL
2009422185	TRIH	TRITURUS	HONGKONGENSIS	ALPINE	NEWT	LIV	250	NO	HK	HK	T	C	C	C	6/6/09	6/7/09	I	LA	AQUACO, INC.
2009422209	CYOR	CYNOPS	ORIENTALIS	CHINESE DWARF	NEWT	LIV	280	NO	HK	HK	T	C	C	C	6/6/09	6/7/09	I	LA	DOLPHIN INTERNATIONAL
2009422209	PACB	PACHYTRITON	BREVIPES	TSITOU	NEWT	LIV	220	NO	HK	HK	T	C	C	C	6/6/09	6/7/09	I	LA	DOLPHIN INTERNATIONAL
2009422219	TRIT	TRITURUS	SPECIES		NEWT	LIV	925	NO	CN	CN	T	W	C	C	6/7/09	6/7/09	I	LA	RENAISSANCE AQUATICS INC
2009422661	CYPY	CYNOPS	PYRRHOGASTER	JAPANESE FIREBELLY	NEWT	LIV	210	NO	HK	HK	T	W	C	C	6/8/09	6/8/09	I	NY	Transship Discounts Ltd.
2009423711	NOVI	NOTOPHTHALMUS	VIRIDESCENS	EASTERN	NEWT	LIV	1	NO	US	CA	T	W	C	C	6/8/09	6/8/09	I	BN	Science Kit, Inc - Boreal
2009423729	TYL?	TYLOTOTRITON	SPECIES	CROCODILE	NEWT	LIV	365	NO	CN	HK	T	W	C	C	6/10/09	6/10/09	I	DF	U.S. GLOBAL EXOTICS, INC.
2009424481	NOVI	NOTOPHTHALMUS	VIRIDESCENS	EASTERN	NEWT	LIV	1	NO	US	CA	T	W	C	C	6/11/09	6/10/09	I	BN	Science Kit, Inc - Boreal
2009425374	CYOR	CYNOPS	ORIENTALIS	CHINESE DWARF	NEWT	LIV	390	NO	HK	HK	T	C	C	C	6/13/09	6/13/09	I	LA	DOLPHIN INTERNATIONAL
2009425479	CYOR	CYNOPS	ORIENTALIS	CHINESE DWARF	NEWT	LIV	1,060	NO	HK	HK	T	C	C	C	6/13/09	6/14/09	I	LA	DOLPHIN INTERNATIONAL

2009425562	BODO	BOLITOGLOSSA	DOFLEINI	ALTA VERAPAZ	SALAMANDER	LIV	3	NO	GT	GT	T	W	C	C	6/15/09	6/14/09	I	MI	D.H.A.R. TROPICALS LLC
2009425872	CYOR	CYNOPS	ORIENTALIS	CHINESE DWARF	NEWT	LIV	300	NO	HK	HK	T	W	C	C	6/15/09	6/15/09	I	CH	AQUATICS INC
2009425901	NOVI	NOTOPHTHALMUS	VIRIDESCENS	EASTERN	NEWT	LIV	1	NO	US	CA	T	W	C	C	6/15/09	6/15/09	I	BN	VWR EDUCATION, LLC
2009427475	CYPY	CYNOPS	PYRRHOGASTER	JAPANESE FIREBELLY	NEWT	LIV	60	NO	HK	HK	T	W	C	C	6/17/09	6/14/09	I	NY	Transship Discounts Ltd.
2009429067	CYOR	CYNOPS	ORIENTALIS	CHINESE DWARF	NEWT	LIV	280	NO	HK	HK	T	C	C	C	6/20/09	6/20/09	I	LA	DOLPHIN INTERNATIONAL
2009429123	TRIH	TRITURUS	HONGKONGENSIS	ALPINE	NEWT	LIV	200	NO	HK	HK	T	C	C	C	6/20/09	6/21/09	I	LA	AQUACO, INC.
2009429139	CYOR	CYNOPS	ORIENTALIS	CHINESE DWARF	NEWT	LIV	390	NO	HK	HK	T	C	C	C	6/21/09	6/21/09	I	LA	DOLPHIN INTERNATIONAL
2009429139	PACB	PACHYTRITON	BREVIPES	TSITOU	NEWT	LIV	110	NO	HK	HK	T	C	C	C	6/21/09	6/21/09	I	LA	DOLPHIN INTERNATIONAL
2009430279	CYPY	CYNOPS	PYRRHOGASTER	JAPANESE FIREBELLY	NEWT	LIV	105	NO	HK	HK	T	W	C	C	6/23/09	6/21/09	I	NY	Transship Discounts Ltd.
2009430765	TRIH	TRITURUS	HONGKONGENSIS	ALPINE	NEWT	LIV	1,600	NO	HK	HK	T	C	C	C	6/24/09	6/24/09	I	TP	5-D TROPICAL INC.
2009432181	CNP?	CYNOPS	SPECIES	FIREBELLY	NEWT	LIV	400	NO	CN	CN	T	C	C	C	6/27/09	6/27/09	I	AT	SUN PET LTD.
2009432189	CYOR	CYNOPS	ORIENTALIS	CHINESE DWARF	NEWT	LIV	560	NO	HK	HK	T	C	C	C	6/27/09	6/27/09	I	LA	DOLPHIN INTERNATIONAL
2009432189	PACB	PACHYTRITON	BREVIPES	TSITOU	NEWT	LIV	110	NO	HK	HK	T	C	C	C	6/27/09	6/27/09	I	LA	DOLPHIN INTERNATIONAL
2009432290	CYOR	CYNOPS	ORIENTALIS	CHINESE DWARF	NEWT	LIV	1,400	NO	HK	HK	T	C	C	C	6/27/09	6/28/09	I	LA	DOLPHIN INTERNATIONAL
2009433809	PDE?	PSEUDOEURYCEA	SPECIES	FALSE BROOK	SALAMANDER	LIV	20	NO	MX	MX	S	W	C	C	7/1/09	7/1/09	I	LA	SAN FRANCISCO STATE UNIVERSITY
2009434986	CYOR	CYNOPS	ORIENTALIS	CHINESE DWARF	NEWT	LIV	3,000	NO	CN	CN	T	W	C	C	7/3/09	7/3/09	I	LA	Z IMPORTS INC.
2009435002	CYOR	CYNOPS	ORIENTALIS	CHINESE DWARF	NEWT	LIV	280	NO	HK	HK	T	C	C	C	7/4/09	7/4/09	I	LA	DOLPHIN INTERNATIONAL
2009435123	TRIH	TRITURUS	HONGKONGENSIS	ALPINE	NEWT	LIV	300	NO	HK	HK	T	C	C	C	7/4/09	7/5/09	I	LA	AQUACO, INC.
2009435162	CYOR	CYNOPS	ORIENTALIS	CHINESE DWARF	NEWT	LIV	840	NO	HK	HK	T	C	C	C	7/5/09	7/5/09	I	LA	DOLPHIN INTERNATIONAL
2009435162	PACB	PACHYTRITON	BREVIPES	TSITOU	NEWT	LIV	110	NO	HK	HK	T	C	C	C	7/5/09	7/5/09	I	LA	DOLPHIN INTERNATIONAL
2009436680	CYPY	CYNOPS	PYRRHOGASTER	JAPANESE FIREBELLY	NEWT	LIV	120	NO	HK	HK	T	W	C	C	7/7/09	7/5/09	I	NY	ERDA INC.
2009438674	CNP?	CYNOPS	SPECIES	FIREBELLY	NEWT	LIV	800	NO	CN	CN	T	C	C	C	7/11/09	7/11/09	I	AT	SUN PET LTD.
2009438694	CYOR	CYNOPS	ORIENTALIS	CHINESE DWARF	NEWT	LIV	280	NO	HK	HK	T	C	C	C	7/11/09	7/11/09	I	LA	DOLPHIN INTERNATIONAL
2009438779	TRIH	TRITURUS	HONGKONGENSIS	ALPINE	NEWT	LIV	300	NO	CN	CN	T	W	C	C	7/11/09	7/12/09	I	LA	RENAISSANCE AQUATICS INC
2009438797	TRIH	TRITURUS	HONGKONGENSIS	ALPINE	NEWT	LIV	200	NO	HK	HK	T	C	C	C	7/11/09	7/12/09	I	LA	AQUACO, INC.
2009438816	CYOR	CYNOPS	ORIENTALIS	CHINESE DWARF	NEWT	LIV	560	NO	HK	HK	T	C	C	C	7/11/09	7/12/09	I	LA	DOLPHIN INTERNATIONAL
2009438816	PACB	PACHYTRITON	BREVIPES	TSITOU	NEWT	LIV	110	NO	HK	HK	T	C	C	C	7/11/09	7/12/09	I	LA	DOLPHIN INTERNATIONAL
2009440720	TRIH	TRITURUS	HONGKONGENSIS	ALPINE	NEWT	LIV	1,600	NO	HK	HK	T	C	C	C	7/15/09	7/15/09	I	TP	5-D TROPICAL INC.
2009440976	NOVI	NOTOPHTHALMUS	VIRIDESCENS	EASTERN	NEWT	LIV	1	NO	US	CA	T	W	C	C	7/15/09	7/15/09	I	BN	Science Kit, Inc - Boreal
2009442637	CYOR	CYNOPS	ORIENTALIS	CHINESE DWARF	NEWT	LIV	280	NO	HK	HK	T	C	C	C	7/18/09	7/18/09	I	LA	DOLPHIN INTERNATIONAL
2009442697	CYOR	CYNOPS	ORIENTALIS	CHINESE DWARF	NEWT	LIV	560	NO	HK	HK	T	C	C	C	7/19/09	7/19/09	I	LA	DOLPHIN INTERNATIONAL
2009444615	TRIH	TRITURUS	HONGKONGENSIS	ALPINE	NEWT	LIV	290	NO	CN	CN	T	W	C	C	7/22/09	7/19/09	I	LA	RENAISSANCE AQUATICS INC
2009445316	CYPY	CYNOPS	PYRRHOGASTER	JAPANESE FIREBELLY	NEWT	LIV	315	NO	HK	HK	T	W	C	C	7/24/09	7/19/09	I	NY	Transship Discounts Ltd.
2009445316	PACB	PACHYTRITON	BREVIPES	TSITOU	NEWT	LIV	60	NO	HK	HK	T	W	C	C	7/24/09	7/19/09	I	NY	Transship Discounts Ltd.
2009445342	CYPY	CYNOPS	PYRRHOGASTER	JAPANESE FIREBELLY	NEWT	LIV	60	NO	HK	HK	T	W	C	C	7/24/09	7/19/09	I	NY	ERDA INC
2009445405	CYOR	CYNOPS	ORIENTALIS	CHINESE DWARF	NEWT	LIV	100	NO	SG	SG	T	W	C	C	2/8/09	2/8/09	I	NY	INTERNATIONAL PET RESOURCES , LLC
2009445428	CYOR	CYNOPS	ORIENTALIS	CHINESE DWARF	NEWT	LIV	100	NO	SG	SG	T	W	C	C	12/8/08	11/30/08	I	NY	INTERNATIONAL PET RESOURCES , LLC
2009445658	CYOR	CYNOPS	ORIENTALIS	CHINESE DWARF	NEWT	LIV	100	NO	SG	SG	T	W	C	C	12/21/08	12/21/08	I	NY	INTERNATIONAL PET RESOURCES , LLC
2009445953	CNP?	CYNOPS	SPECIES	FIREBELLY	NEWT	LIV	400	NO	CN	CN	T	C	C	C	7/25/09	7/25/09	I	AT	SUN PET LTD.
2009445976	CYOR	CYNOPS	ORIENTALIS	CHINESE DWARF	NEWT	LIV	280	NO	HK	HK	T	C	C	C	7/25/09	7/25/09	I	LA	DOLPHIN INTERNATIONAL
2009445976	PACB	PACHYTRITON	BREVIPES	TSITOU	NEWT	LIV	110	NO	HK	HK	T	C	C	C	7/25/09	7/25/09	I	LA	DOLPHIN INTERNATIONAL
2009446084	CYOR	CYNOPS	ORIENTALIS	CHINESE DWARF	NEWT	LIV	840	NO	HK	HK	T	C	C	C	7/25/09	7/26/09	I	LA	DOLPHIN INTERNATIONAL
2009446100	TRIH	TRITURUS	HONGKONGENSIS	ALPINE	NEWT	LIV	143	NO	CN	CN	T	W	C	C	7/26/09	7/26/09	I	LA	RENAISSANCE AQUATICS INC
2009446132	CYOR	CYNOPS	ORIENTALIS	CHINESE DWARF	NEWT	LIV	0	NO	CN	CN	T	W	C	C	1/9/09	1/4/09	I	NY	FISH MART INCORPORATED
2009446132	CNP?	CYNOPS	SPECIES	FIREBELLY	NEWT	LIV	0	NO	CN	CN	T	W	C	C	1/9/09	1/4/09	I	NY	FISH MART INCORPORATED
2009446201	CYPY	CYNOPS	PYRRHOGASTER	JAPANESE FIREBELLY	NEWT	LIV	360	NO	HK	HK	T	C	C	C	7/27/09	7/12/09	I	NY	ERDA INC
2009446348	CYOR	CYNOPS	ORIENTALIS	CHINESE DWARF	NEWT	LIV	350	NO	HK	HK	T	C	C	C	7/27/09	7/27/09	I	CH	AQUATICS INC
2009446834	TYL?	TYLOTOTRITON	SPECIES	CROCODILE	NEWT	LIV	458	NO	HK	HK	T	W	C	C	7/28/09	7/28/09	I	DF	U.S. GLOBAL EXOTICS, INC.
2009446834	SASA	SALAMANDRA	SALAMANDRA	EUROPEAN FIRE	SALAMANDER	LIV	20	NO	HK	HK	T	C	C	C	7/28/09	7/28/09	I	DF	U.S. GLOBAL EXOTICS, INC.
2009447420	CYPY	CYNOPS	PYRRHOGASTER	JAPANESE FIREBELLY	NEWT	LIV	210	NO	HK	HK	T	W	C	C	7/29/09	7/26/09	I	NY	Transship Discounts Ltd.
2009447420	PACB	PACHYTRITON	BREVIPES	TSITOU	NEWT	LIV	120	NO	HK	HK	T	W	C	C	7/29/09	7/26/09	I	NY	Transship Discounts Ltd.
2009448441	BODO	BOLITOGLOSSA	DOFLEINI	ALTA VERAPAZ	SALAMANDER	LIV	11	NO	GT	GT	T	W	C	C	7/30/09	7/30/09	I	MI	U.S. GLOBAL EXOTICS
2009449635	CYOR	CYNOPS	ORIENTALIS	CHINESE DWARF	NEWT	LIV	280	NO	HK	HK	T	C	C	C	7/31/09	8/1/09	I	LA	DOLPHIN INTERNATIONAL
2009449635	PACB	PACHYTRITON	BREVIPES	TSITOU	NEWT	LIV	110	NO	HK	HK	T	C	C	C	7/31/09	8/1/09	I	LA	DOLPHIN INTERNATIONAL
2009449664	TRIH	TRITURUS	HONGKONGENSIS	ALPINE	NEWT	LIV	1,425	NO	HK	HK	T	C	C	C	8/1/09	8/2/09	I	LA	AQUACO, INC.
2009449751	CYOR	CYNOPS	ORIENTALIS	CHINESE DWARF	NEWT	LIV	1,120	NO	HK	HK	T	C	C	C	8/2/09	8/2/09	I	LA	DOLPHIN INTERNATIONAL
2009449758	TRIT	TRITURUS	SPECIES		NEWT	LIV	100	NO	CN	CN	T	W	C	C	8/2/09	8/2/09	I	LA	RENAISSANCE AQUATICS INC
2009451695	CYPY	CYNOPS	PYRRHOGASTER	JAPANESE FIREBELLY	NEWT	LIV	390	NO	HK	HK	T	C	C	C	8/4/09	8/2/09	I	NY	ERDA INCORPORATED
2009452471	PLWA	PLEURODELES	WALTL	IBERIAN RIBBED	NEWT	LIV	100	NO	CZ	CZ	T	C	C	C	8/5/09	8/4/09	I	MI	STRICTLY REPTILES, INC.
2009452875	CYPY	CYNOPS	PYRRHOGASTER	JAPANESE FIREBELLY	NEWT	LIV	150	NO	HK	HK	T	W	C	C	1/27/08	1/18/08	I	NY	ERDA INCORPORATED

2009452971	CYPY	CYNOPS	PYRRHOGASTER	JAPANESE FIREBELLY	NEWT	LIV	240	NO	HK	HK	T	W	C	C	2/2/09	2/1/09	I	NY	ERDA INCORPORATED
2009452988	CYPY	CYNOPS	PYRRHOGASTER	JAPANESE FIREBELLY	NEWT	LIV	340	NO	HK	HK	T	W	C	C	2/10/09	2/7/09	I	NY	ERDA INCORPORATED
2009453283	TRIH	TRITURUS	HONGKONGENSIS	ALPINE	NEWT	LIV	1,600	NO	HK	HK	T	C	C	C	8/6/09	8/5/09	I	TP	5-D TROPICAL INC.
2009454928	CNP?	CYNOPS	SPECIES	FIREBELLY	NEWT	LIV	200	NO	CN	CN	T	C	C	C	8/8/09	8/8/09	I	AT	SUN PET LTD.
2009454962	CYOR	CYNOPS	ORIENTALIS	CHINESE DWARF	NEWT	LIV	280	NO	HK	HK	T	C	C	C	8/8/09	8/8/09	I	LA	DOLPHIN INTERNATIONAL
2009455000	TRIH	TRITURUS	HONGKONGENSIS	ALPINE	NEWT	LIV	254	NO	HK	HK	T	C	C	C	8/8/09	8/9/09	I	LA	AQUACO, INC.
2009455022	CYOR	CYNOPS	ORIENTALIS	CHINESE DWARF	NEWT	LIV	560	NO	HK	HK	T	C	C	C	8/8/09	8/9/09	I	LA	DOLPHIN INTERNATIONAL
2009455039	TRIT	TRITURUS	SPECIES		NEWT	LIV	2,060	NO	CN	CN	T	W	C	C	8/9/09	8/9/09	I	LA	RENAISSANCE AQUATICS INC
2009455832	CYPY	CYNOPS	PYRRHOGASTER	JAPANESE FIREBELLY	NEWT	LIV	105	NO	HK	HK	T	W	C	C	8/11/09	8/9/09	I	NY	Transship Discounts Ltd.
2009456323	NOVI	NOTOPHTHALMUS	VIRIDESCENS	EASTERN	NEWT	LIV	1	NO	US	CA	T	W	C	C	8/10/09	8/10/09	I	BN	Science Kit, Inc - Boreal
2009456545	CYPY	CYNOPS	PYRRHOGASTER	JAPANESE FIREBELLY	NEWT	LIV	220	NO	HK	HK	T	C	C	C	8/12/09	8/9/09	I	NY	ERDA INCORPORATED
2009457400	NOVI	NOTOPHTHALMUS	VIRIDESCENS	EASTERN	NEWT	LIV	1	NO	US	CA	T	W	C	C	8/12/09	8/12/09	I	BN	Science Kit, Inc - Boreal
2009458231	CYOR	CYNOPS	ORIENTALIS	CHINESE DWARF	NEWT	LIV	560	NO	HK	HK	T	C	C	C	8/15/09	8/16/09	I	LA	DOLPHIN INTERNATIONAL
2009458873	TRIT	TRITURUS	SPECIES		NEWT	LIV	100	NO	CN	SG	T	W	C	C	10/5/08	10/5/08	I	NY	FISH MART INCORPORATED
2009458879	CYOR	CYNOPS	ORIENTALIS	CHINESE DWARF	NEWT	LIV	100	NO	SG	SG	T	W	C	C	10/5/08	10/5/08	I	NY	INTERNATIONAL PET RESOURCES , LLC
2009458881	CYOR	CYNOPS	ORIENTALIS	CHINESE DWARF	NEWT	LIV	100	NO	SG	SG	T	W	C	C	12/14/08	12/14/08	I	NY	INTERNATIONAL PET RESOURCES , LLC
2009458904	CNP?	CYNOPS	SPECIES	FIREBELLY	NEWT	LIV	0	NO	CN	CN	T	W	C	C	12/16/08	12/7/08	I	NY	FISH MART INCORPORATED
2009458904	CNP?	CYNOPS	SPECIES	FIREBELLY	NEWT	LIV	0	NO	CN	CN	T	W	C	C	12/16/08	12/7/08	I	NY	FISH MART INCORPORATED
2009459387	TYVE	TYLOTOTRITON	VERRUCOSUS	CROCODILE	NEWT	LIV	40	NO	SG	SG	T	W	C	C	2/15/09	2/15/09	I	NY	INTERNATIONAL PET RESOURCES , LLC
2009460579	NOVI	NOTOPHTHALMUS	VIRIDESCENS	EASTERN	NEWT	LIV	3	NO	US	CA	T	W	C	C	8/19/09	8/19/09	I	BN	Science Kit, Inc - Boreal
2009460614	TRIH	TRITURUS	HONGKONGENSIS	ALPINE	NEWT	LIV	1,600	NO	HK	HK	T	C	C	C	8/19/09	8/19/09	I	TP	5-D TROPICAL INC.
2009461034	TYVE	TYLOTOTRITON	VERRUCOSUS	CROCODILE	NEWT	LIV	40	NO	SG	SG	T	W	C	C	1/18/09	1/18/09	I	NY	INTERNATIONAL PET RESOURCES , LLC
2009461068	CYOR	CYNOPS	ORIENTALIS	CHINESE DWARF	NEWT	LIV	200	NO	SG	SG	T	W	C	C	1/4/09	1/3/09	I	NY	INTERNATIONAL PET RESOURCES , LLC
2009461223	CYPY	CYNOPS	PYRRHOGASTER	JAPANESE FIREBELLY	NEWT	LIV	60	NO	HK	HK	T	W	C	C	2/23/09	2/22/09	I	NY	ERDA INCORPORATED
2009461971	CYOR	CYNOPS	ORIENTALIS	CHINESE DWARF	NEWT	LIV	280	NO	HK	HK	T	C	C	C	8/22/09	8/22/09	I	LA	DOLPHIN INTERNATIONAL
2009462073	CYOR	CYNOPS	ORIENTALIS	CHINESE DWARF	NEWT	LIV	560	NO	HK	HK	T	C	C	C	8/22/09	8/23/09	I	LA	DOLPHIN INTERNATIONAL
2009462075	CYOR	CYNOPS	ORIENTALIS	CHINESE DWARF	NEWT	LIV	840	NO	HK	HK	T	C	C	C	8/23/09	8/23/09	I	LA	DOLPHIN INTERNATIONAL
2009462178	CYOR	CYNOPS	ORIENTALIS	CHINESE DWARF	NEWT	LIV	120	NO	SG	SG	T	W	C	C	3/1/09	3/1/09	I	NY	INTERNATIONAL PET RESOURCES , LLC
2009462197	CYOR	CYNOPS	ORIENTALIS	CHINESE DWARF	NEWT	LIV	100	NO	SG	SG	T	W	C	C	10/26/08	10/26/08	I	NY	INTERNATIONAL PET RESOURCES , LLC
2009462315	CYOR	CYNOPS	ORIENTALIS	CHINESE DWARF	NEWT	LIV	100	NO	SG	SG	T	W	C	C	11/9/08	11/9/08	I	NY	INTERNATIONAL PET RESOURCES , LLC
2009462347	CNP?	CYNOPS	SPECIES	FIREBELLY	NEWT	LIV	0	NO	CN	CN	T	W	C	C	3/3/09	2/22/09	I	NY	FISH MART INCORPORATED
2009462347	CNP?	CYNOPS	SPECIES	FIREBELLY	NEWT	LIV	0	NO	CN	CN	T	W	C	C	3/3/09	2/22/09	I	NY	FISH MART INCORPORATED
2009462364	CYOR	CYNOPS	ORIENTALIS	CHINESE DWARF	NEWT	LIV	200	NO	SG	SG	T	W	C	C	3/9/09	2/22/09	I	NY	INTERNATIONAL PET RESOURCES , LLC
2009462422	TRIT	TRITURUS	SPECIES		NEWT	LIV	1,100	NO	CN	CN	T	W	C	C	8/24/09	8/23/09	I	LA	RENAISSANCE AQUATICS INC
2009462435	CYPY	CYNOPS	PYRRHOGASTER	JAPANESE FIREBELLY	NEWT	LIV	50	NO	HK	HK	T	W	C	C	3/11/09	3/7/09	I	NY	ERDA INCORPORATED
2009462660	NOVI	NOTOPHTHALMUS	VIRIDESCENS	EASTERN	NEWT	LIV	9	NO	US	CA	T	W	C	C	8/24/09	8/24/09	I	BN	Science Kit, Inc - Boreal
2009463567	CYPY	CYNOPS	PYRRHOGASTER	JAPANESE FIREBELLY	NEWT	LIV	360	NO	HK	HK	T	C	C	C	8/25/09	8/23/09	I	NY	ERDA INCORPORATED
2009463627	TRIH	TRITURUS	HONGKONGENSIS	ALPINE	NEWT	LIV	290	NO	CN	CN	T	W	C	C	8/25/09	8/16/09	I	LA	RENAISSANCE AQUATICS INC
2009463627	CNP?	CYNOPS	SPECIES	FIREBELLY	NEWT	LIV	900	NO	CN	CN	T	W	C	C	8/25/09	8/16/09	I	LA	RENAISSANCE AQUATICS INC
2009464062	NOVI	NOTOPHTHALMUS	VIRIDESCENS	EASTERN	NEWT	LIV	3	NO	US	CA	T	W	C	C	8/26/09	8/26/09	I	BN	Science Kit, Inc - Boreal
2009465287	PACB	PACHYTRITON	BREVIPES	TSITOU	NEWT	LIV	100	NO	HK	HK	T	C	C	C	8/29/09	8/29/09	I	LA	DOLPHIN INTERNATIONAL
2009465378	CYOR	CYNOPS	ORIENTALIS	CHINESE DWARF	NEWT	LIV	500	NO	HK	HK	T	C	C	C	8/29/09	8/30/09	I	LA	DOLPHIN INTERNATIONAL
2009465378	PACB	PACHYTRITON	BREVIPES	TSITOU	NEWT	LIV	100	NO	HK	HK	T	C	C	C	8/29/09	8/30/09	I	LA	DOLPHIN INTERNATIONAL
2009465557	CYOR	CYNOPS	ORIENTALIS	CHINESE DWARF	NEWT	LIV	350	NO	HK	HK	T	C	C	C	8/31/09	8/31/09	I	CH	AQUATICS INC
2009465794	NOVI	NOTOPHTHALMUS	VIRIDESCENS	EASTERN	NEWT	LIV	1	NO	US	CA	T	W	C	C	8/31/09	8/31/09	I	BN	Science Kit, Inc - Boreal
2009467010	NOVI	NOTOPHTHALMUS	VIRIDESCENS	EASTERN	NEWT	LIV	7	NO	US	CA	T	W	C	C	9/2/09	9/2/09	I	BN	Science Kit, Inc - Boreal
2009467029	TRIH	TRITURUS	HONGKONGENSIS	ALPINE	NEWT	LIV	1,600	NO	HK	HK	T	C	C	C	9/2/09	9/3/09	I	TP	5-D TROPICAL INC.
2009468773	PACB	PACHYTRITON	BREVIPES	TSITOU	NEWT	LIV	100	NO	HK	HK	T	C	C	C	9/5/09	9/5/09	I	LA	DOLPHIN INTERNATIONAL
2009468773	CYOR	CYNOPS	ORIENTALIS	CHINESE DWARF	NEWT	LIV	250	NO	HK	HK	T	C	C	C	9/5/09	9/5/09	I	LA	DOLPHIN INTERNATIONAL
2009468798	TRIH	TRITURUS	HONGKONGENSIS	ALPINE	NEWT	LIV	100	NO	HK	HK	T	C	C	C	9/5/09	9/6/09	I	LA	AQUACO, INC.
2009468814	CYOR	CYNOPS	ORIENTALIS	CHINESE DWARF	NEWT	LIV	500	NO	HK	HK	T	C	C	C	9/5/09	9/6/09	I	LA	DOLPHIN INTERNATIONAL
2009468824	TRIH	TRITURUS	HONGKONGENSIS	ALPINE	NEWT	LIV	100	NO	CN	CN	T	W	C	C	9/6/09	9/6/09	I	LA	RENAISSANCE AQUATICS INC
2009468945	CYPY	CYNOPS	PYRRHOGASTER	JAPANESE FIREBELLY	NEWT	LIV	105	NO	HK	HK	T	W	C	C	9/8/09	8/30/09	I	NY	Transship Discounts Ltd.
2009469342	CYPY	CYNOPS	PYRRHOGASTER	JAPANESE FIREBELLY	NEWT	LIV	140	NO	HK	HK	T	W	C	C	9/8/09	8/30/09	I	NY	ERDA INCORPORATED
2009470879	NOVI	NOTOPHTHALMUS	VIRIDESCENS	EASTERN	NEWT	LIV	7	NO	US	CA	T	W	C	C	9/9/08	9/9/09	I	BN	Science Kit, Inc - Boreal
2009470900	CYPY	CYNOPS	PYRRHOGASTER	JAPANESE FIREBELLY	NEWT	LIV	105	NO	HK	HK	T	W	C	C	9/9/09	9/6/09	I	NY	Transship Discounts Ltd.
2009472899	CYOR	CYNOPS	ORIENTALIS	CHINESE DWARF	NEWT	LIV	500	NO	HK	HK	T	C	C	C	9/12/09	9/12/09	I	LA	DOLPHIN INTERNATIONAL
2009472975	TRIH	TRITURUS	HONGKONGENSIS	ALPINE	NEWT	LIV	100	NO	HK	HK	T	C	C	C	9/12/09	9/13/09	I	LA	AQUACO, INC.
2009472980	CNP?	CYNOPS	SPECIES	FIREBELLY	NEWT	LIV	592	NO	CN	CN	T	C	C	C	9/12/09	9/13/09	I	LA	RENAISSANCE AQUATICS INC
2009472980	TRIH	TRITURUS	HONGKONGENSIS	ALPINE	NEWT	LIV	1,290	NO	CN	CN	T	C	C	C	9/12/09	9/13/09	I	LA	RENAISSANCE AQUATICS INC

2009472989	CYOR	CYNOPS	ORIENTALIS	CHINESE DWARF	NEWT	LIV	850	NO	HK	HK	T	C	C	C	9/12/09	9/13/09	I	LA	DOLPHIN INTERNATIONAL
2009473358	CYOR	CYNOPS	ORIENTALIS	CHINESE DWARF	NEWT	LIV	120	NO	SG	SG	T	C	C	C	3/31/09	3/15/09	I	NY	INTERNATIONAL PET RESOURCES , LLC
2009473378	CYPY	CYNOPS	PYRRHOGASTER	JAPANESE FIREBELLY	NEWT	LIV	70	NO	HK	HK	T	W	C	C	9/14/09	9/13/09	I	NY	ERDA INCORPORATED
2009473397	CYOR	CYNOPS	ORIENTALIS	CHINESE DWARF	NEWT	LIV	120	NO	SG	SG	T	C	C	C	3/20/09	3/15/09	I	NY	INTERNATIONAL PET RESOURCES , LLC
2009473471	CYOR	CYNOPS	ORIENTALIS	CHINESE DWARF	NEWT	LIV	120	NO	SG	SG	T	W	C	C	3/8/09	3/8/09	I	NY	INTERNATIONAL PET RESOURCES , LLC
2009473767	NOVI	NOTOPHTHALMUS	VIRIDESCENS	EASTERN	NEWT	LIV	5	NO	US	CA	T	W	C	C	9/15/09	9/14/09	I	BN	Science Kit, Inc - Boreal
2009475023	NOVI	NOTOPHTHALMUS	VIRIDESCENS	EASTERN	NEWT	LIV	2	NO	US	CA	T	W	C	C	9/16/09	9/16/09	I	BN	Science Kit, Inc - Boreal
2009475896	SASA	SALAMANDRA	SALAMANDRA	EUROPEAN FIRE	SALAMANDER	LIV	10	NO	DE	DE	T	C	C	C	9/18/09	9/17/09	I	MI	GLADES HERP FARM
2009476041	PLWA	PLEURODELES	WALTL	IBERIAN RIBBED	NEWT	LIV	6	NO	RU	CA	T	F	C	C	9/16/09	9/16/09	I	BL	STRICTLY REPTILE
2009476327	CNP?	CYNOPS	SPECIES	FIREBELLY	NEWT	LIV	400	NO	CN	CN	T	C	C	C	9/19/09	9/19/09	I	AT	SUN PET LTD.
2009476410	TRIH	TRITURUS	HONGKONGENSIS	ALPINE	NEWT	LIV	100	NO	HK	HK	T	C	C	C	9/19/09	9/20/09	I	LA	AQUACO, INC.
2009476417	CNP?	CYNOPS	SPECIES	FIREBELLY	NEWT	LIV	1,340	NO	CN	CN	T	W	C	C	9/19/09	9/20/09	I	LA	RENAISSANCE AQUATICS INC
2009476417	TRIH	TRITURUS	HONGKONGENSIS	ALPINE	NEWT	LIV	200	NO	CN	CN	T	W	C	C	9/19/09	9/20/09	I	LA	RENAISSANCE AQUATICS INC
2009476426	PACB	PACHYTRITON	BREVIPES	TSITOU	NEWT	LIV	200	NO	HK	HK	T	C	C	C	9/20/09	9/20/09	I	LA	DOLPHIN INTERNATIONAL
2009476934	CYPY	CYNOPS	PYRRHOGASTER	JAPANESE FIREBELLY	NEWT	LIV	105	NO	HK	HK	T	W	C	C	9/21/09	9/20/09	I	NY	Transship Discounts Ltd.
2009477061	CYPY	CYNOPS	PYRRHOGASTER	JAPANESE FIREBELLY	NEWT	LIV	140	NO	HK	HK	T	W	C	C	9/21/09	9/20/09	I	NY	ERDA INCORPORATED
2009477291	NOVI	NOTOPHTHALMUS	VIRIDESCENS	EASTERN	NEWT	LIV	2	NO	US	CA	T	W	C	C	9/21/09	9/21/09	I	BN	Science Kit, Inc - Boreal
2009478460	NOVI	NOTOPHTHALMUS	VIRIDESCENS	EASTERN	NEWT	LIV	3	NO	US	CA	T	W	C	C	9/23/09	9/23/09	I	BN	Science Kit, Inc - Boreal
2009478930	CYPY	CYNOPS	PYRRHOGASTER	JAPANESE FIREBELLY	NEWT	LIV	1	NO	CA	CA	P	C	C	C	7/20/09	7/20/09	I	SW	Exemptions 6 and 7(C)
2009480234	CYOR	CYNOPS	ORIENTALIS	CHINESE DWARF	NEWT	LIV	250	NO	HK	HK	T	C	C	C	9/26/09	9/26/09	I	LA	DOLPHIN INTERNATIONAL
2009480304	TRIH	TRITURUS	HONGKONGENSIS	ALPINE	NEWT	LIV	50	NO	HK	HK	T	C	C	C	9/26/09	9/27/09	I	LA	AQUACO, INC.
2009480322	CYOR	CYNOPS	ORIENTALIS	CHINESE DWARF	NEWT	LIV	1,450	NO	HK	HK	T	C	C	C	9/27/09	9/27/09	I	LA	DOLPHIN INTERNATIONAL
2009480322	PACB	PACHYTRITON	BREVIPES	TSITOU	NEWT	LIV	200	NO	HK	HK	T	C	C	C	9/27/09	9/27/09	I	LA	DOLPHIN INTERNATIONAL
2009480943	CYPY	CYNOPS	PYRRHOGASTER	JAPANESE FIREBELLY	NEWT	LIV	420	NO	HK	HK	T	W	C	C	9/28/09	9/27/09	I	NY	Transship Discounts Ltd.
2009481026	CYPY	CYNOPS	PYRRHOGASTER	JAPANESE FIREBELLY	NEWT	LIV	70	NO	HK	HK	T	W	C	C	9/28/09	9/27/09	I	NY	ERDA INCORPORATED
2009481842	NEW?	PACHYTRITON	SPECIES	CHINESE	NEWT	LIV	300	NO	CN	CN	T	W	C	C	9/30/09	9/27/09	I	NY	FISH MART INCORPORATED
2009482124	TRIH	TRITURUS	HONGKONGENSIS	ALPINE	NEWT	LIV	1,600	NO	HK	HK	T	C	C	C	9/30/09	9/30/09	I	TP	5-D TROPICAL INC.
2009482190	NOVI	NOTOPHTHALMUS	VIRIDESCENS	EASTERN	NEWT	LIV	1	NO	US	CA	T	W	C	C	9/30/09	9/30/09	I	BN	Science Kit, Inc - Boreal
2009483548	CNP?	CYNOPS	SPECIES	FIREBELLY	NEWT	LIV	800	NO	CN	CN	T	C	C	C	10/3/09	10/3/09	I	AT	SUN PET LTD.
2009483567	CYOR	CYNOPS	ORIENTALIS	CHINESE DWARF	NEWT	LIV	250	NO	HK	HK	T	C	C	C	10/3/09	10/3/09	I	LA	DOLPHIN INTERNATIONAL
2009483656	CYOR	CYNOPS	ORIENTALIS	CHINESE DWARF	NEWT	LIV	250	NO	HK	HK	T	C	C	C	10/4/09	10/4/09	I	LA	DOLPHIN INTERNATIONAL
2009483664	CNP?	CYNOPS	SPECIES	FIREBELLY	NEWT	LIV	300	NO	CN	CN	T	W	C	C	10/4/09	10/4/09	I	LA	RENAISSANCE AQUATICS INC
2009483783	CYOR	CYNOPS	ORIENTALIS	CHINESE DWARF	NEWT	LIV	450	NO	HK	HK	T	C	C	C	10/5/09	10/5/09	I	CH	AQUATICS INC
2009484060	NOVI	NOTOPHTHALMUS	VIRIDESCENS	EASTERN	NEWT	LIV	1	NO	US	CA	T	W	C	C	10/5/09	10/5/09	I	BN	Science Kit, Inc - Boreal
2009484169	CYPY	CYNOPS	PYRRHOGASTER	JAPANESE FIREBELLY	NEWT	LIV	120	NO	HK	HK	T	W	C	C	10/5/09	10/4/09	I	NY	Transship Discounts Ltd.
2009484443	CYPY	CYNOPS	PYRRHOGASTER	JAPANESE FIREBELLY	NEWT	LIV	70	NO	HK	HK	T	W	C	C	10/6/09	10/4/09	I	NY	ERDA INCORPORATED
2009485796	NOVI	NOTOPHTHALMUS	VIRIDESCENS	EASTERN	NEWT	LIV	3	NO	US	CA	T	W	C	C	10/8/09	10/7/09	I	BN	Science Kit, Inc - Boreal
2009486596	PACB	PACHYTRITON	BREVIPES	TSITOU	NEWT	LIV	100	NO	HK	HK	T	C	C	C	10/10/09	10/10/09	I	LA	DOLPHIN INTERNATIONAL
2009486672	TRIH	TRITURUS	HONGKONGENSIS	ALPINE	NEWT	LIV	300	NO	HK	HK	T	C	C	C	10/10/09	10/11/09	I	LA	AQUACO, INC.
2009486703	TRIT	TRITURUS	SPECIES		NEWT	LIV	100	NO	CN	CN	T	W	C	C	10/11/09	10/11/09	I	LA	RENAISSANCE AQUATICS INC
2009486703	TRIH	TRITURUS	HONGKONGENSIS	ALPINE	NEWT	LIV	1,040	NO	CN	CN	T	W	C	C	10/11/09	10/11/09	I	LA	RENAISSANCE AQUATICS INC
2009487196	CYPY	CYNOPS	PYRRHOGASTER	JAPANESE FIREBELLY	NEWT	LIV	135	NO	HK	HK	T	W	C	C	10/13/09	10/11/09	I	NY	Transship Discounts Ltd.
2009487704	TRIH	TRITURUS	HONGKONGENSIS	ALPINE	NEWT	LIV	1,600	NO	HK	HK	T	C	C	C	10/14/09	10/15/09	I	TP	5-D TROPICAL INC.
2009488064	NOVI	NOTOPHTHALMUS	VIRIDESCENS	EASTERN	NEWT	LIV	7	NO	US	CA	T	W	C	C	10/14/09	10/14/09	I	BN	Science Kit, Inc - Boreal
2009489494	CNP?	CYNOPS	SPECIES	FIREBELLY	NEWT	LIV	200	NO	CN	CN	T	C	C	C	10/17/09	10/17/09	I	AT	SUN PET LTD.
2009489501	CYOR	CYNOPS	ORIENTALIS	CHINESE DWARF	NEWT	LIV	250	NO	HK	HK	T	C	C	C	10/17/09	10/17/09	I	LA	DOLPHIN INTERNATIONAL
2009489590	CYOR	CYNOPS	ORIENTALIS	CHINESE DWARF	NEWT	LIV	850	NO	HK	HK	T	C	C	C	10/18/09	10/18/09	I	LA	DOLPHIN INTERNATIONAL
2009489613	TRIH	TRITURUS	HONGKONGENSIS	ALPINE	NEWT	LIV	345	NO	CN	CN	T	W	C	C	10/18/09	10/18/09	I	LA	RENAISSANCE AQUATICS INC
2009490022	NOVI	NOTOPHTHALMUS	VIRIDESCENS	EASTERN	NEWT	LIV	3	NO	US	CA	T	W	C	C	10/19/09	10/19/09	I	BN	Science Kit, Inc - Boreal
2009491836	TRIH	TRITURUS	HONGKONGENSIS	ALPINE	NEWT	LIV	1,600	NO	HK	HK	T	C	C	C	10/21/09	10/22/09	I	TP	5-D TROPICAL INC.
2009492356	CYPY	CYNOPS	PYRRHOGASTER	JAPANESE FIREBELLY	NEWT	LIV	135	NO	HK	HK	T	W	C	C	10/22/09	10/18/09	I	NY	Transship Discounts Ltd.
2009494315	CYPY	CYNOPS	PYRRHOGASTER	JAPANESE FIREBELLY	NEWT	LIV	150	NO	HK	HK	T	W	C	C	10/26/09	10/25/09	I	NY	ERDA INCORPORATED
2009494436	NOVI	NOTOPHTHALMUS	VIRIDESCENS	EASTERN	NEWT	LIV	7	NO	US	CA	T	W	C	C	10/26/09	10/26/09	I	BN	Science Kit, Inc - Boreal
2009494512	PACB	PACHYTRITON	BREVIPES	TSITOU	NEWT	LIV	100	NO	HK	HK	T	C	C	C	10/26/09	10/24/09	I	LA	DOLPHIN INTERNATIONAL
2009494633	CYOR	CYNOPS	ORIENTALIS	CHINESE DWARF	NEWT	LIV	350	NO	HK	HK	T	C	C	C	10/26/09	10/25/09	I	LA	DOLPHIN INTERNATIONAL
2009495889	PACB	PACHYTRITON	BREVIPES	TSITOU	NEWT	LIV	60	NO	HK	HK	T	W	C	C	10/28/09	10/25/09	I	NY	Transship Discounts Ltd.
2009496586	TRIH	TRITURUS	HONGKONGENSIS	ALPINE	NEWT	LIV	1,600	NO	HK	HK	T	C	C	C	10/29/09	10/27/09	I	TP	5-D TROPICAL INC.
2009497101	NOVI	NOTOPHTHALMUS	VIRIDESCENS	EASTERN	NEWT	LIV	4	NO	US	CA	T	W	C	C	10/29/09	10/28/09	I	BN	Science Kit, Inc - Boreal
2009497773	AMBM	AMBYSTOMA	MEXICANUM		AXOLOTL	LIV	30	NO	DE	DE	S	C	C	C	10/30/09	10/29/09	I	NY	UNIVERSITY OF KENTUCKY
2009498128	CNP?	CYNOPS	SPECIES	FIREBELLY	NEWT	LIV	200	NO	CN	CN	T	C	C	C	10/31/09	10/31/09	I	AT	SUN PET LTD.

2009498138	CYOR	CYNOPS	ORIENTALIS	CHINESE DWARF	NEWT	LIV	250	NO	HK	HK	T	C	C	C	10/31/09	10/31/09	I	LA	DOLPHIN INTERNATIONAL
2009498238	CYOR	CYNOPS	ORIENTALIS	CHINESE DWARF	NEWT	LIV	250	NO	HK	HK	T	C	C	C	10/31/09	11/1/09	I	LA	DOLPHIN INTERNATIONAL
2009498253	TRIH	TRITURUS	HONGKONGENSIS	ALPINE	NEWT	LIV	100	NO	CN	CN	T	W	C	C	11/1/09	11/1/09	I	LA	RENAISSANCE AQUATICS INC
2009502699	CNP?	CYNOPS	SPECIES	FIREBELLY	NEWT	LIV	200	NO	CN	CN	T	C	C	C	11/7/09	11/7/09	I	AT	SUN PET LTD.
2009502772	TRIH	TRITURUS	HONGKONGENSIS	ALPINE	NEWT	LIV	50	NO	HK	HK	T	C	C	C	11/7/09	11/8/09	I	LA	AQUACO, INC.
2009502788	CYOR	CYNOPS	ORIENTALIS	CHINESE DWARF	NEWT	LIV	250	NO	HK	HK	T	C	C	C	11/8/09	11/8/09	I	LA	DOLPHIN INTERNATIONAL
2009502947	CYPY	CYNOPS	PYRRHOGASTER	JAPANESE FIREBELLY	NEWT	LIV	105	NO	HK	HK	T	C	C	C	11/9/09	11/1/09	I	NY	Transship Discounts Ltd.
2009503343	TRIH	TRITURUS	HONGKONGENSIS	ALPINE	NEWT	LIV	450	NO	HK	HK	T	C	C	C	11/9/09	11/9/09	I	CH	AQUATICS INC
2009503414	NOVI	NOTOPHTHALMUS	VIRIDESCENS	EASTERN	NEWT	LIV	3	NO	US	CA	T	W	C	C	11/9/09	11/9/09	I	BN	Science Kit, Inc - Boreal
2009505146	CYPY	CYNOPS	PYRRHOGASTER	JAPANESE FIREBELLY	NEWT	LIV	60	NO	HK	HK	T	C	C	C	11/12/09	11/8/09	I	NY	Transship Discounts Ltd.
2009505146	PACB	PACHYTRITON	BREVIPES	TSITOU	NEWT	LIV	60	NO	HK	HK	T	W	C	C	11/12/09	11/8/09	I	NY	Transship Discounts Ltd.
2009506806	CYOR	CYNOPS	ORIENTALIS	CHINESE DWARF	NEWT	LIV	250	NO	HK	HK	T	C	C	C	11/14/09	11/14/09	I	LA	DOLPHIN INTERNATIONAL
2009506858	TRIH	TRITURUS	HONGKONGENSIS	ALPINE	NEWT	LIV	75	NO	HK	HK	T	C	C	C	11/14/09	11/15/09	I	LA	AQUACO, INC.
2009506881	CYOR	CYNOPS	ORIENTALIS	CHINESE DWARF	NEWT	LIV	450	NO	HK	HK	T	C	C	C	11/15/09	11/15/09	I	LA	DOLPHIN INTERNATIONAL
2009506891	TRIH	TRITURUS	HONGKONGENSIS	ALPINE	NEWT	LIV	300	NO	CN	CN	T	W	C	C	11/15/09	11/15/09	I	LA	RENAISSANCE AQUATICS INC
2009507358	PLAB	PACHYTRITON	LABIATUS	UNTERSTEIN'S	NEWT	LIV	157	NO	HK	HK	T	C	C	C	11/16/09	11/16/09	I	DF	U.S. GLOBAL EXOTICS, INC.
2009507358	CNP?	CYNOPS	SPECIES	FIREBELLY	NEWT	LIV	300	NO	HK	HK	T	C	C	C	11/16/09	11/16/09	I	DF	U.S. GLOBAL EXOTICS, INC.
2009507358	PCHI	PARAMESOTRITON	CHINENSIS	CHINESE WARTY	NEWT	LIV	200	NO	HK	HK	T	C	C	C	11/16/09	11/16/09	I	DF	U.S. GLOBAL EXOTICS, INC.
2009508659	TRIH	TRITURUS	HONGKONGENSIS	ALPINE	NEWT	LIV	1,600	NO	HK	HK	T	C	C	C	11/18/09	11/18/09	I	TP	5-D TROPICAL INC.
2009510631	NOVI	NOTOPHTHALMUS	VIRIDESCENS	EASTERN	NEWT	LIV	3	NO	US	CA	T	W	C	C	11/20/09	11/18/09	I	BN	Science Kit, Inc - Boreal
2009511047	CYOR	CYNOPS	ORIENTALIS	CHINESE DWARF	NEWT	LIV	100	NO	HK	HK	T	C	C	C	11/22/09	11/22/09	I	LA	DOLPHIN INTERNATIONAL
2009511078	TRIH	TRITURUS	HONGKONGENSIS	ALPINE	NEWT	LIV	200	NO	CN	CN	T	W	C	C	11/22/09	11/22/09	I	LA	RENAISSANCE AQUATICS INC
2009511683	NOVI	NOTOPHTHALMUS	VIRIDESCENS	EASTERN	NEWT	LIV	2	NO	US	CA	T	W	C	C	11/23/09	11/23/09	I	BN	Science Kit, Inc - Boreal
2009511695	CYPY	CYNOPS	PYRRHOGASTER	JAPANESE FIREBELLY	NEWT	LIV	270	NO	HK	HK	T	C	C	C	11/23/09	11/22/09	I	NY	Transship Discounts Ltd.
2009512768	CYPY	CYNOPS	PYRRHOGASTER	JAPANESE FIREBELLY	NEWT	LIV	220	NO	HK	HK	T	W	C	C	11/24/09	11/22/09	I	NY	ERDA INCORPORATED
2009514353	CYOR	CYNOPS	ORIENTALIS	CHINESE DWARF	NEWT	LIV	350	NO	HK	HK	T	C	C	C	11/28/09	11/28/09	I	LA	DOLPHIN INTERNATIONAL
2009514457	CYOR	CYNOPS	ORIENTALIS	CHINESE DWARF	NEWT	LIV	750	NO	HK	HK	T	C	C	C	11/29/09	11/29/09	I	LA	DOLPHIN INTERNATIONAL
2009514457	PACB	PACHYTRITON	BREVIPES	TSITOU	NEWT	LIV	100	NO	HK	HK	T	C	C	C	11/29/09	11/29/09	I	LA	DOLPHIN INTERNATIONAL
2009515282	PACB	PACHYTRITON	BREVIPES	TSITOU	NEWT	LIV	60	NO	HK	HK	T	C	C	C	11/30/09	11/29/09	I	NY	Transship Discounts Ltd.
2009515282	CYPY	CYNOPS	PYRRHOGASTER	JAPANESE FIREBELLY	NEWT	LIV	12	NO	HK	HK	T	C	C	C	11/30/09	11/29/09	I	NY	Transship Discounts Ltd.
2009516348	CYPY	CYNOPS	PYRRHOGASTER	JAPANESE FIREBELLY	NEWT	LIV	70	NO	HK	HK	T	W	C	C	12/2/09	11/29/09	I	NY	ERDA INCORPORATED
2009517097	TRIH	TRITURUS	HONGKONGENSIS	ALPINE	NEWT	LIV	1,600	NO	HK	HK	T	C	C	C	12/2/09	12/2/09	I	TP	5-D TROPICAL INC.
2009517727	CYPY	CYNOPS	PYRRHOGASTER	JAPANESE FIREBELLY	NEWT	LIV	230	NO	HK	HK	T	W	C	C	6/14/09	5/14/09	I	NY	ERDA INCORPORATED
2009517778	NOVI	NOTOPHTHALMUS	VIRIDESCENS	EASTERN	NEWT	LIV	4	NO	US	CA	T	W	C	C	12/2/09	12/2/09	I	BN	Science Kit, Inc - Boreal
2009517970	CYPY	CYNOPS	PYRRHOGASTER	JAPANESE FIREBELLY	NEWT	LIV	210	NO	HK	HK	T	W	C	C	6/21/09	6/21/09	I	NY	ERDA INCORPORATED
2009518536	CYPY	CYNOPS	PYRRHOGASTER	JAPANESE FIREBELLY	NEWT	LIV	100	NO	HK	HK	T	W	C	C	11/8/08	11/8/08	I	NY	ERDA INCORPORATED
2009518798	CYOR	CYNOPS	ORIENTALIS	CHINESE DWARF	NEWT	LIV	250	NO	HK	HK	T	C	C	C	12/5/09	12/5/09	I	LA	DOLPHIN INTERNATIONAL
2009518798	PACB	PACHYTRITON	BREVIPES	TSITOU	NEWT	LIV	200	NO	HK	HK	T	C	C	C	12/5/09	12/5/09	I	LA	DOLPHIN INTERNATIONAL
2009518838	CNP?	CYNOPS	SPECIES	FIREBELLY	NEWT	LIV	200	NO	CN	CN	T	C	C	C	12/5/09	12/5/09	I	AT	SUN PET LTD.
2009518919	TRIH	TRITURUS	HONGKONGENSIS	ALPINE	NEWT	LIV	200	NO	HK	HK	T	C	C	C	12/5/09	12/6/09	I	LA	AQUACO, INC.
2009518931	PACB	PACHYTRITON	BREVIPES	TSITOU	NEWT	LIV	100	NO	HK	HK	T	C	C	C	12/5/09	12/6/09	I	LA	DOLPHIN INTERNATIONAL
2009518987	CYPY	CYNOPS	PYRRHOGASTER	JAPANESE FIREBELLY	NEWT	LIV	70	NO	HK	HK	T	W	C	C	12/7/09	12/6/09	I	NY	ERDA INCORPORATED
2009519073	CNP?	CYNOPS	SPECIES	FIREBELLY	NEWT	LIV	0	NO	CN	CN	T	W	C	C	9/9/08	8/31/08	I	NY	FISHMART INCORPORATED
2009519073	CNP?	CYNOPS	SPECIES	FIREBELLY	NEWT	LIV	0	NO	CN	CN	T	W	C	C	9/9/08	8/31/08	I	NY	FISHMART INCORPORATED
2009519274	CYOR	CYNOPS	ORIENTALIS	CHINESE DWARF	NEWT	LIV	100	NO	SG	SG	T	W	C	C	7/27/08	7/27/08	I	NY	INTERNATIONAL PET RESOURCES , LLC
2009519275	ANJA	ANDRIAS	JAPONICUS	JAPANESE GIANT	SALAMANDER	LIV	6	NO	JP	JP	Z	F	C	C	12/7/09	12/7/09	I	DU	SMITHSONIAN NATIONAL ZOOLOGICAL PARK
2009519680	NOVI	NOTOPHTHALMUS	VIRIDESCENS	EASTERN	NEWT	LIV	4	NO	US	CA	T	W	C	C	12/7/09	12/7/09	I	BN	Science Kit, Inc - Boreal
2009519974	CYPY	CYNOPS	PYRRHOGASTER	JAPANESE FIREBELLY	NEWT	LIV	70	NO	HK	HK	T	W	C	C	12/8/09	11/1/09	I	NY	ERDA INCORPORATED
2009520430	CYPY	CYNOPS	PYRRHOGASTER	JAPANESE FIREBELLY	NEWT	LIV	100	NO	HK	HK	T	C	C	C	3/20/08	3/15/08	I	NY	ERDA INCORPORATED
2009520812	CYOR	CYNOPS	ORIENTALIS	CHINESE DWARF	NEWT	LIV	100	NO	SG	SG	T	W	C	C	3/9/08	3/9/08	I	NY	INTERNATIONAL PET RESOURCES, LLC
2009523524	CYPY	CYNOPS	PYRRHOGASTER	JAPANESE FIREBELLY	NEWT	LIV	330	NO	HK	HK	T	W	C	C	6/4/09	5/31/09	I	NY	ERDA INCORPORATED
2009523812	CYOR	CYNOPS	ORIENTALIS	CHINESE DWARF	NEWT	LIV	500	NO	CN	CN	T	W	C	C	12/14/09	12/14/09	I	LA	WORLDWIDE TRADING INC
2009523812	PLAB	PACHYTRITON	LABIATUS	UNTERSTEIN'S	NEWT	LIV	500	NO	CN	CN	T	W	C	C	12/14/09	12/14/09	I	LA	WORLDWIDE TRADING INC
2009524968	CYPY	CYNOPS	PYRRHOGASTER	JAPANESE FIREBELLY	NEWT	LIV	60	NO	HK	HK	T	C	C	C	12/15/09	12/13/09	I	NY	Transship Discounts Ltd.
2009525323	CYOR	CYNOPS	ORIENTALIS	CHINESE DWARF	NEWT	LIV	100	NO	HK	HK	T	C	C	C	12/15/09	12/13/09	I	LA	DOLPHIN INTERNATIONAL
2009525323	PACB	PACHYTRITON	BREVIPES	TSITOU	NEWT	LIV	100	NO	HK	HK	T	C	C	C	12/15/09	12/13/09	I	LA	DOLPHIN INTERNATIONAL
2009526849	CNP?	CYNOPS	SPECIES	FIREBELLY	NEWT	LIV	0	NO	SG	SG	T	W	C	C	6/21/09	6/21/09	I	NY	INTERNATIONAL PET RESOURCES , LLC
2009527075	CYOR	CYNOPS	ORIENTALIS	CHINESE DWARF	NEWT	LIV	0	NO	SG	SG	T	W	C	C	6/10/09	5/24/09	I	NY	INTERNATIONAL PET RESOURCES , LLC
2009527127	NEW?	PACHYTRITON	SPECIES	CHINESE	NEWT	LIV	55	NO	SG	SG	T	W	C	C	4/26/09	4/26/09	I	NY	MERIT IMPORTS INCORPORATED

2009527449	CYOR	CYNOPS	ORIENTALIS	CHINESE DWARF	NEWT	LIV	120	NO	SG	SG	T	C	C	C	5/10/09	5/10/09	I	NY	INTERNATIONAL PET RESOURCES , LLC
2009527524	CYOR	CYNOPS	ORIENTALIS	CHINESE DWARF	NEWT	LIV	120	NO	SG	SG	T	C	C	C	5/17/09	4/17/09	I	NY	INTERNATIONAL PET RESOURCES , LLC
2009527634	CYPY	CYNOPS	PYRRHOGASTER	JAPANESE FIREBELLY	NEWT	LIV	370	NO	HK	HK	T	W	C	C	12/18/09	12/13/09	I	NY	ERDA INCORPORATED
2009527995	CYOR	CYNOPS	ORIENTALIS	CHINESE DWARF	NEWT	LIV	500	NO	HK	HK	T	C	C	C	12/19/09	12/20/09	I	LA	DOLPHIN INTERNATIONAL
2009530339	SASA	SALAMANDRA	SALAMANDRA	EUROPEAN FIRE	SALAMANDER	LIV	9	NO	DE	DE	T	C	C	C	12/23/09	12/22/09	I	MI	GLADES HERP FARM
2009531515	CYOR	CYNOPS	ORIENTALIS	CHINESE DWARF	NEWT	LIV	500	NO	HK	HK	T	C	C	C	12/26/09	12/26/09	I	LA	DOLPHIN INTERNATIONAL
2009531533	CYOR	CYNOPS	ORIENTALIS	CHINESE DWARF	NEWT	LIV	440	NO	CN	CN	T	W	C	C	12/26/09	12/26/09	I	LA	RENAISSANCE AQUATICS INC
2009531584	CYOR	CYNOPS	ORIENTALIS	CHINESE DWARF	NEWT	LIV	500	NO	HK	HK	T	C	C	C	12/26/09	12/27/09	I	LA	DOLPHIN INTERNATIONAL
2009532110	PACB	PACHYTRITON	BREVIPES	TSITOU	NEWT	LIV	60	NO	HK	HK	T	C	C	C	12/28/09	12/27/09	I	NY	Transship Discounts Ltd.
2009533044	CYPY	CYNOPS	PYRRHOGASTER	JAPANESE FIREBELLY	NEWT	LIV	150	NO	HK	HK	T	W	C	C	12/30/09	12/27/09	I	NY	ERDA INCORPORATED
2010534472	CYOR	CYNOPS	ORIENTALIS	CHINESE DWARF	NEWT	LIV	100	NO	HK	HK	T	C	C	C	1/2/10	1/2/10	I	LA	DOLPHIN INTERNATIONAL
2010534558	TRIH	TRITURUS	HONGKONGENSIS	ALPINE	NEWT	LIV	25	NO	HK	HK	T	C	C	C	1/3/10	1/3/10	I	LA	AQUACO, INC.
2010534569	CYOR	CYNOPS	ORIENTALIS	CHINESE DWARF	NEWT	LIV	600	NO	HK	HK	T	C	C	C	1/3/10	1/3/10	I	LA	DOLPHIN INTERNATIONAL
2010534583	TRIT	TRITURUS	SPECIES		NEWT	LIV	210	NO	CN	CN	T	W	C	C	1/3/10	1/3/10	I	LA	RENAISSANCE AQUATICS INC
2010534583	CYOR	CYNOPS	ORIENTALIS	CHINESE DWARF	NEWT	LIV	235	NO	CN	CN	T	W	C	C	1/3/10	1/3/10	I	LA	RENAISSANCE AQUATICS INC
2010537163	NOVI	NOTOPHTHALMUS	VIRIDESCENS	EASTERN	NEWT	LIV	5	NO	US	CA	T	W	C	C	1/6/10	1/6/10	I	BN	Science Kit, Inc - Boreal
2010537235	TRIH	TRITURUS	HONGKONGENSIS	ALPINE	NEWT	LIV	1,600	NO	HK	HK	T	C	C	C	1/6/10	1/6/10	I	TP	5-D TROPICAL INC.
2010537502	CYPY	CYNOPS	PYRRHOGASTER	JAPANESE FIREBELLY	NEWT	LIV	30	NO	HK	HK	T	C	C	C	1/7/10	1/3/10	I	NY	Transship Discounts Ltd.
2010538526	CYOR	CYNOPS	ORIENTALIS	CHINESE DWARF	NEWT	LIV	250	NO	HK	HK	T	C	C	C	1/8/10	1/9/10	I	LA	DOLPHIN INTERNATIONAL
2010538526	PACB	PACHYTRITON	BREVIPES	TSITOU	NEWT	LIV	100	NO	HK	HK	T	C	C	C	1/8/10	1/9/10	I	LA	DOLPHIN INTERNATIONAL
2010538610	CYOR	CYNOPS	ORIENTALIS	CHINESE DWARF	NEWT	LIV	500	NO	HK	HK	T	C	C	C	1/9/10	1/10/10	I	LA	DOLPHIN INTERNATIONAL
2010538622	TRIH	TRITURUS	HONGKONGENSIS	ALPINE	NEWT	LIV	100	NO	HK	HK	T	C	C	C	1/9/10	1/10/10	I	LA	AQUACO, INC.
2010538653	TRIT	TRITURUS	SPECIES		NEWT	LIV	270	NO	CN	CN	T	W	C	C	1/10/10	1/10/10	I	LA	RENAISSANCE AQUATICS INC
2010538950	CYPY	CYNOPS	PYRRHOGASTER	JAPANESE FIREBELLY	NEWT	LIV	220	NO	HK	HK	T	W	C	C	1/11/10	1/3/10	I	NY	ERDA INCORPORATED
2010540012	NOVI	NOTOPHTHALMUS	VIRIDESCENS	EASTERN	NEWT	LIV	3	NO	US	CA	T	W	C	C	1/11/10	1/11/10	I	BN	Science Kit, Inc - Boreal
2010540545	CYPY	CYNOPS	PYRRHOGASTER	JAPANESE FIREBELLY	NEWT	LIV	70	NO	HK	HK	T	W	C	C	1/13/10	1/10/10	I	NY	ERDA INCORPORATED
2010540555	CYPY	CYNOPS	PYRRHOGASTER	JAPANESE FIREBELLY	NEWT	LIV	195	NO	HK	HK	T	C	C	C	1/13/10	1/10/10	I	NY	Transship Discounts Ltd.
2010541965	CYOR	CYNOPS	ORIENTALIS	CHINESE DWARF	NEWT	LIV	250	NO	HK	HK	T	C	C	C	1/16/10	1/16/10	I	LA	DOLPHIN INTERNATIONAL
2010542002	CYOR	CYNOPS	ORIENTALIS	CHINESE DWARF	NEWT	LIV	200	NO	CN	CN	T	W	C	C	1/16/10	1/16/10	I	LA	WORLDWIDE TRADING INC
2010542002	PLAB	PACHYTRITON	LABIATUS	UNTERSTEIN'S	NEWT	LIV	500	NO	CN	CN	T	W	C	C	1/16/10	1/16/10	I	LA	WORLDWIDE TRADING INC
2010542072	CYOR	CYNOPS	ORIENTALIS	CHINESE DWARF	NEWT	LIV	350	NO	HK	HK	T	C	C	C	1/16/10	1/17/10	I	LA	DOLPHIN INTERNATIONAL
2010542117	CYOR	CYNOPS	ORIENTALIS	CHINESE DWARF	NEWT	LIV	145	NO	CN	CN	T	W	C	C	1/17/10	1/17/10	I	LA	RENAISSANCE AQUATICS INC
2010542117	TRIT	TRITURUS	SPECIES		NEWT	LIV	317	NO	CN	CN	T	W	C	C	1/17/10	1/17/10	I	LA	RENAISSANCE AQUATICS INC
2010542461	CYPY	CYNOPS	PYRRHOGASTER	JAPANESE FIREBELLY	NEWT	LIV	210	NO	HK	HK	T	W	C	C	1/19/10	1/17/10	I	NY	ERDA INCORPORATED
2010543541	NOVI	NOTOPHTHALMUS	VIRIDESCENS	EASTERN	NEWT	LIV	5	NO	US	CA	T	W	C	C	1/20/10	1/20/10	I	BN	Science Kit, Inc - Boreal
2010544871	CYOR	CYNOPS	ORIENTALIS	CHINESE DWARF	NEWT	LIV	250	NO	HK	HK	T	C	C	C	1/23/10	1/23/10	I	LA	DOLPHIN INTERNATIONAL
2010544986	TRIT	TRITURUS	SPECIES		NEWT	LIV	450	NO	CN	CN	T	W	C	C	1/24/10	1/24/10	I	LA	RENAISSANCE AQUATICS INC
2010544993	CYOR	CYNOPS	ORIENTALIS	CHINESE DWARF	NEWT	LIV	100	NO	HK	HK	T	C	C	C	1/24/10	1/24/10	I	LA	DOLPHIN INTERNATIONAL
2010544993	PACB	PACHYTRITON	BREVIPES	TSITOU	NEWT	LIV	200	NO	HK	HK	T	C	C	C	1/24/10	1/24/10	I	LA	DOLPHIN INTERNATIONAL
2010545382	NOVI	NOTOPHTHALMUS	VIRIDESCENS	EASTERN	NEWT	LIV	1	NO	US	CA	T	W	C	C	1/25/10	1/25/10	I	BN	Science Kit, Inc - Boreal
2010546114	CYPY	CYNOPS	PYRRHOGASTER	JAPANESE FIREBELLY	NEWT	LIV	60	NO	HK	HK	T	C	C	C	1/26/10	1/17/10	I	NY	Transship Discounts Ltd.
2010546611	CYPY	CYNOPS	PYRRHOGASTER	JAPANESE FIREBELLY	NEWT	LIV	190	NO	HK	HK	T	W	C	C	1/27/10	1/24/10	I	NY	ERDA INCORPORATED
2010547277	CYPY	CYNOPS	PYRRHOGASTER	JAPANESE FIREBELLY	NEWT	LIV	105	NO	HK	HK	T	C	C	C	1/28/10	1/24/10	I	NY	Transship Discounts Ltd.
2010547277	PACB	PACHYTRITON	BREVIPES	TSITOU	NEWT	LIV	30	NO	HK	HK	T	C	C	C	1/28/10	1/24/10	I	NY	Transship Discounts Ltd.
2010548112	TRIT	TRITURUS	SPECIES		NEWT	LIV	316	NO	CN	CN	T	W	C	C	1/31/10	1/31/10	I	LA	RENAISSANCE AQUATICS INC
2010548434	CYPY	CYNOPS	PYRRHOGASTER	JAPANESE FIREBELLY	NEWT	LIV	570	NO	HK	HK	*	W	C	C	5/10/09	5/10/09	I	NY	ERDA INC
2010548483	CYPY	CYNOPS	PYRRHOGASTER	JAPANESE FIREBELLY	NEWT	LIV	270	NO	HK	HK	*	W	C	C	5/3/09	5/3/09	I	NY	ERDA INC
2010548496	CYPY	CYNOPS	PYRRHOGASTER	JAPANESE FIREBELLY	NEWT	LIV	210	NO	HK	HK	T	W	C	C	4/26/09	4/26/09	I	NY	ERDA INC
2010548506	NOVI	NOTOPHTHALMUS	VIRIDESCENS	EASTERN	NEWT	LIV	3	NO	US	CA	T	W	C	C	2/1/10	2/1/10	I	BN	Science Kit, Inc - Boreal
2010548566	CYOR	CYNOPS	ORIENTALIS	CHINESE DWARF	NEWT	LIV	120	NO	SG	SG	T	W	C	C	5/3/09	5/3/09	I	NY	INTERNATIONAL PET RESOURCES , LLC
2010548911	CYPY	CYNOPS	PYRRHOGASTER	JAPANESE FIREBELLY	NEWT	LIV	540	NO	HK	HK	T	W	C	C	3/28/09	3/28/09	I	NY	ERDA INCORPORATED
2010548941	CYPY	CYNOPS	PYRRHOGASTER	JAPANESE FIREBELLY	NEWT	LIV	210	NO	HK	HK	T	W	C	C	12/20/08	12/20/08	I	NY	ERDA INCORPORATED
2010548951	CYPY	CYNOPS	PYRRHOGASTER	JAPANESE FIREBELLY	NEWT	LIV	600	NO	HK	HK	T	W	C	C	3/14/09	3/14/09	I	NY	ERDA INCORPORATED
2010548953	CYPY	CYNOPS	PYRRHOGASTER	JAPANESE FIREBELLY	NEWT	LIV	30	NO	HK	HK	T	W	C	C	3/21/09	3/21/09	I	NY	ERDA INCORPORATED
2010549704	CYOR	CYNOPS	ORIENTALIS	CHINESE DWARF	NEWT	LIV	100	NO	SG	SG	T	W	C	C	11/14/08	11/14/08	I	NY	INTERNATIONAL PET RESOURCES , LLC
2010549788	CYPY	CYNOPS	PYRRHOGASTER	JAPANESE FIREBELLY	NEWT	LIV	150	NO	HK	HK	T	W	C	C	11/29/08	11/29/08	I	NY	ERDA INCORPORATED
2010549869	CYPY	CYNOPS	PYRRHOGASTER	JAPANESE FIREBELLY	NEWT	LIV	350	NO	HK	HK	T	W	C	C	2/28/09	2/28/09	I	NY	ERDA INCORPORATED
2010550112	NOVI	NOTOPHTHALMUS	VIRIDESCENS	EASTERN	NEWT	LIV	7	NO	US	CA	T	W	C	C	2/3/10	2/3/10	I	BN	Science Kit, Inc - Boreal
2010550126	CYPY	CYNOPS	PYRRHOGASTER	JAPANESE FIREBELLY	NEWT	LIV	60	NO	HK	HK	T	W	C	C	6/6/09	6/6/09	I	NY	ERDA INCORPORATED
2010550389	CYOR	CYNOPS	ORIENTALIS	CHINESE DWARF	NEWT	LIV	100	NO	SG	SG	T	W	C	C	1/25/09	1/25/09	I	NY	INTERNATIONAL PET RESOURCES , LLC

2010550447	CNP?	CYNOPS	SPECIES	FIREBELLY	NEWT	LIV	400	NO	CN	CN	T	W	C	C	10/26/08	10/26/08	I	NY	FISH MART INCORPORATED
2010550467	TRIT	TRITURUS	SPECIES		NEWT	LIV	350	NO	HK	HK	T	W	C	C	10/8/09	10/8/09	I	SF	PAN OCEAN AQUARIUM
2010550496	CYPY	CYNOPS	PYRRHOGASTER	JAPANESE FIREBELLY	NEWT	LIV	60	NO	HK	HK	T	W	C	C	1/3/09	1/3/09	I	NY	ERDA INCORPORATED
2010551961	CYOR	CYNOPS	ORIENTALIS	CHINESE DWARF	NEWT	LIV	1,950	NO	HK	HK	T	C	C	C	2/6/10	2/7/10	I	LA	DOLPHIN INTERNATIONAL
2010551977	CYOR	CYNOPS	ORIENTALIS	CHINESE DWARF	NEWT	LIV	200	NO	CN	CN	T	W	C	C	2/7/10	2/7/10	I	LA	RENAISSANCE AQUATICS INC
2010551977	TRIH	TRITURUS	HONGKONGENSIS	ALPINE	NEWT	LIV	678	NO	CN	CN	T	W	C	C	2/7/10	2/7/10	I	LA	RENAISSANCE AQUATICS INC
2010553265	PLAB	PACHYTRITON	LABIATUS	UNTERSTEIN'S	NEWT	LIV	500	NO	CN	CN	T	W	C	C	2/9/10	2/9/10	I	LA	WORLDWIDE TRADING INC
2010553357	NOVI	NOTOPHTHALMUS	VIRIDESCENS	EASTERN	NEWT	LIV	6	NO	US	CA	T	W	C	C	2/8/10	2/8/10	I	BN	Science Kit, Inc - Boreal
2010554040	NOVI	NOTOPHTHALMUS	VIRIDESCENS	EASTERN	NEWT	LIV	1	NO	US	CA	T	W	C	C	2/10/10	2/10/10	I	BN	Science Kit, Inc - Boreal
2010555067	SAL?	SALAMANDRA	SPECIES	FIRE	SALAMANDER	LIV	6	NO	GE	DE	T	C	C	C	8/5/09	8/5/09	I	SF	Exemptions 6 and 7(C)
2010555067	SAL?	SALAMANDRA	SPECIES	FIRE	SALAMANDER	LIV	17	NO	GE	DE	T	C	C	C	8/5/09	8/5/09	I	SF	Exemptions 6 and 7(C)
2010555067	NRR?	NEURERGUS	SPECIES		NEWT	LIV	2	NO	GE	DE	T	C	C	C	8/5/09	8/5/09	I	SF	Exemptions 6 and 7(C)
2010555394	CYPY	CYNOPS	PYRRHOGASTER	JAPANESE FIREBELLY	NEWT	LIV	60	NO	HK	HK	T	C	C	C	2/12/10	2/7/10	I	NY	Transship Discounts Ltd.
2010555555	CYOR	CYNOPS	ORIENTALIS	CHINESE DWARF	NEWT	LIV	250	NO	HK	HK	T	C	C	C	2/13/10	2/13/10	I	LA	DOLPHIN INTERNATIONAL
2010555555	PACB	PACHYTRITON	BREVIPES	TSITOU	NEWT	LIV	100	NO	HK	HK	T	C	C	C	2/13/10	2/13/10	I	LA	DOLPHIN INTERNATIONAL
2010555652	CYOR	CYNOPS	ORIENTALIS	CHINESE DWARF	NEWT	LIV	450	NO	HK	HK	T	C	C	C	2/14/10	2/14/10	I	LA	DOLPHIN INTERNATIONAL
2010555652	PACB	PACHYTRITON	BREVIPES	TSITOU	NEWT	LIV	100	NO	HK	HK	T	C	C	C	2/14/10	2/14/10	I	LA	DOLPHIN INTERNATIONAL
2010557022	CYPY	CYNOPS	PYRRHOGASTER	JAPANESE FIREBELLY	NEWT	LIV	105	NO	HK	HK	T	C	C	C	2/17/10	2/14/10	I	NY	Transship Discounts Ltd.
2010557022	PACB	PACHYTRITON	BREVIPES	TSITOU	NEWT	LIV	60	NO	HK	HK	T	C	C	C	2/17/10	2/14/10	I	NY	Transship Discounts Ltd.
2010557173	NOVI	NOTOPHTHALMUS	VIRIDESCENS	EASTERN	NEWT	LIV	4	NO	US	CA	T	W	C	C	2/17/10	2/17/10	I	BN	Science Kit, Inc - Boreal
2010558344	CYPY	CYNOPS	PYRRHOGASTER	JAPANESE FIREBELLY	NEWT	LIV	300	NO	HK	HK	T	W	C	C	2/19/10	2/7/10	I	NY	ERDA INCORPORATED
2010558498	CYPY	CYNOPS	PYRRHOGASTER	JAPANESE FIREBELLY	NEWT	LIV	130	NO	HK	HK	T	W	C	C	2/19/10	2/14/10	I	NY	ERDA INCORPORATED
2010559005	CYOR	CYNOPS	ORIENTALIS	CHINESE DWARF	NEWT	LIV	100	NO	HK	HK	T	C	C	C	2/20/10	2/20/10	I	LA	DOLPHIN INTERNATIONAL
2010559066	CYOR	CYNOPS	ORIENTALIS	CHINESE DWARF	NEWT	LIV	200	NO	HK	HK	T	C	C	C	2/21/10	2/21/10	I	LA	DOLPHIN INTERNATIONAL
2010559066	PACB	PACHYTRITON	BREVIPES	TSITOU	NEWT	LIV	100	NO	HK	HK	T	C	C	C	2/21/10	2/21/10	I	LA	DOLPHIN INTERNATIONAL
2010559584	NOVI	NOTOPHTHALMUS	VIRIDESCENS	EASTERN	NEWT	LIV	5	NO	US	CA	T	W	C	C	2/22/10	2/22/10	I	BN	Science Kit, Inc - Boreal
2010561209	NOVI	NOTOPHTHALMUS	VIRIDESCENS	EASTERN	NEWT	LIV	2	NO	US	CA	T	W	C	C	2/24/10	2/24/10	I	BN	Science Kit, Inc - Boreal
2010561933	CYOR	CYNOPS	ORIENTALIS	CHINESE DWARF	NEWT	LIV	250	NO	HK	HK	T	C	C	C	2/27/10	2/27/10	I	LA	DOLPHIN INTERNATIONAL
2010562004	CYOR	CYNOPS	ORIENTALIS	CHINESE DWARF	NEWT	LIV	1,050	NO	HK	HK	T	C	C	C	2/28/10	2/28/10	I	LA	DOLPHIN INTERNATIONAL
2010562016	TRIT	TRITURUS	SPECIES		NEWT	LIV	45	NO	CN	CN	T	W	C	C	2/28/10	2/28/10	I	LA	RENAISSANCE AQUATICS INC
2010562021	TRIH	TRITURUS	HONGKONGENSIS	ALPINE	NEWT	LIV	350	NO	HK	HK	T	C	C	C	2/28/10	2/28/10	I	LA	AQUACO, INC.
2010562641	NOVI	NOTOPHTHALMUS	VIRIDESCENS	EASTERN	NEWT	LIV	1	NO	US	CA	T	W	C	C	3/1/10	3/1/10	I	BN	Science Kit, Inc - Boreal
2010565479	CYPY	CYNOPS	PYRRHOGASTER	JAPANESE FIREBELLY	NEWT	LIV	250	NO	HK	HK	T	W	C	C	3/5/10	2/21/10	I	NY	ERDA INCORPORATED
2010565637	CNP?	CYNOPS	SPECIES	FIREBELLY	NEWT	LIV	200	NO	CN	CN	T	C	C	C	3/6/10	3/6/10	I	AT	SUN PET LTD.
2010565642	CYOR	CYNOPS	ORIENTALIS	CHINESE DWARF	NEWT	LIV	500	NO	HK	HK	T	C	C	C	3/6/10	3/6/10	I	LA	DOLPHIN INTERNATIONAL
2010565716	TRIH	TRITURUS	HONGKONGENSIS	ALPINE	NEWT	LIV	342	NO	HK	HK	T	C	C	C	3/7/10	3/7/10	I	LA	AQUACO, INC.
2010565727	CYOR	CYNOPS	ORIENTALIS	CHINESE DWARF	NEWT	LIV	1,375	NO	HK	HK	T	C	C	C	3/7/10	3/7/10	I	LA	DOLPHIN INTERNATIONAL
2010565764	TRIT	TRITURUS	SPECIES		NEWT	LIV	200	NO	CN	CN	T	W	C	C	3/7/10	3/7/10	I	LA	RENAISSANCE AQUATICS INC
2010565810	NOVI	NOTOPHTHALMUS	VIRIDESCENS	EASTERN	NEWT	LIV	2	NO	US	CA	T	W	C	C	3/8/10	3/3/10	I	BN	Science Kit, Inc - Boreal
2010566246	CYPY	CYNOPS	PYRRHOGASTER	JAPANESE FIREBELLY	NEWT	LIV	330	NO	HK	HK	T	W	C	C	3/8/10	3/7/10	I	NY	ERDA INCORPORATED
2010566263	CYPY	CYNOPS	PYRRHOGASTER	JAPANESE FIREBELLY	NEWT	LIV	420	NO	HK	HK	T	C	C	C	3/8/10	3/7/10	I	NY	Transship Discounts Ltd.
2010566263	PACB	PACHYTRITON	BREVIPES	TSITOU	NEWT	LIV	210	NO	HK	HK	T	C	C	C	3/8/10	3/7/10	I	NY	Transship Discounts Ltd.
2010566283	CYPY	CYNOPS	PYRRHOGASTER	JAPANESE FIREBELLY	NEWT	LIV	140	NO	HK	HK	T	W	C	C	3/8/10	2/28/10	I	NY	ERDA INCORPORATED
2010566951	CYOR	CYNOPS	ORIENTALIS	CHINESE DWARF	NEWT	LIV	2,000	NO	CN	CN	T	W	C	C	3/9/10	3/9/10	I	LA	Z IMPORTS INC.
2010568067	TRIH	TRITURUS	HONGKONGENSIS	ALPINE	NEWT	LIV	2,400	NO	HK	HK	T	C	C	C	3/11/10	3/10/10	I	TP	5-D TROPICAL INC.
2010569267	CYOR	CYNOPS	ORIENTALIS	CHINESE DWARF	NEWT	LIV	500	NO	HK	HK	T	C	C	C	3/13/10	3/13/10	I	LA	DOLPHIN INTERNATIONAL
2010569267	PACB	PACHYTRITON	BREVIPES	TSITOU	NEWT	LIV	190	NO	HK	HK	T	C	C	C	3/13/10	3/13/10	I	LA	DOLPHIN INTERNATIONAL
2010569326	TRIH	TRITURUS	HONGKONGENSIS	ALPINE	NEWT	LIV	400	NO	HK	HK	T	C	C	C	3/13/10	3/14/10	I	LA	AQUACO, INC.
2010569337	CYOR	CYNOPS	ORIENTALIS	CHINESE DWARF	NEWT	LIV	300	NO	CN	CN	T	W	C	C	3/13/10	3/14/10	I	LA	RENAISSANCE AQUATICS INC
2010569337	PLAB	PACHYTRITON	LABIATUS	UNTERSTEIN'S	NEWT	LIV	100	NO	CN	CN	T	W	C	C	3/13/10	3/14/10	I	LA	RENAISSANCE AQUATICS INC
2010569351	CYOR	CYNOPS	ORIENTALIS	CHINESE DWARF	NEWT	LIV	500	NO	HK	HK	T	C	C	C	3/14/10	3/14/10	I	LA	DOLPHIN INTERNATIONAL
2010569351	PACB	PACHYTRITON	BREVIPES	TSITOU	NEWT	LIV	240	NO	HK	HK	T	C	C	C	3/14/10	3/14/10	I	LA	DOLPHIN INTERNATIONAL
2010571319	BOG?	BOLITOGLOSSA	SPECIES	MUSHROOMTONGUE	SALAMANDER	LIV	3	NO	MX	MX	P	W	R	A	11/9/09	11/9/09	I	DR	**
2010572070	CYPY	CYNOPS	PYRRHOGASTER	JAPANESE FIREBELLY	NEWT	LIV	150	NO	HK	HK	T	W	C	C	3/18/10	3/14/10	I	NY	ERDA INCORPORATED
2010572327	CYOR	CYNOPS	ORIENTALIS	CHINESE DWARF	NEWT	LIV	100	NO	SG	SG	T	W	C	C	9/13/09	9/13/09	I	NY	INTERNATIONAL PET RESOURCES , LLC
2010572538	CNP?	CYNOPS	SPECIES	FIREBELLY	NEWT	LIV	1	NO	MX	MX	P	W	R	A	4/18/09	4/18/09	I	DR	**
2010572765	CYOR	CYNOPS	ORIENTALIS	CHINESE DWARF	NEWT	LIV	0	NO	SG	SG	T	C	C	C	11/1/09	11/1/09	I	NY	INTERNATIONAL PET RESOURCES , LLC
2010573085	CYOR	CYNOPS	ORIENTALIS	CHINESE DWARF	NEWT	LIV	250	NO	HK	HK	T	C	C	C	3/20/10	3/20/10	I	LA	DOLPHIN INTERNATIONAL
2010573085	PACB	PACHYTRITON	BREVIPES	TSITOU	NEWT	LIV	150	NO	HK	HK	T	C	C	C	3/20/10	3/20/10	I	LA	DOLPHIN INTERNATIONAL
2010573177	CYOR	CYNOPS	ORIENTALIS	CHINESE DWARF	NEWT	LIV	250	NO	HK	HK	T	C	C	C	3/21/10	3/21/10	I	LA	DOLPHIN INTERNATIONAL

2010573177	PACB	PACHYTRITON	BREVIPES	TSITOU	NEWT	LIV	100	NO	HK	HK	T	C	C	C	3/21/10	3/21/10	I	LA	DOLPHIN INTERNATIONAL
2010573201	TRIH	TRITURUS	HONGKONGENSIS	ALPINE	NEWT	LIV	275	NO	HK	HK	T	C	C	C	3/21/10	3/21/10	I	LA	AQUACO, INC.
2010573211	PLAB	PACHYTRITON	LABIATUS	UNTERSTEIN'S	NEWT	LIV	100	NO	CN	CN	T	W	C	C	3/21/10	3/21/10	I	LA	RENAISSANCE AQUATICS INC
2010573211	CYOR	CYNOPS	ORIENTALIS	CHINESE DWARF	NEWT	LIV	740	NO	CN	CN	T	W	C	C	3/21/10	3/21/10	I	LA	RENAISSANCE AQUATICS INC
2010573297	CNP?	CYNOPS	SPECIES	FIREBELLY	NEWT	LIV	0	NO	CN	CN	T	W	C	C	4/5/09	4/5/09	I	NY	FISH MART INCORPORATED
2010573297	CNP?	CYNOPS	SPECIES	FIREBELLY	NEWT	LIV	0	NO	CN	CN	T	W	C	C	4/5/09	4/5/09	I	NY	FISH MART INCORPORATED
2010573305	CYPY	CYNOPS	PYRRHOGASTER	JAPANESE FIREBELLY	NEWT	LIV	360	NO	HK	HK	T	W	C	C	4/12/09	4/12/09	I	NY	ERDA INCORPORATED
2010573309	CYPY	CYNOPS	PYRRHOGASTER	JAPANESE FIREBELLY	NEWT	LIV	120	NO	HK	HK	T	W	C	C	4/5/09	4/5/09	I	NY	ERDA INCORPORATED
2010573312	CYOR	CYNOPS	ORIENTALIS	CHINESE DWARF	NEWT	LIV	120	NO	SG	SG	T	W	C	C	4/12/09	4/12/09	I	NY	INTERNATIONAL PET RESOURCES , LLC
2010573344	CYPY	CYNOPS	PYRRHOGASTER	JAPANESE FIREBELLY	NEWT	LIV	150	NO	HK	HK	T	W	C	C	3/22/10	3/21/10	I	NY	ERDA INCORPORATED
2010573370	NEW?	PACHYTRITON	SPECIES	CHINESE	NEWT	LIV	55	NO	SG	SG	T	W	C	C	5/31/09	5/31/09	I	NY	MERIT IMPORTS INCORPORATED
2010573377	CYPY	CYNOPS	PYRRHOGASTER	JAPANESE FIREBELLY	NEWT	LIV	420	NO	HK	HK	T	C	C	C	6/28/09	6/28/09	I	NY	ERDA INCORPORATED
2010573641	NOVI	NOTOPHTHALMUS	VIRIDESCENS	EASTERN	NEWT	LIV	5	NO	US	CA	T	W	C	C	3/22/10	3/22/10	I	BN	Science Kit, Inc - Boreal
2010573992	CYPY	CYNOPS	PYRRHOGASTER	JAPANESE FIREBELLY	NEWT	LIV	105	NO	HK	HK	T	C	C	C	3/23/10	3/21/10	I	NY	Transship Discounts Ltd.
2010573992	PACB	PACHYTRITON	BREVIPES	TSITOU	NEWT	LIV	30	NO	HK	HK	T	C	C	C	3/23/10	3/21/10	I	NY	Transship Discounts Ltd.
2010574247	NOVI	NOTOPHTHALMUS	VIRIDESCENS	EASTERN	NEWT	LIV	16	NO	US	CA	T	W	C	C	3/15/10	3/15/10	I	BN	Science Kit, Inc - Boreal
2010574532	CNP?	CYNOPS	SPECIES	FIREBELLY	NEWT	LIV	0	NO	SG	SG	T	W	C	C	8/9/09	8/9/09	I	NY	INTERNATIONAL PET RESOURCES , LLC
2010574554	CYOR	CYNOPS	ORIENTALIS	CHINESE DWARF	NEWT	LIV	150	NO	MY	MY	T	C	C	C	9/14/09	9/14/09	I	NY	TRI-COUNTY TROPICALS, INC.
2010574738	CYOR	CYNOPS	ORIENTALIS	CHINESE DWARF	NEWT	LIV	0	NO	SG	SG	T	W	C	C	10/4/09	10/4/09	I	NY	INTERNATIONAL PET RESOURCES , LLC
2010574741	CNP?	CYNOPS	SPECIES	FIREBELLY	NEWT	LIV	0	NO	SG	SG	T	W	C	C	10/11/09	10/11/09	I	NY	INTERNATIONAL PET RESOURCES , LLC
2010575195	TRIH	TRITURUS	HONGKONGENSIS	ALPINE	NEWT	LIV	1,600	NO	HK	HK	T	C	C	C	3/24/10	3/24/10	I	TP	5-D TROPICAL INC.
2010576510	CNP?	CYNOPS	SPECIES	FIREBELLY	NEWT	LIV	200	NO	CN	CN	T	C	C	C	3/27/10	3/27/10	I	AT	SUN PET LTD.
2010576529	CYOR	CYNOPS	ORIENTALIS	CHINESE DWARF	NEWT	LIV	250	NO	HK	HK	T	C	C	C	3/27/10	3/27/10	I	LA	DOLPHIN INTERNATIONAL
2010576611	CYOR	CYNOPS	ORIENTALIS	CHINESE DWARF	NEWT	LIV	700	NO	HK	HK	T	C	C	C	3/28/10	3/28/10	I	LA	DOLPHIN INTERNATIONAL
2010576611	PACB	PACHYTRITON	BREVIPES	TSITOU	NEWT	LIV	200	NO	HK	HK	T	C	C	C	3/28/10	3/28/10	I	LA	DOLPHIN INTERNATIONAL
2010576634	TRIT	TRITURUS	SPECIES		NEWT	LIV	45	NO	CN	CN	T	W	C	C	3/28/10	3/28/10	I	LA	RENAISSANCE AQUATICS INC
2010576634	CYOR	CYNOPS	ORIENTALIS	CHINESE DWARF	NEWT	LIV	1,020	NO	CN	CN	T	W	C	C	3/28/10	3/28/10	I	LA	RENAISSANCE AQUATICS INC
2010576690	HYN?	HYNOBIUS	SPECIES		SALAMANDER	LIV	57	NO	DE	DE	T	C	C	C	12/10/09	12/10/09	I	SF	Exemptions 6 and 7(C)
2010576690	HYN?	HYNOBIUS	SPECIES		SALAMANDER	LIV	14	NO	DE	DE	T	C	C	C	12/10/09	12/10/09	I	SF	Exemptions 6 and 7(C)
2010576690	HYN?	HYNOBIUS	SPECIES		SALAMANDER	LIV	30	NO	DE	DE	T	C	C	C	12/10/09	12/10/09	I	SF	Exemptions 6 and 7(C)
2010578281	CYPY	CYNOPS	PYRRHOGASTER	JAPANESE FIREBELLY	NEWT	LIV	60	NO	HK	HK	T	C	C	C	3/31/10	3/28/10	I	NY	Transship Discounts Ltd.
2010578281	PACB	PACHYTRITON	BREVIPES	TSITOU	NEWT	LIV	120	NO	HK	HK	T	C	C	C	3/31/10	3/28/10	I	NY	Transship Discounts Ltd.
2010578405	TRIH	TRITURUS	HONGKONGENSIS	ALPINE	NEWT	LIV	1,600	NO	HK	HK	T	C	C	C	3/31/10	3/31/10	I	TP	5-D TROPICAL INC.
2010578955	CYPY	CYNOPS	PYRRHOGASTER	JAPANESE FIREBELLY	NEWT	LIV	210	NO	HK	HK	T	W	C	C	4/1/10	3/28/10	I	NY	ERDA INCORPORATED
2010579611	PLAB	PACHYTRITON	LABIATUS	UNTERSTEIN'S	NEWT	LIV	200	NO	CN	CN	T	W	C	C	4/3/10	4/4/10	I	LA	WORLDWIDE TRADING INC
2010579681	CYOR	CYNOPS	ORIENTALIS	CHINESE DWARF	NEWT	LIV	875	NO	HK	HK	T	C	C	C	4/4/10	4/4/10	I	LA	DOLPHIN INTERNATIONAL
2010579686	TRIT	TRITURUS	SPECIES		NEWT	LIV	705	NO	CN	CN	T	W	C	C	4/4/10	4/4/10	I	LA	RENAISSANCE AQUATICS INC
2010579686	CYOR	CYNOPS	ORIENTALIS	CHINESE DWARF	NEWT	LIV	300	NO	CN	CN	T	W	C	C	4/4/10	4/4/10	I	LA	RENAISSANCE AQUATICS INC
2010580186	NOVI	NOTOPHTHALMUS	VIRIDESCENS	EASTERN	NEWT	LIV	3	NO	US	CA	T	W	C	C	4/5/10	4/5/10	I	BN	Science Kit, Inc - Boreal
2010580736	CYOR	CYNOPS	ORIENTALIS	CHINESE DWARF	NEWT	LIV	4,000	NO	CN	CN	T	W	C	C	4/6/10	4/6/10	I	LA	Z IMPORTS INC.
2010581635	CYPY	CYNOPS	PYRRHOGASTER	JAPANESE FIREBELLY	NEWT	LIV	130	NO	HK	HK	T	W	C	C	4/7/10	4/4/10	I	NY	ERDA INCORPORATED
2010582316	CYPY	CYNOPS	PYRRHOGASTER	JAPANESE FIREBELLY	NEWT	LIV	120	NO	HK	HK	T	C	C	C	4/8/10	4/4/10	I	NY	Transship Discounts Ltd.
2010582486	AMBA	AMBYSTOMA	MACULATUM	SPOTTED	SALAMANDER	LIV	140	NO	CA	CA	T	W	C	C	4/8/10	4/7/10	I	BN	Science Kit, Inc - Boreal
2010583490	CNP?	CYNOPS	SPECIES	FIREBELLY	NEWT	LIV	200	NO	CN	CN	T	C	C	C	4/10/10	4/10/10	I	AT	SUN PET LTD.
2010583498	CYOR	CYNOPS	ORIENTALIS	CHINESE DWARF	NEWT	LIV	800	NO	HK	HK	T	C	C	C	4/10/10	4/10/10	I	LA	DOLPHIN INTERNATIONAL
2010583498	PACB	PACHYTRITON	BREVIPES	TSITOU	NEWT	LIV	150	NO	HK	HK	T	C	C	C	4/10/10	4/10/10	I	LA	DOLPHIN INTERNATIONAL
2010583582	TRIH	TRITURUS	HONGKONGENSIS	ALPINE	NEWT	LIV	250	NO	HK	HK	T	C	C	C	4/10/10	4/11/10	I	LA	AQUACO, INC.
2010583603	CYOR	CYNOPS	ORIENTALIS	CHINESE DWARF	NEWT	LIV	50	NO	HK	HK	T	C	C	C	4/11/10	4/11/10	I	LA	DOLPHIN INTERNATIONAL
2010583613	CYOR	CYNOPS	ORIENTALIS	CHINESE DWARF	NEWT	LIV	900	NO	CN	CN	T	W	C	C	4/11/10	4/11/10	I	LA	RENAISSANCE AQUATICS INC
2010583613	TRIT	TRITURUS	SPECIES		NEWT	LIV	100	NO	CN	CN	T	W	C	C	4/11/10	4/11/10	I	LA	RENAISSANCE AQUATICS INC
2010583613	PLAB	PACHYTRITON	LABIATUS	UNTERSTEIN'S	NEWT	LIV	45	NO	CN	CN	T	W	C	C	4/11/10	4/11/10	I	LA	RENAISSANCE AQUATICS INC
2010584242	NOVI	NOTOPHTHALMUS	VIRIDESCENS	EASTERN	NEWT	LIV	2	NO	US	CA	T	W	C	C	4/12/10	4/12/10	I	BN	Science Kit, Inc - Boreal
2010585981	TRIH	TRITURUS	HONGKONGENSIS	ALPINE	NEWT	LIV	1,600	NO	HK	HK	T	C	C	C	4/14/10	4/14/10	I	TP	5-D TROPICAL INC.
2010585989	NOVI	NOTOPHTHALMUS	VIRIDESCENS	EASTERN	NEWT	LIV	2	NO	US	CA	T	W	C	C	4/14/10	4/14/10	I	BN	Science Kit, Inc - Boreal
2010587702	CYPY	CYNOPS	PYRRHOGASTER	JAPANESE FIREBELLY	NEWT	LIV	120	NO	HK	HK	T	C	C	C	4/16/10	4/11/10	I	NY	Transship Discounts Ltd.
2010587926	CNP?	CYNOPS	SPECIES	FIREBELLY	NEWT	LIV	200	NO	CN	CN	T	C	C	C	4/17/10	4/17/10	I	AT	SUN PET LTD.
2010587935	PLAO	PARAMESOTRITON	LAOENSIS	WARTY	NEWT	LIV	15	NO	DE	DE	P	C	C	C	4/15/10	4/15/10	I	CH	Exemptions 6 and 7(C)
2010587935	SASA	SALAMANDRA	SALAMANDRA	EUROPEAN FIRE	SALAMANDER	LIV	9	NO	DE	DE	P	C	C	C	4/15/10	4/15/10	I	CH	Exemptions 6 and 7(C)
2010587935	PRAN	PROTEUS	ANGUINUS		OLM	LIV	3	NO	DE	DE	P	C	C	C	4/15/10	4/15/10	I	CH	Exemptions 6 and 7(C)
2010587984	CYOR	CYNOPS	ORIENTALIS	CHINESE DWARF	NEWT	LIV	125	NO	HK	HK	T	C	C	C	4/18/10	4/18/10	I	LA	DOLPHIN INTERNATIONAL

2010587984	PACB	PACHYTRITON	BREVIPES	TSITOU	NEWT	LIV	150	NO	HK	HK	T	C	C	C	4/18/10	4/18/10	I	LA	DOLPHIN INTERNATIONAL
2010588662	CYOR	CYNOPS	ORIENTALIS	CHINESE DWARF	NEWT	LIV	1,320	NO	VS	CN	T	W	C	C	4/19/10	4/18/10	I	LA	RENAISSANCE AQUATICS INC
2010588662	PLAB	PACHYTRITON	LABIATUS	UNTERSTEIN'S	NEWT	LIV	45	NO	VS	CN	T	W	C	C	4/19/10	4/18/10	I	LA	RENAISSANCE AQUATICS INC
2010588662	TRIT	TRITURUS	SPECIES		NEWT	LIV	200	NO	VS	CN	T	W	C	C	4/19/10	4/18/10	I	LA	RENAISSANCE AQUATICS INC
2010588815	NOVI	NOTOPHTHALMUS	VIRIDESCENS	EASTERN	NEWT	LIV	5	NO	US	CA	T	W	C	C	4/19/10	4/19/10	I	BN	Science Kit, Inc - Boreal
2010589962	PACB	PACHYTRITON	BREVIPES	TSITOU	NEWT	LIV	20	NO	HK	HK	T	C	C	C	4/21/10	4/18/10	I	NY	Transship Discounts Ltd.
2010591144	TRIH	TRITURUS	HONGKONGENSIS	ALPINE	NEWT	LIV	1,600	NO	HK	HK	T	C	C	C	4/22/10	4/21/10	I	TP	5-D TROPICAL INC.
2010591601	CNP?	CYNOPS	SPECIES	FIREBELLY	NEWT	LIV	200	NO	CN	CN	T	C	C	C	4/24/10	4/24/10	I	AT	SUN PET LTD.
2010591680	TRIH	TRITURUS	HONGKONGENSIS	ALPINE	NEWT	LIV	250	NO	HK	HK	T	C	C	C	4/24/10	4/25/10	I	LA	AQUACO, INC.
2010591691	CYOR	CYNOPS	ORIENTALIS	CHINESE DWARF	NEWT	LIV	1,320	NO	CN	CN	T	W	C	C	4/24/10	4/25/10	I	LA	RENAISSANCE AQUATICS INC
2010591691	TRIT	TRITURUS	SPECIES		NEWT	LIV	200	NO	CN	CN	T	W	C	C	4/24/10	4/25/10	I	LA	RENAISSANCE AQUATICS INC
2010591714	CYOR	CYNOPS	ORIENTALIS	CHINESE DWARF	NEWT	LIV	750	NO	HK	HK	T	C	C	C	4/25/10	4/25/10	I	LA	DOLPHIN INTERNATIONAL
2010591714	PACB	PACHYTRITON	BREVIPES	TSITOU	NEWT	LIV	200	NO	HK	HK	T	C	C	C	4/25/10	4/25/10	I	LA	DOLPHIN INTERNATIONAL
2010592390	NOVI	NOTOPHTHALMUS	VIRIDESCENS	EASTERN	NEWT	LIV	3	NO	US	CA	T	W	C	C	4/26/10	4/26/10	I	BN	Science Kit, Inc - Boreal
2010592799	TRIH	TRITURUS	HONGKONGENSIS	ALPINE	NEWT	LIV	375	NO	HK	HK	T	C	C	C	10/25/09	10/25/09	I	LA	AQUACO, INC.
2010593532	CYPY	CYNOPS	PYRRHOGASTER	JAPANESE FIREBELLY	NEWT	LIV	105	NO	HK	HK	T	C	C	C	4/28/10	4/25/10	I	NY	Transship Discounts Ltd.
2010593602	CYPY	CYNOPS	PYRRHOGASTER	JAPANESE FIREBELLY	NEWT	LIV	250	NO	HK	HK	T	W	C	C	4/28/10	4/18/10	I	NY	ERDA INCORPORATED
2010594162	CYPY	CYNOPS	PYRRHOGASTER	JAPANESE FIREBELLY	NEWT	LIV	250	NO	HK	HK	T	W	C	C	4/29/10	4/25/10	I	NY	ERDA INCORPORATED
2010595421	CNP?	CYNOPS	SPECIES	FIREBELLY	NEWT	LIV	200	NO	CN	CN	T	C	C	C	5/1/10	5/1/10	I	AT	SUN PET LTD.
2010595461	PLAB	PACHYTRITON	LABIATUS	UNTERSTEIN'S	NEWT	LIV	200	NO	VS	CN	T	W	C	C	5/1/10	5/2/10	I	LA	RENAISSANCE AQUATICS INC
2010595461	TRIT	TRITURUS	SPECIES		NEWT	LIV	100	NO	VS	CN	T	W	C	C	5/1/10	5/2/10	I	LA	RENAISSANCE AQUATICS INC
2010595482	CYOR	CYNOPS	ORIENTALIS	CHINESE DWARF	NEWT	LIV	575	NO	HK	HK	T	C	C	C	5/2/10	5/2/10	I	LA	DOLPHIN INTERNATIONAL
2010595843	NOVI	NOTOPHTHALMUS	VIRIDESCENS	EASTERN	NEWT	LIV	2	NO	US	CA	T	W	C	C	5/3/10	5/3/10	I	BN	Science Kit, Inc - Boreal
2010595883	CNP?	CYNOPS	SPECIES	FIREBELLY	NEWT	LIV	220	NO	HK	HK	T	W	C	C	5/3/10	4/11/10	I	NY	ERDA INCORPORATED
2010596339	CYPY	CYNOPS	PYRRHOGASTER	JAPANESE FIREBELLY	NEWT	LIV	30	NO	HK	HK	T	C	C	C	5/4/10	5/2/10	I	NY	Transship Discounts Ltd.
2010596339	PACB	PACHYTRITON	BREVIPES	TSITOU	NEWT	LIV	210	NO	HK	HK	T	C	C	C	5/4/10	5/2/10	I	NY	Transship Discounts Ltd.
2010598627	TRIH	TRITURUS	HONGKONGENSIS	ALPINE	NEWT	LIV	56	NO	HK	HK	T	C	C	C	5/9/10	5/9/10	I	LA	AQUACO, INC.
2010598638	CYOR	CYNOPS	ORIENTALIS	CHINESE DWARF	NEWT	LIV	2,080	NO	CN	CN	T	W	C	C	5/9/10	5/9/10	I	LA	RENAISSANCE AQUATICS INC
2010598638	PLAB	PACHYTRITON	LABIATUS	UNTERSTEIN'S	NEWT	LIV	45	NO	CN	CN	T	W	C	C	5/9/10	5/9/10	I	LA	RENAISSANCE AQUATICS INC
2010598667	CYOR	CYNOPS	ORIENTALIS	CHINESE DWARF	NEWT	LIV	925	NO	HK	HK	T	C	C	C	5/9/10	5/9/10	I	LA	DOLPHIN INTERNATIONAL
2010598667	PACB	PACHYTRITON	BREVIPES	TSITOU	NEWT	LIV	100	NO	HK	HK	T	C	C	C	5/9/10	5/9/10	I	LA	DOLPHIN INTERNATIONAL
2010599167	NOVI	NOTOPHTHALMUS	VIRIDESCENS	EASTERN	NEWT	LIV	6	NO	US	CA	T	W	C	C	5/10/10	5/10/10	I	BN	Science Kit, Inc - Boreal
2010599892	CYPY	CYNOPS	PYRRHOGASTER	JAPANESE FIREBELLY	NEWT	LIV	60	NO	HK	HK	T	C	C	C	5/11/10	5/9/10	I	NY	Transship Discounts Ltd.
2010600300	CYPY	CYNOPS	PYRRHOGASTER	JAPANESE FIREBELLY	NEWT	LIV	690	NO	HK	HK	T	W	C	C	5/12/10	5/9/10	I	NY	ERDA INCORPORATED
2010600479	SAL?	SALAMANDRA	SPECIES	FIRE	SALAMANDER	LIV	50	NO	UA	UA	T	W	C	C	5/12/10	5/12/10	I	LA	NAME REMOVED
2010600845	TRIH	TRITURUS	HONGKONGENSIS	ALPINE	NEWT	LIV	1,600	NO	HK	HK	T	C	C	C	5/13/10	5/12/10	I	TP	5-D TROPICAL INC.
2010601523	CYOR	CYNOPS	ORIENTALIS	CHINESE DWARF	NEWT	LIV	1,000	NO	CN	CN	T	W	C	C	5/14/10	5/14/10	I	LA	WORLDWIDE TRADING INC
2010601849	CNP?	CYNOPS	SPECIES	FIREBELLY	NEWT	LIV	200	NO	CN	CN	T	C	C	C	5/15/10	5/15/10	I	AT	SUN PET LTD.
2010601949	TRIH	TRITURUS	HONGKONGENSIS	ALPINE	NEWT	LIV	200	NO	HK	HK	T	C	C	C	5/15/10	5/16/10	I	LA	AQUACO, INC.
2010601960	CYOR	CYNOPS	ORIENTALIS	CHINESE DWARF	NEWT	LIV	1,150	NO	HK	HK	T	C	C	C	5/15/10	5/16/10	I	LA	DOLPHIN INTERNATIONAL
2010601960	PACB	PACHYTRITON	BREVIPES	TSITOU	NEWT	LIV	100	NO	HK	HK	T	C	C	C	5/15/10	5/16/10	I	LA	DOLPHIN INTERNATIONAL
2010602009	TRIH	TRITURUS	HONGKONGENSIS	ALPINE	NEWT	LIV	100	NO	VS	CN	T	W	C	C	5/16/10	5/16/10	I	LA	RENAISSANCE AQUATICS INC
2010602591	NOVI	NOTOPHTHALMUS	VIRIDESCENS	EASTERN	NEWT	LIV	7	NO	US	CA	T	W	C	C	5/17/10	5/17/10	I	BN	Science Kit, Inc - Boreal
2010603337	CYPY	CYNOPS	PYRRHOGASTER	JAPANESE FIREBELLY	NEWT	LIV	135	NO	HK	HK	T	C	C	C	5/18/10	5/16/10	I	NY	Transship Discounts Ltd.
2010603337	PACB	PACHYTRITON	BREVIPES	TSITOU	NEWT	LIV	30	NO	HK	HK	T	C	C	C	5/18/10	5/16/10	I	NY	Transship Discounts Ltd.
2010603398	CYPY	CYNOPS	PYRRHOGASTER	JAPANESE FIREBELLY	NEWT	LIV	160	NO	HK	HK	T	W	C	C	5/18/10	5/16/10	I	NY	ERDA INCORPORATED
2010603994	TRIH	TRITURUS	HONGKONGENSIS	ALPINE	NEWT	LIV	2,100	NO	HK	HK	T	C	C	C	5/19/10	5/19/10	I	TP	5-D TROPICAL INC.
2010605383	CNP?	CYNOPS	SPECIES	FIREBELLY	NEWT	LIV	200	NO	CN	CN	T	C	C	C	5/22/10	5/22/10	I	AT	SUN PET LTD.
2010605475	TRIH	TRITURUS	HONGKONGENSIS	ALPINE	NEWT	LIV	425	NO	HK	HK	T	C	C	C	5/22/10	5/23/10	I	LA	AQUACO, INC.
2010605528	CYOR	CYNOPS	ORIENTALIS	CHINESE DWARF	NEWT	LIV	375	NO	HK	HK	T	C	C	C	5/23/10	5/23/10	I	LA	DOLPHIN INTERNATIONAL
2010605547	TRIT	TRITURUS	SPECIES		NEWT	LIV	645	NO	VS	CN	T	W	C	C	5/23/10	5/23/10	I	LA	RENAISSANCE AQUATICS INC
2010606155	CYPY	CYNOPS	PYRRHOGASTER	JAPANESE FIREBELLY	NEWT	LIV	150	NO	HK	HK	T	W	C	C	5/24/10	5/23/10	I	NY	ERDA INCORPORATED
2010607599	TRIH	TRITURUS	HONGKONGENSIS	ALPINE	NEWT	LIV	500	NO	HK	HK	T	C	C	C	5/26/10	5/26/10	I	TP	5-D TROPICAL INC.
2010607827	NOVI	NOTOPHTHALMUS	VIRIDESCENS	EASTERN	NEWT	LIV	2	NO	US	CA	T	W	C	C	5/26/10	5/26/10	I	BN	Science Kit, Inc - Boreal
2010609488	CNP?	CYNOPS	SPECIES	FIREBELLY	NEWT	LIV	200	NO	CN	CN	T	C	C	C	5/29/10	5/29/10	I	AT	SUN PET LTD.
2010609605	TRIT	TRITURUS	SPECIES		NEWT	LIV	200	NO	VS	CN	T	W	C	C	5/30/10	5/30/10	I	LA	RENAISSANCE AQUATICS INC
2010610756	TRIH	TRITURUS	HONGKONGENSIS	ALPINE	NEWT	LIV	500	NO	HK	HK	T	C	C	C	6/2/10	6/2/10	I	TP	5-D TROPICAL INC.
2010611098	CYOR	CYNOPS	ORIENTALIS	CHINESE DWARF	NEWT	LIV	494	NO	HK	HK	T	C	C	C	6/2/10	6/2/10	I	CH	AQUATICS INC
2010613188	CYOR	CYNOPS	ORIENTALIS	CHINESE DWARF	NEWT	LIV	800	NO	HK	HK	T	C	C	C	6/6/10	6/6/10	I	LA	DOLPHIN INTERNATIONAL
2010613188	PACB	PACHYTRITON	BREVIPES	TSITOU	NEWT	LIV	100	NO	HK	HK	T	C	C	C	6/6/10	6/6/10	I	LA	DOLPHIN INTERNATIONAL

2010613212	TRIH	TRITURUS	HONGKONGENSIS	ALPINE	NEWT	LIV	300	NO	HK	HK	T	C	C	C	6/6/10	6/6/10	I	LA	AQUACO, INC.
2010613219	TRIH	TRITURUS	HONGKONGENSIS	ALPINE	NEWT	LIV	45	NO	VS	CN	T	W	C	C	6/6/10	6/6/10	I	LA	ALL MARINE TRANSHIPPING INC
2010613262	CYPY	CYNOPS	PYRRHOGASTER	JAPANESE FIREBELLY	NEWT	LIV	210	NO	HK	HK	T	W	C	C	6/7/10	5/30/10	I	NY	ERDA INCORPORATED
2010613270	CYPY	CYNOPS	PYRRHOGASTER	JAPANESE FIREBELLY	NEWT	LIV	135	NO	HK	HK	T	C	C	C	6/7/10	5/30/10	I	NY	Transship Discounts Ltd.
2010613674	NOVI	NOTOPHTHALMUS	VIRIDESCENS	EASTERN	NEWT	LIV	1	NO	US	CA	T	W	C	C	6/7/10	6/7/10	I	BN	Science Kit, Inc - Boreal
2010614461	NEW?	PACHYTRITON	SPECIES	CHINESE	NEWT	LIV	1,000	NO	HK	HK	T	W	C	C	6/8/10	6/8/10	I	LA	WORLDWIDE TRADING INC
2010615272	TRIH	TRITURUS	HONGKONGENSIS	ALPINE	NEWT	LIV	1,600	NO	HK	HK	T	C	C	C	6/9/10	6/9/10	I	TP	5-D TROPICAL INC.
2010615814	CYPY	CYNOPS	PYRRHOGASTER	JAPANESE FIREBELLY	NEWT	LIV	150	NO	HK	HK	T	W	C	C	6/10/10	6/6/10	I	NY	ERDA INCORPORATED
2010616065	CYPY	CYNOPS	PYRRHOGASTER	JAPANESE FIREBELLY	NEWT	LIV	60	NO	HK	HK	T	C	C	C	6/11/10	6/6/10	I	NY	Transship Discounts Ltd.
2010616649	TRIH	TRITURUS	HONGKONGENSIS	ALPINE	NEWT	LIV	219	NO	HK	HK	T	C	C	C	6/13/10	6/13/10	I	LA	AQUACO, INC.
2010616679	CYOR	CYNOPS	ORIENTALIS	CHINESE DWARF	NEWT	LIV	1,250	NO	HK	HK	T	C	C	C	6/13/10	6/13/10	I	LA	DOLPHIN INTERNATIONAL
2010616679	PACB	PACHYTRITON	BREVIPES	TSITOU	NEWT	LIV	150	NO	HK	HK	T	C	C	C	6/13/10	6/13/10	I	LA	DOLPHIN INTERNATIONAL
2010616721	CNP?	CYNOPS	SPECIES	FIREBELLY	NEWT	LIV	1,040	NO	CN	CN	T	W	C	C	6/13/10	6/13/10	I	LA	ALL MARINE TRANSHIPPING INC
2010616721	TRIT	TRITURUS	SPECIES		NEWT	LIV	100	NO	CN	CN	T	W	C	C	6/13/10	6/13/10	I	LA	ALL MARINE TRANSHIPPING INC
2010616721	PLAB	PACHYTRITON	LABIATUS	UNTERSTEIN'S	NEWT	LIV	100	NO	CN	CN	T	W	C	C	6/13/10	6/13/10	I	LA	ALL MARINE TRANSHIPPING INC
2010618272	CYPY	CYNOPS	PYRRHOGASTER	JAPANESE FIREBELLY	NEWT	LIV	150	NO	HK	HK	T	W	C	C	6/16/10	6/13/10	I	NY	ERDA INCORPORATED
2010618514	CYPY	CYNOPS	PYRRHOGASTER	JAPANESE FIREBELLY	NEWT	LIV	135	NO	HK	HK	T	C	C	C	6/16/10	6/13/10	I	NY	Transship Discounts Ltd.
2010618514	PACB	PACHYTRITON	BREVIPES	TSITOU	NEWT	LIV	90	NO	HK	HK	T	C	C	C	6/16/10	6/13/10	I	NY	Transship Discounts Ltd.
2010618528	TRIH	TRITURUS	HONGKONGENSIS	ALPINE	NEWT	LIV	1,600	NO	HK	HK	T	C	C	C	6/16/10	6/16/10	I	TP	5-D TROPICAL INC.
2010620178	CNP?	CYNOPS	SPECIES	FIREBELLY	NEWT	LIV	200	NO	CN	CN	T	C	C	C	6/18/10	6/19/10	I	AT	SUN PET LTD.
2010620315	CYOR	CYNOPS	ORIENTALIS	CHINESE DWARF	NEWT	LIV	375	NO	HK	HK	T	C	C	C	6/20/10	6/20/10	I	LA	DOLPHIN INTERNATIONAL
2010620318	TRIH	TRITURUS	HONGKONGENSIS	ALPINE	NEWT	LIV	400	NO	HK	HK	T	C	C	C	6/20/10	6/20/10	I	LA	AQUACO, INC.
2010620332	CNP?	CYNOPS	SPECIES	FIREBELLY	NEWT	LIV	900	NO	VS	CN	T	W	C	C	6/20/10	6/20/10	I	LA	ALL MARINE TRANSHIPPING INC
2010620332	PLAB	PACHYTRITON	LABIATUS	UNTERSTEIN'S	NEWT	LIV	45	NO	VS	CN	T	W	C	C	6/20/10	6/20/10	I	LA	ALL MARINE TRANSHIPPING INC
2010620497	CYOR	CYNOPS	ORIENTALIS	CHINESE DWARF	NEWT	LIV	100	NO	SG	SG	T	C	C	C	2/28/10	2/28/10	I	NY	INTERNATIONAL PET RESOURCES , LLC
2010620515	CYOR	CYNOPS	ORIENTALIS	CHINESE DWARF	NEWT	LIV	100	NO	SG	SG	T	W	C	C	2/2/10	1/31/10	I	NY	INTERNATIONAL PET RESOURCES , LLC
2010620808	NOVI	NOTOPHTHALMUS	VIRIDESCENS	EASTERN	NEWT	LIV	1	NO	US	CA	T	W	C	C	6/21/10	6/21/10	I	BN	Science Kit, Inc - Boreal
2010621220	CYOR	CYNOPS	ORIENTALIS	CHINESE DWARF	NEWT	LIV	100	NO	SG	SG	T	W	C	C	1/28/10	1/17/10	I	NY	INTERNATIONAL PET RESOURCES , LLC
2010621255	CYOR	CYNOPS	ORIENTALIS	CHINESE DWARF	NEWT	LIV	150	NO	MY	MY	T	C	C	C	7/7/09	7/6/09	I	NY	TRI-COUNTY TROPICALS, INC.
2010621290	CYOR	CYNOPS	ORIENTALIS	CHINESE DWARF	NEWT	LIV	0	NO	SG	SG	T	W	C	C	4/12/10	1/3/10	I	NY	INTERNATIONAL PET RESOURCES , LLC
2010621557	CYOR	CYNOPS	ORIENTALIS	CHINESE DWARF	NEWT	LIV	100	NO	SG	SG	T	C	C	C	12/27/09	12/27/09	I	NY	INTERNATIONAL PET RESOURCES , LLC
2010621640	CYOR	CYNOPS	ORIENTALIS	CHINESE DWARF	NEWT	LIV	0	NO	SG	SG	T	W	C	C	1/11/10	1/3/10	I	NY	INTERNATIONAL PET RESOURCES , LLC
2010621679	CYOR	CYNOPS	ORIENTALIS	CHINESE DWARF	NEWT	LIV	100	NO	SG	SG	T	W	C	C	2/14/10	2/14/10	I	NY	INTERNATIONAL PET RESOURCES , LLC
2010621681	CYOR	CYNOPS	ORIENTALIS	CHINESE DWARF	NEWT	LIV	100	NO	SG	SG	T	W	C	C	3/7/10	3/7/10	I	NW	INTERNATIONAL PET RESOURCES , LLC
2010621811	CYPY	CYNOPS	PYRRHOGASTER	JAPANESE FIREBELLY	NEWT	LIV	60	NO	HK	HK	T	W	C	C	6/22/10	6/20/10	I	NY	ERDA INCORPORATED
2010621854	CYPY	CYNOPS	PYRRHOGASTER	JAPANESE FIREBELLY	NEWT	LIV	135	NO	HK	HK	T	C	C	C	6/22/10	6/20/10	I	NY	Transship Discounts Ltd.
2010622417	TRIH	TRITURUS	HONGKONGENSIS	ALPINE	NEWT	LIV	1,600	NO	HK	HK	T	C	C	C	6/23/10	6/23/10	I	TP	5-D TROPICAL INC.
2010623850	TRIH	TRITURUS	HONGKONGENSIS	ALPINE	NEWT	LIV	100	NO	HK	HK	T	C	C	C	6/26/10	6/27/10	I	LA	AQUACO, INC.
2010623903	CYOR	CYNOPS	ORIENTALIS	CHINESE DWARF	NEWT	LIV	250	NO	HK	HK	T	C	C	C	6/27/10	6/27/10	I	LA	DOLPHIN INTERNATIONAL
2010623903	PACB	PACHYTRITON	BREVIPES	TSITOU	NEWT	LIV	50	NO	HK	HK	T	C	C	C	6/27/10	6/27/10	I	LA	DOLPHIN INTERNATIONAL
2010624076	CYPY	CYNOPS	PYRRHOGASTER	JAPANESE FIREBELLY	NEWT	LIV	130	NO	HK	HK	T	W	C	C	6/28/10	6/27/10	I	NY	ERDA INCORPORATED
2010624417	NOVI	NOTOPHTHALMUS	VIRIDESCENS	EASTERN	NEWT	LIV	2	NO	US	CA	T	W	C	C	6/28/10	6/28/10	I	BN	Science Kit, Inc - Boreal
2010624496	CYPY	CYNOPS	PYRRHOGASTER	JAPANESE FIREBELLY	NEWT	LIV	60	NO	HK	HK	T	C	C	C	6/28/10	6/27/10	I	NY	Transship Discounts Ltd.
2010624496	PACB	PACHYTRITON	BREVIPES	TSITOU	NEWT	LIV	60	NO	HK	HK	T	C	C	C	6/28/10	6/27/10	I	NY	Transship Discounts Ltd.
2010625691	CYOR	CYNOPS	ORIENTALIS	CHINESE DWARF	NEWT	LIV	290	NO	HK	HK	T	C	C	C	6/30/10	6/30/10	I	CH	AQUATICS INC
2010626211	PDE?	PSEUDOEURYCEA	SPECIES	FALSE BROOK	SALAMANDER	LIV	45	NO	MX	MX	S	W	C	C	6/30/10	6/23/10	I	LO	CORNELL UNIVERSITY - ZAMUDIO LAB
2010626282	TRIH	TRITURUS	HONGKONGENSIS	ALPINE	NEWT	LIV	2,100	NO	HK	HK	T	C	C	C	6/30/10	6/30/10	I	TP	5-D TROPICAL INC.
2010627626	CYOR	CYNOPS	ORIENTALIS	CHINESE DWARF	NEWT	LIV	1,300	NO	CN	CN	T	W	C	C	7/2/10	7/2/10	I	LA	WORLDWIDE TRADING INC
2010627626	PLAB	PACHYTRITON	LABIATUS	UNTERSTEIN'S	NEWT	LIV	500	NO	CN	CN	T	W	C	C	7/2/10	7/2/10	I	LA	WORLDWIDE TRADING INC
2010628060	CYOR	CYNOPS	ORIENTALIS	CHINESE DWARF	NEWT	LIV	750	NO	HK	HK	T	C	C	C	7/4/10	7/4/10	I	LA	DOLPHIN INTERNATIONAL
2010628060	PACB	PACHYTRITON	BREVIPES	TSITOU	NEWT	LIV	100	NO	HK	HK	T	C	C	C	7/4/10	7/4/10	I	LA	DOLPHIN INTERNATIONAL
2010628072	CYOR	CYNOPS	ORIENTALIS	CHINESE DWARF	NEWT	LIV	2,840	NO	VS	CN	T	W	C	C	7/4/10	7/4/10	I	LA	ALL MARINE TRANSHIPPING INC
2010629305	TRIH	TRITURUS	HONGKONGENSIS	ALPINE	NEWT	LIV	1,600	NO	HK	HK	T	C	C	C	7/7/10	7/7/10	I	TP	5-D TROPICAL INC.
2010631061	CYPY	CYNOPS	PYRRHOGASTER	JAPANESE FIREBELLY	NEWT	LIV	70	NO	HK	HK	T	W	C	C	7/9/10	7/4/10	I	NY	ERDA INCORPORATED
2010631288	CYOR	CYNOPS	ORIENTALIS	CHINESE DWARF	NEWT	LIV	740	NO	VS	CN	T	W	C	C	7/11/10	7/11/10	I	LA	ALL MARINE TRANSHIPPING INC
2010631288	TRIT	TRITURUS	SPECIES		NEWT	LIV	100	NO	VS	CN	T	W	C	C	7/11/10	7/11/10	I	LA	ALL MARINE TRANSHIPPING INC
2010631304	CYOR	CYNOPS	ORIENTALIS	CHINESE DWARF	NEWT	LIV	250	NO	HK	HK	T	C	C	C	7/11/10	7/11/10	I	LA	DOLPHIN INTERNATIONAL
2010631304	PACB	PACHYTRITON	BREVIPES	TSITOU	NEWT	LIV	300	NO	HK	HK	T	C	C	C	7/11/10	7/11/10	I	LA	DOLPHIN INTERNATIONAL
2010631323	CYPY	CYNOPS	PYRRHOGASTER	JAPANESE FIREBELLY	NEWT	LIV	150	NO	HK	HK	T	W	C	C	7/11/10	7/11/10	I	NY	ERDA INCORPORATED
2010632443	CYOR	CYNOPS	ORIENTALIS	CHINESE DWARF	NEWT	LIV	100	NO	SG	SG	T	W	C	C	9/20/09	9/20/09	I	NY	INTERNATIONAL PET RESOURCES , LLC

2010632464	CYOR	CYNOPS	ORIENTALIS	CHINESE DWARF	NEWT	LIV	100	NO	SG	SG	T	W	C	C	11/16/09	9/6/09	I	NY	INTERNATIONAL PET RESOURCES , LLC
2010632732	CYOR	CYNOPS	ORIENTALIS	CHINESE DWARF	NEWT	LIV	0	NO	XX	SG	T	W	C	C	10/23/09	10/18/09	I	NY	INTERNATIONAL PET RESOURCES , LLC
2010632744	CYOR	CYNOPS	ORIENTALIS	CHINESE DWARF	NEWT	LIV	100	NO	SG	SG	T	W	C	C	11/8/09	11/8/09	I	NY	INTERNATIONAL PET RESOURCES , LLC
2010632864	CYOR	CYNOPS	ORIENTALIS	CHINESE DWARF	NEWT	LIV	120	NO	SG	SG	T	W	C	C	12/13/09	12/13/09	I	NY	INTERNATIONAL PET RESOURCES , LLC
2010634187	TRIH	TRITURUS	HONGKONGENSIS	ALPINE	NEWT	LIV	2,500	NO	HK	HK	T	C	C	C	7/14/10	7/14/10	I	TP	5-D TROPICAL INC.
2010636147	TRIT	TRITURUS	SPECIES		NEWT	LIV	100	NO	VS	CN	T	W	C	C	7/17/10	7/18/10	I	LA	ALL MARINE TRANSHIPPING INC
2010636147	CNP?	CYNOPS	SPECIES	FIREBELLY	NEWT	LIV	300	NO	VS	CN	T	W	C	C	7/17/10	7/18/10	I	LA	ALL MARINE TRANSHIPPING INC
2010636193	CYOR	CYNOPS	ORIENTALIS	CHINESE DWARF	NEWT	LIV	1,000	NO	HK	HK	T	C	C	C	7/18/10	7/18/10	I	LA	DOLPHIN INTERNATIONAL
2010636193	PACB	PACHYTRITON	BREVIPES	TSITOU	NEWT	LIV	100	NO	HK	HK	T	C	C	C	7/18/10	7/18/10	I	LA	DOLPHIN INTERNATIONAL
2010636368	CYPY	CYNOPS	PYRRHOGASTER	JAPANESE FIREBELLY	NEWT	LIV	165	NO	HK	HK	T	C	C	C	7/19/10	7/11/10	I	NY	Transship Discounts Ltd.
2010636478	CYPY	CYNOPS	PYRRHOGASTER	JAPANESE FIREBELLY	NEWT	LIV	280	NO	HK	HK	T	W	C	C	7/19/10	7/18/10	I	NY	ERDA INCORPORATED
2010637870	TRIH	TRITURUS	HONGKONGENSIS	ALPINE	NEWT	LIV	2,000	NO	HK	HK	T	C	C	C	7/21/10	7/21/10	I	TP	5-D TROPICAL INC.
2010639881	CYPY	CYNOPS	PYRRHOGASTER	JAPANESE FIREBELLY	NEWT	LIV	200	NO	CN	CN	T	C	C	C	7/24/10	7/24/10	I	AT	SUN PET LTD.
2010639947	TRIH	TRITURUS	HONGKONGENSIS	ALPINE	NEWT	LIV	25	NO	HK	HK	T	C	C	C	7/24/10	7/25/10	I	LA	AQUACO, INC.
2010639960	TRIT	TRITURUS	SPECIES		NEWT	LIV	200	NO	VS	CN	T	W	C	C	7/24/10	7/25/10	I	LA	ALL MARINE TRANSHIPPING INC
2010640025	CYOR	CYNOPS	ORIENTALIS	CHINESE DWARF	NEWT	LIV	375	NO	HK	HK	T	C	C	C	7/25/10	7/25/10	I	LA	DOLPHIN INTERNATIONAL
2010640546	CYPY	CYNOPS	PYRRHOGASTER	JAPANESE FIREBELLY	NEWT	LIV	150	NO	HK	HK	T	W	C	C	7/26/10	7/25/10	I	NY	ERDA INCORPORATED
2010640573	CYPY	CYNOPS	PYRRHOGASTER	JAPANESE FIREBELLY	NEWT	LIV	210	NO	HK	HK	T	C	C	C	7/26/10	7/25/10	I	NY	Transship Discounts Ltd.
2010641604	AMBM	AMBYSTOMA	MEXICANUM		AXOLOTL	LIV	40	NO	US	MX	S	W	C	C	8/20/10	8/20/10	I	NY	UNIVERSITY OF KENTUCKY
2010641604	AMBM	AMBYSTOMA	MEXICANUM		AXOLOTL	LIV	20	NO	US	MX	S	C	C	C	8/20/10	8/20/10	I	NY	UNIVERSITY OF KENTUCKY
2010641604	AMBM	AMBYSTOMA	MEXICANUM		AXOLOTL	LIV	5	NO	US	MX	S	C	C	C	8/20/10	8/20/10	I	NY	UNIVERSITY OF KENTUCKY
2010641604	AMBM	AMBYSTOMA	MEXICANUM		AXOLOTL	LIV	5	NO	US	MX	S	C	C	C	8/20/10	8/20/10	I	NY	UNIVERSITY OF KENTUCKY
2010643934	CYOR	CYNOPS	ORIENTALIS	CHINESE DWARF	NEWT	LIV	850	NO	HK	HK	T	C	C	C	8/1/10	8/1/10	I	LA	DOLPHIN INTERNATIONAL
2010643941	TRIH	TRITURUS	HONGKONGENSIS	ALPINE	NEWT	LIV	50	NO	HK	HK	T	C	C	C	8/1/10	8/1/10	I	LA	AQUACO, INC.
2010643947	CNP?	CYNOPS	SPECIES	FIREBELLY	NEWT	LIV	2,360	NO	VS	CN	T	W	C	C	8/1/10	8/1/10	I	LA	ALL MARINE TRANSHIPPING INC
2010644138	CYPY	CYNOPS	PYRRHOGASTER	JAPANESE FIREBELLY	NEWT	LIV	120	NO	HK	HK	T	W	C	C	8/2/10	8/1/10	I	NY	ERDA INCORPORATED
2010647355	CYPY	CYNOPS	PYRRHOGASTER	JAPANESE FIREBELLY	NEWT	LIV	200	NO	CN	CN	T	C	C	C	8/7/10	8/7/10	I	AT	SUN PET LTD.
2010647434	CYOR	CYNOPS	ORIENTALIS	CHINESE DWARF	NEWT	LIV	475	NO	HK	HK	T	C	C	C	8/8/10	8/8/10	I	LA	DOLPHIN INTERNATIONAL
2010647434	PACB	PACHYTRITON	BREVIPES	TSITOU	NEWT	LIV	100	NO	HK	HK	T	C	C	C	8/8/10	8/8/10	I	LA	DOLPHIN INTERNATIONAL
2010647439	TRIT	TRITURUS	SPECIES		NEWT	LIV	300	NO	VS	CN	T	W	C	C	8/8/10	8/8/10	I	LA	ALL MARINE TRANSHIPPING INC
2010647439	CYOR	CYNOPS	ORIENTALIS	CHINESE DWARF	NEWT	LIV	140	NO	VS	CN	T	W	C	C	8/8/10	8/8/10	I	LA	ALL MARINE TRANSHIPPING INC
2010648448	CYPY	CYNOPS	PYRRHOGASTER	JAPANESE FIREBELLY	NEWT	LIV	120	NO	HK	HK	T	W	C	C	8/10/10	8/8/10	I	NY	ERDA INCORPORATED
2010648819	CYOR	CYNOPS	ORIENTALIS	CHINESE DWARF	NEWT	LIV	800	NO	HK	HK	T	C	C	C	8/10/10	8/11/10	I	TP	5-D TROPICAL, INC.
2010649113	TRIH	TRITURUS	HONGKONGENSIS	ALPINE	NEWT	LIV	2,100	NO	HK	HK	T	C	C	C	8/11/10	8/11/10	I	TP	5-D TROPICAL INC.
2010649130	PACB	PACHYTRITON	BREVIPES	TSITOU	NEWT	LIV	60	NO	HK	HK	T	C	C	C	8/11/10	8/8/10	I	NY	Transship Discounts Ltd.
2010651077	TRIH	TRITURUS	HONGKONGENSIS	ALPINE	NEWT	LIV	50	NO	HK	HK	T	C	C	C	8/15/10	8/15/10	I	LA	AQUACO, INC.
2010651079	CNP?	CYNOPS	SPECIES	FIREBELLY	NEWT	LIV	300	NO	VS	CN	T	W	C	C	8/15/10	8/15/10	I	LA	ALL MARINE TRANSHIPPING INC
2010651079	TRIT	TRITURUS	SPECIES		NEWT	LIV	100	NO	VS	CN	T	W	C	C	8/15/10	8/15/10	I	LA	ALL MARINE TRANSHIPPING INC
2010651093	CYOR	CYNOPS	ORIENTALIS	CHINESE DWARF	NEWT	LIV	375	NO	HK	HK	T	C	C	C	8/15/10	8/15/10	I	LA	DOLPHIN INTERNATIONAL
2010651093	PACB	PACHYTRITON	BREVIPES	TSITOU	NEWT	LIV	100	NO	HK	HK	T	C	C	C	8/15/10	8/15/10	I	LA	DOLPHIN INTERNATIONAL
2010651132	CYPY	CYNOPS	PYRRHOGASTER	JAPANESE FIREBELLY	NEWT	LIV	400	NO	HK	HK	T	W	C	C	8/15/10	8/15/10	I	NY	ERDA INCORPORATED
2010652022	CYPY	CYNOPS	PYRRHOGASTER	JAPANESE FIREBELLY	NEWT	LIV	210	NO	HK	HK	T	C	C	C	8/17/10	8/15/10	I	NY	Transship Discounts Ltd.
2010652022	PACB	PACHYTRITON	BREVIPES	TSITOU	NEWT	LIV	30	NO	HK	HK	T	C	C	C	8/17/10	8/15/10	I	NY	Transship Discounts Ltd.
2010652733	TRIH	TRITURUS	HONGKONGENSIS	ALPINE	NEWT	LIV	1,600	NO	HK	HK	T	C	C	C	8/18/10	8/18/10	I	TP	5-D TROPICAL INC.
2010652806	SASA	SALAMANDRA	SALAMANDRA	EUROPEAN FIRE	SALAMANDER	LIV	2	NO	FR	FR	T	C	C	C	8/18/10	8/17/10	I	MI	TWO AMIGOS IMPORT & EXPORT, INC.
2010654456	CYOR	CYNOPS	ORIENTALIS	CHINESE DWARF	NEWT	LIV	625	NO	HK	HK	T	C	C	C	8/22/10	8/22/10	I	LA	DOLPHIN INTERNATIONAL
2010654467	TRIH	TRITURUS	HONGKONGENSIS	ALPINE	NEWT	LIV	50	NO	TH	HK	T	C	C	C	8/22/10	8/22/10	I	LA	AQUACO, INC.
2010654482	TRIT	TRITURUS	SPECIES		NEWT	LIV	100	NO	VS	CN	T	W	C	C	8/22/10	8/22/10	I	LA	ALL MARINE TRANSHIPPING INC
2010654482	CNP?	CYNOPS	SPECIES	FIREBELLY	NEWT	LIV	440	NO	VS	CN	T	W	C	C	8/22/10	8/22/10	I	LA	ALL MARINE TRANSHIPPING INC
2010654840	CYPY	CYNOPS	PYRRHOGASTER	JAPANESE FIREBELLY	NEWT	LIV	70	NO	HK	HK	T	W	C	C	8/23/10	8/22/10	I	NY	ERDA INCORPORATED
2010654847	CYPY	CYNOPS	PYRRHOGASTER	JAPANESE FIREBELLY	NEWT	LIV	246	NO	HK	HK	T	C	C	C	8/23/10	8/22/10	I	NY	Transship Discounts Ltd.
2010654847	PACB	PACHYTRITON	BREVIPES	TSITOU	NEWT	LIV	60	NO	HK	HK	T	C	C	C	8/23/10	8/22/10	I	NY	Transship Discounts Ltd.
2010655253	TRIH	TRITURUS	HONGKONGENSIS	ALPINE	NEWT	LIV	500	NO	HK	HK	T	C	C	C	8/24/10	8/24/10	I	TP	5-D TROPICAL INC.
2010656880	NOVI	NOTOPHTHALMUS	VIRIDESCENS	EASTERN	NEWT	LIV	9	NO	US	CA	T	W	C	C	8/26/10	8/25/10	I	BN	Science Kit, Inc - Boreal
2010657554	CYPY	CYNOPS	PYRRHOGASTER	JAPANESE FIREBELLY	NEWT	LIV	200	NO	CN	CN	T	C	C	C	8/28/10	8/28/10	I	AT	SUN PET LTD.
2010657655	TRIT	TRITURUS	SPECIES		NEWT	LIV	300	NO	VS	HK	T	W	C	C	8/29/10	8/29/10	I	LA	ALL MARINE TRANSHIPPING INC
2010657655	CNP?	CYNOPS	SPECIES	FIREBELLY	NEWT	LIV	300	NO	VS	HK	T	W	C	C	8/29/10	8/29/10	I	LA	ALL MARINE TRANSHIPPING INC
2010657676	CYOR	CYNOPS	ORIENTALIS	CHINESE DWARF	NEWT	LIV	125	NO	HK	HK	T	C	C	C	8/29/10	8/29/10	I	LA	DOLPHIN INTERNATIONAL
2010657676	PACB	PACHYTRITON	BREVIPES	TSITOU	NEWT	LIV	250	NO	HK	HK	T	C	C	C	8/29/10	8/29/10	I	LA	DOLPHIN INTERNATIONAL
2010657817	CYPY	CYNOPS	PYRRHOGASTER	JAPANESE FIREBELLY	NEWT	LIV	150	NO	HK	HK	T	W	C	C	8/30/10	8/29/10	I	NY	ERDA INCORPORATED

2010658088	PACB	PACHYTRITON	BREVIPES	TSITOU	NEWT	LIV	60	NO	HK	HK	T	C	C	C	8/30/10	8/29/10	I	NY	Transship Discounts Ltd.
2010659206	TRIH	TRITURUS	HONGKONGENSIS	ALPINE	NEWT	LIV	2,100	NO	HK	HK	T	C	C	C	9/1/10	9/1/10	I	TP	5-D TROPICAL INC.
2010660780	NOVI	NOTOPHTHALMUS	VIRIDESCENS	EASTERN	NEWT	LIV	7	NO	US	CA	T	W	C	C	8/30/10	8/30/10	I	BN	Science Kit, Inc - Boreal
2010661301	TRIH	TRITURUS	HONGKONGENSIS	ALPINE	NEWT	LIV	100	NO	HK	HK	T	C	C	C	9/4/10	9/5/10	I	LA	AQUACO, INC.
2010661318	CYOR	CYNOPS	ORIENTALIS	CHINESE DWARF	NEWT	LIV	750	NO	HK	HK	T	C	C	C	9/5/10	9/5/10	I	LA	DOLPHIN INTERNATIONAL
2010661318	PACB	PACHYTRITON	BREVIPES	TSITOU	NEWT	LIV	250	NO	HK	HK	T	C	C	C	9/5/10	9/5/10	I	LA	DOLPHIN INTERNATIONAL
2010661345	CYPY	CYNOPS	PYRRHOGASTER	JAPANESE FIREBELLY	NEWT	LIV	360	NO	HK	HK	T	W	C	C	9/5/10	9/5/10	I	NY	ERDA INCORPORATED
2010661355	PACB	PACHYTRITON	BREVIPES	TSITOU	NEWT	LIV	6	NO	HK	HK	T	C	C	C	9/5/10	9/5/10	I	NY	Transship Discounts Ltd.
2010661355	CYPY	CYNOPS	PYRRHOGASTER	JAPANESE FIREBELLY	NEWT	LIV	10	NO	HK	HK	T	C	C	C	9/5/10	9/5/10	I	NY	Transship Discounts Ltd.
2010662703	TRIH	TRITURUS	HONGKONGENSIS	ALPINE	NEWT	LIV	1,600	NO	HK	HK	T	C	C	C	9/8/10	9/8/10	I	TP	5-D TROPICAL INC.
2010664527	NOVI	NOTOPHTHALMUS	VIRIDESCENS	EASTERN	NEWT	LIV	2	NO	US	CA	T	W	C	C	9/10/10	9/8/10	I	BN	Science Kit, Inc - Boreal
2010664657	CYPY	CYNOPS	PYRRHOGASTER	JAPANESE FIREBELLY	NEWT	LIV	200	NO	CN	CN	T	C	C	C	9/10/10	9/11/10	I	AT	SUN PET LTD.
2010664710	TRIH	TRITURUS	HONGKONGENSIS	ALPINE	NEWT	LIV	200	NO	HK	HK	T	C	C	C	9/11/10	9/12/10	I	LA	AQUACO, INC.
2010664717	TRIT	TRITURUS	SPECIES		NEWT	LIV	290	NO	CN	HK	T	W	C	C	9/11/10	9/12/10	I	LA	ALL MARINE TRANSHIPPING INC
2010664717	CYOR	CYNOPS	ORIENTALIS	CHINESE DWARF	NEWT	LIV	300	NO	CN	HK	T	W	C	C	9/11/10	9/12/10	I	LA	ALL MARINE TRANSHIPPING INC
2010664792	CYOR	CYNOPS	ORIENTALIS	CHINESE DWARF	NEWT	LIV	100	NO	HK	HK	T	C	C	C	9/12/10	9/12/10	I	LA	DOLPHIN INTERNATIONAL
2010664942	CYPY	CYNOPS	PYRRHOGASTER	JAPANESE FIREBELLY	NEWT	LIV	60	NO	HK	HK	T	W	C	C	9/13/10	9/12/10	I	NY	ERDA INCORPORATED
2010665687	CYPY	CYNOPS	PYRRHOGASTER	JAPANESE FIREBELLY	NEWT	LIV	10	NO	HK	HK	T	C	C	C	9/14/10	9/12/10	I	NY	Transship Discounts Ltd.
2010665871	NOVI	NOTOPHTHALMUS	VIRIDESCENS	EASTERN	NEWT	LIV	5	NO	US	CA	T	W	C	C	9/13/10	9/13/10	I	BN	Science Kit, Inc - Boreal
2010668174	CYOR	CYNOPS	ORIENTALIS	CHINESE DWARF	NEWT	LIV	550	NO	HK	HK	T	C	C	C	9/19/10	9/19/10	I	LA	DOLPHIN INTERNATIONAL
2010668174	PACB	PACHYTRITON	BREVIPES	TSITOU	NEWT	LIV	50	NO	HK	HK	T	C	C	C	9/19/10	9/19/10	I	LA	DOLPHIN INTERNATIONAL
2010668177	TRIH	TRITURUS	HONGKONGENSIS	ALPINE	NEWT	LIV	200	NO	HK	HK	T	C	C	C	9/19/10	9/19/10	I	LA	AQUACO, INC.
2010668984	NOVI	NOTOPHTHALMUS	VIRIDESCENS	EASTERN	NEWT	LIV	4	NO	US	CA	T	W	C	C	9/21/10	9/20/10	I	BN	Science Kit, Inc - Boreal
2010669765	NEKA	NEURERGUS	KAISERI	KAISER'S SPOTTED	NEWT	LIV	90	NO	DE	DE	T	C	C	C	9/21/10	6/21/10	I	MI	REPTILE INDUSTRIES, INC.
2010670096	CYPY	CYNOPS	PYRRHOGASTER	JAPANESE FIREBELLY	NEWT	LIV	490	NO	HK	HK	T	C	C	C	9/22/10	9/19/10	I	NY	Transship Discounts Ltd.
2010670108	TRIH	TRITURUS	HONGKONGENSIS	ALPINE	NEWT	LIV	2,100	NO	HK	HK	T	C	C	C	9/22/10	9/22/10	I	TP	5-D TROPICAL INC.
2010671363	NOVI	NOTOPHTHALMUS	VIRIDESCENS	EASTERN	NEWT	LIV	3	NO	US	CA	T	W	C	C	9/23/10	9/22/10	I	BN	Science Kit, Inc - Boreal
2010672053	TRIH	TRITURUS	HONGKONGENSIS	ALPINE	NEWT	LIV	150	NO	HK	HK	T	C	C	C	9/26/10	9/26/10	I	LA	AQUACO, INC.
2010672071	CYOR	CYNOPS	ORIENTALIS	CHINESE DWARF	NEWT	LIV	1,075	NO	HK	HK	T	C	C	C	9/26/10	9/26/10	I	LA	DOLPHIN INTERNATIONAL
2010672085	TRIT	TRITURUS	SPECIES		NEWT	LIV	100	NO	VS	HK	T	W	C	C	9/26/10	9/26/10	I	LA	ALL MARINE TRANSHIPPING INC
2010672085	CNP?	CYNOPS	SPECIES	FIREBELLY	NEWT	LIV	1,480	NO	VS	HK	T	W	C	C	9/26/10	9/26/10	I	LA	ALL MARINE TRANSHIPPING INC
2010672104	CYPY	CYNOPS	PYRRHOGASTER	JAPANESE FIREBELLY	NEWT	LIV	210	NO	HK	HK	T	W	C	C	9/26/10	9/26/10	I	NY	ERDA INCORPORATED
2010672457	CYPY	CYNOPS	PYRRHOGASTER	JAPANESE FIREBELLY	NEWT	LIV	320	NO	HK	HK	T	C	C	C	9/27/10	9/26/10	I	NY	Transship Discounts Ltd.
2010673197	NOVI	NOTOPHTHALMUS	VIRIDESCENS	EASTERN	NEWT	LIV	2	NO	US	CA	T	W	C	C	9/27/10	9/27/10	I	BN	Science Kit, Inc - Boreal
2010674724	CYOR	CYNOPS	ORIENTALIS	CHINESE DWARF	NEWT	LIV	1,000	NO	CN	CN	T	C	C	C	9/30/10	9/30/10	I	LA	WORLDWIDE TRADING INC
2010674794	NOVI	NOTOPHTHALMUS	VIRIDESCENS	EASTERN	NEWT	LIV	8	NO	US	CA	T	W	C	C	9/29/10	9/29/10	I	BN	Science Kit, Inc - Boreal
2010675821	CYPY	CYNOPS	PYRRHOGASTER	JAPANESE FIREBELLY	NEWT	LIV	200	NO	CN	CN	T	C	C	C	10/2/10	10/2/10	I	AT	SUN PET LTD.
2010675882	CYOR	CYNOPS	ORIENTALIS	CHINESE DWARF	NEWT	LIV	375	NO	HK	HK	T	C	C	C	10/3/10	10/3/10	I	LA	DOLPHIN INTERNATIONAL
2010675882	PACB	PACHYTRITON	BREVIPES	TSITOU	NEWT	LIV	50	NO	HK	HK	T	C	C	C	10/3/10	10/3/10	I	LA	DOLPHIN INTERNATIONAL
2010675907	CNP?	CYNOPS	SPECIES	FIREBELLY	NEWT	LIV	740	NO	VS	HK	T	C	C	C	10/3/10	10/3/10	I	LA	ALL MARINE TRANSHIPPING INC
2010675907	TRIT	TRITURUS	SPECIES		NEWT	LIV	100	NO	VS	HK	T	W	C	C	10/3/10	10/3/10	I	LA	ALL MARINE TRANSHIPPING INC
2010676888	NOVI	NOTOPHTHALMUS	VIRIDESCENS	EASTERN	NEWT	LIV	2	NO	US	CA	T	W	C	C	10/4/10	10/4/10	I	BN	Science Kit, Inc - Boreal
2010677403	TRIH	TRITURUS	HONGKONGENSIS	ALPINE	NEWT	LIV	2,100	NO	HK	HK	T	C	C	C	10/6/10	10/3/10	I	TP	5-D TROPICAL INC.
2010678468	NOVI	NOTOPHTHALMUS	VIRIDESCENS	EASTERN	NEWT	LIV	3	NO	US	CA	T	W	C	C	10/6/10	10/6/10	I	BN	Science Kit, Inc - Boreal
2010679330	CYOR	CYNOPS	ORIENTALIS	CHINESE DWARF	NEWT	LIV	2,125	NO	HK	HK	T	C	C	C	10/10/10	10/10/10	I	LA	DOLPHIN INTERNATIONAL
2010679356	TRIT	TRITURUS	SPECIES		NEWT	LIV	200	NO	VS	HK	T	W	C	C	10/10/10	10/10/10	I	LA	ALL MARINE TRANSHIPPING INC
2010679979	CYPY	CYNOPS	PYRRHOGASTER	JAPANESE FIREBELLY	NEWT	LIV	105	NO	HK	HK	T	C	C	C	10/12/10	10/3/10	I	NY	Transship Discounts Ltd.
2010679979	PACB	PACHYTRITON	BREVIPES	TSITOU	NEWT	LIV	60	NO	HK	HK	T	C	C	C	10/12/10	10/3/10	I	NY	Transship Discounts Ltd.
2010680067	CYPY	CYNOPS	PYRRHOGASTER	JAPANESE FIREBELLY	NEWT	LIV	380	NO	HK	HK	T	W	C	C	10/12/10	10/10/10	I	NY	ERDA INCORPORATED
2010680201	CYPY	CYNOPS	PYRRHOGASTER	JAPANESE FIREBELLY	NEWT	LIV	400	NO	HK	HK	T	C	C	C	10/12/10	10/10/10	I	NY	Transship Discounts Ltd.
2010680201	PACB	PACHYTRITON	BREVIPES	TSITOU	NEWT	LIV	30	NO	HK	HK	T	C	C	C	10/12/10	10/10/10	I	NY	Transship Discounts Ltd.
2010681290	TRIH	TRITURUS	HONGKONGENSIS	ALPINE	NEWT	LIV	1,600	NO	HK	HK	T	C	C	C	10/13/10	10/13/10	I	TP	5-D TROPICAL INC.
2010682629	NOVI	NOTOPHTHALMUS	VIRIDESCENS	EASTERN	NEWT	LIV	21	NO	US	CA	T	W	C	C	10/15/10	10/13/10	I	BN	Science Kit, Inc - Boreal
2010682980	CYOR	CYNOPS	ORIENTALIS	CHINESE DWARF	NEWT	LIV	150	NO	HK	HK	T	C	C	C	10/17/10	10/17/10	I	LA	DOLPHIN INTERNATIONAL
2010682980	PACB	PACHYTRITON	BREVIPES	TSITOU	NEWT	LIV	100	NO	HK	HK	T	C	C	C	10/17/10	10/17/10	I	LA	DOLPHIN INTERNATIONAL
2010683000	TRIT	TRITURUS	SPECIES		NEWT	LIV	590	NO	VS	HK	T	W	C	C	10/17/10	10/17/10	I	LA	ALL MARINE TRANSHIPPING INC
2010683000	CYOR	CYNOPS	ORIENTALIS	CHINESE DWARF	NEWT	LIV	600	NO	VS	HK	T	W	C	C	10/17/10	10/17/10	I	LA	ALL MARINE TRANSHIPPING INC
2010683103	CYPY	CYNOPS	PYRRHOGASTER	JAPANESE FIREBELLY	NEWT	LIV	380	NO	HK	HK	T	W	C	C	10/18/10	10/17/10	I	NY	ERDA INCORPORATED
2010683141	CYPY	CYNOPS	PYRRHOGASTER	JAPANESE FIREBELLY	NEWT	LIV	85	NO	HK	HK	T	C	C	C	10/18/10	10/17/10	I	NY	Transship Discounts Ltd.
2010684349	NOVI	NOTOPHTHALMUS	VIRIDESCENS	EASTERN	NEWT	LIV	4	NO	US	CA	T	W	C	C	10/18/10	10/18/10	I	BN	Science Kit, Inc - Boreal

2010684506	TRIH	TRITURUS	HONGKONGENSIS	ALPINE	NEWT	LIV	500	NO	HK	HK	T	C	C	C	10/20/10	10/20/10	I	TP	5-D TROPICAL INC.
2010686259	NOVI	NOTOPHTHALMUS	VIRIDESCENS	EASTERN	NEWT	LIV	1	NO	US	CA	T	W	C	C	10/20/10	10/20/10	I	BN	Science Kit, Inc - Boreal
2010686483	CYOR	CYNOPS	ORIENTALIS	CHINESE DWARF	NEWT	LIV	125	NO	HK	HK	T	C	C	C	10/24/10	10/24/10	I	LA	DOLPHIN INTERNATIONAL
2010686483	PACB	PACHYTRITON	BREVIPES	TSITOU	NEWT	LIV	50	NO	HK	HK	T	C	C	C	10/24/10	10/24/10	I	LA	DOLPHIN INTERNATIONAL
2010686485	TRIH	TRITURUS	HONGKONGENSIS	ALPINE	NEWT	LIV	50	NO	HK	HK	T	C	C	C	10/24/10	10/24/10	I	LA	AQUACO, INC.
2010686489	TRIT	TRITURUS	SPECIES		NEWT	LIV	46	NO	VS	HK	T	W	C	C	10/24/10	10/24/10	I	LA	ALL MARINE TRANSHIPPING INC
2010686998	CYPY	CYNOPS	PYRRHOGASTER	JAPANESE FIREBELLY	NEWT	LIV	130	NO	HK	HK	T	C	C	C	10/25/10	10/24/10	I	NY	Transship Discounts Ltd.
2010689984	CNP?	CYNOPS	SPECIES	FIREBELLY	NEWT	LIV	200	NO	CN	CN	T	C	C	C	10/30/10	10/30/10	I	AT	SUN PET LTD.
2010690054	TRIH	TRITURUS	HONGKONGENSIS	ALPINE	NEWT	LIV	250	NO	HK	HK	T	C	C	C	10/30/10	10/31/10	I	LA	AQUACO, INC.
2010690114	CYOR	CYNOPS	ORIENTALIS	CHINESE DWARF	NEWT	LIV	500	NO	HK	HK	T	C	C	C	10/31/10	10/31/10	I	LA	DOLPHIN INTERNATIONAL
2010690114	PACB	PACHYTRITON	BREVIPES	TSITOU	NEWT	LIV	50	NO	HK	HK	T	C	C	C	10/31/10	10/31/10	I	LA	DOLPHIN INTERNATIONAL
2010690129	CYOR	CYNOPS	ORIENTALIS	CHINESE DWARF	NEWT	LIV	880	NO	VS	HK	T	W	C	C	10/31/10	10/31/10	I	LA	ALL MARINE TRANSHIPPING INC
2010690129	TRIH	TRITURUS	HONGKONGENSIS	ALPINE	NEWT	LIV	100	NO	VS	HK	T	W	C	C	10/31/10	10/31/10	I	LA	ALL MARINE TRANSHIPPING INC
2010691073	CYOR	CYNOPS	ORIENTALIS	CHINESE DWARF	NEWT	LIV	500	NO	CN	CN	T	W	C	C	11/2/10	11/2/10	I	LA	WORLDWIDE TRADING INC
2010691073	PLAB	PACHYTRITON	LABIATUS	UNTERSTEIN'S	NEWT	LIV	1,000	NO	CN	CN	T	W	C	C	11/2/10	11/2/10	I	LA	WORLDWIDE TRADING INC
2010691455	CYOR	CYNOPS	ORIENTALIS	CHINESE DWARF	NEWT	LIV	300	NO	HK	HK	T	C	C	C	11/2/10	11/2/10	I	CH	AQUATICS INC
2010691803	TRIH	TRITURUS	HONGKONGENSIS	ALPINE	NEWT	LIV	2,100	NO	HK	HK	T	C	C	C	11/3/10	11/3/10	I	TP	5-D TROPICAL INC.
2010692058	NOVI	NOTOPHTHALMUS	VIRIDESCENS	EASTERN	NEWT	LIV	8	NO	US	CA	T	W	C	C	11/3/10	11/3/10	I	BN	Science Kit, Inc - Boreal
2010692066	NOVI	NOTOPHTHALMUS	VIRIDESCENS	EASTERN	NEWT	LIV	7	NO	US	CA	T	W	C	C	10/25/10	10/25/10	I	BN	Science Kit, Inc - Boreal
2010693596	CYPY	CYNOPS	PYRRHOGASTER	JAPANESE FIREBELLY	NEWT	LIV	50	NO	SG	SG	T	C	C	C	11/7/10	11/7/10	I	LA	TROPICAL FISH CONSOLIDATORS
2010693610	CNP?	CYNOPS	SPECIES	FIREBELLY	NEWT	LIV	140	NO	VS	HK	T	W	C	C	11/7/10	11/7/10	I	LA	ALL MARINE TRANSHIPPING INC
2010693655	CYOR	CYNOPS	ORIENTALIS	CHINESE DWARF	NEWT	LIV	250	NO	HK	HK	T	C	C	C	11/7/10	11/7/10	I	LA	DOLPHIN INTERNATIONAL
2010693655	PACB	PACHYTRITON	BREVIPES	TSITOU	NEWT	LIV	100	NO	HK	HK	T	C	C	C	11/7/10	11/7/10	I	LA	DOLPHIN INTERNATIONAL
2010693990	CYPY	CYNOPS	PYRRHOGASTER	JAPANESE FIREBELLY	NEWT	LIV	360	NO	HK	HK	T	W	C	C	11/8/10	11/7/10	I	NY	ERDA INCORPORATED
2010694044	CYPY	CYNOPS	PYRRHOGASTER	JAPANESE FIREBELLY	NEWT	LIV	60	NO	HK	HK	T	W	C	C	11/8/10	11/7/10	I	NY	Transship Discounts Ltd.
2010694344	NOVI	NOTOPHTHALMUS	VIRIDESCENS	EASTERN	NEWT	LIV	3	NO	US	CA	T	W	C	C	11/8/10	11/8/10	I	BN	Science Kit, Inc - Boreal
2010696154	AMBM	AMBYSTOMA	MEXICANUM		AXOLOTL	LIV	340	NO	CZ	CZ	T	C	C	C	11/12/10	11/10/10	I	TP	JOSEPH TROPICAL
2010696367	SAL?	SALAMANDRA	SPECIES	FIRE	SALAMANDER	LIV	100	NO	UA	UA	T	W	C	C	11/12/10	11/12/10	I	LA	NAME REMOVED
2010696745	SASA	SALAMANDRA	SALAMANDRA	EUROPEAN FIRE	SALAMANDER	LIV	13	NO	GB	GB	P	C	C	C	11/12/10	11/10/10	I	MI	NAME REMOVED
2010697101	CNP?	CYNOPS	SPECIES	FIREBELLY	NEWT	LIV	200	NO	CN	CN	T	C	C	C	11/13/10	11/13/10	I	AT	SUN PET LTD.
2010697215	CYOR	CYNOPS	ORIENTALIS	CHINESE DWARF	NEWT	LIV	250	NO	HK	HK	T	C	C	C	11/14/10	11/14/10	I	LA	DOLPHIN INTERNATIONAL
2010697222	TRIH	TRITURUS	HONGKONGENSIS	ALPINE	NEWT	LIV	145	NO	VS	HK	T	W	C	C	11/14/10	11/14/10	I	LA	ALL MARINE TRANSHIPPING INC
2010697222	CNP?	CYNOPS	SPECIES	FIREBELLY	NEWT	LIV	600	NO	VS	HK	T	W	C	C	11/14/10	11/14/10	I	LA	ALL MARINE TRANSHIPPING INC
2010697833	NOVI	NOTOPHTHALMUS	VIRIDESCENS	EASTERN	NEWT	LIV	1	NO	US	CA	T	W	C	C	11/15/10	11/15/10	I	BN	Science Kit, Inc - Boreal
2010699385	SASA	SALAMANDRA	SALAMANDRA	EUROPEAN FIRE	SALAMANDER	LIV	4	NO	DE	DE	T	C	C	C	9/22/10	9/22/10	I	SF	Exemptions 6 and 7(C)
2010699747	NOVI	NOTOPHTHALMUS	VIRIDESCENS	EASTERN	NEWT	LIV	4	NO	US	CA	T	W	C	C	11/17/10	11/17/10	I	BN	Science Kit, Inc - Boreal
2010700826	CYPY	CYNOPS	PYRRHOGASTER	JAPANESE FIREBELLY	NEWT	LIV	290	NO	HK	HK	T	W	C	C	11/18/10	11/14/10	I	NY	ERDA INCORPORATED
2010700951	PACB	PACHYTRITON	BREVIPES	TSITOU	NEWT	LIV	390	NO	HK	HK	T	W	C	C	11/18/10	11/14/10	I	NY	Transship Discounts Ltd.
2010701993	TRIH	TRITURUS	HONGKONGENSIS	ALPINE	NEWT	LIV	25	NO	HK	HK	T	C	C	C	11/21/10	11/21/10	I	LA	AQUACO, INC.
2010701996	CYOR	CYNOPS	ORIENTALIS	CHINESE DWARF	NEWT	LIV	625	NO	HK	HK	T	C	C	C	11/21/10	11/21/10	I	LA	DOLPHIN INTERNATIONAL
2010702075	CNP?	CYNOPS	SPECIES	FIREBELLY	NEWT	LIV	140	NO	VS	HK	T	W	C	C	11/21/10	11/21/10	I	LA	ALL MARINE TRANSHIPPING INC
2010702383	CYPY	CYNOPS	PYRRHOGASTER	JAPANESE FIREBELLY	NEWT	LIV	210	NO	HK	HK	T	W	C	C	11/22/10	11/21/10	I	NY	ERDA INCORPORATED
2010702439	CYPY	CYNOPS	PYRRHOGASTER	JAPANESE FIREBELLY	NEWT	LIV	105	NO	HK	HK	T	W	C	C	11/22/10	11/21/10	I	NY	Transship Discounts Ltd.
2010702977	TRIH	TRITURUS	HONGKONGENSIS	ALPINE	NEWT	LIV	2,100	NO	HK	HK	T	C	C	C	11/22/10	11/22/10	I	TP	5-D TROPICAL INC.
2010705192	PACB	PACHYTRITON	BREVIPES	TSITOU	NEWT	LIV	50	NO	HK	HK	T	C	C	C	11/28/10	11/28/10	I	LA	DOLPHIN INTERNATIONAL
2010705206	TRIT	TRITURUS	SPECIES		NEWT	LIV	740	NO	VS	HK	T	W	C	C	11/28/10	11/28/10	I	LA	ALL MARINE TRANSHIPPING INC
2010706858	PACB	PACHYTRITON	BREVIPES	TSITOU	NEWT	LIV	90	NO	HK	HK	T	W	C	C	11/30/10	11/28/10	I	NY	Transship Discounts Ltd.
2010708214	NOVI	NOTOPHTHALMUS	VIRIDESCENS	EASTERN	NEWT	LIV	2	NO	US	CA	T	W	C	C	12/1/10	12/1/10	I	BN	Science Kit, Inc - Boreal
2010708244	SAL?	SALAMANDRA	SPECIES	FIRE	SALAMANDER	LIV	100	NO	UA	UA	T	W	C	C	12/2/10	12/2/10	I	LA	NAME REMOVED
2010709304	TRIH	TRITURUS	HONGKONGENSIS	ALPINE	NEWT	LIV	350	NO	HK	HK	T	C	C	C	12/4/10	12/5/10	I	LA	AQUACO, INC.
2010709355	CYOR	CYNOPS	ORIENTALIS	CHINESE DWARF	NEWT	LIV	500	NO	HK	HK	T	C	C	C	12/5/10	12/5/10	I	LA	DOLPHIN INTERNATIONAL
2010709355	PACB	PACHYTRITON	BREVIPES	TSITOU	NEWT	LIV	50	NO	HK	HK	T	C	C	C	12/5/10	12/5/10	I	LA	DOLPHIN INTERNATIONAL
2010709371	CNP?	CYNOPS	SPECIES	FIREBELLY	NEWT	LIV	140	NO	VS	HK	T	W	C	C	12/5/10	12/5/10	I	LA	ALL MARINE TRANSHIPPING INC
2010709537	CYPY	CYNOPS	PYRRHOGASTER	JAPANESE FIREBELLY	NEWT	LIV	60	NO	HK	HK	T	W	C	C	12/6/10	11/28/10	I	NY	ERDA INCORPORATED
2010709552	CYPY	CYNOPS	PYRRHOGASTER	JAPANESE FIREBELLY	NEWT	LIV	300	NO	HK	HK	T	W	C	C	12/6/10	12/5/10	I	NY	ERDA INCORPORATED
2010709746	CYPY	CYNOPS	PYRRHOGASTER	JAPANESE FIREBELLY	NEWT	LIV	20	NO	HK	HK	T	C	C	C	12/6/10	12/5/10	I	NY	Transship Discounts Ltd.
2010710960	TRIH	TRITURUS	HONGKONGENSIS	ALPINE	NEWT	LIV	2,100	NO	HK	HK	T	C	C	C	12/7/10	12/8/10	I	TP	5-D TROPICAL INC.
2010713708	CNP?	CYNOPS	SPECIES	FIREBELLY	NEWT	LIV	200	NO	CN	CN	T	C	C	C	12/11/10	12/11/10	I	AT	SUN PET LTD.
2010713816	CYOR	CYNOPS	ORIENTALIS	CHINESE DWARF	NEWT	LIV	375	NO	HK	HK	T	C	C	C	12/12/10	12/12/10	I	LA	DOLPHIN INTERNATIONAL
2010713843	TRIH	TRITURUS	HONGKONGENSIS	ALPINE	NEWT	LIV	450	NO	HK	HK	T	C	C	C	12/12/10	12/12/10	I	LA	AQUACO, INC.

2010713845	CYOR	CYNOPS	ORIENTALIS	CHINESE DWARF	NEWT	LIV	1,440	NO	VS	HK	T	W	C	C	12/12/10	12/12/10	I	LA	ALL MARINE TRANSHIPPING INC
2010713986	CYPY	CYNOPS	PYRRHOGASTER	JAPANESE FIREBELLY	NEWT	LIV	200	NO	HK	HK	T	W	C	C	12/13/10	12/12/10	I	NY	ERDA INCORPORATED
2010714087	CYPY	CYNOPS	PYRRHOGASTER	JAPANESE FIREBELLY	NEWT	LIV	210	NO	HK	HK	T	C	C	C	12/13/10	12/12/10	I	NY	Transship Discounts Ltd.
2010714087	PACB	PACHYTRITON	BREVIPES	TSITOU	NEWT	LIV	60	NO	HK	HK	T	C	C	C	12/13/10	12/12/10	I	NY	Transship Discounts Ltd.
2010716653	NOVI	NOTOPHTHALMUS	VIRIDESCENS	EASTERN	NEWT	LIV	1	NO	US	CA	T	W	C	C	12/13/10	12/13/10	I	BN	Science Kit, Inc - Boreal
2010718192	TRIH	TRITURUS	HONGKONGENSIS	ALPINE	NEWT	LIV	200	NO	HK	HK	T	C	C	C	12/18/10	12/19/10	I	LA	AQUACO, INC.
2010718239	CYOR	CYNOPS	ORIENTALIS	CHINESE DWARF	NEWT	LIV	225	NO	HK	HK	T	C	C	C	12/19/10	12/19/10	I	LA	DOLPHIN INTERNATIONAL
2010718239	PACB	PACHYTRITON	BREVIPES	TSITOU	NEWT	LIV	50	NO	HK	HK	T	C	C	C	12/19/10	12/19/10	I	LA	DOLPHIN INTERNATIONAL
2010718737	NOVI	NOTOPHTHALMUS	VIRIDESCENS	EASTERN	NEWT	LIV	1	NO	US	CA	T	W	C	C	12/20/10	12/20/10	I	BN	Science Kit, Inc - Boreal
2010719337	TRIH	TRITURUS	HONGKONGENSIS	ALPINE	NEWT	LIV	1,600	NO	HK	HK	T	C	C	C	12/21/10	12/21/10	I	TP	5-D TROPICAL INC.
2010721408	CYOR	CYNOPS	ORIENTALIS	CHINESE DWARF	NEWT	LIV	125	NO	HK	HK	T	C	C	C	12/26/10	12/26/10	I	LA	DOLPHIN INTERNATIONAL
2010721408	PACB	PACHYTRITON	BREVIPES	TSITOU	NEWT	LIV	50	NO	HK	HK	T	C	C	C	12/26/10	12/26/10	I	LA	DOLPHIN INTERNATIONAL
2010721440	PLAB	PACHYTRITON	LABIATUS	UNTERSTEIN'S	NEWT	LIV	145	NO	CN	HK	T	W	C	C	12/26/10	12/26/10	I	LA	ALL MARINE TRANSHIPPING INC
2010721440	CYOR	CYNOPS	ORIENTALIS	CHINESE DWARF	NEWT	LIV	300	NO	CN	HK	T	W	C	C	12/26/10	12/26/10	I	LA	ALL MARINE TRANSHIPPING INC
2010721440	TRIT	TRITURUS	SPECIES		NEWT	LIV	100	NO	CN	HK	T	W	C	C	12/26/10	12/26/10	I	LA	ALL MARINE TRANSHIPPING INC
2010722007	TRIH	TRITURUS	HONGKONGENSIS	ALPINE	NEWT	LIV	2,100	NO	HK	HK	T	C	C	C	12/28/10	12/28/10	I	TP	5-D TROPICAL INC.
2010722137	CYPY	CYNOPS	PYRRHOGASTER	JAPANESE FIREBELLY	NEWT	LIV	80	NO	HK	HK	T	W	C	C	12/28/10	12/26/10	I	NY	ERDA INCORPORATED
2011723831	CYOR	CYNOPS	ORIENTALIS	CHINESE DWARF	NEWT	LIV	650	NO	HK	HK	T	C	C	C	1/2/11	1/2/11	I	LA	DOLPHIN INTERNATIONAL
2011723831	PACB	PACHYTRITON	BREVIPES	TSITOU	NEWT	LIV	100	NO	HK	HK	T	C	C	C	1/2/11	1/2/11	I	LA	DOLPHIN INTERNATIONAL
2011723854	CNP?	CYNOPS	SPECIES	FIREBELLY	NEWT	LIV	1,620	NO	VS	HK	T	W	C	C	1/2/11	1/2/11	I	LA	ALL MARINE TRANSHIPPING INC
2011724995	CYPY	CYNOPS	PYRRHOGASTER	JAPANESE FIREBELLY	NEWT	LIV	165	NO	HK	HK	T	C	C	C	1/4/11	1/2/11	I	NY	Transship Discounts Ltd.
2011725960	CYOR	CYNOPS	ORIENTALIS	CHINESE DWARF	NEWT	LIV	6,100	NO	CN	CN	T	W	C	C	1/6/11	1/6/11	I	LA	WORLDWIDE TRADING INC
2011725960	PLAB	PACHYTRITON	LABIATUS	UNTERSTEIN'S	NEWT	LIV	1,000	NO	CN	CN	T	W	C	C	1/6/11	1/6/11	I	LA	WORLDWIDE TRADING INC
2011726143	NOVI	NOTOPHTHALMUS	VIRIDESCENS	EASTERN	NEWT	LIV	28	NO	US	CA	T	W	C	C	1/5/11	1/5/11	I	BN	Science Kit, Inc - Boreal
2011727194	CNP?	CYNOPS	SPECIES	FIREBELLY	NEWT	LIV	200	NO	CN	CN	T	C	C	C	1/9/11	1/9/11	I	AT	SUN PET LTD.
2011727795	CNP?	CYNOPS	SPECIES	FIREBELLY	NEWT	LIV	740	NO	VS	HK	T	W	C	C	1/10/11	1/9/11	I	LA	ALL MARINE TRANSHIPPING INC
2011728463	TRIH	TRITURUS	HONGKONGENSIS	ALPINE	NEWT	LIV	50	NO	HK	HK	T	C	C	C	1/11/11	1/9/11	I	LA	AQUACO, INC.
2011728638	TRIH	TRITURUS	HONGKONGENSIS	ALPINE	NEWT	LIV	1,600	NO	HK	HK	T	C	C	C	1/12/11	1/12/11	I	TP	5-D TROPICAL INC.
2011729959	CYPY	CYNOPS	PYRRHOGASTER	JAPANESE FIREBELLY	NEWT	LIV	80	NO	HK	HK	T	W	C	C	1/13/11	1/2/11	I	NY	ERDA INCORPORATED
2011731057	TRIH	TRITURUS	HONGKONGENSIS	ALPINE	NEWT	LIV	50	NO	HK	HK	T	C	C	C	1/15/11	1/16/11	I	LA	AQUACO, INC.
2011731098	PLAB	PACHYTRITON	LABIATUS	UNTERSTEIN'S	NEWT	LIV	400	NO	VS	HK	T	W	C	C	1/16/11	1/16/11	I	LA	AQUA-NAUTIC SPECIALIST
2011731100	CYOR	CYNOPS	ORIENTALIS	CHINESE DWARF	NEWT	LIV	325	NO	HK	HK	T	C	C	C	1/16/11	1/16/11	I	LA	DOLPHIN INTERNATIONAL
2011731100	PACB	PACHYTRITON	BREVIPES	TSITOU	NEWT	LIV	50	NO	HK	HK	T	C	C	C	1/16/11	1/16/11	I	LA	DOLPHIN INTERNATIONAL
2011731841	TRIH	TRITURUS	HONGKONGENSIS	ALPINE	NEWT	LIV	500	NO	HK	HK	T	C	C	C	1/18/11	1/18/11	I	TP	5-D TROPICAL INC.
2011731907	CYPY	CYNOPS	PYRRHOGASTER	JAPANESE FIREBELLY	NEWT	LIV	150	NO	HK	HK	T	W	C	C	1/18/11	1/9/11	I	NY	ERDA INCORPORATED
2011731927	PACB	PACHYTRITON	BREVIPES	TSITOU	NEWT	LIV	30	NO	HK	HK	T	C	C	C	1/18/11	1/16/11	I	NY	Transship Discounts Ltd.
2011732009	CYPY	CYNOPS	PYRRHOGASTER	JAPANESE FIREBELLY	NEWT	LIV	140	NO	HK	HK	T	W	C	C	1/18/11	1/16/11	I	NY	ERDA INCORPORATED
2011734341	NOVI	NOTOPHTHALMUS	VIRIDESCENS	EASTERN	NEWT	LIV	2	NO	US	CA	T	W	C	C	1/21/11	1/19/11	I	BN	Science Kit, Inc - Boreal
2011734626	CYOR	CYNOPS	ORIENTALIS	CHINESE DWARF	NEWT	LIV	500	NO	HK	HK	T	C	C	C	1/23/11	1/23/11	I	LA	DOLPHIN INTERNATIONAL
2011734637	TRIH	TRITURUS	HONGKONGENSIS	ALPINE	NEWT	LIV	500	NO	HK	HK	T	C	C	C	1/23/11	1/23/11	I	LA	AQUACO, INC.
2011734673	PLAB	PACHYTRITON	LABIATUS	UNTERSTEIN'S	NEWT	LIV	700	NO	VS	HK	T	W	C	C	1/23/11	1/23/11	I	LA	AQUA-NAUTIC SPECIALIST
2011736005	NOVI	NOTOPHTHALMUS	VIRIDESCENS	EASTERN	NEWT	LIV	1	NO	US	CA	T	W	C	C	1/24/11	1/24/11	I	BN	Science Kit, Inc - Boreal
2011736504	TRIH	TRITURUS	HONGKONGENSIS	ALPINE	NEWT	LIV	100	NO	HK	HK	T	C	C	C	1/26/11	1/26/11	I	TP	5-D TROPICAL, INC.
2011736803	NOVI	NOTOPHTHALMUS	VIRIDESCENS	EASTERN	NEWT	LIV	4	NO	US	CA	T	W	C	C	1/26/11	1/26/11	I	BN	Science Kit, Inc - Boreal
2011738441	PACB	PACHYTRITON	BREVIPES	TSITOU	NEWT	LIV	50	NO	HK	HK	T	C	C	C	1/30/11	1/30/11	I	LA	DOLPHIN INTERNATIONAL
2011738441	CYOR	CYNOPS	ORIENTALIS	CHINESE DWARF	NEWT	LIV	200	NO	HK	HK	T	C	C	C	1/30/11	1/30/11	I	LA	DOLPHIN INTERNATIONAL
2011738442	PACB	PACHYTRITON	BREVIPES	TSITOU	NEWT	LIV	100	NO	HK	HK	T	C	C	C	1/30/11	1/30/11	I	LA	DOLPHIN INTERNATIONAL
2011739458	CYPY	CYNOPS	PYRRHOGASTER	JAPANESE FIREBELLY	NEWT	LIV	80	NO	HK	HK	T	W	C	C	2/1/11	1/23/11	I	NY	ERDA INCORPORATED
2011739534	CYPY	CYNOPS	PYRRHOGASTER	JAPANESE FIREBELLY	NEWT	LIV	105	NO	HK	HK	T	C	C	C	2/1/11	1/23/11	I	NY	Transship Discounts Ltd.
2011739570	NOVI	NOTOPHTHALMUS	VIRIDESCENS	EASTERN	NEWT	LIV	3	NO	US	CA	T	W	C	C	2/1/11	1/31/11	I	BN	Science Kit, Inc - Boreal
2011739643	CYPY	CYNOPS	PYRRHOGASTER	JAPANESE FIREBELLY	NEWT	LIV	25	NO	HK	HK	T	C	C	C	2/1/11	1/30/11	I	NY	Transship Discounts Ltd.
2011739946	TRIH	TRITURUS	HONGKONGENSIS	ALPINE	NEWT	LIV	500	NO	HK	HK	T	C	C	C	2/2/11	2/3/11	I	TP	5-D TROPICAL INC.
2011740890	NOVI	NOTOPHTHALMUS	VIRIDESCENS	EASTERN	NEWT	LIV	1	NO	US	CA	T	W	C	C	2/2/11	2/2/11	I	BN	Science Kit, Inc - Boreal
2011741652	TRIH	TRITURUS	HONGKONGENSIS	ALPINE	NEWT	LIV	50	NO	HK	HK	T	C	C	C	2/6/11	2/6/11	I	LA	AQUACO, INC.
2011741669	PACB	PACHYTRITON	BREVIPES	TSITOU	NEWT	LIV	50	NO	HK	HK	T	C	C	C	2/6/11	2/6/11	I	LA	DOLPHIN INTERNATIONAL
2011741669	CYOR	CYNOPS	ORIENTALIS	CHINESE DWARF	NEWT	LIV	575	NO	HK	HK	T	C	C	C	2/6/11	2/6/11	I	LA	DOLPHIN INTERNATIONAL
2011741705	TRIH	TRITURUS	HONGKONGENSIS	ALPINE	NEWT	LIV	75	NO	SG	SG	T	C	C	C	2/6/11	2/6/11	I	LA	PET-SIAM INTERNATIONAL
2011744031	NOVI	NOTOPHTHALMUS	VIRIDESCENS	EASTERN	NEWT	LIV	5	NO	US	CA	T	W	C	C	2/9/11	2/7/11	I	BN	Science Kit, Inc - Boreal
2011745583	CYOR	CYNOPS	ORIENTALIS	CHINESE DWARF	NEWT	LIV	925	NO	HK	HK	T	C	C	C	2/13/11	2/13/11	I	LA	DOLPHIN INTERNATIONAL
2011745592	TRIH	TRITURUS	HONGKONGENSIS	ALPINE	NEWT	LIV	100	NO	HK	HK	T	C	C	C	2/13/11	2/13/11	I	LA	AQUACO, INC.

2011745604	CYPY	CYNOPS	PYRRHOGASTER	JAPANESE FIREBELLY	NEWT	LIV	100	NO	SG	SG	T	C	C	C	2/13/11	2/13/11	I	LA	TROPICAL FISH CONSOLIDATORS
2011746829	CYPY	CYNOPS	PYRRHOGASTER	JAPANESE FIREBELLY	NEWT	LIV	300	NO	HK	HK	T	W	C	C	2/15/11	2/13/11	I	NY	ERDA INCORPORATED
2011746843	CYPY	CYNOPS	PYRRHOGASTER	JAPANESE FIREBELLY	NEWT	LIV	60	NO	HK	HK	T	C	C	C	2/15/11	2/13/11	I	NY	Transship Discounts Ltd.
2011747164	NOVI	NOTOPHTHALMUS	VIRIDESCENS	EASTERN	NEWT	LIV	5	NO	US	CA	T	W	C	C	2/16/11	2/14/11	I	BN	Science Kit, Inc - Boreal
2011748748	CYOR	CYNOPS	ORIENTALIS	CHINESE DWARF	NEWT	LIV	600	NO	HK	HK	T	C	C	C	2/20/11	2/20/11	I	LA	DOLPHIN INTERNATIONAL
2011748763	TRIH	TRITURUS	HONGKONGENSIS	ALPINE	NEWT	LIV	100	NO	VS	HK	T	W	C	C	2/20/11	2/20/11	I	LA	AQUA-NAUTIC SPECIALIST
2011748763	PLAB	PACHYTRITON	LABIATUS	UNTERSTEIN'S	NEWT	LIV	45	NO	VS	HK	T	W	C	C	2/20/11	2/20/11	I	LA	AQUA-NAUTIC SPECIALIST
2011749046	CYPY	CYNOPS	PYRRHOGASTER	JAPANESE FIREBELLY	NEWT	LIV	400	NO	HK	HK	T	C	C	C	2/22/11	2/20/11	I	NY	Transship Discounts Ltd.
2011749216	CYOR	CYNOPS	ORIENTALIS	CHINESE DWARF	NEWT	LIV	1,700	NO	HK	HK	T	C	C	C	2/22/11	2/23/11	I	TP	5-D TROPICAL, INC.
2011749479	CYPY	CYNOPS	PYRRHOGASTER	JAPANESE FIREBELLY	NEWT	LIV	120	NO	HK	HK	T	W	C	C	2/22/11	2/20/11	I	NY	ERDA INCORPORATED
2011749725	CYOR	CYNOPS	ORIENTALIS	CHINESE DWARF	NEWT	LIV	492	NO	HK	HK	T	C	C	C	2/23/11	2/23/11	I	CH	AQUATICS INC
2011750172	NOVI	NOTOPHTHALMUS	VIRIDESCENS	EASTERN	NEWT	LIV	11	NO	US	CA	T	W	C	C	2/23/11	2/23/11	I	BN	Science Kit, Inc - Boreal
2011750549	AMBM	AMBYSTOMA	MEXICANUM		AXOLOTL	LIV	500	NO	CZ	CZ	T	C	C	C	2/24/11	2/23/11	I	TP	JOSEPH TROPICAL
2011751617	TRIH	TRITURUS	HONGKONGENSIS	ALPINE	NEWT	LIV	50	NO	HK	HK	T	C	C	C	2/27/11	2/27/11	I	LA	AQUACO, INC.
2011751640	TRIH	TRITURUS	HONGKONGENSIS	ALPINE	NEWT	LIV	45	NO	VS	HK	T	W	C	C	2/27/11	2/27/11	I	LA	AQUA-NAUTIC SPECIALIST
2011751640	PLAB	PACHYTRITON	LABIATUS	UNTERSTEIN'S	NEWT	LIV	45	NO	VS	HK	T	W	C	C	2/27/11	2/27/11	I	LA	AQUA-NAUTIC SPECIALIST
2011751659	CYOR	CYNOPS	ORIENTALIS	CHINESE DWARF	NEWT	LIV	350	NO	HK	HK	T	C	C	C	2/27/11	2/27/11	I	LA	DOLPHIN INTERNATIONAL
2011752132	CYPY	CYNOPS	PYRRHOGASTER	JAPANESE FIREBELLY	NEWT	LIV	30	NO	HK	HK	T	C	C	C	2/28/11	2/27/11	I	NY	Transship Discounts Ltd.
2011752506	CYPY	CYNOPS	PYRRHOGASTER	JAPANESE FIREBELLY	NEWT	LIV	60	NO	HK	HK	T	W	C	C	3/1/11	2/27/11	I	NY	ERDA INCORPORATED
2011752974	NOVI	NOTOPHTHALMUS	VIRIDESCENS	EASTERN	NEWT	LIV	1	NO	US	CA	T	W	C	C	3/1/11	2/28/11	I	BN	Science Kit, Inc - Boreal
2011753000	CYOR	CYNOPS	ORIENTALIS	CHINESE DWARF	NEWT	LIV	1,200	NO	HK	HK	T	C	C	C	3/1/11	3/2/11	I	TP	5-D TROPICAL, INC.
2011753001	CYOR	CYNOPS	ORIENTALIS	CHINESE DWARF	NEWT	LIV	500	NO	HK	HK	T	C	C	C	3/1/11	3/2/11	I	TP	5-D TROPICAL INC.
2011754924	SAL?	SALAMANDRA	SPECIES	FIRE	SALAMANDER	LIV	5	NO	DE	DE	T	C	C	C	3/4/11	2/24/11	I	SF	NAME REMOVED
2011754924	AADR	AMBYSTOMA	ANDERSONI	ANDERSON'S	SALAMANDER	LIV	4	NO	DE	DE	T	C	R	S	2/24/11	2/24/11	I	SF	NAME REMOVED
2011754924	PLAO	PARAMESOTRITON	LAOENSIS	WARTY	NEWT	LIV	15	NO	DE	DE	T	C	C	C	3/4/11	2/24/11	I	SF	NAME REMOVED
2011754924	TCRI	TRITURUS	CRISTATUS	NORTHERN CRESTED	NEWT	LIV	2	NO	DE	DE	T	C	C	C	3/4/11	2/24/11	I	SF	NAME REMOVED
2011754924	AMBM	AMBYSTOMA	MEXICANUM		AXOLOTL	LIV	15	NO	DE	DE	T	C	R	S	2/24/11	2/24/11	I	SF	NAME REMOVED
2011754924	AMBY	AMBYSTOMA	SPECIES	MOLE	SALAMANDER	LIV	5	NO	DE	DE	T	C	R	S	2/24/11	2/24/11	I	SF	NAME REMOVED
2011755546	CYOR	CYNOPS	ORIENTALIS	CHINESE DWARF	NEWT	LIV	1,000	NO	HK	HK	T	C	C	C	3/6/11	3/6/11	I	LA	DOLPHIN INTERNATIONAL
2011755546	PACB	PACHYTRITON	BREVIPES	TSITOU	NEWT	LIV	100	NO	HK	HK	T	C	C	C	3/6/11	3/6/11	I	LA	DOLPHIN INTERNATIONAL
2011755550	TRIH	TRITURUS	HONGKONGENSIS	ALPINE	NEWT	LIV	75	NO	HK	HK	T	C	C	C	3/6/11	3/6/11	I	LA	AQUACO, INC.
2011755554	CNP?	CYNOPS	SPECIES	FIREBELLY	NEWT	LIV	1,600	NO	VS	HK	T	W	C	C	3/6/11	3/6/11	I	LA	AQUA-NAUTIC SPECIALIST
2011755554	TRIT	TRITURUS	SPECIES		NEWT	LIV	100	NO	VS	HK	T	W	C	C	3/6/11	3/6/11	I	LA	AQUA-NAUTIC SPECIALIST
2011756061	NOVI	NOTOPHTHALMUS	VIRIDESCENS	EASTERN	NEWT	LIV	3	NO	US	CA	T	W	C	C	3/7/11	3/7/11	I	BN	Science Kit, Inc - Boreal
2011757252	NOVI	NOTOPHTHALMUS	VIRIDESCENS	EASTERN	NEWT	LIV	1	NO	US	CA	T	W	C	C	3/9/11	3/9/11	I	BN	Science Kit, Inc - Boreal
2011758020	CYPY	CYNOPS	PYRRHOGASTER	JAPANESE FIREBELLY	NEWT	LIV	85	NO	HK	HK	T	C	C	C	3/10/11	3/6/11	I	NY	Transship Discounts Ltd.
2011758659	CYPY	CYNOPS	PYRRHOGASTER	JAPANESE FIREBELLY	NEWT	LIV	90	NO	HK	HK	T	W	C	C	3/11/11	3/6/11	I	NY	ERDA INCORPORATED
2011758972	CYPY	CYNOPS	PYRRHOGASTER	JAPANESE FIREBELLY	NEWT	LIV	100	NO	SG	SG	T	C	C	C	3/12/11	3/13/11	I	LA	TROPICAL FISH CONSOLIDATORS
2011759028	TRIH	TRITURUS	HONGKONGENSIS	ALPINE	NEWT	LIV	50	NO	HK	HK	T	C	C	C	3/13/11	3/13/11	I	LA	AQUACO, INC.
2011759040	CYOR	CYNOPS	ORIENTALIS	CHINESE DWARF	NEWT	LIV	625	NO	HK	HK	T	C	C	C	3/13/11	3/13/11	I	LA	DOLPHIN INTERNATIONAL
2011759053	TRIT	TRITURUS	SPECIES		NEWT	LIV	100	NO	VS	HK	T	W	C	C	3/13/11	3/13/11	I	LA	AQUA-NAUTIC SPECIALIST
2011759053	CNP?	CYNOPS	SPECIES	FIREBELLY	NEWT	LIV	600	NO	VS	HK	T	W	C	C	3/13/11	3/13/11	I	LA	AQUA-NAUTIC SPECIALIST
2011759222	CYPY	CYNOPS	PYRRHOGASTER	JAPANESE FIREBELLY	NEWT	LIV	240	NO	HK	HK	T	C	C	C	3/14/11	3/13/11	I	NY	Transship Discounts Ltd.
2011759459	CYOR	CYNOPS	ORIENTALIS	CHINESE DWARF	NEWT	LIV	2,000	NO	HK	HK	T	C	C	C	3/14/11	3/16/11	I	TP	5-D TROPICAL, INC.
2011760354	TRIH	TRITURUS	HONGKONGENSIS	ALPINE	NEWT	LIV	1,600	NO	HK	HK	T	C	C	C	3/16/11	3/16/11	I	TP	5-D TROPICAL INC.
2011760354	CYOR	CYNOPS	ORIENTALIS	CHINESE DWARF	NEWT	LIV	500	NO	HK	HK	T	C	C	C	3/16/11	3/16/11	I	TP	5-D TROPICAL INC.
2011761418	NOVI	NOTOPHTHALMUS	VIRIDESCENS	EASTERN	NEWT	LIV	2	NO	US	CA	T	W	C	C	3/17/11	3/16/11	I	BN	Science Kit, Inc - Boreal
2011762194	CNP?	CYNOPS	SPECIES	FIREBELLY	NEWT	LIV	200	NO	CN	CN	T	C	C	C	3/19/11	3/19/11	I	AT	SUN PET LTD.
2011762302	CYOR	CYNOPS	ORIENTALIS	CHINESE DWARF	NEWT	LIV	750	NO	HK	HK	T	C	C	C	3/20/11	3/20/11	I	LA	DOLPHIN INTERNATIONAL
2011762313	CNP?	CYNOPS	SPECIES	FIREBELLY	NEWT	LIV	440	NO	VS	HK	T	W	C	C	3/20/11	3/20/11	I	LA	AQUA-NAUTIC SPECIALIST
2011762315	TRIH	TRITURUS	HONGKONGENSIS	ALPINE	NEWT	LIV	165	NO	HK	HK	T	C	C	C	3/20/11	3/20/11	I	LA	AQUACO, INC.
2011762628	CYPY	CYNOPS	PYRRHOGASTER	JAPANESE FIREBELLY	NEWT	LIV	135	NO	HK	HK	T	C	C	C	3/21/11	3/20/11	I	NY	Transship Discounts Ltd.
2011762720	CYPY	CYNOPS	PYRRHOGASTER	JAPANESE FIREBELLY	NEWT	LIV	410	NO	HK	HK	T	W	C	C	3/21/11	3/20/11	I	NY	ERDA INCORPORATED
2011762735	NOVI	NOTOPHTHALMUS	VIRIDESCENS	EASTERN	NEWT	LIV	1	NO	US	CA	T	W	C	C	3/21/11	3/21/11	I	BN	Science Kit, Inc - Boreal
2011763120	CYOR	CYNOPS	ORIENTALIS	CHINESE DWARF	NEWT	LIV	3,500	NO	CN	CN	T	W	C	C	3/22/11	3/22/11	I	LA	Z IMPORTS INC.
2011764958	CYOR	CYNOPS	ORIENTALIS	CHINESE DWARF	NEWT	LIV	4,200	NO	CN	CN	T	W	C	C	3/25/11	3/25/11	I	LA	WORLDWIDE TRADING INC
2011764958	PLAB	PACHYTRITON	LABIATUS	UNTERSTEIN'S	NEWT	LIV	1,200	NO	CN	CN	T	W	C	C	3/25/11	3/25/11	I	LA	WORLDWIDE TRADING INC
2011765494	CYOR	CYNOPS	ORIENTALIS	CHINESE DWARF	NEWT	LIV	275	NO	HK	HK	T	C	C	C	3/27/11	3/27/11	I	LA	DOLPHIN INTERNATIONAL
2011765494	PACB	PACHYTRITON	BREVIPES	TSITOU	NEWT	LIV	250	NO	HK	HK	T	C	C	C	3/27/11	3/27/11	I	LA	DOLPHIN INTERNATIONAL
2011765528	PLAB	PACHYTRITON	LABIATUS	UNTERSTEIN'S	NEWT	LIV	200	NO	VS	HK	T	W	C	C	3/27/11	3/27/11	I	LA	AQUA-NAUTIC SPECIALIST

2011765528	CNP?	CYNOPS	SPECIES	FIREBELLY	NEWT	LIV	300	NO	VS	HK	T	W	C	C	3/27/11	3/27/11	I	LA	AQUA-NAUTIC SPECIALIST
2011765985	NOVI	NOTOPHTHALMUS	VIRIDESCENS	EASTERN	NEWT	LIV	2	NO	US	CA	T	W	C	C	3/28/11	3/28/11	I	BN	Science Kit, Inc - Boreal
2011766659	CYPY	CYNOPS	PYRRHOGASTER	JAPANESE FIREBELLY	NEWT	LIV	80	NO	HK	HK	T	W	C	C	3/29/11	3/27/11	I	NY	ERDA INCORPORATED
2011766725	CYOR	CYNOPS	ORIENTALIS	CHINESE DWARF	NEWT	LIV	1,200	NO	HK	HK	T	C	C	C	3/29/11	3/30/11	I	TP	5-D TROPICAL, INC.
2011766732	CYOR	CYNOPS	ORIENTALIS	CHINESE DWARF	NEWT	LIV	500	NO	HK	HK	T	C	C	C	3/29/11	3/30/11	I	TP	5-D TROPICAL INC.
2011766732	TRIH	TRITURUS	HONGKONGENSIS	ALPINE	NEWT	LIV	1,600	NO	HK	HK	T	C	C	C	3/29/11	3/30/11	I	TP	5-D TROPICAL INC.
2011767323	NOVI	NOTOPHTHALMUS	VIRIDESCENS	EASTERN	NEWT	LIV	2	NO	US	CA	T	W	C	C	3/30/11	3/30/11	I	BN	Science Kit, Inc - Boreal
2011768775	CNP?	CYNOPS	SPECIES	FIREBELLY	NEWT	LIV	400	NO	CN	CN	T	C	C	C	4/2/11	4/2/11	I	AT	SUN PET LTD.
2011768892	CYOR	CYNOPS	ORIENTALIS	CHINESE DWARF	NEWT	LIV	200	NO	HK	HK	T	C	C	C	4/3/11	4/3/11	I	LA	DOLPHIN INTERNATIONAL
2011768892	PACB	PACHYTRITON	BREVIPES	TSITOU	NEWT	LIV	50	NO	HK	HK	T	C	C	C	4/3/11	4/3/11	I	LA	DOLPHIN INTERNATIONAL
2011768907	CYOR	CYNOPS	ORIENTALIS	CHINESE DWARF	NEWT	LIV	75	NO	SG	SG	T	C	C	C	4/3/11	4/3/11	I	LA	PET-SIAM INTERNATIONAL
2011768918	TRIT	TRITURUS	SPECIES		NEWT	LIV	100	NO	VS	HK	T	W	C	C	4/3/11	4/3/11	I	LA	AQUA-NAUTIC SPECIALIST
2011768919	TRIH	TRITURUS	HONGKONGENSIS	ALPINE	NEWT	LIV	200	NO	HK	HK	T	C	C	C	4/3/11	4/3/11	I	LA	AQUACO, INC.
2011769505	CYOR	CYNOPS	ORIENTALIS	CHINESE DWARF	NEWT	LIV	500	NO	HK	HK	T	C	C	C	4/4/11	4/6/11	I	TP	5-D TROPICAL INC.
2011769505	TRIH	TRITURUS	HONGKONGENSIS	ALPINE	NEWT	LIV	1,600	NO	HK	HK	T	C	C	C	4/4/11	4/6/11	I	TP	5-D TROPICAL INC.
2011769506	CYOR	CYNOPS	ORIENTALIS	CHINESE DWARF	NEWT	LIV	620	NO	HK	HK	T	C	C	C	4/4/11	4/6/11	I	TP	5-D TROPICAL, INC.
2011769847	CYOR	CYNOPS	ORIENTALIS	CHINESE DWARF	NEWT	LIV	3,000	NO	CN	CN	T	W	C	C	4/5/11	4/5/11	I	LA	Z IMPORTS INC.
2011769885	CYPY	CYNOPS	PYRRHOGASTER	JAPANESE FIREBELLY	NEWT	LIV	235	NO	HK	HK	T	C	C	C	4/5/11	4/3/11	I	NY	Transship Discounts Ltd.
2011771540	ANDA	ANDRIAS	DAVIDIANUS	CHINESE GIANT	SALAMANDER	LIV	1	NO	AT	AT	Z	C	C	C	4/7/11	4/7/11	I	LA	LOS ANGELES ZOO & BOTANICAKL GARDENS
2011773177	CNP?	CYNOPS	SPECIES	FIREBELLY	NEWT	LIV	540	NO	VS	HK	T	W	C	C	4/11/11	4/10/11	I	LA	AQUA-NAUTIC SPECIALIST
2011773328	CYOR	CYNOPS	ORIENTALIS	CHINESE DWARF	NEWT	LIV	725	NO	HK	HK	T	C	C	C	4/11/11	4/10/11	I	LA	DOLPHIN INTERNATIONAL
2011773651	CYPY	CYNOPS	PYRRHOGASTER	JAPANESE FIREBELLY	NEWT	LIV	310	NO	HK	HK	T	W	C	C	4/11/11	4/10/11	I	NY	ERDA INCORPORATED
2011774434	CYPY	CYNOPS	PYRRHOGASTER	JAPANESE FIREBELLY	NEWT	LIV	135	NO	HK	HK	T	C	C	C	4/12/11	4/10/11	I	NY	Transship Discounts Ltd.
2011774434	PACB	PACHYTRITON	BREVIPES	TSITOU	NEWT	LIV	90	NO	HK	HK	T	C	C	C	4/12/11	4/10/11	I	NY	Transship Discounts Ltd.
2011775991	NOVI	NOTOPHTHALMUS	VIRIDESCENS	EASTERN	NEWT	LIV	1	NO	US	CA	T	W	C	C	4/14/11	4/13/11	I	BN	Science Kit, Inc - Boreal
2011777063	CNP?	CYNOPS	SPECIES	FIREBELLY	NEWT	LIV	200	NO	CN	CN	T	C	C	C	4/16/11	4/16/11	I	AT	SUN PET LTD.
2011777102	CNP?	CYNOPS	SPECIES	FIREBELLY	NEWT	LIV	1,320	NO	VS	HK	T	W	C	C	4/17/11	4/17/11	I	LA	AQUA-NAUTIC SPECIALIST
2011777109	CYOR	CYNOPS	ORIENTALIS	CHINESE DWARF	NEWT	LIV	625	NO	HK	HK	T	C	C	C	4/17/11	4/17/11	I	LA	DOLPHIN INTERNATIONAL
2011777109	PACB	PACHYTRITON	BREVIPES	TSITOU	NEWT	LIV	50	NO	HK	HK	T	C	C	C	4/17/11	4/17/11	I	LA	DOLPHIN INTERNATIONAL
2011777119	TRIH	TRITURUS	HONGKONGENSIS	ALPINE	NEWT	LIV	35	NO	SG	SG	T	C	C	C	4/17/11	4/17/11	I	LA	PET-SIAM INTERNATIONAL
2011777125	TRIH	TRITURUS	HONGKONGENSIS	ALPINE	NEWT	LIV	39	NO	HK	HK	T	C	C	C	4/17/11	4/17/11	I	LA	AQUACO, INC.
2011777672	NOVI	NOTOPHTHALMUS	VIRIDESCENS	EASTERN	NEWT	LIV	3	NO	US	CA	T	W	C	C	4/18/11	4/18/11	I	BN	Science Kit, Inc - Boreal
2011778249	CYOR	CYNOPS	ORIENTALIS	CHINESE DWARF	NEWT	LIV	500	NO	HK	HK	T	C	C	C	4/19/11	4/20/11	I	TP	5-D TROPICAL INC.
2011779291	AMBA	AMBYSTOMA	MACULATUM	SPOTTED	SALAMANDER	LIV	175	NO	CA	CA	T	W	C	C	4/21/11	4/20/11	I	ME	VWR EDUCATION , LLC
2011779672	NOVI	NOTOPHTHALMUS	VIRIDESCENS	EASTERN	NEWT	LIV	2	NO	US	CA	T	W	C	C	4/21/11	4/20/11	I	BN	Science Kit, Inc - Boreal
2011780458	CYOR	CYNOPS	ORIENTALIS	CHINESE DWARF	NEWT	LIV	100	NO	HK	HK	T	C	C	C	4/24/11	4/24/11	I	LA	DOLPHIN INTERNATIONAL
2011780462	TRIH	TRITURUS	HONGKONGENSIS	ALPINE	NEWT	LIV	200	NO	HK	HK	T	C	C	C	4/24/11	4/24/11	I	LA	AQUACO, INC.
2011780470	PLAB	PACHYTRITON	LABIATUS	UNTERSTEIN'S	NEWT	LIV	100	NO	VS	HK	T	W	C	C	4/24/11	4/24/11	I	LA	AQUA-NAUTIC SPECIALIST
2011780904	NOVI	NOTOPHTHALMUS	VIRIDESCENS	EASTERN	NEWT	LIV	7	NO	US	CA	T	W	C	C	4/25/11	4/25/11	I	BN	Science Kit, Inc - Boreal
2011781600	TRIH	TRITURUS	HONGKONGENSIS	ALPINE	NEWT	LIV	1,600	NO	HK	HK	T	C	C	C	4/26/11	4/27/11	I	TP	5-D TROPICAL INC.
2011781838	CYPY	CYNOPS	PYRRHOGASTER	JAPANESE FIREBELLY	NEWT	LIV	140	NO	HK	HK	T	W	C	C	4/27/11	4/24/11	I	NY	ERDA INCORPORATED
2011783669	NOVI	NOTOPHTHALMUS	VIRIDESCENS	EASTERN	NEWT	LIV	3	NO	US	CA	T	W	C	C	4/27/11	4/27/11	I	BN	Science Kit, Inc - Boreal
2011784071	CYOR	CYNOPS	ORIENTALIS	CHINESE DWARF	NEWT	LIV	625	NO	HK	HK	T	C	C	C	5/1/11	5/1/11	I	LA	DOLPHIN INTERNATIONAL
2011784077	TRIH	TRITURUS	HONGKONGENSIS	ALPINE	NEWT	LIV	50	NO	HK	HK	T	C	C	C	5/1/11	5/1/11	I	LA	AQUACO, INC.
2011784082	TRIH	TRITURUS	HONGKONGENSIS	ALPINE	NEWT	LIV	245	NO	VS	HK	T	W	C	C	5/1/11	5/1/11	I	LA	AQUA-NAUTIC SPECIALIST
2011784851	NOVI	NOTOPHTHALMUS	VIRIDESCENS	EASTERN	NEWT	LIV	2	NO	US	CA	T	W	C	C	5/2/11	5/2/11	I	BN	Science Kit, Inc - Boreal
2011788310	CYOR	CYNOPS	ORIENTALIS	CHINESE DWARF	NEWT	LIV	775	NO	HK	HK	T	C	C	C	5/8/11	5/8/11	I	LA	DOLPHIN INTERNATIONAL
2011788310	PACB	PACHYTRITON	BREVIPES	TSITOU	NEWT	LIV	507	NO	HK	HK	T	C	C	C	5/8/11	5/8/11	I	LA	DOLPHIN INTERNATIONAL
2011788343	TRIT	TRITURUS	SPECIES		NEWT	LIV	45	NO	VS	HK	T	W	C	C	5/8/11	5/8/11	I	LA	AQUA-NAUTIC SPECIALIST
2011788802	AMBM	AMBYSTOMA	MEXICANUM		AXOLOTL	LIV	7	NO	CA	CA	M	C	C	C	5/9/11	5/9/11	I	CH	UNIVERSITY OF KENTUCKY
2011788987	CYPY	CYNOPS	PYRRHOGASTER	JAPANESE FIREBELLY	NEWT	LIV	80	NO	HK	HK	T	W	C	C	5/10/11	5/8/11	I	NY	ERDA INCORPORATED
2011789010	PACB	PACHYTRITON	BREVIPES	TSITOU	NEWT	LIV	30	NO	HK	HK	T	C	C	C	5/10/11	5/8/11	I	NY	Transship Discounts Ltd.
2011789010	CYPY	CYNOPS	PYRRHOGASTER	JAPANESE FIREBELLY	NEWT	LIV	210	NO	HK	HK	T	W	C	C	5/10/11	5/8/11	I	NY	Transship Discounts Ltd.
2011789044	PCHI	PARAMESOTRITON	CHINENSIS	CHINESE WARTY	NEWT	LIV	100	NO	CN	HK	T	W	C	C	5/10/11	5/10/11	I	DF	BUSHMASTER REPTILES INC.
2011789044	PLAB	PACHYTRITON	LABIATUS	UNTERSTEIN'S	NEWT	LIV	83	NO	CN	HK	T	W	C	C	5/10/11	5/10/11	I	DF	BUSHMASTER REPTILES INC.
2011790395	TRIT	TRITURUS	SPECIES		NEWT	LIV	390	NO	HK	HK	T	C	C	C	5/12/11	5/12/11	I	CH	AQUATICS INC
2011791507	CNP?	CYNOPS	SPECIES	FIREBELLY	NEWT	LIV	200	NO	CN	CN	T	C	C	C	5/14/11	5/14/11	I	AT	SUN PET LTD.
2011791550	CYOR	CYNOPS	ORIENTALIS	CHINESE DWARF	NEWT	LIV	425	NO	HK	HK	T	C	C	C	5/15/11	5/15/11	I	LA	DOLPHIN INTERNATIONAL
2011791550	PACB	PACHYTRITON	BREVIPES	TSITOU	NEWT	LIV	50	NO	HK	HK	T	C	C	C	5/15/11	5/15/11	I	LA	DOLPHIN INTERNATIONAL

2011791556	CYOR	CYNOPS	ORIENTALIS	CHINESE DWARF	NEWT	LIV	2,220	NO	CN	HK	T	W	C	C	5/15/11	5/15/11	I	LA	AQUA-NAUTIC SPECIALIST
2011791556	PLAB	PACHYTRITON	LABIATUS	UNTERSTEIN'S	NEWT	LIV	100	NO	CN	HK	T	W	C	C	5/15/11	5/15/11	I	LA	AQUA-NAUTIC SPECIALIST
2011791888	CYPY	CYNOPS	PYRRHOGASTER	JAPANESE FIREBELLY	NEWT	LIV	80	NO	HK	HK	T	W	C	C	5/16/11	5/15/11	I	NY	ERDA INCORPORATED
2011791915	CYPY	CYNOPS	PYRRHOGASTER	JAPANESE FIREBELLY	NEWT	LIV	25	NO	HK	HK	T	W	C	C	5/16/11	5/15/11	I	NY	Transship Discounts Ltd.
2011792677	CYOR	CYNOPS	ORIENTALIS	CHINESE DWARF	NEWT	LIV	1,440	NO	HK	HK	T	C	C	C	5/17/11	5/18/11	I	TP	5-D TROPICAL, INC.
2011794937	CYOR	CYNOPS	ORIENTALIS	CHINESE DWARF	NEWT	LIV	375	NO	HK	HK	T	C	C	C	5/22/11	5/22/11	I	LA	DOLPHIN INTERNATIONAL
2011794979	TRIH	TRITURUS	HONGKONGENSIS	ALPINE	NEWT	LIV	50	NO	HK	HK	T	C	C	C	5/22/11	5/22/11	I	LA	AQUACO, INC.
2011794981	TRIH	TRITURUS	HONGKONGENSIS	ALPINE	NEWT	LIV	75	NO	SG	SG	T	C	C	C	5/22/11	5/22/11	I	LA	PET-SIAM INTERNATIONAL
2011796308	TRIH	TRITURUS	HONGKONGENSIS	ALPINE	NEWT	LIV	1,600	NO	HK	HK	T	C	C	C	5/24/11	5/25/11	I	TP	5-D TROPICAL INC.
2011796985	CYPY	CYNOPS	PYRRHOGASTER	JAPANESE FIREBELLY	NEWT	LIV	60	NO	HK	HK	T	W	C	C	5/25/11	5/22/11	I	NY	Transship Discounts Ltd.
2011796985	PACB	PACHYTRITON	BREVIPES	TSITOU	NEWT	LIV	60	NO	HK	HK	T	C	C	C	5/25/11	5/22/11	I	NY	Transship Discounts Ltd.
2011797132	AMBM	AMBYSTOMA	MEXICANUM		AXOLOTL	LIV	300	NO	CZ	CZ	T	C	C	C	5/25/11	5/25/11	I	TP	JOSEPH TROPICAL
2011798435	CNP?	CYNOPS	SPECIES	FIREBELLY	NEWT	LIV	200	NO	CN	CN	T	C	C	C	5/28/11	5/28/11	I	AT	SUN PET LTD.
2011798485	CYOR	CYNOPS	ORIENTALIS	CHINESE DWARF	NEWT	LIV	375	NO	HK	HK	T	C	C	C	5/29/11	5/29/11	I	LA	DOLPHIN INTERNATIONAL
2011798506	TRIT	TRITURUS	SPECIES		NEWT	LIV	245	NO	VS	HK	T	W	C	C	5/29/11	5/29/11	I	LA	AQUA-NAUTIC SPECIALIST
2011799227	CYOR	CYNOPS	ORIENTALIS	CHINESE DWARF	NEWT	LIV	1,200	NO	HK	HK	T	C	C	C	5/31/11	6/1/11	I	TP	5-D TROPICAL, INC.
2011799249	CYPY	CYNOPS	PYRRHOGASTER	JAPANESE FIREBELLY	NEWT	LIV	105	NO	HK	HK	T	W	C	C	5/31/11	5/29/11	I	NY	Transship Discounts Ltd.
2011799249	PACB	PACHYTRITON	BREVIPES	TSITOU	NEWT	LIV	30	NO	HK	HK	T	C	C	C	5/31/11	5/29/11	I	NY	Transship Discounts Ltd.
2011799701	CYPY	CYNOPS	PYRRHOGASTER	JAPANESE FIREBELLY	NEWT	LIV	60	NO	HK	HK	T	W	C	C	6/1/11	5/29/11	I	NY	ERDA INCORPORATED
2011801921	CYOR	CYNOPS	ORIENTALIS	CHINESE DWARF	NEWT	LIV	455	NO	HK	HK	T	C	C	C	6/5/11	6/5/11	I	LA	DOLPHIN INTERNATIONAL
2011801958	TRIH	TRITURUS	HONGKONGENSIS	ALPINE	NEWT	LIV	50	NO	HK	HK	T	C	C	C	6/5/11	6/5/11	I	LA	AQUACO, INC.
2011802437	CYPY	CYNOPS	PYRRHOGASTER	JAPANESE FIREBELLY	NEWT	LIV	30	NO	HK	HK	T	W	C	C	6/6/11	6/5/11	I	NY	Transship Discounts Ltd.
2011803197	TRIH	TRITURUS	HONGKONGENSIS	ALPINE	NEWT	LIV	1,600	NO	HK	HK	T	C	C	C	6/7/11	6/8/11	I	TP	5-D TROPICAL INC.
2011803197	CYOR	CYNOPS	ORIENTALIS	CHINESE DWARF	NEWT	LIV	500	NO	HK	HK	T	C	C	C	6/7/11	6/8/11	I	TP	5-D TROPICAL INC.
2011805900	CYOR	CYNOPS	ORIENTALIS	CHINESE DWARF	NEWT	LIV	540	NO	HK	HK	T	C	C	C	6/12/11	6/12/11	I	LA	DOLPHIN INTERNATIONAL
2011805900	PACB	PACHYTRITON	BREVIPES	TSITOU	NEWT	LIV	50	NO	HK	HK	T	C	C	C	6/12/11	6/12/11	I	LA	DOLPHIN INTERNATIONAL
2011805913	TRIT	TRITURUS	SPECIES		NEWT	LIV	190	NO	VS	HK	T	W	C	C	6/12/11	6/12/11	I	LA	AQUA-NAUTIC SPECIALIST
2011809017	CYOR	CYNOPS	ORIENTALIS	CHINESE DWARF	NEWT	LIV	500	NO	HK	HK	T	C	C	C	6/19/11	6/19/11	I	LA	DOLPHIN INTERNATIONAL
2011809031	CYPY	CYNOPS	PYRRHOGASTER	JAPANESE FIREBELLY	NEWT	LIV	344	NO	CN	CN	T	C	C	C	6/19/11	6/19/11	I	LA	AQUA-NAUTIC SPECIALIST
2011810196	CYOR	CYNOPS	ORIENTALIS	CHINESE DWARF	NEWT	LIV	2,240	NO	HK	HK	T	C	C	C	6/21/11	6/22/11	I	TP	5-D TROPICAL, INC.
2011812029	CYPY	CYNOPS	PYRRHOGASTER	JAPANESE FIREBELLY	NEWT	LIV	200	NO	CN	CN	T	C	C	C	6/25/11	6/25/11	I	AT	SUN PET LTD.
2011812059	CYOR	CYNOPS	ORIENTALIS	CHINESE DWARF	NEWT	LIV	7,000	NO	CN	CN	T	W	C	C	6/26/11	6/26/11	I	LA	WORLDWIDE TRADING INC
2011812059	PLAB	PACHYTRITON	LABIATUS	UNTERSTEIN'S	NEWT	LIV	1,000	NO	CN	CN	T	W	C	C	6/26/11	6/26/11	I	LA	WORLDWIDE TRADING INC
2011812072	CYOR	CYNOPS	ORIENTALIS	CHINESE DWARF	NEWT	LIV	500	NO	HK	HK	T	C	C	C	6/26/11	6/26/11	I	LA	DOLPHIN INTERNATIONAL
2011812082	TRIH	TRITURUS	HONGKONGENSIS	ALPINE	NEWT	LIV	200	NO	HK	HK	T	C	C	C	6/26/11	6/26/11	I	LA	AQUACO, INC.
2011812098	CYPY	CYNOPS	PYRRHOGASTER	JAPANESE FIREBELLY	NEWT	LIV	3,600	NO	CN	CN	T	C	C	C	6/26/11	6/26/11	I	LA	AQUA-NAUTIC SPECIALIST
2011812236	PACB	PACHYTRITON	BREVIPES	TSITOU	NEWT	LIV	60	NO	HK	HK	T	C	C	C	6/27/11	6/19/11	I	NY	Transship Discounts Ltd.
2011813618	CYOR	CYNOPS	ORIENTALIS	CHINESE DWARF	NEWT	LIV	500	NO	HK	HK	T	C	C	C	6/28/11	6/29/11	I	TP	5-D TROPICAL INC.
2011813647	CYPY	CYNOPS	PYRRHOGASTER	JAPANESE FIREBELLY	NEWT	LIV	210	NO	HK	HK	T	W	C	C	6/28/11	6/26/11	I	NY	ERDA INCORPORATED
2011814401	CYPY	CYNOPS	PYRRHOGASTER	JAPANESE FIREBELLY	NEWT	LIV	140	NO	HK	HK	T	W	C	C	6/29/11	6/12/11	I	NY	ERDA INCORPORATED
2011815647	CYOR	CYNOPS	ORIENTALIS	CHINESE DWARF	NEWT	LIV	500	NO	HK	HK	T	C	C	C	7/3/11	7/3/11	I	LA	DOLPHIN INTERNATIONAL
2011815647	PACB	PACHYTRITON	BREVIPES	TSITOU	NEWT	LIV	150	NO	HK	HK	T	C	C	C	7/3/11	7/3/11	I	LA	DOLPHIN INTERNATIONAL
2011815663	CYPY	CYNOPS	PYRRHOGASTER	JAPANESE FIREBELLY	NEWT	LIV	810	NO	CN	CN	T	C	C	C	7/3/11	7/3/11	I	LA	AQUA-NAUTIC SPECIALIST
2011816549	CYOR	CYNOPS	ORIENTALIS	CHINESE DWARF	NEWT	LIV	500	NO	HK	HK	T	C	C	C	7/6/11	7/6/11	I	TP	5-D TROPICAL INC.
2011816549	TRIH	TRITURUS	HONGKONGENSIS	ALPINE	NEWT	LIV	1,600	NO	HK	HK	T	C	C	C	7/6/11	7/6/11	I	TP	5-D TROPICAL INC.
2011818411	CYPY	CYNOPS	PYRRHOGASTER	JAPANESE FIREBELLY	NEWT	LIV	315	NO	HK	HK	T	C	C	C	7/8/11	7/3/11	I	NY	Transship Discounts Ltd.
2011818626	CNP?	CYNOPS	SPECIES	FIREBELLY	NEWT	LIV	200	NO	CN	CN	T	C	C	C	7/9/11	7/9/11	I	AT	SUN PET LTD.
2011818676	CYPY	CYNOPS	PYRRHOGASTER	JAPANESE FIREBELLY	NEWT	LIV	2,660	NO	CN	CN	T	C	C	C	7/10/11	7/10/11	I	LA	AQUA-NAUTIC SPECIALIST
2011818704	CYOR	CYNOPS	ORIENTALIS	CHINESE DWARF	NEWT	LIV	750	NO	HK	HK	T	C	C	C	7/10/11	7/10/11	I	LA	DOLPHIN INTERNATIONAL
2011818704	PACB	PACHYTRITON	BREVIPES	TSITOU	NEWT	LIV	100	NO	HK	HK	T	C	C	C	7/10/11	7/10/11	I	LA	DOLPHIN INTERNATIONAL
2011819720	TRIH	TRITURUS	HONGKONGENSIS	ALPINE	NEWT	LIV	1,600	NO	HK	HK	T	C	C	C	7/12/11	7/13/11	I	TP	5-D TROPICAL INC.
2011819895	CYPY	CYNOPS	PYRRHOGASTER	JAPANESE FIREBELLY	NEWT	LIV	130	NO	HK	HK	T	C	C	C	7/12/11	7/10/11	I	NY	Transship Discounts Ltd.
2011821007	TYKW	TYLOTOTRITON	KWEICHOWENSIS	KWEICHOW CROCODILE	NEWT	LIV	200	NO	HK	HK	T	C	C	C	7/14/11	7/14/11	I	DF	BUSHMASTER REPTILES INC.
2011821007	TYTA	TYLOTOTRITON	TALIANGENSIS	PUSAKANG CROCODILE	NEWT	LIV	3	NO	HK	HK	T	C	C	C	7/14/11	7/14/11	I	DF	BUSHMASTER REPTILES INC.
2011821616	CYPY	CYNOPS	PYRRHOGASTER	JAPANESE FIREBELLY	NEWT	LIV	60	NO	JP	JP	T	F	C	C	7/14/11	6/16/11	I	LA	HERP TRADING INC
2011821616	CNP?	CYNOPS	SPECIES	FIREBELLY	NEWT	LIV	45	NO	JP	JP	T	F	C	C	7/14/11	6/16/11	I	LA	HERP TRADING INC
2011822294	CYOR	CYNOPS	ORIENTALIS	CHINESE DWARF	NEWT	LIV	1,000	NO	HK	HK	T	C	C	C	7/17/11	7/17/11	I	LA	DOLPHIN INTERNATIONAL
2011822294	PACB	PACHYTRITON	BREVIPES	TSITOU	NEWT	LIV	150	NO	HK	HK	T	C	C	C	7/17/11	7/17/11	I	LA	DOLPHIN INTERNATIONAL
2011822314	CYPY	CYNOPS	PYRRHOGASTER	JAPANESE FIREBELLY	NEWT	LIV	1,565	NO	CN	CN	T	C	C	C	7/17/11	7/17/11	I	LA	AQUA-NAUTIC SPECIALIST
2011823136	CYPY	CYNOPS	PYRRHOGASTER	JAPANESE FIREBELLY	NEWT	LIV	210	NO	HK	HK	T	W	C	C	7/19/11	6/19/11	I	NY	ERDA INCORPORATED

2011823298	TRIH	TRITURUS	HONGKONGENSIS	ALPINE	NEWT	LIV	1,600	NO	HK	HK	T	C	C	C	7/19/11	7/20/11	I	TP	5-D TROPICAL INC.
2011823876	CYPY	CYNOPS	PYRRHOGASTER	JAPANESE FIREBELLY	NEWT	LIV	150	NO	HK	HK	T	W	C	C	7/20/11	7/17/11	I	NY	ERDA INCORPORATED
2011824072	CNP?	CYNOPS	SPECIES	FIREBELLY	NEWT	LIV	3,000	NO	CN	CN	T	W	C	C	7/20/11	7/20/11	I	LA	Z IMPORTS INC.
2011824072	TYVE	TYLOTOTRITON	VERRUCOSUS	CROCODILE	NEWT	LIV	400	NO	CN	CN	T	W	C	C	7/20/11	7/20/11	I	LA	Z IMPORTS INC.
2011825757	CNP?	CYNOPS	SPECIES	FIREBELLY	NEWT	LIV	200	NO	CN	CN	T	C	C	C	7/23/11	7/23/11	I	AT	SUN PET LTD.
2011825814	CYOR	CYNOPS	ORIENTALIS	CHINESE DWARF	NEWT	LIV	1,125	NO	HK	HK	T	C	C	C	7/24/11	7/24/11	I	LA	DOLPHIN INTERNATIONAL
2011825841	CYPY	CYNOPS	PYRRHOGASTER	JAPANESE FIREBELLY	NEWT	LIV	1,620	NO	CN	CN	T	C	C	C	7/24/11	7/24/11	I	LA	AQUA-NAUTIC SPECIALIST
2011826198	CYPY	CYNOPS	PYRRHOGASTER	JAPANESE FIREBELLY	NEWT	LIV	105	NO	HK	HK	T	C	C	C	7/25/11	7/24/11	I	NY	Transship Discounts Ltd.
2011826198	PACB	PACHYTRITON	BREVIPES	TSITOU	NEWT	LIV	60	NO	HK	HK	T	C	C	C	7/25/11	7/24/11	I	NY	Transship Discounts Ltd.
2011826939	TRIH	TRITURUS	HONGKONGENSIS	ALPINE	NEWT	LIV	1,600	NO	HK	HK	T	C	C	C	7/26/11	7/27/11	I	TP	5-D TROPICAL INC.
2011827166	CYPY	CYNOPS	PYRRHOGASTER	JAPANESE FIREBELLY	NEWT	LIV	420	NO	HK	HK	T	W	C	C	7/26/11	7/24/11	I	NY	ERDA INCORPORATED
2011829712	TRIH	TRITURUS	HONGKONGENSIS	ALPINE	NEWT	LIV	50	NO	HK	HK	T	C	C	C	7/31/11	7/31/11	I	LA	AQUACO, INC.
2011829716	CYPY	CYNOPS	PYRRHOGASTER	JAPANESE FIREBELLY	NEWT	LIV	2,010	NO	CN	CN	T	C	C	C	7/31/11	7/31/11	I	LA	AQUA-NAUTIC SPECIALIST
2011829728	CYOR	CYNOPS	ORIENTALIS	CHINESE DWARF	NEWT	LIV	875	NO	HK	HK	T	C	C	C	7/31/11	7/31/11	I	LA	DOLPHIN INTERNATIONAL
2011829728	PACB	PACHYTRITON	BREVIPES	TSITOU	NEWT	LIV	100	NO	HK	HK	T	C	C	C	7/31/11	7/31/11	I	LA	DOLPHIN INTERNATIONAL
2011830261	CYPY	CYNOPS	PYRRHOGASTER	JAPANESE FIREBELLY	NEWT	LIV	80	NO	HK	HK	T	W	C	C	8/1/11	7/31/11	I	NY	ERDA INCORPORATED
2011831151	TRIH	TRITURUS	HONGKONGENSIS	ALPINE	NEWT	LIV	2,100	NO	HK	HK	T	C	C	C	8/3/11	8/3/11	I	TP	5-D TROPICAL INC.
2011833218	NOVI	NOTOPHTHALMUS	VIRIDESCENS	EASTERN	NEWT	LIV	48	NO	US	CA	T	W	C	C	8/5/11	8/5/11	I	BN	VWR EDUCATION LLC
2011833430	CNP?	CYNOPS	SPECIES	FIREBELLY	NEWT	LIV	200	NO	CN	CN	T	C	C	C	8/6/11	8/6/11	I	AT	SUN PET LTD.
2011833448	TRIT	TRITURUS	SPECIES		NEWT	LIV	1,540	NO	CN	HK	T	W	C	C	8/6/11	8/7/11	I	LA	AQUA-NAUTIC SPECIALIST
2011833507	CYOR	CYNOPS	ORIENTALIS	CHINESE DWARF	NEWT	LIV	500	NO	HK	HK	T	C	C	C	8/7/11	8/7/11	I	LA	DOLPHIN INTERNATIONAL
2011833507	PACB	PACHYTRITON	BREVIPES	TSITOU	NEWT	LIV	100	NO	HK	HK	T	C	C	C	8/7/11	8/7/11	I	LA	DOLPHIN INTERNATIONAL
2011833680	CYPY	CYNOPS	PYRRHOGASTER	JAPANESE FIREBELLY	NEWT	LIV	105	NO	HK	HK	T	C	C	C	8/8/11	8/7/11	I	NY	Transship Discounts Ltd.
2011834101	NOVI	NOTOPHTHALMUS	VIRIDESCENS	EASTERN	NEWT	LIV	2	NO	US	CA	T	W	C	C	8/8/11	8/8/11	I	BN	Science Kit, Inc - Boreal
2011834685	CYOR	CYNOPS	ORIENTALIS	CHINESE DWARF	NEWT	LIV	500	NO	HK	HK	T	C	C	C	8/9/11	8/10/11	I	TP	5-D TROPICAL INC.
2011836158	TYL?	TYLOTOTRITON	SPECIES	CROCODILE	NEWT	LIV	500	NO	CI	CN	T	W	C	C	8/11/11	8/11/11	I	LA	Z IMPORTS INC.
2011836277	TYKW	TYLOTOTRITON	KWEICHOWENSIS	KWEICHOW CROCODILE	NEWT	LIV	542	NO	HK	HK	T	F	C	C	8/11/11	8/10/11	I	SF	WONDERFUL WORLD PET SUPPLIERS
2011836277	TYTA	TYLOTOTRITON	TALIANGENSIS	PUSAKANG CROCODILE	NEWT	LIV	97	NO	HK	HK	T	F	C	C	8/11/11	8/10/11	I	SF	WONDERFUL WORLD PET SUPPLIERS
2011836277	TYSH	TYLOTOTRITON	SHANJING	MANDARIN	NEWT	LIV	190	NO	HK	HK	T	F	C	C	8/11/11	8/10/11	I	SF	WONDERFUL WORLD PET SUPPLIERS
2011837357	CYOR	CYNOPS	ORIENTALIS	CHINESE DWARF	NEWT	LIV	500	NO	HK	HK	T	C	C	C	8/14/11	8/14/11	I	LA	DOLPHIN INTERNATIONAL
2011837357	PACB	PACHYTRITON	BREVIPES	TSITOU	NEWT	LIV	150	NO	HK	HK	T	C	C	C	8/14/11	8/14/11	I	LA	DOLPHIN INTERNATIONAL
2011837368	CNP?	CYNOPS	SPECIES	FIREBELLY	NEWT	LIV	280	NO	VS	HK	T	W	C	C	8/14/11	8/14/11	I	LA	AQUA-NAUTIC SPECIALIST
2011838177	CYPY	CYNOPS	PYRRHOGASTER	JAPANESE FIREBELLY	NEWT	LIV	300	NO	HK	HK	T	W	C	C	8/15/11	8/14/11	I	NY	ERDA INCORPORATED
2011838843	TRIH	TRITURUS	HONGKONGENSIS	ALPINE	NEWT	LIV	1,600	NO	HK	HK	T	C	C	C	8/16/11	8/17/11	I	TP	5-D TROPICAL INC.
2011841225	CYOR	CYNOPS	ORIENTALIS	CHINESE DWARF	NEWT	LIV	250	NO	HK	HK	T	C	C	C	8/21/11	8/21/11	I	LA	DOLPHIN INTERNATIONAL
2011841225	PACB	PACHYTRITON	BREVIPES	TSITOU	NEWT	LIV	100	NO	HK	HK	T	C	C	C	8/21/11	8/21/11	I	LA	DOLPHIN INTERNATIONAL
2011841239	CNP?	CYNOPS	SPECIES	FIREBELLY	NEWT	LIV	2,360	NO	CN	HK	T	W	C	C	8/21/11	8/21/11	I	LA	AQUA-NAUTIC SPECIALIST
2011841239	TRIT	TRITURUS	SPECIES		NEWT	LIV	144	NO	CN	HK	T	W	C	C	8/21/11	8/21/11	I	LA	AQUA-NAUTIC SPECIALIST
2011841241	TRIH	TRITURUS	HONGKONGENSIS	ALPINE	NEWT	LIV	300	NO	HK	HK	T	C	C	C	8/21/11	8/21/11	I	LA	AQUACO, INC.
2011841248	CNP?	CYNOPS	SPECIES	FIREBELLY	NEWT	LIV	200	NO	CN	CN	T	C	C	C	8/21/11	8/21/11	I	AT	SUN PET LTD.
2011841268	CYPY	CYNOPS	PYRRHOGASTER	JAPANESE FIREBELLY	NEWT	LIV	140	NO	HK	HK	T	W	C	C	8/21/11	8/21/11	I	NY	ERDA INCORPORATED
2011842154	TRIH	TRITURUS	HONGKONGENSIS	ALPINE	NEWT	LIV	2,000	NO	HK	HK	T	C	C	C	8/23/11	8/24/11	I	TP	5-D TROPICAL INC.
2011844364	CYOR	CYNOPS	ORIENTALIS	CHINESE DWARF	NEWT	LIV	925	NO	HK	HK	T	C	C	C	8/28/11	8/28/11	I	LA	DOLPHIN INTERNATIONAL
2011844364	PACB	PACHYTRITON	BREVIPES	TSITOU	NEWT	LIV	150	NO	HK	HK	T	C	C	C	8/28/11	8/28/11	I	LA	DOLPHIN INTERNATIONAL
2011844371	TRIT	TRITURUS	SPECIES		NEWT	LIV	90	NO	CN	HK	T	W	C	C	8/28/11	8/28/11	I	LA	AQUA-NAUTIC SPECIALIST
2011844371	CNP?	CYNOPS	SPECIES	FIREBELLY	NEWT	LIV	280	NO	CN	HK	T	W	C	C	8/28/11	8/28/11	I	LA	AQUA-NAUTIC SPECIALIST
2011844391	TRIH	TRITURUS	HONGKONGENSIS	ALPINE	NEWT	LIV	50	NO	TH	HK	T	C	C	C	8/28/11	8/28/11	I	LA	AQUACO, INC.
2011845387	TRIH	TRITURUS	HONGKONGENSIS	ALPINE	NEWT	LIV	1,600	NO	HK	HK	T	C	C	C	8/30/11	8/31/11	I	TP	5-D TROPICAL INC.
2011845387	CYOR	CYNOPS	ORIENTALIS	CHINESE DWARF	NEWT	LIV	500	NO	HK	HK	T	C	C	C	8/30/11	8/31/11	I	TP	5-D TROPICAL INC.
2011847320	CYOR	CYNOPS	ORIENTALIS	CHINESE DWARF	NEWT	LIV	520	NO	HK	HK	T	C	C	C	9/4/11	9/4/11	I	LA	DOLPHIN INTERNATIONAL
2011847343	CNP?	CYNOPS	SPECIES	FIREBELLY	NEWT	LIV	278	NO	VS	HK	T	W	C	C	9/4/11	9/4/11	I	LA	AQUA-NAUTIC SPECIALIST
2011848039	TRIH	TRITURUS	HONGKONGENSIS	ALPINE	NEWT	LIV	1,600	NO	HK	HK	T	C	C	C	9/6/11	9/7/11	I	TP	5-D TROPICAL INC.
2011849208	CYPY	CYNOPS	PYRRHOGASTER	JAPANESE FIREBELLY	NEWT	LIV	105	NO	HK	HK	T	C	C	C	9/7/11	9/4/11	I	NY	Transship Discounts Ltd.
2011849208	PACB	PACHYTRITON	BREVIPES	TSITOU	NEWT	LIV	60	NO	HK	HK	T	C	C	C	9/7/11	9/4/11	I	NY	Transship Discounts Ltd.
2011849465	NOVI	NOTOPHTHALMUS	VIRIDESCENS	EASTERN	NEWT	LIV	7	NO	US	CA	T	W	C	C	9/8/11	9/7/11	I	BN	Science Kit, Inc - Boreal
2011849521	CYPY	CYNOPS	PYRRHOGASTER	JAPANESE FIREBELLY	NEWT	LIV	230	NO	HK	HK	T	W	C	C	9/8/11	9/4/11	I	NY	ERDA INCORPORATED
2011850755	CYOR	CYNOPS	ORIENTALIS	CHINESE DWARF	NEWT	LIV	1,150	NO	HK	HK	T	C	C	C	9/11/11	9/11/11	I	LA	DOLPHIN INTERNATIONAL
2011850755	PACB	PACHYTRITON	BREVIPES	TSITOU	NEWT	LIV	50	NO	HK	HK	T	C	C	C	9/11/11	9/11/11	I	LA	DOLPHIN INTERNATIONAL
2011850774	CYOR	CYNOPS	ORIENTALIS	CHINESE DWARF	NEWT	LIV	2,820	NO	VS	HK	T	W	C	C	9/11/11	9/11/11	I	LA	AQUA-NAUTIC SPECIALIST
2011850774	TRIT	TRITURUS	SPECIES		NEWT	LIV	45	NO	VS	HK	T	W	C	C	9/11/11	9/11/11	I	LA	AQUA-NAUTIC SPECIALIST

2011851055	CYPY	CYNOPS	PYRRHOGASTER	JAPANESE FIREBELLY	NEWT	LIV	201	NO	HK	HK	T	C	C	C	9/12/11	9/11/11	I	NY	Transship Discounts Ltd.
2011851055	PACB	PACHYTRITON	BREVIPES	TSITOU	NEWT	LIV	60	NO	HK	HK	T	C	C	C	9/12/11	9/11/11	I	NY	Transship Discounts Ltd.
2011852335	PLWA	PLEURODELES	WALTL	IBERIAN RIBBED	NEWT	LIV	50	NO	FR	FR	T	C	C	C	9/13/11	8/16/11	I	MI	TWO AMIGOS IMPORT & EXPORT, INC.
2011852650	NOVI	NOTOPHTHALMUS	VIRIDESCENS	EASTERN	NEWT	LIV	1	NO	US	CA	T	W	C	C	9/14/11	9/14/11	I	BN	Science Kit, Inc - Boreal
2011852676	NOVI	NOTOPHTHALMUS	VIRIDESCENS	EASTERN	NEWT	LIV	5	NO	US	CA	T	W	C	C	9/13/11	9/12/11	I	BN	Science Kit, Inc - Boreal
2011853224	TYSH	TYLOTOTRITON	SHANJING	MANDARIN	NEWT	LIV	10	NO	DE	DE	T	C	C	C	9/15/11	9/15/11	I	MI	REPTILE INDUSTRIES, INC.
2011854274	CYOR	CYNOPS	ORIENTALIS	CHINESE DWARF	NEWT	LIV	1,250	NO	HK	HK	T	C	C	C	9/18/11	9/18/11	I	LA	DOLPHIN INTERNATIONAL
2011854274	PACB	PACHYTRITON	BREVIPES	TSITOU	NEWT	LIV	150	NO	HK	HK	T	C	C	C	9/18/11	9/18/11	I	LA	DOLPHIN INTERNATIONAL
2011854303	TRIH	TRITURUS	HONGKONGENSIS	ALPINE	NEWT	LIV	50	NO	HK	HK	T	C	C	C	9/18/11	9/18/11	I	LA	AQUACO, INC.
2011854580	CYPY	CYNOPS	PYRRHOGASTER	JAPANESE FIREBELLY	NEWT	LIV	290	NO	HK	HK	T	W	C	C	9/19/11	9/18/11	I	NY	ERDA INCORPORATED
2011854899	NOVI	NOTOPHTHALMUS	VIRIDESCENS	EASTERN	NEWT	LIV	5	NO	US	CA	T	W	C	C	9/19/11	9/19/11	I	BN	Science Kit, Inc - Boreal
2011855608	TRIH	TRITURUS	HONGKONGENSIS	ALPINE	NEWT	LIV	1,600	NO	HK	HK	T	C	C	C	9/20/11	9/21/11	I	TP	5-D TROPICAL INC.
2011855608	CYOR	CYNOPS	ORIENTALIS	CHINESE DWARF	NEWT	LIV	500	NO	HK	HK	T	C	C	C	9/20/11	9/21/11	I	TP	5-D TROPICAL INC.
2011858096	CYOR	CYNOPS	ORIENTALIS	CHINESE DWARF	NEWT	LIV	925	NO	HK	HK	T	C	C	C	9/25/11	9/25/11	I	LA	DOLPHIN INTERNATIONAL
2011858096	PACB	PACHYTRITON	BREVIPES	TSITOU	NEWT	LIV	150	NO	HK	HK	T	C	C	C	9/25/11	9/25/11	I	LA	DOLPHIN INTERNATIONAL
2011858122	TRIH	TRITURUS	HONGKONGENSIS	ALPINE	NEWT	LIV	200	NO	HK	HK	T	C	C	C	9/25/11	9/25/11	I	LA	AQUACO, INC.
2011858132	TRIH	TRITURUS	HONGKONGENSIS	ALPINE	NEWT	LIV	75	NO	SG	SG	T	C	C	C	9/25/11	9/25/11	I	LA	PET-SIAM INTERNATIONAL
2011858316	CYPY	CYNOPS	PYRRHOGASTER	JAPANESE FIREBELLY	NEWT	LIV	230	NO	HK	HK	T	W	C	C	9/26/11	9/25/11	I	NY	ERDA INCORPORATED
2011858378	PACB	PACHYTRITON	BREVIPES	TSITOU	NEWT	LIV	60	NO	HK	HK	T	C	C	C	9/26/11	9/25/11	I	NY	Transship Discounts Ltd.
2011859310	TRIH	TRITURUS	HONGKONGENSIS	ALPINE	NEWT	LIV	1,600	NO	HK	HK	T	C	C	C	9/27/11	9/28/11	I	TP	5-D TROPICAL INC.
2011859310	CYOR	CYNOPS	ORIENTALIS	CHINESE DWARF	NEWT	LIV	500	NO	HK	HK	T	C	C	C	9/27/11	9/28/11	I	TP	5-D TROPICAL INC.
2011861791	CNP?	CYNOPS	SPECIES	FIREBELLY	NEWT	LIV	200	NO	CN	CN	T	C	C	C	10/1/11	10/1/11	I	AT	SUN PET LTD.
2011861857	CYOR	CYNOPS	ORIENTALIS	CHINESE DWARF	NEWT	LIV	1,000	NO	CN	HK	T	C	C	C	10/2/11	10/2/11	I	LA	DOLPHIN INTERNATIONAL
2011861857	CYPY	CYNOPS	PYRRHOGASTER	JAPANESE FIREBELLY	NEWT	LIV	150	NO	CN	HK	T	C	C	C	10/2/11	10/2/11	I	LA	DOLPHIN INTERNATIONAL
2011862894	TRIH	TRITURUS	HONGKONGENSIS	ALPINE	NEWT	LIV	1,600	NO	HK	HK	T	C	C	C	10/4/11	10/5/11	I	TP	5-D TROPICAL INC.
2011862894	CYOR	CYNOPS	ORIENTALIS	CHINESE DWARF	NEWT	LIV	500	NO	HK	HK	T	C	C	C	10/4/11	10/5/11	I	TP	5-D TROPICAL INC.
2011864550	PACB	PACHYTRITON	BREVIPES	TSITOU	NEWT	LIV	60	NO	HK	HK	T	C	C	C	10/7/11	10/2/11	I	NY	Transship Discounts Ltd.
2011864550	CYPY	CYNOPS	PYRRHOGASTER	JAPANESE FIREBELLY	NEWT	LIV	105	NO	HK	HK	T	C	C	C	10/7/11	10/2/11	I	NY	Transship Discounts Ltd.
2011864803	CYPY	CYNOPS	PYRRHOGASTER	JAPANESE FIREBELLY	NEWT	LIV	80	NO	HK	HK	T	W	C	C	10/7/11	10/2/11	I	NY	ERDA INCORPORATED
2011865058	CYOR	CYNOPS	ORIENTALIS	CHINESE DWARF	NEWT	LIV	500	NO	CN	CN	T	C	C	C	10/9/11	10/9/11	I	LA	DOLPHIN INTERNATIONAL
2011865058	PACB	PACHYTRITON	BREVIPES	TSITOU	NEWT	LIV	100	NO	CN	CN	T	C	C	C	10/9/11	10/9/11	I	LA	DOLPHIN INTERNATIONAL
2011865094	CNP?	CYNOPS	SPECIES	FIREBELLY	NEWT	LIV	600	NO	VS	HK	T	W	C	C	10/9/11	10/9/11	I	LA	AQUA-NAUTIC SPECIALIST
2011865094	TRIT	TRITURUS	SPECIES		NEWT	LIV	45	NO	VS	HK	T	W	C	C	10/9/11	10/9/11	I	LA	AQUA-NAUTIC SPECIALIST
2011865101	TRIH	TRITURUS	HONGKONGENSIS	ALPINE	NEWT	LIV	50	NO	HK	HK	T	C	C	C	10/9/11	10/9/11	I	LA	AQUACO, INC.
2011865428	CYPY	CYNOPS	PYRRHOGASTER	JAPANESE FIREBELLY	NEWT	LIV	210	NO	HK	HK	T	C	C	C	10/11/11	10/9/11	I	NY	Transship Discounts Ltd.
2011865677	CYOR	CYNOPS	ORIENTALIS	CHINESE DWARF	NEWT	LIV	320	NO	HK	HK	T	C	C	C	10/11/11	10/12/11	I	TP	5-D TROPICAL, INC.
2011865704	CYPY	CYNOPS	PYRRHOGASTER	JAPANESE FIREBELLY	NEWT	LIV	210	NO	HK	HK	T	W	C	C	10/11/11	10/9/11	I	NY	ERDA INCORPORATED
2011868160	CNP?	CYNOPS	SPECIES	FIREBELLY	NEWT	LIV	200	NO	CN	CN	T	C	C	C	10/15/11	10/15/11	I	AT	SUN PET LTD.
2011868207	CYOR	CYNOPS	ORIENTALIS	CHINESE DWARF	NEWT	LIV	500	NO	HK	HK	T	C	C	C	10/16/11	10/16/11	I	LA	DOLPHIN INTERNATIONAL
2011868207	PACB	PACHYTRITON	BREVIPES	TSITOU	NEWT	LIV	50	NO	HK	HK	T	C	C	C	10/16/11	10/16/11	I	LA	DOLPHIN INTERNATIONAL
2011868240	TRIT	TRITURUS	SPECIES		NEWT	LIV	45	NO	VS	HK	T	W	C	C	10/16/11	10/16/11	I	LA	AQUA-NAUTIC SPECIALIST
2011868250	TRIH	TRITURUS	HONGKONGENSIS	ALPINE	NEWT	LIV	113	NO	SG	SG	T	C	C	C	10/16/11	10/16/11	I	LA	PET-SIAM INTERNATIONAL
2011868584	CYPY	CYNOPS	PYRRHOGASTER	JAPANESE FIREBELLY	NEWT	LIV	235	NO	HK	HK	T	C	C	C	10/17/11	10/16/11	I	NY	Transship Discounts Ltd.
2011868584	PACB	PACHYTRITON	BREVIPES	TSITOU	NEWT	LIV	60	NO	HK	HK	T	C	C	C	10/17/11	10/16/11	I	NY	Transship Discounts Ltd.
2011868605	CYPY	CYNOPS	PYRRHOGASTER	JAPANESE FIREBELLY	NEWT	LIV	150	NO	HK	HK	T	W	C	C	10/17/11	10/16/11	I	NY	ERDA INCORPORATED
2011869416	TRIH	TRITURUS	HONGKONGENSIS	ALPINE	NEWT	LIV	1,600	NO	HK	HK	T	C	C	C	10/18/11	10/19/11	I	TP	5-D TROPICAL INC.
2011869416	CYOR	CYNOPS	ORIENTALIS	CHINESE DWARF	NEWT	LIV	500	NO	HK	HK	T	C	C	C	10/18/11	10/19/11	I	TP	5-D TROPICAL INC.
2011870086	NOVI	NOTOPHTHALMUS	VIRIDESCENS	EASTERN	NEWT	LIV	2	NO	US	CA	T	W	C	C	10/19/11	10/17/11	I	BN	Science Kit, Inc - Boreal
2011870783	NOVI	NOTOPHTHALMUS	VIRIDESCENS	EASTERN	NEWT	LIV	1	NO	US	CA	T	W	C	C	10/20/11	10/19/11	I	BN	Science Kit, Inc - Boreal
2011871765	CYOR	CYNOPS	ORIENTALIS	CHINESE DWARF	NEWT	LIV	500	NO	HK	HK	T	C	C	C	10/23/11	10/23/11	I	LA	DOLPHIN INTERNATIONAL
2011871765	PACB	PACHYTRITON	BREVIPES	TSITOU	NEWT	LIV	150	NO	HK	HK	T	C	C	C	10/23/11	10/23/11	I	LA	DOLPHIN INTERNATIONAL
2011871787	TRIT	TRITURUS	SPECIES		NEWT	LIV	190	NO	VS	HK	T	W	C	C	10/23/11	10/23/11	I	LA	AQUA-NAUTIC SPECIALIST
2011872148	CYPY	CYNOPS	PYRRHOGASTER	JAPANESE FIREBELLY	NEWT	LIV	105	NO	HK	HK	T	C	C	C	10/24/11	10/23/11	I	NY	Transship Discounts Ltd.
2011872345	NOVI	NOTOPHTHALMUS	VIRIDESCENS	EASTERN	NEWT	LIV	2	NO	US	CA	T	W	C	C	10/24/11	10/24/11	I	BN	Science Kit, Inc - Boreal
2011872852	TRIH	TRITURUS	HONGKONGENSIS	ALPINE	NEWT	LIV	1,600	NO	HK	HK	T	C	C	C	10/25/11	10/26/11	I	TP	5-D TROPICAL INC.
2011872852	CYOR	CYNOPS	ORIENTALIS	CHINESE DWARF	NEWT	LIV	500	NO	HK	HK	T	C	C	C	10/25/11	10/26/11	I	TP	5-D TROPICAL INC.
2011876457	TRIT	TRITURUS	SPECIES		NEWT	LIV	45	NO	VS	HK	T	W	C	C	10/30/11	10/30/11	I	LA	AQUA-NAUTIC SPECIALIST
2011876462	TRIH	TRITURUS	HONGKONGENSIS	ALPINE	NEWT	LIV	110	NO	SG	SG	T	C	C	C	10/30/11	10/30/11	I	LA	PET-SIAM INTERNATIONAL
2011877694	TRIH	TRITURUS	HONGKONGENSIS	ALPINE	NEWT	LIV	1,600	NO	HK	HK	T	C	C	C	11/1/11	11/2/11	I	TP	5-D TROPICAL INC.
2011877694	CYOR	CYNOPS	ORIENTALIS	CHINESE DWARF	NEWT	LIV	500	NO	HK	HK	T	C	C	C	11/1/11	11/2/11	I	TP	5-D TROPICAL INC.

2011877918	CNP?	CYNOPS	SPECIES	FIREBELLY	NEWT	LIV	1	NO	US	CA	T	W	C	C	11/1/11	10/31/11	I	BN	Science Kit, Inc - Boreal
2011880636	CYOR	CYNOPS	ORIENTALIS	CHINESE DWARF	NEWT	LIV	1,125	NO	HK	HK	T	C	C	C	11/6/11	11/6/11	I	LA	DOLPHIN INTERNATIONAL
2011880636	PACB	PACHYTRITON	BREVIPES	TSITOU	NEWT	LIV	200	NO	HK	HK	T	C	C	C	11/6/11	11/6/11	I	LA	DOLPHIN INTERNATIONAL
2011880659	CNP?	CYNOPS	SPECIES	FIREBELLY	NEWT	LIV	440	NO	VS	HK	T	W	C	C	11/6/11	11/6/11	I	LA	AQUA-NAUTIC SPECIALIST
2011880659	PLAB	PACHYTRITON	LABIATUS	UNTERSTEIN'S	NEWT	LIV	290	NO	VS	HK	T	W	C	C	11/6/11	11/6/11	I	LA	AQUA-NAUTIC SPECIALIST
2011881496	CYOR	CYNOPS	ORIENTALIS	CHINESE DWARF	NEWT	LIV	500	NO	HK	HK	T	C	C	C	11/8/11	11/9/11	I	TP	5-D TROPICAL INC.
2011881861	NOVI	NOTOPHTHALMUS	VIRIDESCENS	EASTERN	NEWT	LIV	3	NO	US	CA	T	W	C	C	11/8/11	11/7/11	I	BN	Science Kit, Inc - Boreal
2011882198	CYPY	CYNOPS	PYRRHOGASTER	JAPANESE FIREBELLY	NEWT	LIV	80	NO	HK	HK	T	W	C	C	11/9/11	11/6/11	I	NY	ERDA INCORPORATED
2011883575	NOVI	NOTOPHTHALMUS	VIRIDESCENS	EASTERN	NEWT	LIV	1	NO	US	CA	T	W	C	C	11/10/11	11/9/11	I	BN	Science Kit, Inc - Boreal
2011883988	CYOR	CYNOPS	ORIENTALIS	CHINESE DWARF	NEWT	LIV	500	NO	HK	HK	T	C	C	C	11/13/11	11/13/11	I	LA	DOLPHIN INTERNATIONAL
2011883988	PACB	PACHYTRITON	BREVIPES	TSITOU	NEWT	LIV	50	NO	HK	HK	T	C	C	C	11/13/11	11/13/11	I	LA	DOLPHIN INTERNATIONAL
2011884006	TRIH	TRITURUS	HONGKONGENSIS	ALPINE	NEWT	LIV	35	NO	SG	SG	T	C	C	C	11/13/11	11/13/11	I	LA	PET-SIAM INTERNATIONAL
2011884015	CNP?	CYNOPS	SPECIES	FIREBELLY	NEWT	LIV	1,320	NO	CN	HK	T	W	C	C	11/13/11	11/13/11	I	LA	AQUA-NAUTIC SPECIALIST
2011885601	TRIH	TRITURUS	HONGKONGENSIS	ALPINE	NEWT	LIV	1,600	NO	HK	HK	T	C	C	C	11/15/11	11/16/11	I	TP	5-D TROPICAL INC.
2011886644	PACB	PACHYTRITON	BREVIPES	TSITOU	NEWT	LIV	60	NO	HK	HK	T	C	C	C	11/16/11	11/13/11	I	NY	Transship Discounts Ltd.
2011888241	CYOR	CYNOPS	ORIENTALIS	CHINESE DWARF	NEWT	LIV	250	NO	HK	HK	T	C	C	C	11/20/11	11/20/11	I	LA	DOLPHIN INTERNATIONAL
2011888241	PACB	PACHYTRITON	BREVIPES	TSITOU	NEWT	LIV	50	NO	HK	HK	T	C	C	C	11/20/11	11/20/11	I	LA	DOLPHIN INTERNATIONAL
2011888260	TRIT	TRITURUS	SPECIES		NEWT	LIV	150	NO	VS	HK	T	W	C	C	11/20/11	11/20/11	I	LA	AQUA-NAUTIC SPECIALIST
2011888270	TRIH	TRITURUS	HONGKONGENSIS	ALPINE	NEWT	LIV	290	NO	SG	SG	T	C	C	C	11/20/11	11/20/11	I	LA	PET-SIAM INTERNATIONAL
2011890214	NOVI	NOTOPHTHALMUS	VIRIDESCENS	EASTERN	NEWT	LIV	2	NO	US	CA	T	W	C	C	11/23/11	11/21/11	I	BN	Science Kit, Inc - Boreal
2011890573	AMBM	AMBYSTOMA	MEXICANUM		AXOLOTL	LIV	50	NO	DE	DE	T	C	C	C	11/25/11	11/21/11	I	MI	NAME REMOVED
2011891043	CNP?	CYNOPS	SPECIES	FIREBELLY	NEWT	LIV	200	NO	CN	CN	T	C	C	C	11/26/11	11/26/11	I	AT	SUN PET LTD.
2011891136	CYOR	CYNOPS	ORIENTALIS	CHINESE DWARF	NEWT	LIV	250	NO	HK	HK	T	C	C	C	11/27/11	11/27/11	I	LA	DOLPHIN INTERNATIONAL
2011891136	PACB	PACHYTRITON	BREVIPES	TSITOU	NEWT	LIV	100	NO	HK	HK	T	C	C	C	11/27/11	11/27/11	I	LA	DOLPHIN INTERNATIONAL
2011891227	CYPY	CYNOPS	PYRRHOGASTER	JAPANESE FIREBELLY	NEWT	LIV	60	NO	HK	HK	T	W	C	C	11/28/11	11/27/11	I	NY	ERDA INCORPORATED
2011892137	PACB	PACHYTRITON	BREVIPES	TSITOU	NEWT	LIV	60	NO	HK	HK	T	C	C	C	11/29/11	11/20/11	I	NY	Transship Discounts Ltd.
2011892137	CYPY	CYNOPS	PYRRHOGASTER	JAPANESE FIREBELLY	NEWT	LIV	105	NO	HK	HK	T	C	C	C	11/29/11	11/20/11	I	NY	Transship Discounts Ltd.
2011892172	TRIH	TRITURUS	HONGKONGENSIS	ALPINE	NEWT	LIV	1,600	NO	HK	HK	T	C	C	C	11/29/11	11/30/11	I	TP	5-D TROPICAL INC.
2011892172	CYOR	CYNOPS	ORIENTALIS	CHINESE DWARF	NEWT	LIV	500	NO	HK	HK	T	C	C	C	11/29/11	11/30/11	I	TP	5-D TROPICAL INC.
2011894012	CNP?	CYNOPS	SPECIES	FIREBELLY	NEWT	LIV	3	NO	US	CA	T	W	C	C	12/1/11	11/30/11	I	BN	Science Kit, Inc - Boreal
2011895850	CYOR	CYNOPS	ORIENTALIS	CHINESE DWARF	NEWT	LIV	1,375	NO	HK	HK	T	C	C	C	12/4/11	12/4/11	I	LA	DOLPHIN INTERNATIONAL
2011895850	PACB	PACHYTRITON	BREVIPES	TSITOU	NEWT	LIV	250	NO	HK	HK	T	C	C	C	12/4/11	12/4/11	I	LA	DOLPHIN INTERNATIONAL
2011895869	CNP?	CYNOPS	SPECIES	FIREBELLY	NEWT	LIV	1,760	NO	VS	HK	T	W	C	C	12/4/11	12/4/11	I	LA	AQUA-NAUTIC SPECIALIST
2011896229	CYPY	CYNOPS	PYRRHOGASTER	JAPANESE FIREBELLY	NEWT	LIV	105	NO	HK	HK	T	C	C	C	12/5/11	12/4/11	I	NY	Transship Discounts Ltd.
2011897813	TRIH	TRITURUS	HONGKONGENSIS	ALPINE	NEWT	LIV	1,600	NO	HK	HK	T	C	C	C	12/6/11	12/7/11	I	TP	5-D TROPICAL INC.
2011897813	CYOR	CYNOPS	ORIENTALIS	CHINESE DWARF	NEWT	LIV	500	NO	HK	HK	T	C	C	C	12/6/11	12/7/11	I	TP	5-D TROPICAL INC.
2011897860	CYOR	CYNOPS	ORIENTALIS	CHINESE DWARF	NEWT	LIV	60	NO	HK	HK	T	C	C	C	12/6/11	12/7/11	I	TP	5-D TROPICAL, INC.
2011898958	CNP?	CYNOPS	SPECIES	FIREBELLY	NEWT	LIV	2	NO	US	CA	T	W	C	C	12/7/11	12/5/11	I	BN	Science Kit, Inc - Boreal
2011900771	CYOR	CYNOPS	ORIENTALIS	CHINESE DWARF	NEWT	LIV	300	NO	HK	HK	T	C	C	C	12/11/11	12/11/11	I	LA	DOLPHIN INTERNATIONAL
2011900771	PACB	PACHYTRITON	BREVIPES	TSITOU	NEWT	LIV	100	NO	HK	HK	T	C	C	C	12/11/11	12/11/11	I	LA	DOLPHIN INTERNATIONAL
2011900789	CNP?	CYNOPS	SPECIES	FIREBELLY	NEWT	LIV	2,240	NO	VS	HK	T	W	C	C	12/11/11	12/11/11	I	LA	AQUA-NAUTIC SPECIALIST
2011900803	TRIH	TRITURUS	HONGKONGENSIS	ALPINE	NEWT	LIV	110	NO	SG	SG	T	C	C	C	12/11/11	12/11/11	I	LA	PET-SIAM INTERNATIONAL
2011900955	CYPY	CYNOPS	PYRRHOGASTER	JAPANESE FIREBELLY	NEWT	LIV	315	NO	HK	HK	T	C	C	C	12/12/11	12/11/11	I	NY	Transship Discounts Ltd.
2011900955	PACB	PACHYTRITON	BREVIPES	TSITOU	NEWT	LIV	90	NO	HK	HK	T	C	C	C	12/12/11	12/11/11	I	NY	Transship Discounts Ltd.
2011902300	CYOR	CYNOPS	ORIENTALIS	CHINESE DWARF	NEWT	LIV	500	NO	HK	HK	T	C	C	C	12/13/11	12/14/11	I	TP	5-D TROPICAL INC.
2011904351	NOVI	NOTOPHTHALMUS	VIRIDESCENS	EASTERN	NEWT	LIV	3	NO	US	CA	T	W	C	C	12/16/11	12/14/11	I	BN	Science Kit, Inc - Boreal
2011905161	CYOR	CYNOPS	ORIENTALIS	CHINESE DWARF	NEWT	LIV	375	NO	HK	HK	T	C	C	C	12/18/11	12/18/11	I	LA	DOLPHIN INTERNATIONAL
2011905180	TRIH	TRITURUS	HONGKONGENSIS	ALPINE	NEWT	LIV	25	NO	SG	SG	T	C	C	C	12/18/11	12/18/11	I	LA	PET-SIAM INTERNATIONAL
2011905191	TRIH	TRITURUS	HONGKONGENSIS	ALPINE	NEWT	LIV	200	NO	HK	HK	T	C	C	C	12/18/11	12/18/11	I	LA	AQUACO, INC.
2011906086	CYPY	CYNOPS	PYRRHOGASTER	JAPANESE FIREBELLY	NEWT	LIV	210	NO	HK	HK	T	C	C	C	12/20/11	12/18/11	I	NY	Transship Discounts Ltd.
2011908065	NOVI	NOTOPHTHALMUS	VIRIDESCENS	EASTERN	NEWT	LIV	2	NO	US	CA	T	W	C	C	12/22/11	12/21/11	I	BN	Science Kit, Inc - Boreal
2011908856	CYOR	CYNOPS	ORIENTALIS	CHINESE DWARF	NEWT	LIV	175	NO	HK	HK	T	C	C	C	12/25/11	12/25/11	I	LA	DOLPHIN INTERNATIONAL
2011909731	CYPY	CYNOPS	PYRRHOGASTER	JAPANESE FIREBELLY	NEWT	LIV	10	NO	HK	HK	T	C	C	C	12/28/11	12/23/11	I	NY	Transship Discounts Ltd.
2011909981	TRIH	TRITURUS	HONGKONGENSIS	ALPINE	NEWT	LIV	1,600	NO	HK	HK	T	C	C	C	12/28/11	12/29/11	I	TP	5-D TROPICAL INC.
2012100042	CYOR	CYNOPS	ORIENTALIS	CHINESE DWARF	NEWT	LIV	1,150	NO	HK	HK	T	C	C	C	6/17/12	6/17/12	I	LA	DOLPHIN INTERNATIONAL
2012100056	TRIH	TRITURUS	HONGKONGENSIS	ALPINE	NEWT	LIV	75	NO	SG	SG	T	C	C	C	6/17/12	6/17/12	I	LA	PET-SIAM INTERNATIONAL
2012100080	CNP?	CYNOPS	SPECIES	FIREBELLY	NEWT	LIV	140	NO	CN	HK	T	W	C	C	6/17/12	6/17/12	I	LA	AQUA-NAUTIC SPECIALIST
2012100080	PLAB	PACHYTRITON	LABIATUS	UNTERSTEIN'S	NEWT	LIV	200	NO	CN	HK	T	W	C	C	6/17/12	6/17/12	I	LA	AQUA-NAUTIC SPECIALIST
2012101205	CYPY	CYNOPS	PYRRHOGASTER	JAPANESE FIREBELLY	NEWT	LIV	525	NO	HK	HK	T	C	C	C	6/18/12	6/17/12	I	NY	Transship Discounts Ltd.
2012101546	TRIH	TRITURUS	HONGKONGENSIS	ALPINE	NEWT	LIV	1,600	NO	HK	HK	T	C	C	C	6/19/12	6/20/12	I	TP	5-D TROPICAL INC.

2012101546	CYOR	CYNOPS	ORIENTALIS	CHINESE DWARF	NEWT	LIV	500	NO	HK	HK	T	C	C	C	6/19/12	6/20/12	I	TP	5-D TROPICAL INC.
2012103968	TYKW	TYLOTOTRITON	KWEICHOWENSIS	KWEICHOW CROCODILE	NEWT	LIV	313	NO	CN	HK	T	W	C	C	6/21/12	6/20/12	I	SF	WONDERFUL WORLD OF PET SUPPLIES INC
2012103968	TYL?	TYLOTOTRITON	SPECIES	CROCODILE	NEWT	LIV	118	NO	CN	HK	T	W	C	C	6/21/12	6/20/12	I	SF	WONDERFUL WORLD OF PET SUPPLIES INC
2012103968	TYTA	TYLOTOTRITON	TALIANGENSIS	PUSAKANG CROCODILE	NEWT	LIV	210	NO	CN	HK	T	W	C	C	6/21/12	6/20/12	I	SF	WONDERFUL WORLD OF PET SUPPLIES INC
2012103968	TYSH	TYLOTOTRITON	SHANJING	MANDARIN	NEWT	LIV	130	NO	CN	HK	T	W	C	C	6/21/12	6/20/12	I	SF	WONDERFUL WORLD OF PET SUPPLIES INC
2012105052	CYOR	CYNOPS	ORIENTALIS	CHINESE DWARF	NEWT	LIV	250	NO	HK	HK	T	C	C	C	6/24/12	6/24/12	I	LA	DOLPHIN INTERNATIONAL
2012105083	TRIH	TRITURUS	HONGKONGENSIS	ALPINE	NEWT	LIV	265	NO	SG	SG	T	C	C	C	6/24/12	6/24/12	I	LA	PET-SIAM INTERNATIONAL
2012105094	CNP?	CYNOPS	SPECIES	FIREBELLY	NEWT	LIV	1,640	NO	CN	HK	T	W	C	C	6/24/12	6/24/12	I	LA	AQUA-NAUTIC SPECIALIST
2012106265	TRIH	TRITURUS	HONGKONGENSIS	ALPINE	NEWT	LIV	1,600	NO	HK	HK	T	C	C	C	6/26/12	6/27/12	I	TP	5-D TROPICAL INC.
2012106265	CYOR	CYNOPS	ORIENTALIS	CHINESE DWARF	NEWT	LIV	366	NO	HK	HK	T	C	C	C	6/26/12	6/27/12	I	TP	5-D TROPICAL INC.
2012106281	CYPY	CYNOPS	PYRRHOGASTER	JAPANESE FIREBELLY	NEWT	LIV	105	NO	HK	HK	T	C	C	C	6/26/12	6/24/12	I	NY	Transship Discounts Ltd.
2012109397	CYOR	CYNOPS	ORIENTALIS	CHINESE DWARF	NEWT	LIV	250	NO	HK	HK	T	C	C	C	7/1/12	7/1/12	I	LA	DOLPHIN INTERNATIONAL
2012109397	PACB	PACHYTRITON	BREVIPES	TSITOU	NEWT	LIV	100	NO	HK	HK	T	C	C	C	7/1/12	7/1/12	I	LA	DOLPHIN INTERNATIONAL
2012109399	TRIH	TRITURUS	HONGKONGENSIS	ALPINE	NEWT	LIV	200	NO	HK	HK	T	C	C	C	7/1/12	7/1/12	I	LA	AQUACO, INC.
2012109460	CNP?	CYNOPS	SPECIES	FIREBELLY	NEWT	LIV	580	NO	CN	HK	T	W	C	C	7/1/12	7/1/12	I	LA	AQUA-NAUTIC SPECIALIST
2012110198	CYPY	CYNOPS	PYRRHOGASTER	JAPANESE FIREBELLY	NEWT	LIV	525	NO	HK	HK	T	C	C	C	7/3/12	7/1/12	I	NY	Transship Discounts Ltd.
2012112361	CNP?	CYNOPS	SPECIES	FIREBELLY	NEWT	LIV	500	NO	HK	HK	T	C	C	C	7/7/12	7/7/12	I	AT	SUN PET LTD.
2012112465	TRIH	TRITURUS	HONGKONGENSIS	ALPINE	NEWT	LIV	36	NO	HK	HK	T	C	C	C	7/8/12	7/8/12	I	LA	AQUACO, INC.
2012112469	TRIH	TRITURUS	HONGKONGENSIS	ALPINE	NEWT	LIV	75	NO	SG	SG	T	C	C	C	7/8/12	7/8/12	I	LA	PET-SIAM INTERNATIONAL
2012112477	CYOR	CYNOPS	ORIENTALIS	CHINESE DWARF	NEWT	LIV	925	NO	HK	HK	T	C	C	C	7/8/12	7/8/12	I	LA	DOLPHIN INTERNATIONAL
2012112494	CNP?	CYNOPS	SPECIES	FIREBELLY	NEWT	LIV	2,680	NO	CN	HK	T	W	C	C	7/8/12	7/8/12	I	LA	AQUA-NAUTIC SPECIALIST
2012113042	CYPY	CYNOPS	PYRRHOGASTER	JAPANESE FIREBELLY	NEWT	LIV	270	NO	HK	HK	T	C	C	C	7/9/12	7/8/12	I	NY	Transship Discounts Ltd.
2012113042	PACB	PACHYTRITON	BREVIPES	TSITOU	NEWT	LIV	30	NO	HK	HK	T	C	C	C	7/9/12	7/8/12	I	NY	Transship Discounts Ltd.
2012113412	TRIH	TRITURUS	HONGKONGENSIS	ALPINE	NEWT	LIV	1,600	NO	HK	HK	T	C	C	C	7/10/12	7/11/12	I	TP	5-D TROPICAL INC.
2012113412	CYOR	CYNOPS	ORIENTALIS	CHINESE DWARF	NEWT	LIV	500	NO	HK	HK	T	C	C	C	7/10/12	7/11/12	I	TP	5-D TROPICAL INC.
2012116871	CYOR	CYNOPS	ORIENTALIS	CHINESE DWARF	NEWT	LIV	500	NO	HK	HK	T	C	C	C	7/15/12	7/15/12	I	LA	DOLPHIN INTERNATIONAL
2012116871	PACB	PACHYTRITON	BREVIPES	TSITOU	NEWT	LIV	150	NO	HK	HK	T	C	C	C	7/15/12	7/15/12	I	LA	DOLPHIN INTERNATIONAL
2012116888	CNP?	CYNOPS	SPECIES	FIREBELLY	NEWT	LIV	140	NO	CN	HK	T	W	C	C	7/15/12	7/15/12	I	LA	AQUA-NAUTIC SPECIALIST
2012116888	TRIT	TRITURUS	SPECIES		NEWT	LIV	45	NO	CN	HK	T	W	C	C	7/15/12	7/15/12	I	LA	AQUA-NAUTIC SPECIALIST
2012117922	TRIH	TRITURUS	HONGKONGENSIS	ALPINE	NEWT	LIV	1,600	NO	HK	HK	T	C	C	C	7/17/12	7/18/12	I	TP	5-D TROPICAL INC.
2012117922	CYOR	CYNOPS	ORIENTALIS	CHINESE DWARF	NEWT	LIV	500	NO	HK	HK	T	C	C	C	7/17/12	7/18/12	I	TP	5-D TROPICAL INC.
2012117951	CYOR	CYNOPS	ORIENTALIS	CHINESE DWARF	NEWT	LIV	890	NO	HK	HK	T	C	C	C	7/17/12	7/18/12	I	TP	5-D TROPICAL, INC.
2012120716	CYPY	CYNOPS	PYRRHOGASTER	JAPANESE FIREBELLY	NEWT	LIV	505	NO	HK	HK	T	C	C	C	7/21/12	7/15/12	I	NY	Transship Discounts Ltd.
2012120716	PACB	PACHYTRITON	BREVIPES	TSITOU	NEWT	LIV	30	NO	HK	HK	T	C	C	C	7/21/12	7/15/12	I	NY	Transship Discounts Ltd.
2012120885	CYOR	CYNOPS	ORIENTALIS	CHINESE DWARF	NEWT	LIV	125	NO	HK	HK	T	C	C	C	7/22/12	7/22/12	I	LA	DOLPHIN INTERNATIONAL
2012120898	TRIH	TRITURUS	HONGKONGENSIS	ALPINE	NEWT	LIV	115	NO	SG	SG	T	C	C	C	7/22/12	7/22/12	I	LA	PET-SIAM INTERNATIONAL
2012120939	CNP?	CYNOPS	SPECIES	FIREBELLY	NEWT	LIV	1,400	NO	CN	HK	T	W	C	C	7/22/12	7/22/12	I	LA	AQUA-NAUTIC SPECIALIST
2012120939	TRIT	TRITURUS	SPECIES		NEWT	LIV	100	NO	CN	HK	T	W	C	C	7/22/12	7/22/12	I	LA	AQUA-NAUTIC SPECIALIST
2012120994	CYPY	CYNOPS	PYRRHOGASTER	JAPANESE FIREBELLY	NEWT	LIV	105	NO	HK	HK	T	C	C	C	7/23/12	7/22/12	I	NY	Transship Discounts Ltd.
2012122486	TRIH	TRITURUS	HONGKONGENSIS	ALPINE	NEWT	LIV	1,600	NO	HK	HK	T	C	C	C	7/25/12	7/25/12	I	TP	5-D TROPICAL INC.
2012124436	CYPY	CYNOPS	PYRRHOGASTER	JAPANESE FIREBELLY	NEWT	LIV	210	NO	HK	HK	T	C	C	C	7/30/12	7/29/12	I	NY	Transship Discounts Ltd.
2012124610	TRIH	TRITURUS	HONGKONGENSIS	ALPINE	NEWT	LIV	185	NO	SG	SG	T	C	C	C	7/29/12	7/29/12	I	LA	PET-SIAM INTERNATIONAL
2012124611	CYOR	CYNOPS	ORIENTALIS	CHINESE DWARF	NEWT	LIV	1,000	NO	HK	HK	T	C	C	C	7/29/12	7/29/12	I	LA	DOLPHIN INTERNATIONAL
2012124619	CNP?	CYNOPS	SPECIES	FIREBELLY	NEWT	LIV	580	NO	CN	HK	T	W	C	C	7/30/12	7/29/12	I	LA	AQUA-NAUTIC SPECIALIST
2012125256	TRIH	TRITURUS	HONGKONGENSIS	ALPINE	NEWT	LIV	1,600	NO	HK	HK	T	C	C	C	7/31/12	8/1/12	I	TP	5-D TROPICAL INC.
2012125256	CYOR	CYNOPS	ORIENTALIS	CHINESE DWARF	NEWT	LIV	500	NO	HK	HK	T	C	C	C	7/31/12	8/1/12	I	TP	5-D TROPICAL INC.
2012128424	CYOR	CYNOPS	ORIENTALIS	CHINESE DWARF	NEWT	LIV	250	NO	HK	HK	T	C	C	C	8/5/12	8/5/12	I	LA	DOLPHIN INTERNATIONAL
2012128424	PACB	PACHYTRITON	BREVIPES	TSITOU	NEWT	LIV	50	NO	HK	HK	T	C	C	C	8/5/12	8/5/12	I	LA	DOLPHIN INTERNATIONAL
2012128466	CNP?	CYNOPS	SPECIES	FIREBELLY	NEWT	LIV	1,040	NO	CN	HK	T	W	C	C	8/5/12	8/5/12	I	LA	AQUA-NAUTIC SPECIALIST
2012128466	TRIT	TRITURUS	SPECIES		NEWT	LIV	45	NO	CN	HK	T	W	C	C	8/5/12	8/5/12	I	LA	AQUA-NAUTIC SPECIALIST
2012128471	TRIH	TRITURUS	HONGKONGENSIS	ALPINE	NEWT	LIV	25	NO	SG	SG	T	C	C	C	8/5/12	8/5/12	I	LA	PET-SIAM INTERNATIONAL
2012129501	TRIH	TRITURUS	HONGKONGENSIS	ALPINE	NEWT	LIV	1,600	NO	HK	HK	T	C	C	C	8/7/12	8/8/12	I	TP	5-D TROPICAL INC.
2012129501	CYOR	CYNOPS	ORIENTALIS	CHINESE DWARF	NEWT	LIV	500	NO	HK	HK	T	C	C	C	8/7/12	8/8/12	I	TP	5-D TROPICAL INC.
2012132398	CYOR	CYNOPS	ORIENTALIS	CHINESE DWARF	NEWT	LIV	250	NO	HK	HK	T	C	C	C	8/12/12	8/12/12	I	LA	DOLPHIN INTERNATIONAL
2012132398	PACB	PACHYTRITON	BREVIPES	TSITOU	NEWT	LIV	100	NO	HK	HK	T	C	C	C	8/12/12	8/12/12	I	LA	DOLPHIN INTERNATIONAL
2012132441	CNP?	CYNOPS	SPECIES	FIREBELLY	NEWT	LIV	880	NO	CN	HK	T	W	C	C	8/12/12	8/12/12	I	LA	AQUA-NAUTIC SPECIALIST

2012132759	CYPY	CYNOPS	PYRRHOGASTER	JAPANESE FIREBELLY	NEWT	LIV	660	NO	HK	HK	T	C	C	C	8/13/12	8/5/12	I	NY	Transship Discounts Ltd.
2012132970	PACB	PACHYTRITON	BREVIPES	TSITOU	NEWT	LIV	30	NO	HK	HK	T	C	C	C	8/13/12	8/12/12	I	NY	Transship Discounts Ltd.
2012133854	TRIH	TRITURUS	HONGKONGENSIS	ALPINE	NEWT	LIV	1,600	NO	HK	HK	T	C	C	C	8/14/12	8/15/12	I	TP	5-D TROPICAL INC.
2012133854	CYOR	CYNOPS	ORIENTALIS	CHINESE DWARF	NEWT	LIV	460	NO	HK	HK	T	C	C	C	8/14/12	8/15/12	I	TP	5-D TROPICAL INC.
2012134258	SAL?	SALAMANDRA	SPECIES	FIRE	SALAMANDER	LIV	3	NO	FR	FR	T	C	C	C	8/14/12	8/14/12	I	MI	TWO AMIGOS IMPORTS, INC.
2012134258	NEUC	NEURERGUS	CROCATUS	LAKE URMIA	NEWT	LIV	13	NO	FR	FR	T	C	C	C	8/14/12	8/14/12	I	MI	TWO AMIGOS IMPORTS, INC.
2012134520	NOVI	NOTOPHTHALMUS	VIRIDESCENS	EASTERN	NEWT	LIV	1	NO	US	CA	T	W	C	C	8/15/12	8/14/12	I	BN	Science Kit, Inc - Boreal
2012134779	CNP?	CYNOPS	SPECIES	FIREBELLY	NEWT	LIV	370	NO	HK	HK	T	C	C	C	8/16/12	8/16/12	I	AT	SUN PET LTD.
2012136018	CYOR	CYNOPS	ORIENTALIS	CHINESE DWARF	NEWT	LIV	500	NO	HK	HK	T	C	C	C	8/19/12	8/19/12	I	LA	DOLPHIN INTERNATIONAL
2012136018	PACB	PACHYTRITON	BREVIPES	TSITOU	NEWT	LIV	100	NO	HK	HK	T	C	C	C	8/19/12	8/19/12	I	LA	DOLPHIN INTERNATIONAL
2012136068	CNP?	CYNOPS	SPECIES	FIREBELLY	NEWT	LIV	1,160	NO	CN	HK	T	W	C	C	8/19/12	8/19/12	I	LA	AQUA-NAUTIC SPECIALIST
2012136833	TRIH	TRITURUS	HONGKONGENSIS	ALPINE	NEWT	LIV	1,600	NO	HK	HK	T	C	C	C	8/21/12	8/22/12	I	TP	5-D TROPICAL INC.
2012136838	PACB	PACHYTRITON	BREVIPES	TSITOU	NEWT	LIV	210	NO	HK	HK	T	C	C	C	8/21/12	8/19/12	I	NY	Transship Discounts Ltd.
2012136838	CYPY	CYNOPS	PYRRHOGASTER	JAPANESE FIREBELLY	NEWT	LIV	315	NO	HK	HK	T	C	C	C	8/21/12	8/19/12	I	NY	Transship Discounts Ltd.
2012138958	CYOR	CYNOPS	ORIENTALIS	CHINESE DWARF	NEWT	LIV	50	NO	HK	HK	T	C	C	C	8/26/12	8/26/12	I	LA	DOLPHIN INTERNATIONAL
2012138989	CNP?	CYNOPS	SPECIES	FIREBELLY	NEWT	LIV	720	NO	CN	HK	T	W	C	C	8/26/12	8/26/12	I	LA	AQUA-NAUTIC SPECIALIST
2012139474	CYPY	CYNOPS	PYRRHOGASTER	JAPANESE FIREBELLY	NEWT	LIV	210	NO	HK	HK	T	C	C	C	8/27/12	8/26/12	I	NY	Transship Discounts Ltd.
2012139830	TRIH	TRITURUS	HONGKONGENSIS	ALPINE	NEWT	LIV	1,600	NO	HK	HK	T	C	C	C	8/28/12	8/29/12	I	TP	5-D TROPICAL INC.
2012139830	CYOR	CYNOPS	ORIENTALIS	CHINESE DWARF	NEWT	LIV	500	NO	HK	HK	T	C	C	C	8/28/12	8/29/12	I	TP	5-D TROPICAL INC.
2012141462	TYL?	TYLOTOTRITON	SPECIES	CROCODILE	NEWT	LIV	6	NO	DE	DE	T	C	C	C	8/31/12	8/30/12	I	MI	TWO AMIGOS IMPORTS, INC.
2012141462	TYSH	TYLOTOTRITON	SHANJING	MANDARIN	NEWT	LIV	6	NO	DE	DE	T	C	C	C	8/31/12	8/30/12	I	MI	TWO AMIGOS IMPORTS, INC.
2012141462	TYTA	TYLOTOTRITON	TALIANGENSIS	PUSAKANG CROCODILE	NEWT	LIV	6	NO	DE	DE	T	C	C	C	8/31/12	8/30/12	I	MI	TWO AMIGOS IMPORTS, INC.
2012141462	TYKW	TYLOTOTRITON	KWEICHOWENSIS	KWEICHOW CROCODILE	NEWT	LIV	6	NO	DE	DE	T	C	C	C	8/31/12	8/30/12	I	MI	TWO AMIGOS IMPORTS, INC.
2012142003	CYOR	CYNOPS	ORIENTALIS	CHINESE DWARF	NEWT	LIV	875	NO	HK	HK	T	C	C	C	9/2/12	9/2/12	I	LA	DOLPHIN INTERNATIONAL
2012142003	PACB	PACHYTRITON	BREVIPES	TSITOU	NEWT	LIV	100	NO	HK	HK	T	C	C	C	9/2/12	9/2/12	I	LA	DOLPHIN INTERNATIONAL
2012142016	TRIH	TRITURUS	HONGKONGENSIS	ALPINE	NEWT	LIV	50	NO	SG	SG	T	C	C	C	9/2/12	9/2/12	I	LA	AQUACO, INC.
2012142017	TRIH	TRITURUS	HONGKONGENSIS	ALPINE	NEWT	LIV	75	NO	SG	SG	T	C	C	C	9/2/12	9/2/12	I	LA	PET-SIAM INTERNATIONAL
2012142042	CNP?	CYNOPS	SPECIES	FIREBELLY	NEWT	LIV	140	NO	CN	HK	T	W	C	C	9/2/12	9/2/12	I	LA	AQUA-NAUTIC SPECIALIST
2012142579	TRIH	TRITURUS	HONGKONGENSIS	ALPINE	NEWT	LIV	1,600	NO	HK	HK	T	C	C	C	9/4/12	9/5/12	I	TP	5-D TROPICAL INC.
2012145285	NOVI	NOTOPHTHALMUS	VIRIDESCENS	EASTERN	NEWT	LIV	1	NO	US	CA	T	W	C	C	9/7/12	9/6/12	I	BN	Science Kit, Inc - Boreal
2012146034	CYOR	CYNOPS	ORIENTALIS	CHINESE DWARF	NEWT	LIV	550	NO	HK	HK	T	C	C	C	9/9/12	9/9/12	I	LA	DOLPHIN INTERNATIONAL
2012146071	CNP?	CYNOPS	SPECIES	FIREBELLY	NEWT	LIV	560	NO	CN	HK	T	W	C	C	9/9/12	9/9/12	I	LA	AQUA-NAUTIC SPECIALIST
2012146071	TRIT	TRITURUS	SPECIES		NEWT	LIV	45	NO	CN	HK	T	W	C	C	9/9/12	9/9/12	I	LA	AQUA-NAUTIC SPECIALIST
2012146505	CYPY	CYNOPS	PYRRHOGASTER	JAPANESE FIREBELLY	NEWT	LIV	105	NO	HK	HK	T	C	C	C	9/10/12	9/9/12	I	NY	Transship Discounts Ltd.
2012146505	PACB	PACHYTRITON	BREVIPES	TSITOU	NEWT	LIV	90	NO	HK	HK	T	C	C	C	9/10/12	9/9/12	I	NY	Transship Discounts Ltd.
2012146978	TRIH	TRITURUS	HONGKONGENSIS	ALPINE	NEWT	LIV	1,600	NO	HK	HK	T	C	C	C	9/11/12	9/12/12	I	TP	5-D TROPICAL INC.
2012146978	CYOR	CYNOPS	ORIENTALIS	CHINESE DWARF	NEWT	LIV	500	NO	HK	HK	T	C	C	C	9/11/12	9/12/12	I	TP	5-D TROPICAL INC.
2012148529	NOVI	NOTOPHTHALMUS	VIRIDESCENS	EASTERN	NEWT	LIV	6	NO	US	CA	T	W	C	C	9/13/12	9/13/12	I	BN	Science Kit, Inc - Boreal
2012149446	CYOR	CYNOPS	ORIENTALIS	CHINESE DWARF	NEWT	LIV	375	NO	HK	HK	T	C	C	C	9/16/12	9/16/12	I	LA	DOLPHIN INTERNATIONAL
2012149482	CNP?	CYNOPS	SPECIES	FIREBELLY	NEWT	LIV	880	NO	CN	HK	T	W	C	C	9/16/12	9/16/12	I	LA	AQUA-NAUTIC SPECIALIST
2012149482	TRIT	TRITURUS	SPECIES		NEWT	LIV	45	NO	CN	HK	T	W	C	C	9/16/12	9/16/12	I	LA	AQUA-NAUTIC SPECIALIST
2012149934	CYPY	CYNOPS	PYRRHOGASTER	JAPANESE FIREBELLY	NEWT	LIV	345	NO	HK	HK	T	C	C	C	9/17/12	9/16/12	I	NY	Transship Discounts Ltd.
2012149934	PACB	PACHYTRITON	BREVIPES	TSITOU	NEWT	LIV	30	NO	HK	HK	T	C	C	C	9/17/12	9/16/12	I	NY	Transship Discounts Ltd.
2012150417	TRIH	TRITURUS	HONGKONGENSIS	ALPINE	NEWT	LIV	1,600	NO	HK	HK	T	C	C	C	9/18/12	9/19/12	I	TP	5-D TROPICAL INC.
2012150417	CYOR	CYNOPS	ORIENTALIS	CHINESE DWARF	NEWT	LIV	500	NO	HK	HK	T	C	C	C	9/18/12	9/19/12	I	TP	5-D TROPICAL INC.
2012150621	NOVI	NOTOPHTHALMUS	VIRIDESCENS	EASTERN	NEWT	LIV	4	NO	US	CA	T	W	C	C	9/18/12	9/18/12	I	BN	Science Kit, Inc - Boreal
2012151607	CNP?	CYNOPS	SPECIES	FIREBELLY	NEWT	LIV	400	NO	HK	HK	T	C	C	C	9/20/12	9/20/12	I	AT	SUN PET LTD.
2012153072	CNP?	CYNOPS	SPECIES	FIREBELLY	NEWT	LIV	440	NO	CN	HK	T	W	C	C	9/23/12	9/23/12	I	LA	AQUA-NAUTIC SPECIALIST
2012153072	TRIT	TRITURUS	SPECIES		NEWT	LIV	45	NO	CN	HK	T	W	C	C	9/23/12	9/23/12	I	LA	AQUA-NAUTIC SPECIALIST
2012153744	CYOR	CYNOPS	ORIENTALIS	CHINESE DWARF	NEWT	LIV	250	NO	HK	HK	T	C	C	C	9/24/12	9/25/12	I	LA	DOLPHIN INTERNATIONAL
2012153744	PACB	PACHYTRITON	BREVIPES	TSITOU	NEWT	LIV	200	NO	HK	HK	T	C	C	C	9/24/12	9/25/12	I	LA	DOLPHIN INTERNATIONAL
2012154422	CYOR	CYNOPS	ORIENTALIS	CHINESE DWARF	NEWT	LIV	540	NO	HK	HK	T	C	C	C	9/25/12	9/26/12	I	TP	5-D TROPICAL, INC.
2012154588	NOVI	NOTOPHTHALMUS	VIRIDESCENS	EASTERN	NEWT	LIV	5	NO	US	CA	T	W	C	C	9/25/12	9/25/12	I	BN	Science Kit, Inc - Boreal
2012154781	CYPY	CYNOPS	PYRRHOGASTER	JAPANESE FIREBELLY	NEWT	LIV	470	NO	HK	HK	T	C	C	C	9/25/12	9/23/12	I	NY	Transship Discounts Ltd.
2012157038	CYOR	CYNOPS	ORIENTALIS	CHINESE DWARF	NEWT	LIV	800	NO	HK	HK	T	C	C	C	9/30/12	9/30/12	I	LA	DOLPHIN INTERNATIONAL
2012157038	PACB	PACHYTRITON	BREVIPES	TSITOU	NEWT	LIV	50	NO	HK	HK	T	C	C	C	9/30/12	9/30/12	I	LA	DOLPHIN INTERNATIONAL
2012157076	TRIT	TRITURUS	SPECIES		NEWT	LIV	90	NO	CN	HK	T	W	C	C	9/30/12	9/30/12	I	LA	AQUA-NAUTIC SPECIALIST
2012157076	CNP?	CYNOPS	SPECIES	FIREBELLY	NEWT	LIV	580	NO	CN	HK	T	W	C	C	9/30/12	9/30/12	I	LA	AQUA-NAUTIC SPECIALIST
2012157086	TRIH	TRITURUS	HONGKONGENSIS	ALPINE	NEWT	LIV	100	NO	SG	SG	T	C	C	C	9/30/12	9/30/12	I	LA	PET-SIAM INTERNATIONAL
2012157767	PACB	PACHYTRITON	BREVIPES	TSITOU	NEWT	LIV	90	NO	HK	HK	T	C	C	C	10/1/12	9/30/12	I	NY	Transship Discounts Ltd.

2012159047	CYPY	CYNOPS	PYRRHOGASTER	JAPANESE FIREBELLY	NEWT	LIV	10	NO	JP	JP	T	C	C	C	10/3/12	10/3/12	I	LA	HERP TRADING INC
2012159400	NOVI	NOTOPHTHALMUS	VIRIDESCENS	EASTERN	NEWT	LIV	1	NO	US	CA	T	W	C	C	10/4/12	10/3/12	I	BN	Science Kit, Inc - Boreal
2012160646	CYOR	CYNOPS	ORIENTALIS	CHINESE DWARF	NEWT	LIV	250	NO	HK	HK	T	C	C	C	10/7/12	10/7/12	I	LA	DOLPHIN INTERNATIONAL
2012160646	PACB	PACHYTRITON	BREVIPES	TSITOU	NEWT	LIV	100	NO	HK	HK	T	C	C	C	10/7/12	10/7/12	I	LA	DOLPHIN INTERNATIONAL
2012160687	PLAB	PACHYTRITON	LABIATUS	UNTERSTEIN'S	NEWT	LIV	34	NO	CN	HK	T	W	C	C	10/7/12	10/7/12	I	LA	AQUA-NAUTIC SPECIALIST
2012160687	CNP?	CYNOPS	SPECIES	FIREBELLY	NEWT	LIV	1,470	NO	CN	HK	T	W	C	C	10/7/12	10/7/12	I	LA	AQUA-NAUTIC SPECIALIST
2012161153	TRIH	TRITURUS	HONGKONGENSIS	ALPINE	NEWT	LIV	1,600	NO	HK	HK	T	C	C	C	10/9/12	10/10/12	I	TP	5-D TROPICAL INC.
2012161153	CYOR	CYNOPS	ORIENTALIS	CHINESE DWARF	NEWT	LIV	500	NO	HK	HK	T	C	C	C	10/9/12	10/10/12	I	TP	5-D TROPICAL INC.
2012161532	NOVI	NOTOPHTHALMUS	VIRIDESCENS	EASTERN	NEWT	LIV	2	NO	US	CA	T	W	C	C	10/9/12	10/9/12	I	BN	Science Kit, Inc - Boreal
2012162495	CYPY	CYNOPS	PYRRHOGASTER	JAPANESE FIREBELLY	NEWT	LIV	105	NO	HK	HK	T	C	C	C	10/10/12	10/7/12	I	NY	Transship Discounts Ltd.
2012163226	TYKW	TYLOTOTRITON	KWEICHOWENSIS	KWEICHOW CROCODILE	NEWT	LIV	6	NO	DE	DE	T	C	C	C	10/11/12	10/11/12	I	MI	TWO AMIGOS IMPORTS, INC.
2012164300	CYOR	CYNOPS	ORIENTALIS	CHINESE DWARF	NEWT	LIV	675	NO	HK	HK	T	C	C	C	10/14/12	10/14/12	I	LA	DOLPHIN INTERNATIONAL
2012164300	PACB	PACHYTRITON	BREVIPES	TSITOU	NEWT	LIV	100	NO	HK	HK	T	C	C	C	10/14/12	10/14/12	I	LA	DOLPHIN INTERNATIONAL
2012164310	TRIH	TRITURUS	HONGKONGENSIS	ALPINE	NEWT	LIV	35	NO	SG	SG	T	C	C	C	10/14/12	10/14/12	I	LA	PET-SIAM INTERNATIONAL
2012164326	CNP?	CYNOPS	SPECIES	FIREBELLY	NEWT	LIV	440	NO	VS	HK	T	W	C	C	10/14/12	10/14/12	I	LA	AQUA-NAUTIC SPECIALIST
2012164326	TRIT	TRITURUS	SPECIES		NEWT	LIV	45	NO	VS	HK	T	W	C	C	10/14/12	10/14/12	I	LA	AQUA-NAUTIC SPECIALIST
2012165200	TRIH	TRITURUS	HONGKONGENSIS	ALPINE	NEWT	LIV	1,600	NO	HK	HK	T	C	C	C	10/16/12	10/17/12	I	TP	5-D TROPICAL INC.
2012165200	CYOR	CYNOPS	ORIENTALIS	CHINESE DWARF	NEWT	LIV	500	NO	HK	HK	T	C	C	C	10/16/12	10/17/12	I	TP	5-D TROPICAL INC.
2012165463	NOVI	NOTOPHTHALMUS	VIRIDESCENS	EASTERN	NEWT	LIV	7	NO	US	CA	T	W	C	C	10/16/12	10/16/12	I	BN	Science Kit, Inc - Boreal
2012168007	CYOR	CYNOPS	ORIENTALIS	CHINESE DWARF	NEWT	LIV	375	NO	HK	HK	T	C	C	C	10/21/12	10/21/12	I	LA	DOLPHIN INTERNATIONAL
2012168007	PACB	PACHYTRITON	BREVIPES	TSITOU	NEWT	LIV	50	NO	HK	HK	T	C	C	C	10/21/12	10/21/12	I	LA	DOLPHIN INTERNATIONAL
2012168072	TRIH	TRITURUS	HONGKONGENSIS	ALPINE	NEWT	LIV	35	NO	SG	SG	T	C	C	C	10/21/12	10/21/12	I	LA	PET-SIAM INTERNATIONAL
2012168075	CNP?	CYNOPS	SPECIES	FIREBELLY	NEWT	LIV	280	NO	CN	HK	T	W	C	C	10/21/12	10/21/12	I	LA	AQUA-NAUTIC SPECIALIST
2012168870	CYPY	CYNOPS	PYRRHOGASTER	JAPANESE FIREBELLY	NEWT	LIV	210	NO	HK	HK	T	C	C	C	10/22/12	10/21/12	I	NY	Transship Discounts Ltd.
2012170220	TRIH	TRITURUS	HONGKONGENSIS	ALPINE	NEWT	LIV	1,600	NO	HK	HK	T	C	C	C	10/23/12	10/24/12	I	TP	5-D TROPICAL INC.
2012170220	CYOR	CYNOPS	ORIENTALIS	CHINESE DWARF	NEWT	LIV	500	NO	HK	HK	T	C	C	C	10/23/12	10/24/12	I	TP	5-D TROPICAL INC.
2012173649	CYOR	CYNOPS	ORIENTALIS	CHINESE DWARF	NEWT	LIV	1,000	NO	HK	HK	T	C	C	C	10/28/12	10/28/12	I	LA	DOLPHIN INTERNATIONAL
2012173684	CNP?	CYNOPS	SPECIES	FIREBELLY	NEWT	LIV	2,460	NO	VS	HK	T	W	C	C	10/28/12	10/28/12	I	LA	AQUA-NAUTIC SPECIALIST
2012174352	TRIH	TRITURUS	HONGKONGENSIS	ALPINE	NEWT	LIV	1,600	NO	HK	HK	T	C	C	C	10/30/12	10/31/12	I	TP	5-D TROPICAL INC.
2012174352	CYOR	CYNOPS	ORIENTALIS	CHINESE DWARF	NEWT	LIV	500	NO	HK	HK	T	C	C	C	10/30/12	10/31/12	I	TP	5-D TROPICAL INC.
2012175153	SASA	SALAMANDRA	SALAMANDRA	EUROPEAN FIRE	SALAMANDER	LIV	600	NO	SI	SI	T	C	C	C	11/1/12	10/31/12	I	MI	NAME REMOVED
2012176221	CYOR	CYNOPS	ORIENTALIS	CHINESE DWARF	NEWT	LIV	375	NO	HK	HK	T	C	C	C	11/4/12	11/4/12	I	LA	DOLPHIN INTERNATIONAL
2012179012	PACB	PACHYTRITON	BREVIPES	TSITOU	NEWT	LIV	60	NO	HK	HK	T	C	C	C	11/7/12	10/28/12	I	NY	Transship Discounts Ltd.
2012179445	NOVI	NOTOPHTHALMUS	VIRIDESCENS	EASTERN	NEWT	LIV	1	NO	US	CA	T	W	C	C	11/7/12	11/6/12	I	BN	Science Kit, Inc - Boreal
2012180017	NOVI	NOTOPHTHALMUS	VIRIDESCENS	EASTERN	NEWT	LIV	4	NO	US	CA	T	W	C	C	8/31/12	8/28/12	I	BN	Science Kit, Inc - Boreal
2012181087	CYOR	CYNOPS	ORIENTALIS	CHINESE DWARF	NEWT	LIV	425	NO	HK	HK	T	C	C	C	11/11/12	11/11/12	I	LA	DOLPHIN INTERNATIONAL
2012181087	PACB	PACHYTRITON	BREVIPES	TSITOU	NEWT	LIV	100	NO	HK	HK	T	C	C	C	11/11/12	11/11/12	I	LA	DOLPHIN INTERNATIONAL
2012181118	TRIH	TRITURUS	HONGKONGENSIS	ALPINE	NEWT	LIV	175	NO	SG	SG	T	C	C	C	11/11/12	11/11/12	I	LA	PET-SIAM INTERNATIONAL
2012181126	CNP?	CYNOPS	SPECIES	FIREBELLY	NEWT	LIV	720	NO	CN	HK	T	W	C	C	11/11/12	11/11/12	I	LA	AQUA-NAUTIC SPECIALIST
2012181126	PLAB	PACHYTRITON	LABIATUS	UNTERSTEIN'S	NEWT	LIV	100	NO	CN	HK	T	W	C	C	11/11/12	11/11/12	I	LA	AQUA-NAUTIC SPECIALIST
2012182295	CYOR	CYNOPS	ORIENTALIS	CHINESE DWARF	NEWT	LIV	1,400	NO	HK	HK	T	C	C	C	11/13/12	11/14/12	I	TP	5-D TROPICAL, INC.
2012182584	NOVI	NOTOPHTHALMUS	VIRIDESCENS	EASTERN	NEWT	LIV	2	NO	US	CA	T	W	C	C	11/13/12	11/13/12	I	BN	Science Kit, Inc - Boreal
2012183213	PACB	PACHYTRITON	BREVIPES	TSITOU	NEWT	LIV	75	NO	HK	HK	T	C	C	C	11/14/12	11/11/12	I	NY	Transship Discounts Ltd.
2012183213	CYPY	CYNOPS	PYRRHOGASTER	JAPANESE FIREBELLY	NEWT	LIV	420	NO	HK	HK	T	C	C	C	11/14/12	11/11/12	I	NY	Transship Discounts Ltd.
2012185474	CYOR	CYNOPS	ORIENTALIS	CHINESE DWARF	NEWT	LIV	375	NO	HK	HK	T	C	C	C	11/18/12	11/18/12	I	LA	DOLPHIN INTERNATIONAL
2012185502	CNP?	CYNOPS	SPECIES	FIREBELLY	NEWT	LIV	950	NO	CN	HK	T	W	C	C	11/18/12	11/18/12	I	LA	AQUA-NAUTIC SPECIALIST
2012185613	NMAC	NECTURUS	MACULOSUS		MUDPUPPY	LIV	1	NO	CA	CA	Z	W	C	C	11/19/12	11/19/12	I	DE	DETROIT ZOOLOGICAL SOCIETY
2012186979	CYPY	CYNOPS	PYRRHOGASTER	JAPANESE FIREBELLY	NEWT	LIV	105	NO	HK	HK	T	C	C	C	11/20/12	11/18/12	I	NY	Transship Discounts Ltd.
2012188930	CYOR	CYNOPS	ORIENTALIS	CHINESE DWARF	NEWT	LIV	750	NO	HK	HK	T	C	C	C	11/25/12	11/25/12	I	LA	DOLPHIN INTERNATIONAL
2012188942	TRIH	TRITURUS	HONGKONGENSIS	ALPINE	NEWT	LIV	20	NO	SG	SG	T	C	C	C	11/25/12	11/25/12	I	LA	PET-SIAM INTERNATIONAL
2012188960	CNP?	CYNOPS	SPECIES	FIREBELLY	NEWT	LIV	140	NO	CN	HK	T	W	C	C	11/25/12	11/25/12	I	LA	AQUA-NAUTIC SPECIALIST
2012190106	TRIH	TRITURUS	HONGKONGENSIS	ALPINE	NEWT	LIV	1,600	NO	HK	HK	T	C	C	C	11/27/12	11/28/12	I	TP	5-D TROPICAL INC.
2012190106	CYOR	CYNOPS	ORIENTALIS	CHINESE DWARF	NEWT	LIV	250	NO	HK	HK	T	C	C	C	11/27/12	11/28/12	I	TP	5-D TROPICAL INC.
2012191426	CNP?	CYNOPS	SPECIES	FIREBELLY	NEWT	LIV	210	NO	HK	HK	T	W	C	C	11/28/12	11/25/12	I	NY	Transship Discounts Ltd.
2012191990	NOVI	NOTOPHTHALMUS	VIRIDESCENS	EASTERN	NEWT	LIV	2	NO	US	CA	T	W	C	C	11/29/12	11/28/12	I	BN	Science Kit, Inc - Boreal
2012193163	CYOR	CYNOPS	ORIENTALIS	CHINESE DWARF	NEWT	LIV	550	NO	HK	HK	T	C	C	C	12/2/12	12/2/12	I	LA	DOLPHIN INTERNATIONAL
2012193163	PACB	PACHYTRITON	BREVIPES	TSITOU	NEWT	LIV	150	NO	HK	HK	T	C	C	C	12/2/12	12/2/12	I	LA	DOLPHIN INTERNATIONAL
2012193173	TRIH	TRITURUS	HONGKONGENSIS	ALPINE	NEWT	LIV	105	NO	SG	SG	T	C	C	C	12/2/12	12/2/12	I	LA	PET-SIAM INTERNATIONAL
2012193183	CNP?	CYNOPS	SPECIES	FIREBELLY	NEWT	LIV	300	NO	CN	HK	T	W	C	C	12/2/12	12/2/12	I	LA	AQUA-NAUTIC SPECIALIST
2012194885	TRIH	TRITURUS	HONGKONGENSIS	ALPINE	NEWT	LIV	1,600	NO	HK	HK	T	C	C	C	12/4/12	12/5/12	I	TP	5-D TROPICAL INC.

2012194885	CYOR	CYNOPS	ORIENTALIS	CHINESE DWARF	NEWT	LIV	500	NO	HK	HK	T	C	C	C	12/4/12	12/5/12	I	TP	5-D TROPICAL INC.
2012195491	NOVI	NOTOPHTHALMUS	VIRIDESCENS	EASTERN	NEWT	LIV	5	NO	US	CA	T	W	C	C	12/5/12	12/4/12	I	BN	Science Kit, Inc - Boreal
2012195847	CYPY	CYNOPS	PYRRHOGASTER	JAPANESE FIREBELLY	NEWT	LIV	25	NO	DE	DE	T	C	C	C	12/6/12	12/5/12	I	MI	TWO AMIGOS IMPORTS, INC.
2012195847	CNP?	CYNOPS	SPECIES	FIREBELLY	NEWT	LIV	25	NO	DE	DE	T	C	C	C	12/6/12	12/5/12	I	MI	TWO AMIGOS IMPORTS, INC.
2012195847	TYKW	TYLOTOTRITON	KWEICHOWENSIS	KWEICHOW CROCODILE	NEWT	LIV	25	NO	DE	DE	T	C	C	C	12/6/12	12/5/12	I	MI	TWO AMIGOS IMPORTS, INC.
2012195847	TYL?	TYLOTOTRITON	SPECIES	CROCODILE	NEWT	LIV	10	NO	DE	DE	T	C	C	C	12/6/12	12/5/12	I	MI	TWO AMIGOS IMPORTS, INC.
2012197187	CYOR	CYNOPS	ORIENTALIS	CHINESE DWARF	NEWT	LIV	1,125	NO	HK	HK	T	C	C	C	12/9/12	12/9/12	I	LA	DOLPHIN INTERNATIONAL
2012197187	PACB	PACHYTRITON	BREVIPES	TSITOU	NEWT	LIV	200	NO	HK	HK	T	C	C	C	12/9/12	12/9/12	I	LA	DOLPHIN INTERNATIONAL
2012197240	CNP?	CYNOPS	SPECIES	FIREBELLY	NEWT	LIV	1,330	NO	CI	HK	T	W	C	C	12/9/12	12/9/12	I	LA	AQUA-NAUTIC SPECIALIST
2012197240	PLAB	PACHYTRITON	LABIATUS	UNTERSTEIN'S	NEWT	LIV	45	NO	CI	HK	T	W	C	C	12/9/12	12/9/12	I	LA	AQUA-NAUTIC SPECIALIST
2012197458	CYPY	CYNOPS	PYRRHOGASTER	JAPANESE FIREBELLY	NEWT	LIV	210	NO	HK	HK	T	C	C	C	12/10/12	12/9/12	I	NY	Transship Discounts Ltd.
2012197458	PACB	PACHYTRITON	BREVIPES	TSITOU	NEWT	LIV	30	NO	HK	HK	T	C	C	C	12/10/12	12/9/12	I	NY	Transship Discounts Ltd.
2012197853	CNP?	CYNOPS	SPECIES	FIREBELLY	NEWT	LIV	880	NO	VS	HK	T	W	C	C	12/10/12	11/4/12	I	LA	AQUA-NAUTIC SPECIALIST
2012197853	PLAB	PACHYTRITON	LABIATUS	UNTERSTEIN'S	NEWT	LIV	45	NO	VS	HK	T	W	C	C	12/10/12	11/4/12	I	LA	AQUA-NAUTIC SPECIALIST
2012198586	CYPY	CYNOPS	PYRRHOGASTER	JAPANESE FIREBELLY	NEWT	LIV	130	NO	HK	HK	T	C	C	C	12/11/12	12/2/12	I	NY	Transship Discounts Ltd.
2012198586	PACB	PACHYTRITON	BREVIPES	TSITOU	NEWT	LIV	90	NO	HK	HK	T	C	C	C	12/11/12	12/2/12	I	NY	Transship Discounts Ltd.
2012199586	NOVI	NOTOPHTHALMUS	VIRIDESCENS	EASTERN	NEWT	LIV	1	NO	US	CA	T	W	C	C	12/13/12	12/12/12	I	BN	Science Kit, Inc - Boreal
2012201153	CYOR	CYNOPS	ORIENTALIS	CHINESE DWARF	NEWT	LIV	500	NO	HK	HK	T	C	C	C	12/16/12	12/16/12	I	LA	DOLPHIN INTERNATIONAL
2012201821	PACB	PACHYTRITON	BREVIPES	TSITOU	NEWT	LIV	60	NO	HK	HK	T	C	C	C	12/17/12	12/16/12	I	NY	Transship Discounts Ltd.
2012202567	CYOR	CYNOPS	ORIENTALIS	CHINESE DWARF	NEWT	LIV	500	NO	HK	HK	T	C	C	C	12/18/12	12/19/12	I	TP	5-D TROPICAL INC.
2012205235	CNP?	CYNOPS	SPECIES	FIREBELLY	NEWT	LIV	600	NO	CN	HK	T	W	C	C	12/22/12	12/22/12	I	LA	AQUA-NAUTIC SPECIALIST
2012205718	CYPY	CYNOPS	PYRRHOGASTER	JAPANESE FIREBELLY	NEWT	LIV	3,000	NO	JP	JP	T	C	C	C	12/26/12	12/26/12	I	LA	HERP TRADING INC.
2012207153	TYKW	TYLOTOTRITON	KWEICHOWENSIS	KWEICHOW CROCODILE	NEWT	LIV	25	NO	DE	DE	T	C	C	C	12/28/12	12/21/12	I	MI	TWO AMIGOS IMPORTS, INC.
2012207663	CNP?	CYNOPS	SPECIES	FIREBELLY	NEWT	LIV	440	NO	CN	HK	T	W	C	C	12/29/12	12/29/12	I	LA	AQUA-NAUTIC SPECIALIST
2012207701	CYOR	CYNOPS	ORIENTALIS	CHINESE DWARF	NEWT	LIV	40	NO	SG	SG	T	C	C	C	12/30/12	12/30/12	I	LA	PET-SIAM INTERNATIONAL
2012911197	CYOR	CYNOPS	ORIENTALIS	CHINESE DWARF	NEWT	LIV	1,125	NO	HK	HK	T	C	C	C	1/1/12	1/1/12	I	LA	DOLPHIN INTERNATIONAL
2012911197	PACB	PACHYTRITON	BREVIPES	TSITOU	NEWT	LIV	150	NO	HK	HK	T	C	C	C	1/1/12	1/1/12	I	LA	DOLPHIN INTERNATIONAL
2012911203	CNP?	CYNOPS	SPECIES	FIREBELLY	NEWT	LIV	2,220	NO	CN	HK	T	W	C	C	1/1/12	1/1/12	I	LA	AQUA-NAUTIC SPECIALIST
2012912454	NOVI	NOTOPHTHALMUS	VIRIDESCENS	EASTERN	NEWT	LIV	1	NO	US	CA	T	W	C	C	1/4/12	1/4/12	I	BN	Science Kit, Inc - Boreal
2012912479	TRIH	TRITURUS	HONGKONGENSIS	ALPINE	NEWT	LIV	1,600	NO	HK	HK	T	C	C	C	1/4/12	1/5/12	I	TP	5-D TROPICAL INC.
2012912479	CYOR	CYNOPS	ORIENTALIS	CHINESE DWARF	NEWT	LIV	500	NO	HK	HK	T	C	C	C	1/4/12	1/5/12	I	TP	5-D TROPICAL INC.
2012913267	CYPY	CYNOPS	PYRRHOGASTER	JAPANESE FIREBELLY	NEWT	LIV	315	NO	HK	HK	T	C	C	C	1/5/12	12/30/11	I	NY	Transship Discounts Ltd.
2012913267	PACB	PACHYTRITON	BREVIPES	TSITOU	NEWT	LIV	120	NO	HK	HK	T	C	C	C	1/5/12	12/30/11	I	NY	Transship Discounts Ltd.
2012914396	TRIH	TRITURUS	HONGKONGENSIS	ALPINE	NEWT	LIV	25	NO	SG	SG	T	C	C	C	1/7/12	1/8/12	I	LA	PET-SIAM INTERNATIONAL
2012914448	CYOR	CYNOPS	ORIENTALIS	CHINESE DWARF	NEWT	LIV	250	NO	HK	HK	T	C	C	C	1/8/12	1/8/12	I	LA	DOLPHIN INTERNATIONAL
2012914448	PACB	PACHYTRITON	BREVIPES	TSITOU	NEWT	LIV	150	NO	HK	HK	T	C	C	C	1/8/12	1/8/12	I	LA	DOLPHIN INTERNATIONAL
2012914464	CNP?	CYNOPS	SPECIES	FIREBELLY	NEWT	LIV	880	NO	CN	HK	T	W	C	C	1/8/12	1/8/12	I	LA	AQUA-NAUTIC SPECIALIST
2012914464	TRIT	TRITURUS	SPECIES		NEWT	LIV	45	NO	CN	HK	T	W	C	C	1/8/12	1/8/12	I	LA	AQUA-NAUTIC SPECIALIST
2012914464	PLAB	PACHYTRITON	LABIATUS	UNTERSTEIN'S	NEWT	LIV	90	NO	CN	HK	T	W	C	C	1/8/12	1/8/12	I	LA	AQUA-NAUTIC SPECIALIST
2012915572	CYOR	CYNOPS	ORIENTALIS	CHINESE DWARF	NEWT	LIV	500	NO	HK	HK	T	C	C	C	1/10/12	1/11/12	I	TP	5-D TROPICAL INC.
2012915954	CYPY	CYNOPS	PYRRHOGASTER	JAPANESE FIREBELLY	NEWT	LIV	315	NO	HK	HK	T	C	C	C	1/10/12	1/8/12	I	NY	Transship Discounts Ltd.
2012919082	CYOR	CYNOPS	ORIENTALIS	CHINESE DWARF	NEWT	LIV	850	NO	HK	HK	T	C	C	C	1/15/12	1/15/12	I	LA	DOLPHIN INTERNATIONAL
2012919082	PACB	PACHYTRITON	BREVIPES	TSITOU	NEWT	LIV	150	NO	HK	HK	T	C	C	C	1/15/12	1/15/12	I	LA	DOLPHIN INTERNATIONAL
2012919118	PLAB	PACHYTRITON	LABIATUS	UNTERSTEIN'S	NEWT	LIV	200	NO	CN	HK	T	W	C	C	1/15/12	1/15/12	I	LA	AQUA-NAUTIC SPECIALIST
2012919118	TRIT	TRITURUS	SPECIES		NEWT	LIV	90	NO	CN	HK	T	W	C	C	1/15/12	1/15/12	I	LA	AQUA-NAUTIC SPECIALIST
2012919118	CNP?	CYNOPS	SPECIES	FIREBELLY	NEWT	LIV	3,900	NO	CN	HK	T	W	C	C	1/15/12	1/15/12	I	LA	AQUA-NAUTIC SPECIALIST
2012920225	TRIH	TRITURUS	HONGKONGENSIS	ALPINE	NEWT	LIV	1,600	NO	HK	HK	T	C	C	C	1/17/12	1/18/12	I	TP	5-D TROPICAL INC.
2012920225	CYOR	CYNOPS	ORIENTALIS	CHINESE DWARF	NEWT	LIV	500	NO	HK	HK	T	C	C	C	1/17/12	1/18/12	I	TP	5-D TROPICAL INC.
2012921415	CYPY	CYNOPS	PYRRHOGASTER	JAPANESE FIREBELLY	NEWT	LIV	180	NO	HK	HK	T	C	C	C	1/18/12	1/15/12	I	NY	Transship Discounts Ltd.
2012921415	PACB	PACHYTRITON	BREVIPES	TSITOU	NEWT	LIV	60	NO	HK	HK	T	C	C	C	1/18/12	1/15/12	I	NY	Transship Discounts Ltd.
2012921583	NOVI	NOTOPHTHALMUS	VIRIDESCENS	EASTERN	NEWT	LIV	2	NO	US	CA	T	W	C	C	1/19/12	1/16/12	I	BN	Science Kit, Inc - Boreal
2012923262	CYOR	CYNOPS	ORIENTALIS	CHINESE DWARF	NEWT	LIV	300	NO	HK	HK	T	C	C	C	1/22/12	1/22/12	I	LA	DOLPHIN INTERNATIONAL
2012923277	TRIH	TRITURUS	HONGKONGENSIS	ALPINE	NEWT	LIV	110	NO	SG	SG	T	C	C	C	1/22/12	1/22/12	I	LA	PET-SIAM INTERNATIONAL
2012923290	CNP?	CYNOPS	SPECIES	FIREBELLY	NEWT	LIV	140	NO	CN	HK	T	W	C	C	1/22/12	1/22/12	I	LA	AQUA-NAUTIC SPECIALIST
2012923290	TRIT	TRITURUS	SPECIES		NEWT	LIV	480	NO	CN	HK	T	W	C	C	1/22/12	1/22/12	I	LA	AQUA-NAUTIC SPECIALIST
2012924303	NOVI	NOTOPHTHALMUS	VIRIDESCENS	EASTERN	NEWT	LIV	4	NO	US	CA	T	W	C	C	1/24/12	1/23/12	I	BN	Science Kit, Inc - Boreal
2012925241	CYPY	CYNOPS	PYRRHOGASTER	JAPANESE FIREBELLY	NEWT	LIV	145	NO	HK	HK	T	C	C	C	1/25/12	1/22/12	I	NY	Transship Discounts Ltd.
2012927804	CYOR	CYNOPS	ORIENTALIS	CHINESE DWARF	NEWT	LIV	125	NO	HK	HK	T	C	C	C	1/29/12	1/29/12	I	LA	DOLPHIN INTERNATIONAL
2012927804	PACB	PACHYTRITON	BREVIPES	TSITOU	NEWT	LIV	100	NO	HK	HK	T	C	C	C	1/29/12	1/29/12	I	LA	DOLPHIN INTERNATIONAL
2012927829	TRIH	TRITURUS	HONGKONGENSIS	ALPINE	NEWT	LIV	110	NO	SG	SG	T	C	C	C	1/29/12	1/29/12	I	LA	PET-SIAM INTERNATIONAL

2012928438	CYPY	CYNOPS	PYRRHOGASTER	JAPANESE FIREBELLY	NEWT	LIV	585	NO	HK	HK	T	C	C	C	1/30/12	1/29/12	I	NY	Transship Discounts Ltd.
2012929504	NOVI	NOTOPHTHALMUS	VIRIDESCENS	EASTERN	NEWT	LIV	1	NO	US	CA	T	W	C	C	2/1/12	1/30/12	I	BN	Science Kit, Inc - Boreal
2012930908	CYOR	CYNOPS	ORIENTALIS	CHINESE DWARF	NEWT	LIV	875	NO	HK	HK	T	C	C	C	2/5/12	2/5/12	I	LA	DOLPHIN INTERNATIONAL
2012930943	TRIT	TRITURUS	SPECIES		NEWT	LIV	90	NO	CN	HK	T	W	C	C	2/5/12	2/5/12	I	LA	AQUA-NAUTIC SPECIALIST
2012930943	CNP?	CYNOPS	SPECIES	FIREBELLY	NEWT	LIV	1,200	NO	CN	HK	T	W	C	C	2/5/12	2/5/12	I	LA	AQUA-NAUTIC SPECIALIST
2012930943	PLAB	PACHYTRITON	LABIATUS	UNTERSTEIN'S	NEWT	LIV	200	NO	CN	HK	T	W	C	C	2/5/12	2/5/12	I	LA	AQUA-NAUTIC SPECIALIST
2012932199	CYOR	CYNOPS	ORIENTALIS	CHINESE DWARF	NEWT	LIV	320	NO	HK	HK	T	C	C	C	2/7/12	2/8/12	I	TP	5-D TROPICAL, INC.
2012933898	NOVI	NOTOPHTHALMUS	VIRIDESCENS	EASTERN	NEWT	LIV	6	NO	US	CA	T	W	C	C	2/9/12	2/8/12	I	BN	Science Kit, Inc - Boreal
2012934953	CYOR	CYNOPS	ORIENTALIS	CHINESE DWARF	NEWT	LIV	900	NO	HK	HK	T	C	C	C	2/12/12	2/12/12	I	LA	DOLPHIN INTERNATIONAL
2012934953	PACB	PACHYTRITON	BREVIPES	TSITOU	NEWT	LIV	150	NO	HK	HK	T	C	C	C	2/12/12	2/12/12	I	LA	DOLPHIN INTERNATIONAL
2012934961	CNP?	CYNOPS	SPECIES	FIREBELLY	NEWT	LIV	560	NO	CN	HK	T	W	C	C	2/12/12	2/12/12	I	LA	AQUA-NAUTIC SPECIALIST
2012935289	CYPY	CYNOPS	PYRRHOGASTER	JAPANESE FIREBELLY	NEWT	LIV	300	NO	HK	HK	T	C	C	C	2/13/12	2/12/12	I	NY	Transship Discounts Ltd.
2012936142	TRIH	TRITURUS	HONGKONGENSIS	ALPINE	NEWT	LIV	3,200	NO	HK	HK	T	C	C	C	2/14/12	2/15/12	I	TP	5-D TROPICAL INC.
2012936144	CYOR	CYNOPS	ORIENTALIS	CHINESE DWARF	NEWT	LIV	200	NO	HK	HK	T	C	C	C	2/14/12	2/15/12	I	TP	5-D TROPICAL, INC.
2012938480	TRIT	TRITURUS	SPECIES		NEWT	LIV	90	NO	CN	HK	T	W	C	C	2/19/12	2/19/12	I	LA	AQUA-NAUTIC SPECIALIST
2012938521	CYOR	CYNOPS	ORIENTALIS	CHINESE DWARF	NEWT	LIV	625	NO	HK	HK	T	C	C	C	2/19/12	2/19/12	I	LA	DOLPHIN INTERNATIONAL
2012938521	PACB	PACHYTRITON	BREVIPES	TSITOU	NEWT	LIV	100	NO	HK	HK	T	C	C	C	2/19/12	2/19/12	I	LA	DOLPHIN INTERNATIONAL
2012939216	CYOR	CYNOPS	ORIENTALIS	CHINESE DWARF	NEWT	LIV	300	NO	HK	HK	T	C	C	C	2/21/12	2/22/12	I	TP	5-D TROPICAL, INC.
2012939710	CYPY	CYNOPS	PYRRHOGASTER	JAPANESE FIREBELLY	NEWT	LIV	180	NO	HK	HK	T	C	C	C	2/22/12	2/19/12	I	NY	Transship Discounts Ltd.
2012941822	CYOR	CYNOPS	ORIENTALIS	CHINESE DWARF	NEWT	LIV	650	NO	HK	HK	T	C	C	C	2/25/12	2/26/12	I	LA	DOLPHIN INTERNATIONAL
2012943139	TRIH	TRITURUS	HONGKONGENSIS	ALPINE	NEWT	LIV	3,200	NO	HK	HK	T	C	C	C	2/28/12	2/29/12	I	TP	5-D TROPICAL INC.
2012943143	CYOR	CYNOPS	ORIENTALIS	CHINESE DWARF	NEWT	LIV	1,400	NO	HK	HK	T	C	C	C	2/28/12	2/28/12	I	TP	5-D TROPICAL, INC.
2012943201	CYPY	CYNOPS	PYRRHOGASTER	JAPANESE FIREBELLY	NEWT	LIV	295	NO	HK	HK	T	C	C	C	2/28/12	2/26/12	I	NY	Transship Discounts Ltd.
2012943201	PACB	PACHYTRITON	BREVIPES	TSITOU	NEWT	LIV	30	NO	HK	HK	T	W	C	C	2/28/12	2/26/12	I	NY	Transship Discounts Ltd.
2012943865	NEW?	PACHYTRITON	SPECIES	CHINESE	NEWT	LIV	18	NO	SG	SG	T	C	C	C	2/29/12	2/25/12	I	NY	EMARK TROPICAL IMPORTS, INC.
2012944069	CYOR	CYNOPS	ORIENTALIS	CHINESE DWARF	NEWT	LIV	100	NO	SG	SG	T	C	C	C	3/1/12	2/1/12	I	HN	GOLDEN VIVA, INC.
2012945333	CYOR	CYNOPS	ORIENTALIS	CHINESE DWARF	NEWT	LIV	500	NO	HK	HK	T	C	C	C	3/4/12	3/4/12	I	LA	DOLPHIN INTERNATIONAL
2012945333	PACB	PACHYTRITON	BREVIPES	TSITOU	NEWT	LIV	100	NO	HK	HK	T	C	C	C	3/4/12	3/4/12	I	LA	DOLPHIN INTERNATIONAL
2012945364	CNP?	CYNOPS	SPECIES	FIREBELLY	NEWT	LIV	440	NO	CN	HK	T	W	C	C	3/4/12	3/4/12	I	LA	AQUA-NAUTIC SPECIALIST
2012945377	TRIH	TRITURUS	HONGKONGENSIS	ALPINE	NEWT	LIV	35	NO	SG	SG	T	C	C	C	3/4/12	3/4/12	I	LA	PET-SIAM INTERNATIONAL
2012948593	CYPY	CYNOPS	PYRRHOGASTER	JAPANESE FIREBELLY	NEWT	LIV	375	NO	HK	HK	T	C	C	C	3/9/12	3/4/12	I	NY	Transship Discounts Ltd.
2012948593	PACB	PACHYTRITON	BREVIPES	TSITOU	NEWT	LIV	120	NO	HK	HK	T	W	C	C	3/9/12	3/4/12	I	NY	Transship Discounts Ltd.
2012948844	CNP?	CYNOPS	SPECIES	FIREBELLY	NEWT	LIV	200	NO	CN	CN	T	C	C	C	3/10/12	3/10/12	I	AT	SUN PET LTD.
2012948955	CYOR	CYNOPS	ORIENTALIS	CHINESE DWARF	NEWT	LIV	500	NO	HK	HK	T	C	C	C	3/11/12	3/11/12	I	LA	DOLPHIN INTERNATIONAL
2012948955	PACB	PACHYTRITON	BREVIPES	TSITOU	NEWT	LIV	50	NO	HK	HK	T	C	C	C	3/11/12	3/11/12	I	LA	DOLPHIN INTERNATIONAL
2012948982	TRIH	TRITURUS	HONGKONGENSIS	ALPINE	NEWT	LIV	110	NO	SG	SG	T	C	C	C	3/11/12	3/11/12	I	LA	PET-SIAM INTERNATIONAL
2012949291	CYPY	CYNOPS	PYRRHOGASTER	JAPANESE FIREBELLY	NEWT	LIV	270	NO	HK	HK	T	C	C	C	3/12/12	3/11/12	I	NY	Transship Discounts Ltd.
2012950093	TRIH	TRITURUS	HONGKONGENSIS	ALPINE	NEWT	LIV	1,600	NO	HK	HK	T	C	C	C	3/13/12	3/14/12	I	TP	5-D TROPICAL INC.
2012950094	CYOR	CYNOPS	ORIENTALIS	CHINESE DWARF	NEWT	LIV	1,100	NO	HK	HK	T	C	C	C	3/13/12	3/14/12	I	TP	5-D TROPICAL, INC.
2012952107	NEUC	NEURERGUS	CROCATUS	LAKE URMIA	NEWT	LIV	170	NO	IQ	DE	T	C	C	C	3/16/12	3/16/12	I	CH	NAME REMOVED
2012952107	NRR?	NEURERGUS	SPECIES		NEWT	LIV	10	NO	TR	DE	T	C	C	C	3/16/12	3/16/12	I	CH	NAME REMOVED
2012952107	PDEL	PARAMESOTRITON	DELOUSTALI	VIETNAM WARTY	NEWT	LIV	8	NO	VN	DE	T	C	C	C	3/16/12	3/16/12	I	CH	NAME REMOVED
2012952337	CNP?	CYNOPS	SPECIES	FIREBELLY	NEWT	LIV	200	NO	CN	CN	T	C	C	C	3/17/12	3/17/12	I	AT	SUN PET LTD.
2012952388	CYOR	CYNOPS	ORIENTALIS	CHINESE DWARF	NEWT	LIV	400	NO	HK	HK	T	C	C	C	3/18/12	3/18/12	I	LA	DOLPHIN INTERNATIONAL
2012952388	PACB	PACHYTRITON	BREVIPES	TSITOU	NEWT	LIV	100	NO	HK	HK	T	C	C	C	3/18/12	3/18/12	I	LA	DOLPHIN INTERNATIONAL
2012952445	TRIH	TRITURUS	HONGKONGENSIS	ALPINE	NEWT	LIV	110	NO	SG	SG	T	C	C	C	3/18/12	3/18/12	I	LA	PET-SIAM INTERNATIONAL
2012956033	CYOR	CYNOPS	ORIENTALIS	CHINESE DWARF	NEWT	LIV	750	NO	HK	HK	T	C	C	C	3/25/12	3/25/12	I	LA	DOLPHIN INTERNATIONAL
2012956033	PACB	PACHYTRITON	BREVIPES	TSITOU	NEWT	LIV	100	NO	HK	HK	T	C	C	C	3/25/12	3/25/12	I	LA	DOLPHIN INTERNATIONAL
2012956042	TRIH	TRITURUS	HONGKONGENSIS	ALPINE	NEWT	LIV	96	NO	HK	HK	T	C	C	C	3/25/12	3/25/12	I	LA	AQUACO, INC.
2012956060	CYOR	CYNOPS	ORIENTALIS	CHINESE DWARF	NEWT	LIV	280	NO	CN	HK	T	W	C	C	3/25/12	3/25/12	I	LA	AQUA-NAUTIC SPECIALIST
2012956060	TRIT	TRITURUS	SPECIES		NEWT	LIV	90	NO	CN	HK	T	W	C	C	3/25/12	3/25/12	I	LA	AQUA-NAUTIC SPECIALIST
2012957489	CYOR	CYNOPS	ORIENTALIS	CHINESE DWARF	NEWT	LIV	500	NO	HK	HK	T	C	C	C	3/27/12	3/28/12	I	TP	5-D TROPICAL INC.
2012957496	CYOR	CYNOPS	ORIENTALIS	CHINESE DWARF	NEWT	LIV	320	NO	HK	HK	T	C	C	C	3/27/12	3/28/12	I	TP	5-D TROPICAL, INC.
2012959450	CYPY	CYNOPS	PYRRHOGASTER	JAPANESE FIREBELLY	NEWT	LIV	270	NO	HK	HK	T	C	C	C	3/30/12	3/25/12	I	NY	Transship Discounts Ltd.
2012959450	PACB	PACHYTRITON	BREVIPES	TSITOU	NEWT	LIV	60	NO	HK	HK	T	C	C	C	3/30/12	3/25/12	I	NY	Transship Discounts Ltd.
2012959673	CNP?	CYNOPS	SPECIES	FIREBELLY	NEWT	LIV	200	NO	CN	CN	T	C	C	C	3/31/12	3/31/12	I	AT	SUN PET LTD.
2012959729	CYOR	CYNOPS	ORIENTALIS	CHINESE DWARF	NEWT	LIV	475	NO	HK	HK	T	C	C	C	4/1/12	4/1/12	I	LA	DOLPHIN INTERNATIONAL
2012959729	PACB	PACHYTRITON	BREVIPES	TSITOU	NEWT	LIV	100	NO	HK	HK	T	C	C	C	4/1/12	4/1/12	I	LA	DOLPHIN INTERNATIONAL
2012959738	TRIH	TRITURUS	HONGKONGENSIS	ALPINE	NEWT	LIV	25	NO	SG	SG	T	C	C	C	4/1/12	4/1/12	I	LA	PET-SIAM INTERNATIONAL
2012959750	CYPY	CYNOPS	PYRRHOGASTER	JAPANESE FIREBELLY	NEWT	LIV	75	NO	SG	SG	T	C	C	C	4/1/12	4/1/12	I	LA	TROPICAL FISH CONSOLIDATORS

2012959754	TRIT	TRITURUS	SPECIES		NEWT	LIV	200	NO	HK	HK	T	W	C	C	4/1/12	4/1/12	I	LA	AQUA-NAUTIC SPECIALIST
2012960810	CYPY	CYNOPS	PYRRHOGASTER	JAPANESE FIREBELLY	NEWT	LIV	210	NO	HK	HK	T	C	C	C	4/3/12	4/1/12	I	NY	Transship Discounts Ltd.
2012960810	PACB	PACHYTRITON	BREVIPES	TSITOU	NEWT	LIV	30	NO	HK	HK	T	C	C	C	4/3/12	4/1/12	I	NY	Transship Discounts Ltd.
2012960973	CYOR	CYNOPS	ORIENTALIS	CHINESE DWARF	NEWT	LIV	390	NO	HK	HK	T	C	C	C	4/3/12	4/4/12	I	TP	5-D TROPICAL, INC.
2012963207	CYOR	CYNOPS	ORIENTALIS	CHINESE DWARF	NEWT	LIV	50	NO	HK	HK	T	C	C	C	4/8/12	4/8/12	I	LA	DOLPHIN INTERNATIONAL
2012963214	TRIH	TRITURUS	HONGKONGENSIS	ALPINE	NEWT	LIV	32	NO	SG	SG	T	C	C	C	4/8/12	4/8/12	I	LA	PET-SIAM INTERNATIONAL
2012963235	TRIT	TRITURUS	SPECIES		NEWT	LIV	89	NO	HK	HK	T	W	C	C	4/8/12	4/8/12	I	LA	AQUA-NAUTIC SPECIALIST
2012963374	CYPY	CYNOPS	PYRRHOGASTER	JAPANESE FIREBELLY	NEWT	LIV	130	NO	HK	HK	T	C	C	C	4/9/12	4/8/12	I	NY	Transship Discounts Ltd.
2012964438	TRIH	TRITURUS	HONGKONGENSIS	ALPINE	NEWT	LIV	1,600	NO	HK	HK	T	C	C	C	4/10/12	4/11/12	I	TP	5-D TROPICAL INC.
2012964438	CYOR	CYNOPS	ORIENTALIS	CHINESE DWARF	NEWT	LIV	500	NO	HK	HK	T	C	C	C	4/10/12	4/11/12	I	TP	5-D TROPICAL INC.
2012964448	CYOR	CYNOPS	ORIENTALIS	CHINESE DWARF	NEWT	LIV	1,100	NO	HK	HK	T	C	C	C	4/10/12	4/11/12	I	TP	5-D TROPICAL, INC.
2012966819	CYOR	CYNOPS	ORIENTALIS	CHINESE DWARF	NEWT	LIV	500	NO	HK	HK	T	C	C	C	4/15/12	4/15/12	I	LA	DOLPHIN INTERNATIONAL
2012966819	PACB	PACHYTRITON	BREVIPES	TSITOU	NEWT	LIV	100	NO	HK	HK	T	C	C	C	4/15/12	4/15/12	I	LA	DOLPHIN INTERNATIONAL
2012966831	TRIH	TRITURUS	HONGKONGENSIS	ALPINE	NEWT	LIV	100	NO	HK	HK	T	C	C	C	4/15/12	4/15/12	I	LA	AQUACO, INC.
2012966850	TRIT	TRITURUS	SPECIES		NEWT	LIV	45	NO	HK	HK	T	W	C	C	4/15/12	4/15/12	I	LA	AQUA-NAUTIC SPECIALIST
2012966852	CNP?	CYNOPS	SPECIES	FIREBELLY	NEWT	LIV	200	NO	CN	CN	T	C	C	C	4/15/12	4/15/12	I	AT	SUN PET LTD.
2012966858	TRIH	TRITURUS	HONGKONGENSIS	ALPINE	NEWT	LIV	75	NO	SG	SG	T	C	C	C	4/15/12	4/15/12	I	LA	PET-SIAM INTERNATIONAL
2012967995	CYOR	CYNOPS	ORIENTALIS	CHINESE DWARF	NEWT	LIV	1,740	NO	HK	HK	T	C	C	C	4/17/12	4/18/12	I	TP	5-D TROPICAL, INC.
2012968385	SASA	SALAMANDRA	SALAMANDRA	EUROPEAN FIRE	SALAMANDER	LIV	750	NO	SI	SI	T	F	C	C	4/18/12	4/17/12	I	MI	NAME REMOVED
2012970554	CYOR	CYNOPS	ORIENTALIS	CHINESE DWARF	NEWT	LIV	300	NO	HK	HK	T	C	C	C	4/22/12	4/22/12	I	LA	DOLPHIN INTERNATIONAL
2012970562	TRIT	TRITURUS	SPECIES		NEWT	LIV	90	NO	CN	HK	T	W	C	C	4/22/12	4/22/12	I	LA	AQUA-NAUTIC SPECIALIST
2012970571	TRIH	TRITURUS	HONGKONGENSIS	ALPINE	NEWT	LIV	85	NO	SG	SG	T	C	C	C	4/22/12	4/22/12	I	LA	PET-SIAM INTERNATIONAL
2012972124	TRIH	TRITURUS	HONGKONGENSIS	ALPINE	NEWT	LIV	1,600	NO	HK	HK	T	C	C	C	4/24/12	4/25/12	I	TP	5-D TROPICAL INC.
2012974669	CYOR	CYNOPS	ORIENTALIS	CHINESE DWARF	NEWT	LIV	375	NO	HK	HK	T	C	C	C	4/29/12	4/29/12	I	LA	DOLPHIN INTERNATIONAL
2012974669	PACB	PACHYTRITON	BREVIPES	TSITOU	NEWT	LIV	100	NO	HK	HK	T	C	C	C	4/29/12	4/29/12	I	LA	DOLPHIN INTERNATIONAL
2012974686	TRIH	TRITURUS	HONGKONGENSIS	ALPINE	NEWT	LIV	75	NO	SG	SG	T	C	C	C	4/29/12	4/29/12	I	LA	PET-SIAM INTERNATIONAL
2012974706	TRIT	TRITURUS	SPECIES		NEWT	LIV	45	NO	CN	HK	T	W	C	C	4/29/12	4/29/12	I	LA	AQUA-NAUTIC SPECIALIST
2012975359	CYPY	CYNOPS	PYRRHOGASTER	JAPANESE FIREBELLY	NEWT	LIV	270	NO	HK	HK	T	C	C	C	5/1/12	4/29/12	I	NY	Transship Discounts Ltd.
2012975766	TRIH	TRITURUS	HONGKONGENSIS	ALPINE	NEWT	LIV	1,600	NO	HK	HK	T	C	C	C	5/1/12	5/2/12	I	TP	5-D TROPICAL INC.
2012975766	CYOR	CYNOPS	ORIENTALIS	CHINESE DWARF	NEWT	LIV	500	NO	HK	HK	T	C	C	C	5/1/12	5/2/12	I	TP	5-D TROPICAL INC.
2012976449	NOVI	NOTOPHTHALMUS	VIRIDESCENS	EASTERN	NEWT	LIV	1	NO	US	CA	T	W	C	C	5/2/12	5/2/12	I	BN	Science Kit, Inc - Boreal
2012977928	CYOR	CYNOPS	ORIENTALIS	CHINESE DWARF	NEWT	LIV	550	NO	HK	HK	T	C	C	C	5/6/12	5/6/12	I	LA	DOLPHIN INTERNATIONAL
2012977928	PACB	PACHYTRITON	BREVIPES	TSITOU	NEWT	LIV	150	NO	HK	HK	T	C	C	C	5/6/12	5/6/12	I	LA	DOLPHIN INTERNATIONAL
2012977955	TRIH	TRITURUS	HONGKONGENSIS	ALPINE	NEWT	LIV	75	NO	SG	SG	T	C	C	C	5/6/12	5/6/12	I	LA	PET-SIAM INTERNATIONAL
2012978628	NOVI	NOTOPHTHALMUS	VIRIDESCENS	EASTERN	NEWT	LIV	5	NO	US	CA	T	W	C	C	5/7/12	5/7/12	I	BN	Science Kit, Inc - Boreal
2012979390	TRIH	TRITURUS	HONGKONGENSIS	ALPINE	NEWT	LIV	1,600	NO	HK	HK	T	C	C	C	5/8/12	5/9/12	I	TP	5-D TROPICAL INC.
2012979390	CYOR	CYNOPS	ORIENTALIS	CHINESE DWARF	NEWT	LIV	500	NO	HK	HK	T	C	C	C	5/8/12	5/9/12	I	TP	5-D TROPICAL INC.
2012979899	CYPY	CYNOPS	PYRRHOGASTER	JAPANESE FIREBELLY	NEWT	LIV	315	NO	HK	HK	T	C	C	C	5/9/12	5/6/12	I	NY	Transship Discounts Ltd.
2012980480	SASA	SALAMANDRA	SALAMANDRA	EUROPEAN FIRE	SALAMANDER	LIV	600	NO	SI	SI	T	C	C	C	5/10/12	5/9/12	I	MI	NAME REMOVED
2012981325	SASA	SALAMANDRA	SALAMANDRA	EUROPEAN FIRE	SALAMANDER	LIV	6	NO	RU	CA	T	W	C	C	4/24/12	4/24/12	I	BL	STRICTLY REPTILES INCL
2012981558	NOVI	NOTOPHTHALMUS	VIRIDESCENS	EASTERN	NEWT	LIV	1	NO	US	CA	T	W	C	C	5/11/12	5/9/12	I	BN	Science Kit, Inc - Boreal
2012981824	CNP?	CYNOPS	SPECIES	FIREBELLY	NEWT	LIV	500	NO	HK	HK	T	C	C	C	5/12/12	5/12/12	I	AT	SUN PET LTD.
2012981869	CYOR	CYNOPS	ORIENTALIS	CHINESE DWARF	NEWT	LIV	425	NO	HK	HK	T	C	C	C	5/13/12	5/13/12	I	LA	DOLPHIN INTERNATIONAL
2012981869	PACB	PACHYTRITON	BREVIPES	TSITOU	NEWT	LIV	100	NO	HK	HK	T	C	C	C	5/13/12	5/13/12	I	LA	DOLPHIN INTERNATIONAL
2012981875	TRIH	TRITURUS	HONGKONGENSIS	ALPINE	NEWT	LIV	40	NO	SG	SG	T	C	C	C	5/13/12	5/13/12	I	LA	PET-SIAM INTERNATIONAL
2012981899	PLAB	PACHYTRITON	LABIATUS	UNTERSTEIN'S	NEWT	LIV	300	NO	CN	HK	T	W	C	C	5/13/12	5/13/12	I	LA	AQUA-NAUTIC SPECIALIST
2012982063	CYPY	CYNOPS	PYRRHOGASTER	JAPANESE FIREBELLY	NEWT	LIV	210	NO	HK	HK	T	C	C	C	5/14/12	5/13/12	I	NY	Transship Discounts Ltd.
2012982239	TRIT	TRITURUS	SPECIES		NEWT	LIV	12	NO	DE	DE	T	C	C	C	5/14/12	5/11/12	I	MI	TWO AMIGOS IMPORT & EXPORT, INC.
2012982416	NOVI	NOTOPHTHALMUS	VIRIDESCENS	EASTERN	NEWT	LIV	1	NO	US	CA	T	W	C	C	5/14/12	5/14/12	I	BN	Science Kit, Inc - Boreal
2012982664	TRIH	TRITURUS	HONGKONGENSIS	ALPINE	NEWT	LIV	1,600	NO	HK	HK	T	C	C	C	5/15/12	5/16/12	I	TP	5-D TROPICAL INC.
2012982664	CYOR	CYNOPS	ORIENTALIS	CHINESE DWARF	NEWT	LIV	500	NO	HK	HK	T	C	C	C	5/15/12	5/16/12	I	TP	5-D TROPICAL INC.
2012984323	PLWA	PLEURODELES	WALTL	IBERIAN RIBBED	NEWT	LIV	37	NO	CZ	CZ	T	C	C	C	5/17/12	5/17/12	I	MI	REPTILE INDUSTRIES, INC.
2012985290	CYOR	CYNOPS	ORIENTALIS	CHINESE DWARF	NEWT	LIV	675	NO	HK	HK	T	C	C	C	5/20/12	5/20/12	I	LA	DOLPHIN INTERNATIONAL
2012985290	PACB	PACHYTRITON	BREVIPES	TSITOU	NEWT	LIV	200	NO	HK	HK	T	C	C	C	5/20/12	5/20/12	I	LA	DOLPHIN INTERNATIONAL
2012985295	TRIH	TRITURUS	HONGKONGENSIS	ALPINE	NEWT	LIV	200	NO	HK	HK	T	C	C	C	5/20/12	5/20/12	I	LA	AQUACO, INC.
2012986605	TRIH	TRITURUS	HONGKONGENSIS	ALPINE	NEWT	LIV	1,600	NO	HK	HK	T	C	C	C	5/22/12	5/23/12	I	TP	5-D TROPICAL INC.
2012986605	CYOR	CYNOPS	ORIENTALIS	CHINESE DWARF	NEWT	LIV	500	NO	HK	HK	T	C	C	C	5/22/12	5/23/12	I	TP	5-D TROPICAL INC.
2012988099	PCHI	PARAMESOTRITON	CHINENSIS	CHINESE WARTY	NEWT	LIV	96	NO	HK	HK	T	C	C	C	5/24/12	5/24/12	I	DF	BUSHMASTER REPTILES INC.
2012988099	TYKW	TYLOTOTRITON	KWEICHOWENSIS	KWEICHOW CROCODILE	NEWT	LIV	48	NO	HK	HK	T	C	C	C	5/24/12	5/24/12	I	DF	BUSHMASTER REPTILES INC.
2012989371	CYOR	CYNOPS	ORIENTALIS	CHINESE DWARF	NEWT	LIV	425	NO	HK	HK	T	C	C	C	5/27/12	5/27/12	I	LA	DOLPHIN INTERNATIONAL

2012989371	PACB	PACHYTRITON	BREVIPES	TSITOU	NEWT	LIV	100	NO	HK	HK	T	C	C	C	5/27/12	5/27/12	I	LA	DOLPHIN INTERNATIONAL
2012989383	TRIH	TRITURUS	HONGKONGENSIS	ALPINE	NEWT	LIV	50	NO	HK	HK	T	C	C	C	5/27/12	5/27/12	I	LA	AQUACO, INC.
2012989394	TRIH	TRITURUS	HONGKONGENSIS	ALPINE	NEWT	LIV	75	NO	SG	SG	T	C	C	C	5/27/12	5/27/12	I	LA	PET-SIAM INTERNATIONAL
2012989398	TRIT	TRITURUS	SPECIES		NEWT	LIV	144	NO	CN	HK	T	W	C	C	5/27/12	5/27/12	I	LA	AQUA-NAUTIC SPECIALIST
2012989398	PLAB	PACHYTRITON	LABIATUS	UNTERSTEIN'S	NEWT	LIV	245	NO	CN	HK	T	W	C	C	5/27/12	5/27/12	I	LA	AQUA-NAUTIC SPECIALIST
2012991024	CYPY	CYNOPS	PYRRHOGASTER	JAPANESE FIREBELLY	NEWT	LIV	60	NO	HK	HK	T	C	C	C	5/31/12	5/20/12	I	NY	Transship Discounts Ltd.
2012991024	PACB	PACHYTRITON	BREVIPES	TSITOU	NEWT	LIV	30	NO	HK	HK	T	W	C	C	5/31/12	5/20/12	I	NY	Transship Discounts Ltd.
2012992441	CYOR	CYNOPS	ORIENTALIS	CHINESE DWARF	NEWT	LIV	625	NO	HK	HK	T	C	C	C	6/3/12	6/3/12	I	LA	DOLPHIN INTERNATIONAL
2012992451	TRIH	TRITURUS	HONGKONGENSIS	ALPINE	NEWT	LIV	50	NO	HK	HK	T	C	C	C	6/3/12	6/3/12	I	LA	AQUACO, INC.
2012992489	CNP?	CYNOPS	SPECIES	FIREBELLY	NEWT	LIV	1,020	NO	CN	HK	T	W	C	C	6/3/12	6/3/12	I	LA	AQUA-NAUTIC SPECIALIST
2012992489	TRIT	TRITURUS	SPECIES		NEWT	LIV	100	NO	CN	HK	T	W	C	C	6/3/12	6/3/12	I	LA	AQUA-NAUTIC SPECIALIST
2012993373	TRIH	TRITURUS	HONGKONGENSIS	ALPINE	NEWT	LIV	1,600	NO	HK	HK	T	C	C	C	6/5/12	6/6/12	I	TP	5-D TROPICAL INC.
2012993373	CYOR	CYNOPS	ORIENTALIS	CHINESE DWARF	NEWT	LIV	500	NO	HK	HK	T	C	C	C	6/5/12	6/6/12	I	TP	5-D TROPICAL INC.
2012993476	CYPY	CYNOPS	PYRRHOGASTER	JAPANESE FIREBELLY	NEWT	LIV	105	NO	HK	HK	T	C	C	C	6/5/12	5/27/12	I	NY	Transship Discounts Ltd.
2012994403	SASA	SALAMANDRA	SALAMANDRA	EUROPEAN FIRE	SALAMANDER	LIV	900	NO	SI	SI	T	C	C	C	6/6/12	6/5/12	I	MI	TWO AMIGOS IMPORTS, INC.
2012996425	CYOR	CYNOPS	ORIENTALIS	CHINESE DWARF	NEWT	LIV	300	NO	HK	HK	T	C	C	C	6/10/12	6/10/12	I	LA	DOLPHIN INTERNATIONAL
2012996425	PACB	PACHYTRITON	BREVIPES	TSITOU	NEWT	LIV	100	NO	HK	HK	T	C	C	C	6/10/12	6/10/12	I	LA	DOLPHIN INTERNATIONAL
2012996456	TRIH	TRITURUS	HONGKONGENSIS	ALPINE	NEWT	LIV	40	NO	SG	SG	T	C	C	C	6/10/12	6/10/12	I	LA	PET-SIAM INTERNATIONAL
2012996463	CNP?	CYNOPS	SPECIES	FIREBELLY	NEWT	LIV	870	NO	CN	HK	T	W	C	C	6/10/12	6/10/12	I	LA	AQUA-NAUTIC SPECIALIST
2012996463	TRIT	TRITURUS	SPECIES		NEWT	LIV	45	NO	CN	HK	T	W	C	C	6/10/12	6/10/12	I	LA	AQUA-NAUTIC SPECIALIST
2012996463	PLAB	PACHYTRITON	LABIATUS	UNTERSTEIN'S	NEWT	LIV	45	NO	CN	HK	T	W	C	C	6/10/12	6/10/12	I	LA	AQUA-NAUTIC SPECIALIST
2012996914	NOVI	NOTOPHTHALMUS	VIRIDESCENS	EASTERN	NEWT	LIV	5	NO	US	CA	T	W	C	C	6/11/12	6/11/12	I	BN	Science Kit, Inc - Boreal
2012997234	CYPY	CYNOPS	PYRRHOGASTER	JAPANESE FIREBELLY	NEWT	LIV	165	NO	HK	HK	T	C	C	C	6/12/12	6/10/12	I	NY	Transship Discounts Ltd.
2012997558	TRIH	TRITURUS	HONGKONGENSIS	ALPINE	NEWT	LIV	1,600	NO	HK	HK	T	C	C	C	6/12/12	6/13/12	I	TP	5-D TROPICAL INC.
2012997558	CYOR	CYNOPS	ORIENTALIS	CHINESE DWARF	NEWT	LIV	500	NO	HK	HK	T	C	C	C	6/12/12	6/13/12	I	TP	5-D TROPICAL INC.
2012998928	CYPY	CYNOPS	PYRRHOGASTER	JAPANESE FIREBELLY	NEWT	LIV	30	NO	HK	HK	T	C	C	C	6/14/12	6/3/12	I	NY	Transship Discounts Ltd.
2013208620	TRIH	TRITURUS	HONGKONGENSIS	ALPINE	NEWT	LIV	1,600	NO	HK	HK	T	C	C	C	1/2/13	1/3/13	I	TP	5-D TROPICAL INC.
2013209142	PACB	PACHYTRITON	BREVIPES	TSITOU	NEWT	LIV	90	NO	HK	HK	T	C	C	C	1/3/13	12/30/12	I	NY	Transship Discounts Ltd.
2013209142	CYPY	CYNOPS	PYRRHOGASTER	JAPANESE FIREBELLY	NEWT	LIV	105	NO	HK	HK	T	C	C	C	1/3/13	12/30/12	I	NY	Transship Discounts Ltd.
2013210224	CYOR	CYNOPS	ORIENTALIS	CHINESE DWARF	NEWT	LIV	150	NO	SG	SG	T	C	C	C	1/6/13	1/6/13	I	LA	PET-SIAM INTERNATIONAL
2013210238	CYOR	CYNOPS	ORIENTALIS	CHINESE DWARF	NEWT	LIV	675	NO	HK	HK	T	C	C	C	1/6/13	1/6/13	I	LA	DOLPHIN INTERNATIONAL
2013210238	PACB	PACHYTRITON	BREVIPES	TSITOU	NEWT	LIV	150	NO	HK	HK	T	C	C	C	1/6/13	1/6/13	I	LA	DOLPHIN INTERNATIONAL
2013211837	CNP?	CYNOPS	SPECIES	FIREBELLY	NEWT	LIV	1,030	NO	CN	HK	T	W	C	C	1/8/13	1/6/13	I	LA	AQUA-NAUTIC SPECIALIST
2013211837	PLAB	PACHYTRITON	LABIATUS	UNTERSTEIN'S	NEWT	LIV	445	NO	CN	HK	T	W	C	C	1/8/13	1/6/13	I	LA	AQUA-NAUTIC SPECIALIST
2013213577	NOVI	NOTOPHTHALMUS	VIRIDESCENS	EASTERN	NEWT	LIV	3	NO	US	CA	T	W	C	C	1/11/13	1/9/13	I	BN	Science Kit, Inc - Boreal
2013213953	CYOR	CYNOPS	ORIENTALIS	CHINESE DWARF	NEWT	LIV	375	NO	HK	HK	T	C	C	C	1/13/13	1/13/13	I	LA	DOLPHIN INTERNATIONAL
2013213967	CNP?	CYNOPS	SPECIES	FIREBELLY	NEWT	LIV	880	NO	CN	HK	T	W	C	C	1/13/13	1/13/13	I	LA	AQUA-NAUTIC SPECIALIST
2013213967	PLAB	PACHYTRITON	LABIATUS	UNTERSTEIN'S	NEWT	LIV	100	NO	CN	HK	T	W	C	C	1/13/13	1/13/13	I	LA	AQUA-NAUTIC SPECIALIST
2013213983	CYOR	CYNOPS	ORIENTALIS	CHINESE DWARF	NEWT	LIV	70	NO	SG	SG	T	C	C	C	1/13/13	1/13/13	I	LA	PET-SIAM INTERNATIONAL
2013214783	TRIH	TRITURUS	HONGKONGENSIS	ALPINE	NEWT	LIV	1,600	NO	HK	HK	T	C	C	C	1/15/13	1/16/13	I	TP	5-D TROPICAL INC.
2013214783	CYOR	CYNOPS	ORIENTALIS	CHINESE DWARF	NEWT	LIV	500	NO	HK	HK	T	C	C	C	1/15/13	1/16/13	I	TP	5-D TROPICAL INC.
2013216678	CYPY	CYNOPS	PYRRHOGASTER	JAPANESE FIREBELLY	NEWT	LIV	105	NO	HK	HK	T	C	C	C	1/18/13	1/13/13	I	NY	Transship Discounts Ltd.
2013216678	PACB	PACHYTRITON	BREVIPES	TSITOU	NEWT	LIV	90	NO	HK	HK	T	C	C	C	1/18/13	1/13/13	I	NY	Transship Discounts Ltd.
2013217303	CYOR	CYNOPS	ORIENTALIS	CHINESE DWARF	NEWT	LIV	625	NO	HK	HK	T	C	C	C	1/20/13	1/20/13	I	LA	DOLPHIN INTERNATIONAL
2013217348	CNP?	CYNOPS	SPECIES	FIREBELLY	NEWT	LIV	1,020	NO	CN	HK	T	W	C	C	1/20/13	1/20/13	I	LA	AQUA-NAUTIC SPECIALIST
2013219574	TRIH	TRITURUS	HONGKONGENSIS	ALPINE	NEWT	LIV	1,600	NO	HK	HK	T	C	C	C	1/23/13	1/24/13	I	TP	5-D TROPICAL INC.
2013219574	CYOR	CYNOPS	ORIENTALIS	CHINESE DWARF	NEWT	LIV	500	NO	HK	HK	T	C	C	C	1/23/13	1/24/13	I	TP	5-D TROPICAL INC.
2013219745	CYPY	CYNOPS	PYRRHOGASTER	JAPANESE FIREBELLY	NEWT	LIV	315	NO	HK	HK	T	C	C	C	1/23/13	1/20/13	I	NY	Transship Discounts Ltd.
2013219745	PACB	PACHYTRITON	BREVIPES	TSITOU	NEWT	LIV	30	NO	HK	HK	T	C	C	C	1/23/13	1/20/13	I	NY	Transship Discounts Ltd.
2013220532	TRIT	TRITURUS	SPECIES		NEWT	LIV	350	NO	HK	HK	T	W	C	C	1/24/13	1/24/13	I	SF	PAN OCEAN AQUARIUM
2013221496	CYOR	CYNOPS	ORIENTALIS	CHINESE DWARF	NEWT	LIV	125	NO	HK	HK	T	C	C	C	1/27/13	1/27/13	I	LA	DOLPHIN INTERNATIONAL
2013221496	PACB	PACHYTRITON	BREVIPES	TSITOU	NEWT	LIV	250	NO	HK	HK	T	C	C	C	1/27/13	1/27/13	I	LA	DOLPHIN INTERNATIONAL
2013221518	CNP?	CYNOPS	SPECIES	FIREBELLY	NEWT	LIV	1,320	NO	CN	HK	T	W	C	C	1/27/13	1/27/13	I	LA	AQUA-NAUTIC SPECIALIST
2013221518	PLAB	PACHYTRITON	LABIATUS	UNTERSTEIN'S	NEWT	LIV	100	NO	CN	HK	T	W	C	C	1/27/13	1/27/13	I	LA	AQUA-NAUTIC SPECIALIST
2013222977	CYOR	CYNOPS	ORIENTALIS	CHINESE DWARF	NEWT	LIV	500	NO	HK	HK	T	C	C	C	1/29/13	1/31/13	I	TP	5-D TROPICAL INC.
2013223555	NOVI	NOTOPHTHALMUS	VIRIDESCENS	EASTERN	NEWT	LIV	2	NO	US	CA	T	W	C	C	1/30/13	1/29/13	I	BN	Science Kit, Inc - Boreal
2013225283	CYOR	CYNOPS	ORIENTALIS	CHINESE DWARF	NEWT	LIV	300	NO	HK	HK	T	C	C	C	2/3/13	2/3/13	I	LA	DOLPHIN INTERNATIONAL
2013225283	PACB	PACHYTRITON	BREVIPES	TSITOU	NEWT	LIV	100	NO	HK	HK	T	C	C	C	2/3/13	2/3/13	I	LA	DOLPHIN INTERNATIONAL
2013225294	CYOR	CYNOPS	ORIENTALIS	CHINESE DWARF	NEWT	LIV	75	NO	SG	SG	T	C	C	C	2/3/13	2/3/13	I	LA	PET-SIAM INTERNATIONAL
2013226891	CYPY	CYNOPS	PYRRHOGASTER	JAPANESE FIREBELLY	NEWT	LIV	210	NO	HK	HK	T	C	C	C	2/6/13	2/3/13	I	NY	Transship Discounts Ltd.

2013227183	CYOR	CYNOPS	ORIENTALIS	CHINESE DWARF	NEWT	LIV	250	NO	HK	HK	T	C	C	C	2/6/13	2/7/13	I	TP	5-D TROPICAL INC.
2013227183	TRIH	TRITURUS	HONGKONGENSIS	ALPINE	NEWT	LIV	1,600	NO	HK	HK	T	C	C	C	2/6/13	2/7/13	I	TP	5-D TROPICAL INC.
2013228627	NOVI	NOTOPHTHALMUS	VIRIDESCENS	EASTERN	NEWT	LIV	8	NO	US	CA	T	W	C	C	2/8/13	2/6/13	I	BN	Science Kit, Inc - Boreal
2013228649	CYOR	CYNOPS	ORIENTALIS	CHINESE DWARF	NEWT	LIV	300	NO	HK	HK	T	W	C	C	2/8/13	2/7/13	I	LA	TWO AMIGOS IMPORTS, INC.
2013228649	PCHI	PARAMESOTRITON	CHINENSIS	CHINESE WARTY	NEWT	LIV	200	NO	HK	HK	T	W	C	C	2/8/13	2/7/13	I	LA	TWO AMIGOS IMPORTS, INC.
2013229032	CNP?	CYNOPS	SPECIES	FIREBELLY	NEWT	LIV	140	NO	CN	HK	T	W	C	C	2/9/13	2/3/13	I	LA	AQUA-NAUTIC SPECIALIST
2013229032	PLAB	PACHYTRITON	LABIATUS	UNTERSTEIN'S	NEWT	LIV	100	NO	CN	HK	T	W	C	C	2/9/13	2/3/13	I	LA	AQUA-NAUTIC SPECIALIST
2013229047	CYOR	CYNOPS	ORIENTALIS	CHINESE DWARF	NEWT	LIV	110	NO	SG	SG	T	C	C	C	2/9/13	2/9/13	I	LA	PET-SIAM INTERNATIONAL
2013229059	CNP?	CYNOPS	SPECIES	FIREBELLY	NEWT	LIV	440	NO	CN	HK	T	W	C	C	2/9/13	2/9/13	I	LA	AQUA-NAUTIC SPECIALIST
2013229112	CYOR	CYNOPS	ORIENTALIS	CHINESE DWARF	NEWT	LIV	375	NO	HK	HK	T	C	C	C	2/10/13	2/10/13	I	LA	DOLPHIN INTERNATIONAL
2013229112	PACB	PACHYTRITON	BREVIPES	TSITOU	NEWT	LIV	50	NO	HK	HK	T	C	C	C	2/10/13	2/10/13	I	LA	DOLPHIN INTERNATIONAL
2013230711	CYPY	CYNOPS	PYRRHOGASTER	JAPANESE FIREBELLY	NEWT	LIV	105	NO	HK	HK	T	C	C	C	2/13/13	2/10/13	I	NY	Transship Discounts Ltd.
2013230711	PACB	PACHYTRITON	BREVIPES	TSITOU	NEWT	LIV	30	NO	HK	HK	T	C	C	C	2/13/13	2/10/13	I	NY	Transship Discounts Ltd.
2013231844	NOVI	NOTOPHTHALMUS	VIRIDESCENS	EASTERN	NEWT	LIV	1	NO	US	CA	T	W	C	C	2/15/13	2/12/13	I	BN	Science Kit, Inc - Boreal
2013232360	SASA	SALAMANDRA	SALAMANDRA	EUROPEAN FIRE	SALAMANDER	LIV	450	NO	SI	SI	T	C	C	C	2/15/13	2/15/13	I	MI	TWO AMIGOS IMPORTS, INC.
2013232580	CYOR	CYNOPS	ORIENTALIS	CHINESE DWARF	NEWT	LIV	415	NO	HK	HK	T	C	C	C	2/17/13	2/17/13	I	LA	DOLPHIN INTERNATIONAL
2013232580	PACB	PACHYTRITON	BREVIPES	TSITOU	NEWT	LIV	50	NO	HK	HK	T	C	C	C	2/17/13	2/17/13	I	LA	DOLPHIN INTERNATIONAL
2013233031	PACB	PACHYTRITON	BREVIPES	TSITOU	NEWT	LIV	150	NO	HK	HK	T	C	C	C	2/19/13	2/17/13	I	NY	Transship Discounts Ltd.
2013233524	SASA	SALAMANDRA	SALAMANDRA	EUROPEAN FIRE	SALAMANDER	LIV	682	NO	SI	SI	T	W	C	C	2/19/13	2/19/13	I	LA	CALIFORNIA ZOOLOGICAL SUPPLY
2013233993	CYOR	CYNOPS	ORIENTALIS	CHINESE DWARF	NEWT	LIV	250	NO	HK	HK	T	C	C	C	2/20/13	2/21/13	I	TP	5-D TROPICAL INC.
2013233993	TRIH	TRITURUS	HONGKONGENSIS	ALPINE	NEWT	LIV	1,600	NO	HK	HK	T	C	C	C	2/20/13	2/21/13	I	TP	5-D TROPICAL INC.
2013235830	CYOR	CYNOPS	ORIENTALIS	CHINESE DWARF	NEWT	LIV	550	NO	HK	HK	T	C	C	C	2/24/13	2/24/13	I	LA	DOLPHIN INTERNATIONAL
2013235858	CYOR	CYNOPS	ORIENTALIS	CHINESE DWARF	NEWT	LIV	96	NO	SG	SG	T	C	C	C	2/24/13	2/24/13	I	LA	PET-SIAM INTERNATIONAL
2013235865	CNP?	CYNOPS	SPECIES	FIREBELLY	NEWT	LIV	1,330	NO	CN	HK	T	W	C	C	2/24/13	2/24/13	I	LA	AQUA-NAUTIC SPECIALIST
2013236487	PACB	PACHYTRITON	BREVIPES	TSITOU	NEWT	LIV	90	NO	HK	HK	T	C	C	C	2/25/13	2/24/13	I	NY	Transship Discounts Ltd.
2013236487	CYPY	CYNOPS	PYRRHOGASTER	JAPANESE FIREBELLY	NEWT	LIV	420	NO	HK	HK	T	C	C	C	2/25/13	2/24/13	I	NY	Transship Discounts Ltd.
2013237708	NOVI	NOTOPHTHALMUS	VIRIDESCENS	EASTERN	NEWT	LIV	5	NO	US	CA	T	W	C	C	2/26/13	2/26/13	I	BN	Science Kit, Inc - Boreal
2013237926	CYOR	CYNOPS	ORIENTALIS	CHINESE DWARF	NEWT	LIV	500	NO	HK	HK	T	C	C	C	2/27/13	2/28/13	I	TP	5-D TROPICAL INC.
2013239570	CYOR	CYNOPS	ORIENTALIS	CHINESE DWARF	NEWT	LIV	550	NO	HK	HK	T	C	C	C	3/3/13	3/3/13	I	LA	DOLPHIN INTERNATIONAL
2013239570	PACB	PACHYTRITON	BREVIPES	TSITOU	NEWT	LIV	100	NO	HK	HK	T	C	C	C	3/3/13	3/3/13	I	LA	DOLPHIN INTERNATIONAL
2013239572	CYOR	CYNOPS	ORIENTALIS	CHINESE DWARF	NEWT	LIV	75	NO	SG	SG	T	C	C	C	3/3/13	3/3/13	I	LA	PET-SIAM INTERNATIONAL
2013239607	CNP?	CYNOPS	SPECIES	FIREBELLY	NEWT	LIV	720	NO	CN	HK	T	W	C	C	3/3/13	3/3/13	I	LA	AQUA-NAUTIC SPECIALIST
2013239798	PACB	PACHYTRITON	BREVIPES	TSITOU	NEWT	LIV	10	NO	HK	HK	T	C	C	C	3/4/13	3/3/13	I	NY	Transship Discounts Ltd.
2013239798	CYPY	CYNOPS	PYRRHOGASTER	JAPANESE FIREBELLY	NEWT	LIV	10	NO	HK	HK	T	C	C	C	3/4/13	3/3/13	I	NY	Transship Discounts Ltd.
2013241245	CYOR	CYNOPS	ORIENTALIS	CHINESE DWARF	NEWT	LIV	500	NO	HK	HK	T	C	C	C	3/6/13	3/7/13	I	TP	5-D TROPICAL INC.
2013241245	TRIH	TRITURUS	HONGKONGENSIS	ALPINE	NEWT	LIV	1,600	NO	HK	HK	T	C	C	C	3/6/13	3/7/13	I	TP	5-D TROPICAL INC.
2013241346	NOVI	NOTOPHTHALMUS	VIRIDESCENS	EASTERN	NEWT	LIV	6	NO	US	CA	T	W	C	C	3/6/13	3/6/13	I	BN	Science Kit, Inc - Boreal
2013242897	CYOR	CYNOPS	ORIENTALIS	CHINESE DWARF	NEWT	LIV	400	NO	HK	HK	T	C	C	C	3/10/13	3/10/13	I	LA	DOLPHIN INTERNATIONAL
2013242897	PACB	PACHYTRITON	BREVIPES	TSITOU	NEWT	LIV	150	NO	HK	HK	T	C	C	C	3/10/13	3/10/13	I	LA	DOLPHIN INTERNATIONAL
2013242945	PLAB	PACHYTRITON	LABIATUS	UNTERSTEIN'S	NEWT	LIV	100	NO	CN	HK	T	W	C	C	3/10/13	3/10/13	I	LA	AQUA-NAUTIC SPECIALIST
2013242945	CNP?	CYNOPS	SPECIES	FIREBELLY	NEWT	LIV	1,600	NO	CN	HK	T	W	C	C	3/10/13	3/10/13	I	LA	AQUA-NAUTIC SPECIALIST
2013243516	CYPY	CYNOPS	PYRRHOGASTER	JAPANESE FIREBELLY	NEWT	LIV	6	NO	JP	JP	S	W	C	C	3/11/13	3/11/13	I	SF	UNIVERSITY OF CALIFORNIA, DAVIS
2013244401	TRIH	TRITURUS	HONGKONGENSIS	ALPINE	NEWT	LIV	1,600	NO	HK	HK	T	C	C	C	3/13/13	3/14/13	I	TP	5-D TROPICAL INC.
2013246482	CYOR	CYNOPS	ORIENTALIS	CHINESE DWARF	NEWT	LIV	425	NO	HK	HK	T	C	C	C	3/18/13	3/18/13	I	LA	DOLPHIN INTERNATIONAL
2013246482	PACB	PACHYTRITON	BREVIPES	TSITOU	NEWT	LIV	100	NO	HK	HK	T	C	C	C	3/18/13	3/18/13	I	LA	DOLPHIN INTERNATIONAL
2013246795	PLAB	PACHYTRITON	LABIATUS	UNTERSTEIN'S	NEWT	LIV	45	NO	CN	HK	T	W	C	C	3/18/13	3/18/13	I	LA	AQUA-NAUTIC SPECIALIST
2013246795	CNP?	CYNOPS	SPECIES	FIREBELLY	NEWT	LIV	740	NO	CN	HK	T	W	C	C	3/18/13	3/18/13	I	LA	AQUA-NAUTIC SPECIALIST
2013247078	CYPY	CYNOPS	PYRRHOGASTER	JAPANESE FIREBELLY	NEWT	LIV	510	NO	HK	HK	T	C	C	C	3/19/13	3/18/13	I	NY	Transship Discounts Ltd.
2013248989	TRIT	TRITURUS	SPECIES		NEWT	LIV	350	NO	HK	HK	T	C	C	C	3/21/13	3/21/13	I	SF	PAN OCEAN AQUARIUM
2013249956	CNP?	CYNOPS	SPECIES	FIREBELLY	NEWT	LIV	600	NO	CN	HK	T	W	C	C	3/24/13	3/24/13	I	LA	AQUA-NAUTIC SPECIALIST
2013249956	PLAB	PACHYTRITON	LABIATUS	UNTERSTEIN'S	NEWT	LIV	100	NO	CN	HK	T	W	C	C	3/24/13	3/24/13	I	LA	AQUA-NAUTIC SPECIALIST
2013250817	CYPY	CYNOPS	PYRRHOGASTER	JAPANESE FIREBELLY	NEWT	LIV	105	NO	HK	HK	T	C	C	C	3/26/13	3/24/13	I	NY	Transship Discounts Ltd.
2013251814	TRIH	TRITURUS	HONGKONGENSIS	ALPINE	NEWT	LIV	1,600	NO	HK	HK	T	C	C	C	3/27/13	3/28/13	I	TP	5-D TROPICAL INC.
2013251814	CYOR	CYNOPS	ORIENTALIS	CHINESE DWARF	NEWT	LIV	500	NO	HK	HK	T	C	C	C	3/27/13	3/28/13	I	TP	5-D TROPICAL INC.
2013252164	CYOR	CYNOPS	ORIENTALIS	CHINESE DWARF	NEWT	LIV	1,040	NO	HK	HK	T	C	C	C	3/28/13	3/28/13	I	TP	5-D TROPICAL, INC.
2013253199	TCRI	TRITURUS	CRISTATUS	NORTHERN CRESTED	NEWT	LIV	12	NO	DE	DE	T	C	C	C	3/29/13	3/28/13	I	MI	TWO AMIGOS IMPORTS, INC.
2013253730	CYOR	CYNOPS	ORIENTALIS	CHINESE DWARF	NEWT	LIV	625	NO	HK	HK	T	C	C	C	3/31/13	3/31/13	I	LA	DOLPHIN INTERNATIONAL
2013253730	PACB	PACHYTRITON	BREVIPES	TSITOU	NEWT	LIV	150	NO	HK	HK	T	C	C	C	3/31/13	3/31/13	I	LA	DOLPHIN INTERNATIONAL
2013253748	CNP?	CYNOPS	SPECIES	FIREBELLY	NEWT	LIV	140	NO	CN	HK	T	W	C	C	3/31/13	3/31/13	I	LA	AQUA-NAUTIC SPECIALIST
2013253756	CYOR	CYNOPS	ORIENTALIS	CHINESE DWARF	NEWT	LIV	360	NO	SG	SG	T	C	C	C	3/31/13	3/31/13	I	LA	PET-SIAM INTERNATIONAL

2013255921	TRIH	TRITURUS	HONGKONGENSIS	ALPINE	NEWT	LIV	1,600	NO	HK	HK	T	C	C	C	4/3/13	4/4/13	I	TP	5-D TROPICAL INC.
2013257426	CYOR	CYNOPS	ORIENTALIS	CHINESE DWARF	NEWT	LIV	750	NO	HK	HK	T	C	C	C	4/7/13	4/7/13	I	LA	DOLPHIN INTERNATIONAL
2013257480	CNP?	CYNOPS	SPECIES	FIREBELLY	NEWT	LIV	1,050	NO	CN	HK	T	W	C	C	4/7/13	4/7/13	I	LA	AQUA-NAUTIC SPECIALIST
2013259446	CYPY	CYNOPS	PYRRHOGASTER	JAPANESE FIREBELLY	NEWT	LIV	270	NO	HK	HK	T	C	C	C	4/10/13	4/7/13	I	NY	Transship Discounts Ltd.
2013259446	PACB	PACHYTRITON	BREVIPES	TSITOU	NEWT	LIV	60	NO	HK	HK	T	C	C	C	4/10/13	4/7/13	I	NY	Transship Discounts Ltd.
2013260322	PLD?	PLEURODELES	SPECIES	RIBBED	NEWT	LIV	30	NO	CZ	CZ	T	C	C	C	4/12/13	4/12/13	I	AT	SUN PET LTD.
2013261510	CYOR	CYNOPS	ORIENTALIS	CHINESE DWARF	NEWT	LIV	750	NO	HK	HK	T	C	C	C	4/14/13	4/14/13	I	LA	DOLPHIN INTERNATIONAL
2013261510	PACB	PACHYTRITON	BREVIPES	TSITOU	NEWT	LIV	250	NO	HK	HK	T	C	C	C	4/14/13	4/14/13	I	LA	DOLPHIN INTERNATIONAL
2013261553	CYOR	CYNOPS	ORIENTALIS	CHINESE DWARF	NEWT	LIV	75	NO	SG	SG	T	C	C	C	4/14/13	4/14/13	I	LA	PET-SIAM INTERNATIONAL
2013261558	PLAB	PACHYTRITON	LABIATUS	UNTERSTEIN'S	NEWT	LIV	100	NO	CN	HK	T	W	C	C	4/14/13	4/14/13	I	LA	AQUA-NAUTIC SPECIALIST
2013261558	CNP?	CYNOPS	SPECIES	FIREBELLY	NEWT	LIV	440	NO	CN	HK	T	W	C	C	4/14/13	4/14/13	I	LA	AQUA-NAUTIC SPECIALIST
2013261952	CYOR	CYNOPS	ORIENTALIS	CHINESE DWARF	NEWT	LIV	200	NO	HK	HK	T	W	C	C	4/15/13	4/15/13	I	MI	TWO AMIGOS IMPORTS, INC.
2013261952	PCHI	PARAMESOTRITON	CHINENSIS	CHINESE WARTY	NEWT	LIV	100	NO	HK	HK	T	W	C	C	4/15/13	4/15/13	I	MI	TWO AMIGOS IMPORTS, INC.
2013261952	PLAB	PACHYTRITON	LABIATUS	UNTERSTEIN'S	NEWT	LIV	100	NO	HK	HK	T	W	C	C	4/15/13	4/15/13	I	MI	TWO AMIGOS IMPORTS, INC.
2013261952	TYL?	TYLOTOTRITON	SPECIES	CROCODILE	NEWT	LIV	50	NO	HK	HK	T	W	C	C	4/15/13	4/15/13	I	MI	TWO AMIGOS IMPORTS, INC.
2013262599	CYPY	CYNOPS	PYRRHOGASTER	JAPANESE FIREBELLY	NEWT	LIV	60	NO	HK	HK	T	C	C	C	4/16/13	3/31/13	I	NY	Transship Discounts Ltd.
2013262918	CYPY	CYNOPS	PYRRHOGASTER	JAPANESE FIREBELLY	NEWT	LIV	405	NO	HK	HK	T	C	C	C	4/16/13	4/14/13	I	NY	Transship Discounts Ltd.
2013262918	PACB	PACHYTRITON	BREVIPES	TSITOU	NEWT	LIV	60	NO	HK	HK	T	C	C	C	4/16/13	4/14/13	I	NY	Transship Discounts Ltd.
2013263623	TRIH	TRITURUS	HONGKONGENSIS	ALPINE	NEWT	LIV	1,600	NO	HK	HK	T	C	C	C	4/17/13	4/18/13	I	TP	5-D TROPICAL INC.
2013263626	CYOR	CYNOPS	ORIENTALIS	CHINESE DWARF	NEWT	LIV	1,440	NO	HK	HK	T	C	C	C	4/17/13	4/18/13	I	TP	5-D TROPICAL, INC.
2013265425	PLAB	PACHYTRITON	LABIATUS	UNTERSTEIN'S	NEWT	LIV	45	NO	CN	HK	T	W	C	C	4/21/13	4/21/13	I	LA	AQUA-NAUTIC SPECIALIST
2013268322	CYPY	CYNOPS	PYRRHOGASTER	JAPANESE FIREBELLY	NEWT	LIV	210	NO	HK	HK	T	C	C	C	4/24/13	4/21/13	I	NY	Transship Discounts Ltd.
2013270190	CYOR	CYNOPS	ORIENTALIS	CHINESE DWARF	NEWT	LIV	1,050	NO	HK	HK	T	C	C	C	4/28/13	4/28/13	I	LA	DOLPHIN INTERNATIONAL
2013270190	PACB	PACHYTRITON	BREVIPES	TSITOU	NEWT	LIV	50	NO	HK	HK	T	C	C	C	4/28/13	4/28/13	I	LA	DOLPHIN INTERNATIONAL
2013270222	CYOR	CYNOPS	ORIENTALIS	CHINESE DWARF	NEWT	LIV	110	NO	SG	SG	T	C	C	C	4/28/13	4/28/13	I	LA	PET-SIAM INTERNATIONAL
2013270448	CYPY	CYNOPS	PYRRHOGASTER	JAPANESE FIREBELLY	NEWT	LIV	165	NO	HK	HK	T	C	C	C	4/29/13	4/28/13	I	NY	Transship Discounts Ltd.
2013270448	PACB	PACHYTRITON	BREVIPES	TSITOU	NEWT	LIV	60	NO	HK	HK	T	C	C	C	4/29/13	4/28/13	I	NY	Transship Discounts Ltd.
2013271332	CNP?	CYNOPS	SPECIES	FIREBELLY	NEWT	LIV	600	NO	CN	HK	T	W	C	C	4/30/13	4/28/13	I	LA	AQUA-NAUTIC SPECIALIST
2013273695	CYOR	CYNOPS	ORIENTALIS	CHINESE DWARF	NEWT	LIV	375	NO	HK	HK	T	C	C	C	5/5/13	5/5/13	I	LA	DOLPHIN INTERNATIONAL
2013273764	CNP?	CYNOPS	SPECIES	FIREBELLY	NEWT	LIV	1,460	NO	CN	HK	T	W	C	C	5/5/13	5/5/13	I	LA	AQUA-NAUTIC SPECIALIST
2013274837	CYPY	CYNOPS	PYRRHOGASTER	JAPANESE FIREBELLY	NEWT	LIV	52	NO	HK	HK	T	C	C	C	5/7/13	5/5/13	I	NY	Transship Discounts Ltd.
2013277041	CYOR	CYNOPS	ORIENTALIS	CHINESE DWARF	NEWT	LIV	425	NO	HK	HK	T	C	C	C	5/12/13	5/12/13	I	LA	DOLPHIN INTERNATIONAL
2013277041	PACB	PACHYTRITON	BREVIPES	TSITOU	NEWT	LIV	250	NO	HK	HK	T	C	C	C	5/12/13	5/12/13	I	LA	DOLPHIN INTERNATIONAL
2013277084	CYOR	CYNOPS	ORIENTALIS	CHINESE DWARF	NEWT	LIV	35	NO	SG	SG	T	C	C	C	5/12/13	5/12/13	I	LA	PET-SIAM INTERNATIONAL
2013277107	CNP?	CYNOPS	SPECIES	FIREBELLY	NEWT	LIV	580	NO	CN	HK	T	W	C	C	5/12/13	5/12/13	I	LA	AQUA-NAUTIC SPECIALIST
2013278505	SASA	SALAMANDRA	SALAMANDRA	EUROPEAN FIRE	SALAMANDER	LIV	750	NO	SI	SI	T	C	C	C	5/15/13	5/14/13	I	MI	NAME REMOVED
2013278655	CYPY	CYNOPS	PYRRHOGASTER	JAPANESE FIREBELLY	NEWT	LIV	375	NO	HK	HK	T	C	C	C	5/15/13	5/12/13	I	NY	Transship Discounts Ltd.
2013278655	PACB	PACHYTRITON	BREVIPES	TSITOU	NEWT	LIV	60	NO	HK	HK	T	C	C	C	5/15/13	5/12/13	I	NY	Transship Discounts Ltd.
2013278867	TRIH	TRITURUS	HONGKONGENSIS	ALPINE	NEWT	LIV	1,600	NO	HK	HK	T	C	C	C	5/15/13	5/16/13	I	TP	5-D TROPICAL, INC.
2013278867	CYOR	CYNOPS	ORIENTALIS	CHINESE DWARF	NEWT	LIV	500	NO	HK	HK	T	C	C	C	5/15/13	5/16/13	I	TP	5-D TROPICAL, INC.
2013278875	CYOR	CYNOPS	ORIENTALIS	CHINESE DWARF	NEWT	LIV	2,100	NO	HK	HK	T	C	C	C	5/15/13	5/16/13	I	TP	5-D TROPICAL INC.
2013280322	CYOR	CYNOPS	ORIENTALIS	CHINESE DWARF	NEWT	LIV	475	NO	HK	HK	T	C	C	C	5/19/13	5/19/13	I	LA	DOLPHIN INTERNATIONAL
2013280343	PLAB	PACHYTRITON	LABIATUS	UNTERSTEIN'S	NEWT	LIV	145	NO	CN	HK	T	W	C	C	5/19/13	5/19/13	I	LA	AQUA-NAUTIC SPECIALIST
2013280343	CNP?	CYNOPS	SPECIES	FIREBELLY	NEWT	LIV	1,180	NO	CN	HK	T	W	C	C	5/19/13	5/19/13	I	LA	AQUA-NAUTIC SPECIALIST
2013280913	CYPY	CYNOPS	PYRRHOGASTER	JAPANESE FIREBELLY	NEWT	LIV	325	NO	HK	HK	T	C	C	C	5/20/13	5/19/13	I	NY	Transship Discounts Ltd.
2013283783	CYOR	CYNOPS	ORIENTALIS	CHINESE DWARF	NEWT	LIV	125	NO	HK	HK	T	C	C	C	5/26/13	5/26/13	I	LA	DOLPHIN INTERNATIONAL
2013283795	CYOR	CYNOPS	ORIENTALIS	CHINESE DWARF	NEWT	LIV	75	NO	SG	SG	T	C	C	C	5/26/13	5/26/13	I	LA	PET-SIAM INTERNATIONAL
2013283809	CNP?	CYNOPS	SPECIES	FIREBELLY	NEWT	LIV	290	NO	CN	HK	T	W	C	C	5/26/13	5/26/13	I	LA	AQUA-NAUTIC SPECIALIST
2013284551	CYPY	CYNOPS	PYRRHOGASTER	JAPANESE FIREBELLY	NEWT	LIV	345	NO	HK	HK	T	C	C	C	5/28/13	5/26/13	I	NY	Transship Discounts Ltd.
2013284551	PACB	PACHYTRITON	BREVIPES	TSITOU	NEWT	LIV	30	NO	HK	HK	T	C	C	C	5/28/13	5/26/13	I	NY	Transship Discounts Ltd.
2013285222	TRIH	TRITURUS	HONGKONGENSIS	ALPINE	NEWT	LIV	1,600	NO	HK	HK	T	C	C	C	5/29/13	5/30/13	I	TP	5-D TROPICAL INC.
2013285238	CYOR	CYNOPS	ORIENTALIS	CHINESE DWARF	NEWT	LIV	1,600	NO	HK	HK	T	C	C	C	5/29/13	5/30/13	I	TP	5-D TROPICAL INC.
2013286634	BOG?	BOLITOGLOSSA	SPECIES	MUSHROOMTONGUE	SALAMANDER	LIV	8	NO	GT	GT	S	W	C	C	5/31/13	5/31/13	I	MI	THE TOLEDO ZOO
2013287014	CYOR	CYNOPS	ORIENTALIS	CHINESE DWARF	NEWT	LIV	600	NO	HK	HK	T	C	C	C	6/2/13	6/2/13	I	LA	DOLPHIN INTERNATIONAL
2013287014	PACB	PACHYTRITON	BREVIPES	TSITOU	NEWT	LIV	50	NO	HK	HK	T	C	C	C	6/2/13	6/2/13	I	LA	DOLPHIN INTERNATIONAL
2013287067	CNP?	CYNOPS	SPECIES	FIREBELLY	NEWT	LIV	600	NO	CN	HK	T	W	C	C	6/2/13	6/2/13	I	LA	AQUA-NAUTIC SPECIALIST
2013287067	TRIT	TRITURUS	SPECIES		NEWT	LIV	450	NO	CN	HK	T	W	C	C	6/2/13	6/2/13	I	LA	AQUA-NAUTIC SPECIALIST
2013288583	CYPY	CYNOPS	PYRRHOGASTER	JAPANESE FIREBELLY	NEWT	LIV	135	NO	HK	HK	T	C	C	C	6/5/13	6/2/13	I	NY	Transship Discounts Ltd.
2013289244	CNP?	CYNOPS	SPECIES	FIREBELLY	NEWT	LIV	500	NO	HK	HK	T	C	C	C	6/6/13	6/6/13	I	AT	SUN PET LTD.
2013289489	CYOR	CYNOPS	ORIENTALIS	CHINESE DWARF	NEWT	LIV	250	NO	HK	HK	T	W	C	C	6/6/13	6/5/13	I	MI	TWO AMIGOS IMPORTS, INC.

2013289489	PLAB	PACHYTRITON	LABIATUS	UNTERSTEIN'S	NEWT	LIV	200	NO	HK	HK	T	W	C	C	6/6/13	6/5/13	I	MI	TWO AMIGOS IMPORTS, INC.
2013289489	TYKW	TYLOTOTRITON	KWEICHOWENSIS	KWEICHOW CROCODILE	NEWT	LIV	100	NO	HK	HK	T	W	C	C	6/6/13	6/5/13	I	MI	TWO AMIGOS IMPORTS, INC.
2013289489	TYSH	TYLOTOTRITON	SHANJING	MANDARIN	NEWT	LIV	50	NO	HK	HK	T	W	C	C	6/6/13	6/5/13	I	MI	TWO AMIGOS IMPORTS, INC.
2013289489	TYL?	TYLOTOTRITON	SPECIES	CROCODILE	NEWT	LIV	50	NO	HK	HK	T	W	C	C	6/6/13	6/5/13	I	MI	TWO AMIGOS IMPORTS, INC.
2013289493	TRIT	TRITURUS	SPECIES		NEWT	LIV	350	NO	HK	HK	T	C	C	C	6/6/13	6/6/13	I	SF	PAN OCEAN AQUARIUM
2013290027	AMBM	AMBYSTOMA	MEXICANUM		AXOLOTL	LIV	4	NO	UY	UY	T	F	R	S	3/27/13	3/27/13	I	MI	Exemptions 6 and 7(C)
2013290048	AMBM	AMBYSTOMA	MEXICANUM		AXOLOTL	LIV	4	NO	UY	UY	T	C	R	S	3/27/13	3/27/13	I	MI	NAME REMOVED
2013290502	CYOR	CYNOPS	ORIENTALIS	CHINESE DWARF	NEWT	LIV	300	NO	HK	HK	T	C	C	C	6/9/13	6/9/13	I	LA	DOLPHIN INTERNATIONAL
2013290502	PACB	PACHYTRITON	BREVIPES	TSITOU	NEWT	LIV	100	NO	HK	HK	T	C	C	C	6/9/13	6/9/13	I	LA	DOLPHIN INTERNATIONAL
2013290530	CYOR	CYNOPS	ORIENTALIS	CHINESE DWARF	NEWT	LIV	60	NO	SG	SG	T	C	C	C	6/9/13	6/9/13	I	LA	PET-SIAM INTERNATIONAL
2013290537	CNP?	CYNOPS	SPECIES	FIREBELLY	NEWT	LIV	580	NO	CN	HK	T	W	C	C	6/9/13	6/9/13	I	LA	AQUA-NAUTIC SPECIALIST
2013290537	TRIT	TRITURUS	SPECIES		NEWT	LIV	36	NO	CN	HK	T	W	C	C	6/9/13	6/9/13	I	LA	AQUA-NAUTIC SPECIALIST
2013292113	TRIH	TRITURUS	HONGKONGENSIS	ALPINE	NEWT	LIV	1,600	NO	HK	HK	T	C	C	C	6/12/13	6/13/13	I	TP	5-D TROPICAL, INC.
2013292160	TYSH	TYLOTOTRITON	SHANJING	MANDARIN	NEWT	LIV	10	NO	CA	CA	Z	C	C	C	6/12/13	6/12/13	I	DN	THE DENVER ZOO
2013293933	CYOR	CYNOPS	ORIENTALIS	CHINESE DWARF	NEWT	LIV	125	NO	HK	HK	T	C	C	C	6/16/13	6/16/13	I	LA	DOLPHIN INTERNATIONAL
2013293970	CNP?	CYNOPS	SPECIES	FIREBELLY	NEWT	LIV	489	NO	CN	HK	T	W	C	C	6/16/13	6/16/13	I	LA	AQUA-NAUTIC SPECIALIST
2013295642	TRIH	TRITURUS	HONGKONGENSIS	ALPINE	NEWT	LIV	1,600	NO	HK	HK	T	C	C	C	6/19/13	6/20/13	I	TP	5-D TROPICAL, INC.
2013297157	CYPY	CYNOPS	PYRRHOGASTER	JAPANESE FIREBELLY	NEWT	LIV	210	NO	HK	HK	T	C	C	C	6/21/13	6/16/13	I	NY	Transship Discounts Ltd.
2013297647	CYOR	CYNOPS	ORIENTALIS	CHINESE DWARF	NEWT	LIV	375	NO	HK	HK	T	C	C	C	6/23/13	6/23/13	I	LA	DOLPHIN INTERNATIONAL
2013297647	CNEW	CYNOPS	CYANURUS	CYAN	NEWT	LIV	200	NO	HK	HK	T	C	C	C	6/23/13	6/23/13	I	LA	DOLPHIN INTERNATIONAL
2013297700	CNP?	CYNOPS	SPECIES	FIREBELLY	NEWT	LIV	880	NO	CN	HK	T	W	C	C	6/23/13	6/23/13	I	LA	AQUA-NAUTIC SPECIALIST
2013299159	TRIH	TRITURUS	HONGKONGENSIS	ALPINE	NEWT	LIV	1,600	NO	HK	HK	T	C	C	C	6/26/13	6/26/13	I	TP	5-D TROPICAL, INC.
2013299216	CYPY	CYNOPS	PYRRHOGASTER	JAPANESE FIREBELLY	NEWT	LIV	315	NO	HK	HK	T	C	C	C	6/26/13	6/23/13	I	NY	Transship Discounts Ltd.
2013299216	PACB	PACHYTRITON	BREVIPES	TSITOU	NEWT	LIV	30	NO	HK	HK	T	C	C	C	6/26/13	6/23/13	I	NY	Transship Discounts Ltd.
2013299500	CYOR	CYNOPS	ORIENTALIS	CHINESE DWARF	NEWT	LIV	400	NO	HK	HK	T	W	C	C	6/26/13	6/26/13	I	MI	TWO AMIGOS IMPORTS, INC.
2013299500	PLAB	PACHYTRITON	LABIATUS	UNTERSTEIN'S	NEWT	LIV	150	NO	HK	HK	T	W	C	C	6/26/13	6/26/13	I	MI	TWO AMIGOS IMPORTS, INC.
2013299500	TYKW	TYLOTOTRITON	KWEICHOWENSIS	KWEICHOW CROCODILE	NEWT	LIV	80	NO	HK	HK	T	W	C	C	6/26/13	6/26/13	I	MI	TWO AMIGOS IMPORTS, INC.
2013301049	PACB	PACHYTRITON	BREVIPES	TSITOU	NEWT	LIV	200	NO	HK	HK	T	C	C	C	6/30/13	6/30/13	I	LA	DOLPHIN INTERNATIONAL
2013301077	CYOR	CYNOPS	ORIENTALIS	CHINESE DWARF	NEWT	LIV	35	NO	SG	SG	T	C	C	C	6/30/13	6/30/13	I	LA	PET-SIAM INTERNATIONAL
2013301085	CNP?	CYNOPS	SPECIES	FIREBELLY	NEWT	LIV	880	NO	CN	HK	T	W	C	C	6/30/13	6/30/13	I	LA	AQUA-NAUTIC SPECIALIST
2013302241	CYPY	CYNOPS	PYRRHOGASTER	JAPANESE FIREBELLY	NEWT	LIV	105	NO	HK	HK	T	C	C	C	7/2/13	6/30/13	I	NY	Transship Discounts Ltd.
2013302241	PACB	PACHYTRITON	BREVIPES	TSITOU	NEWT	LIV	120	NO	HK	HK	T	C	C	C	7/2/13	6/30/13	I	NY	Transship Discounts Ltd.
2013302833	CYPY	CYNOPS	PYRRHOGASTER	JAPANESE FIREBELLY	NEWT	LIV	9	NO	JP	JP	T	F	C	C	7/3/13	7/3/13	I	LA	HERP TRADING INC
2013302833	SNEW	CYNOPS	ENSICAUDA	SWORDTAIL	NEWT	LIV	14	NO	JP	JP	T	F	C	C	7/3/13	7/3/13	I	LA	HERP TRADING INC
2013302833	ONJA	ONYCHODACTYLUS	JAPONICUS	JAPANESE CLAWED	SALAMANDER	LIV	20	NO	JP	JP	T	F	C	C	7/3/13	7/3/13	I	LA	HERP TRADING INC
2013303800	CYOR	CYNOPS	ORIENTALIS	CHINESE DWARF	NEWT	LIV	550	NO	HK	HK	T	C	C	C	7/7/13	7/7/13	I	LA	DOLPHIN INTERNATIONAL
2013303800	PACB	PACHYTRITON	BREVIPES	TSITOU	NEWT	LIV	50	NO	HK	HK	T	C	C	C	7/7/13	7/7/13	I	LA	DOLPHIN INTERNATIONAL
2013303800	CNEW	CYNOPS	CYANURUS	CYAN	NEWT	LIV	50	NO	HK	HK	T	C	C	C	7/7/13	7/7/13	I	LA	DOLPHIN INTERNATIONAL
2013303862	CNP?	CYNOPS	SPECIES	FIREBELLY	NEWT	LIV	580	NO	CN	HK	T	W	C	C	7/7/13	7/7/13	I	LA	AQUA-NAUTIC SPECIALIST
2013304923	PACB	PACHYTRITON	BREVIPES	TSITOU	NEWT	LIV	30	NO	HK	HK	T	C	C	C	7/9/13	7/7/13	I	NY	Transship Discounts Ltd.
2013305717	TRIH	TRITURUS	HONGKONGENSIS	ALPINE	NEWT	LIV	1,600	NO	HK	HK	T	C	C	C	7/10/13	7/11/13	I	TP	5-D TROPICAL, INC.
2013307544	CYOR	CYNOPS	ORIENTALIS	CHINESE DWARF	NEWT	LIV	875	NO	HK	HK	T	C	C	C	7/14/13	7/14/13	I	LA	DOLPHIN INTERNATIONAL
2013307544	CNEW	CYNOPS	CYANURUS	CYAN	NEWT	LIV	92	NO	HK	HK	T	C	C	C	7/14/13	7/14/13	I	LA	DOLPHIN INTERNATIONAL
2013307571	CYOR	CYNOPS	ORIENTALIS	CHINESE DWARF	NEWT	LIV	75	NO	SG	SG	T	C	C	C	7/14/13	7/14/13	I	LA	PET-SIAM INTERNATIONAL
2013307579	CNP?	CYNOPS	SPECIES	FIREBELLY	NEWT	LIV	995	NO	CN	HK	T	W	C	C	7/14/13	7/14/13	I	LA	AQUA-NAUTIC SPECIALIST
2013311235	TYKW	TYLOTOTRITON	KWEICHOWENSIS	KWEICHOW CROCODILE	NEWT	LIV	121	NO	CN	HK	T	W	C	C	7/19/13	7/18/13	I	SF	WONDERFUL WORLD PET SUPPLIERS INC
2013311235	TYSH	TYLOTOTRITON	SHANJING	MANDARIN	NEWT	LIV	84	NO	CN	HK	T	W	C	C	7/19/13	7/18/13	I	SF	WONDERFUL WORLD PET SUPPLIERS INC
2013311609	CYOR	CYNOPS	ORIENTALIS	CHINESE DWARF	NEWT	LIV	375	NO	HK	HK	T	C	C	C	7/21/13	7/21/13	I	LA	DOLPHIN INTERNATIONAL
2013311609	PACB	PACHYTRITON	BREVIPES	TSITOU	NEWT	LIV	100	NO	HK	HK	T	C	C	C	7/21/13	7/21/13	I	LA	DOLPHIN INTERNATIONAL
2013311662	CYOR	CYNOPS	ORIENTALIS	CHINESE DWARF	NEWT	LIV	60	NO	SG	SG	T	C	C	C	7/21/13	7/21/13	I	LA	PET-SIAM INTERNATIONAL
2013311673	CNP?	CYNOPS	SPECIES	FIREBELLY	NEWT	LIV	740	NO	ID	HK	T	W	C	C	7/21/13	7/21/13	I	LA	AQUA-NAUTIC SPECIALIST
2013315366	CYOR	CYNOPS	ORIENTALIS	CHINESE DWARF	NEWT	LIV	625	NO	HK	HK	T	C	C	C	7/28/13	7/28/13	I	LA	DOLPHIN INTERNATIONAL
2013315366	PACB	PACHYTRITON	BREVIPES	TSITOU	NEWT	LIV	100	NO	HK	HK	T	C	C	C	7/28/13	7/28/13	I	LA	DOLPHIN INTERNATIONAL
2013315417	CYOR	CYNOPS	ORIENTALIS	CHINESE DWARF	NEWT	LIV	500	NO	SG	SG	T	C	C	C	7/28/13	7/28/13	I	LA	PET-SIAM INTERNATIONAL
2013315422	CNP?	CYNOPS	SPECIES	FIREBELLY	NEWT	LIV	880	NO	CN	HK	T	W	C	C	7/28/13	7/28/13	I	LA	AQUA-NAUTIC SPECIALIST
2013317369	CYPY	CYNOPS	PYRRHOGASTER	JAPANESE FIREBELLY	NEWT	LIV	180	NO	HK	HK	T	C	C	C	7/31/13	7/21/13	I	NY	Transship Discounts Ltd.
2013317369	PACB	PACHYTRITON	BREVIPES	TSITOU	NEWT	LIV	75	NO	HK	HK	T	C	C	C	7/31/13	7/21/13	I	NY	Transship Discounts Ltd.
2013318987	CNEW	CYNOPS	CYANURUS	CYAN	NEWT	LIV	100	NO	HK	HK	T	C	C	C	8/4/13	8/4/13	I	LA	DOLPHIN INTERNATIONAL
2013318987	CYOR	CYNOPS	ORIENTALIS	CHINESE DWARF	NEWT	LIV	330	NO	HK	HK	T	C	C	C	8/4/13	8/4/13	I	LA	DOLPHIN INTERNATIONAL
2013318987	PACB	PACHYTRITON	BREVIPES	TSITOU	NEWT	LIV	50	NO	HK	HK	T	C	C	C	8/4/13	8/4/13	I	LA	DOLPHIN INTERNATIONAL

2013319054	CYOR	CYNOPS	ORIENTALIS	CHINESE DWARF	NEWT	LIV	35	NO	SG	SG	T	C	C	C	8/4/13	8/4/13	I	LA	PET-SIAM INTERNATIONAL
2013319056	CNP?	CYNOPS	SPECIES	FIREBELLY	NEWT	LIV	140	NO	CN	HK	T	W	C	C	8/4/13	8/4/13	I	LA	AQUA-NAUTIC SPECIALIST
2013319426	CYPY	CYNOPS	PYRRHOGASTER	JAPANESE FIREBELLY	NEWT	LIV	315	NO	HK	HK	T	C	C	C	8/5/13	8/4/13	I	NY	Transship Discounts Ltd.
2013320892	CYOR	CYNOPS	ORIENTALIS	CHINESE DWARF	NEWT	LIV	1,120	NO	HK	HK	T	C	C	C	8/7/13	8/8/13	I	TP	5-D TROPICAL INC.
2013322500	CNEW	CYNOPS	CYANURUS	CYAN	NEWT	LIV	50	NO	HK	HK	T	C	C	C	8/11/13	8/11/13	I	LA	DOLPHIN INTERNATIONAL
2013322500	CYOR	CYNOPS	ORIENTALIS	CHINESE DWARF	NEWT	LIV	375	NO	HK	HK	T	C	C	C	8/11/13	8/11/13	I	LA	DOLPHIN INTERNATIONAL
2013322562	CYOR	CYNOPS	ORIENTALIS	CHINESE DWARF	NEWT	LIV	25	NO	SG	SG	T	C	C	C	8/11/13	8/11/13	I	LA	PET-SIAM INTERNATIONAL
2013325818	CNEW	CYNOPS	CYANURUS	CYAN	NEWT	LIV	50	NO	HK	HK	T	C	C	C	8/18/13	8/18/13	I	LA	DOLPHIN INTERNATIONAL
2013325818	CYOR	CYNOPS	ORIENTALIS	CHINESE DWARF	NEWT	LIV	375	NO	HK	HK	T	C	C	C	8/18/13	8/18/13	I	LA	DOLPHIN INTERNATIONAL
2013325872	CNP?	CYNOPS	SPECIES	FIREBELLY	NEWT	LIV	880	NO	HK	HK	T	W	C	C	8/18/13	8/18/13	I	LA	AQUA-NAUTIC SPECIALIST
2013325872	TRIT	TRITURUS	SPECIES		NEWT	LIV	145	NO	HK	HK	T	W	C	C	8/18/13	8/18/13	I	LA	AQUA-NAUTIC SPECIALIST
2013326831	TRIT	TRITURUS	SPECIES		NEWT	LIV	13	NO	FR	FR	T	C	C	C	8/20/13	8/14/13	I	MI	TWO AMIGOS IMPORTS, INC.
2013328979	CYOR	CYNOPS	ORIENTALIS	CHINESE DWARF	NEWT	LIV	250	NO	HK	HK	T	C	C	C	8/25/13	8/25/13	I	LA	DOLPHIN INTERNATIONAL
2013328979	PACB	PACHYTRITON	BREVIPES	TSITOU	NEWT	LIV	50	NO	HK	HK	T	C	C	C	8/25/13	8/25/13	I	LA	DOLPHIN INTERNATIONAL
2013329000	CNP?	CYNOPS	SPECIES	FIREBELLY	NEWT	LIV	280	NO	CN	HK	T	W	C	C	8/25/13	8/25/13	I	LA	AQUA-NAUTIC SPECIALIST
2013329000	TRIT	TRITURUS	SPECIES		NEWT	LIV	145	NO	CN	HK	T	W	C	C	8/25/13	8/25/13	I	LA	AQUA-NAUTIC SPECIALIST
2013330271	CYPY	CYNOPS	PYRRHOGASTER	JAPANESE FIREBELLY	NEWT	LIV	105	NO	HK	HK	T	C	C	C	8/27/13	8/11/13	I	NY	Transship Discounts Ltd.
2013330271	PACB	PACHYTRITON	BREVIPES	TSITOU	NEWT	LIV	30	NO	HK	HK	T	C	C	C	8/27/13	8/11/13	I	NY	Transship Discounts Ltd.
2013331129	CNP?	CYNOPS	SPECIES	FIREBELLY	NEWT	LIV	500	NO	HK	HK	T	C	C	C	8/29/13	8/29/13	I	AT	SUN PET LTD.
2013331135	CYPY	CYNOPS	PYRRHOGASTER	JAPANESE FIREBELLY	NEWT	LIV	105	NO	HK	HK	T	C	C	C	8/29/13	8/18/13	I	NY	Transship Discounts Ltd.
2013331135	PACB	PACHYTRITON	BREVIPES	TSITOU	NEWT	LIV	120	NO	HK	HK	T	C	C	C	8/29/13	8/18/13	I	NY	Transship Discounts Ltd.
2013332398	CYOR	CYNOPS	ORIENTALIS	CHINESE DWARF	NEWT	LIV	125	NO	HK	HK	T	C	C	C	9/1/13	9/1/13	I	LA	DOLPHIN INTERNATIONAL
2013332398	PACB	PACHYTRITON	BREVIPES	TSITOU	NEWT	LIV	250	NO	HK	HK	T	C	C	C	9/1/13	9/1/13	I	LA	DOLPHIN INTERNATIONAL
2013332409	CYOR	CYNOPS	ORIENTALIS	CHINESE DWARF	NEWT	LIV	25	NO	SG	SG	T	C	C	C	9/1/13	9/1/13	I	LA	PET-SIAM INTERNATIONAL
2013332428	CNP?	CYNOPS	SPECIES	FIREBELLY	NEWT	LIV	880	NO	CN	HK	T	W	C	C	9/1/13	9/1/13	I	LA	AQUA-NAUTIC SPECIALIST
2013332428	TRIT	TRITURUS	SPECIES		NEWT	LIV	45	NO	CN	HK	T	W	C	C	9/1/13	9/1/13	I	LA	AQUA-NAUTIC SPECIALIST
2013332901	PACB	PACHYTRITON	BREVIPES	TSITOU	NEWT	LIV	30	NO	HK	HK	T	C	C	C	9/3/13	9/1/13	I	NY	Transship Discounts Ltd.
2013332901	CYPY	CYNOPS	PYRRHOGASTER	JAPANESE FIREBELLY	NEWT	LIV	315	NO	HK	HK	T	C	C	C	9/3/13	9/1/13	I	NY	Transship Discounts Ltd.
2013336055	PACB	PACHYTRITON	BREVIPES	TSITOU	NEWT	LIV	50	NO	HK	HK	T	C	C	C	9/8/13	9/8/13	I	LA	DOLPHIN INTERNATIONAL
2013336072	CNP?	CYNOPS	SPECIES	FIREBELLY	NEWT	LIV	2,100	NO	CN	HK	T	W	C	C	9/8/13	9/8/13	I	LA	AQUA-NAUTIC SPECIALIST
2013336079	CYOR	CYNOPS	ORIENTALIS	CHINESE DWARF	NEWT	LIV	75	NO	SG	SG	T	C	C	C	9/8/13	9/8/13	I	LA	PET-SIAM INTERNATIONAL
2013337532	PACB	PACHYTRITON	BREVIPES	TSITOU	NEWT	LIV	60	NO	HK	HK	T	C	C	C	9/10/13	9/9/13	I	NY	Transship Discounts Ltd.
2013337696	TYVE	TYLOTOTRITON	VERRUCOSUS	CROCODILE	NEWT	LIV	6	NO	CN	CA	T	W	C	C	8/14/13	8/14/13	I	BL	STRICTLY REPTILES INC.
2013338011	CYOR	CYNOPS	ORIENTALIS	CHINESE DWARF	NEWT	LIV	1,200	NO	HK	HK	T	C	C	C	9/11/13	9/12/13	I	TP	5-D TROPICAL INC.
2013339838	CNP?	CYNOPS	SPECIES	FIREBELLY	NEWT	LIV	560	NO	CN	HK	T	W	C	C	9/15/13	9/15/13	I	LA	AQUA-NAUTIC SPECIALIST
2013339838	TRIT	TRITURUS	SPECIES		NEWT	LIV	45	NO	CN	HK	T	W	C	C	9/15/13	9/15/13	I	LA	AQUA-NAUTIC SPECIALIST
2013339844	CNEW	CYNOPS	CYANURUS	CYAN	NEWT	LIV	100	NO	HK	HK	T	C	C	C	9/15/13	9/15/13	I	LA	DOLPHIN INTERNATIONAL
2013339844	CYOR	CYNOPS	ORIENTALIS	CHINESE DWARF	NEWT	LIV	500	NO	HK	HK	T	C	C	C	9/15/13	9/15/13	I	LA	DOLPHIN INTERNATIONAL
2013339858	CYOR	CYNOPS	ORIENTALIS	CHINESE DWARF	NEWT	LIV	125	NO	SG	SG	T	C	C	C	9/15/13	9/15/13	I	LA	PET-SIAM INTERNATIONAL
2013340873	PACB	PACHYTRITON	BREVIPES	TSITOU	NEWT	LIV	30	NO	HK	HK	T	C	C	C	9/17/13	9/15/13	I	NY	Transship Discounts Ltd.
2013340873	CYPY	CYNOPS	PYRRHOGASTER	JAPANESE FIREBELLY	NEWT	LIV	315	NO	HK	HK	T	C	C	C	9/17/13	9/15/13	I	NY	Transship Discounts Ltd.
2013343412	CYOR	CYNOPS	ORIENTALIS	CHINESE DWARF	NEWT	LIV	250	NO	HK	HK	T	C	C	C	9/22/13	9/22/13	I	LA	DOLPHIN INTERNATIONAL
2013343412	PACB	PACHYTRITON	BREVIPES	TSITOU	NEWT	LIV	50	NO	HK	HK	T	C	C	C	9/22/13	9/22/13	I	LA	DOLPHIN INTERNATIONAL
2013343450	CYOR	CYNOPS	ORIENTALIS	CHINESE DWARF	NEWT	LIV	35	NO	SG	SG	T	C	C	C	9/22/13	9/22/13	I	LA	PET-SIAM INTERNATIONAL
2013347298	CYOR	CYNOPS	ORIENTALIS	CHINESE DWARF	NEWT	LIV	50	NO	HK	HK	T	C	C	C	9/29/13	9/29/13	I	LA	DOLPHIN INTERNATIONAL
2013347335	CYOR	CYNOPS	ORIENTALIS	CHINESE DWARF	NEWT	LIV	435	NO	SG	SG	T	C	C	C	9/29/13	9/29/13	I	LA	PET-SIAM INTERNATIONAL
2013347338	TRIT	TRITURUS	SPECIES		NEWT	LIV	45	NO	CN	HK	T	W	C	C	9/29/13	9/29/13	I	LA	AQUA-NAUTIC SPECIALIST
2013347338	CNP?	CYNOPS	SPECIES	FIREBELLY	NEWT	LIV	2,080	NO	CN	HK	T	W	C	C	9/29/13	9/29/13	I	LA	AQUA-NAUTIC SPECIALIST
2013347351	CYOR	CYNOPS	ORIENTALIS	CHINESE DWARF	NEWT	LIV	435	NO	SG	SG	T	C	C	C	9/29/13	9/29/13	I	LA	PET-SIAM INTERNATIONAL
2013348777	CYPY	CYNOPS	PYRRHOGASTER	JAPANESE FIREBELLY	NEWT	LIV	270	NO	HK	HK	T	C	C	C	10/2/13	9/29/13	I	NY	Transship Discounts Ltd.
2013348777	PACB	PACHYTRITON	BREVIPES	TSITOU	NEWT	LIV	30	NO	HK	HK	T	C	C	C	10/2/13	9/29/13	I	NY	Transship Discounts Ltd.
2013348791	CYPY	CYNOPS	PYRRHOGASTER	JAPANESE FIREBELLY	NEWT	LIV	105	NO	HK	HK	T	C	C	C	10/2/13	9/22/13	I	NY	Transship Discounts Ltd.
2013350989	CYOR	CYNOPS	ORIENTALIS	CHINESE DWARF	NEWT	LIV	475	NO	HK	HK	T	C	C	C	10/20/13	10/20/13	I	LA	DOLPHIN INTERNATIONAL
2013350989	PACB	PACHYTRITON	BREVIPES	TSITOU	NEWT	LIV	100	NO	HK	HK	T	C	C	C	10/20/13	10/20/13	I	LA	DOLPHIN INTERNATIONAL
2013351026	CYOR	CYNOPS	ORIENTALIS	CHINESE DWARF	NEWT	LIV	35	NO	SG	SG	T	C	C	C	10/20/13	10/20/13	I	LA	PET-SIAM INTERNATIONAL
2013351028	CNP?	CYNOPS	SPECIES	FIREBELLY	NEWT	LIV	140	NO	CN	HK	T	W	C	C	10/20/13	10/20/13	I	LA	AQUA-NAUTIC SPECIALIST
2013351098	CHI*	CHIROPTEROTRITON	SPECIES	SPLAYFOOT	SALAMANDER	LIV	15	NO	MX	MX	S	W	C	C	10/21/13	8/18/12	I	MI	Exemptions 6 and 7(C)
2013351098	PDE?	PSEUDOEURYCEA	SPECIES	FALSE BROOK	SALAMANDER	LIV	36	NO	MX	MX	S	W	C	C	10/21/13	8/18/12	I	MI	Exemptions 6 and 7(C)
2013351098	THO*	THORIUS	SPECIES	PIGMY	SALAMANDER	LIV	16	NO	MX	MX	S	W	C	C	10/21/13	8/18/12	I	MI	Exemptions 6 and 7(C)
2013353562	CYOR	CYNOPS	ORIENTALIS	CHINESE DWARF	NEWT	LIV	1,100	NO	HK	HK	T	C	C	C	10/23/13	10/24/13	I	TP	5-D TROPICAL INC.

2013354134	CNEW	CYNOPS	CYANURUS	CYAN	NEWT	LIV	60	NO	HK	HK	T	C	C	C	10/24/13	10/20/13	I	NY	Transship Discounts Ltd.
2013354134	CYPY	CYNOPS	PYRRHOGASTER	JAPANESE FIREBELLY	NEWT	LIV	105	NO	HK	HK	T	C	C	C	10/24/13	10/20/13	I	NY	Transship Discounts Ltd.
2013355485	PACB	PACHYTRITON	BREVIPES	TSITOU	NEWT	LIV	100	NO	HK	HK	T	C	C	C	10/27/13	10/27/13	I	LA	DOLPHIN INTERNATIONAL
2013355523	CNP?	CYNOPS	SPECIES	FIREBELLY	NEWT	LIV	440	NO	CN	HK	T	W	C	C	10/27/13	10/27/13	I	LA	AQUA-NAUTIC SPECIALIST
2013355523	TRIT	TRITURUS	SPECIES		NEWT	LIV	45	NO	CN	HK	T	W	C	C	10/27/13	10/27/13	I	LA	AQUA-NAUTIC SPECIALIST
2013355539	CYOR	CYNOPS	ORIENTALIS	CHINESE DWARF	NEWT	LIV	400	NO	SG	SG	T	C	C	C	10/27/13	10/27/13	I	LA	PET-SIAM INTERNATIONAL
2013357202	CYPY	CYNOPS	PYRRHOGASTER	JAPANESE FIREBELLY	NEWT	LIV	25	NO	HK	HK	T	C	C	C	10/29/13	10/27/13	I	NY	Transship Discounts Ltd.
2013357944	CYOR	CYNOPS	ORIENTALIS	CHINESE DWARF	NEWT	LIV	3,200	NO	HK	HK	T	C	C	C	10/30/13	10/31/13	I	TP	5-D TROPICAL INC.
2013358484	SASA	SALAMANDRA	SALAMANDRA	EUROPEAN FIRE	SALAMANDER	LIV	750	NO	SI	SI	T	C	C	C	10/31/13	10/31/13	I	MI	TWO AMIGOS IMPORTS, INC.
2013359890	CYOR	CYNOPS	ORIENTALIS	CHINESE DWARF	NEWT	LIV	275	NO	HK	HK	T	C	C	C	11/3/13	11/3/13	I	LA	DOLPHIN INTERNATIONAL
2013359921	CNP?	CYNOPS	SPECIES	FIREBELLY	NEWT	LIV	900	NO	CN	HK	T	W	C	C	11/3/13	11/3/13	I	LA	AQUA-NAUTIC SPECIALIST
2013362504	PACB	PACHYTRITON	BREVIPES	TSITOU	NEWT	LIV	30	NO	HK	HK	T	C	C	C	11/6/13	11/3/13	I	NY	Transship Discounts Ltd.
2013364705	CYOR	CYNOPS	ORIENTALIS	CHINESE DWARF	NEWT	LIV	500	NO	HK	HK	T	C	C	C	11/10/13	11/10/13	I	LA	DOLPHIN INTERNATIONAL
2013364758	CYOR	CYNOPS	ORIENTALIS	CHINESE DWARF	NEWT	LIV	110	NO	SG	SG	T	C	C	C	11/10/13	11/10/13	I	LA	PET-SIAM INTERNATIONAL
2013364778	CNP?	CYNOPS	SPECIES	FIREBELLY	NEWT	LIV	140	NO	CN	HK	T	W	C	C	11/10/13	11/10/13	I	LA	AQUA-NAUTIC SPECIALIST
2013364778	TRIT	TRITURUS	SPECIES		NEWT	LIV	45	NO	CN	HK	T	W	C	C	11/10/13	11/10/13	I	LA	AQUA-NAUTIC SPECIALIST
2013369088	CNEW	CYNOPS	CYANURUS	CYAN	NEWT	LIV	30	NO	HK	HK	T	C	C	C	11/15/13	11/10/13	I	NY	Transship Discounts Ltd.
2013369264	CNEW	CYNOPS	CYANURUS	CYAN	NEWT	LIV	50	NO	HK	HK	T	C	C	C	11/17/13	11/17/13	I	LA	DOLPHIN INTERNATIONAL
2013369264	CYOR	CYNOPS	ORIENTALIS	CHINESE DWARF	NEWT	LIV	425	NO	HK	HK	T	C	C	C	11/17/13	11/17/13	I	LA	DOLPHIN INTERNATIONAL
2013369264	PACB	PACHYTRITON	BREVIPES	TSITOU	NEWT	LIV	50	NO	HK	HK	T	C	C	C	11/17/13	11/17/13	I	LA	DOLPHIN INTERNATIONAL
2013371285	SASA	SALAMANDRA	SALAMANDRA	EUROPEAN FIRE	SALAMANDER	LIV	750	NO	SI	SI	T	C	C	C	11/20/13	11/19/13	I	MI	ALL PRO SHIPPING INC.
2013372048	CNEW	CYNOPS	CYANURUS	CYAN	NEWT	LIV	420	NO	HK	HK	T	C	C	C	11/20/13	11/17/13	I	NY	Transship Discounts Ltd.
2013372048	PACB	PACHYTRITON	BREVIPES	TSITOU	NEWT	LIV	60	NO	HK	HK	T	C	C	C	11/20/13	11/17/13	I	NY	Transship Discounts Ltd.
2013372406	CYOR	CYNOPS	ORIENTALIS	CHINESE DWARF	NEWT	LIV	25	NO	SG	SG	T	C	C	C	10/6/13	10/6/13	I	LA	PET-SIAM INTERNATIONAL
2013374099	TRIT	TRITURUS	SPECIES		NEWT	LIV	45	NO	CN	HK	T	W	C	C	11/24/13	11/24/13	I	LA	AQUA-NAUTIC SPECIALIST
2013374099	CNP?	CYNOPS	SPECIES	FIREBELLY	NEWT	LIV	140	NO	CN	HK	T	W	C	C	11/24/13	11/24/13	I	LA	AQUA-NAUTIC SPECIALIST
2013374901	CNP?	CYNOPS	SPECIES	FIREBELLY	NEWT	LIV	1,740	NO	CN	HK	T	C	C	C	10/6/13	10/6/13	I	LA	AQUA-NAUTIC SPECIALIST
2013374938	CNEW	CYNOPS	CYANURUS	CYAN	NEWT	LIV	30	NO	HK	HK	T	C	C	C	11/25/13	11/24/13	I	NY	Transship Discounts Ltd.
2013375767	CYOR	CYNOPS	ORIENTALIS	CHINESE DWARF	NEWT	LIV	625	NO	HK	HK	T	C	C	C	10/6/13	10/6/13	I	LA	DOLPHIN INTERNATIONAL
2013375767	PACB	PACHYTRITON	BREVIPES	TSITOU	NEWT	LIV	150	NO	HK	HK	T	C	C	C	10/6/13	10/6/13	I	LA	DOLPHIN INTERNATIONAL
2013377655	CYOR	CYNOPS	ORIENTALIS	CHINESE DWARF	NEWT	LIV	375	NO	HK	HK	T	C	C	C	12/1/13	12/1/13	I	LA	DOLPHIN INTERNATIONAL
2013377655	PACB	PACHYTRITON	BREVIPES	TSITOU	NEWT	LIV	50	NO	HK	HK	T	C	C	C	12/1/13	12/1/13	I	LA	DOLPHIN INTERNATIONAL
2013377704	CNP?	CYNOPS	SPECIES	FIREBELLY	NEWT	LIV	440	NO	CN	HK	T	W	C	C	12/1/13	12/1/13	I	LA	AQUA-NAUTIC SPECIALIST
2013377704	TRIT	TRITURUS	SPECIES		NEWT	LIV	100	NO	CN	HK	T	W	C	C	12/1/13	12/1/13	I	LA	AQUA-NAUTIC SPECIALIST
2013378164	CYPY	CYNOPS	PYRRHOGASTER	JAPANESE FIREBELLY	NEWT	LIV	135	NO	HK	HK	T	C	C	C	12/2/13	12/1/13	I	NY	Transship Discounts Ltd.
2013382118	CYOR	CYNOPS	ORIENTALIS	CHINESE DWARF	NEWT	LIV	350	NO	HK	HK	T	C	C	C	12/8/13	12/8/13	I	LA	DOLPHIN INTERNATIONAL
2013382169	CYOR	CYNOPS	ORIENTALIS	CHINESE DWARF	NEWT	LIV	325	NO	SG	SG	T	C	C	C	12/8/13	12/8/13	I	LA	PET-SIAM INTERNATIONAL
2013382189	TRIT	TRITURUS	SPECIES		NEWT	LIV	80	NO	CN	HK	T	W	C	C	12/8/13	12/8/13	I	LA	AQUA-NAUTIC SPECIALIST
2013382189	CNP?	CYNOPS	SPECIES	FIREBELLY	NEWT	LIV	2,980	NO	CN	HK	T	W	C	C	12/8/13	12/8/13	I	LA	AQUA-NAUTIC SPECIALIST
2013382231	CYOR	CYNOPS	ORIENTALIS	CHINESE DWARF	NEWT	LIV	425	NO	HK	HK	T	C	C	C	10/13/13	10/13/13	I	LA	DOLPHIN INTERNATIONAL
2013383038	CYPY	CYNOPS	PYRRHOGASTER	JAPANESE FIREBELLY	NEWT	LIV	165	NO	HK	HK	T	C	C	C	12/9/13	12/8/13	I	NY	Transship Discounts Ltd.
2013383038	PACB	PACHYTRITON	BREVIPES	TSITOU	NEWT	LIV	30	NO	HK	HK	T	C	C	C	12/9/13	12/8/13	I	NY	Transship Discounts Ltd.
2013387098	AMBM	AMBYSTOMA	MEXICANUM		AXOLOTL	LIV	100	NO	DE	DE	S	C	C	C	12/13/13	12/11/13	I	SF	UNIVERSITY OF CALIFORNIA, SAN FRANCISCO
2013387539	CYOR	CYNOPS	ORIENTALIS	CHINESE DWARF	NEWT	LIV	875	NO	HK	HK	T	C	C	C	12/15/13	12/15/13	I	LA	DOLPHIN INTERNATIONAL
2013387589	CYOR	CYNOPS	ORIENTALIS	CHINESE DWARF	NEWT	LIV	290	NO	CN	HK	T	W	C	C	12/15/13	12/15/13	I	LA	AQUA-NAUTIC SPECIALIST
2013388320	PACB	PACHYTRITON	BREVIPES	TSITOU	NEWT	LIV	30	NO	HK	HK	T	C	C	C	12/16/13	12/15/13	I	NY	Transship Discounts Ltd.
2013388320	CYPY	CYNOPS	PYRRHOGASTER	JAPANESE FIREBELLY	NEWT	LIV	105	NO	HK	HK	T	C	C	C	12/16/13	12/15/13	I	NY	Transship Discounts Ltd.
2013392290	AMOP	AMBYSTOMA	OPACUM	MARBLED	SALAMANDER	LIV	3	NO	US	CA	P	W	C	C	2/8/13	2/8/13	I	DS	Exemptions 6 and 7(C)
2013392290	AMBY	AMBYSTOMA	SPECIES	MOLE	SALAMANDER	LIV	4	NO	US	CA	P	W	C	C	2/8/13	2/8/13	I	DS	Exemptions 6 and 7(C)
2013392290	AMBY	AMBYSTOMA	SPECIES	MOLE	SALAMANDER	LIV	1	NO	US	CA	P	W	C	C	2/8/13	2/8/13	I	DS	Exemptions 6 and 7(C)
2013392290	PLET	PLETHODON	SPECIES		SALAMANDER	LIV	7	NO	US	CA	P	W	C	C	2/8/13	2/8/13	I	DS	Exemptions 6 and 7(C)
2013392290	PLET	PLETHODON	SPECIES		SALAMANDER	LIV	5	NO	US	CA	P	W	C	C	2/8/13	2/8/13	I	DS	Exemptions 6 and 7(C)
2013392290	PLET	PLETHODON	SPECIES		SALAMANDER	LIV	1	NO	US	CA	P	W	C	C	2/8/13	2/8/13	I	DS	Exemptions 6 and 7(C)
2013392290	EUR?	EURYCEA	SPECIES		SALAMANDER	LIV	5	NO	US	CA	P	W	C	C	2/8/13	2/8/13	I	DS	Exemptions 6 and 7(C)
2013392290	DSM?	DESMOGNATHUS	SPECIES	DUSKY	SALAMANDER	LIV	1	NO	US	CA	P	W	C	C	12/8/13	2/8/13	I	DS	Exemptions 6 and 7(C)
2013392290	HAMJ	HYNOBIUS	AMJIENSIS	AMJI'S	SALAMANDER	LIV	1	NO	JP	CA	P	W	C	C	12/8/13	2/8/13	I	DS	Exemptions 6 and 7(C)
2013392290	CYPY	CYNOPS	PYRRHOGASTER	JAPANESE FIREBELLY	NEWT	LIV	1	NO	JP	CA	P	W	C	C	12/8/13	2/8/13	I	DS	Exemptions 6 and 7(C)
2013392290	CNP?	CYNOPS	SPECIES	FIREBELLY	NEWT	LIV	1	NO	JP	CA	P	W	C	C	12/8/13	2/8/13	I	DS	Exemptions 6 and 7(C)
2013392290	CNP?	CYNOPS	SPECIES	FIREBELLY	NEWT	LIV	1	NO	JP	CA	P	W	C	C	12/8/13	2/8/13	I	DS	Exemptions 6 and 7(C)

2013392290	CNP?	CYNOPS	SPECIES	FIREBELLY	NEWT	LIV	4	NO	JP	CA	P	W	C	C	12/8/13	2/8/13	I	DS	Exemptions 6 and 7(C)
2013392290	WNEW	PARAMESOTRITON	SPECIES	WARTY	NEWT	LIV	1	NO	JP	CA	P	W	C	C	12/8/13	2/8/13	I	DS	Exemptions 6 and 7(C)
2013392290	NEW?	PACHYTRITON	SPECIES	CHINESE	NEWT	LIV	3	NO	JP	CA	P	W	C	C	12/8/13	2/8/13	I	DS	Exemptions 6 and 7(C)
2013392290	NEW?	PACHYTRITON	SPECIES	CHINESE	NEWT	LIV	1	NO	JP	CA	P	W	C	C	12/8/13	2/8/13	I	DS	Exemptions 6 and 7(C)
2013392290	TYL?	TYLOTOTRITON	SPECIES	CROCODILE	NEWT	LIV	1	NO	JP	CA	P	W	C	C	12/8/13	2/8/13	I	DS	Exemptions 6 and 7(C)
2013392290	NOVI	NOTOPHTHALMUS	VIRIDESCENS	EASTERN	NEWT	LIV	1	NO	JP	CA	P	W	C	C	12/8/13	2/8/13	I	DS	Exemptions 6 and 7(C)
2013392290	ENES	ENSATINA	ESCHSCHOLTZII		ENSATINA	LIV	3	NO	JP	CA	P	W	C	C	12/8/13	2/8/13	I	DS	Exemptions 6 and 7(C)
2013394865	CYOR	CYNOPS	ORIENTALIS	CHINESE DWARF	NEWT	LIV	375	NO	HK	HK	T	C	C	C	12/28/13	12/28/13	I	LA	DOLPHIN INTERNATIONAL
2013394865	PACB	PACHYTRITON	BREVIPES	TSITOU	NEWT	LIV	100	NO	HK	HK	T	C	C	C	12/28/13	12/28/13	I	LA	DOLPHIN INTERNATIONAL
2013394886	CNP?	CYNOPS	SPECIES	FIREBELLY	NEWT	LIV	140	NO	CN	HK	T	W	C	C	12/28/13	12/28/13	I	LA	AQUA-NAUTIC SPECIALIST
2013394935	CYOR	CYNOPS	ORIENTALIS	CHINESE DWARF	NEWT	LIV	75	NO	SG	SG	T	C	C	C	12/29/13	12/29/13	I	LA	PET-SIAM INTERNATIONAL
2014396899	CNP?	CYNOPS	SPECIES	FIREBELLY	NEWT	LIV	1	NO	XX	CN	P	U	R	S	12/30/13	12/30/13	I	DF	Exemptions 6 and 7(C)
2014397102	CYOR	CYNOPS	ORIENTALIS	CHINESE DWARF	NEWT	LIV	175	NO	HK	HK	T	C	C	C	1/5/14	1/5/14	I	LA	DOLPHIN INTERNATIONAL
2014397130	CYOR	CYNOPS	ORIENTALIS	CHINESE DWARF	NEWT	LIV	110	NO	SG	SG	T	C	C	C	1/5/14	1/5/14	I	LA	PET-SIAM INTERNATIONAL
2014397134	CNP?	CYNOPS	SPECIES	FIREBELLY	NEWT	LIV	140	NO	CN	HK	T	W	C	C	1/5/14	1/5/14	I	LA	AQUA-NAUTIC SPECIALIST
2014398907	CYOR	CYNOPS	ORIENTALIS	CHINESE DWARF	NEWT	LIV	1,600	NO	HK	HK	T	C	C	C	1/8/14	1/9/14	I	TP	5-D TROPICAL INC.
2014400690	CYOR	CYNOPS	ORIENTALIS	CHINESE DWARF	NEWT	LIV	35	NO	SG	SG	T	C	C	C	1/12/14	1/12/14	I	LA	PET-SIAM INTERNATIONAL
2014400905	CNEW	CYNOPS	CYANURUS	CYAN	NEWT	LIV	30	NO	HK	HK	T	C	C	C	1/13/14	1/5/14	I	NY	Transship Discounts Ltd.
2014400905	CYPY	CYNOPS	PYRRHOGASTER	JAPANESE FIREBELLY	NEWT	LIV	420	NO	HK	HK	T	C	C	C	1/13/14	1/5/14	I	NY	Transship Discounts Ltd.
2014402932	CNEW	CYNOPS	CYANURUS	CYAN	NEWT	LIV	29	NO	HK	HK	T	C	C	C	1/15/14	1/12/14	I	NY	Transship Discounts Ltd.
2014404934	CYOR	CYNOPS	ORIENTALIS	CHINESE DWARF	NEWT	LIV	200	NO	HK	HK	T	C	C	C	1/19/14	1/19/14	I	LA	DOLPHIN INTERNATIONAL
2014404934	PACB	PACHYTRITON	BREVIPES	TSITOU	NEWT	LIV	100	NO	HK	HK	T	C	C	C	1/19/14	1/19/14	I	LA	DOLPHIN INTERNATIONAL
2014404978	CYOR	CYNOPS	ORIENTALIS	CHINESE DWARF	NEWT	LIV	400	NO	SG	SG	T	C	C	C	1/19/14	1/19/14	I	LA	PET-SIAM INTERNATIONAL
2014410922	PCHI	PARAMESOTRITON	CHINENSIS	CHINESE WARTY	NEWT	LIV	5	NO	HK	HK	T	C	C	C	10/9/13	10/9/13	I	MI	NAME REMOVED
2014411187	TYL?	TYLOTOTRITON	SPECIES	CROCODILE	NEWT	LIV	31	NO	DE	DE	T	C	C	C	10/3/13	10/3/13	I	MI	TWO AMIGOS IMPORT & EXPORT INC
2014411737	CNEW	CYNOPS	CYANURUS	CYAN	NEWT	LIV	30	NO	HK	HK	T	C	C	C	1/30/14	1/26/14	I	NY	Transship Discounts Ltd.
2014411737	PACB	PACHYTRITON	BREVIPES	TSITOU	NEWT	LIV	60	NO	HK	HK	T	C	C	C	1/30/14	1/26/14	I	NY	Transship Discounts Ltd.
2014413093	PACB	PACHYTRITON	BREVIPES	TSITOU	NEWT	LIV	100	NO	HK	HK	T	C	C	C	2/2/14	2/2/14	I	LA	DOLPHIN INTERNATIONAL
2014415831	CYPY	CYNOPS	PYRRHOGASTER	JAPANESE FIREBELLY	NEWT	LIV	30	NO	HK	HK	T	C	C	C	10/11/13	10/6/13	I	NY	TRANSSHIP DISCOUNTS LIMITED
2014415831	NEW?	PACHYTRITON	SPECIES	CHINESE	NEWT	LIV	60	NO	HK	HK	T	C	C	C	10/11/13	10/6/13	I	NY	TRANSSHIP DISCOUNTS LIMITED
2014416304	CYOR	CYNOPS	ORIENTALIS	CHINESE DWARF	NEWT	LIV	150	NO	SG	SG	T	C	C	C	2/9/14	2/9/14	I	LA	PET-SIAM INTERNATIONAL
2014416337	CNEW	CYNOPS	CYANURUS	CYAN	NEWT	LIV	50	NO	HK	HK	T	C	C	C	2/9/14	2/9/14	I	LA	DOLPHIN INTERNATIONAL
2014416337	PACB	PACHYTRITON	BREVIPES	TSITOU	NEWT	LIV	100	NO	HK	HK	T	C	C	C	2/9/14	2/9/14	I	LA	DOLPHIN INTERNATIONAL
2014416910	CNEW	CYNOPS	CYANURUS	CYAN	NEWT	LIV	30	NO	HK	HK	T	W	C	C	2/10/14	2/9/14	I	NY	Transship Discounts Ltd.
2014416910	PACB	PACHYTRITON	BREVIPES	TSITOU	NEWT	LIV	30	NO	HK	HK	T	W	C	C	2/10/14	2/9/14	I	NY	Transship Discounts Ltd.
2014419624	CYOR	CYNOPS	ORIENTALIS	CHINESE DWARF	NEWT	LIV	50	NO	DE	DE	T	C	C	C	2/14/14	2/13/14	I	MI	TWO AMIGOS IMPORTS, INC.
2014423492	TRIT	TRITURUS	SPECIES		NEWT	LIV	120	NO	CN	HK	T	W	C	C	2/23/14	2/23/14	I	LA	AQUA-NAUTIC SPECIALIST
2014427066	CYOR	CYNOPS	ORIENTALIS	CHINESE DWARF	NEWT	LIV	500	NO	HK	HK	T	C	C	C	3/2/14	3/2/14	I	LA	DOLPHIN INTERNATIONAL
2014427066	PACB	PACHYTRITON	BREVIPES	TSITOU	NEWT	LIV	100	NO	HK	HK	T	C	C	C	3/2/14	3/2/14	I	LA	DOLPHIN INTERNATIONAL
2014427087	CNP?	CYNOPS	SPECIES	FIREBELLY	NEWT	LIV	1,780	NO	HK	HK	T	W	C	C	3/2/14	3/2/14	I	LA	AQUA-NAUTIC SPECIALIST
2014427087	TRIT	TRITURUS	SPECIES		NEWT	LIV	100	NO	HK	HK	T	W	C	C	3/2/14	3/2/14	I	LA	AQUA-NAUTIC SPECIALIST
2014427094	CYOR	CYNOPS	ORIENTALIS	CHINESE DWARF	NEWT	LIV	100	NO	SG	SG	T	C	C	C	3/2/14	3/2/14	I	LA	PET-SIAM INTERNATIONAL
2014429210	CNP?	CYNOPS	SPECIES	FIREBELLY	NEWT	LIV	500	NO	HK	HK	T	C	C	C	3/6/14	3/6/14	I	AT	SUN PET LTD.
2014430871	CYOR	CYNOPS	ORIENTALIS	CHINESE DWARF	NEWT	LIV	875	NO	HK	HK	T	C	C	C	3/9/14	3/9/14	I	LA	DOLPHIN INTERNATIONAL
2014430887	CNP?	CYNOPS	SPECIES	FIREBELLY	NEWT	LIV	2,300	NO	CN	HK	T	W	C	C	3/9/14	3/9/14	I	LA	AQUA-NAUTIC SPECIALIST
2014430887	TRIT	TRITURUS	SPECIES		NEWT	LIV	40	NO	CN	HK	T	W	C	C	3/9/14	3/9/14	I	LA	AQUA-NAUTIC SPECIALIST
2014430896	CYOR	CYNOPS	ORIENTALIS	CHINESE DWARF	NEWT	LIV	25	NO	SG	SG	T	C	C	C	3/9/14	3/9/14	I	LA	PET-SIAM INTERNATIONAL
2014431065	CYPY	CYNOPS	PYRRHOGASTER	JAPANESE FIREBELLY	NEWT	LIV	210	NO	HK	HK	T	C	C	C	3/10/14	3/9/14	I	NY	Transship Discounts Ltd.
2014431374	CYOR	CYNOPS	ORIENTALIS	CHINESE DWARF	NEWT	LIV	120	NO	SG	SG	T	C	C	C	3/10/14	3/9/14	I	NY	EMARK TROPICAL IMPORTS, INC.
2014432882	CYOR	CYNOPS	ORIENTALIS	CHINESE DWARF	NEWT	LIV	1,600	NO	HK	HK	T	C	C	C	3/12/14	3/13/14	I	TP	5-D TROPICAL INC.
2014434578	CYOR	CYNOPS	ORIENTALIS	CHINESE DWARF	NEWT	LIV	300	NO	HK	HK	T	C	C	C	3/16/14	3/16/14	I	LA	DOLPHIN INTERNATIONAL
2014434627	CYOR	CYNOPS	ORIENTALIS	CHINESE DWARF	NEWT	LIV	145	NO	SG	SG	T	C	C	C	3/16/14	3/16/14	I	LA	PET-SIAM INTERNATIONAL
2014434640	CNP?	CYNOPS	SPECIES	FIREBELLY	NEWT	LIV	2,680	NO	CN	HK	T	W	C	C	3/16/14	3/16/14	I	LA	AQUA-NAUTIC SPECIALIST
2014434809	CYPY	CYNOPS	PYRRHOGASTER	JAPANESE FIREBELLY	NEWT	LIV	420	NO	HK	HK	T	C	C	C	3/17/14	3/16/14	I	NY	Transship Discounts Ltd.
2014436345	CYOR	CYNOPS	ORIENTALIS	CHINESE DWARF	NEWT	LIV	1,600	NO	HK	HK	T	C	C	C	3/19/14	3/20/14	I	TP	5-D TROPICAL INC.
2014438269	CYOR	CYNOPS	ORIENTALIS	CHINESE DWARF	NEWT	LIV	250	NO	HK	HK	T	C	C	C	3/23/14	3/23/14	I	LA	DOLPHIN INTERNATIONAL
2014438269	PACB	PACHYTRITON	BREVIPES	TSITOU	NEWT	LIV	100	NO	HK	HK	T	C	C	C	3/23/14	3/23/14	I	LA	DOLPHIN INTERNATIONAL
2014438301	CYOR	CYNOPS	ORIENTALIS	CHINESE DWARF	NEWT	LIV	510	NO	SG	SG	T	C	C	C	3/23/14	3/23/14	I	LA	PET-SIAM INTERNATIONAL
2014439727	CNP?	CYNOPS	SPECIES	FIREBELLY	NEWT	LIV	880	NO	CN	HK	T	W	C	C	3/25/14	3/23/14	I	LA	AQUA-NAUTIC SPECIALIST
2014439727	TRIT	TRITURUS	SPECIES		NEWT	LIV	360	NO	CN	HK	T	W	C	C	3/25/14	3/23/14	I	LA	AQUA-NAUTIC SPECIALIST

2014440218	CYOR	CYNOPS	ORIENTALIS	CHINESE DWARF	NEWT	LIV	1,500	NO	HK	HK	T	C	C	C	3/26/14	3/27/14	I	TP	5-D TROPICAL INC.
2014441879	CYOR	CYNOPS	ORIENTALIS	CHINESE DWARF	NEWT	LIV	75	NO	SG	SG	T	C	C	C	3/30/14	3/30/14	I	LA	PET-SIAM INTERNATIONAL
2014441894	CNP?	CYNOPS	SPECIES	FIREBELLY	NEWT	LIV	440	NO	CN	HK	T	W	C	C	3/30/14	3/30/14	I	LA	AQUA-NAUTIC SPECIALIST
2014441905	CYOR	CYNOPS	ORIENTALIS	CHINESE DWARF	NEWT	LIV	400	NO	HK	HK	T	C	C	C	3/30/14	3/30/14	I	LA	DOLPHIN INTERNATIONAL
2014441905	PACB	PACHYTRITON	BREVIPES	TSITOU	NEWT	LIV	100	NO	HK	HK	T	C	C	C	3/30/14	3/30/14	I	LA	DOLPHIN INTERNATIONAL
2014442786	CYPY	CYNOPS	PYRRHOGASTER	JAPANESE FIREBELLY	NEWT	LIV	270	NO	HK	HK	T	C	C	C	10/16/13	10/13/13	I	NY	TRANSSHIP DISCOUNTS LIMITED
2014442786	PACB	PACHYTRITON	BREVIPES	TSITOU	NEWT	LIV	60	NO	HK	HK	T	C	C	C	10/16/13	10/13/13	I	NY	TRANSSHIP DISCOUNTS LIMITED
2014442786	CNEW	CYNOPS	CYANURUS	CYAN	NEWT	LIV	30	NO	HK	HK	T	C	C	C	10/16/13	10/13/13	I	NY	TRANSSHIP DISCOUNTS LIMITED
2014442880	CYPY	CYNOPS	PYRRHOGASTER	JAPANESE FIREBELLY	NEWT	LIV	435	NO	HK	HK	T	C	C	C	4/1/14	3/30/14	I	NY	Transship Discounts Ltd.
2014442880	PACB	PACHYTRITON	BREVIPES	TSITOU	NEWT	LIV	60	NO	HK	HK	T	C	C	C	4/1/14	3/30/14	I	NY	Transship Discounts Ltd.
2014444267	CYOR	CYNOPS	ORIENTALIS	CHINESE DWARF	NEWT	LIV	1,600	NO	HK	HK	T	C	C	C	4/2/14	4/3/14	I	TP	5-D TROPICAL INC.
2014445548	CNP?	CYNOPS	SPECIES	FIREBELLY	NEWT	LIV	2,520	NO	CN	HK	T	W	C	C	10/13/13	10/13/13	I	LA	AQUA-NAUTIC SPECIALIST
2014445548	TRIT	TRITURUS	SPECIES		NEWT	LIV	45	NO	CN	HK	T	W	C	C	10/13/13	10/13/13	I	LA	AQUA-NAUTIC SPECIALIST
2014446129	CNEW	CYNOPS	CYANURUS	CYAN	NEWT	LIV	100	NO	HK	HK	T	C	C	C	4/6/14	4/6/14	I	LA	DOLPHIN INTERNATIONAL
2014446129	CYOR	CYNOPS	ORIENTALIS	CHINESE DWARF	NEWT	LIV	125	NO	HK	HK	T	C	C	C	4/6/14	4/6/14	I	LA	DOLPHIN INTERNATIONAL
2014446196	CNP?	CYNOPS	SPECIES	FIREBELLY	NEWT	LIV	2,080	NO	CN	HK	T	W	C	C	4/6/14	4/6/14	I	LA	AQUA-NAUTIC SPECIALIST
2014446196	TRIT	TRITURUS	SPECIES		NEWT	LIV	120	NO	CN	HK	T	W	C	C	4/6/14	4/6/14	I	LA	AQUA-NAUTIC SPECIALIST
2014446917	PACB	PACHYTRITON	BREVIPES	TSITOU	NEWT	LIV	60	NO	HK	HK	T	C	C	C	4/7/14	4/6/14	I	NY	Transship Discounts Ltd.
2014447997	CYOR	CYNOPS	ORIENTALIS	CHINESE DWARF	NEWT	LIV	1,600	NO	HK	HK	T	C	C	C	4/9/14	4/10/14	I	TP	5-D TROPICAL INC.
2014448315	CNP?	CYNOPS	SPECIES	FIREBELLY	NEWT	LIV	500	NO	HK	HK	T	C	C	C	4/10/14	4/10/14	I	AT	SUN PET LTD.
2014449526	CYOR	CYNOPS	ORIENTALIS	CHINESE DWARF	NEWT	LIV	400	NO	HK	HK	T	C	C	C	4/13/14	4/13/14	I	LA	DOLPHIN INTERNATIONAL
2014449526	PACB	PACHYTRITON	BREVIPES	TSITOU	NEWT	LIV	250	NO	HK	HK	T	C	C	C	4/13/14	4/13/14	I	LA	DOLPHIN INTERNATIONAL
2014449562	PLAB	PACHYTRITON	LABIATUS	UNTERSTEIN'S	NEWT	LIV	40	NO	CN	HK	T	W	C	C	4/13/14	4/13/14	I	LA	AQUA-NAUTIC SPECIALIST
2014449562	CNP?	CYNOPS	SPECIES	FIREBELLY	NEWT	LIV	880	NO	CN	HK	T	W	C	C	4/13/14	4/13/14	I	LA	AQUA-NAUTIC SPECIALIST
2014449652	CYPY	CYNOPS	PYRRHOGASTER	JAPANESE FIREBELLY	NEWT	LIV	345	NO	HK	HK	T	C	C	C	4/14/14	4/13/14	I	NY	Transship Discounts Ltd.
2014449652	PACB	PACHYTRITON	BREVIPES	TSITOU	NEWT	LIV	60	NO	HK	HK	T	C	C	C	4/14/14	4/13/14	I	NY	Transship Discounts Ltd.
2014452913	CYOR	CYNOPS	ORIENTALIS	CHINESE DWARF	NEWT	LIV	125	NO	HK	HK	T	C	C	C	4/20/14	4/20/14	I	LA	DOLPHIN INTERNATIONAL
2014452913	PACB	PACHYTRITON	BREVIPES	TSITOU	NEWT	LIV	90	NO	HK	HK	T	C	C	C	4/20/14	4/20/14	I	LA	DOLPHIN INTERNATIONAL
2014452956	CYOR	CYNOPS	ORIENTALIS	CHINESE DWARF	NEWT	LIV	100	NO	SG	SG	T	C	C	C	4/20/14	4/20/14	I	LA	PET-SIAM INTERNATIONAL
2014452972	CNP?	CYNOPS	SPECIES	FIREBELLY	NEWT	LIV	580	NO	CN	HK	T	W	C	C	4/20/14	4/20/14	I	LA	AQUA-NAUTIC SPECIALIST
2014452972	TRIT	TRITURUS	SPECIES		NEWT	LIV	120	NO	CN	HK	T	W	C	C	4/20/14	4/20/14	I	LA	AQUA-NAUTIC SPECIALIST
2014453052	CYPY	CYNOPS	PYRRHOGASTER	JAPANESE FIREBELLY	NEWT	LIV	105	NO	HK	HK	T	C	C	C	4/21/14	4/20/14	I	NY	Transship Discounts Ltd.
2014453052	PACB	PACHYTRITON	BREVIPES	TSITOU	NEWT	LIV	8	NO	HK	HK	T	C	C	C	4/21/14	4/20/14	I	NY	Transship Discounts Ltd.
2014454834	CYOR	CYNOPS	ORIENTALIS	CHINESE DWARF	NEWT	LIV	1,600	NO	HK	HK	T	C	C	C	4/23/14	4/24/14	I	TP	5-D TROPICAL, INC.
2014456450	PACB	PACHYTRITON	BREVIPES	TSITOU	NEWT	LIV	50	NO	HK	HK	T	C	C	C	4/27/14	4/27/14	I	LA	DOLPHIN INTERNATIONAL
2014456473	CNP?	CYNOPS	SPECIES	FIREBELLY	NEWT	LIV	880	NO	CN	HK	T	W	C	C	4/27/14	4/27/14	I	LA	AQUA-NAUTIC SPECIALIST
2014456473	TRIT	TRITURUS	SPECIES		NEWT	LIV	40	NO	CN	HK	T	W	C	C	4/27/14	4/27/14	I	LA	AQUA-NAUTIC SPECIALIST
2014458302	CYPY	CYNOPS	PYRRHOGASTER	JAPANESE FIREBELLY	NEWT	LIV	60	NO	HK	HK	T	C	C	C	4/30/14	4/27/14	I	NY	Transship Discounts Ltd.
2014458302	PACB	PACHYTRITON	BREVIPES	TSITOU	NEWT	LIV	30	NO	HK	HK	T	C	C	C	4/30/14	4/27/14	I	NY	Transship Discounts Ltd.
2014460080	CYOR	CYNOPS	ORIENTALIS	CHINESE DWARF	NEWT	LIV	325	NO	SG	SG	T	C	C	C	5/4/14	5/4/14	I	LA	PET-SIAM INTERNATIONAL
2014460087	CYOR	CYNOPS	ORIENTALIS	CHINESE DWARF	NEWT	LIV	50	NO	HK	HK	T	C	C	C	5/4/14	5/4/14	I	LA	DOLPHIN INTERNATIONAL
2014460087	PACB	PACHYTRITON	BREVIPES	TSITOU	NEWT	LIV	100	NO	HK	HK	T	C	C	C	5/4/14	5/4/14	I	LA	DOLPHIN INTERNATIONAL
2014460118	CNP?	CYNOPS	SPECIES	FIREBELLY	NEWT	LIV	2,520	NO	CN	HK	T	W	C	C	5/4/14	5/4/14	I	LA	AQUA-NAUTIC SPECIALIST
2014460118	TRIT	TRITURUS	SPECIES		NEWT	LIV	40	NO	CN	HK	T	W	C	C	5/4/14	5/4/14	I	LA	AQUA-NAUTIC SPECIALIST
2014460210	CYPY	CYNOPS	PYRRHOGASTER	JAPANESE FIREBELLY	NEWT	LIV	210	NO	HK	HK	T	C	C	C	5/5/14	5/4/14	I	NY	Transship Discounts Ltd.
2014462228	CYOR	CYNOPS	ORIENTALIS	CHINESE DWARF	NEWT	LIV	1,600	NO	HK	HK	T	C	C	C	5/7/14	5/8/14	I	TP	5-D TROPICAL, INC.
2014463163	SAL?	SALAMANDRA	SPECIES	FIRE	SALAMANDER	LIV	24	NO	DE	DE	T	C	C	C	5/8/14	5/8/14	I	SF	Exemptions 6 and 7(C)
2014463163	SAL?	SALAMANDRA	SPECIES	FIRE	SALAMANDER	LIV	28	NO	DE	DE	T	C	C	C	5/8/14	5/8/14	I	SF	Exemptions 6 and 7(C)
2014463820	CYOR	CYNOPS	ORIENTALIS	CHINESE DWARF	NEWT	LIV	725	NO	HK	HK	T	C	C	C	5/11/14	5/11/14	I	LA	DOLPHIN INTERNATIONAL
2014463820	PACB	PACHYTRITON	BREVIPES	TSITOU	NEWT	LIV	50	NO	HK	HK	T	C	C	C	5/11/14	5/11/14	I	LA	DOLPHIN INTERNATIONAL
2014463880	CNP?	CYNOPS	SPECIES	FIREBELLY	NEWT	LIV	1,480	NO	CN	HK	T	W	C	C	5/11/14	5/11/14	I	LA	AQUA-NAUTIC SPECIALIST
2014463880	TRIT	TRITURUS	SPECIES		NEWT	LIV	40	NO	CN	HK	T	W	C	C	5/11/14	5/11/14	I	LA	AQUA-NAUTIC SPECIALIST
2014464411	CYPY	CYNOPS	PYRRHOGASTER	JAPANESE FIREBELLY	NEWT	LIV	105	NO	HK	HK	T	C	C	C	5/12/14	5/11/14	I	NY	Transship Discounts Ltd.
2014464411	PACB	PACHYTRITON	BREVIPES	TSITOU	NEWT	LIV	60	NO	HK	HK	T	C	C	C	5/12/14	5/11/14	I	NY	Transship Discounts Ltd.
2014465073	AMBT	AMBYSTOMA	TIGRINUM	TIGER	SALAMANDER	LIV	50	NO	US	IT	T	W	C	C	5/13/14	5/13/14	I	MI	TWO AMIGOS IMPORTS, INC.
2014465936	CYOR	CYNOPS	ORIENTALIS	CHINESE DWARF	NEWT	LIV	1,600	NO	HK	HK	T	C	C	C	5/14/14	5/15/14	I	TP	5-D TROPICAL, INC.
2014466401	CNP?	CYNOPS	SPECIES	FIREBELLY	NEWT	LIV	800	NO	HK	HK	T	C	C	C	5/15/14	5/15/14	I	AT	SUN PET LTD.
2014467812	CYOR	CYNOPS	ORIENTALIS	CHINESE DWARF	NEWT	LIV	500	NO	HK	HK	T	C	C	C	5/18/14	5/18/14	I	LA	DOLPHIN INTERNATIONAL
2014467821	CYOR	CYNOPS	ORIENTALIS	CHINESE DWARF	NEWT	LIV	325	NO	SG	SG	T	C	C	C	5/18/14	5/18/14	I	LA	PET-SIAM INTERNATIONAL
2014467846	CNP?	CYNOPS	SPECIES	FIREBELLY	NEWT	LIV	1,160	NO	CN	HK	T	W	C	C	5/18/14	5/18/14	I	LA	AQUA-NAUTIC SPECIALIST

2014467846	TRIT	TRITURUS	SPECIES		NEWT	LIV	80	NO	CN	HK	T	W	C	C	5/18/14	5/18/14	I	LA	AQUA-NAUTIC SPECIALIST
2014469409	CYOR	CYNOPS	ORIENTALIS	CHINESE DWARF	NEWT	LIV	1,600	NO	HK	HK	T	C	C	C	5/21/14	5/22/14	I	TP	5-D TROPICAL, INC.
2014469638	CYPY	CYNOPS	PYRRHOGASTER	JAPANESE FIREBELLY	NEWT	LIV	135	NO	HK	HK	T	C	C	C	5/21/14	5/18/14	I	NY	Transship Discounts Ltd.
2014469638	PACB	PACHYTRITON	BREVIPES	TSITOU	NEWT	LIV	30	NO	HK	HK	T	C	C	C	5/21/14	5/18/14	I	NY	Transship Discounts Ltd.
2014471016	CYOR	CYNOPS	ORIENTALIS	CHINESE DWARF	NEWT	LIV	300	NO	HK	HK	T	C	C	C	5/25/14	5/25/14	I	LA	DOLPHIN INTERNATIONAL
2014471064	CYOR	CYNOPS	ORIENTALIS	CHINESE DWARF	NEWT	LIV	375	NO	SG	SG	T	C	C	C	5/25/14	5/25/14	I	LA	PET-SIAM INTERNATIONAL
2014471075	CNP?	CYNOPS	SPECIES	FIREBELLY	NEWT	LIV	580	NO	CN	HK	T	W	C	C	5/25/14	5/25/14	I	LA	AQUA-NAUTIC SPECIALIST
2014471075	TRIT	TRITURUS	SPECIES		NEWT	LIV	90	NO	CN	HK	T	W	C	C	5/25/14	5/25/14	I	LA	AQUA-NAUTIC SPECIALIST
2014471280	CYPY	CYNOPS	PYRRHOGASTER	JAPANESE FIREBELLY	NEWT	LIV	135	NO	HK	HK	T	C	C	C	5/27/14	5/25/14	I	NY	Transship Discounts Ltd.
2014471280	PACB	PACHYTRITON	BREVIPES	TSITOU	NEWT	LIV	30	NO	HK	HK	T	C	C	C	5/27/14	5/25/14	I	NY	Transship Discounts Ltd.
2014474518	CYOR	CYNOPS	ORIENTALIS	CHINESE DWARF	NEWT	LIV	125	NO	HK	HK	T	C	C	C	6/1/14	6/1/14	I	LA	DOLPHIN INTERNATIONAL
2014474532	CYOR	CYNOPS	ORIENTALIS	CHINESE DWARF	NEWT	LIV	50	NO	SG	SG	T	C	C	C	6/1/14	6/1/14	I	LA	PET-SIAM INTERNATIONAL
2014475102	CYPY	CYNOPS	PYRRHOGASTER	JAPANESE FIREBELLY	NEWT	LIV	270	NO	HK	HK	T	C	C	C	6/2/14	6/1/14	I	NY	Transship Discounts Ltd.
2014478027	CYOR	CYNOPS	ORIENTALIS	CHINESE DWARF	NEWT	LIV	350	NO	HK	HK	T	C	C	C	6/8/14	6/8/14	I	LA	DOLPHIN INTERNATIONAL
2014478051	CYOR	CYNOPS	ORIENTALIS	CHINESE DWARF	NEWT	LIV	110	NO	SG	SG	T	C	C	C	6/8/14	6/8/14	I	LA	PET-SIAM INTERNATIONAL
2014478064	TRIT	TRITURUS	SPECIES		NEWT	LIV	90	NO	CN	HK	T	W	C	C	6/8/14	6/8/14	I	LA	AQUA-NAUTIC SPECIALIST
2014478064	CNP?	CYNOPS	SPECIES	FIREBELLY	NEWT	LIV	1,320	NO	CN	HK	T	W	C	C	6/8/14	6/8/14	I	LA	AQUA-NAUTIC SPECIALIST
2014478937	CYPY	CYNOPS	PYRRHOGASTER	JAPANESE FIREBELLY	NEWT	LIV	105	NO	HK	HK	T	C	C	C	6/10/14	6/8/14	I	NY	Transship Discounts Ltd.
2014480796	SASA	SALAMANDRA	SALAMANDRA	EUROPEAN FIRE	SALAMANDER	LIV	4	NO	DE	DE	T	C	C	C	6/12/14	6/12/14	I	MI	REPTILE INDUSTRIES, INC.
2014481695	CYOR	CYNOPS	ORIENTALIS	CHINESE DWARF	NEWT	LIV	250	NO	HK	HK	T	C	C	C	6/15/14	6/15/14	I	LA	DOLPHIN INTERNATIONAL
2014481739	TRIT	TRITURUS	SPECIES		NEWT	LIV	80	NO	CN	HK	T	W	C	C	6/15/14	6/15/14	I	LA	AQUA-NAUTIC SPECIALIST
2014481739	CNP?	CYNOPS	SPECIES	FIREBELLY	NEWT	LIV	1,920	NO	CN	HK	T	W	C	C	6/15/14	6/15/14	I	LA	AQUA-NAUTIC SPECIALIST
2014482351	CYPY	CYNOPS	PYRRHOGASTER	JAPANESE FIREBELLY	NEWT	LIV	105	NO	HK	HK	T	C	R	C	6/17/14	4/22/12	I	NY	Transship Discounts Ltd.
2014484036	TYKW	TYLOTOTRITON	KWEICHOWENSIS	KWEICHOW CROCODILE	NEWT	LIV	90	NO	HK	HK	T	C	C	C	6/19/14	6/19/14	I	LA	Bushmaster Reptiles Inc
2014484693	CYPY	CYNOPS	PYRRHOGASTER	JAPANESE FIREBELLY	NEWT	LIV	105	NO	HK	HK	T	C	C	C	6/20/14	6/15/14	I	NY	Transship Discounts Ltd.
2014485340	CNP?	CYNOPS	SPECIES	FIREBELLY	NEWT	LIV	140	NO	CI	HK	T	W	C	C	6/22/14	6/22/14	I	LA	AQUA-NAUTIC SPECIALIST
2014488878	CYOR	CYNOPS	ORIENTALIS	CHINESE DWARF	NEWT	LIV	125	NO	HK	HK	T	C	C	C	6/29/14	6/29/14	I	LA	DOLPHIN INTERNATIONAL
2014488913	CNP?	CYNOPS	SPECIES	FIREBELLY	NEWT	LIV	440	NO	CI	HK	T	W	C	C	6/29/14	6/29/14	I	LA	AQUA-NAUTIC SPECIALIST
2014488913	TRIT	TRITURUS	SPECIES		NEWT	LIV	40	NO	CI	HK	T	W	C	C	6/29/14	6/29/14	I	LA	AQUA-NAUTIC SPECIALIST
2014489290	PACB	PACHYTRITON	BREVIPES	TSITOU	NEWT	LIV	30	NO	HK	HK	T	C	C	C	6/30/14	6/29/14	I	NY	Transship Discounts Ltd.
2014489290	CYPY	CYNOPS	PYRRHOGASTER	JAPANESE FIREBELLY	NEWT	LIV	105	NO	HK	HK	T	C	C	C	6/30/14	6/29/14	I	NY	Transship Discounts Ltd.
2014491881	CYOR	CYNOPS	ORIENTALIS	CHINESE DWARF	NEWT	LIV	400	NO	SG	SG	T	C	C	C	7/6/14	7/6/14	I	LA	PET-SIAM INTERNATIONAL
2014491889	CNEW	CYNOPS	CYANURUS	CYAN	NEWT	LIV	50	NO	HK	HK	T	C	C	C	7/6/14	7/6/14	I	LA	DOLPHIN INTERNATIONAL
2014491889	CYOR	CYNOPS	ORIENTALIS	CHINESE DWARF	NEWT	LIV	500	NO	HK	HK	T	C	C	C	7/6/14	7/6/14	I	LA	DOLPHIN INTERNATIONAL
2014491992	PACB	PACHYTRITON	BREVIPES	TSITOU	NEWT	LIV	60	NO	HK	HK	T	C	C	C	7/7/14	7/6/14	I	NY	Transship Discounts Ltd.
2014491992	CYPY	CYNOPS	PYRRHOGASTER	JAPANESE FIREBELLY	NEWT	LIV	105	NO	HK	HK	T	C	C	C	7/7/14	7/6/14	I	NY	Transship Discounts Ltd.
2014495644	CYOR	CYNOPS	ORIENTALIS	CHINESE DWARF	NEWT	LIV	250	NO	HK	HK	T	C	C	C	7/13/14	7/13/14	I	LA	DOLPHIN INTERNATIONAL
2014495644	PACB	PACHYTRITON	BREVIPES	TSITOU	NEWT	LIV	100	NO	HK	HK	T	C	C	C	7/13/14	7/13/14	I	LA	DOLPHIN INTERNATIONAL
2014495693	CYOR	CYNOPS	ORIENTALIS	CHINESE DWARF	NEWT	LIV	100	NO	SG	SG	T	C	C	C	7/13/14	7/13/14	I	LA	PET-SIAM INTERNATIONAL
2014495703	TRIT	TRITURUS	SPECIES		NEWT	LIV	40	NO	CI	HK	T	W	C	C	7/13/14	7/13/14	I	LA	AQUA-NAUTIC SPECIALIST
2014495703	CNP?	CYNOPS	SPECIES	FIREBELLY	NEWT	LIV	1,020	NO	CI	HK	T	W	C	C	7/13/14	7/13/14	I	LA	AQUA-NAUTIC SPECIALIST
2014496615	CYPY	CYNOPS	PYRRHOGASTER	JAPANESE FIREBELLY	NEWT	LIV	210	NO	HK	HK	T	C	C	C	7/15/14	7/13/14	I	NY	Transship Discounts Ltd.
2014496615	PACB	PACHYTRITON	BREVIPES	TSITOU	NEWT	LIV	30	NO	HK	HK	T	C	C	C	7/15/14	7/13/14	I	NY	Transship Discounts Ltd.
2014497142	TYKW	TYLOTOTRITON	KWEICHOWENSIS	KWEICHOW CROCODILE	NEWT	LIV	128	NO	CN	HK	T	W	C	C	7/15/14	7/14/14	I	SF	WONDERFUL WORLD PET SUPPLIERS INC
2014497142	TYSH	TYLOTOTRITON	SHANJING	MANDARIN	NEWT	LIV	160	NO	CN	HK	T	W	C	C	7/15/14	7/14/14	I	SF	WONDERFUL WORLD PET SUPPLIERS INC
2014498068	CYOR	CYNOPS	ORIENTALIS	CHINESE DWARF	NEWT	LIV	1,600	NO	HK	HK	T	C	C	C	7/16/14	7/17/14	I	TP	5-D TROPICAL, INC.
2014499735	PACB	PACHYTRITON	BREVIPES	TSITOU	NEWT	LIV	50	NO	HK	HK	T	C	C	C	7/20/14	7/20/14	I	LA	DOLPHIN INTERNATIONAL
2014499754	CYOR	CYNOPS	ORIENTALIS	CHINESE DWARF	NEWT	LIV	35	NO	SG	SG	T	C	C	C	7/20/14	7/20/14	I	LA	PET-SIAM INTERNATIONAL
2014499789	TRIT	TRITURUS	SPECIES		NEWT	LIV	40	NO	CN	HK	T	W	C	C	7/20/14	7/20/14	I	LA	AQUA-NAUTIC SPECIALIST
2014499789	CNP?	CYNOPS	SPECIES	FIREBELLY	NEWT	LIV	140	NO	CN	HK	T	W	C	C	7/20/14	7/20/14	I	LA	AQUA-NAUTIC SPECIALIST
2014503265	TRIT	TRITURUS	SPECIES		NEWT	LIV	40	NO	CN	HK	T	W	C	C	7/27/14	7/27/14	I	LA	AQUA-NAUTIC SPECIALIST
2014503265	CNP?	CYNOPS	SPECIES	FIREBELLY	NEWT	LIV	1,760	NO	CN	HK	T	W	C	C	7/27/14	7/27/14	I	LA	AQUA-NAUTIC SPECIALIST
2014504351	CNEW	CYNOPS	CYANURUS	CYAN	NEWT	LIV	60	NO	HK	HK	T	C	C	C	7/29/14	7/27/14	I	NY	Transship Discounts Ltd.
2014504351	PACB	PACHYTRITON	BREVIPES	TSITOU	NEWT	LIV	90	NO	HK	HK	T	C	C	C	7/29/14	7/27/14	I	NY	Transship Discounts Ltd.
2014504351	CYPY	CYNOPS	PYRRHOGASTER	JAPANESE FIREBELLY	NEWT	LIV	262	NO	HK	HK	T	C	C	C	7/29/14	7/27/14	I	NY	Transship Discounts Ltd.
2014505101	CYOR	CYNOPS	ORIENTALIS	CHINESE DWARF	NEWT	LIV	1,600	NO	HK	HK	T	C	C	C	7/30/14	7/31/14	I	TP	5-D TROPICAL, INC.
2014506819	CYOR	CYNOPS	ORIENTALIS	CHINESE DWARF	NEWT	LIV	125	NO	HK	HK	T	C	C	C	8/3/14	8/3/14	I	LA	DOLPHIN INTERNATIONAL
2014506854	CNP?	CYNOPS	SPECIES	FIREBELLY	NEWT	LIV	280	NO	HK	HK	T	W	C	C	8/3/14	8/3/14	I	LA	AQUA-NAUTIC SPECIALIST
2014506965	PACB	PACHYTRITON	BREVIPES	TSITOU	NEWT	LIV	30	NO	HK	HK	T	C	C	C	8/4/14	8/3/14	I	NY	Transship Discounts Ltd.
2014511327	CYOR	CYNOPS	ORIENTALIS	CHINESE DWARF	NEWT	LIV	425	NO	SG	SG	T	C	C	C	8/10/14	8/10/14	I	LA	PET-SIAM INTERNATIONAL

2014511329	CYOR	CYNOPS	ORIENTALIS	CHINESE DWARF	NEWT	LIV	250	NO	HK	HK	T	C	C	C	8/10/14	8/10/14	I	LA	DOLPHIN INTERNATIONAL
2014511356	CNP?	CYNOPS	SPECIES	FIREBELLY	NEWT	LIV	720	NO	CN	HK	T	W	C	C	8/10/14	8/10/14	I	LA	AQUA-NAUTIC SPECIALIST
2014511356	TRIT	TRITURUS	SPECIES		NEWT	LIV	40	NO	CN	HK	T	W	C	C	8/10/14	8/10/14	I	LA	AQUA-NAUTIC SPECIALIST
2014511662	TYKW	TYLOTOTRITON	KWEICHOWENSIS	KWEICHOW CROCODILE	NEWT	LIV	20	NO	HK	HK	T	W	C	C	8/11/14	8/11/14	I	LA	CALIFORNIA ZOOLOGICAL SUPPLY
2014511662	TYL?	TYLOTOTRITON	SPECIES	CROCODILE	NEWT	LIV	20	NO	HK	HK	T	W	C	C	8/11/14	8/11/14	I	LA	CALIFORNIA ZOOLOGICAL SUPPLY
2014512807	CNP?	CYNOPS	SPECIES	FIREBELLY	NEWT	LIV	50	NO	HK	HK	T	C	C	C	8/13/14	8/10/14	I	NY	Transship Discounts Ltd.
2014513339	CYOR	CYNOPS	ORIENTALIS	CHINESE DWARF	NEWT	LIV	3,200	NO	HK	HK	T	C	C	C	8/13/14	8/14/14	I	TP	5-D TROPICAL, INC.
2014513600	CNP?	CYNOPS	SPECIES	FIREBELLY	NEWT	LIV	800	NO	HK	HK	T	C	C	C	8/14/14	8/14/14	I	AT	SUN PET LTD.
2014514830	PACB	PACHYTRITON	BREVIPES	TSITOU	NEWT	LIV	100	NO	HK	HK	T	C	C	C	8/17/14	8/17/14	I	LA	DOLPHIN INTERNATIONAL
2014514869	CYOR	CYNOPS	ORIENTALIS	CHINESE DWARF	NEWT	LIV	150	NO	SG	SG	T	C	C	C	8/17/14	8/17/14	I	LA	PET-SIAM INTERNATIONAL
2014514897	CNP?	CYNOPS	SPECIES	FIREBELLY	NEWT	LIV	1,200	NO	HK	HK	T	W	C	C	8/17/14	8/17/14	I	LA	AQUA-NAUTIC SPECIALIST
2014514897	TRIT	TRITURUS	SPECIES		NEWT	LIV	90	NO	HK	HK	T	W	C	C	8/17/14	8/17/14	I	LA	AQUA-NAUTIC SPECIALIST
2014517314	CLUS	CHIOGLOSSA	LUSITANICA	GOLD-STRIPED	SALAMANDER	LIV	10	NO	ES	ES	S	W	C	C	8/21/14	8/19/14	I	AT	UNIVERSITY OF SOUTH FLORIDA, DPT OF INTEGRATIVE BIOLOGY
2014518349	CNEW	CYNOPS	CYANURUS	CYAN	NEWT	LIV	50	NO	HK	HK	T	C	C	C	8/24/14	8/24/14	I	LA	DOLPHIN INTERNATIONAL
2014518349	CYOR	CYNOPS	ORIENTALIS	CHINESE DWARF	NEWT	LIV	50	NO	HK	HK	T	C	C	C	8/24/14	8/24/14	I	LA	DOLPHIN INTERNATIONAL
2014518349	PACB	PACHYTRITON	BREVIPES	TSITOU	NEWT	LIV	50	NO	HK	HK	T	C	C	C	8/24/14	8/24/14	I	LA	DOLPHIN INTERNATIONAL
2014518366	CNP?	CYNOPS	SPECIES	FIREBELLY	NEWT	LIV	1,620	NO	HK	HK	T	W	C	C	8/24/14	8/24/14	I	LA	AQUA-NAUTIC SPECIALIST
2014518366	TRIT	TRITURUS	SPECIES		NEWT	LIV	40	NO	HK	HK	T	W	C	C	8/24/14	8/24/14	I	LA	AQUA-NAUTIC SPECIALIST
2014519770	CYOR	CYNOPS	ORIENTALIS	CHINESE DWARF	NEWT	LIV	1,600	NO	HK	HK	T	C	C	C	8/27/14	8/28/14	I	TP	5-D TROPICAL, INC.
2014521559	CYPY	CYNOPS	PYRRHOGASTER	JAPANESE FIREBELLY	NEWT	LIV	158	NO	HK	HK	T	C	C	C	8/31/14	8/24/14	I	NY	Transship Discounts Ltd.
2014521607	CNEW	CYNOPS	CYANURUS	CYAN	NEWT	LIV	100	NO	HK	HK	T	C	C	C	8/31/14	8/31/14	I	LA	DOLPHIN INTERNATIONAL
2014521607	CYOR	CYNOPS	ORIENTALIS	CHINESE DWARF	NEWT	LIV	625	NO	HK	HK	T	C	C	C	8/31/14	8/31/14	I	LA	DOLPHIN INTERNATIONAL
2014521607	PACB	PACHYTRITON	BREVIPES	TSITOU	NEWT	LIV	50	NO	HK	HK	T	C	C	C	8/31/14	8/31/14	I	LA	DOLPHIN INTERNATIONAL
2014521625	CNP?	CYNOPS	SPECIES	FIREBELLY	NEWT	LIV	1,040	NO	HK	HK	T	W	C	C	8/31/14	8/31/14	I	LA	AQUA-NAUTIC SPECIALIST
2014522777	CYOR	CYNOPS	ORIENTALIS	CHINESE DWARF	NEWT	LIV	1,970	NO	HK	HK	T	C	C	C	9/3/14	9/4/14	I	TP	5-D TROPICAL, INC.
2014525187	CYOR	CYNOPS	ORIENTALIS	CHINESE DWARF	NEWT	LIV	305	NO	SG	SG	T	C	C	C	9/7/14	9/7/14	I	LA	PET-SIAM INTERNATIONAL
2014525188	CNP?	CYNOPS	SPECIES	FIREBELLY	NEWT	LIV	420	NO	TH	HK	T	W	C	C	9/7/14	9/7/14	I	LA	AQUA-NAUTIC SPECIALIST
2014525188	TRIT	TRITURUS	SPECIES		NEWT	LIV	80	NO	TH	HK	T	W	C	C	9/7/14	9/7/14	I	LA	AQUA-NAUTIC SPECIALIST
2014525193	CNEW	CYNOPS	CYANURUS	CYAN	NEWT	LIV	50	NO	HK	HK	T	C	C	C	9/7/14	9/7/14	I	LA	DOLPHIN INTERNATIONAL
2014525193	CYOR	CYNOPS	ORIENTALIS	CHINESE DWARF	NEWT	LIV	375	NO	HK	HK	T	C	C	C	9/7/14	9/7/14	I	LA	DOLPHIN INTERNATIONAL
2014525928	CYPY	CYNOPS	PYRRHOGASTER	JAPANESE FIREBELLY	NEWT	LIV	105	NO	HK	HK	T	C	C	C	9/9/14	9/7/14	I	NY	Transship Discounts Ltd.
2014525928	PACB	PACHYTRITON	BREVIPES	TSITOU	NEWT	LIV	60	NO	HK	HK	T	C	C	C	9/9/14	9/7/14	I	NY	Transship Discounts Ltd.
2014528987	CNEW	CYNOPS	CYANURUS	CYAN	NEWT	LIV	50	NO	HK	HK	T	C	C	C	9/14/14	9/14/14	I	LA	DOLPHIN INTERNATIONAL
2014529000	CYOR	CYNOPS	ORIENTALIS	CHINESE DWARF	NEWT	LIV	200	NO	SG	SG	T	C	C	C	9/14/14	9/14/14	I	LA	PET-SIAM INTERNATIONAL
2014529026	TRIT	TRITURUS	SPECIES		NEWT	LIV	40	NO	TH	HK	T	W	C	C	9/14/14	9/14/14	I	LA	AQUA-NAUTIC SPECIALIST
2014529622	CYPY	CYNOPS	PYRRHOGASTER	JAPANESE FIREBELLY	NEWT	LIV	262	NO	HK	HK	T	C	C	C	9/15/14	9/14/14	I	NY	Transship Discounts Ltd.
2014530641	SAKE	SALAMANDRELLA	KEYSERLINGII	SIBERIAN	SALAMANDER	LIV	2	NO	DE	DE	T	C	C	C	9/15/14	9/15/14	I	AT	TIM TYTLE LIZARDS
2014531321	CNP?	CYNOPS	SPECIES	FIREBELLY	NEWT	LIV	500	NO	HK	HK	T	C	C	C	9/18/14	9/18/14	I	AT	SUN PET LTD.
2014532787	CYOR	CYNOPS	ORIENTALIS	CHINESE DWARF	NEWT	LIV	550	NO	HK	HK	T	C	C	C	9/21/14	9/21/14	I	LA	DOLPHIN INTERNATIONAL
2014532787	PACB	PACHYTRITON	BREVIPES	TSITOU	NEWT	LIV	50	NO	HK	HK	T	C	C	C	9/21/14	9/21/14	I	LA	DOLPHIN INTERNATIONAL
2014532840	CNP?	CYNOPS	SPECIES	FIREBELLY	NEWT	LIV	600	NO	HK	HK	T	W	C	C	9/21/14	9/21/14	I	LA	AQUA-NAUTIC SPECIALIST
2014533277	SASA	SALAMANDRA	SALAMANDRA	EUROPEAN FIRE	SALAMANDER	LIV	5	NO	DE	DE	T	C	C	C	9/22/14	9/22/14	I	MI	REPTILE INDUSTRIES, INC.
2014534317	SASA	SALAMANDRA	SALAMANDRA	EUROPEAN FIRE	SALAMANDER	LIV	950	NO	SI	SI	T	C	C	C	9/24/14	9/23/14	I	MI	EPSILON GLOBAL TRADE INC
2014536126	CYPY	CYNOPS	PYRRHOGASTER	JAPANESE FIREBELLY	NEWT	LIV	105	NO	HK	HK	T	C	C	C	9/26/14	9/21/14	I	NY	Transship Discounts Ltd.
2014536198	NEUC	NEURERGUS	CROCATUS	LAKE URMIA	NEWT	LIV	10	NO	DE	DE	T	C	C	C	9/26/14	9/26/14	I	MI	NAME REMOVED
2014536488	CYOR	CYNOPS	ORIENTALIS	CHINESE DWARF	NEWT	LIV	200	NO	HK	HK	T	C	C	C	9/28/14	9/28/14	I	LA	DOLPHIN INTERNATIONAL
2014536524	CYOR	CYNOPS	ORIENTALIS	CHINESE DWARF	NEWT	LIV	25	NO	SG	SG	T	C	C	C	9/28/14	9/28/14	I	LA	PET-SIAM INTERNATIONAL
2014536525	CNP?	CYNOPS	SPECIES	FIREBELLY	NEWT	LIV	12,000	NO	HK	HK	T	W	C	C	9/28/14	9/28/14	I	LA	AQUA-NAUTIC SPECIALIST
2014538109	CYPY	CYNOPS	PYRRHOGASTER	JAPANESE FIREBELLY	NEWT	LIV	345	NO	HK	HK	T	C	C	C	9/30/14	9/28/14	I	NY	Transship Discounts Ltd.
2014538109	PACB	PACHYTRITON	BREVIPES	TSITOU	NEWT	LIV	30	NO	HK	HK	T	C	C	C	9/30/14	9/28/14	I	NY	Transship Discounts Ltd.
2014540034	CYOR	CYNOPS	ORIENTALIS	CHINESE DWARF	NEWT	LIV	1,848	NO	HK	HK	T	C	C	C	10/2/14	10/2/14	I	TP	5-D TROPICAL, INC.
2014541508	CYOR	CYNOPS	ORIENTALIS	CHINESE DWARF	NEWT	LIV	425	NO	HK	HK	T	C	C	C	10/5/14	10/5/14	I	LA	DOLPHIN INTERNATIONAL
2014541508	PACB	PACHYTRITON	BREVIPES	TSITOU	NEWT	LIV	100	NO	HK	HK	T	C	C	C	10/5/14	10/5/14	I	LA	DOLPHIN INTERNATIONAL
2014541572	CNP?	CYNOPS	SPECIES	FIREBELLY	NEWT	LIV	880	NO	HK	HK	T	W	C	C	10/5/14	10/5/14	I	LA	AQUA-NAUTIC SPECIALIST
2014541572	TRIT	TRITURUS	SPECIES		NEWT	LIV	40	NO	HK	HK	T	W	C	C	10/5/14	10/5/14	I	LA	AQUA-NAUTIC SPECIALIST
2014545212	CYOR	CYNOPS	ORIENTALIS	CHINESE DWARF	NEWT	LIV	600	NO	HK	HK	T	C	C	C	10/12/14	10/12/14	I	LA	DOLPHIN INTERNATIONAL
2014545212	CNEW	CYNOPS	CYANURUS	CYAN	NEWT	LIV	50	NO	HK	HK	T	C	C	C	10/12/14	10/12/14	I	LA	DOLPHIN INTERNATIONAL
2014545255	CNP?	CYNOPS	SPECIES	FIREBELLY	NEWT	LIV	300	NO	HK	HK	T	W	C	C	10/12/14	10/12/14	I	LA	AQUA-NAUTIC SPECIALIST
2014545255	TRIT	TRITURUS	SPECIES		NEWT	LIV	120	NO	HK	HK	T	W	C	C	10/12/14	10/12/14	I	LA	AQUA-NAUTIC SPECIALIST

2014546904	CYOR	CYNOPS	ORIENTALIS	CHINESE DWARF	NEWT	LIV	300	NO	HK	HK	T	C	C	C	10/15/14	10/16/14	I	TP	5-D TROPICAL, INC.
2014548843	CYOR	CYNOPS	ORIENTALIS	CHINESE DWARF	NEWT	LIV	475	NO	HK	HK	T	C	C	C	10/19/14	10/19/14	I	LA	DOLPHIN INTERNATIONAL
2014548883	CYOR	CYNOPS	ORIENTALIS	CHINESE DWARF	NEWT	LIV	360	NO	SG	SG	T	C	C	C	10/19/14	10/19/14	I	LA	PET-SIAM INTERNATIONAL
2014548897	CNP?	CYNOPS	SPECIES	FIREBELLY	NEWT	LIV	440	NO	CN	HK	T	W	C	C	10/19/14	10/19/14	I	LA	AQUA-NAUTIC SPECIALIST
2014549007	NEUC	NEURERGUS	CROCATUS	LAKE URMIA	NEWT	LIV	12	NO	DE	DE	T	C	C	C	10/20/14	10/17/14	I	MI	TWO AMIGOS IMPORTS, INC.
2014553218	CYOR	CYNOPS	ORIENTALIS	CHINESE DWARF	NEWT	LIV	50	NO	HK	HK	T	C	C	C	10/26/14	10/26/14	I	LA	DOLPHIN INTERNATIONAL
2014553245	CNP?	CYNOPS	SPECIES	FIREBELLY	NEWT	LIV	420	NO	CN	HK	T	W	C	C	10/26/14	10/26/14	I	LA	AQUA-NAUTIC SPECIALIST
2014553248	CYOR	CYNOPS	ORIENTALIS	CHINESE DWARF	NEWT	LIV	350	NO	SG	SG	T	C	C	C	10/26/14	10/26/14	I	LA	PET-SIAM INTERNATIONAL
2014553982	TRIT	TRITURUS	SPECIES		NEWT	LIV	110	NO	NL	NL	T	W	C	C	10/27/14	10/27/14	I	LA	EPSILON GLOBAL TRADE INC
2014554535	CYPY	CYNOPS	PYRRHOGASTER	JAPANESE FIREBELLY	NEWT	LIV	105	NO	HK	HK	T	C	C	C	10/28/14	10/26/14	I	NY	Transship Discounts Ltd.
2014554535	PACB	PACHYTRITON	BREVIPES	TSITOU	NEWT	LIV	30	NO	HK	HK	T	C	C	C	10/28/14	10/26/14	I	NY	Transship Discounts Ltd.
2014555483	PDE?	PSEUDOEURYCEA	SPECIES	FALSE BROOK	SALAMANDER	LIV	40	NO	MX	MX	S	W	C	C	10/29/14	6/17/14	I	MI	University of South Florida
2014555483	BOG?	BOLITOGLOSSA	SPECIES	MUSHROOMTONGUE	SALAMANDER	LIV	30	NO	MX	MX	S	W	C	C	10/29/14	6/17/14	I	MI	University of South Florida
2014555761	CYPY	CYNOPS	PYRRHOGASTER	JAPANESE FIREBELLY	NEWT	LIV	80	NO	HK	HK	T	C	C	C	10/30/14	10/19/14	I	NY	Transship Discounts Ltd.
2014557424	CNEW	CYNOPS	CYANURUS	CYAN	NEWT	LIV	50	NO	HK	HK	T	C	C	C	11/2/14	11/2/14	I	LA	DOLPHIN INTERNATIONAL
2014557435	CNP?	CYNOPS	SPECIES	FIREBELLY	NEWT	LIV	1,940	NO	CN	HK	T	W	C	C	11/2/14	11/2/14	I	LA	AQUA-NAUTIC SPECIALIST
2014559543	CYOR	CYNOPS	ORIENTALIS	CHINESE DWARF	NEWT	LIV	1,960	NO	HK	HK	T	C	C	C	11/5/14	11/6/14	I	TP	5-D TROPICAL, INC.
2014561417	CYOR	CYNOPS	ORIENTALIS	CHINESE DWARF	NEWT	LIV	600	NO	HK	HK	T	C	C	C	11/9/14	11/9/14	I	LA	DOLPHIN INTERNATIONAL
2014561468	CNP?	CYNOPS	SPECIES	FIREBELLY	NEWT	LIV	300	NO	CN	HK	T	W	C	C	11/9/14	11/9/14	I	LA	AQUA-NAUTIC SPECIALIST
2014565180	CYOR	CYNOPS	ORIENTALIS	CHINESE DWARF	NEWT	LIV	125	NO	HK	HK	T	C	C	C	11/16/14	11/16/14	I	LA	DOLPHIN INTERNATIONAL
2014565180	CNEW	CYNOPS	CYANURUS	CYAN	NEWT	LIV	50	NO	HK	HK	T	C	C	C	11/16/14	11/16/14	I	LA	DOLPHIN INTERNATIONAL
2014565180	PACB	PACHYTRITON	BREVIPES	TSITOU	NEWT	LIV	100	NO	HK	HK	T	C	C	C	11/16/14	11/16/14	I	LA	DOLPHIN INTERNATIONAL
2014565197	CNP?	CYNOPS	SPECIES	FIREBELLY	NEWT	LIV	420	NO	CN	HK	T	W	C	C	11/16/14	11/16/14	I	LA	AQUA-NAUTIC SPECIALIST
2014565286	CYPY	CYNOPS	PYRRHOGASTER	JAPANESE FIREBELLY	NEWT	LIV	52	NO	HK	HK	T	C	C	C	11/17/14	11/2/14	I	NY	Transship Discounts Ltd.
2014566087	CNEW	CYNOPS	CYANURUS	CYAN	NEWT	LIV	10	NO	HK	HK	T	W	C	C	11/17/14	11/17/14	I	LA	CALIFORNIA ZOOLOGICAL SUPPLY
2014566087	PCHI	PARAMESOTRITON	CHINENSIS	CHINESE WARTY	NEWT	LIV	200	NO	HK	HK	T	W	C	C	11/17/14	11/17/14	I	LA	CALIFORNIA ZOOLOGICAL SUPPLY
2014567172	CYPY	CYNOPS	PYRRHOGASTER	JAPANESE FIREBELLY	NEWT	LIV	30	NO	HK	HK	T	C	C	C	11/18/14	10/5/14	I	NY	Transship Discounts Ltd.
2014567628	CYPY	CYNOPS	PYRRHOGASTER	JAPANESE FIREBELLY	NEWT	LIV	210	NO	HK	HK	T	C	C	C	11/19/14	11/16/14	I	NY	Transship Discounts Ltd.
2014567628	CNEW	CYNOPS	CYANURUS	CYAN	NEWT	LIV	30	NO	HK	HK	T	C	C	C	11/19/14	11/16/14	I	NY	Transship Discounts Ltd.
2014569823	TYTA	TYLOTOTRITON	TALIANGENSIS	PUSAKANG CROCODILE	NEWT	LIV	16	NO	HK	HK	T	W	C	C	11/21/14	11/21/14	I	MI	TWO AMIGOS IMPORTS, INC.
2014569823	PCHI	PARAMESOTRITON	CHINENSIS	CHINESE WARTY	NEWT	LIV	100	NO	HK	HK	T	W	C	C	11/21/14	11/21/14	I	MI	TWO AMIGOS IMPORTS, INC.
2014569823	PLAB	PACHYTRITON	LABIATUS	UNTERSTEIN'S	NEWT	LIV	200	NO	HK	HK	T	W	C	C	11/21/14	11/21/14	I	MI	TWO AMIGOS IMPORTS, INC.
2014569823	CYOR	CYNOPS	ORIENTALIS	CHINESE DWARF	NEWT	LIV	300	NO	HK	HK	T	W	C	C	11/21/14	11/21/14	I	MI	TWO AMIGOS IMPORTS, INC.
2014570181	CYOR	CYNOPS	ORIENTALIS	CHINESE DWARF	NEWT	LIV	350	NO	HK	HK	T	C	C	C	11/23/14	11/23/14	I	LA	DOLPHIN INTERNATIONAL
2014570181	CNEW	CYNOPS	CYANURUS	CYAN	NEWT	LIV	100	NO	HK	HK	T	C	C	C	11/23/14	11/23/14	I	LA	DOLPHIN INTERNATIONAL
2014570222	CNP?	CYNOPS	SPECIES	FIREBELLY	NEWT	LIV	880	NO	CN	HK	T	W	C	C	11/23/14	11/23/14	I	LA	AQUA-NAUTIC SPECIALIST
2014570222	TRIT	TRITURUS	SPECIES		NEWT	LIV	40	NO	CN	HK	T	W	C	C	11/23/14	11/23/14	I	LA	AQUA-NAUTIC SPECIALIST
2014570799	PACB	PACHYTRITON	BREVIPES	TSITOU	NEWT	LIV	30	NO	HK	HK	T	C	C	C	11/24/14	11/23/14	I	NY	Transship Discounts Ltd.
2014570799	CYPY	CYNOPS	PYRRHOGASTER	JAPANESE FIREBELLY	NEWT	LIV	165	NO	HK	HK	T	C	C	C	11/24/14	11/23/14	I	NY	Transship Discounts Ltd.
2014576103	CYPY	CYNOPS	PYRRHOGASTER	JAPANESE FIREBELLY	NEWT	LIV	30	NO	HK	HK	T	C	C	C	12/3/14	11/30/14	I	NY	Transship Discounts Ltd.
2014576362	CYOR	CYNOPS	ORIENTALIS	CHINESE DWARF	NEWT	LIV	1,600	NO	HK	HK	T	C	C	C	12/3/14	12/4/14	I	TP	5-D TROPICAL, INC.
2014578436	CNEW	CYNOPS	CYANURUS	CYAN	NEWT	LIV	50	NO	HK	HK	T	C	C	C	12/7/14	12/7/14	I	LA	DOLPHIN INTERNATIONAL
2014578451	CYOR	CYNOPS	ORIENTALIS	CHINESE DWARF	NEWT	LIV	135	NO	SG	SG	T	C	C	C	12/7/14	12/7/14	I	LA	PET-SIAM INTERNATIONAL
2014578477	CNP?	CYNOPS	SPECIES	FIREBELLY	NEWT	LIV	880	NO	CN	HK	T	W	C	C	12/7/14	12/7/14	I	LA	AQUA-NAUTIC SPECIALIST
2014579971	CYPY	CYNOPS	PYRRHOGASTER	JAPANESE FIREBELLY	NEWT	LIV	140	NO	HK	HK	T	C	C	C	12/9/14	12/7/14	I	NY	Transship Discounts Ltd.
2014580699	NRR?	NEURERGUS	SPECIES		NEWT	LIV	12	NO	DE	DE	T	C	C	C	12/10/14	12/9/14	I	MI	TWO AMIGOS IMPORTS, INC.
2014583003	CYOR	CYNOPS	ORIENTALIS	CHINESE DWARF	NEWT	LIV	425	NO	HK	HK	T	C	C	C	12/14/14	12/13/14	I	LA	DOLPHIN INTERNATIONAL
2014583032	CNP?	CYNOPS	SPECIES	FIREBELLY	NEWT	LIV	280	NO	CN	HK	T	W	C	C	12/14/14	12/14/14	I	LA	AQUA-NAUTIC SPECIALIST
2014583194	CYPY	CYNOPS	PYRRHOGASTER	JAPANESE FIREBELLY	NEWT	LIV	82	NO	HK	HK	T	C	C	C	12/15/14	12/14/14	I	NY	Transship Discounts Ltd.
2014583194	PACB	PACHYTRITON	BREVIPES	TSITOU	NEWT	LIV	30	NO	HK	HK	T	C	C	C	12/15/14	12/14/14	I	NY	Transship Discounts Ltd.
2014584677	SASA	SALAMANDRA	SALAMANDRA	EUROPEAN FIRE	SALAMANDER	LIV	10	NO	DE	DE	T	C	C	C	12/16/14	12/15/14	I	SF	Exemptions 6 and 7(C)
2014584677	SAL?	SALAMANDRA	SPECIES	FIRE	SALAMANDER	LIV	6	NO	DE	DE	T	C	C	C	12/16/14	12/15/14	I	SF	Exemptions 6 and 7(C)
2014585759	TRIT	TRITURUS	SPECIES		NEWT	LIV	156	NO	NL	NL	T	W	C	C	12/17/14	12/17/14	I	LA	EPSILON GLOBAL TRADE INC
2014585770	CYOR	CYNOPS	ORIENTALIS	CHINESE DWARF	NEWT	LIV	2,004	NO	HK	HK	T	C	C	C	12/17/14	12/18/14	I	TP	5-D TROPICAL, INC.
2014588571	CYOR	CYNOPS	ORIENTALIS	CHINESE DWARF	NEWT	LIV	250	NO	HK	HK	T	C	C	C	12/21/14	12/20/14	I	LA	DOLPHIN INTERNATIONAL
2014588601	CNP?	CYNOPS	SPECIES	FIREBELLY	NEWT	LIV	1,640	NO	CN	HK	T	W	C	C	12/21/14	12/21/14	I	LA	AQUA-NAUTIC SPECIALIST
2014590961	PACB	PACHYTRITON	BREVIPES	TSITOU	NEWT	LIV	50	NO	HK	HK	T	C	C	C	12/28/14	12/27/14	I	LA	DOLPHIN INTERNATIONAL
2014590972	CNP?	CYNOPS	SPECIES	FIREBELLY	NEWT	LIV	300	NO	CN	HK	T	W	C	C	12/28/14	12/28/14	I	LA	AQUA-NAUTIC SPECIALIST
2014592168	PACB	PACHYTRITON	BREVIPES	TSITOU	NEWT	LIV	30	NO	HK	HK	T	C	C	C	12/30/14	12/21/14	I	NY	Transship Discounts Ltd.
2014592168	CYPY	CYNOPS	PYRRHOGASTER	JAPANESE FIREBELLY	NEWT	LIV	30	NO	HK	HK	T	C	C	C	12/30/14	12/21/14	I	NY	Transship Discounts Ltd.

2014592183	CNEW	CYNOPS	CYANURUS	CYAN	NEWT	LIV	60	NO	HK	HK	T	C	C	C	12/30/14	12/28/14	I	NY	Transship Discounts Ltd.
2014592183	CYPY	CYNOPS	PYRRHOGASTER	JAPANESE FIREBELLY	NEWT	LIV	210	NO	HK	HK	T	C	C	C	12/30/14	12/28/14	I	NY	Transship Discounts Ltd.
